# A Cataract-Causing Mutation in the TRPM3 Cation Channel Disrupts Calcium Dynamics in the Lens

**DOI:** 10.3390/cells13030257

**Published:** 2024-01-30

**Authors:** Yuefang Zhou, Thomas M. Bennett, Philip A. Ruzycki, Zhaohua Guo, Yu-Qing Cao, Mohammad Shahidullah, Nicholas A. Delamere, Alan Shiels

**Affiliations:** 1 Department of Ophthalmology and Visual Sciences, Washington University School of Medicine, St. Louis, MO 63110, USA; 2 Department of Anesthesiology and Washington University Pain Center, Washington University School of Medicine, St. Louis, MO 63110, USA; 3 Department of Physiology, University of Arizona College of Medicine, Tucson, AZ 85724, USA

**Keywords:** lens, cataract, TRPM3, calcium, gene expression, miR-204/211

## Abstract

TRPM3 belongs to the melastatin sub-family of transient receptor potential (TRPM) cation channels and has been shown to function as a steroid-activated, heat-sensitive calcium ion (Ca^2+^) channel. A missense substitution (p.I65M) in the TRPM3 gene of humans (*TRPM3*) and mice (*Trpm3*) has been shown to underlie an inherited form of early-onset, progressive cataract. Here, we model the pathogenetic effects of this cataract-causing mutation using ‘knock-in’ mutant mice and human cell lines. *Trpm3* and its intron-hosted micro-RNA gene (*Mir204*) were strongly co-expressed in the lens epithelium and other non-pigmented and pigmented ocular epithelia. Homozygous *Trpm3*-mutant lenses displayed elevated cytosolic Ca^2+^ levels and an imbalance of sodium (Na^+^) and potassium (K^+^) ions coupled with increased water content. Homozygous *TRPM3*-mutant human lens epithelial (HLE-B3) cell lines and *Trpm3*-mutant lenses exhibited increased levels of phosphorylated mitogen-activated protein kinase 1/extracellular signal-regulated kinase 2 (MAPK1/ERK2/p42) and MAPK3/ERK1/p44. Mutant TRPM3-M65 channels displayed an increased sensitivity to external Ca^2+^ concentration and an altered dose response to pregnenolone sulfate (PS) activation. *Trpm3*-mutant lenses shared the downregulation of genes involved in insulin/peptide secretion and the upregulation of genes involved in Ca^2+^ dynamics. By contrast, *Trpm3*-deficient lenses did not replicate the pathophysiological changes observed in *Trpm3*-mutant lenses. Collectively, our data suggest that a cataract-causing substitution in the TRPM3 cation channel elicits a deleterious gain-of-function rather than a loss-of-function mechanism in the lens.

## 1. Introduction

First discovered in a spontaneous, light-sensitive, retinal mutant strain of the fruit fly *D. melanogaster*, the transient receptor potential (TRP) superfamily of cation channels serve as polymodal sensors in myriad physiological processes, including the perception of light, temperature, pressure, and pain—rendering TRP channels therapeutic targets of interest for human disease [1,2,3,4,5]. Of the 28 mammalian (27 human) TRP channels, which are divided into six sequence-related sub-families, eight belong to the melastatin sub-family (TRPM1-8) that share homology with the charter member TRPM1 (melastatin-1) [6]. In addition to serving as a diagnostic and prognostic marker for primary cutaneous metastatic melanoma [7], mutations in the TRPM1 gene underlie several inherited eye disorders in humans, including night-blindness (nyctalopia), high myopia, and involuntary eye movements (strabismus, nystagmus), along with nyctalopia and leopard complex coat-spotting in Appaloosa horses [8,9,10,11]. TRPM3 (melastatin-2), which shares most homology with TRPM1 (~57% amino acid Identity), has been shown to function as a calcium ion (Ca^2+^) channel with permeability to other cations influenced by extensive alternative splicing and/or activation of non-canonical channel activity [12,13,14,15,16,17]. Like other TRP-channels and voltage-gated ion channels, TRPM3 channels share a multi-pass (type-2) transmembrane topology, typically assembled from tetramers of identical (homomeric) or similar (heteromeric) subunits around a central pore [18,19,20]. Each TRPM3 subunit possesses a cytoplasmic amino (N) terminus with four characteristic melastatin homology regions (MHR1-4) and several calmodulin binding sites, a transmembrane domain comprising six α-helical segments (S1-6) with the central cation pore lined by α-helices S5 and S6 connected by the hydrophobic pore-forming (P) loop, and a large cytoplasmic carboxy (C) terminal domain with a conserved TRP motif and a coiled-coil region [18,19,21,22]. Functional expression studies have shown that TRPM3 channels can be activated by several physico-chemical stimuli, including hypotonic extracellular challenge, the neuro-steroid pregnenolone sulfate (PS), a potent synthetic agonist (CIM0216), noxious heat, cell membrane phosphoinositol phosphates, and sex hormone steroids [15,23,24,25,26,27,28,29]. PS activation of TRPM3 channels elicits a strong increase in intracellular Ca^2+^ that activates a downstream signaling cascade involving mitogen-activated protein kinases (MAPKs), calmodulin, several nuclear phosphatases, the stimulus-response transcription factors (e.g., AP1), and epigenetic factors to regulate gene transcription [30,31,32,33,34,35]. Conversely, TRPM3 channels can be inhibited by several clinically approved drugs (e.g., non-steroidal anti-inflammatory drugs), antibiotics (e.g., voriconazole), volatile anesthetics (e.g., isoflurane), plant-derived secondary metabolites (e.g., citrus flavanones), and G-protein-coupled receptor βγ subunits [22,36,37,38,39,40,41]. TRPM3 channels are widely expressed in neurons and other cell types and have been implicated in a variety of cellular processes, including insulin/peptide secretion, vascular constriction and dilation, noxious heat sensing, inflammatory and spontaneous pain sensitivity, and tumorigenesis [24,25,28,42,43,44].

Genetic variations in the human TRPM3 gene (*TRPM3*) have been associated with a broad spectrum of inherited “channelopathies” and acquired diseases or conditions. Non-coding single-nucleotide variants (SNVs) in *TRPM3* have been associated with longevity, elevated low-density lipoproteins and triglycerides, systemic sclerosis, and thyroid nodules in humans [4,5] along with racing speed in dogs [45]. Rare deletions involving coding exons in *TRPM3* have been reported in Kabuki syndrome and autism [46,47], and a *TRPM3* variant has been implicated in an autosomal dominant form of centrofacial pruritis [48]. Recently, de novo missense mutations in *TRPM3* have been found to underlie a spectrum of neurological disorders, referred to as *TRPM3*-related neurodevelopmental disorder (*TRPM3*-NDD), that includes congenital hypotonia, developmental delay, intellectual disability, seizures, musculoskeletal, and ophthalmological findings [49,50,51]. Like *TRPM1*, *TRPM3* has also been associated with human eye diseases. A missense mutation near the 5′-end of *TRPM3* has been linked with autosomal dominant forms of cataract with or without glaucoma and anterior eye defects (e.g., persistent pupillary membrane) [52,53,54], and SNVs in *TRPM3* have been associated with age-related nuclear cataract, incipient senile cataract, age-related macular degeneration (AMD), and glaucoma [55,56,57,58]. Notably, the ophthalmological findings reported in *TRPM3*-NDD include strabismus, nystagmus, and refractive errors but not cataracts, AMD, or glaucoma [50]. Finally, mutation of the gene for micro-RNA 204 (*MIR204*), which is embedded within an intron of *TRPM3*, underlies autosomal dominant retinal dystrophy and iris coloboma with or without congenital cataract (RDICC) [59].

The vertebrate lens is a highly transparent cellular structure that plays a central role in eye development and—along with the cornea—serves to focus light onto the photosensitive retina [60,61]. Surrounded by a basement membrane or capsule, the ellipsoidal lens is composed of an anterior monolayer of lens epithelial cells that terminally differentiate into elongated lens fiber cells arranged in tightly packed, concentric growth shells to generate the refractive mass of the lens [62,63]. *Trpm3* has been identified as one of 65 “signature” genes for mouse lens epithelial cells [64] and, along with *Mir204,* is co-regulated by the paired-box transcription factor, PAX6, during eye development [65]. Mice lacking *Trpm3* have been found to display an attenuated pupillary response to dim light, whereas mice lacking *Mir204* acquire an AMD-like phenotype associated with pathogenic accumulation of rhodopsin and altered barrier function in the retinal pigment epithelium [66,67]. Previously, we have shown that *Trpm3*-null mice fail to develop early-onset cataract, whereas knock-in of a human cataract-associated mutation in *Trpm3* (p.I65M) elicits early-onset, progressive cataract with lens calcification and pro-fibrotic immune responses [68]. Here, we determine the ocular co-expression profile of *Trpm3* and *Mir204* and model the pathogenetic effects of the TRPM3 p.I65M substitution expressed in the mouse lens and human cell lines.

## 2. Materials and Methods

### 2.1. Mice and Lenses

C57BL6/J mice (B6J, strain #000664), B6(Cg)-*Tyr^c^*^−*2J*^/J mice (B6 albino, strain #000058), and B6J.Cg-Gt(ROSA)26Sor*^tm96^*^(*CAG-GCaMP6s*)*Hze*^/MwarJ mice (Ai96(RCL-GCaMP6s) or Ai96, strain #028866) [69] were obtained from The Jackson Laboratory (Bar Harbor, ME, USA). *Trpm3*-mutant mice were generated by CRISPR/Cas-9 gene editing as described by [68]. *Trpm3*-knockout (KO) mice were generated by homologous recombination as described by [70] and re-derived from frozen embryos obtained from the European Mutant Mouse Archive (EMMA) using standard techniques [68]. Transgenic *Cre*-recombinase (MLR10) mice were generously provided by Dr. M.L. Robinson (Ohio State University) [71]. *Mir204*-KO and *Mir204_Mir211* double KO (dKO) mice were generously provided by Dr. C. Zhang (National Eye Institute, National Institutes of Health, Bethesda, MD, USA) [67]. All experimental mouse strains were maintained on the B6J genetic background to avoid a deletion mutation in *Bfsp1* carried by certain inbred strains [72] and genotyped using the polymerase chain reaction (PCR) and gene-specific primers (Table S1) and as described by [68]. Mice were sacrificed in accordance with the American Veterinary Medical Association (AVMA) guidelines. Intact eyes or dissected lenses immersed in phosphate-buffered saline (PBS, #P4417-100TAB, MilliporeSigma, Burlington, MA, USA) were imaged with a digital camera (Spot Insight, Sterling Heights, MI, USA) fitted to a dissecting microscope (Stemi 2000, Zeiss, Thornwood, NY, USA). All mouse studies were approved by the Institutional Animal Care and Use Committee (IACUC) at Washington University School of Medicine (Protocol No. 22-0114).

### 2.2. Mutant Cell Lines

Human lens epithelial B3 (HLE-B3) cells (CRL-11421) were obtained from the American Type Culture Collection (ATCC, Manassas, VA, USA) and cultured in Eagle’s minimum essential medium (EMEM) supplemented with 20% fetal bovine serum (FBS, Gibco, Thermo Fisher Scientific, Waltham, MA, USA) under standard conditions (5% CO_2_, 37 °C). Mutant cell lines were produced using CRISPR/Cas9 (Clustered Regularly Interspersed Short Palindromic Repeats and CRISPR-associated protein 9) gene editing in our Genome Engineering and Stem Cell Center (geneediting.wustl.edu). Briefly, guide ribonucleic acids (gRNAs) and a single-stranded donor oligonucleotide (ssODN) were designed in silico, synthesized in vitro, and validated by sequencing to introduce a missense transition (c.195A > G) into exon-4 of *TRPM3* with ~30% non-homologous end joining (NHEJ) frequency. Sequences of the gRNA and ssODN were as follows: hTRPM3.sp13: 5′-ataaaatgctctttctatccngg-3′ and hTRPM3.ssODN: 5′-tacctatgggggtctttg gtgctgggtatgatgtggacacattctcttttataaaatgccctttccatccaggatttctgagcctgaaaaagaaaacaaaaaaaaaaaaaaaagaaaaaagaaagaa-3′. Of the five resulting cell lines, one was homozygous mutant, one was heterozygous mutant, and three were hemizygous mutants.

### 2.3. In Situ Hybridization

Lens RNA transcripts were localized in situ using custom-synthesized oligonucleotide probes to *Trpm3* (NM_001362504.1, target region 2400–3563 bp, Mm *Trpm3*-O5-C1, Cat. No. 1114341-C1) and the RNAscope 2.5 HD Detection Reagent—RED (Cat. No. 322360, Advanced Cell Diagnostics, ACD, Inc, Hayward, CA, USA) and to *Mir204* (NR_029591, SR-mmu-miR-204-5p-S1, Cat. no. 885551-S1) and the miRNAscope HD Detection Reagent—RED (Cat. No. 324510, ACD) according to the manufacturer’s instructions and as described by [73].

### 2.4. Immunofluorescence Confocal Microscopy

Whole eyes were processed using standard cryo-section techniques, and immunofluorescence microscopy was performed as described by [68,73,74,75,76,77]. Briefly, whole eyes were fixed in 4% paraformaldehyde and cryo-protected by serial incubation in 15% and 30% sucrose/PBS, then embedded in Tissue-Tek OCT compound (EMS, 62550-01) and saggital sections cut using a cryostat (Cryotome E, Thermo Fisher Scientific). Serial cryo-sections of eyes from at least three animals (*n* ≥ 6) were used to obtain reproducible images. TRPM3 was labeled with a polyclonal primary antibody (bs-9046R, Bioss Antibodies, Woburn, MA, USA) followed by Alexa-fluor conjugated secondary antibody (Thermo Fisher Scientific), and the actin cytoskeleton was labeled with phalloidin (ActinGreen 488, Cat no. R37110, Invitrogen, Thermo Fisher Scientific). Cell nuclei were counterstained with 4′,6-diaminido-2-phenylindole (DAPI, Millipore Sigma, Burlington, MA, USA) and sections imaged using a confocal microscope (FV1000, Olympus, Center Valley, PA, USA; Zeiss LSM800 with Airyscan, Carl Zeiss, White Plains, NY, USA).

### 2.5. Cation and Water Content

Lens cation content was measured using atomic absorption spectrometry (AAS) as described by [78]. Briefly, freshly dissected lenses were blotted dry and weighed, then lyophilized for 16 h (Freeze Dry System 7754000, LabConco Corporation, Kansas City, MO, USA) and re-weighed to determine the water content. Dried lenses were digested with 30% nitric acid (16 h, 20 °C), and dilutions of digest in ultra-pure water (>18.18 MΩ·cm) were quantified for Na^+^, K^+^, and Ca^2+^ using an atomic absorption spectrometer (Analyst 100, Perkin-Elmer, Norwalk, CT, USA) at wavelengths of 589.0 nm, 766.5 nm, or 422.7 nm, respectively. Data were presented as ion concentrations with units of millimoles per Kg of lens water and were not corrected for extracellular space since the lens extracellular space accounts for less than 2% of its volume [79].

### 2.6. Immunoblot Analysis

Immunoblot analysis of MAP kinase activation was performed with a polyclonal antibody to a synthetic peptide derived from the C-terminus of p44/42 MAPK (Erk1/2) (#9102, CST), Phospho-p44/42 MAPK (Erk1/2) (Thr202/Tyr204) (E10) Mouse mAb (#9106, CST), and IRdye-labelled secondary antibody and size-markers using the Odyssey Infrared (IR) imaging System (Li-Cor, Lincoln, NE, USA) according to the manufacturer’s protocol and as described by [68,76].

### 2.7. Expression Constructs

Wild-type (I65) and mutant (M65) open reading frames for human TRPM3 transcript variant-9 (NM_001007471.2) (Figure S1) were custom synthesized using de novo gene synthesis chemistry and ligated into the pReceiver-M61 (CMV-IRES-eGFP) mammalian expression vector (GeneCopia, Rockville, MD, USA).

### 2.8. Cell Culture and Transfection

Human embryonic kidney 293T (HEK293T) cells (CRL-1573, ATCC), maintained in 6-well tissue culture dishes in Dulbecco’s modified Eagle’s media (DMEM) supplemented with 10% FBS (Gibco, Thermo Fisher Scientific), were transfected with TRPM3-IRES-eGFP constructs using Lipofectamine 2000 (Invitrogen, Thermo Fisher Scientific) according to the manufacturer’s instructions. One day post-transfection, HEK-293 cells were seeded on Matrigel-coated coverslips (BD Biosciences, Bedford, MA, USA), and transfected cells were identified by membrane-targeted eGFP fluorescence prior to Ca^2+^ imaging analysis 24–48 h later.

### 2.9. Cytoplasmic Ca^2+^ Imaging

Fluorescent calcium measurement was performed using the Fura-2 ratiometric assay, as described in detail in [80]. Briefly, coverslips were loaded (45 min, 37 °C) with Fura-2 AM (2.5 µM) and 10% Pluronic F-68 (Molecular Probes, Eugene, OR, USA) in HBSS/HEPES buffer then washed in the same buffer and de-esterified in the dark (15 min, 37 °C) and used for Ca^2+^ imaging within 1 hr of loading. Fura-2-loaded cells were perfused in Tyrode’s solution (containing in mM: 130 NaCl, 2 KCl, 2 CaCl_2_, 2 MgCl_2_, 25 Hepes, 30 glucose, pH 7.3 to 7.4 with NaOH, and 310 mosmol/kgH_2_O) and alternately excited by 340 nm and 380 nm light with emission detected at 510 nm and the fluorescence ratio (R_340/380_) determined in >150 cells. After first establishing a baseline fluorescence ratio, the cells were exposed to (1 min) pregnenolone sulfate (PS, 10–50 µM, Sigma-Aldrich, St. Louis, MO, USA) or high (10 mM) external Ca^2+^ concentration. Cells were washed with Tyrode’s solution for 2–3 min between stimuli.

### 2.10. Mouse RNA-Sequencing Analysis

Mouse lens RNA samples (4 male and 4 female lenses per sample) were prepared in triplicate, and cDNA libraries were synthesized, indexed, pooled, and then sequenced using an Illumina NovaSeq 6000 in our Genome Technology Access Center (GTAC) according to the manufacturer’s protocols (Illumina, San Diego, CA, USA) and as described by [76]. Illumina’s bcl2fastq2 software version 2.20 was used to perform basecalls and demultiplexing. An Illumina DRAGEN Bio-IT on-premise server running version 3.9.3-8 software was used to align and quantify RNA-seq reads to the Ensembl release 101 primary assembly. All gene counts were then imported into the R/Bioconductor package EdgeR [81] for read depth normalization and pairwise comparison of each line to the appropriate control group. Only genes with counts-per-million (CPM) ≥ 5 within at least 3 samples were included in the analysis. Samples were validated for consistency across biological replicates by plotMDS function of the R package Limma [82]. To analyze for differences between conditions, we performed differential expression analysis, and the results were filtered for only those genes with Benjamini–Hochberg false-discovery rate-adjusted *p*-values less than or equal to (≤) 0.05 and with fold-change greater than or equal to (≥) 2. Normalized gene counts were then clustered using the R ComplexHeatmap package [83] before genes with similar differential expression patterns were analyzed for functional classification with the Gene Ontology knowledgebase [84,85].

### 2.11. Statistical Analysis

One-way analysis of variance (ANOVA) was used to determine statistical significance (*p*) ± standard error (SE) or standard deviation (SD).

## 3. Results

### 3.1. Ocular Expression Profiles of Trpm3 and Mir204

**Figure 1:**
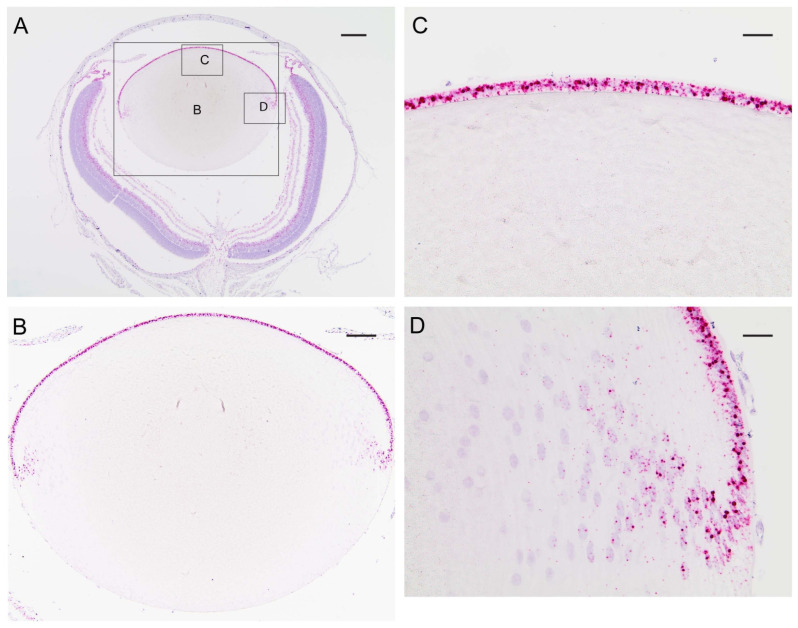
In situ hybridization of *Trpm3* transcripts in the neonatal mouse lens. Representative chromogenic labeling of *Trpm3* transcripts (red dots) in the albino mouse eye (**A**) and lens (**B**–**D**) at P5 showing strong localization to the anterior epithelium (**B**,**C**) and the equatorial epithelium and nascent lens fiber cells (**D**). Cell nuclei were counterstained with hematoxylin (blue). Scale bar: 200 µm (**A**), 100 µm (**B**), 50 µm (**C**,**D**).

**Figure 2:**
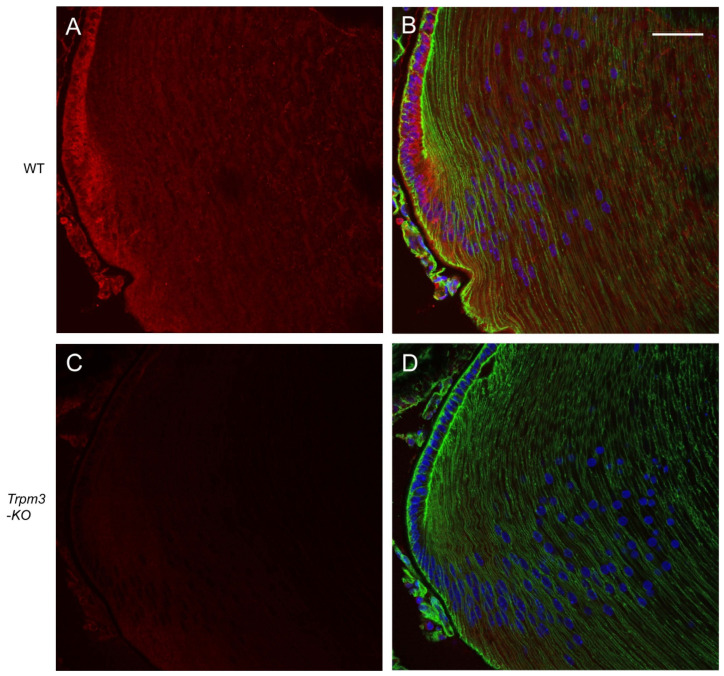
Immunofluorescence confocal microscopy of TRPM3 channels in the mature mouse lens. Representative immuno-fluorescent labeling of TRPM3 (red) in the wild-type lens (**A**,**B**) and *Trpm3*-KO lens (**C**,**D**) at P21 showing localization to the lens equatorial epithelium in (**A**,**B**) but not (**C**,**D**). Actin cytoskeleton and cell nuclei were labeled with phalloidin (green) and DAPI (blue), respectively. Scale bar: 50 µm.

In order to determine the distribution of transcripts for *Trpm3* in the wild-type mouse eye, we performed in situ hybridization (ISH) with an RNAscope probe that spanned the coding region for the transmembrane channel domain of TRPM3. ISH revealed that *Trpm3* transcripts were strongly localized to the postnatal day 5 (P5) lens epithelium and nascent lens fiber cells (Figure 1). *Trpm3* transcripts were also localized to other ocular tissues at P5, including the non-pigmented and pigmented iris and ciliary epithelia, the corneal epithelium, the retinal pigmented epithelium, the inner neuro-retina, and the optic nerve (Figure S2). We note that *Trpm3* transcripts were also localized to the mature (P21) *Trpm3*-KO lens epithelium (Figure S3), suggesting that the ISH probe cross-hybridizes with *Trpm3* transcripts interrupted by the IRES-*LacZ-neo* cassette (Figure S1), which enables expression of the β-galactosidase reporter gene [68]. Since *Trpm3*-cassette “fusion” transcripts were present in *Trpm3*-KO lenses, we sought to compare the localization of TRPM3 channels in wild-type versus *Trpm3*-KO lenses using a polyclonal antibody that recognizes a peptide derived from the C-terminal region of human TRPM3. Immunofluorescence confocal microscopy confirmed strong localization of TRPM3 to the wild-type lens epithelium that was absent from the *Trpm3*-KO lens epithelium (Figure 2). 

To compare the ocular distribution of *Trpm3* with that of its intron-hosted micro-RNA gene, *Mir204* (on mouse chromosome 19), we performed ISH with an RNAscope probe spanning the 5p arm of *Mir204*. Since *Mir204* shares strong homology with *Mir211*, which is hosted in an intron of *Trpm1* (on mouse chromosome 7), the ISH probe may not discriminate between these miRNAs. Like *Trpm3* transcripts, *Mir204/211* transcripts were strongly localized to the neonatal (P5) lens epithelium, non-pigmented and pigmented epithelia of the iris and ciliary body, and the retinal pigmented epithelium (Figures S4 and S5). Overall, our data suggest that co-incident expression of *Trpm3* and *Mir204/211* in the eye was strongest in epithelia from the lens, iris, ciliary body, and retina.

### 3.2. Lens Phenotype of Mir204- and Mir211-Deficient Mice

**Figure 3:**
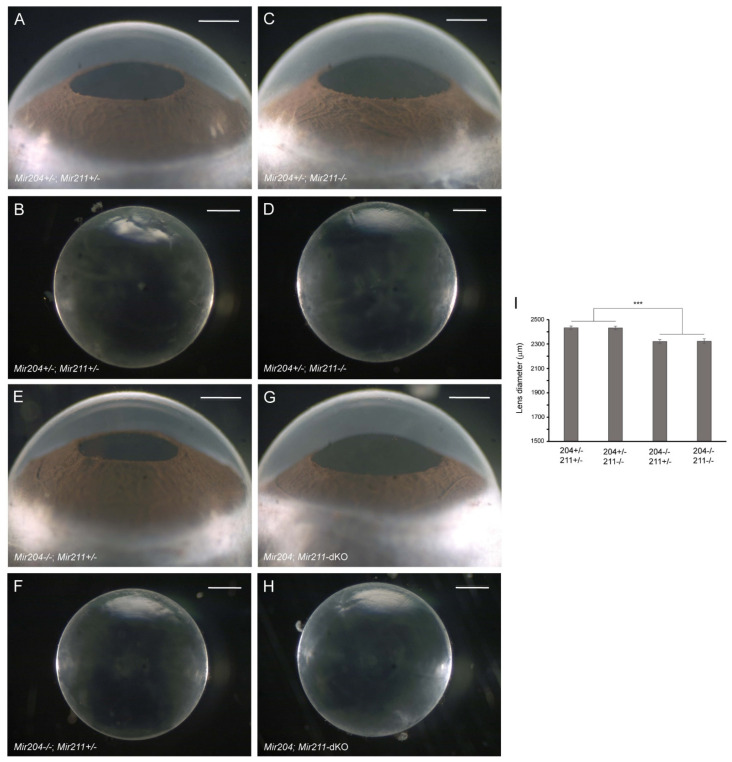
Lens phenotype of *Mir204-* and *Mir211*-deficient mice. Representative dark-field images of eyes (**A**,**C**,**E**,**G**) and corresponding lenses (**B**,**D**,**F**,**H**) from *Mir204*^+/−^; *Mir211*^+/−^ mice (**A**,**B**), *Mir204*^+/−^; *Mir211^−/−^* mice (**C**,**D**), *Mir204*^−/−^; *Mir211*^+/−^ mice (**E**,**F**), and *Mir204*; *Mir211*-dKO mice (**G**,**H**) at 4 months of age. Scale bar: 500 µm (**A**–**H**). (**I**) Lens diameter for each genotype ± SD. *** *p* < 0.001.

Since *Mir204* is hosted within an intron of *Trpm3* and a mutation in *MIR204* has been associated with RDICC in humans [59], we next sought to determine the lens phenotype of *Mir204*-KO mice. At 4–6 months of age, *Mir204*-KO mice displayed a grossly normal eye phenotype with clear lenses similar to wild-type mice (data not shown). Thus, *Mir204*-KO mice—like *Trpm3*-KO mice—maintained clear lenses well beyond early-onset cataract in *Trpm3*-mutant (I/M and M/M) mice [68]. Since *Mir204* shares homology with *Mir211*, we sought to address the possibility of functional redundancy between the two micro-RNAs by examining the lens phenotype of *Mir204*; *Mir211*-dKO mice (*Mir204*^−/−^; *Mir211*^−/−^). Littermates hemizygous for *Mir204* and either hemizygous or null for *Mir211* (*Mir204*^+/−^; *Mir211*^+/−^ or *Mir204*^+/−^; *Mir211*^−/−^) developed a grossly normal eye phenotype with clear lenses at 4 months of age (Figure 3A–D). Similarly, *Mir204*; *Mir211*-dKO and *Mir204*^−/−^; *Mir211*^+/−^ littermates displayed a grossly normal eye phenotype with clear lenses at 4 months of age (Figure 3E–H). However, *Mir204*; *Mir211*-dKO and *Mir204*^−/−^; *Mir211*^+/−^ lenses were smaller than those from *Mir204*^+/−^; *Mir211*^+/−^ and *Mir204*^+/−^; *Mir211*^−/−^ littermates (Figure 3I). Thus, the loss of both *Mir204* copies and at least one *Mir211* copy resulted in a lens growth deficit in the absence of early-onset cataract. We note that hyper-mature cataracts have been reported in *Mir204*; *Mir211*-dKO mice at 10–15 months of age [86]. Regardless, such a late onset contrasts with the early cataract development in *Trpm3*-mutant (I/M, M/M) mice. Overall, our data suggest that, like loss of *Trpm3*, loss of *Mir204* and/or *Mir211* did not cause early-onset cataract similar to that found in *Trpm3*-mutant mice [68].

### 3.3. Cation and Water Content of Trpm3-Mutant Lenses

**Figure 4:**
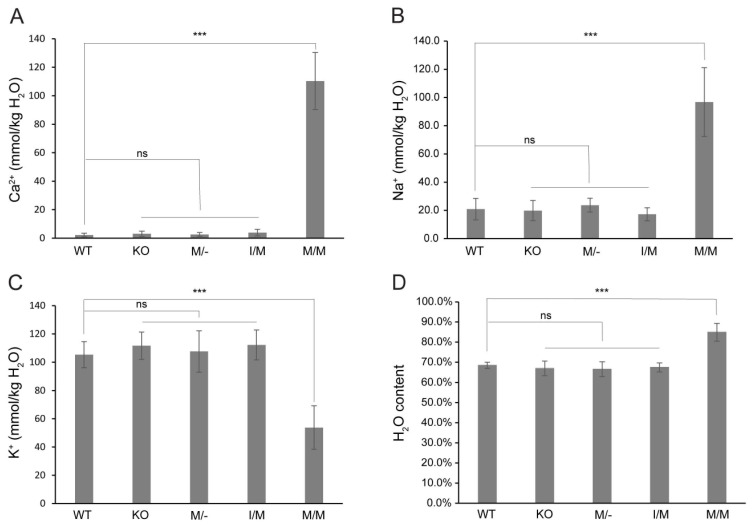
Cation and water content of *Trpm3*-mutant and *Trpm3*-KO lenses. Atomic absorption spectroscopy measurements of Ca^2+^ (**A**), Na^+^ (**B**), and K^+^ (**C**) concentrations and water content (**D**) in lenses (P21) from wild-type (WT), *Trpm3*-KO, hemizygous (M/−), heterozygous (I/M), and homozygous (M/M) *Trpm3*-mutant mice. *** *p* < 0.001; ns—not significant; error bars ± SD.

**Figure 5:**
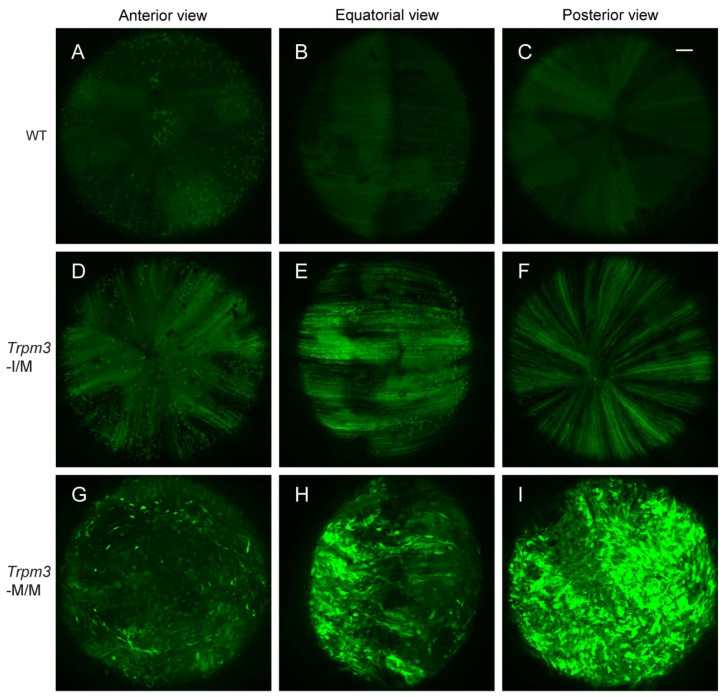
Elevated calcium in *Trpm3*-mutant lenses determined using a genetically encoded calcium sensor. Representative confocal fluorescence microscopy images of *Cre*-dependent GCaMP6s activation in wild-type (**A**–**C**), heterozygous *Trpm3*-I/M (**D**–**F**), and homozygous *Trpm3*-M/M (**G**–**I**) lenses at P21 showing anterior (**A**,**D**,**G**), equatorial (**B**,**E**,**H**), and posterior (**C**,**F**,**I**) views. Scale bar: 100 µm.

Previously, we provided histochemical evidence for Ca^2+^ accumulation in *Trpm3*-mutant lenses [68]. In order to further quantify the cation content of *Trpm3*-mutant and *Trpm3*-KO lenses, we undertook atomic absorption spectrometry (AAS). AAS revealed that *Trpm3*-KO, hemizygous *Trpm3*-mutant (M/−), and heterozygous *Trpm3*-mutant (I/M) lenses had intracellular cation (Ca^2+^, Na^+^, K^+^) levels and water content similar to those of wild-type lenses (Figure 4). By contrast, homozygous *Trpm3*-mutant (M/M) lenses had dramatically elevated intracellular Ca^2+^ concentrations ([Ca^2+^]_i_) coupled with increased ([Na^+^]_i_), reduced ([K^+^]_i_), and an increased water content (85–90%) compared to wild-type lenses (66–70%) (Figure 4)—consistent with a failure of *Trpm3*-M/M lenses to osmoregulate. To further visualize Ca^2+^ levels in intact *Trpm3*-mutant lenses ex vivo, we crossed *Trpm3*-mutant mice with mice transgenic for the genetically encoded, *Cre*-dependent fluorescent Ca^2+^ indicator variant (GCaMP6s) that is characterized by high sensitivity with slow decay and response kinetics [87]. Subsequent breeding of GCaMP6s;*Trpm3*-mutant mice with lens-specific *Cre* mice [71] enabled deletion of a floxed-STOP cassette preventing GCaMP6s expression and resulted in excitation of enhanced green fluorescent protein (EGFP) proportional to the binding of cytosolic Ca^2+^ to the calmodulin and calmodulin-interacting M13 peptide (CaMP) complex. Figure 5 revealed mildly increased fluorescence in *Trpm3*-I/M lenses and intense fluorescence in the *Trpm3*-M/M lens compared to the wild-type lens—supporting Ca^2+^ influx. Overall, these cation data were consistent with Ca^2+^ accumulation and failed osmoregulation, contributing to cataract development in *Trpm3*-mutant lenses.

### 3.4. Functional Expression of Mutant TRPM3 Channels

**Figure 6:**
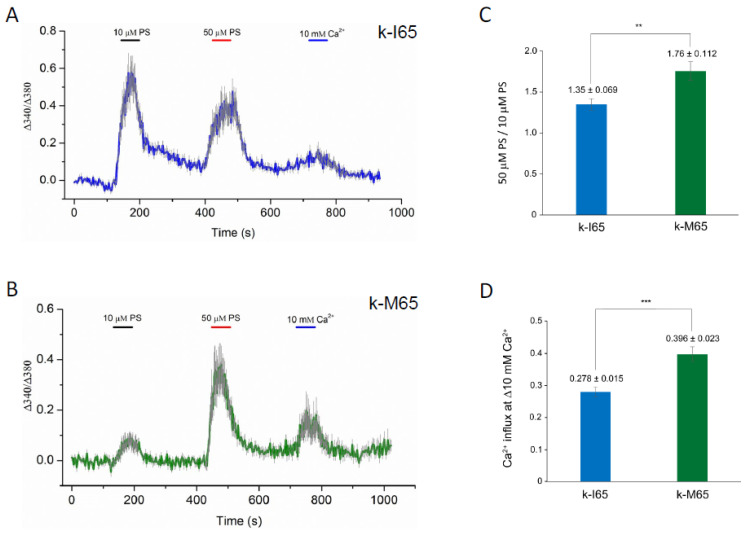
Cytoplasmic calcium responses in Fura-2-loaded HEK-293T cells that express either wild-type (I65) or mutant (M65) TRPM3 (k-isoform) channels. (**A**,**B**) Representative traces of Ca^2+^ transients evoked by 10 μM and 50 μM pregnenolone sulfate (PS) or by changes of extracellular Ca^2+^ (2 mM to 10 mM). Traces are averaged from cells in one region of interest expressing wild-type k-I65 (**A**) and mutant k-M65 (**B**) TRPM3 channels, respectively. (**C**) Ratio of peak Ca^2+^ influx in response to 50 μM PS versus 10 μM PS. (**D**) Peak Ca^2+^ influx in response to increase in extracellular Ca^2+^ concentration. ** *p* < 0.01; *** *p* < 0.001; error bars ± SD.

TRPM3 was originally shown to function as a spontaneous Ca^2+^ channel that can be activated by PS in vitro [23,24,88]. In order to determine the pathogenic effects of the cataract-causing p.I65M substitution on TRPM3 channel properties, we undertook transient (over-)expression of wild-type (I65) and mutant (M65) channels in HEK-293T cells followed by fluorescent Ca^2+^ imaging with Fura-2. According to the current human reference sequence (GRCh39p.14), *TRPM3* comprises 30 exons that generate at least 23 transcript variants (1–23) and channel isoforms (a–w) via alternative exon splicing [54] (https://www.ncbi.nlm.nih.gov/gene/80036; accessed on 5 December 2023). Of the 23 predicted channel isoforms (a–w), 19 are full-length with a transmembrane channel pore domain (a, b, d–g, i–k, m–t, v, w), and 4 are truncated lacking a channel pore (h, c, l, u). Since most of the full-length channel isoforms start translation in exon-2, skip exon-3 (non-coding), and include exon-4 containing the p.I65M substitution (codons 60–86), we selected variant-9 (NM_001007471) for custom synthesis of wild-type and mutant expression plasmids (Figure S1). Variant-9 is expressed in the human lens [52] and encodes channel isoform-k (NP_001007472), comprising 1707 amino acid residues. Following transient expression of wild-type (k-I65) and mutant (k-M65) channels, the Fura-2 ratio-metric assay revealed robust and long-lasting Ca^2+^ influx in response to 50 µM PS activation (Figure 6A,B). However, compared to wild-type k-I65 channels, the mutant k-M65 channels exhibited a higher ratio of peak Ca^2+^ influx in response to 50 μM PS versus 10 µM PS, suggesting a steeper dose–response relationship at this concentration range (Figure 6A,C). In addition, the mutant k-M65 channels elicited an increased Ca^2+^ influx response to elevated extracellular Ca^2+^ (from 2 mM to 10 mM) compared to wild-type k-I65 channels (Figure 6B,D). Overall, while the significance of the altered sensitivity of mutant k-M65 channels to PS activation in the lens is unclear, the increased sensitivity of the mutant k-M65 channels to extracellular Ca^2+^ concentration may contribute to cataract formation.

### 3.5. MAP Kinase Activation in TRPM3-Mutant Cell Lines and Lenses

**Figure 7:**
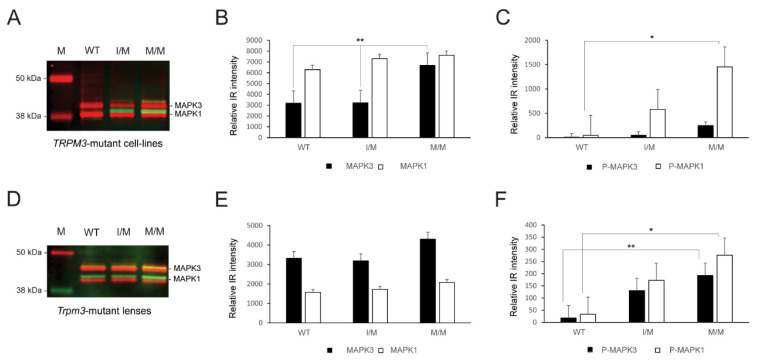
MAP kinase activation in the *TRPM3*-mutant human lens epithelial B3 (HLE-B3) cells and *Trpm3*-mutant mouse lenses. Quantitative immunoblot analysis of MAPK3/p44 and MAPK1/p42 in heterozygous *TRPM3*-I/M and homozygous *TRPM3*-M/M mutant HLE-B3 cell-lines (**A**–**C**) and in *Trpm3*-mutant mouse lenses at P21 (**D**–**F**). Constitutive MAPK3 and MAPK1 levels are shown in (**A**,**D**) (red bands) and in (**B**,**E**) (black and white bars). Phosphorylated (P) MAPK3 and MAPK1 levels are shown in (**A**,**D**) (green/yellow bands) and in (**C**,**F**) (black and white bars). WT, wild-type. M, molecular weight markers. * *p* < 0.05; ** *p* < 0.01; error bars ± SE.

Since TRPM3-facilitated Ca^2+^ influx has been associated with the activation of mitogen-activated protein kinase-1 MAPK1/ERK2/p42 and MAPK3/ERK1/p44 in vitro [32,33], we performed immunoblot analysis to compare MAP kinase status in knock-in *TRPM3*-mutant HLE-B3 cell lines and *Trpm3*-mutant lenses versus wild-type lenses. Immunoblot analysis revealed that constitutive MAPK1 levels were significantly greater than MAPK3 levels in wild-type and heterozygous *TRPM3*-mutant (I/M) HLE-B3 cells, whereas constitutive MAPK3 levels were significantly elevated and similar to MAPK1 levels in homozygous *TRPM3*-mutant (M/M) HLE-B3 cells (Figure 7A,B). Moreover, phospho-activation of MAPK1 was significantly greater in *TRPM3*-M/M HLE-B3 cells compared to wild-type cells (Figure 7A,C). By contrast, constitutive MAPK3 levels were greater than MAPK1 levels in wild-type, *Trpm3*-I/M, and *Trpm3*-M/M lenses (Figure 7D,E), and phosphorylated MAPK1 and MAPK3 levels were both significantly elevated in *Trpm3*-M/M lenses compared to wild-type lenses (Figure 6D,F). Overall, while the relative basal levels of MAPK1 and MAPK3 differed between HLE-B3 cells and mouse lenses, phospho-activation of MAPK3 and/or MAPK1 was increased in the presence of mutant TRPM3 channels.

### 3.6. Gene Expression Profiles of Trpm3-Mutant and Trpm3-KO Lenses

**Figure 8:**
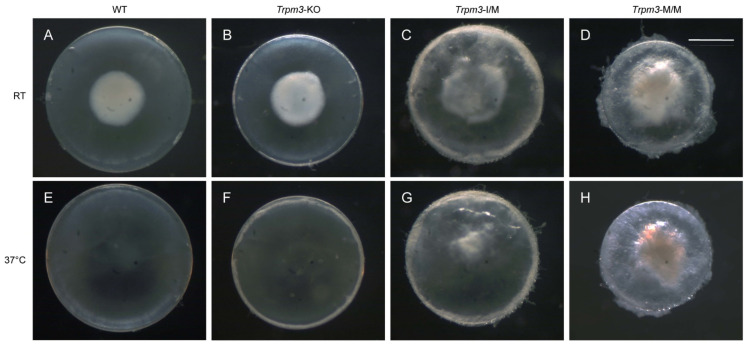
Lens phenotype of neonatal *Trpm3*-mutant and *Trpm3*-KO mice. Representative dark-field images of wild-type (**A**,**E**), *Trpm3*-KO (**B**,**F**), *Trpm3*-I/M (**C**,**G**), and *Trpm3*-M/M (**D**,**H**) lenses at P7 showing cold-cataract formation (**A**–**D**) during dissection at room temperature (RT) that was reversible at 37 °C (**E**–**H**). Note the *Trpm3*-mutant cataract was not temperature sensitive (**C**,**D**,**G**,**H**). Scale bar: 500 µm.

To determine the effects of the *Trpm3* mutation versus *Trpm3* deficiency on lens gene expression, we performed RNA-seq analysis to compare global transcriptional changes in *Trpm3*-mutant (I/M, M/M) lenses during early cataract development versus non-cataractous *Trpm3*-KO and wild-type lenses at P7. We selected lenses at P7 since resorption of the embryonic tunica vasculosa lentis (TVL) was largely complete, and at ages beyond P7, *Trpm3*-M/M lenses became severely opacified, fragile, and prone to rupture since they could not be cleanly dissected away from adherent contamination of other ocular tissues including the iris anteriorly and the retina posteriorly. During the dissection of P7 eyes at room temperature (20–22 °C), we observed so-called “cold cataract” formation in the core regions of wild-type, *Trpm3*-KO, and *Trpm3*-mutant lenses (Figure 8A–D). A cold cataract is a natural phenomenon of neonatal rodent lenses believed to result from changes in the supramolecular organization of specific lens proteins, including the beaded filament-specific proteins BFSP1 and BFSP2 [89], which can be effectively reversed by warming to 37 °C (Figure 8E–H). Despite the role of TRPM3 in heat detection by sensory neurons [28,70], neither *Trpm3* deficiency nor *Trpm3* mutation appeared to impact the reversal of cold cataracts in the lens.

**Figure 9:**
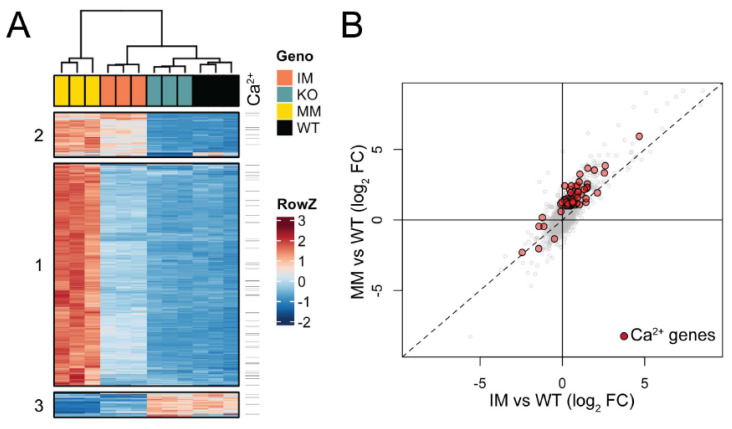
Gene expression changes in *Trpm3*-mutant and *Trpm3*-KO lenses using RNA-seq analysis. (**A**) Heatmap showing unique expression changes (≥2-fold) in *Trpm3*-mutant (I/M, M/M) and *Trpm3*-KO lenses compared to wild-type lenses at P7. (**B**) Scatterplot highlighting genes involved in Ca^2+^-associated processes (red) that are differentially expressed in *Trpm3*-I/M and *Trpm3*-M/M lenses.

Triplicate lens RNA samples were sequenced to a depth of >60 M reads and aligned to the mouse mm10 genome build with a >98% alignment rate. Multi-dimensional scaling (MDS) analysis of RNA-seq data showed high consistency between biological triplicates at P7, with data from each genotype clustering independent from other samples (Figure S6A). Pairwise differential expression analysis comparing each *Trpm3* genotype to wild-type identified 1249 genes with significant expression changes (fold-change, FC ≥ 2, false discovery rate, FDR ≤ 0.05). Across *Trpm3* genotypes, 1131 genes were upregulated, with the majority identified in *Trpm3*-M/M versus wild-type comparison, while the comparison of *Trpm3*-I/M and *Trpm3*-KO to wild type identified few uniquely affected genes (Figure S6B). The 129 downregulated genes were less consistent between genotypes (Figure S6C). To better understand the relative effects of these gene sets in the three mouse models compared to the wild type, we clustered the 1249 mis-expressed genes by their relative expression across all samples (Figure 9A). Generally, the data supported the intermediate phenotype of *Trpm3*-I/M lenses compared to *Trpm3*-M/M lenses, as well as the lack of a significant transcriptional phenotype in *Trpm3*-KO lenses. Hierarchical clustering identified three primary gene sets (Figure 9A). The largest, Cluster 1, comprised 951 genes that were mostly upregulated in *Trpm3*-M/M lenses, with only moderate effects noted in *Trpm3*-I/M (Table S2). Clusters 2 (191 genes) and 3 (107 genes) were significantly smaller and comprised up- and downregulated gene sets, respectively, where *Trpm3*-I/M and *Trpm3*-M/M lenses were more similar to each other than to WT and *Trpm3*-KO lenses (Table S2). To identify genes that could be mediating the more severe phenotype in *Trpm3*-M/M versus *Trpm3*-I/M lenses, we first focused on the genes within Cluster 1. Gene ontogeny (GO) analysis of genes in Cluster 1 indicated enrichment of at least 16 GO categories comprising 680 genes related to Ca^2+^ homeostasis and transport (Table S3). To better comprehend the calcium-related transcriptional response in *Trpm3*-mutant lenses, we identified all genes significantly affected in our dataset that are within those 16 Ca^2+^-associated GO categories. Of the 80 genes identified, the majority lie within Cluster 1 and show a more severe upregulation in *Trpm3*-M/M than in *Trpm3*-I/M lenses (Figure 9, Table S4). Four of the most upregulated genes (>5-fold) in *Trpm3*-M/M lenses included those coding for the plasma-membrane Ca^2+^ transporting ATPases *Atp2b1* and *Atp2b4*, the Na^+^/K^+^ transporting ATPase beta-1 subunit, *Atp1b1*, and the Ca^2+^ binding protein, *Cabp5*. In addition, the genes for two other TRP channels, *Trpv4* and *Trpm1*, were upregulated (~ 2.4-fold) in *Trpm3*-M/M lenses but not in *Trpm3*-KO lenses, suggesting these channels do not compensate for the loss of *Trpm3* function in the lens (Table S4). Further, since the relative expression level of *Trpm3* in mutant lenses is over 25-fold greater than those of *Trpm1* and *Trpv4* (Figure S7), it is unlikely that mild upregulation of the latter channels contributes greatly to the lens phenotype of *Trpm3*-mutant mice.

GO analysis of genes within Cluster 2 suggested that genes related to membrane potential and development are upregulated in both *Trpm3*-I/M and *Trpm3*-M/M lenses at P7 (Table S3). GO analysis of the downregulated genes in Cluster 3 showed enrichment for genes involved in the regulation of insulin and peptide secretion (Table S3)—a known function of TRPM3 [24,25,44]. Overall, the relative upregulation of gene expression in *Trpm3*-mutant lenses revealed a progressive transcriptional phenotype in which *Trpm3*-I/M lenses were moderately affected and *Trpm3*-M/M lenses were severely affected with significant activation of genes underlying Ca^2+^ homeostasis/dynamics (Figure 9B). 

## 4. Discussion

*Trpm3* has been identified as a signature gene for the lens epithelium and, along with its intron-hosted miRNA gene, *Mir204*, has been implicated in eye development and disease [52,53,54,55,56,57,58,59,64,65,68]. In this study, we have shown that *Trpm3* and *Mir204/211* were strongly co-expressed in the lens epithelium and other non-pigmented and pigmented ocular epithelia from the iris, ciliary body, and retina. Further, we found that a cataract-causing substitution (p.I65M) in *Trpm3* resulted in (1) altered sensitivity of mutant TRPM3-M65 channels to extracellular [Ca^2+^] and PS activation in vitro and (2) disturbed cation (Ca^2+^, Na^+^, K^+^) and water homeostasis, increased MAPK1/3 phospho-activation, and upregulation of genes involved in Ca^2+^-dependent processes in the lens. By contrast, *Trpm3*-KO lenses did not replicate the pathophysiological changes found in *Trpm3*-mutant (I/M, M/M) lenses and, like *Trpm3*-KO lenses, neither *Mir204*-KO nor *Mir204*; *Mir211*-dKO lenses developed early-onset cataract. While the combined loss-of-function for *Trpm3* and *Mir204/211* on lens phenotype remains to be determined, our data suggest that early-onset cataract in *Trpm3*-mutant lenses resulted from a deleterious gain-of-function mechanism rather than a loss-of-function mechanism in the lens. Further, since the signature function of TRPM3 channels in the lens remains unclear, future studies will be needed to examine whether and how other mechanisms might compensate for the absence of the channel.

Elevated cytoplasmic Ca^2+^ concentrations, believed to result from an increase in non-selective cation conductance, have long been implicated in the pathophysiology of lens aging and cataract formation in humans and experimental animals [90]. Atomic absorption spectroscopy and fluorescent calcium imaging (GCaMP6s) studies of ex vivo *Trpm3*-mutant mouse lenses revealed that while cytosolic Ca^2+^ levels in heterozygous (I/M) and hemizygous (M/−) *Trpm3*-mutant lenses were mildly elevated, those in homozygous (M/M) *Trpm3*-mutant lenses were dramatically higher (~50-fold) than wild-type lenses (Figure 4A and Figure 5)—consistent with previously reported histochemical calcification of *Trpm3*-M/M lenses [68]. The high ([Ca^2+^]_i_) in *Trpm3*-M/M lenses was coupled with elevated ([Na+]_i_), decreased ([K+]_i_), and increased water content compared to wild-type lenses, further suggesting that osmoregulation had failed in *Trpm3*-M/M lenses (Figure 4B–D). In addition, functional over-expression studies in transfected HLE-B3 cells revealed that recombinant mutant TRPM3-M65 channels displayed a steeper dose–response to PS activation (between 10 and 50 μM) and an increased sensitivity to extracellular [Ca^2+^] (10 mM) when compared with wild-type TRPM3-I65 channels in vitro. Further, constitutive expression of knock-in mutant TRPM3-M65 channels in HLE-B3 cells and *Trpm3*-M/M lenses significantly increased phospho-activation of MAPK1/3 (Figure 7)—a known downstream target of TRPM3-mediated Ca^2+^ influx [32,33]. While PS is an effective experimental agonist for TRPM3 channels, the role of this neuro-steroid in lens biology is unclear, as is the significance of an altered sensitivity to PS activation. The true physiological agonist(s) of TRPM3 in the eye/lens remains elusive. Regardless, our data suggest that mutant TRPM3-M65 channels ‘leak’ inwardly when exposed to elevated extracellular [Ca^2+^], leading to cytoplasmic Ca^2+^ overload coupled with MAPK1/3 activation. Since the extracellular Ca^2+^ levels in the eye’s aqueous humor (1–2 mM), which bathes the anterior lens epithelium, are ~10,000-fold higher than intracellular Ca^2+^ levels in the lens (100 nM range) [90], it is likely that this steep inward concentration gradient causes increased Ca^2+^ entry via mutant TRPM3-M65 channels resulting in chronic Ca^2+^ accumulation in the developing lens triggering early-onset, progressive cataract. Beyond a role for *TRPM3* in rare forms of inherited pediatric cataract, it is conceivable that aging human TRPM3 channels may also contribute to the historically enigmatic, non-selective cation leak implicated in the much more common forms of age-related cataract.

Recently, functional expression studies of de novo missense mutations underlying *TRPM3*-NDD, which include ophthalmological findings other than cataract, have been shown to increase basal channel activity leading to cellular calcium overload and enhanced response to PS activation—consistent with a deleterious gain-of-function effect(s) [51]. These *TRPM3*-NDD studies focused on transcript variant-14 (NM_001366145.2) coding for channel isoform-m (NP_ 1353074.2, 1719 amino-acids) that starts in exon-2 but contains one more coding exon (exon-16, 12 amino acids) than the variant-9/isoform-k (1707 amino-acids) used here (Figure S1). In addition to the different variants/isoforms studied, the *TRPM3*-NDD missense mutations lie in or near the transmembrane (TM) pore domain toward the C-terminus of channel isoform-m, whereas the p.I65M mutation underlying *TRPM3* cataract lies distant to the pore near the N-terminus of channel isoform-k within a predicted calmodulin binding motif [21]. Further, since we have shown that the p.I65M missense mutation may also act as an alternative translation start site in vitro [52], we cannot exclude the possibility that it may generate N-terminal mutant isoforms of TRPM3 (starting at codon 65 of isoform-k) with novel channel properties in vivo. These TRPM3 mutants may include N-terminal truncation mutants of isoforms starting translation upstream in exon-2 (p.M1del-64 mutants) and N-terminal extension mutants of isoforms starting translation downstream at the end of exon-5 (p.M1ext-89 mutants) (Figure S1). Such differences in the location of mutations within functionally distinct regions of TRPM3 coupled with alternative splice and/or start sites between TRPM3 variants and isoforms may contribute to pleiotropic effects that generate variable phenotypes in the brain (*TRPM3*-NDD) versus the lens (*TRPM3* cataract).

*Trpm3*-mutant lenses undergoing cataract development at P7 revealed >1240 differentially regulated genes, whereas transparent *Trpm3*-KO lenses at P7 yielded ~50 differentially regulated genes with minimal sharing between mutant and null genotypes (Figure S6B). These data highlight the dramatically distinct effects of *Trpm3* mutation versus *Trpm3* deficiency on global gene expression in the lens. *Trpm3*-mutant lenses revealed modest downregulation of genes involved in insulin and peptide secretion (Figure 9A, Tables S2 and S3). Activation of TRPM3 channels with PS or the synthetic agonist CIM0216 has been shown to control insulin release from pancreatic β cells and calcitonin gene-related peptide (CGRP) release from sensory nerve terminals [24,25,44]. However, the precise role of TRPM3-mediated insulin and/or neuro-peptide release in the lens is unclear. Conversely, *Trpm3*-mutant lenses revealed strong upregulation of genes involved in cation homeostasis—including those for the P-type ion-transport ATPases *Atp1b1*, *Atp2b1*, and *Atp2b4* (Table S4). *Atp1b1* encodes the glycosylated Na^+^/K^+^-transporting ATPase subunit β-1 (ATP1B1) that complexes with the large catalytic α-subunit to regulate the formation of functional Na^+^/K^+^ pumps, which actively extrude Na^+^ and uptake K^+^ in order to establish and maintain electrochemical gradients across the plasma membrane [91]. *Atp2b1* and *Atp2b4* encode the calmodulin-dependent plasma membrane Ca^2+^-ATPases ATP2B1 and ATP2B4, respectively, that function in Ca^2+^ export from eukaryotic cells against steep concentration gradients and play a critical role in intracellular Ca^2+^ homeostasis [92]. Such upregulation of ion-transport ATPase genes is consistent with attempts to counteract increased Ca^2+^ influx caused by functionally mutant TRPM3 channels expressed in the anterior epithelium of the lens.

## 5. Conclusions

Overall, our data support a deleterious gain-of-function mechanism that activates TRPM3 (p.I65M) mutant channels to drive chronic Ca^2+^-influx in the lens, resulting in an inherited form of early-onset, progressive cataract. Further, they suggest that TRPM3 channels may participate in the non-selective cation leak long associated with lens aging and cataract formation. The fact that TRPM3 channels can be inhibited by certain drugs (e.g., NSAIDs) and nutraceuticals—including citrus flavanones that have been associated with the attenuation of cataract [93,94]—raises the possibility of therapeutic interventions for *TRPM3* cataract.

## Data Availability

RNA-seq data files have been deposited in the Gene Expression Omnibus (GEO) database under accession no. GSE250518.

## Supplementary Materials

The following supporting information can be downloaded at: https://www.mdpi.com/article/10.3390/cells13030257/s1, Figure S1: Schematic of exon usage for TRPM3 transcript variant-9 used herein for functional expression studies of channel isoform-k in Figure 6; Figure S2: In situ hybridization (ISH) of Trpm3-transcripts in the neonatal mouse eye; Figure S3: In situ hybridization of *Trpm3*-transcripts in the mature mouse lens; Figure S4: In situ hybridization of Mir204 transcripts in the neonatal mouse lens; Figure S5: In situ hybridization of Mir204-transcripts in the neonatal mouse eye; Figure S6: Multi-dimensional scaling (MDS) plot (A) and Venn diagrams of upregulated genes (B) and downregulated genes (C) in heterozygous *Trpm3*-I/M, homozygous *Trpm3*-M/M, and *Trpm3*-KO lenses versus wild-type lenses at P7; Figure S7: Scatterplot of differentially regulated genes in *Trpm3*-KO (A), *Trpm3*-I/M (B), and *Trpm3*-M/M (C) lenses highlighting the relative expression levels of *Trpm3* versus *Trpm1* and *Trpv4*; Table S1: Primer sequences used for PCR-genotyping of *Mir204*-KO and *Mir204*; *Mir211*-dKO mice; Table S2: Differentially expressed genes (>2-fold) in *Trpm3* -mutant lenses summarized in Figure 9A.; Table S3: Enriched GO categories for genes within each cluster in Figure 9A; Table S4: Genes associated with Ca^2+^ dynamics within Cluster 1 in Figure 9A that were most upregulated in *Trpm3*-M/M lenses.
