# Supplementary material for: A Cataract-Causing Mutation in the TRPM3 Cation Channel Disrupts Calcium Dynamics in the Lens
Source: Cells. 2024 Jan 30;13(3):257. doi: 10.3390/cells13030257 (PMC10854584; doi:10.3390/cells13030257)
Supplement: Supplementary file 1 [file cells-13-00257-s001.zip › cells-2834700-supplementary.pdf]

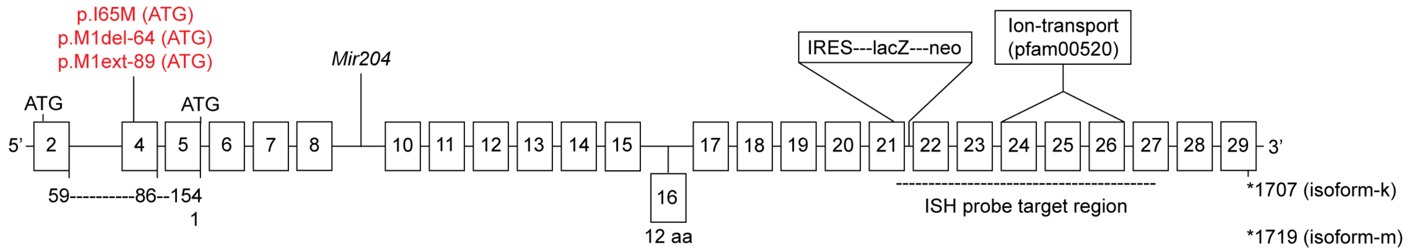

Figure S1. Schematic of exon usage for *TRPM3* transcript variant-9 used herein for functional expression studies of channel isoform-k in Figure 6. Variant-9 (NM_001007471) skips exon-3, exon-9, and exon-16, and translation of isoform-k (NP_ 001007472, 1707 amino acids) initiates from a constitutive start-site located in exon-2. Another constitutive translation start-site for other TRPM3 isoforms is located at the end of exon-5. The p.I65M substitution located in exon-4 may also serve as an alternative translation start-site generating N-terminal truncated mutant channels (p.M1del-64) or N-terminal extension mutant channels (p.M1ext-89). The approximate target region of the RNAscope probe used for *in situ* hybridization (ISH) is indicated. For comparison, variant-14 (NM_001366145) also skips exon-3 and exon-9 but retains exon-16 (12 amino acids) to encode isoform-m (NP_1353074, 1719 amino acids) used for functional studies of *TRPM3*-NDD mutations [**51**].

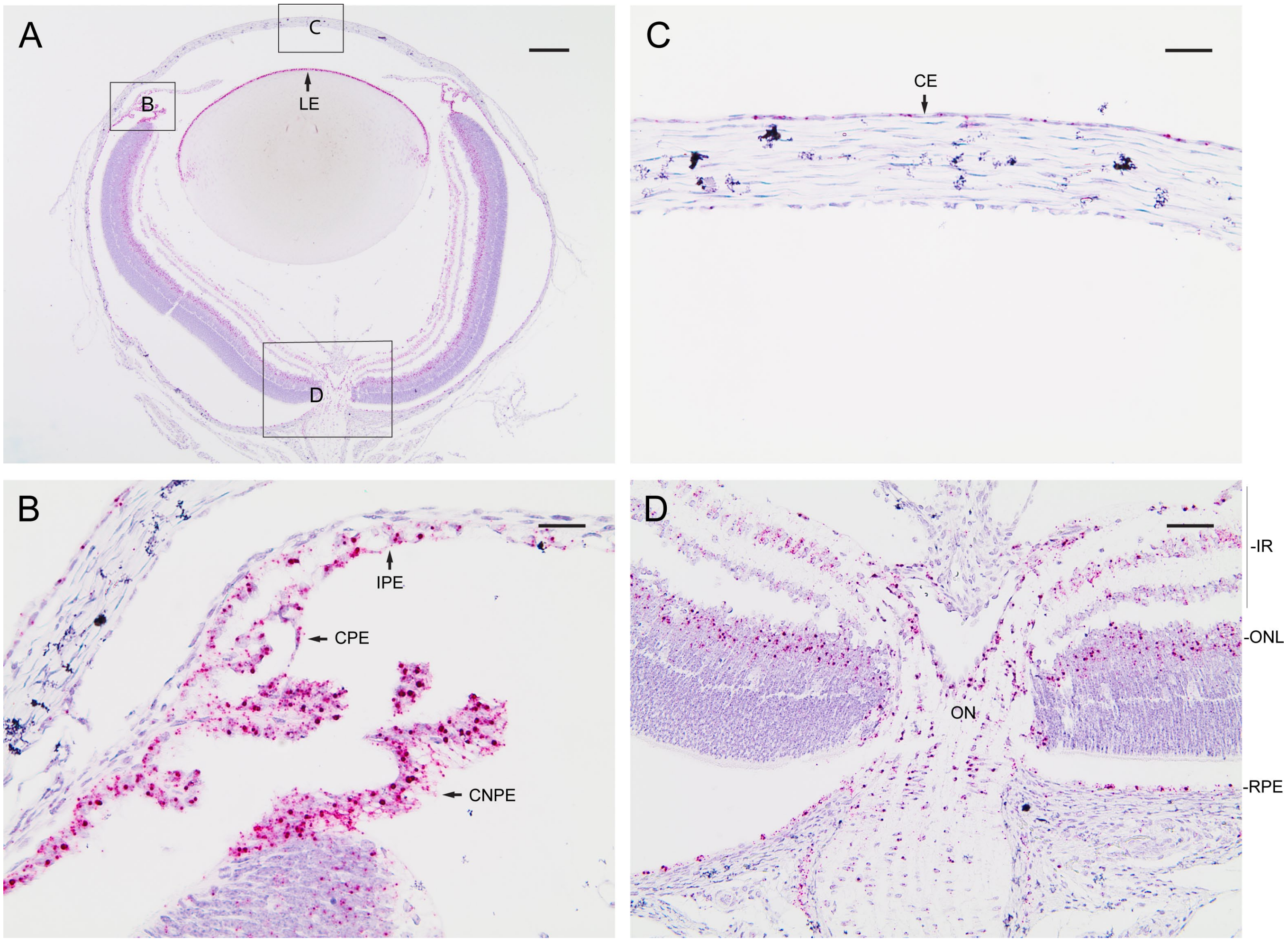

Figure S2. *In situ* hybridization (ISH) of *Trpm3*-transcripts in the neonatal mouse eye. Chromogenic labeling of *Trpm3* transcripts (red dots) in the albino mouse eye at P5 showing strong localization to the lens epithelium (LE) in (**A**), ciliary (pigmented) epithelium (CPE), ciliary non-pigmented epithelium (CNPE), and iris (pigmented) epithelium (IPE) in (**B**), corneal epithelium (CE) in (**C**), and retinal pigmented epithelium (RPE), outer nuclear layer (ONL), inner neuro-retina (IR), and optic nerve (ON) in (**D**). Cell nuclei were counterstained with hematoxylin (blue). Scale bar: 200 µm (A), 50 µm (B, C), 100 µm (D).

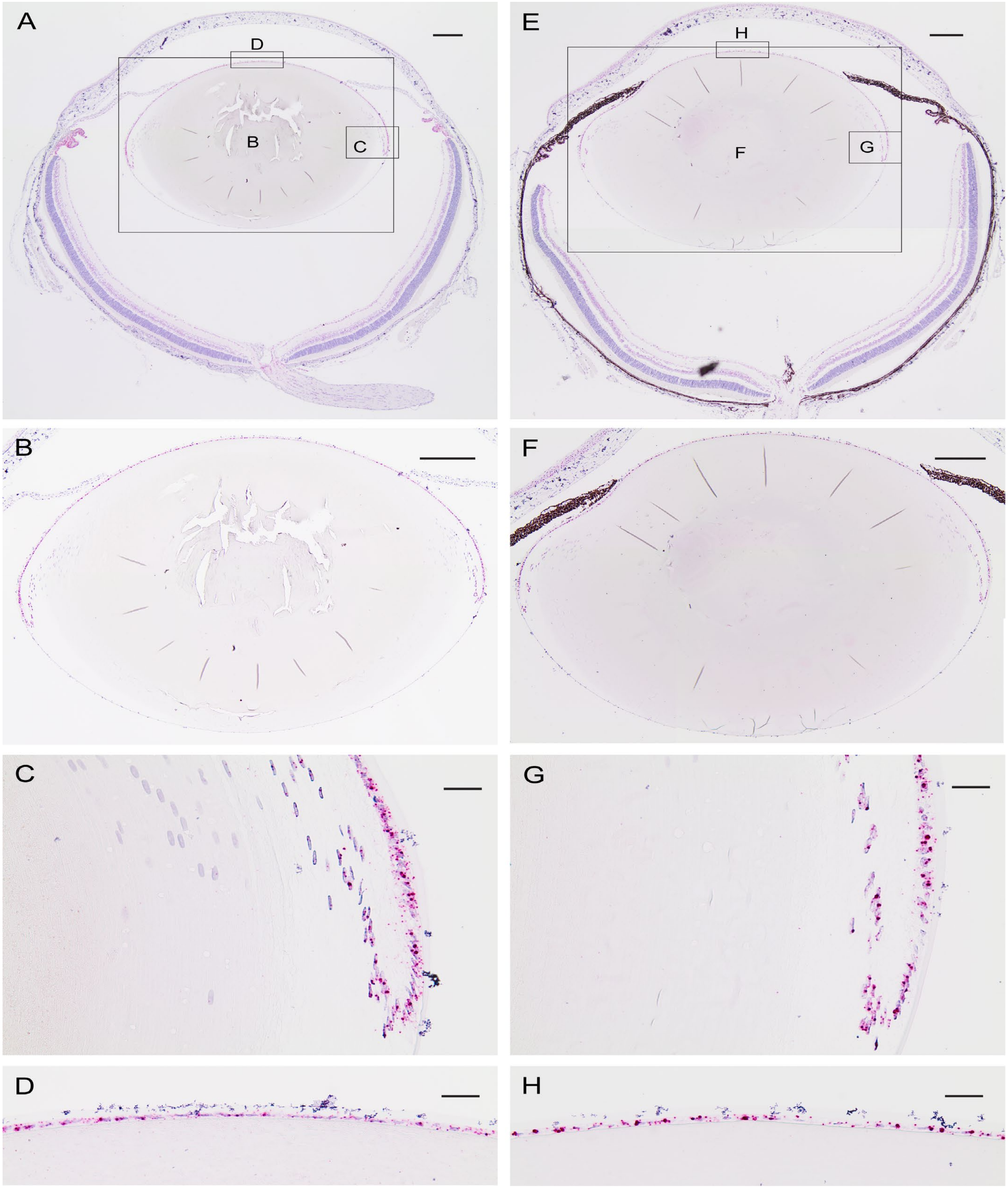

Figure S3. *In situ* hybridization of *Trpm3*-transcripts in the mature mouse lens. Chromogenic labeling of *Trpm3* transcripts (red dots) in the wild-type albino mouse eye at P21 (**A-D**) and the *Trpm3*-KO pigmented eye at P29 (**E-H**) showing localization to the whole lens epithelium (**B, F**), the equatorial lens epithelium (**C, G**), and the anterior lens epithelium (**D, H**). Cell nuclei were counterstained with hematoxylin (blue). Scale bar: 200 µm (A, B, E, F), 50 µm (C, D, G, H).

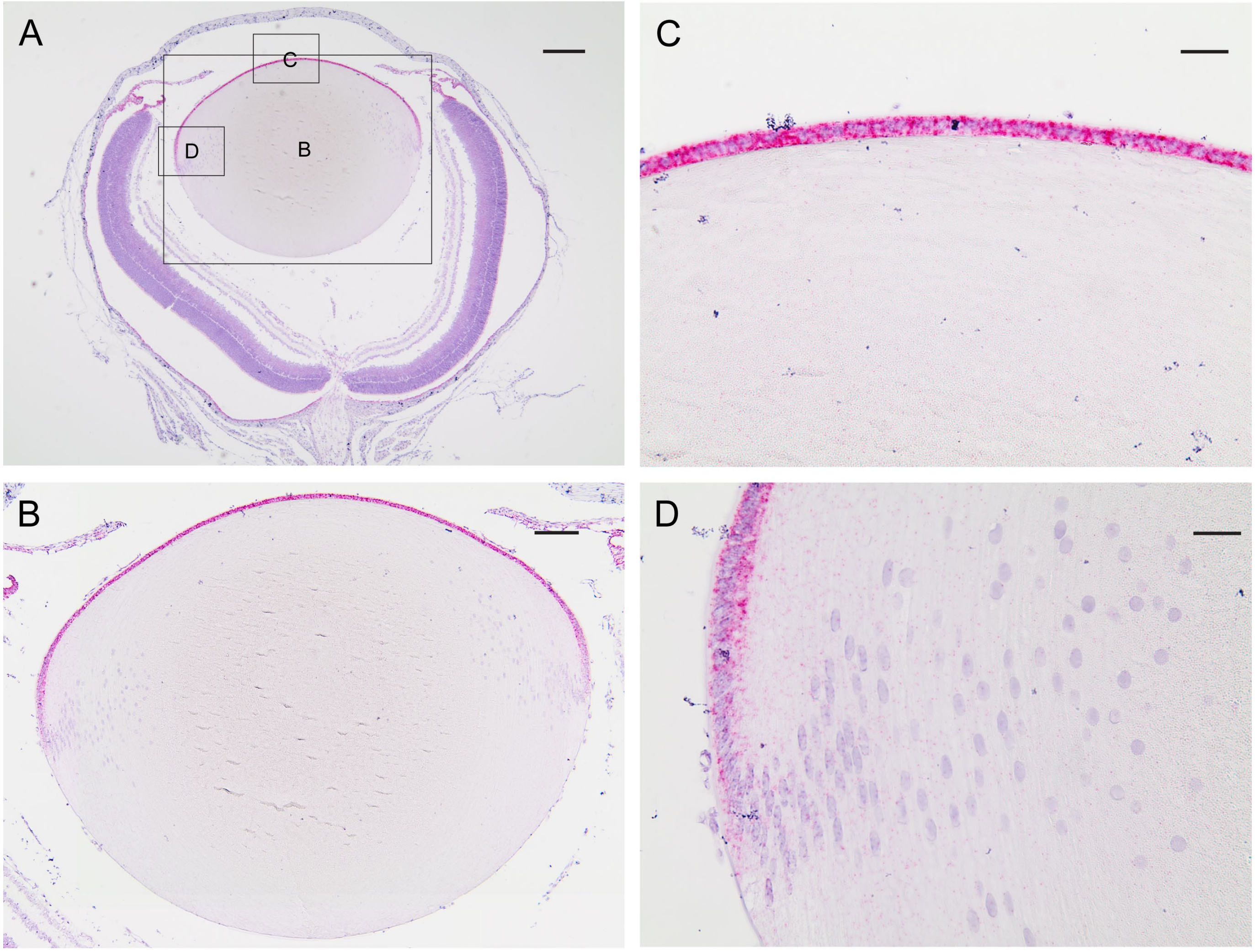

Figure S4. *In situ* hybridization of *Mir204* transcripts in the neonatal mouse lens. Chromogenic labeling of *Mir204* transcripts (red dots) in the albino mouse eye (**A**) and lens (**B-D**) at P5 showing strong localization to the lens anterior epithelium (**C**) and equatorial epithelium (**D**). Cell nuclei were counterstained with hematoxylin (blue). Scale bar: 200 µm (A), 100 µm (B), 50 µm (C, D).

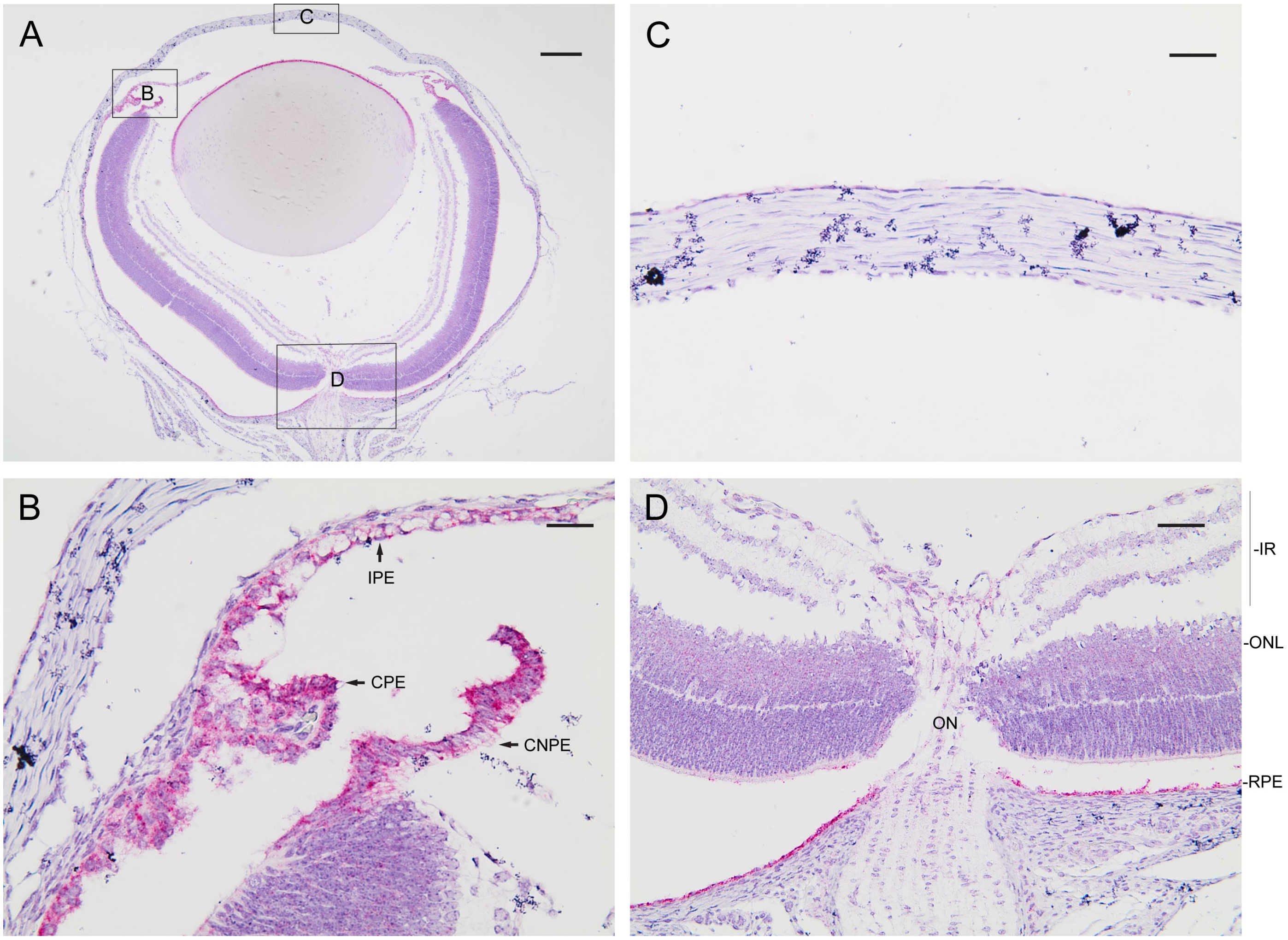

Figure S5. *In situ* hybridization of *Mir204*-transcripts in the neonatal mouse eye. Chromogenic labeling of *Mir204* transcripts (red dots) in the albino mouse eye (**A**) at P5 showing localization to the ciliary pigmented epithelium (CPE), ciliary non-pigmented epithelium (CNPE), and iris pigmented epithelium (IPE) (**B**), corneal epithelium (CE) (**C**), and retinal (pigmented) epithelium (RPE), outer nuclear layer (ONL), inner neuro-retina (IR), and optic nerve (ON) (**D**). Cell nuclei were counterstained with hematoxylin (blue). Scale bar: 200 µm (A), 100 µm (D), 50 µm (B, C).

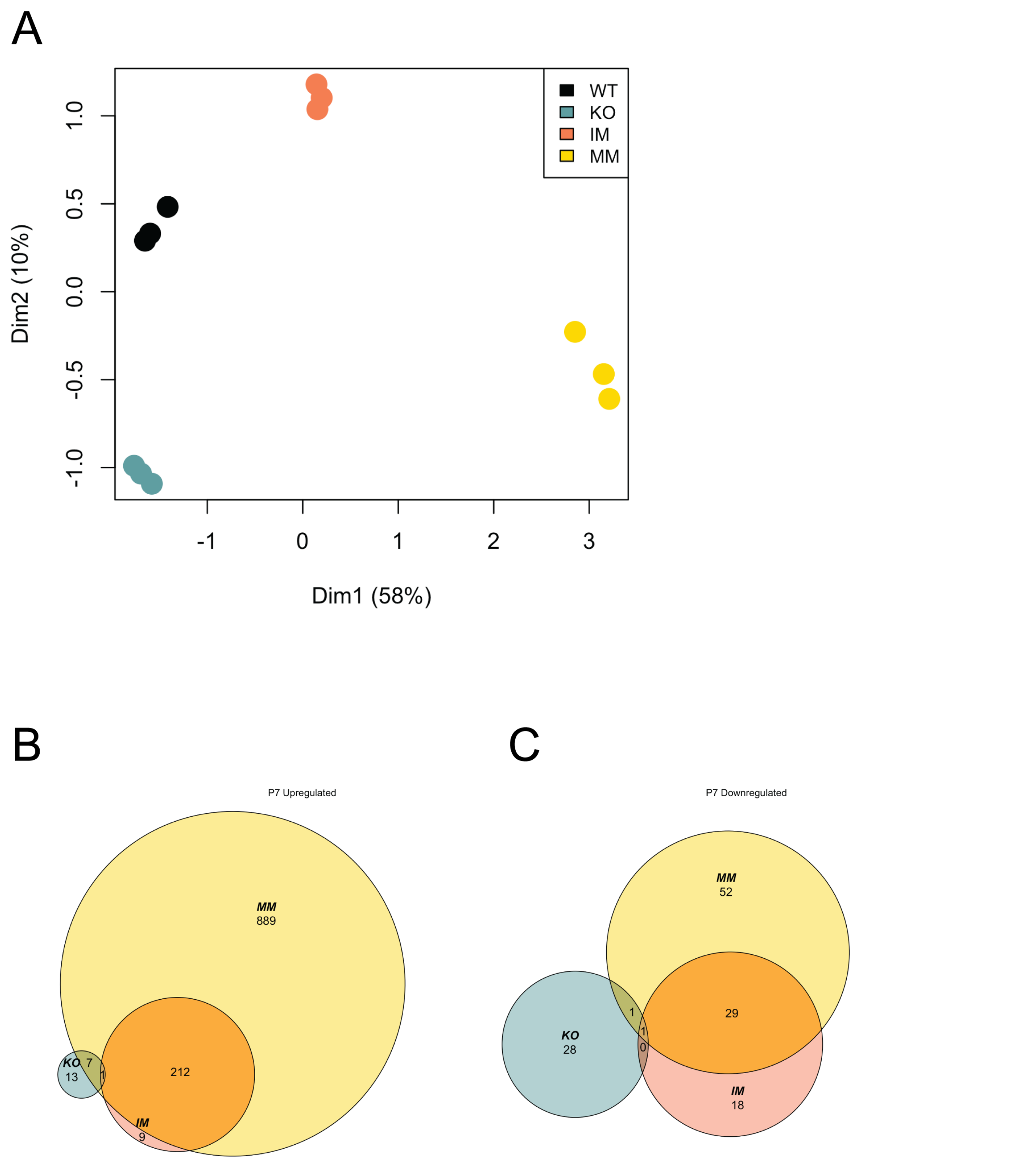

Figure S6. Multi-dimensional scaling (MDS) plot (**A**) and Venn diagrams of upregulated genes (**B**) and downregulated genes (**C**) in heterozygous *Trpm3*-I/M, homozygous *Trpm3*-M/M, and *Trpm3*-KO lenses versus wild-type lenses at P7.

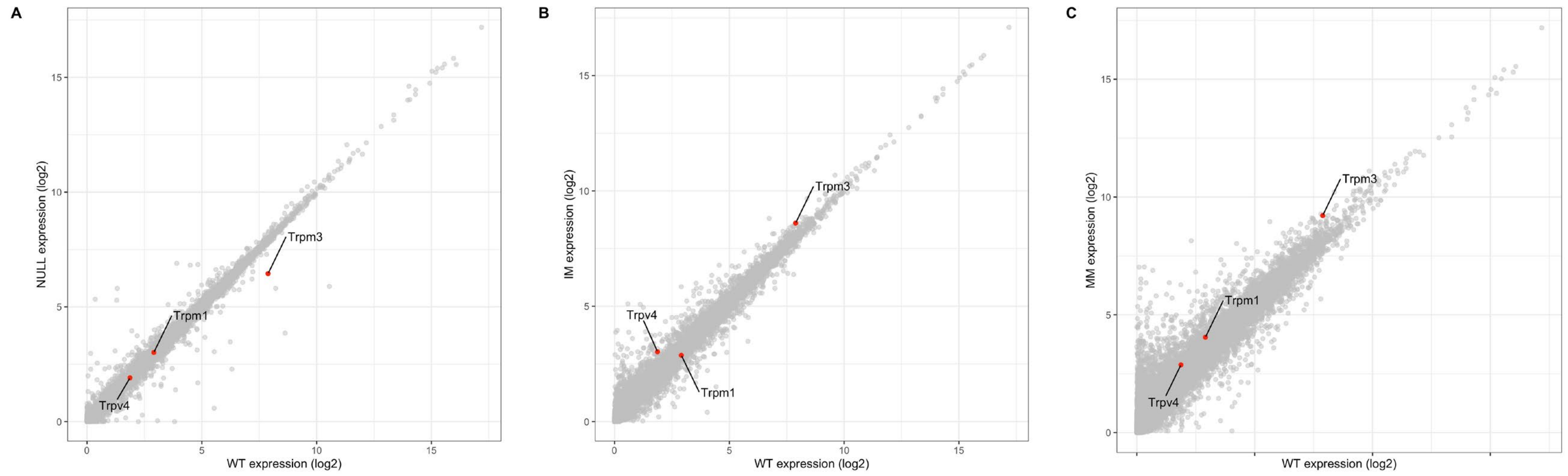

Figure S7. Scatterplot of differentially regulated genes in *Trpm3*-KO (A), *Trpm3*-I/M (B), and *Trpm3*-M/M (C) lenses highlighting the relative expression levels of *Trpm3* versus *Trpm1* and *Trpv4*.

Table S1. Primer sequences used for PCR-genotyping of *Mir204* -KO and *Mir204* ; *Mir211* -dKO mice

Primer name	Sequence (5' - 3')	Amplicon (bp)
Mir204 WT_F_S	TGGCTAAGATGCCGGAGAATCAAG	
Mir204 loxP_R148	GCTAGCAAGCTTTCGCGACGAGTC	148
Mir204 WT_R195_S	TGAACGTCCCTTTGCCTTCCCAG	195
Mir211_F	GACTCCTACTGTTCTGACCATGCAATCAC	
Mir211_KO_Cre_R458	TGGATCAGGGTGGCATCGTCTGTCTC	466/502
Mir211 WT-R	GTAGAGGTGACCACTGAGCC	368
Mir211 KO-F	CTAGCATAACTTCGTATAGC	177

Table S2. Differentially expressed genes (> 2-fold) in *Trpm3*-mutant lenses summarized in Fig. 9A.

unique	ensembl ge	entrezgene	external ge	cluster	gene biotyp	external ge	transcript c	description	p7.wt 1	p7.wt 2	p7.wt 3	p7.null 1	p7.null 2	p7.null 3	p7.im 1	p7.im 2	p7.im 3	p7.mm 1	p7.mm 2	p7.mm 3	p7.wt 1.cpn	p7.wt 2.cpn	p7.wt 3.cpn	p7.null 1.cp	p7.null 2.cp	p7.null 3.cp	p7.im 1.cpn	p7.im 2.cpn	p7.im 3.cpn	p7.mm 1.cp	p7.mm 2.cp	p7.mm 3.cp	logFC.Null.W	logCPM.Null	PValue.Null.	FDR.Null.WT	logFC.IM.WT	logCPM.IM.\	PValue.IM.W	FDR.IM.WT.I	logFC.MM.W	logCPM.MM	PValue.MM.	FDR.MM.WT.P7
10006	ENSMUSG00	233552	Gdpd5	1	protein codi	MGI Symbol	8	glycerophos	176	164	178	162	190	167	293	313	306	523	613	525	3.1271008	2.8487823	3.1643564	2.6630207	3.1101372	2.9279797	3.9819722	3.8066054	4.4538765	7.9762501	8.2826954	7.5365241	-0.070693	2.191319	0.5981849	0.909608	0.4193248	2.191319	0.0004857	0.0021445	1.3804275	2.191319	2.03E-33	2.64E-32
10010	ENSMUSG00	226154	Lzts2	1	protein codi	MGI Symbol	8	leucine zipp	884.86	793.6	749.86	856.83	781.73	780.78	1424.89	1499.76	1322.67	2000.71	2261.66	2083.79	15.721855	13.785327	13.330474	14.084914	12.79625	13.689269	19.364752	18.239599	19.251663	30.512741	30.558957	29.913397	-0.078462	4.2749043	0.4302231	0.8463211	0.4081781	4.2749043	2.53E-05	0.0001643	1.0867105	4.2749043	1.51E-29	1.67E-28
10013	ENSMUSG00	78610	Uvrag	1	protein codi	MGI Symbol	8	UV radiation	295	236	290	307	272	280	393	457.14	380.12	620	790	744	5.2414474	4.0994672	5.1554122	5.0465886	4.452407	4.9091875	5.3410071	5.5595898	5.5327044	9.4555929	10.674273	10.680331	-0.008466	2.6812708	0.9519457	1	0.1816448	2.6812708	0.1041689	0.1675876	1.0887282	2.6812708	1.55E-24	1.31E-23
10016	ENSMUSG00	55983	Pdzrn3	1	protein codi	MGI Symbol	5	PDZ domain	26	18	23	40	52	38	277	337	252	618	736	610	0.4619581	0.3126712	0.4088775	0.657536	0.8511954	0.6662469	3.7645267	4.0984857	3.6678983	9.425091	9.9446391	8.7567232	0.8786364	1.9010055	0.0001672	0.0042597	3.2820353	1.9010055	5.56E-82	1.04E-79	4.5662114	1.9010055	2.49E-184	4.63E-182
10046	ENSMUSG00	338367	Myo1d	1	protein codi	MGI Symbol	3	myosin ID [S	256	254	266	337	303	269	824	906	684	1151	1375	1282	4.5485103	4.4121384	4.7287574	5.5397406	4.9598504	4.7163266	11.198448	11.018481	9.955724	17.553851	18.57864	18.403474	0.1533773	3.2878846	0.184706	0.6501324	1.233264	3.2878846	4.46E-31	2.29E-29	1.994026	3.2878846	3.41E-78	1.29E-76
10062	ENSMUSG00	21810	Tgfbi	1	protein codi	MGI Symbol	5	transformin	18	18	17	25	29	22	634	360	408	3919	6398	6672	0.3198171	0.3126712	0.3022138	0.41096	0.4747052	0.3857219	8.6162812	4.3782043	5.9385021	59.768498	86.448099	95.778455	0.4437694	4.4697009	0.1126282	0.5253857	4.3280838	4.4697009	8.93E-144	4.04E-141	8.0074149	4.4697009	0	0
10065	ENSMUSG00	70335	Reep6	1	protein codi	MGI Symbol	7	receptor acc	600	562	534	825	730	681	1091	1141	921	1885	2105	1857	10.660571	9.7622905	9.4930693	13.561679	11.949475	11.939845	14.827071	13.876475	13.405295	28.748053	28.442208	26.657762	0.3243185	4.018132	0.0013442	0.0258157	0.4933139	4.018132	7.39E-07	6.83E-06	1.4867006	4.018132	9.57E-52	2.17E-50
10069	ENSMUSG00	171171	Ntng2	1	protein codi	MGI Symbol	11	netrin G2 [S	639.86	549.72	669.26	808.73	782.65	760	1293.11	1343.32	1173.14	2432.09	2900.4	2603	11.368788	9.5489793	11.897625	13.294227	12.81131	13.324938	17.573816	16.337026	17.075231	37.091698	39.189445	37.366804	0.2651091	4.3111322	0.0082983	0.1091876	0.6356866	4.3111322	1.09E-10	1.66E-09	1.7923456	4.3111322	3.52E-75	1.27E-73
10104	ENSMUSG00	237422	Ric8b	1	protein codi	MGI Symbol	5	RIC8 guanin	463	388	412	484	507	431	700	765	650	895	1143	1158	8.2264072	6.739802	7.3242407	7.9561853	8.2991556	7.5566422	9.5132443	9.3036842	9.4608489	13.649606	15.443916	16.623419	0.095952	3.3339516	0.3704062	0.8085781	0.343299	3.3339516	0.0009042	0.0036198	1.0368869	3.3339516	1.44E-24	1.22E-23
10119	ENSMUSG00	216161	Sbno2	1	protein codi	MGI Symbol	9	strawberry r	232	180	192	229	248	230	297	295	296	801	1074	980	4.1220874	3.1267123	3.4132384	3.7643935	4.0595475	4.0325469	4.0363336	3.5876952	4.308325	12.216016	14.511607	14.068178	0.15346	2.6690451	0.2112634	0.6879531	0.1599786	2.6690451	0.1796274	0.2587898	1.9367047	2.6690451	3.27E-69	1.06E-67
10124	ENSMUSG00	21835	Thrsp	1	protein codi	MGI Symbol	2	thyroid horn	90	95	104	149	112	83	377	442	286	582	902	519	1.5990856	1.6502093	1.8488375	2.4493215	1.833344	1.4552234	5.1235615	5.375462	4.1627735	8.8760566	12.187588	7.4503924	0.1749506	2.2121788	0.258348	0.73241	1.5264662	2.2121788	1.16E-32	6.28E-31	2.484545	2.2121788	1.58E-90	7.07E-89
10136	ENSMUSG00	269060	Dagla	1	protein codi	MGI Symbol	3	diacylglycer	152	139	160	173	207	170	263	328	264	428	424	363	2.700678	2.4145167	2.8443653	2.8438431	3.3884126	2.9805781	3.5742618	3.9890306	3.8425602	6.5274093	5.7289769	5.2109681	0.2118745	1.964783	0.1037643	0.5056472	0.5200833	1.964783	2.38E-05	0.0001559	1.1326433	1.964783	1.66E-21	1.19E-20
10150	ENSMUSG00	14594	Ggta1	1	protein codi	MGI Symbol	9	glycoprotein	522	477	479	541	503	503	1474	1700	1263	2173	2689	2818	9.2746967	8.2857875	8.5153187	8.8931741	8.233679	8.8190047	20.032174	20.674854	18.383157	33.140328	36.333063	40.45319	-0.007254	4.2148039	0.9517325	1	1.1806716	4.2148039	1.59E-32	8.56E-31	2.0758028	4.2148039	1.68E-97	8.64E-96
10153	ENSMUSG00	11475	Acta2	1	protein codi	MGI Symbol	2	actin, alpha	3386	3607	3158	2816	2627	2553	7237	8348.15	5025	13544.44	23397.76	18770	60.161155	62.65584	56.140661	46.290533	43.001739	44.76127	98.353355	101.52752	73.139639	206.56566	316.1444	269.44868	-0.416827	6.8456865	6.29E-06	0.0002361	0.6096638	6.8456865	2.77E-11	4.58E-10	2.1462198	6.8456865	9.78E-115	7.09E-113
10163	ENSMUSG00	223775	Pim3	1	protein codi	MGI Symbol	2	proviral inte	399	432	463	425	475	457	867	953	822	1179	1340	1148	7.0892797	7.5041095	8.2308822	6.9863197	7.775343	8.0124953	11.782833	11.59008	11.964335	17.980877	18.105729	16.479866	-0.003586	3.4878455	0.9826677	1	0.6303167	3.4878455	6.42E-10	9.15E-09	1.2034315	3.4878455	6.49E-33	8.22E-32
1017	ENSMUSG00	228026	Pdk1	1	protein codi	MGI Symbol	8	pyruvate del	839	804	908	798	922	854	1300	1342	953	2217	3333	3001	14.907032	13.965981	16.141773	13.117843	15.09235	14.973022	17.667454	16.320973	13.87106	33.81137	45.034623	43.080207	-0.059984	4.4326055	0.5454439	0.8906445	0.0892424	4.4326055	0.3628079	0.455247	1.4380132	4.4326055	1.05E-50	2.32E-49
10181	ENSMUSG00	114774	Pawr	1	protein codi	MGI Symbol	2	PRKC, apopt	145	103	157	131	136	121	270	274	268	301	401	420	2.5763046	1.7891742	2.7910335	2.1534303	2.2262035	2.1214703	3.6693942	3.3323	3.9007808	4.5905378	5.4182069	6.0292193	-0.136488	1.7900651	0.3229684	0.777949	0.6058157	1.7900651	1.26E-06	1.10E-05	1.1664904	1.7900651	8.33E-22	6.11E-21
10192	ENSMUSG00	195208	Dcdc2a	1	protein codi	MGI Symbol	4	doublecortir	61	62	59	82	89	61	460	472	387	677	887	944	1.0838247	1.0769787	1.0488597	1.3479487	1.4568537	1.0695016	6.2515605	5.7403124	5.6328439	10.324897	11.984912	13.551388	0.2747951	2.3800689	0.113552	0.5277611	2.4550078	2.3800689	1.37E-72	2.28E-70	3.4801559	2.3800689	3.45E-155	4.20E-153
1020	ENSMUSG00	13057	Cyba	1	protein codi	MGI Symbol	3	cytochrome	225	231	222	266	239	201	363	410	394	611	693	682	3.9977141	4.0126141	3.9465569	4.3726142	3.9122252	3.5240953	4.9332967	4.9862883	5.7347299	9.3183343	9.3636344	9.7903037	-0.016614	2.5196021	0.9018796	0.9896888	0.3867806	2.5196021	0.0006732	0.0028473	1.2513002	2.5196021	5.21E-30	5.91E-29
10210	ENSMUSG00	238130	Dock4	1	protein codi	MGI Symbol	11	dedicator of	227	191	201	216	274	241	283	325	290	532	626	607	4.0332493	3.3177891	3.573234	3.5506943	4.4851452	4.2254078	3.8460687	3.9525456	4.2209941	8.1135088	8.458348	8.7136574	0.166559	2.3513367	0.1664786	0.6233079	0.1373058	2.3513367	0.2530094	0.3408863	1.2110415	2.3513367	2.59E-27	2.58E-26
10219	ENSMUSG00	216742	Fnip1	1	protein codi	MGI Symbol	2	folliculin int	214	173	233	208	240	201	346	400	347	544	608	606	3.8022703	3.0051179	4.142107	3.4191871	3.9285944	3.5240953	4.7022607	4.8646715	5.0506378	8.2965202	8.2151367	8.6993021	-0.008912	2.3818594	0.954937	1	0.4171178	2.3818594	0.000296	0.0014051	1.2035738	2.3818594	4.96E-27	4.88E-26
10235	ENSMUSG00	71409	Fmnl2	1	protein codi	MGI Symbol	5	formin-like 2	750	809	842	802	969	805	1712	1808	1475	2901	3509	3323	13.325714	14.052835	14.968473	13.183596	15.8617	14.113914	23.266677	21.988315	21.468849	44.243024	47.412688	47.702609	0.027549	4.6099511	0.7847774	0.9653561	0.6559393	4.6099511	1.12E-11	1.94E-10	1.7184394	4.6099511	5.01E-71	1.71E-69
10249	ENSMUSG00	226151	Slf2	1	protein codi	MGI Symbol	5	SMC5-SMC6	403	335	327.86	402	397	452	613.07	714.07	626.98	863	1023.92	916	7.1603501	5.819159	5.8284601	6.6082366	6.4985499	7.9248313	8.3318352	8.6842899	9.1257893	13.161575	13.834939	13.14944	0.1601796	3.1570907	0.139401	0.5747489	0.4747729	3.1570907	6.25E-06	4.68E-05	1.0941454	3.1570907	2.40E-26	2.29E-25
10252	ENSMUSG00	140792	Colec12	1	protein codi	MGI Symbol	4	collectin sub	478	457	553	241	245	252	761	785	698	1260	1528	1463	8.4929215	7.938375	9.8308377	3.9616543	4.0104401	4.4182688	10.342256	9.5469178	10.159496	19.216205	20.645936	21.00178	-1.084018	3.4458908	1.28E-22	2.53E-20	0.1939971	3.4458908	0.0573619	0.1039166	1.2129138	3.4458908	3.39E-34	4.51E-33
10288	ENSMUSG00	98402	Sh3bp4	1	protein codi	MGI Symbol	2	SH3-domain	286	265	306	283	298	285	620	726	593	947	1111	971	5.0815388	4.6032153	5.4398487	4.652067	4.8780047	4.9968516	8.4260163	8.8293788	8.6312052	14.442656	15.011541	13.938981	-0.058145	3.0617184	0.6155773	0.9155107	0.7757003	3.0617184	4.08E-13	8.16E-12	1.5207305	3.0617184	3.74E-48	7.69E-47
10309	ENSMUSG00	107227	Macrod1	1	protein codi	MGI Symbol	1	mono-ADP r	282	258	259	298	276	244	500	497	402	800	1105	842	5.0104683	4.4816209	4.6043164	4.898643	4.5178835	4.2780063	6.7951745	6.0443543	5.8511711	12.200765	14.93047	12.087149	-0.040763	2.8547401	0.7320916	0.9514929	0.4071775	2.8547401	0.0002216	0.0010907	1.4769719	2.8547401	1.80E-43	3.32E-42
1032	ENSMUSG00	20536	Slc4a3	1	protein codi	MGI Symbol	12	solute carrie	295	230	256	369	408	348	339	476	334	600	740	624	5.2414474	3.9952435	4.5509845	6.0657694	6.6786104	6.1014188	4.6071283	5.7889591	4.8614208	9.1505738	9.9986861	8.9576972	0.4515703	2.6754539	5.66E-05	0.0016563	0.1493797	2.6754539	0.1890814	0.2694679	1.0283442	2.6754539	2.44E-21	1.74E-20
10328	ENSMUSG00	242050	Igsf10	1	protein codi	MGI Symbol	4	immunoglob	192.54	123.51	211.68	172.99	167.37	156.2	323.31	458.68	409.06	548.4	705.78	797.49	3.4209772	2.1454457	3.7630953	2.8436787	2.7397035	2.7386253	4.3938957	5.5783188	5.9539305	8.3636244	9.5363144	11.448195	-0.162865	2.4181154	0.2068904	0.6796504	0.7724238	2.4181154	3.82E-11	6.16E-10	1.6551891	2.4181154	1.85E-48	3.81E-47
10351	ENSMUSG00	14751	Gpi1	1	protein codi	MGI Symbol	11	glucose-6-pl	4601.84	4141.66	4428.74	4892.78	4776.63	4876.57	7859.55	8612.95	6976.34	14421.75	19249.54	17271.43	81.763736	71.943217	78.730966	80.429472	78.189341	85.499988	106.81403	104.74793	101.54169	219.94548	260.09474	247.93628	0.0707081	6.9839824	0.4389988	0.8505994	0.4298396	6.9839824	2.38E-06	1.98E-05	1.6470999	6.9839824	1.44E-70	4.83E-69
10361	ENSMUSG00	71302	Arhgap26	1	protein codi	MGI Symbol	12	Rho GTPase	173.99	163	159.99	184	161.92	178	289.47	356	246.14	388.4	465.48	413	3.0913879	2.8314117	2.8441876	3.0246655	2.6504917	3.1208406	3.9339983	4.3295576	3.5826051	5.9234714	6.2894438	5.9287323	0.0038421	2.0118174	1	1	0.4372118	2.0118174	0.0003103	0.0014628	1.0493277	2.0118174	3.57E-19	2.19E-18
10363	ENSMUSG00	13709	Elf1	1	protein codi	MGI Symbol	4	E74-like fact	171.78	123.97	157.29	121.48	169.42	145.35	259.11	310.87	264.85	320.66	390.23	411.6	3.0521215	2.1534362	2.7961889	1.9969368	2.7732602	2.5483943	3.5213953	3.7807011	3.854932	4.8903716	5.2726855	5.9086349	-0.128116	1.854119	0.3374655	0.7875273	0.4802681	1.854119	9.86E-05	0.0005428	1.0073895	1.854119	4.21E-17	2.24E-16
10367	ENSMUSG00	224617	Tbc1d24	1	protein codi	MGI Symbol	15	TBC1 domai	225	189	164	242	238	230	311	372	320.67	462	504	504	3.9977141	3.2830479	2.9154745	3.9780927	3.8958561	4.0325469	4.2265985	4.5241445	4.6674006	7.0459418	6.8099159	7.2350631	0.2235123	2.2572973	0.0674583	0.4097528	0.3958215	2.2572973	0.0007499	0.0031042	1.0480573	2.2572973	3.47E-20	2.29E-19
10396	ENSMUSG00	234593	Ndrg4	1	protein codi	MGI Symbol	11	N-myc dowr	1210	1209	1192	1434	1391	1383	2162	2218	2158	5332	8633	8180	21.498818	21.001084	21.190522	23.572665	22.769478	24.24788	29.382334	26.974603	31.410018	81.318099	116.64683	117.42622	0.1482763	5.4895274	0.1208469	0.5394952	0.4620993	5.4895274	1.02E-06	9.09E-06	2.3081293	5.4895274	2.58E-127	2.25E-125
10399	ENSMUSG00	56188	Fxyd1	1	protein codi	MGI Symbol	17	FXYD domai	594	695	614	776	756	733	1161	1144	974	1592	2136	2068	10.553965	12.072583	10.915252	12.756198	12.375072	12.851552	15.778395	13.91296	14.176718	24.279522	28.861072	29.686727	0.1790718	4.0537363	0.074747	0.4322417	0.3867651	4.0537363	9.10E-05	0.0005049	1.3042113	4.0537363	9.82E-41	1.66E-39
10417	ENSMUSG00	208618	Etl4	1	protein codi	MGI Symbol	21	enhancer tra	4661	3585	3822.93	4300	4654	4588.97	6707.89	8596.88	6179.94	10093.86	11743.98	11451.92	82.814869	62.273686	67.96131	70.685117	76.181993	80.457551	91.162566	104.55249	89.949967	153.94102	158.68158	164.39556	0.0935437	6.6489003	0.306576	0.7652318	0.423343	6.6489003	3.49E-06	2.78E-05	1.1628991	6.6489003	1.06E-36	1.57E-35
10418	ENSMUSG00	103534	Mgat4b	1	protein codi	MGI Symbol	5	mannoside a	873	817	860	895	925	912	1656	1791	1495	3332	4278	3859	15.511131	14.1918	15.288464	14.712367	15.141457	15.989925	22.505618	21.781567	21.759952	50.816186	57.803215	55.397041	0.0269372	4.7476179	0.7872061	0.9660842	0.5538735	4.7476179	9.04E-09	1.13E-07	1.8662429	4.7476179	1.39E-83	5.65E-82
10434	ENSMUSG00	223921	Aaas	1	protein codi	MGI Symbol	13	achalasia, ac	191	173	197	199	224	211	351	348	345	464	643	647	3.3936151	3.0051179	3.5021248	3.2712415	3.6666881	3.6994234	4.7702125	4.2322642	5.0215275	7.0764437	8.6880475	9.2878687	0.1033493	2.3352087	0.418471	0.8410143	0.5000232	2.3352087	1.96E-05	0.0001308	1.3405709	2.3352087	2.37E-32	2.93E-31
10449	ENSMUSG00	70892	Ttll7	1	protein codi	MGI Symbol	6	tubulin tyros	268	372	239	315	348	362	380	403	343	898	972	894	4.7617217	6.461872	4.2487707	5.1780958	5.6964618	6.3468781	5.1643326	4.9011565	4.9924172	13.695359	13.133409	12.833624	0.1523054	2.8777021	0.1727791	0.6310968	-0.040528	2.8777021	0.7292627	0.7894252	1.3565061	2.8777021	1.42E-38	2.25E-37
10476	ENSMUSG00	245847	Amdhd2	1	protein codi	MGI Symbol	8	amidohydrol	331	266	333	355	317	315	567	653	544	700	941	762	5.8810816	4.6205859	5.9198354	5.8356318	5.1890184	5.522836	7.7057278	7.9415762	7.9180027	10.675669	12.714545	10.938726	0.0115799	2.936391	0.9298445	0.9943662	0.5216088	2.936391	1.08E-06	9.60E-06	1.0651004	2.936391	3.06E-24	2.55E-23
10483	ENSMUSG00	66340	Psenen	1	protein codi	MGI Symbol	7	presenilin er	1334.34	1290.45	1362.91	1506.28	1547.56	1391.34	2219.33	2398.53	2025.6	2876.54	3658.63	3580.61	23.708044	22.415921	24.228837	24.760832	25.332231	24.394103	30.161469	29.170151	29.482916	43.869986	49.43445	51.400673	0.0824943	4.9824778	0.3872763	0.8221507	0.336175	4.9824778	0.0003615	0.0016696	1.0405966	4.9824778	1.98E-28	2.08E-27
10488	ENSMUSG00	243912	Hspb6	1	protein codi	MGI Symbol	4	heat shock p	1287	1434	1211	1563	1667	1541	2390	2376	1954	4409	4968	4757	22.866925	24.909474	21.52829	25.693218	27.287362	27.018064	32.480934	28.896149	28.440767	67.241466	67.126314	68.288086	0.2068677	5.2060874	0.0293574	0.2604066	0.3739157	5.2060874	7.28E-05	0.0004138	1.5478234	5.2060874	2.95E-60	8.17E-59
10564	ENSMUSG00	15275	Hk1	1	protein codi	MGI Symbol	20	hexokinase 1	755	698.59	666.3	764.14	850.77	894.33	1922.82	2022.7	1750.69	3949.33	4476.74	4310.81	13.414552	12.134944	11.845004	12.561239	13.926376	15.68012	26.131795	24.599428	25.481559	60.231059	60.488538	61.882902	0.172718	4.8248942	0.0821872	0.4558759	1.0270032	4.8248942	3.34E-26	1.44E-24	2.2876858	4.8248942	1.99E-121	1.57E-119
10647	ENSMUSG00	29876	Clic4	1	protein codi	MGI Symbol	2	chloride intr	290	222	204	257	269	258	359	420	402	1217	1424.99	1336	5.1526093	3.8562785	3.6265658	4.2246686	4.4032995	4.5234656	4.8789353	5.1079051	5.8511711	18.560414	19.254091	19.178659	0.057693	3.0572328	0.6321123	0.9225563	0.3245195	3.0572328	0.0039391	0.0120405	2.1732778	3.0572328	5.13E-91	2.33E-89
1065	ENSMUSG00	72043	Sulf2	1	protein codi	MGI Symbol	7	sulfatase 2 [	393.01	240	378.07	468.05	463.03	447.29	705.17	547.07	594.12	1976.28	2492.65	2561.2	6.9828516	4.1689497	6.7210575	7.6939928	7.5794044	7.8422517	9.5835064	6.6532896	8.647507	30.14016	33.680034	36.766753	0.3719362	3.806612	0.0005747	0.0124073	0.475876	3.806612	6.68E-06	4.97E-05	2.4935395	3.806612	4.29E-133	4.24E-131
10656	ENSMUSG00	52120	Hgsnat	1	protein codi	MGI Symbol	4	heparan-alp	893	867	510	1678	1659	1490	2011	1720	1258	2340	2067	2193	15.866483	15.060331	9.0664145	27.583634	27.156409	26.123891	27.330192	20.918087	18.310381	35.687238	27.928762	31.481138	1.0154409	4.561357	1.21E-25	2.75E-23	0.7347986	4.561357	3.23E-14	6.99E-13	1.2487263	4.561357	2.92E-38	4.58E-37
10667	ENSMUSG00	100017	Ldlrap1	1	protein codi	MGI Symbol	3	low density l	695	679	676	852	779	787	1470	1672	1435	1881	2143	1940	12.348495	11.794654	12.017444	14.005516	12.751563	13.798323	19.977813	20.334327	20.886643	28.687049	28.955654	27.849251	0.1653605	4.2264058	0.0977482	0.4904394	0.7589381	4.2264058	7.10E-15	1.60E-13	1.2413014	4.2264058	2.28E-37	3.46E-36
10690	ENSMUSG00	71904	Paqr7	1	protein codi	MGI Symbol	6	progestin an	1531	1351	1233	1329	1334	1294	3562	3830	3174	10146	12984	12116	27.202223	23.467713	21.91939	21.846633	21.836437	22.687459	48.408823	46.57923	46.198053	154.7362	175.43641	173.92862	-0.129264	6.0340474	0.1756921	0.6335282	0.9597524	6.0340474	1.57E-24	6.35E-23	2.7958697	6.0340474	1.48E-181	2.61E-179
1071	ENSMUSG00	66180	P3h4	1	protein codi	MGI Symbol	2	prolyl 3-hydr	1228	1253	1367	1461	1416	1490	2997	3026	2481	3188	3994	3766	21.818635	21.765392	24.301546	24.016502	23.178707	26.123891	40.730276	36.80124	36.111332	48.620049	53.965881	54.061999	0.1108855	5.1042897	0.2447	0.72048	0.7433407	5.1042897	2.86E-15	6.72E-14	1.2064654	5.1042897	1.74E-37	2.66E-36
10720	ENSMUSG00	71946	Endod1	1	protein codi	MGI Symbol	3	endonucleas	207	224	223	246	224	264	401	432	364	818	1017	826	3.677897	3.8910197	3.9643342	4.0438462	3.6666881	4.6286625	5.4497299	5.2538452	5.2980754	12.475282	13.741438	11.857465	0.09564	2.7198894	0.4295302	0.8463211	0.4719974	2.7198894	3.47E-05	0.0002173	1.7229111	2.7198894	5.55E-56	1.41E-54
10731	ENSMUSG00	214855	Arid5a	1	protein codi	MGI Symbol	15	AT rich inter	168	140	123	178	151	167	278	263	264	425	483	518	2.9849599	2.4318873	2.1866059	2.9260351	2.4717406	2.9279797	3.778117	3.1985215	3.8425602	6.4816564	6.5261694	7.4360371	0.1300564	2.0020799	0.3273391	0.7793548	0.5055658	2.0020799	4.57E-05	0.0002749	1.4265158	2.0020799	1.98E-33	2.57E-32
10737	ENSMUSG00	21847	Klf10	1	protein codi	MGI Symbol	6	Kruppel-like	167	181	281	189	229	228	331	335	275	563	765	828	2.9671922	3.1440829	4.9954166	3.1068575	3.7485338	3.9974813	4.4984055	4.0741624	4.0026668	8.5862884	10.33648	11.886175	-0.03397	2.4656455	0.7931155	0.9685832	0.1798607	2.4656455	0.1289934	0.1988378	1.4731899	2.4656455	1.54E-40	2.59E-39
10760	ENSMUSG00	192786	Rapgef6	1	protein codi	MGI Symbol	13	Rap guanine	207	157	196	165	215	194	270	313	261	400	512	522	3.677897	2.7271879	3.4843475	2.7123359	3.5193658	3.4013656	3.6693942	3.8066054	3.7988947	6.1003825	6.9180098	7.4934582	-0.037738	2.117394	0.7740088	0.9623913	0.1900827	2.117394	0.1163406	0.1831377	1.0538958	2.117394	3.13E-20	2.07E-19
10762	ENSMUSG00	78938	Fbxo34	1	protein codi	MGI Symbol	14	F-box protei	329	323.94	360.92	555.23	567.86	555.5	885.89	1022.99	812.96	1704.62	1999.96	1822.94	5.8455464	5.6270399	6.4161771	9.1270925	9.2953817	9.7394774	12.039554	12.441276	11.832756	25.997085	27.022935	26.168821	0.6544368	3.7635824	7.19E-10	4.88E-08	1.0217911	3.7635824	6.11E-23	2.32E-21	2.1461837	3.7635824	4.38E-99	2.32E-97
10821	ENSMUSG00	12520	Cd81	1	protein codi	MGI Symbol	6	CD81 antige	2650	2551	2453	2679	2713	2578	4500	4714	3816	5612	7221	6920	47.084188	44.312461	43.607676	44.038472	44.409486	45.199591	61.15657	57.330154	55.54246	85.588367	97.56826	99.338565	-0.01459	5.9194242	0.8763171	0.9850516	0.3663532	5.9194242	6.94E-05	0.0003975	1.065294	5.9194242	9.83E-31	1.15E-29
10852	ENSMUSG00	232023	Vopp1	1	protein codi	MGI Symbol	6	vesicular, ov	239	222	221	229	245	241	458	558	487	851	987	1006	4.2464608	3.8562785	3.9287796	3.7643935	4.0104401	4.2254078	6.2243798	6.7862167	7.0883591	12.978564	13.336085	14.441416	-0.004308	2.8436457	1	1	0.7398759	2.8436457	3.35E-11	5.48E-10	1.7599972	2.8436457	3.68E-59	9.97E-58
1086	ENSMUSG00	114584	Clic1	1	protein codi	MGI Symbol	2	chloride intr	1026	995	1011	1224	1174	1186	1615	1728	1416	2308	2749	2689	18.229576	17.283771	17.972833	20.120601	19.217374	20.793916	21.948414	21.015381	20.610095	35.199207	37.143768	38.601359	0.1688352	4.5901264	0.0805639	0.4515381	0.2493429	4.5901264	0.0092738	0.0241504	1.0526622	4.5901264	1.97E-28	2.07E-27
10917	ENSMUSG00	18081	Ninj1	1	protein codi	MGI Symbol	5	ninjurin 1 [S	507	481	525	606	579	555	1308	1394	1119	1666	2184	1842	9.0081824	8.35527	9.3330738	9.96167	9.4777339	9.730711	17.776176	16.95338	16.287215	25.408093	29.509636	26.442433	0.1279648	3.9826557	0.2172776	0.6949955	0.9344994	3.9826557	6.73E-21	2.32E-19	1.6079954	3.9826557	2.15E-59	5.84E-58
10933	ENSMUSG00	76376	Slc24a2	1	protein codi	MGI Symbol	9	solute carrie	265	239	280	256	308	307	381	467	339	653	696	713	4.7084188	4.1515791	4.9776393	4.2082302	5.0416961	5.3825735	5.1779229	5.679504	4.9341966	9.9588745	9.4041696	10.235317	0.0798066	2.6369214	0.4925184	0.8711141	0.1928363	2.6369214	0.0880517	0.146727	1.0967053	2.6369214	3.05E-24	2.55E-23
10966	ENSMUSG00	106878	Smim3	1	protein codi	MGI Symbol	2	small integra	245	266	342	204	213	186	383	402	305	590	817	775	4.3530665	4.6205859	6.0798309	3.3534335	3.4866275	3.2611031	5.2051036	4.8889948	4.4393214	8.9980642	11.03909	11.125345	-0.574443	2.5796855	1.23E-06	5.30E-05	-0.049295	2.5796855	0.6632813	0.7345736	1.0508754	2.5796855	3.97E-23	3.12E-22
10990	ENSMUSG00	57810	Cdon	1	protein codi	MGI Symbol	6	cell adhesior	831	751	792	905	938	928	1463	1729	1349	2176	2072	1903	14.764891	13.045338	14.079608	14.876751	15.354256	16.27045	19.88268	21.027543	19.6349	33.186081	27.996321	27.318105	0.150534	4.312611	0.1267459	0.5491396	0.5317969	4.312611	4.05E-08	4.62E-07	1.0787038	4.312611	4.54E-29	4.91E-28
11012	ENSMUSG00	210530	P3h2	1	protein codi	MGI Symbol	4	prolyl 3-hydr	1598.04	1359.75	1482.49	1704.37	1631.55	1666.22	2934.83	2499.48	2354.14	4542.54	5417.87	4963.17	28.393365	23.619706	26.354645	28.017115	26.707076	29.213523	39.885364	30.397873	34.264866	69.278079	73.20484	71.247715	0.0990224	5.3272496	0.2955971	0.7583646	0.4155434	5.3272496	9.21E-06	6.65E-05	1.4475561	5.3272496	2.02E-53	4.80E-52
1102	ENSMUSG00	17113	M6pr	1	protein codi	MGI Symbol	3	mannose-6-p	576.87	562	602	577	616	559.82	1016	1012.68	810.81	1308.88	1818.97	1771.83	10.249606	9.7622905	10.701925	9.4849564	10.083392	9.8152191	13.807795	12.315889	11.801463	19.961672	24.577446	25.435123	-0.063748	3.8164159	0.5369435	0.8863533	0.304478	3.8164159	0.0023292	0.0078079	1.1884583	3.8164159	1.20E-33	1.57E-32
1103	ENSMUSG00	241296	Lrrc8a	1	protein codi	MGI Symbol	2	leucine rich	578.14	501	425.55	606.12	577.8	547.25	1085.31	1059.74	928.9	1383.87	1555.17	1573.71	10.272171	8.7026825	7.5651229	9.9636426	9.4580909	9.5948317	14.749742	12.888217	13.520281	21.105341	21.01305	22.591054	0.1287626	3.7598361	0.2145316	0.6914094	0.632557	3.7598361	2.93E-10	4.28E-09	1.2856451	3.7598361	2.31E-38	3.63E-37
11042	ENSMUSG00	380712	Tlcd2	1	protein codi	MGI Symbol	4	TLC domain	194	191	146	234	178	196	334.78	350.17	346.6	548.12	626.35	591.23	3.4469179	3.3177891	2.5954834	3.8465855	2.9137075	3.4364313	4.549777	4.258655	5.0448157	8.3593542	8.4630771	8.4872745	0.122762	2.3141479	0.3254179	0.7793548	0.5625601	2.3141479	1.81E-06	1.54E-05	1.4342253	2.3141479	1.89E-36	2.76E-35
11054	ENSMUSG00	320878	Mical2	1	protein codi	MGI Symbol	13	microtubule	399	256	325	402	489	382	809	900	771	2404	3023	2696	7.0892797	4.4468797	5.7776171	6.6082366	8.004511	6.6975344	10.994592	10.945511	11.222022	36.663299	40.845984	38.701846	0.3009798	3.9829908	0.0058316	0.0843229	0.9380725	3.9829908	2.46E-19	7.67E-18	2.7473864	3.9829908	3.96E-160	5.30E-158
11055	ENSMUSG00	100504195	Mical2	1	protein codi	MGI Symbol	13	microtubule	399	256	325	402	489	382	809	900	771	2404	3023	2696	7.0892797	4.4468797	5.7776171	6.6082366	8.004511	6.6975344	10.994592	10.945511	11.222022	36.663299	40.845984	38.701846	0.3009798	3.9829908	0.0058316	0.0843229	0.9380725	3.9829908	2.46E-19	7.67E-18	2.7473864	3.9829908	3.96E-160	5.30E-158
11086	ENSMUSG00	67198	Spats2l	1	protein codi	MGI Symbol	15	spermatoge	128	121	142	215	172	158	336	316	286	499	561	522	2.2742551	2.1018455	2.5243742	3.5342559	2.8154926	2.7701844	4.5663572	3.8430905	4.1627735	7.6102272	7.580085	7.4934582	0.4038163	2.1241357	0.0023013	0.0410173	0.864003	2.1241357	3.37E-12	6.13E-11	1.7165494	2.1241357	5.15E-47	1.03E-45
11114	ENSMUSG00	68728	Trp53inp2	1	protein codi	MGI Symbol	5	transformati	492	454	454	528	491	563	1221	1395	1010	2399	2848	2491	8.7416682	7.8862632	8.0708866	8.6794749	8.0372493	9.8709735	16.593816	16.965542	14.700704	36.587044	38.48143	35.759012	0.1054905	4.1431875	0.3132715	0.7701806	0.9670741	4.1431875	5.34E-22	1.94E-20	2.1657472	4.1431875	3.14E-105	1.90E-103
11118	ENSMUSG00	20130	Rras	1	protein codi	MGI Symbol	3	related RAS	557	629	616	750	751	672	1318	1362	1210	2377	2622	2672	9.8965633	10.926122	10.950807	12.3288	12.293227	11.78205	17.91208	16.564206	17.611734	36.251523	35.427777	38.357319	0.1963445	4.270999	0.0520676	0.3633439	0.7126141	4.270999	4.83E-13	9.61E-12	1.7917827	4.270999	6.62E-75	2.37E-73
11123	ENSMUSG00	65112	Pmepa1	1	protein codi	MGI Symbol	3	prostate trar	518	499	403	438	446	425	1555	2090	1284	2339	3399	3090	9.2036262	8.6679412	7.1642452	7.2000189	7.3006379	7.4514453	21.132993	25.417909	18.688815	35.671987	45.926397	44.357827	-0.189868	4.3235542	0.0730205	0.4276789	1.3827357	4.3235542	6.34E-44	5.28E-42	2.3308606	4.3235542	1.67E-121	1.33E-119
11132	ENSMUSG00	13653	Egr1	1	protein codi	MGI Symbol	2	early growth	225	158	257	98	213	82	110	75	135	1053	2373	1751	3.9977141	2.7445585	4.5687618	1.6109631	3.4866275	1.4376906	1.4949384	0.9121259	1.9649455	16.059257	32.063354	25.136102	-0.782794	3.016009	1.02E-09	6.82E-08	-1.388403	3.016009	2.49E-25	1.03E-23	2.6977375	3.016009	9.33E-136	9.63E-134
11139	ENSMUSG00	14794	Spsb2	1	protein codi	MGI Symbol	5	splA/ryanod	129	134	134	135	135	111	235	278	236	325	450	376	2.2920228	2.3276636	2.382156	2.2191839	2.2098343	1.9461422	3.193732	3.3809467	3.4350159	4.9565608	6.0802821	5.3975868	-0.133576	1.7633504	0.3428011	0.7893553	0.5151451	1.7633504	4.97E-05	0.0002975	1.2320909	1.7633504	3.96E-24	3.26E-23
11171	ENSMUSG00	75480	1700003F12	1	protein codi	MGI Symbol	3	RIKEN cDNA	435	372	480	568	512	455	754	729	680	1181	1310	1622	7.7289139	6.461872	8.533096	9.3370109	8.3810013	7.9774297	10.247123	8.8658638	9.8975034	18.011379	17.700377	23.284271	0.178253	3.5174087	0.0918534	0.4777935	0.3517615	3.5174087	0.0006425	0.0027329	1.3766064	3.5174087	9.52E-43	1.70E-41
1118	ENSMUSG00	12048	Bcl2l1	1	protein codi	MGI Symbol	9	BCL2-like 1 [	826	830	925	851	869	755	1746	1954	1504	2338	2759	2595	14.676053	14.417618	16.443987	13.989078	14.224785	13.237273	23.728749	23.76392	21.890949	35.656736	37.278885	37.251962	-0.135296	4.4804114	0.1701778	0.6268665	0.6078641	4.4804114	2.67E-10	3.92E-09	1.2748962	4.4804114	2.62E-40	4.36E-39
11206	ENSMUSG00	83397	Akap12	1	protein codi	MGI Symbol	5	A kinase (PR	363	255	317	305	375	340	1013	1026	941	1553	1898	2064	6.4496454	4.4295091	5.6353988	5.0137118	6.1384287	5.9611563	13.767023	12.477882	13.696398	23.684735	25.645279	29.629306	0.0516981	3.6841928	0.6515176	0.9285027	1.273981	3.6841928	1.22E-34	7.28E-33	2.2579151	3.6841928	3.13E-108	2.02E-106
11214	ENSMUSG00	75687	Ripor1	1	protein codi	MGI Symbol	1	RHO family i	379	288	277	335	373	350	544	675	538	945	1130	985	6.7339273	5.0027396	4.9243075	5.5068638	6.1056904	6.1364844	7.3931498	8.2091331	7.8306718	14.412154	15.268264	14.139955	0.0912453	3.0978067	0.416011	0.8392661	0.4928838	3.0978067	4.02E-06	3.17E-05	1.3954269	3.0978067	2.09E-41	3.60E-40
1124	ENSMUSG00	78070	Cpt1c	1	protein codi	MGI Symbol	7	carnitine pal	710.98	663.98	650.99	757.96	727	714	1159	1225	1004.99	1477	1819.99	1707	12.632421	11.533747	11.572834	12.459649	11.900367	12.518428	15.751214	14.898056	14.627782	22.525662	24.591228	24.50447	0.0452176	3.9884362	0.6558992	0.9286055	0.3413523	3.9884362	0.0005283	0.0023126	1.0030786	3.9884362	7.53E-25	6.49E-24
11262	ENSMUSG00	75007	Mindy1	1	protein codi	MGI Symbol	12	MINDY lysin	167	177	171	201	177	180	317	323	293	446	492	432	2.9671922	3.0746004	3.0399155	3.3041183	2.8973383	3.1559063	4.3081406	3.9282222	4.2646596	6.8019265	6.6477751	6.2014827	0.042778	2.0992629	0.7480226	0.9568223	0.4593168	2.0992629	0.0001198	0.0006438	1.1127955	2.0992629	8.32E-22	6.11E-21
1128	ENSMUSG00	328365	Zmiz1	1	protein codi	MGI Symbol	8	zinc finger, N	1641.99	1306.92	1777.92	1418.96	1721.97	1512.88	2216.99	2668.98	2376.94	4156.92	5157.89	4925.81	29.174251	22.702016	31.606588	23.325431	28.187174	26.525041	30.129668	32.459277	34.596723	63.397005	69.692058	70.711401	-0.097146	5.2718444	0.304652	0.7643114	0.2193011	5.2718444	0.0192406	0.0435828	1.287842	5.2718444	5.98E-43	1.08E-41
11292	ENSMUSG00	13849	Ephx1	1	protein codi	MGI Symbol	4	epoxide hyd	251	217	265	274	266	267	662	817	544	1115	1576	1332	4.4596722	3.7694253	4.7109801	4.5041214	4.3541921	4.6812609	8.996811	9.9360915	7.9180027	17.004816	21.294499	19.121238	0.065401	3.2271044	0.5823576	0.9044299	1.0553545	3.2271044	3.31E-22	1.21E-20	2.1505349	3.2271044	1.28E-89	5.58E-88
11294	ENSMUSG00	75788	Smurf1	1	protein codi	MGI Symbol	5	SMAD specif	374	337	374	350	335	391	520	554	522	777	1022	949	6.6450892	5.8539002	6.648704	5.7534398	5.483663	6.8553297	7.0669814	6.73757	7.5977894	11.849993	13.808996	13.623164	-0.082904	3.0408037	0.457288	0.8564485	0.1595945	3.0408037	0.1338978	0.204572	1.0374663	3.0408037	7.43E-24	6.05E-23
11309	ENSMUSG00	209462	Hace1	1	protein codi	MGI Symbol	7	HECT domai	478	506	481	526	543	465	952	1044	931	1190	1436	1363	8.4929215	8.7895356	8.5508733	8.6465981	8.8884448	8.1527578	12.938012	12.696793	13.550847	18.148638	19.402856	19.566252	-0.007811	3.6337005	0.9460797	0.9996602	0.6005129	3.6337005	2.50E-09	3.28E-08	1.1446882	3.6337005	1.65E-30	1.92E-29
11320	ENSMUSG00	320404	Itpkb	1	protein codi	MGI Symbol	2	inositol 1,4,5	376	360	380	283	313	303	1320	1672	1264	2803	3343	2993	6.6806245	6.2534245	6.7553677	4.652067	5.1235418	5.3124422	17.939261	20.334327	18.397712	42.748431	45.16974	42.965365	-0.384331	4.2254446	0.0005476	0.0119199	1.5256197	4.2254446	3.19E-51	3.19E-49	2.7326735	4.2254446	7.93E-161	1.12E-158
11336	ENSMUSG00	384783	Irs2	1	protein codi	MGI Symbol	1	insulin recep	725	676	743.97	691	783.86	756	1749.99	1851.99	1604.97	3013.98	3065.96	2863.99	12.881523	11.742542	13.225765	11.358934	12.831117	13.254806	23.782975	22.523307	23.360582	45.966077	41.426448	41.11339	-0.015722	4.5181879	0.8806193	0.9859489	0.8800756	4.5181879	1.21E-19	3.84E-18	1.7632944	4.5181879	6.92E-74	2.46E-72
11342	ENSMUSG00	224860	Plcl2	1	protein codi	MGI Symbol	2	phospholipa	130	125	169	200	214	167	220	233	172	367	383	341	2.3097904	2.171328	3.0043609	3.2876799	3.5029966	2.9279797	2.9898768	2.8336711	2.5034862	5.597101	5.1749956	4.8951518	0.378666	1.8006604	0.0035784	0.0579123	0.1563804	1.8006604	0.2335244	0.3194358	1.0650291	1.8006604	1.34E-18	7.93E-18
11361	ENSMUSG00	217124	Ppp1r9b	1	protein codi	MGI Symbol	3	protein phos	1415	1294	1262	1488	1473	1417	2236	2461	2144	3077	3462	3304	25.14118	22.477587	22.434932	24.460338	24.111748	24.843995	30.38802	29.929891	31.206246	46.927193	46.777637	47.429858	0.0676209	4.9739151	0.4788286	0.8651334	0.3855849	4.9739151	4.26E-05	0.0002577	1.0105193	4.9739151	6.64E-27	6.47E-26
1139	ENSMUSG00	13033	Ctsd	1	protein codi	MGI Symbol	3	cathepsin D	13023.97	12488.16	12593.61	15211.2	15080.59	14432.1	26625.1	27394.68	24158.58	37522.88	47317.5	45068.38	231.40493	216.92713	223.88017	250.04778	246.85634	253.0353	361.8444	333.1653	351.63181	572.2598	639.34166	646.96939	0.1578558	8.4947709	0.0800558	0.4500179	0.6387619	8.4947709	1.58E-12	2.98E-11	1.4672143	8.4947709	1.50E-57	3.92E-56
11413	ENSMUSG00	68810	Nexn	1	protein codi	MGI Symbol	16	nexilin [Sour	320	336	379	333	433	345	630	745	578	812.46	936	961	5.6856378	5.8365296	6.7375904	5.473987	7.087839	6.0488203	8.5619198	9.0604507	8.4128779	12.390792	12.646987	13.795428	0.0279477	3.0979423	0.8090813	0.9715852	0.5125242	3.0979423	1.16E-06	1.02E-05	1.0886321	3.0979423	6.27E-26	5.80E-25
11414	ENSMUSG00	66290	Atp6v1g1	1	protein codi	MGI Symbol	1	ATPase, H+ t	2616.63	2628.07	2974.62	2958.83	3174.98	3028.16	5528.32	5985.11	5062.61	6758.26	8026.15	7671.88	46.491283	45.651215	52.880663	48.638429	51.971702	53.092162	75.131798	72.788985	73.687059	103.06993	108.44724	110.13202	0.0838296	6.1347794	0.364275	0.8055715	0.611723	6.1347794	2.86E-11	4.70E-10	1.1492431	6.1347794	1.64E-35	2.32E-34
1142	ENSMUSG00	208982	Hmgcll1	1	protein codi	MGI Symbol	7	3-hydroxym	139.98	164.98	150	197	225	208.98	324	332	331.99	399.99	614.93	519	2.4871112	2.8658055	2.6665925	3.2383647	3.6830572	3.6640072	4.4032781	4.0376773	4.8321649	6.10023	8.308773	7.4503924	0.3992983	2.1885503	0.0016107	0.0301674	0.7234497	2.1885503	2.14E-09	2.86E-08	1.4478154	2.1885503	4.41E-35	6.14E-34
11423	ENSMUSG00	353211	Prune2	1	protein codi	MGI Symbol	9	prune homo	132	95	128	276	262	279	216	301	223	493	737	725	2.3453256	1.6502093	2.2754923	4.5369982	4.2887155	4.8916547	2.9355154	3.6606653	3.2457989	7.5187215	9.9581509	10.407581	1.1289066	2.289514	6.97E-19	1.09E-16	0.6538237	2.289514	4.25E-07	4.09E-06	2.1546232	2.289514	1.67E-72	5.80E-71
11428	ENSMUSG00	73750	Whrn	1	protein codi	MGI Symbol	17	whirlin [Sour	507	555	418	698	636	680	1514	1747	1394	2350	2912	2509	9.0081824	9.6406962	7.4309044	11.474003	10.410775	11.922313	20.575788	21.246453	20.289882	35.839747	39.346181	36.017407	0.3735939	4.2909426	0.0002624	0.0063749	1.2516486	4.2909426	1.95E-36	1.27E-34	2.0918	4.2909426	5.28E-99	2.77E-97
11437	ENSMUSG00	98952	Fam102a	1	protein codi	MGI Symbol	5	family with s	259	270	259	245	247	288	482	539	425	915	1052	915	4.6018131	4.6900684	4.6043164	4.0274078	4.0431784	5.04945	6.5505482	6.5551448	6.1859396	13.954625	14.214348	13.135085	-0.084912	2.8865513	0.4737508	0.864464	0.4736733	2.8865513	1.69E-05	0.0001143	1.5712099	2.8865513	5.62E-49	1.19E-47
11444	ENSMUSG00	223453	Dap	1	protein codi	MGI Symbol	5	death-associ	373	385	367	399	401	394	649	722	549	960	1540	1464	6.6273216	6.6876901	6.524263	6.5589214	6.5640264	6.9079281	8.8201365	8.780732	7.9907785	14.640918	20.808076	21.016136	0.0134854	3.3588432	0.9083026	0.9905825	0.3679653	3.3588432	0.0004556	0.0020292	1.5098015	3.3588432	4.24E-50	9.21E-49
11462	ENSMUSG00	54608	Abhd2	1	protein codi	MGI Symbol	2	abhydrolase	353	316.05	380	381	373.5	395	758.33	961.89	840.81	1967.22	2270	2104	6.2719692	5.4899856	6.7553677	6.2630302	6.113875	6.925461	10.305969	11.698197	12.238117	30.001986	30.671645	30.203517	0.0595166	3.7773102	0.5912525	0.9073765	0.8869079	3.7773102	1.16E-17	3.24E-16	2.2951278	3.7773102	1.10E-113	7.83E-112
11486	ENSMUSG00	246316	Lgi2	1	protein codi	MGI Symbol	2	leucine-rich	10708	9740	9956	11122	11413	10976	21075	23724	20804	34415	38057	38217	190.25566	169.18988	176.99063	182.82788	186.82103	192.44015	286.4166	288.52367	302.80538	524.86166	514.21621	548.61589	0.0673881	8.214898	0.4558511	0.8562065	0.7103851	8.214898	4.35E-15	9.98E-14	1.5654508	8.214898	7.77E-65	2.34E-63
1149	ENSMUSG00	68041	Mid1ip1	1	protein codi	MGI Symbol	5	Mid1 interac	528	487.11	464	575.91	545.11	608.38	741.53	815.51	638	1369.7	1717.22	1512.85	9.3813024	8.4614045	8.2486595	9.4670386	8.9229837	10.666612	10.077651	9.9179706	9.286187	20.889235	23.202627	21.717391	0.1545664	3.6552115	0.135285	0.5655029	0.1669484	3.6552115	0.1035671	0.166987	1.3349044	3.6552115	3.69E-41	6.32E-40
11507	ENSMUSG00	76438	Rftn1	1	protein codi	MGI Symbol	3	raftlin lipid r	261	185	257	218	196	201	595	685	597	921	1074	923	4.6373484	3.2135654	4.5687618	3.5835711	3.2083521	3.5240953	8.0862576	8.3307499	8.6894258	14.046131	14.511607	13.249927	-0.266961	2.924232	0.0269696	0.2461199	1.0158046	2.924232	2.18E-20	7.26E-19	1.751841	2.924232	5.56E-59	1.50E-57
11510	ENSMUSG00	16008	Igfbp2	1	protein codi	MGI Symbol	3	insulin-like g	7	20	15	13	23	8	684	737	487	10055	9362	7666	0.1243733	0.3474125	0.2666593	0.2136992	0.3764903	0.1402625	9.2957987	8.9631572	7.0883591	153.34837	126.49689	110.04761	-0.006801	5.126958	1	1	5.0881915	5.126958	3.59E-191	2.43E-188	9.0286479	5.126958	0	0
11525	ENSMUSG00	233489	Picalm	1	protein codi	MGI Symbol	15	phosphatidy	2994	2755	3265	3369	3463	3274	5505	6349	5172	8984	10588	10468	53.196249	47.856068	58.04283	55.380968	56.686343	57.402428	74.814871	77.214498	75.279247	137.01459	143.06228	150.27111	0.0911687	6.3627048	0.3225619	0.777949	0.5148416	6.3627048	2.00E-08	2.38E-07	1.4356604	6.3627048	4.32E-54	1.05E-52
11555	ENSMUSG00	70546	Zdhhc2	1	protein codi	MGI Symbol	6	zinc finger, D	519	347	551.49	110.34	127.2	106.45	1134.64	746.66	923.21	1741.03	2134.57	1234.87	9.2213939	6.0276064	9.803994	1.813813	2.082155	1.8663679	15.420154	9.080639	13.437462	26.552372	28.84175	17.72691	-2.117567	3.5794832	2.92E-67	1.54E-64	0.596775	3.5794832	3.39E-09	4.40E-08	1.5461339	3.5794832	1.72E-54	4.21E-53
11557	ENSMUSG00	22393	Wfs1	1	protein codi	MGI Symbol	3	wolframin E	1253	1171	1200	1363	1442	1477	2403	2415	1947	2667	3461	3364	22.262826	20.341	21.33274	22.405538	23.604305	25.895964	32.657608	29.370454	28.338881	40.6743	46.764125	48.291175	0.1692377	4.918917	0.0762877	0.4386019	0.4992316	4.918917	1.28E-07	1.35E-06	1.08622	4.918917	1.32E-30	1.54E-29
11558	ENSMUSG00	20204	Prrx2	1	protein codi	MGI Symbol	4	paired relate	142	125	120	168	138	122	517	526	437	679	881	754	2.5230018	2.171328	2.133274	2.7616511	2.2589418	2.1390031	7.0262104	6.397043	6.3606015	10.355399	11.903841	10.823884	0.0704099	2.5119634	0.6181682	0.9160629	1.5342637	2.5119634	1.63E-37	1.13E-35	2.2766755	2.5119634	7.97E-84	3.25E-82
11568	ENSMUSG00	212898	Dse	1	protein codi	MGI Symbol	5	dermatan su	356	313	367	354	402	383	627	662	553	839	1003	949	6.3252721	5.4370052	6.524263	5.8191934	6.5803956	6.7150672	8.5211488	8.0510313	8.0489991	12.795552	13.552273	13.623164	0.0637677	3.100718	0.5660855	0.8974987	0.4293244	3.100718	4.94E-05	0.0002957	1.1285763	3.100718	8.32E-28	8.50E-27
1159	ENSMUSG00	67260	Cers4	1	protein codi	MGI Symbol	9	ceramide syr	561	510.25	543	593	596	532	1222.88	1380	1146	1855	2339	2073.93	9.9676338	8.8633608	9.6530649	9.7479708	9.7560094	9.3274563	16.619366	16.783117	16.680204	28.290524	31.603955	29.771854	0.0178804	4.0481555	0.8698548	0.9837644	0.8142462	4.0481555	2.44E-16	6.24E-15	1.6546305	4.0481555	2.46E-63	7.28E-62
11599	ENSMUSG00	53901	Rcan2	1	protein codi	MGI Symbol	7	regulator of	3493	3335	3809	3735	4033	3642	5078	5572	4901	8884	11751	12754	62.06229	57.93103	67.713672	61.397422	66.016755	63.854503	69.011792	67.764874	71.3348	135.4895	158.77643	183.08729	0.0271566	6.4724296	0.7683006	0.961343	0.148838	6.4724296	0.1037399	0.1670671	1.3466529	6.4724296	3.59E-48	7.39E-47
1162	ENSMUSG00	21885	Tle1	1	protein codi	MGI Symbol	10	transducin-li	299	335	297	333	343	319	559	606	533	933.23	1202	1151	5.3125178	5.819159	5.2798532	5.473987	5.6146161	5.5929672	7.5970051	7.3699773	7.7578961	14.23265	16.241109	16.522932	0.02323	3.1147478	0.8440068	0.9800758	0.4686501	3.1147478	1.21E-05	8.52E-05	1.5177747	3.1147478	7.81E-49	1.65E-47
1170	ENSMUSG00	13713	Elk3	1	protein codi	MGI Symbol	4	ELK3, memb	111.99	109.85	126.8	113.96	120.9	87	655.36	682.79	553.68	1715.32	2248.22	2032.42	1.9897956	1.908163	2.2541595	1.87332	1.9790294	1.5253547	8.9065711	8.3038726	8.0588966	26.16027	30.37736	29.175966	-0.19044	3.3775533	0.1952098	0.6632667	2.0378459	3.3775533	1.25E-64	1.74E-62	3.7998905	3.3775533	1.89E-229	5.12E-227
11729	ENSMUSG00	13138	Dag1	1	protein codi	MGI Symbol	7	dystroglycar	6377	5627	6791	6298	6885	6375	9598	11239	9453	15192	17659	18690	113.3041	97.7445	120.72553	103.52904	112.70155	111.77168	130.44017	136.68511	137.58985	231.69253	238.60378	268.30026	-0.016477	7.2321641	0.8565469	0.98209	0.2867351	7.2321641	0.001564	0.005656	1.1546143	7.2321641	1.61E-36	2.36E-35
11746	ENSMUSG00	68770	Phtf2	1	protein codi	MGI Symbol	7	putative hon	169	157	169	188	189	188	370	374	288	409	500	543	3.0027275	2.7271879	3.0043609	3.0904191	3.0937681	3.2961688	5.0284291	4.5484678	4.1918838	6.2376411	6.755869	7.7949192	0.1178662	2.1626907	0.363888	0.8050619	0.6574672	2.1626907	3.72E-08	4.27E-07	1.2513961	2.1626907	4.74E-27	4.66E-26
11784	ENSMUSG00	171543	Bmf	1	protein codi	MGI Symbol	8	BCL2 modify	508	414	461	417	432	389	1008	1213	1124	1492	1927	1846	9.0259501	7.1914382	8.1953276	6.8548125	7.0714699	6.8202641	13.699072	14.752116	16.359991	22.754427	26.037119	26.499854	-0.23421	3.7966985	0.027477	0.2485464	0.8758932	3.7966985	3.12E-18	9.19E-17	1.6253063	3.7966985	6.78E-60	1.86E-58
11811	ENSMUSG00	76281	Tax1bp3	1	protein codi	MGI Symbol	4	Tax1 (humar	444.65	418.16	465.63	441.05	451.43	450.67	709.6	783.35	692.46	1058.27	1368.16	1242.97	7.9003714	7.2637	8.2776364	7.250156	7.3895223	7.9015126	9.6437116	9.526851	10.078861	16.13963	18.486219	17.843187	-0.056737	3.4228052	0.5986919	0.909608	0.3190306	3.4228052	0.0019262	0.0066834	1.1628927	3.4228052	6.28E-31	7.45E-30
11870	ENSMUSG00	229731	Slc25a24	1	protein codi	MGI Symbol	2	solute carrie	429	379	475	500	531	504	694	843	688	1235	1561	1540	7.6223082	6.5834664	8.4442096	8.2191997	8.692015	8.8365375	9.4317022	10.252295	10.013945	18.834931	21.091823	22.107137	0.1850691	3.5566735	0.0794047	0.4480404	0.3914528	3.5566735	0.0001463	0.0007643	1.4540611	3.5566735	1.44E-47	2.90E-46
11920	ENSMUSG00	208449	Sgms1	1	protein codi	MGI Symbol	15	sphingomyel	175	140	169	139	189	153	452	545	398	831	967	1005	3.1093332	2.4318873	3.0043609	2.2849375	3.0937681	2.6825203	6.1428377	6.6281149	5.7929505	12.673545	13.065851	14.42706	-0.083098	2.6789958	0.5319077	0.8841481	1.1211589	2.6789958	9.13E-22	3.26E-20	2.2329225	2.6789958	1.31E-86	5.47E-85
11934	ENSMUSG00	104418	Dgkz	1	protein codi	MGI Symbol	14	diacylglycerc	897.54	780	758.37	874.07	821.74	824.44	1124	1241.47	964.71	2031.44	2458.12	2183.72	15.947148	13.549087	13.481758	14.368312	13.45118	14.454752	15.275552	15.098359	14.041501	30.981403	33.213473	31.347921	-0.023868	4.2360112	0.813331	0.97293	0.0479339	4.2360112	0.6285334	0.7034716	1.1526867	4.2360112	4.64E-33	5.92E-32
11947	ENSMUSG00	52118	Pvr	1	protein codi	MGI Symbol	5	poliovirus re	148	157	91	153	127	145	275	292	238	302	432	399	2.6296075	2.7271879	1.6177328	2.5150751	2.0788812	2.5422578	3.737346	3.5512102	3.4641262	4.6057888	5.8370708	5.7277583	0.0306127	1.8057505	0.834784	0.9788296	0.6232507	1.8057505	7.35E-07	6.80E-06	1.213498	1.8057505	2.07E-23	1.66E-22
1198	ENSMUSG00	15184	Hdac5	1	protein codi	MGI Symbol	20	histone deac	523	484	439	545	503	515	896	816	863	1614	1692	1583	9.2924643	8.4073819	7.8042274	8.9589277	8.233679	9.0293985	12.176953	9.9239298	12.561096	24.615043	22.861861	22.724415	0.0397806	3.7158319	0.7077678	0.9446879	0.4408435	3.7158319	1.33E-05	9.29E-05	1.4603715	3.7158319	6.80E-49	1.44E-47
11999	ENSMUSG00	114255	Dok4	1	protein codi	MGI Symbol	4	docking prot	127.77	126.34	117.48	132.17	125.53	133.59	255.5	255.33	233.87	377.64	441.11	384.67	2.2701686	2.1946046	2.0884752	2.1726632	2.0548185	2.3422084	3.4723342	3.1052414	3.4040134	5.7593711	5.9601627	5.5220471	0.0022783	1.7816041	1	1	0.6048246	1.7816041	2.25E-06	1.88E-05	1.3950674	1.7816041	4.14E-30	4.72E-29
12006	ENSMUSG00	18247	Oaz2	1	protein codi	MGI Symbol	7	ornithine de	2024.83	1937	2130	2165	2172.92	2154	3526	4057.91	3368.94	4611.98	5671.82	5251.97	35.976406	33.646898	37.865614	35.589135	35.568839	37.765678	47.91957	49.350998	49.035434	70.337105	76.636146	75.393521	0.0190851	5.6102655	0.8395175	0.978988	0.444853	5.6102655	1.58E-06	1.36E-05	1.0488458	5.6102655	1.44E-29	1.60E-28
12007	ENSMUSG00	76142	Ppp1r14c	1	protein codi	MGI Symbol	2	protein phos	11	16	19	13	15	9	435	457	280	628	946	866	0.1954438	0.27793	0.3377684	0.2136992	0.2455371	0.1577953	5.9118018	5.5578872	4.0754426	9.5776006	12.782104	12.431676	-0.384311	2.1689284	0.2908075	0.7545748	4.2539537	2.1689284	1.71E-126	6.77E-124	5.4135718	2.1689284	2.71E-233	7.79E-231
12020	ENSMUSG00	228355	Madd	1	protein codi	MGI Symbol	28	MAP-kinase	390	288	309	391	393	358	1083	1039	880	1304	1526	1550	6.9293711	5.0027396	5.4931806	6.4274142	6.4330733	6.2767469	14.718348	12.635984	12.808534	19.887247	20.618912	22.25069	0.1358267	3.5550129	0.2197884	0.6985581	1.2047137	3.5550129	2.11E-31	1.10E-29	1.8488617	3.5550129	2.80E-73	9.89E-72
12022	ENSMUSG00	107581	Col16a1	1	protein codi	MGI Symbol	13	collagen, typ	87	102	102	139	132	133	392	431	268	1410	2178	1934	1.5457828	1.7718036	1.8132829	2.2849375	2.1607269	2.3318641	5.3274168	5.2416835	3.9007808	21.503848	29.428565	27.763119	0.4002669	3.1580179	0.0055905	0.0819588	1.4980552	3.1580179	1.46E-31	7.61E-30	3.9379722	3.1580179	1.36E-234	4.03E-232
12026	ENSMUSG00	67803	Limd2	1	protein codi	MGI Symbol	4	LIM domain	496	429	495	451	426	464	977	953	813	1612	1928	1728	8.8127386	7.4519976	8.7997553	7.4137181	6.973255	8.135225	13.277771	11.59008	11.833339	24.584542	26.050631	24.805931	-0.154705	3.7461966	0.1441966	0.5824137	0.5501375	3.7461966	5.53E-08	6.15E-07	1.5899468	3.7461966	1.39E-57	3.64E-56
12032	ENSMUSG00	268396	Sh3pxd2b	1	protein codi	MGI Symbol	3	SH3 and PX d	699	607	776	592	701	649	1258	1402	1213	3042	4354	3801	12.419565	10.543969	13.795172	9.7315324	11.474769	11.378795	17.096659	17.050674	17.655399	46.393409	58.830107	54.564434	-0.173823	4.5574549	0.0849077	0.4633184	0.4950174	4.5574549	4.26E-07	4.09E-06	2.1204333	4.5574549	5.87E-105	3.44E-103
12088	ENSMUSG00	103554	Psme4	1	protein codi	MGI Symbol	7	proteasome	7557	7180	7891	8565	9636	8569	12555	14822	13478	21279	21060	20916	134.26989	124.72108	140.28054	140.79489	157.73306	150.23867	170.62683	180.2604	196.17434	324.5251	284.5572	300.25512	0.1686077	7.5859119	0.0626886	0.3957086	0.4543028	7.5859119	5.24E-07	4.95E-06	1.1874179	7.5859119	1.40E-38	2.23E-37
12092	ENSMUSG00	13875	Erf	1	protein codi	MGI Symbol	2	Ets2 repress	782	670	678	736	723	720	1257	1445	1171	2066	2219	2136	13.894277	11.638318	12.052998	12.098662	11.834891	12.623625	17.083069	17.573626	17.044083	31.508476	29.982547	30.662887	-0.040096	4.1907513	0.6930151	0.9421356	0.460261	4.1907513	2.53E-06	2.07E-05	1.2937713	4.1907513	1.21E-40	2.04E-39

Table S2. Differentially expressed genes (> 2-fold) in *Trpm3*-mutant lenses summarized in Fig. 9A.

12100	ENSMUSG00	235043	Tmem205	1	protein codi	MGI Symbol	7	transmembr	396	405	349	392	427	408	708	656	610	856	1125	962	7.0359768	7.0351026	6.2042719	6.4438526	6.9896242	7.1533875	9.621967	7.9780612	8.8786428	13.054819	15.200705	13.809783	0.021464	3.2008081	0.8485626	0.9812274	0.3837793	3.2008081	0.0002343	0.001147	1.0532092	3.2008081	7.28E-25	6.29E-24
12116	ENSMUSG00	16801	Arhgef1	1	protein codi	MGI Symbol	19	Rho guanine	409	354	314	385	404	432	470	542	475	951	1301	1111	7.2669559	6.1492008	5.582067	6.3287838	6.6131339	7.574175	6.387464	6.5916299	6.9136972	14.503659	17.578771	15.94872	0.1099294	3.1742973	0.3121099	0.7697482	0.0660006	3.1742973	0.5417966	0.6260117	1.3386759	3.1742973	3.56E-39	5.75E-38
12121	ENSMUSG00	63955	Cables1	1	protein codi	MGI Symbol	3	CDK5 and Ab	112.95	132.74	108.94	369.01	275.27	344.76	348.92	337.4	247.48	282.28	391.21	297.04	2.0068525	2.3057766	1.9366572	6.0659338	4.5059341	6.0446125	4.7419445	4.1033504	3.602109	4.3050399	5.285927	4.2640936	1.4081171	2.0527925	1.80E-29	5.03E-27	0.9940944	2.0527925	3.03E-15	7.11E-14	1.149348	2.0527925	4.67E-20	3.05E-19
12123	ENSMUSG00	72754	Arhgef10l	1	protein codi	MGI Symbol	12	Rho guanine	595	571	614	610	634	608	931	876	833	1346	1827	1396	10.571733	9.9186262	10.915252	10.027424	10.378037	10.65995	12.652615	10.653631	12.124442	20.527787	24.685945	20.039976	-0.015779	3.7730159	0.8829221	0.9859489	0.1730942	3.7730159	0.0836347	0.1407243	1.0555919	3.7730159	6.77E-27	6.59E-26
12139	ENSMUSG00	66092	Ghitm	1	protein codi	MGI Symbol	4	growth horn	3811	3811	3821	3557	3595	3468	6986	7626	6511	8391	9975	9818	67.712393	66.199447	67.927	58.471387	58.84707	60.803794	94.942178	92.744962	94.768595	127.97077	134.77959	140.94018	-0.180358	6.4747962	0.049355	0.3541817	0.4847931	6.4747962	1.10E-07	1.17E-06	1.0000598	6.4747962	1.14E-27	1.16E-26
12152	ENSMUSG00	14369	Fzd7	1	protein codi	MGI Symbol	1	frizzled class	406	338	290	427	387	464	652	676	484	1026	1078	898	7.213653	5.8712708	5.1554122	7.0191965	6.3348584	8.135225	8.8609075	8.2212948	7.0446936	15.647481	14.565654	12.891045	0.2352323	3.1682061	0.0297698	0.2635722	0.4047096	3.1682061	0.0001351	0.0007144	1.2402037	3.1682061	1.92E-33	2.49E-32
12171	ENSMUSG00	77056	Tmco4	1	protein codi	MGI Symbol	4	transmembr	236	213	322	314	332	306	604	545	506	812	892	703	4.1931579	3.6999429	5.7242852	5.1616574	5.4345555	5.3650406	8.2085708	6.6281149	7.364907	12.383777	12.05247	10.091765	0.2297816	2.8635226	0.045662	0.3403169	0.7045511	2.8635226	1.24E-10	1.88E-09	1.3426104	2.8635226	1.06E-35	1.51E-34
12179	ENSMUSG00	226098	Hectd2	1	protein codi	MGI Symbol	9	HECT domai	161.95	134.58	164.35	204	222.35	178.31	326.04	337.28	313.69	440.46	494.69	447.38	2.8774658	2.3377385	2.9216965	3.3534335	3.639679	3.1262758	4.4309974	4.101891	4.5658057	6.7174362	6.6841216	6.4222669	0.3162737	2.1173421	0.0129709	0.1504369	0.6856554	2.1173421	1.37E-08	1.68E-07	1.2848262	2.1173421	8.24E-28	8.44E-27
12256	ENSMUSG00	98682	Mfsd6	1	protein codi	MGI Symbol	6	major facilit	126	151	143	170	159	165	264	289	218	422	531	479	2.2387199	2.6229642	2.5421515	2.7945279	2.6026938	2.8929141	3.5878521	3.5147252	3.1730232	6.4359036	7.1747329	6.876181	0.1618122	1.9810727	0.2273758	0.7080256	0.4737394	1.9810727	0.0001606	0.0008279	1.4680806	1.9810727	4.48E-35	6.22E-34
12272	ENSMUSG00	20908	Stx3	1	protein codi	MGI Symbol	8	syntaxin 3 [S	442	413	428	472	446	433	1104	998	858	1428	1616	1599	7.8532873	7.1740676	7.6086773	7.7589245	7.3006379	7.5917078	15.003745	12.137355	12.488321	21.778366	21.834969	22.954099	0.0010591	3.6707646	1	1	0.8075985	3.6707646	1.82E-15	4.32E-14	1.5561102	3.6707646	2.25E-54	5.50E-53
12297	ENSMUSG00	20364	Selenow	1	protein codi	MGI Symbol	1	selenoprotei	11990	12446	12646	14292	14161	13291	21353	21597	18399	26437	33606	31819	213.03374	216.19478	224.81153	234.9376	231.80344	233.02861	290.19472	262.65578	267.80024	403.18953	454.07547	456.77078	0.0975018	8.1838503	0.2797905	0.7485243	0.3273771	8.1838503	0.0002831	0.0013522	1.0065677	8.1838503	1.62E-28	1.71E-27
12319	ENSMUSG00	16565	Kif21b	1	protein codi	MGI Symbol	6	kinesin famil	147	113	132	165	128	156	277	414	365	369	624	621	2.6118399	1.9628805	2.3466014	2.7123359	2.0952503	2.7351188	3.7645267	5.034935	5.3126305	5.6276029	8.4313245	8.9146313	0.1232108	2.1369887	0.3693224	0.8079609	1.0279242	2.1369887	5.93E-17	1.56E-15	1.7335383	2.1369887	5.17E-48	1.06E-46
12359	ENSMUSG00	12257	Tspo	1	protein codi	MGI Symbol	2	translocator	160	133	151	176	161	155	344	319	288	436	721	580	2.8428189	2.310293	2.6843698	2.8931583	2.6354321	2.7175859	4.67508	3.8795755	4.1918838	6.6494169	9.7419631	8.3260647	0.0740942	2.1878024	0.585729	0.9062583	0.7004597	2.1878024	9.26E-09	1.16E-07	1.6595288	2.1878024	6.11E-46	1.20E-44
1238	ENSMUSG00	240638	Slc16a12	1	protein codi	MGI Symbol	2	solute carrie	1871	1681	1843	1911	1842	1878	4142	4608	3885	7730	8284	7952	33.243214	29.200018	32.763533	31.413781	30.151962	32.926622	56.291225	56.041016	56.546766	117.88989	111.93124	114.15322	-0.010916	5.8748133	0.9096661	0.9905825	0.8268777	5.8748133	6.09E-19	1.87E-17	1.8531445	5.8748133	6.34E-86	2.64E-84
12384	ENSMUSG00	27280	Phlda3	1	protein codi	MGI Symbol	2	pleckstrin hc	97	95	100	99	105	109	387	339	259	1187	1763	1478	1.723459	1.6502093	1.7777283	1.6274015	1.71876	1.9110766	5.259465	4.1228091	3.7697844	18.102885	23.821194	21.21711	0.0278145	2.8843772	0.8971858	0.9891116	1.3520941	2.8843772	1.02E-25	4.32E-24	3.615133	2.8843772	3.92E-198	8.37E-196
1239	ENSMUSG00	20965	Syn2	1	protein codi	MGI Symbol	7	synapsin II [S	1321.45	977.07	1763.43	1126.47	1645.49	1139.71	1915.64	2457	2288.62	3634.8	4458.61	3839.6	23.479019	16.972315	31.348995	18.517364	26.935261	19.982322	26.034216	29.881245	33.311212	55.434176	60.24357	55.118548	-0.133188	5.0531353	0.1620567	0.614973	0.3135469	5.0531353	0.000871	0.0035121	1.2506126	5.0531353	2.89E-40	4.79E-39
12392	ENSMUSG00	19267	Ptpre	1	protein codi	MGI Symbol	8	protein tyros	262	219	228	218	278	236	365	400	375	761	1000	989	4.655116	3.8041666	4.0532206	3.5835711	4.5506218	4.1377438	4.9604774	4.8646715	5.458182	11.605978	13.511738	14.197376	-0.027647	2.7451105	0.8245744	0.975947	0.2873772	2.7451105	0.0110437	0.0278143	1.6523287	2.7451105	1.18E-52	2.73E-51
12393	ENSMUSG00	50996	Pdcd7	1	protein codi	MGI Symbol	3	programmed	347	353	384	389	443	403	633	638	524	962	1122	923	6.1653635	6.1318302	6.8264768	6.3945374	7.2515304	7.0657235	8.6026909	7.759151	7.6268997	14.67142	15.16017	13.249927	0.1150094	3.1674469	0.2913055	0.7545748	0.3270542	3.1674469	0.0019626	0.0067833	1.1717396	3.1674469	3.29E-30	3.77E-29
12398	ENSMUSG00	11854	Rhod	1	protein codi	MGI Symbol	4	ras homolog	277.79	297	271	265	255	253	519	528.86	463	759.5	873.9	780	4.9356667	5.1590752	4.8176438	4.3561758	4.1741315	4.4358016	7.0533911	6.4318254	6.7390354	11.583101	11.807908	11.197122	-0.202187	2.8023483	0.0820397	0.4558759	0.438659	2.8023483	5.62E-05	0.000332	1.213378	2.8023483	1.30E-30	1.52E-29
12403	ENSMUSG00	17215	Mcm3	1	protein codi	MGI Symbol	5	minichromo	345	304	322	284	321	310	539	470	454	739	844	858	6.1298283	5.2806696	5.7242852	4.6685054	5.254495	5.4351719	7.3251981	5.715989	6.6080391	11.270457	11.403907	12.316834	-0.158248	2.8738237	0.1621444	0.614973	0.1959454	2.8738237	0.0692437	0.1209441	1.0301586	2.8738237	5.68E-23	4.45E-22
1241	ENSMUSG00	215690	Nav1	1	protein codi	MGI Symbol	9	neuron navig	713	518	601.92	686	903	858	1120.6	1627.88	1302.67	2907.88	3813.61	3653.72	12.668312	8.9979831	10.700502	11.276742	14.781336	15.043153	15.229345	19.797754	18.96056	44.347951	51.528499	52.450188	0.3444946	4.5305544	0.0005862	0.0125994	0.7387912	4.5305544	6.11E-14	1.29E-12	2.1965854	4.5305544	4.52E-111	3.02E-109
12425	ENSMUSG00	11603	Agrn	1	protein codi	MGI Symbol	7	agrin [Sourc	1053	716	947	946	895	866	1501	1606	1374	2665	3265	3286	18.709302	12.437367	16.835087	15.550726	14.650383	15.183416	20.399114	19.531656	19.998779	40.643799	44.115824	47.171463	-0.079915	4.5770779	0.4158316	0.8392661	0.3210121	4.5770779	0.0008655	0.0034988	1.4596275	4.5770779	1.83E-52	4.20E-51
12432	ENSMUSG00	20194	S100a10	1	protein codi	MGI Symbol	3	S100 calcium	485	564	556	574	580	572	1663	1871	1430	2642	3511	3596	8.6172948	9.7970318	9.8841695	9.4356412	9.4941031	10.028769	22.60075	22.754501	20.813868	40.293027	47.439712	51.621601	0.032867	4.4631321	0.7537371	0.958233	1.2255063	4.4631321	2.14E-35	1.32E-33	2.2998522	4.4631321	7.46E-120	5.81E-118
12437	ENSMUSG00	213498	Arhgef11	1	protein codi	MGI Symbol	6	Rho guanine	907.88	839.74	855.29	920.2	1001.66	991.17	1270	1490.39	1186.57	2217.72	2654.19	2476.06	16.130865	14.586808	15.204733	15.126615	16.396316	17.377998	17.259743	18.125644	17.270707	33.822351	35.86272	35.544544	0.0904253	4.4018513	0.3567342	0.8002987	0.1977251	4.4018513	0.0415686	0.0801505	1.1963628	4.4018513	9.97E-36	1.42E-34
12447	ENSMUSG00	76044	Ncapg2	1	protein codi	MGI Symbol	6	non-SMC cor	152	125	140	137	143	137	249	272	208	326	387	390	2.700678	2.171328	2.4888197	2.2520607	2.3407875	2.4019953	3.3839969	3.3079766	3.0274716	4.9718118	5.2290426	5.5985608	-0.073328	1.7615521	0.6064597	0.912186	0.4028013	1.7615521	0.0014357	0.0052884	1.1024674	1.7615521	8.87E-20	5.68E-19
12490	ENSMUSG00	233912	Armc5	1	protein codi	MGI Symbol	2	armadillo re	1322.17	1152.94	1431.39	1314.31	1377.53	1225.24	2191.01	2542.84	2168.73	3660.74	4060.79	4108.67	23.491812	20.027287	25.446226	21.605153	22.548986	21.481903	29.77659	30.925203	31.566195	55.829786	54.86833	58.981125	-0.071136	5.0507135	0.4571462	0.8564485	0.4200521	5.0507135	8.35E-06	6.09E-05	1.2989408	5.0507135	3.65E-43	6.65E-42
12506	ENSMUSG00	107035	Fbxo38	1	protein codi	MGI Symbol	4	F-box protei	715	622	654	810	832	777	1175	1277	1164	1453	1757	1732	12.703847	10.804528	11.626343	13.315104	13.619127	13.622995	15.96866	15.530464	16.942197	22.15964	23.740124	24.863352	0.2071596	4.0285337	0.0382886	0.3108739	0.4629899	4.0285337	2.47E-06	2.03E-05	1.0103402	4.0285337	3.92E-25	3.45E-24
12509	ENSMUSG00	213539	Bag2	1	protein codi	MGI Symbol	4	BCL2-associa	290	266	271	260	272	239	507.84	528.96	443	803.98	998.9	912.59	5.1526093	4.6205859	4.8176438	4.2739838	4.452407	4.1903422	6.9017228	6.4330416	6.4479324	12.261464	13.496875	13.100489	-0.175169	2.8621915	0.1318829	0.5585707	0.4390984	2.8621915	5.96E-05	0.0003501	1.4134691	2.8621915	3.04E-41	5.22E-40
12512	ENSMUSG00	56068	Ammecr1	1	protein codi	MGI Symbol	1	Alport syndr	396	336	380	407	417	423	589	664	542	1000	1071	1282	7.0359768	5.8365296	6.7553677	6.6904285	6.8259327	7.4163797	8.0047155	8.0753547	7.8888924	15.250956	14.471071	18.403474	0.0926147	3.2426994	0.3948658	0.8260973	0.2887846	3.2426994	0.0062268	0.0175639	1.2938374	3.2426994	7.23E-37	1.08E-35
12531	ENSMUSG00	228071	Sestd1	1	protein codi	MGI Symbol	4	SEC14 and sp	162	137	141.98	148	178	126.98	261	284	263	460	565.99	514	2.8783542	2.3797755	2.5240187	2.4328831	2.9137075	2.2263165	3.5470811	3.4539168	3.828005	7.0154399	7.6475086	7.378616	-0.03669	2.0354555	0.8062402	0.9710044	0.4751623	2.0354555	0.000119	0.0006402	1.502389	2.0354555	2.01E-37	3.06E-36
12536	ENSMUSG00	192187	Stab1	1	protein codi	MGI Symbol	13	stabilin 1 [Sc	2246.64	1865.52	1719.7	2316.41	2224.87	2350.83	3147.65	3523.7	2974.68	5064.12	6114.36	5274.08	39.917442	32.405246	30.571594	38.078073	36.419216	41.216662	42.777662	42.854107	43.29692	77.232673	82.61563	75.710916	0.1692784	5.6051729	0.0696073	0.4172048	0.3254202	5.6051729	0.0004561	0.0020302	1.194987	5.6051729	1.08E-37	1.66E-36
12542	ENSMUSG00	105835	Sgsm3	1	protein codi	MGI Symbol	15	small G prot	159	152	152	178	198	132	251	283	252	383	541	443	2.8250513	2.6403348	2.7021471	2.9260351	3.2410904	2.3143313	3.4111776	3.4417551	3.6678983	5.8411163	7.3098502	6.3593908	0.0575296	1.9864142	0.677594	0.9366622	0.3642237	1.9864142	0.0033119	0.0104772	1.2576422	1.9864142	1.16E-26	1.13E-25
12554	ENSMUSG00	218639	Arl15	1	protein codi	MGI Symbol	3	ADP-ribosyla	596	596	690	637	743	720	1402	1790	1286	2043	2483	2360	10.5895	10.352892	12.266326	10.47126	12.162273	12.623625	19.053669	21.769405	18.717926	31.157704	33.549645	33.87847	0.0860512	4.2481791	0.3955672	0.8264544	0.8430968	4.2481791	8.08E-18	2.30E-16	1.5699556	4.2481791	3.07E-58	8.15E-57
12615	ENSMUSG00	76850	Ago4	1	protein codi	MGI Symbol	2	argonaute R	209	208	167	226	235	231	324	387	296	414	577	527	3.7134322	3.6130897	2.9688063	3.7150783	3.8467487	4.0500797	4.4032731	4.7065697	4.308325	6.3138959	7.7962728	7.5652347	0.1726045	2.2687656	0.1590432	0.6096476	0.3830838	2.2687656	0.001117	0.0043083	1.0748767	2.2687656	2.75E-21	1.95E-20
12627	ENSMUSG00	223697	Sun2	1	protein codi	MGI Symbol	10	Sad1 and UN	186	145	158	192	168	174	257	322	268	405	560	487	3.304777	2.5187404	2.8088108	3.1561727	2.7500161	3.0507094	3.4927197	3.9160606	3.9007808	6.1766373	7.5665733	6.9910233	0.0534925	2.0736431	0.6896791	0.9405615	0.3905289	2.0736431	0.0013686	0.0050929	1.2658547	2.0736431	1.40E-27	1.42E-26
12648	ENSMUSG00	16923	Sh2b3	1	protein codi	MGI Symbol	8	SH2B adapto	175	165	187	186	177	191	301	330	299	454	531	446	3.1093332	2.8661529	3.324352	3.0575423	2.8973383	3.3487672	4.090695	4.013354	4.3519905	6.9239342	7.1747329	6.4024567	-0.000254	2.1264742	1	1	0.4205511	2.1264742	0.0004208	0.0019034	1.1406164	2.1264742	4.78E-23	3.75E-22
1268	ENSMUSG00	110279	Bcr	1	protein codi	MGI Symbol	5	BCR activato	1747	1504	1635	1733	1694	1603	2516	2945	2444	4013	4854	4481	31.040029	26.125418	29.065858	28.487746	27.729329	28.105099	34.193318	35.816144	35.572792	61.202088	65.585976	64.326027	-0.032205	5.2864024	0.7342525	0.9514929	0.2921946	5.2864024	0.0017748	0.0062818	1.1482665	5.2864024	1.19E-34	1.62E-33
12685	ENSMUSG00	50772	Mapk6	1	protein codi	MGI Symbol	7	mitogen-acti	810	708	811.49	740	824	774	1469	1648.66	1376	1869.98	2272.66	2364.52	14.391771	12.298402	14.426088	12.164416	13.488174	13.570397	19.964223	20.050473	20.027889	28.518983	30.707586	33.943356	-0.067997	4.2899237	0.4962657	0.8711141	0.5464264	4.2899237	1.75E-08	2.10E-07	1.1804012	4.2899237	2.00E-34	2.70E-33
12693	ENSMUSG00	22381	Tceal9	1	protein codi	MGI Symbol	2	transcriptior	879	866	1033	906	1083	989	1736	1808	1507	2419	2859	2873	15.617736	15.04296	18.363934	14.89319	17.727782	17.339952	23.592846	21.988315	21.934614	36.892063	38.630059	41.242731	0.0272584	4.5668557	0.783243	0.9653561	0.461783	4.5668557	1.50E-06	1.29E-05	1.2521475	4.5668557	3.28E-39	5.31E-38
12701	ENSMUSG00	101148	Bmt2	1	protein codi	MGI Symbol	2	base methyl	417	417	374	409	421	411	629	708	601	929	1100	1006	7.4090968	7.2435501	6.648704	6.7233053	6.8914093	7.205986	8.5483295	8.6104685	8.7476464	14.168138	14.862912	14.441416	-0.033343	3.2274459	0.7649024	0.9602466	0.2821016	3.2274459	0.0068337	0.0189438	1.0290854	3.2274459	5.09E-24	4.18E-23
12725	ENSMUSG00	100129	Gpr153	1	protein codi	MGI Symbol	4	G protein-co	700	595	602	640	571	601	883	953	852	1616	2213	1775	12.437333	10.335521	10.701925	10.520576	9.3467808	10.53722	12.000278	11.59008	12.40099	24.645545	29.901476	25.480629	-0.138899	3.9141946	0.1731173	0.6310968	0.1042565	3.9141946	0.2970227	0.3881382	1.2579304	3.9141946	5.74E-38	8.93E-37
12792	ENSMUSG00	104027	Synpo	1	protein codi	MGI Symbol	12	synaptopodi	1111	1001	1072	1155	1175	1142	2257	2396	1775	3868	4519	4541	19.739824	17.387994	19.057248	18.986351	19.233743	20.022472	30.673418	29.139382	25.835395	58.990699	61.059544	65.187345	0.0518608	5.0103066	0.593363	0.9073765	0.6086203	5.0103066	1.54E-10	2.31E-09	1.7211609	5.0103066	9.12E-73	3.20E-71
12835	ENSMUSG00	384309	Trim56	1	protein codi	MGI Symbol	2	tripartite mc	366	306	341	349	336	356	391	377	296	788	1043	978	6.5029483	5.3154109	6.0620536	5.7370014	5.5000321	6.2416813	5.3138264	4.5849529	4.308325	12.017754	14.092743	14.039468	-0.033145	2.9135815	0.7739593	0.9623913	-0.330807	2.9135815	0.0026473	0.008701	1.1678628	2.9135815	1.41E-29	1.57E-28
12843	ENSMUSG00	26879	B3galnt1	1	protein codi	MGI Symbol	1	UDP-GalNAc	232	206	222	212	249	230	430	447	382	491	657	683	4.1220874	3.5783485	3.9465569	3.4849407	4.0759167	4.0325469	5.84385	5.4362704	5.5600681	7.4882195	8.8772118	9.804659	-0.006805	2.4858596	0.9713964	1	0.5317185	2.4858596	2.79E-06	2.27E-05	1.1689691	2.4858596	5.16E-26	4.83E-25
12898	ENSMUSG00	66873	Tril	1	protein codi	MGI Symbol	1	TLR4 interac	454.21	481.07	507.52	447.32	481.53	501.9	837.8	1308.77	866.44	2203.46	2703.7	2270.57	8.0702299	8.356486	9.0223268	7.3532248	7.8822335	8.7997186	11.385994	15.91684	12.611166	33.604872	36.531686	32.594677	-0.083219	4.0126165	0.4335892	0.8466798	0.6509858	4.0126165	1.09E-10	1.67E-09	2.0131517	4.0126165	1.32E-91	6.03E-90
12912	ENSMUSG00	74762	Mdga1	1	protein codi	MGI Symbol	10	MAM domai	126	123	117	175	189	185	302	375	299	717	986	836	2.2387199	2.1365867	2.0799422	2.8767199	3.0937681	3.2435703	4.1042854	4.5606295	4.3519905	10.934936	13.322574	12.001017	0.5121359	2.4662175	0.0001149	0.0030488	1.0121415	2.4662175	6.03E-16	1.50E-14	2.4897052	2.4662175	5.17E-99	2.73E-97
1292	ENSMUSG00	15586	Hyal1	1	protein codi	MGI Symbol	5	hyaluronoglu	474.86	309.37	460.5	305.5	362.63	370.68	723.38	428.95	409.29	1232.22	1389.77	1513.95	8.4371312	5.3739499	8.186439	5.021931	5.9359424	6.499063	9.8309866	5.2167521	5.9572782	18.792533	18.778208	21.733182	-0.333767	3.3306244	0.0021073	0.0380592	-0.067795	3.3306244	0.5321515	0.6172698	1.4314079	3.3306244	6.93E-46	1.35E-44
12941	ENSMUSG00	68918	1190005I06	1	protein codi	MGI Symbol	3	RIKEN cDNA	161.1	145.85	158.25	169.81	213.68	179.43	284.87	335.12	237.96	366.48	521.42	457.1	2.8623633	2.5335055	2.8132551	2.7914046	3.4977585	3.1459126	3.8714827	4.0756218	3.463544	5.5891705	7.0452904	6.5618003	0.2011919	2.033611	0.1158214	0.532319	0.4777819	2.033611	9.69E-05	0.0005342	1.2272085	2.033611	1.79E-25	1.61E-24
13005	ENSMUSG00	269999	Orai3	1	protein codi	MGI Symbol	3	ORAI calcium	199	184	215	236	244	224	367	445	348	593	687	570	3.535756	3.1961948	3.8221159	3.8794623	3.9940709	3.92735	4.9876581	5.411947	5.0651929	9.0438171	9.282564	8.1825119	0.1615088	2.4439045	0.1894068	0.6567026	0.5524064	2.4439045	1.81E-06	1.54E-05	1.3288261	2.4439045	1.71E-32	2.13E-31
13013	ENSMUSG00	217664	Mgat2	1	protein codi	MGI Symbol	1	mannoside a	571	553	619	600	600	667	1151	1171	940	1307	1574	1520	10.14531	9.6059549	11.004138	9.8630396	9.8214859	11.694386	15.642492	14.241326	13.681843	19.933	21.267476	21.820032	0.0282835	3.8223056	0.7844026	0.9653561	0.5025681	3.8223056	4.26E-07	4.09E-06	1.0351985	3.8223056	7.67E-26	7.04E-25
13069	ENSMUSG00	76522	Lsm8	1	protein codi	MGI Symbol	4	LSM8 homol	217	235	249	264	235	256	357	378	350	499	654	628	3.8555732	4.0820966	4.4265436	4.3397374	3.8467487	4.4884	4.8517546	4.5971146	5.0943032	7.6102272	8.8366766	9.0151183	0.034974	2.4556314	0.7738447	0.9623913	0.2326914	2.4556314	0.041267	0.0797633	1.0428309	2.4556314	2.76E-21	1.95E-20
13131	ENSMUSG00	277010	Marveld1	1	protein codi	MGI Symbol	2	MARVEL (me	214	177	204	185	193	175	299	311	245	455	543	469	3.8022703	3.0746004	3.6265658	3.0411039	3.1592446	3.0682422	4.0635143	3.7822821	3.5660123	6.9391851	7.3368737	6.7326282	-0.179576	2.1416171	0.15471	0.6000942	0.1208994	2.1416171	0.3190905	0.410642	1.0007661	2.1416171	1.23E-18	7.29E-18
13163	ENSMUSG00	217835	Rin3	1	protein codi	MGI Symbol	7	Ras and Rab	97	73	102	110	112	121	389	364	263	538	758	645	1.723459	1.2680555	1.8132829	1.8082239	1.833344	2.1214703	5.2866457	4.4268511	3.828005	8.2050145	10.241897	9.2591582	0.2615435	2.1511862	0.0783011	0.4454986	1.4966235	2.1511862	1.45E-30	7.32E-29	2.5290499	2.1511862	7.40E-92	3.41E-90
13192	ENSMUSG00	594844	Tceal3	1	protein codi	MGI Symbol	7	transcriptior	661.82	684.32	723.89	710.7	731.76	703.84	1354.01	1463.8	1163.23	1597.97	1780.76	2039.88	11.758965	11.887065	12.868798	11.68277	11.978284	12.340295	18.401468	17.802265	16.93099	24.370571	24.061162	29.283057	-0.020547	4.0908245	0.8429649	0.9799518	0.5413611	4.0908245	3.15E-08	3.65E-07	1.0896141	4.0908245	3.70E-29	4.01E-28
13318	ENSMUSG00	103768	Tubg2	1	protein codi	MGI Symbol	1	tubulin, gam	313.25	273.53	321.16	319.33	317.9	254.72	436.67	490.07	369.81	683	845.13	769.61	5.5657064	4.7513867	5.7093523	5.2492741	5.2037506	4.465958	5.9344977	5.9600739	5.3826408	10.416403	11.419175	11.04797	-0.101619	2.7714949	0.3691902	0.8079491	0.109977	2.7714949	0.3235711	0.4151709	1.0375732	2.7714949	6.44E-23	5.03E-22
13372	ENSMUSG00	110380	Shroom2	1	protein codi	MGI Symbol	6	shroom fam	302	263	294	299	307	328	495	636	489	711	801	828	5.3658207	4.568474	5.2265213	4.9150814	5.025327	5.7507625	6.7272227	7.7348277	7.1174694	10.84343	10.822902	11.886175	0.0488479	2.8572424	0.6679893	0.9330831	0.5105945	2.8572424	2.48E-06	2.04E-05	1.146156	2.8572424	1.86E-27	1.87E-26
13381	ENSMUSG00	50785	Hs6st1	1	protein codi	MGI Symbol	2	heparan sulf	2211.09	2093.43	2213	2309.47	2312.62	2404	4024.8	4664.83	3566.47	5230.92	6126.66	5651.75	39.285803	36.364185	39.341128	37.96399	37.855608	42.148881	54.698436	56.732164	51.910513	79.776532	82.781824	81.132476	0.0367586	5.7395488	0.6937534	0.9424837	0.5065396	5.7395488	4.30E-08	4.86E-07	1.0835637	5.7395488	1.60E-31	1.94E-30
13494	ENSMUSG00	12091	Glb1	1	protein codi	MGI Symbol	2	galactosidas	1387.71	1363.25	1536.44	1755.33	1746.2	1611.42	2817.97	3506.17	2672.19	3621.17	4681.43	4335.27	24.656301	23.680503	27.313729	28.854816	28.583798	28.252725	38.297196	42.640913	38.894132	55.226305	63.254255	62.234032	0.1798899	5.2704261	0.0573124	0.3834055	0.6638678	5.2704261	1.43E-12	2.71E-11	1.2564534	5.2704261	9.19E-41	1.56E-39
13515	ENSMUSG00	114716	Spred2	1	protein codi	MGI Symbol	3	sprouty-rela	248	222	230	221	243	246	481	540	421	1030	1213	1065	4.4063693	3.8562785	4.0887752	3.6328863	3.9777018	4.3130719	6.5369578	6.5673065	6.127719	15.708485	16.389738	15.288377	-0.051646	2.9418344	0.6775078	0.9366622	0.6396186	2.9418344	1.05E-08	1.30E-07	1.9397496	2.9418344	2.84E-72	9.85E-71
13516	ENSMUSG00	373864	Col27a1	1	protein codi	MGI Symbol	7	collagen, typ	416	375	372	473	510	483	1089	1061	833	2091	2830	3070	7.3913292	6.5139839	6.6131494	7.7753629	8.348263	8.4683485	14.79989	12.903541	12.124442	31.88975	38.238218	44.070722	0.2610867	4.0650651	0.014097	0.1601935	0.9570415	4.0650651	7.69E-21	2.64E-19	2.4766709	4.0650651	1.18E-133	1.18E-131
13550	ENSMUSG00	NA	Gm9800	1	processed p	MGI Symbol	1	predicted ge	177.25	164.51	192.17	286.47	252.77	187.82	309.81	294.66	291.46	377.68	488.68	747.32	3.1493103	2.8576413	3.4162605	4.7091083	4.1376283	3.2930129	4.210426	3.5835603	4.2422446	5.7599812	6.6029161	10.727991	0.3681488	2.2601652	0.0026358	0.0456942	0.3506266	2.2601652	0.0035217	0.0110234	1.2938091	2.2601652	1.02E-29	1.13E-28
13576	ENSMUSG00	230279	Tmem268	1	protein codi	MGI Symbol	5	transmembr	269	238	219	299	272	281	580	657	541	901	1219	1024	4.7794893	4.1342084	3.8932251	4.9150814	4.452407	4.9267203	7.8824024	7.9902229	7.8743373	13.741112	16.470809	14.699811	0.1581147	3.0166135	0.177172	0.6359708	0.8908079	3.0166135	4.90E-16	1.23E-14	1.8105774	3.0166135	1.38E-63	4.10E-62
13585	ENSMUSG00	20324	Cavin2	1	protein codi	MGI Symbol	1	caveolae ass	271	208	220	395	441	388	848	860	645	932	1240	1090	4.8150245	3.6130897	3.9110023	6.4931678	7.2187922	6.8027313	11.524616	10.459044	9.3880731	14.213891	16.754555	15.64726	0.7337561	3.2258637	7.40E-11	5.54E-09	1.3470105	3.2258637	3.85E-35	2.36E-33	1.91828	3.2258637	1.18E-70	3.98E-69
13636	ENSMUSG00	268567	Tmem229b	1	protein codi	MGI Symbol	7	transmembr	192	168	154	192	152	195	278	364.95	240	435	711	518	3.4113827	2.9182648	2.7377016	3.1561727	2.4881098	3.4188984	3.778117	4.4384047	3.4932365	6.634166	9.6068457	7.4360371	-0.002664	2.1834482	1	1	0.3729194	2.1834482	0.0022056	0.0074725	1.3869417	2.1834482	1.08E-33	1.41E-32
13704	ENSMUSG00	19659	Rbp1	1	protein codi	MGI Symbol	1	retinol bindi	411	301	571	491	518	458	305	339	230	691	1399	1369	7.3024911	5.2285577	10.150829	8.0712541	8.4792162	8.0300281	4.1450564	4.1228091	3.347685	10.538411	18.902921	19.652384	0.1171116	3.1768619	0.2720817	0.7416878	-0.961183	3.1768619	1.97E-18	5.91E-17	1.1167579	3.1768619	2.14E-28	2.25E-27
13711	ENSMUSG00	192976	Lrrc75a	1	protein codi	MGI Symbol	3	leucine rich	224	198	164	249	179	192	381	442	351	469	646	591	3.9799465	3.4393835	2.9154745	4.0931614	2.9300766	3.3663	5.1779229	5.375462	5.1088584	7.1526985	8.7285827	8.4839728	0.0078582	2.3638959	0.9687444	1	0.600198	2.3638959	2.14E-07	2.18E-06	1.2381526	2.3638959	4.99E-28	5.17E-27
1377	ENSMUSG00	18648	Pgam1	1	protein codi	MGI Symbol	3	phosphoglyc	6639.63	6427.61	6793.29	7023.1	6939.57	6911.52	11711.02	12327.78	9929.02	17955.7	24138.75	24059.7	117.97041	111.65159	120.76624	115.44852	113.59482	121.17838	159.15685	149.9265	144.5184	273.8416	326.15646	345.38383	-0.000704	7.4518309	0.9945056	1	0.3724954	7.4518309	3.96E-05	0.0002429	1.4319783	7.4518309	1.16E-54	2.86E-53
1383	ENSMUSG00	12175	Bnip2	1	protein codi	MGI Symbol	8	BCL2/adeno	442.9	422.8	387.9	491	445	487.63	785.83	760.81	612.96	933.72	1079.8	1135.88	7.8692781	7.3442997	6.8958082	8.0712541	7.2842687	8.5495254	10.679704	9.2527268	8.921726	14.240123	14.589975	16.30588	0.1119778	3.3326989	0.292091	0.7550319	0.3841327	3.3326989	0.0002015	0.0010051	1.0295308	3.3326989	3.27E-24	2.72E-23
13876	ENSMUSG00	211949	Spsb4	1	protein codi	MGI Symbol	4	splA/ryanod	323	269.93	325.67	364.22	329.52	358.82	483.78	535.38	472.34	752.35	885.42	784.41	5.7389407	4.6888525	5.7895279	5.9871938	5.3939601	6.2911238	6.574739	6.5111196	6.8749805	11.474057	11.963563	11.260428	0.1236771	2.8968527	0.2693048	0.7394206	0.2994879	2.8968527	0.0057453	0.0164695	1.0977792	2.8968527	1.21E-25	1.10E-24
13895	ENSMUSG00	209497	Tmem164	1	protein codi	MGI Symbol	9	transmembr	811	741	793	758	670	800	1448	1656	1247	2235	2727	2430	14.409538	12.871632	14.097386	12.460307	10.967326	14.02625	19.678825	20.13974	18.150275	34.085887	36.846509	34.88334	-0.144386	4.3450428	0.1483279	0.5900779	0.4869262	4.3450428	5.27E-07	4.97E-06	1.3547546	4.3450428	7.36E-45	1.42E-43
13990	ENSMUSG00	399558	Flrt2	1	protein codi	MGI Symbol	2	fibronectin l	4018	3814	4314	4522	5025	4895	6178	7395	6238	9928	11342	12779	71.39029	66.251559	76.6912	74.334442	82.254945	85.823117	83.961176	89.935614	90.795039	151.41149	153.25013	183.44617	0.1775715	6.656503	0.0520278	0.3633439	0.3044848	6.656503	0.0008431	0.0034186	1.1873627	6.656503	3.64E-38	5.68E-37
14029	ENSMUSG00	74325	Cltb	1	protein codi	MGI Symbol	4	clathrin, ligh	1354	1567	1383	1583	1576	1462	2874	3148	2404	3445	4488	4138	24.057355	27.219767	24.585983	26.021986	25.79777	25.632972	39.058663	38.284965	34.990586	52.539544	60.64068	59.402165	0.0298477	5.1947123	0.754851	0.958233	0.5664009	5.1947123	1.53E-09	2.08E-08	1.1858326	5.1947123	1.50E-36	2.21E-35
14037	ENSMUSG00	378435	Mafa	1	protein codi	MGI Symbol	1	v-maf muscu	243	170	182	237	198	195	297	335	368	611	773	712	4.3175312	2.953006	3.2354656	3.8959007	3.2410904	3.4188984	4.0363336	4.0741624	5.356296	9.3183343	10.444573	10.220962	0.0079384	2.4479793	0.9691376	1	0.3549329	2.4479793	0.0023457	0.0078548	1.5138821	2.4479793	4.97E-42	8.71E-41
14041	ENSMUSG00	212398	Frat2	1	protein codi	MGI Symbol	1	frequently re	155	148	190	116	171	154	269	285	244.98	398	527.71	457	2.7539808	2.5708523	3.3776838	1.9068543	2.7991235	2.7000531	3.6558039	3.4660784	3.5657212	6.0698806	7.1302792	6.5603648	-0.23331	1.9825215	0.0752796	0.4339538	0.2963374	1.9825215	0.0155517	0.0366331	1.1845029	1.9825215	2.95E-24	2.47E-23
14043	ENSMUSG00	97159	A430005L14	1	protein codi	MGI Symbol	7	RIKEN cDNA	158	146	143	163	119	153	276	274	231	345	565	437	2.8072837	2.5361111	2.5421515	2.6794591	1.947928	2.6825203	3.7509363	3.3323	3.3622401	5.2615799	7.6341319	6.2732591	-0.110984	1.9302852	0.4286479	0.8463211	0.4050891	1.9302852	0.0011278	0.004336	1.2840668	1.9302852	2.01E-27	2.01E-26
1417	ENSMUSG00	104156	Etv5	1	protein codi	MGI Symbol	5	ets variant 5	1679.74	1506.17	1596.19	1655.53	1757.61	1813.32	5979.25	7020.63	5120.02	10730.48	12808.74	11853.06	29.844979	26.163112	28.375922	27.214263	28.77057	31.7926	81.260094	85.382647	74.52267	163.65008	173.06834	170.15404	0.0567028	6.2642954	0.5483182	0.891083	1.5151524	6.2642954	1.63E-58	2.04E-56	2.5865945	6.2642954	1.75E-158	2.24E-156
14187	ENSMUSG00	23966	Tenm4	1	protein codi	MGI Symbol	12	teneurin trai	1766	1502.32	1857.43	1587	1899	1759	2220	3010.45	2813.37	5033.05	6091.09	5964.54	31.377614	26.096235	33.020059	26.08774	31.085003	30.840217	30.170575	36.612126	40.949028	76.758826	82.301212	85.622665	-0.040099	5.4705809	0.6716961	0.9350986	0.2514407	5.4705809	0.0070471	0.0193601	1.4351561	5.4705809	6.10E-53	1.43E-51
14233	ENSMUSG00	208440	Dip2c	1	protein codi	MGI Symbol	4	disco interac	571	455	485	595	578	511	878	956	790	1127	1322	1380	10.14531	7.9036338	8.6219824	9.7808476	9.4613648	8.9592672	11.932326	11.626565	11.49857	17.187828	17.862518	19.810292	0.0811377	3.6022828	0.4384778	0.8505513	0.3947321	3.6022828	9.50E-05	0.0005242	1.0407791	3.6022828	1.54E-25	1.38E-24
14263	ENSMUSG00	14062	F2r	1	protein codi	MGI Symbol	1	coagulation	2118	1814	2273	1891	2027	2050	1284	1265	989	1993	3477	3205	37.631815	31.510311	40.407765	31.085013	33.180253	35.942266	17.450008	15.384524	14.395045	30.395156	46.980313	46.008685	-0.128664	4.9858969	0.1689366	0.6268354	-1.213386	4.9858969	3.50E-37	2.36E-35	0.1723505	4.9858969	0.0640188	0.0747424
14303	ENSMUSG00	216829	Mmgt2	1	protein codi	MGI Symbol	7	membrane r	130	113	104	136	126	129	259	312	221	339	436	404	2.3097904	1.9628805	1.8488375	2.2356223	2.062512	2.2617328	3.5199004	3.7944438	3.2166886	5.1700742	5.8911177	5.7995347	0.0992397	1.775487	0.4990843	0.8714425	0.785162	1.775487	1.07E-09	1.47E-08	1.4621969	1.775487	3.93E-32	4.81E-31
14441	ENSMUSG00	71703	Armcx3	1	protein codi	MGI Symbol	3	armadillo re	834	756.17	846	964	946.93	913	1305.46	1521.21	1123	1739.82	2147.15	2144.48	14.818194	13.135145	15.039582	15.846617	15.500433	16.007458	17.741657	18.500467	16.345436	26.533919	29.011728	30.784619	0.1394697	4.2617013	0.156719	0.6054618	0.2913531	4.2617013	0.0027432	0.0089525	1.0060317	4.2617013	1.53E-25	1.38E-24
14500	ENSMUSG00	280411	Lix1l	1	protein codi	MGI Symbol	2	Lix1-like [Sou	439	463	479	406	431	428	732.53	859	660.97	986	1323	1163	7.7999844	8.0425988	8.5153187	6.6739902	7.0551007	7.5040438	9.9553383	10.446882	9.6205189	15.037443	17.876029	16.695195	-0.198532	3.3949108	0.062095	0.3941672	0.3023099	3.3949108	0.0032175	0.0102196	1.026708	3.3949108	1.66E-24	1.41E-23
14535	ENSMUSG00	103172	Chchd10	1	protein codi	MGI Symbol	3	coiled-coil-h	353	367	402.92	394	407	379	660	678.91	564	914.87	1289.8	1085.74	6.2719692	6.3750189	7.162823	6.4767294	6.6622413	6.644936	8.9696303	8.2566853	8.2091058	13.952642	17.42744	15.586106	-0.001828	3.2355896	0.9954482	1	0.3605959	3.2355896	0.0005898	0.0025376	1.2461668	3.2355896	2.64E-34	3.53E-33
14567	ENSMUSG00	56430	Clip1	1	protein codi	MGI Symbol	15	CAP-GLY dor	174	135	180	136	155	165	296	383	268	495	564	610	3.0915656	2.3450342	3.199911	2.2356223	2.5372172	2.8929141	4.0227433	4.657923	3.9007808	7.5492234	7.6206202	8.7567232	-0.173017	2.1659496	0.1881084	0.654813	0.5464318	2.1659496	6.32E-06	4.73E-05	1.470584	2.1659496	2.87E-37	4.34E-36
14593	ENSMUSG00	235504	Slc17a5	1	protein codi	MGI Symbol	3	solute carrie	140	129	170	124	146	128	242	259	220	357	454	409	2.4874666	2.2408105	3.0221382	2.0383615	2.3898949	2.2442	3.2888644	3.1498748	3.2021335	5.4445914	6.134329	5.8713111	-0.21513	1.8194634	0.1168563	0.5328909	0.3150957	1.8194634	0.0121945	0.0302081	1.172124	1.8194634	1.02E-22	7.87E-22
14610	ENSMUSG00	109305	Orai1	1	protein codi	MGI Symbol	3	ORAI calcium	264	298	239	324	270	305	539	556	452	784	901	832	4.6906512	5.1764459	4.2487707	5.3260414	4.4196687	5.3475078	7.3251981	6.7618934	6.5789287	11.95675	12.174076	11.943596	0.0952977	2.8577623	0.4110519	0.8370832	0.5494608	2.8577623	4.89E-07	4.64E-06	1.3529417	2.8577623	1.28E-36	1.89E-35
14636	ENSMUSG00	19241	Tmsb4x	1	protein codi	MGI Symbol	3	thymosin, be	4398	4505	4321	5267	5769	5171	14178	14141	12439	19357	23584	23497	78.141985	78.25466	76.815641	86.58105	94.433587	90.662174	192.68397	171.9783	181.05154	295.21276	318.66083	337.30611	0.2202407	7.3834542	0.0157819	0.1727286	1.2264445	7.3834542	1.21E-40	9.21E-39	2.0280608	7.3834542	7.88E-104	4.54E-102
14643	ENSMUSG00	100503043	Armcx4	1	protein codi	MGI Symbol	1	armadillo re	984	896	1072	913	979	957	1438	1845	1556	2338	2569	2826	17.483336	15.564079	19.057248	15.008259	16.025391	16.778902	19.542922	22.438297	22.647817	35.656736	34.711655	40.568033	-0.124088	4.5262565	0.2040058	0.6760426	0.3109492	4.5262565	0.0011819	0.0045004	1.0902915	4.5262565	2.61E-30	3.02E-29
1469	ENSMUSG00	56398	Chp1	1	protein codi	MGI Symbol	5	calcineurin-l	603	542	612.92	572	614.94	586.92	978	1172.92	946.9	1334.85	1680.17	1528.88	10.713874	9.4148781	10.896053	9.4027644	10.066041	10.290358	13.291361	14.264676	13.782274	20.357739	22.702017	21.947507	-0.060108	3.8088602	0.560694	0.8961357	0.4144777	3.8088602	3.15E-05	0.0001991	1.0675839	3.8088602	1.96E-27	1.97E-26
14748	ENSMUSG00	107515	Lgr4	1	protein codi	MGI Symbol	4	leucine-rich	2698	2337	2193	2554	2618	2482	5231	6136	4867	6600	8070	7033	47.937034	40.595148	38.985582	41.983672	42.854417	43.516441	71.091115	74.624061	70.839925	100.65631	109.03973	100.96071	0.0094328	6.0306799	0.9205629	0.9921337	0.7641146	6.0306799	1.22E-16	3.16E-15	1.2846695	6.0306799	1.40E-43	2.60E-42
14786	ENSMUSG00	16854	Lgals3	1	protein codi	MGI Symbol	6	lectin, galact	321	336	358	572	637	583	951	1129	742	2561	4467	4911	5.7034054	5.8365296	6.3642674	9.4027644	10.427144	10.22163	12.924422	13.730535	10.799923	39.057699	60.356933	70.4988	0.7468929	4.4224731	1.62E-12	1.45E-10	1.0662317	4.4224731	6.80E-25	2.78E-23	3.2465571	4.4224731	8.95E-221	2.30E-218
14795	ENSMUSG00	101113	Snx21	1	protein codi	MGI Symbol	7	sorting nexir	285.49	278.21	274.98	291.09	286.55	369.57	487.54	486.77	425.24	596.35	804.32	698.27	5.0724773	4.8326812	4.8883974	4.7850537	4.690578	6.4796015	6.6258387	5.9199404	6.1894329	9.0949078	10.867761	10.023864	0.1066125	2.742189	0.3439649	0.7903445	0.3400912	2.742189	0.0019343	0.0067041	1.0201617	2.742189	9.58E-22	7.01E-21
148	ENSMUSG00	14265	Fmr1	1	protein codi	MGI Symbol	13	fragile X mer	589	481	549.07	518.01	613.03	599	982.03	1045.16	883	1204.03	1469.03	1489	10.465127	8.35527	9.760973	8.5152553	10.034776	10.502155	13.34613	12.7109	12.852199	18.362609	19.849148	21.375018	0.0233005	3.7108362	0.8257539	0.975947	0.4451759	3.7108362	8.95E-06	6.47E-05	1.0603343	3.7108362	9.45E-27	9.17E-26
14813	ENSMUSG00	320343	Lypd6	1	protein codi	MGI Symbol	5	LY6/PLAUR c	343	292	327	404	349	332	503	602	492	895	1042	919	6.0942931	5.0722221	5.8131717	6.6411134	5.712831	5.8208938	6.8359455	7.3213306	7.1611348	13.649606	14.079231	13.192506	0.0988512	3.0347298	0.3776201	0.8140211	0.3289136	3.0347298	0.0022334	0.0075373	1.2692842	3.0347298	1.87E-34	2.53E-33
14876	ENSMUSG00	14325	Ftl1	1	protein codi	MGI Symbol	6	ferritin light	51885.5	51458.48	54632.21	53792.66	55017.05	53094.89	97043.99	97984.66	86413.67	141573.93	170470.7	149058.12	921.88176	893.86589	971.21228	884.26523	900.58197	930.90275	1318.8617	1191.658	1257.7641	2159.1378	2303.3554	2139.7716	-0.037343	10.369461	0.6773546	0.9366622	0.435211	10.369461	1.28E-06	1.12E-05	1.2442705	10.369461	1.52E-42	2.70E-41
14881	ENSMUSG00	22320	Vamp8	1	protein codi	MGI Symbol	2	vesicle-assoc	146	158	186	180	174	173	325	298	263	404	625	497	2.5940723	2.7445585	3.3065747	2.9589119	2.8482309	3.0331766	4.4168634	3.6241803	3.828005	6.1613863	8.4448362	7.1345761	0.0319759	2.1165163	0.8236919	0.975947	0.456021	2.1165163	0.0001665	0.0008547	1.3324565	2.1165163	1.43E-30	1.66E-29
1492	ENSMUSG00	26407	Map3k4	1	protein codi	MGI Symbol	9	mitogen-acti	1007	941	843	1095	1153	1064	1563	1693	1492.99	2030	2504	2348	17.891992	16.345757	14.98625	18.000047	18.873622	18.654913	21.241715	20.589722	21.730697	30.959441	33.833392	33.706207	0.1738222	4.4797627	0.0737527	0.4294221	0.3685404	4.4797627	0.0001248	0.0006659	1.0008444	4.4797627	1.18E-25	1.07E-24
15007	ENSMUSG00	18795	Plcb1	1	protein codi	MGI Symbol	8	phospholipa	158	147	163	169	191	197	299	307	270	436.99	378.98	438.99	2.8072837	2.5534817	2.8976972	2.7780895	3.1265064	3.4539641	4.0635143	3.7336354	3.9298911	6.6645154	5.1206785	6.3018261	0.1791019	2.0057841	0.1623529	0.614973	0.5050714	2.0057841	3.47E-05	0.0002174	1.1286945	2.0057841	1.09E-21	7.94E-21
15056	ENSMUSG00	52055	Rab11fip5	1	protein codi	MGI Symbol	3	RAB11 famil	166	162	205	164	152	194	371	408	362	666	894	762	2.9494246	2.814041	3.6443431	2.6958975	2.4881098	3.4013656	5.0420195	4.9619649	5.2689651	10.157137	12.079494	10.938726	-0.134391	2.4956376	0.3050715	0.7643114	0.6984989	2.4956376	2.40E-09	3.17E-08	1.8190268	2.4956376	7.67E-59	2.06E-57
151	ENSMUSG00	59038	Pxmp4	1	protein codi	MGI Symbol	2	peroxisomal	149	155	181	222	182	190	213	244	226	342	452	422	2.6473751	2.6924467	3.2176883	3.6493247	2.9791841	3.3312344	2.8947443	2.9674496	3.2894644	5.2158271	6.1073056	6.0579298	0.2189505	1.928616	0.0875139	0.4697071	0.0953648	1.928616	0.4530091	0.5427653	1.0234246	1.928616	4.00E-18	2.30E-17
15170	ENSMUSG00	216856	Nlgn2	1	protein codi	MGI Symbol	4	neuroligin 2	836	647	792	765	767	765	1118	1453	1173	1703	2093	2018	14.853729	11.238794	14.079608	12.575376	12.555133	13.412602	15.19401	17.670919	17.073193	25.972379	28.280068	28.968963	-0.059648	4.1489221	0.5517694	0.8925267	0.3145146	4.1489221	0.0012836	0.0048124	1.051177	4.1489221	1.56E-27	1.57E-26
15199	ENSMUSG00	381694	B3glct	1	protein codi	MGI Symbol	3	beta-3-glucc	244	221	249	240	230	255	417	427	342	651	715	721	4.3352988	3.8389078	4.4265436	3.9452159	3.7649029	4.4708672	5.6671755	5.1930368	4.977862	9.9283726	9.6608926	10.35016	-0.049825	2.5742061	0.6949192	0.9427892	0.3304759	2.5742061	0.0034306	0.010781	1.248391	2.5742061	2.37E-30	2.74E-29
15221	ENSMUSG00	50794	Klf13	1	protein codi	MGI Symbol	4	Kruppel-like	411	339	373	430	415	378	624	659	556	1184	1402	1266	7.3024911	5.8886414	6.6309267	7.0685117	6.7931944	6.6274031	8.4803777	8.0145463	8.0926646	18.057132	18.943457	18.17379	0.0483799	3.3354812	0.6634815	0.9319939	0.3109556	3.3354812	0.0031249	0.009992	1.4771542	3.3354812	5.81E-48	1.18E-46
15334	ENSMUSG00	20447	St6galnac3	1	protein codi	MGI Symbol	5	ST6 (alpha-N	152	116	206	100	89	82	330	336	229	546	940	889	2.700678	2.0149924	3.6621204	1.6438399	1.4568537	1.4376906	4.4848151	4.0863241	3.3331298	8.3270221	12.701034	12.761847	-0.880875	2.3224273	3.98E-10	2.76E-08	0.5107131	2.3224273	2.84E-05	0.0001828	2.0148572	2.3224273	1.01E-69	3.31E-68
15363	ENSMUSG00	215449	Rap1b	1	protein codi	MGI Symbol	2	RAS related	1716	1663	1743	1852	2066	1900	3449	3628	3161	6490	7810	7586	30.489233	28.887347	30.985805	30.443916	33.81865	33.312344	46.873113	44.12257	46.008836	98.978706	105.52667	108.89918	0.1107662	5.7365421	0.2385213	0.7163933	0.6003432	5.7365421	1.15E-10	1.75E-09	1.7942492	5.7365421	1.05E-80	4.12E-79
1540	ENSMUSG00	27362	Dnajb9	1	protein codi	MGI Symbol	2	DnaJ heat sh	306	261	319.96	252	342	320.97	637	712	630	1016.98	1221	1285.99	5.4368912	4.5337328	5.6880196	4.1424766	5.598247	5.6275068	8.6570523	8.6591153	9.1697458	15.509918	16.497832	18.460752	-0.027503	3.18696	0.8198742	0.9746325	0.7581053	3.18696	1.08E-12	2.08E-11	1.6888099	3.18696	6.94E-60	1.90E-58
15409	ENSMUSG00	16779	Lamb2	1	protein codi	MGI Symbol	7	laminin, beta	583	445	523	536	579	546	949	1115	1034	2427	2711	2541	10.358521	7.7299276	9.2975192	8.8109821	9.4777339	9.5729156	12.897241	13.560272	15.050027	37.014071	36.630322	36.476777	0.025036	4.1176381	0.8155633	0.9734524	0.5996735	4.1176381	2.15E-09	2.87E-08	2.0077462	4.1176381	7.16E-92	3.32E-90
15424	ENSMUSG00	14451	Gas1	1	protein codi	MGI Symbol	2	growth arres	462.7	349.17	327.4	380.88	397.07	417.95	1170.6	1324.29	958.31	2631.29	2419.53	1870.14	8.221077	6.0653007	5.8202826	6.2610576	6.4996957	7.327839	15.908862	16.10559	13.948348	40.129689	32.692055	26.846391	-0.001891	3.966152	0.9974475	1	1.1936254	3.966152	9.61E-32	5.05E-30	2.3089672	3.966152	2.61E-116	2.00E-114
1544	ENSMUSG00	70231	Gorasp2	1	protein codi	MGI Symbol	6	golgi reasser	2150.11	1993.65	2215.43	2304.67	2396.97	2338.1	3983.26	4409.94	3675.72	5106.76	5855.58	6072.76	38.202334	34.630944	39.384327	37.885086	39.236345	40.993469	54.133893	53.632273	53.500664	77.882974	79.119063	87.176194	0.0738604	5.7300162	0.4283379	0.8463211	0.5231919	5.7300162	1.56E-08	1.89E-07	1.1216708	5.7300162	1.35E-33	1.77E-32
15446	ENSMUSG00	72635	Lins1	1	protein codi	MGI Symbol	12	lines homolo	214	236	239	248	253	248	388	444	350	542	647	652	3.8022703	4.0994672	4.2487707	4.076723	4.1413932	4.3481375	5.2730554	5.3997854	5.0943032	8.2660183	8.7420945	9.3596452	0.0479686	2.4966688	0.6946007	0.9426793	0.376454	2.4966688	0.0009344	0.0037156	1.1176815	2.4966688	3.23E-24	2.69E-23
15450	ENSMUSG00	22601	Yap1	1	protein codi	MGI Symbol	10	yes-associat	1941.99	1718	1865.99	1862.98	1796	1893	3284	3554.99	2856	4536	5863	5419.96	34.504537	29.842732	33.172233	30.624409	29.398981	33.189614	44.630706	43.234646	41.569514	69.178338	79.21932	77.805065	-0.06527	5.5119331	0.4881051	0.869911	0.4085891	5.5119331	1.11E-05	7.90E-05	1.2140048	5.5119331	9.91E-39	1.58E-37
15452	ENSMUSG00	12702	Socs3	1	protein codi	MGI Symbol	3	suppressor c	260	244	230	328	317	318	484	485	414	741	1093	909	4.6195807	4.2384322	4.0887752	5.391795	5.1890184	5.5754344	6.5777289	5.8984142	6.025833	11.300959	14.76833	13.048953	0.3190201	2.8711577	0.0052415	0.0779246	0.5145561	2.8711577	3.96E-06	3.14E-05	1.5961171	2.8711577	1.28E-49	2.75E-48
1547	ENSMUSG00	76740	Efr3a	1	protein codi	MGI Symbol	14	EFR3 homolo	643	590	610	649	739	704	962	1118	928	1406	1752	1657	11.424579	10.248668	10.844143	10.668521	12.096797	12.3431	13.073916	13.596757	13.507181	21.442845	23.672565	23.786706	0.1103882	3.8878331	0.275953	0.7450743	0.3053442	3.8878331	0.002138	0.00728	1.0836059	3.8878331	2.05E-28	2.16E-27
15536	ENSMUSG00	21413	Tcf4	1	protein codi	MGI Symbol	62	transcriptior	1138	1097	1196	1035	1244	1124	2151	2398	1926	3469	3922	3661	20.21955	19.055574	21.261631	17.013743	20.363214	19.706881	29.232841	29.163706	28.033223	52.905567	52.993036	52.554695	-0.084768	4.9219927	0.380462	0.8167045	0.5138735	4.9219927	5.90E-08	6.56E-07	1.3881702	4.9219927	1.50E-48	3.12E-47
15564	ENSMUSG00	14218	Sh3pxd2a	1	protein codi	MGI Symbol	4	SH3 and PX c	871	744	835	757	776	766	1171	1485	1179	3390	4163	4067	15.475595	12.923744	14.844032	12.443868	12.702455	13.430134	15.914299	18.060093	17.160524	51.700742	56.249365	58.38294	-0.164795	4.6473363	0.0969201	0.4889758	0.2423387	4.6473363	0.0127939	0.0313092	1.9436789	4.6473363	4.75E-90	2.09E-88
15582	ENSMUSG00	235584	Dusp7	1	protein codi	MGI Symbol	4	dual specific	391	331.99	334	357	438	392	692	677	532	902	1070	1004	6.9471387	5.7668734	5.9376126	5.8685086	7.1696847	6.8728625	9.4045215	8.2334565	7.7433409	13.756363	14.45756	14.412705	0.0942043	3.1639428	0.3911062	0.8252156	0.4449204	3.1639428	2.44E-05	0.0001593	1.1926655	3.1639428	4.17E-31	4.96E-30
15607	ENSMUSG00	108058	Camk2d	1	protein codi	MGI Symbol	28	calcium/calr	588	536	463	651	754	704	1133	1238	1150	1806	2246	2198	10.44736	9.3106543	8.2308822	10.701398	12.342334	12.3431	15.397865	15.056158	16.738425	27.543227	30.347363	31.552914	0.3380186	4.0682595	0.0008936	0.0183355	0.7530129	4.0682595	3.93E-14	8.46E-13	1.6761238	4.0682595	8.05E-65	2.42E-63
15637	ENSMUSG00	71994	Cnn3	1	protein codi	MGI Symbol	8	calponin 3, a	9869.12	9706.04	10596.63	11347.25	11296.42	11254.71	20087.95	22139.93	17753.44	31196.85	40854.27	41969.34	175.35076	168.59997	188.37929	186.53063	184.91272	197.32672	273.00225	269.25872	258.40402	475.7818	552.01219	602.48179	0.0955129	8.2019236	0.2904531	0.7545748	0.5888954	8.2019236	7.48E-11	1.15E-09	1.6147408	8.2019236	1.10E-68	3.56E-67
15644	ENSMUSG00	242202	Pde5a	1	protein codi	MGI Symbol	4	phosphodies	432	334	444	352	343	392	688	732	571	890	1143	1094	7.6756111	5.8017883	7.8931138	5.7863166	5.6146161	6.8728625	9.3501601	8.9023488	8.3109918	13.573351	15.443916	15.704681	-0.2265	3.2213445	0.0372224	0.3037914	0.3148509	3.2213445	0.0025338	0.0084077	1.0662909	3.2213445	1.05E-25	9.55E-25
15656	ENSMUSG00	103466	Nt5dc3	1	protein codi	MGI Symbol	2	5prime-nucl	317	325	383	422	451.35	427.23	675.15	741.25	622	773	983.27	827.47	5.632335	5.6454527	6.8086995	6.9370045	7.3882128	7.4905435	9.1755241	9.0148444	9.0533046	11.788989	13.285687	11.878567	0.270356	3.1298273	0.0126091	0.1478452	0.5910432	3.1298273	1.89E-08	2.26E-07	1.0313802	3.1298273	2.66E-23	2.11E-22
1567	ENSMUSG00	109711	Actn1	1	protein codi	MGI Symbol	7	actinin, alph	1345.11	1124.81	1047.08	1542.93	1488.85	1466.89	2150.4	2341.57	2088.05	3407.53	3956	3533.63	23.899401	19.538651	18.614238	25.3633	24.371199	25.718707	29.224686	28.477422	30.391885	51.968091	53.452435	50.726262	0.2820654	4.9956814	0.0031183	0.0523382	0.5053917	4.9956814	9.29E-08	1.00E-06	1.3313741	4.9956814	6.45E-45	1.24E-43
15711	ENSMUSG00	16880	Lifr	1	protein codi	MGI Symbol	8	LIF receptor	380	354	392	481	547	545	672	735	602	941	1071	970	6.7516949	6.1492008	6.9686951	7.9068701	8.9539213	9.5553828	9.1327145	8.9388339	8.7622016	14.35115	14.471071	13.924625	0.4103425	3.2815824	0.0001068	0.002857	0.4337637	3.2815824	3.36E-05	0.0002111	1.1053089	3.2815824	3.54E-27	3.51E-26
15740	ENSMUSG00	76687	Spcs3	1	protein codi	MGI Symbol	1	signal peptic	558	496	527	540	514	520	1016	971	861	1213	1524	1481	9.914331	8.6158294	9.3686283	8.8767357	8.4137396	9.1170625	13.807795	11.80899	12.531986	18.49941	20.591889	21.260176	-0.079335	3.6805457	0.4478396	0.8526004	0.4510573	3.6805457	7.05E-06	5.22E-05	1.113556	3.6805457	2.83E-29	3.09E-28
15755	ENSMUSG00	14797	Tle5	1	protein codi	MGI Symbol	10	TLE family m	10028	9904	9450	10615	9928	10238	18341	19906	16334	24172	29006	26217	178.17368	172.03866	167.99533	174.49361	162.51285	179.50093	249.26059	242.09038	237.74385	368.64612	391.92147	376.35248	-0.004765	7.9178416	0.958465	1	0.4925774	7.9178416	5.06E-08	5.63E-07	1.1335318	7.9178416	1.76E-35	2.48E-34
15769	ENSMUSG00	328417	Parp4	1	protein codi	MGI Symbol	2	poly (ADP-ri	359	314	330	345	447.99	397	715	935	815.99	1224	1402	1360.99	6.3785749	5.4543759	5.8665035	5.6712478	7.3332125	6.9605266	9.7170995	11.37117	11.876859	18.667171	18.943457	19.537398	0.1736146	3.427559	0.1132016	0.5268244	0.8971355	3.427559	7.17E-18	2.06E-16	1.6909945	3.427559	1.74E-61	4.97E-60
1578	ENSMUSG00	11303	Abca1	1	protein codi	MGI Symbol	2	ATP-binding	495	360	444	313	413	357	497	726	672	1567	1730	1889	8.794971	6.2534245	7.8931138	5.145219	6.7604561	6.2592141	6.7544034	8.8293788	9.7810622	23.898249	23.375307	27.117131	-0.336399	3.5653378	0.0017918	0.0332276	0.144755	3.5653378	0.1644882	0.2414086	1.6974203	3.5653378	1.22E-64	3.65E-63
15795	ENSMUSG00	20541	Slc8a1	1	protein codi	MGI Symbol	6	solute carrie	800	586	743	706	790	696	1545	1882	1536	3785	5239	4775	14.214095	10.179186	13.208522	11.60551	12.931623	12.202838	20.997089	22.888279	22.356714	57.72487	70.787995	68.546481	-0.033001	4.8221786	0.7441802	0.9554955	0.817392	4.8221786	4.12E-17	1.10E-15	2.3901861	4.8221786	6.50E-132	6.30E-130

Table S2. Differentially expressed genes (> 2-fold) in *Trpm3*-mutant lenses summarized in Fig. 9A.

15969	ENSMUSG00	16423	Cd47	1	protein codi	MGI Symbol	10	CD47 antige	1171	1077	1116	1216	1324	1215	1995	2185	1681	3095	3997	3512	20.805881	18.708162	19.839448	19.989094	21.672746	21.302367	27.112746	26.573268	24.467211	47.20171	54.006417	50.415757	0.0852199	4.8797048	0.3766114	0.8134757	0.3972731	4.8797048	2.91E-05	0.0001863	1.3532669	4.8797048	3.50E-46	6.89E-45
15983	ENSMUSG00	71918	Zcchc24	1	protein codi	MGI Symbol	2	zinc finger, C	523	564	571	543	527	589	821	887	672	1256	1678	1489	9.2924643	9.7970318	10.150829	8.9260509	8.6265385	10.326827	11.157676	10.787409	9.7810622	19.155201	22.672696	21.375018	-0.069614	3.6721868	0.5056596	0.8738887	0.1183266	3.6721868	0.2446702	0.3320353	1.1123773	3.6721868	1.99E-29	2.19E-28
16009	ENSMUSG00	74901	Kbtbd11	1	protein codi	MGI Symbol	4	kelch repeat	107.14	112.06	76.61	70.72	66.41	84.8	320.89	376.71	262.13	561.62	854.54	646.44	1.9036226	1.9465521	1.3619177	1.1625236	1.0870748	1.4867825	4.3610071	4.581426	3.815342	8.5652421	11.546321	9.2798298	-0.48475	2.134121	0.0022784	0.0406853	1.2922853	2.134121	1.63E-23	6.28E-22	2.4950234	2.134121	2.89E-92	1.37E-90
16108	ENSMUSG00	20454	St3gal5	1	protein codi	MGI Symbol	4	ST3 beta-gal	402	385	383	436	420	417	696	813	656	1012	1027	987	7.1425825	6.6876901	6.8086995	7.1671421	6.8750402	7.3111828	9.4588829	9.8874448	9.5481798	15.433968	13.876555	14.168665	0.0489045	3.2646316	0.6549237	0.9286055	0.4857277	3.2646316	2.89E-06	2.35E-05	1.0743684	3.2646316	6.19E-26	5.73E-25
16113	ENSMUSG00	56386	B4galt6	1	protein codi	MGI Symbol	1	UDP-Gal:bet	161	157	144	170	166	156	374	455	368	572	694	583	2.8605865	2.7271879	2.5599288	2.7945279	2.7172778	2.7351188	5.0827905	5.5335638	5.356296	8.723547	9.3771461	8.3691306	0.0174853	2.3238043	0.9070011	0.9905825	0.9711768	2.3238043	2.97E-16	7.53E-15	1.6995927	2.3238043	6.84E-49	1.44E-47
16119	ENSMUSG00	NA	AI504432	1	lincRNA	MGI Symbol	1	expressed se	145	108	187	99	141	109	357	449	292	495	634	685	2.5763046	1.8760274	3.324352	1.6274015	2.3080492	1.9110766	4.8517546	5.4605937	4.2501044	7.5492234	8.5664419	9.8333695	-0.408935	2.2083703	0.0031442	0.0526814	0.9096594	2.2083703	4.74E-14	1.01E-12	1.740125	2.2083703	1.74E-50	3.82E-49
16174	ENSMUSG00	16881	Lig1	1	protein codi	MGI Symbol	12	ligase I, DNA	309	261	293	291	318	323	494	509	461	768	889	835	5.490194	4.5337328	5.208744	4.7835742	5.2053875	5.6630985	6.7136324	6.1902945	6.7099251	11.712734	12.011935	11.986662	0.0386548	2.860067	0.7426279	0.9548821	0.3640607	2.860067	0.0008403	0.0034086	1.2294568	2.860067	1.47E-31	1.78E-30
1618	ENSMUSG00	16398	Itga2	1	protein codi	MGI Symbol	2	integrin alph	886	736	873	1129	1249	1064	1312	1669	1580	1988	2312	2464	15.74211	12.784779	15.519568	18.558953	20.44506	18.654913	17.830538	20.297842	22.99714	30.318901	31.239138	35.37142	0.3888489	4.4412518	6.61E-05	0.0018921	0.4724695	4.4412518	1.00E-06	8.99E-06	1.1381111	4.4412518	2.40E-32	2.96E-31
16217	ENSMUSG00	320365	Fry	1	protein codi	MGI Symbol	18	FRY microtul	510	379	489	445	521	596	768	1126	958	1822	2021	2058	9.0614853	6.5834664	8.6930916	7.3150877	8.5283236	10.449556	10.437388	13.69405	13.943836	27.787242	27.307222	29.543174	0.1104932	3.8634799	0.2913498	0.7545748	0.6461969	3.8634799	1.78E-10	2.66E-09	1.7983625	3.8634799	5.07E-73	1.78E-71
16222	ENSMUSG00	14227	Fkbp2	1	protein codi	MGI Symbol	4	FK506 bindi	276	274	257	347	342	341	482	469	429	653	888	920	4.9038626	4.7595509	4.5687618	5.7041246	5.598247	5.9786891	6.5505482	5.7038273	6.2441603	9.9588745	11.998423	13.206861	0.2794887	2.8426095	0.0133219	0.1530324	0.3769131	2.8426095	0.0006168	0.0026418	1.3054714	2.8426095	5.62E-35	7.77E-34
16223	ENSMUSG00	114841036	Fkbp2	1	protein codi	MGI Symbol	4	FK506 bindi	276	274	257	347	342	341	482	469	429	653	888	920	4.9038626	4.7595509	4.5687618	5.7041246	5.598247	5.9786891	6.5505482	5.7038273	6.2441603	9.9588745	11.998423	13.206861	0.2794887	2.8426095	0.0133219	0.1530324	0.3769131	2.8426095	0.0006168	0.0026418	1.3054714	2.8426095	5.62E-35	7.77E-34
16248	ENSMUSG00	18030	Nfil3	1	protein codi	MGI Symbol	1	nuclear fact	234	192	171	208	180	190	363	389	344	693	918	799	4.1576227	3.3351598	3.0399155	3.4191871	2.9464458	3.3312344	4.9332967	4.730893	5.0069723	10.568913	12.403775	11.469872	-0.119506	2.5544437	0.3480396	0.7940385	0.4773152	2.5544437	3.96E-05	0.0002431	1.7098166	2.5544437	8.35E-54	2.00E-52
16262	ENSMUSG00	53424	Tsnax	1	protein codi	MGI Symbol	2	translin-asso	1887	1756.94	1920	1981.95	1989	2017.94	3290	3586	2859.94	3955	5267	5182.87	33.527496	30.519144	34.132384	32.580086	32.558226	35.380164	44.712248	43.61178	41.626862	60.317532	71.166324	74.401571	0.0338909	5.4801424	0.7187616	0.9477493	0.4046086	5.4801424	1.34E-05	9.35E-05	1.0685358	5.4801424	1.77E-30	2.06E-29
1627	ENSMUSG00	14462	Gata3	1	protein codi	MGI Symbol	6	GATA bindin	654	566	512	698	671	746	1419.96	1560	1137	1841.94	2003.94	1790.99	11.620022	9.831773	9.1019691	11.474003	10.983695	13.079478	19.297752	18.972219	16.549208	28.091346	27.076712	25.71017	0.2173476	4.0809591	0.0318086	0.2753708	0.8438777	4.0809591	1.22E-17	3.39E-16	1.4042119	4.0809591	1.94E-46	3.84E-45
1629	ENSMUSG00	16776	Lama5	1	protein codi	MGI Symbol	4	laminin, alph	3877	2596	3937	3507	4309	3888	4963	6500	6201	9064	9971	11570	68.885056	45.094139	69.989165	57.649467	70.534638	68.167575	67.448902	79.050912	90.256498	138.23467	134.72554	166.09064	0.094068	6.4610853	0.3057083	0.7649214	0.3639338	6.4610853	6.90E-05	0.0003963	1.2550312	6.4610853	3.51E-42	6.20E-41
16321	ENSMUSG00	67475	Ero1lb	1	protein codi	MGI Symbol	4	ERO1-like be	492	507	404	530.96	556	541	777	910.8	728	1012.94	1342.97	1225.96	8.7416682	8.8069062	7.1820225	8.7281325	9.1012436	9.4852516	10.559701	11.076857	10.596151	15.448304	18.145859	17.599004	0.1428168	3.5068744	0.171106	0.6295534	0.3823104	3.5068744	0.0001778	0.0009012	1.0498531	3.5068744	1.19E-25	1.08E-24
16382	ENSMUSG00	545389	Cep170	1	protein codi	MGI Symbol	15	centrosomal	382	373	345	469	422	456	596	689	518	799	966	1117	6.7872302	6.4792427	6.1331628	7.7096093	6.9077784	7.9949625	8.099848	8.3793966	7.5395688	12.185514	13.052339	16.034852	0.2204297	3.1719904	0.040213	0.3175663	0.309031	3.1719904	0.0034483	0.0108221	1.0892093	3.1719904	2.71E-26	2.58E-25
164	ENSMUSG00	17387	Mmp14	1	protein codi	MGI Symbol	7	matrix meta	601	489	691	448	387	482	833	889	682	1311	2051	1814	10.678339	8.494235	12.284103	7.3644029	6.3348584	8.4508156	11.320761	10.811732	9.9266137	19.994004	27.712575	26.040485	-0.506808	3.7415941	1.09E-06	4.75E-05	0.028377	3.7415941	0.7862561	0.8363491	1.2303799	3.7415941	4.51E-36	6.47E-35
16424	ENSMUSG00	230857	Ece1	1	protein codi	MGI Symbol	4	endothelin c	3141	2844	2989	3163	3305	3185	6784	7266	6267	9316	11582	11234	55.808089	49.402054	53.1363	51.994657	54.100018	55.842008	92.196927	88.366758	91.217138	142.07791	156.49295	161.26726	0.0323271	6.4558945	0.7266401	0.9504507	0.7793381	6.4558945	2.17E-17	5.89E-16	1.5380885	6.4558945	1.47E-61	4.21E-60
1645	ENSMUSG00	67220	Plekho1	1	protein codi	MGI Symbol	5	pleckstrin ho	243	205	252	219	234	215	297	327	290	513	620	629	4.3175312	3.5609779	4.4798754	3.6000095	3.8303795	3.7695547	4.0363336	3.9768689	4.2209941	7.8237406	8.3772775	9.0294736	-0.141394	2.3632474	0.2413967	0.72048	-0.014827	2.3632474	0.9001389	0.9275756	1.0304056	2.3632474	9.35E-21	6.38E-20
16450	ENSMUSG00	14433	Gapdh	1	protein codi	MGI Symbol	13	glyceraldehy	20139.76	20354.42	19664.21	23078.93	23950.92	23321.74	34420.34	34612.96	28111.05	42395.63	59880.91	65022.34	357.83557	353.56897	349.57623	379.38067	392.05604	408.8957	467.78443	420.9517	409.16061	646.5739	809.09516	933.41416	0.1537847	8.9487028	0.0873943	0.4697071	0.2907783	8.9487028	0.0012298	0.0046418	1.1710731	8.9487028	6.85E-38	1.06E-36
16507	ENSMUSG00	217837	Itpk1	1	protein codi	MGI Symbol	5	inositol 1,3,4	2476	2494	2801	3180	3239	3097	5246	6055	5203	6709	7603	6835	43.992623	43.322336	49.794171	52.27411	53.019655	54.29912	71.29497	73.638965	75.730456	102.31867	102.72974	98.118366	0.219065	6.0975631	0.0176951	0.185469	0.6865139	6.0975631	8.92E-14	1.85E-12	1.1447919	6.0975631	3.48E-35	4.86E-34
16534	ENSMUSG00	66052	Sdhc	1	protein codi	MGI Symbol	3	succinate de	655	634	620	729	764	763	1258	1419	1163	1482	1960	1756	11.63779	11.012975	11.021916	11.983593	12.506025	13.377536	17.096659	17.257422	16.927642	22.601917	26.483006	25.207879	0.1690277	4.0460682	0.0926051	0.4799502	0.6069237	4.0460682	6.75E-10	9.56E-09	1.1419645	4.0460682	1.47E-31	1.78E-30
16546	ENSMUSG00	233332	Adamts17	1	protein codi	MGI Symbol	8	a disintegrin	230	184	205	236	230	203	1432	1450	1415	3973	4321	4719	4.0865522	3.1961948	3.6443431	3.8794623	3.7649029	3.5591609	19.46138	17.634434	20.59554	60.592049	58.38422	67.742585	0.0372725	4.4863837	0.7657596	0.9607648	2.3997185	4.4863837	2.50E-107	6.98E-105	4.0947189	4.4863837	2.50E-296	1.25E-293
16573	ENSMUSG00	21912	Tspan7	1	protein codi	MGI Symbol	3	tetraspanin 7	1353	1408	1450	1355	1320	1329	3854	3959	3328	5439	6717	5701	24.039587	24.457838	25.777061	22.274031	21.607269	23.301108	52.377205	48.148086	48.439546	82.949951	90.758344	81.839474	-0.144908	5.5123292	0.1285962	0.5536637	1.0039613	5.5123292	1.01E-26	4.39E-25	1.782663	5.5123292	4.96E-79	1.89E-77
1661	ENSMUSG00	18412	Sqstm1	1	protein codi	MGI Symbol	7	sequestoson	6524.13	6432.04	7847.58	8417.24	8237.25	8233.47	22217.81	21322.41	19759.67	28507.58	37052.93	30847.21	115.91825	111.72855	139.50865	138.36595	134.83672	144.35589	301.94779	259.3163	287.605	434.76786	500.64948	442.82046	0.1855832	7.9723405	0.0406512	0.3184001	1.2091227	7.9723405	7.13E-40	5.25E-38	1.9083909	7.9723405	2.54E-93	1.22E-91
16643	ENSMUSG00	50875	Tmod3	1	protein codi	MGI Symbol	6	tropomoduli	175	143	149	125	144	142	286	238	239	451	624	651	3.1093332	2.4839992	2.6488152	2.0547999	2.3571566	2.4896594	3.8868398	2.8944795	3.4786814	6.8781813	8.4313245	9.3452899	-0.256715	2.0889421	0.056006	0.3776131	0.3127604	2.0889421	0.0114024	0.0285435	1.581918	2.0889421	1.94E-42	3.44E-41
16670	ENSMUSG00	72972	Ccser2	1	protein codi	MGI Symbol	10	coiled-coil se	399	367	418	378	412	370	617	709	658	883	1062	1045	7.0892797	6.3750189	7.4309044	6.213715	6.744087	6.4871406	8.3852453	8.6226302	9.5772901	13.466594	14.349466	15.001272	-0.103441	3.2052537	0.3413303	0.7880077	0.3467907	3.2052537	0.0008729	0.0035182	1.0353703	3.2052537	3.38E-24	2.80E-23
16729	ENSMUSG00	16502	Kcnc1	1	protein codi	MGI Symbol	3	potassium vo	108	110	138	175	208	159	307	289	259	803	706	722	1.9189028	1.9107686	2.4532651	2.8767199	3.4047818	2.7877172	4.1722371	3.5147252	3.7697844	12.246518	9.539287	10.364515	0.5309809	2.3245115	7.38E-05	0.0020673	0.8652582	2.3245115	8.70E-12	1.53E-10	2.3534296	2.3245115	3.41E-87	1.45E-85
16744	ENSMUSG00	433182	Eno1b	1	protein codi	MGI Symbol	2	enolase 1B, r	6702.26	6809.56	11667.39	5314.71	5674.91	5385.41	21141.46	18714.02	8767.15	23720.53	32728.6	31511.27	119.0832	118.2863	207.4145	87.365326	92.893415	94.421384	287.31982	227.5939	127.6072	361.76077	442.22027	452.35323	-0.695322	7.7704183	2.34E-14	2.53E-12	0.5308632	7.7704183	4.52E-09	5.77E-08	1.4981108	7.7704183	1.25E-59	3.39E-58
16778	ENSMUSG00	NA	Gm12715	1	processed p	MGI Symbol	1	predicted ge	3203.06	3165.12	3187.82	4219.2	4112.87	3835.78	6507.64	7635.32	6220.6	8196.63	9373.89	7928.75	56.910747	54.980109	56.670779	69.356895	67.324158	67.252012	88.441098	92.858309	90.541779	125.00645	126.65755	113.81946	0.2748349	6.396619	0.002762	0.0473631	0.6895138	6.396619	5.49E-14	1.16E-12	1.1165214	6.396619	8.35E-34	1.10E-32
16791	ENSMUSG00	53860	Septin9	1	protein codi	MGI Symbol	10	septin 9 [Sou	612	576	675	818	782	761	1496	1651	1512	2925	3635	3504	10.873782	10.005479	11.999666	13.446611	12.80067	13.34247	20.331162	20.078932	22.00739	44.609047	49.115167	50.300915	0.2680537	4.5469814	0.0075775	0.1029845	0.9244999	4.5469814	3.85E-21	1.35E-19	2.1312318	4.5469814	5.43E-105	3.20E-103
1682	ENSMUSG00	11566	Adss	1	protein codi	MGI Symbol	3	adenylosucc	457	426	498	460	533	450	797	834	785	1020	1161	1266	8.1198015	7.3998857	8.8530871	7.5616637	8.7247533	7.8897656	10.831508	10.14284	11.425794	15.555975	15.687128	18.17379	-0.011194	3.4518319	0.9207707	0.9921337	0.4100084	3.4518319	5.81E-05	0.0003418	1.0198654	3.4518319	3.37E-24	2.80E-23
16901	ENSMUSG00	53902	Rcan3	1	protein codi	MGI Symbol	3	regulator of	316	284	322	347	334	310	545	642	500	900	1220	1157	5.6145674	4.9332571	5.7242852	5.7041246	5.4672938	5.4351719	7.4067402	7.8077977	7.277576	13.725861	16.48432	16.609064	0.029887	3.1063926	0.8006742	0.9701843	0.4679813	3.1063926	1.33E-05	9.29E-05	1.5254276	3.1063926	3.31E-49	7.03E-48
16917	ENSMUSG00	50780	Rgs3	1	protein codi	MGI Symbol	17	regulator of	306.82	249.13	274.69	331.93	281.48	310.75	448.6	434.89	380.47	716.59	909.67	934.12	5.4514606	4.3275435	4.883242	5.4563979	4.6075864	5.4483215	6.0966305	5.2889925	5.5377987	10.928683	12.291223	13.409558	0.0810711	2.8179518	0.4756684	0.864464	0.2065432	2.8179518	0.0627097	0.1119816	1.3215847	2.8179518	4.35E-36	6.25E-35
16947	ENSMUSG00	19245	Ptp4a3	1	protein codi	MGI Symbol	10	protein tyros	2448	2294	2380	3058	2889	2920	5460	6461	5683	9450	11865	10848	43.495129	39.848211	42.309934	50.268625	47.290455	51.195813	74.203305	78.576606	82.716929	144.12154	160.31677	155.72612	0.2434519	6.3393955	0.0085435	0.1116408	0.9062131	6.3393955	1.03E-22	3.83E-21	1.8727368	6.3393955	1.95E-88	8.44E-87
1706	ENSMUSG00	64138	Ctsz	1	protein codi	MGI Symbol	1	cathepsin Z [	2250	2012	2148	2639	2394	2357	4001	4100	3590	4796	5513	5738	39.977141	34.949695	38.185605	43.380936	39.187729	41.324839	54.374986	49.862883	52.252996	73.143586	74.490211	82.370619	0.1313872	5.7016969	0.1580826	0.6082563	0.4682611	5.7016969	4.14E-07	4.00E-06	1.023943	5.7016969	2.45E-28	2.57E-27
17089	ENSMUSG00	29820	Tnfrsf19	1	protein codi	MGI Symbol	5	tumor necro	288	249	303	320	307	304	429	477	430	648	719	718	5.117074	4.3252853	5.3865169	5.2602878	5.025327	5.329975	5.8302597	5.8011208	6.2587154	9.8826197	9.7149396	10.307094	0.0747812	2.7191287	0.5155521	0.8782226	0.2703171	2.7191287	0.0141772	0.0341057	1.0121024	2.7191287	1.98E-21	1.42E-20
17115	ENSMUSG00	54667	Atp8b2	1	protein codi	MGI Symbol	17	ATPase, clas	263	251	213	261	277	259	397	501	396	571	699	675	4.6728836	4.3600266	3.7865614	4.2904222	4.5342527	4.5409984	5.3953685	6.093001	5.7638402	8.708296	9.4447048	9.6898167	0.0598401	2.588816	0.6129428	0.9147302	0.4290493	2.588816	0.0001328	0.0007038	1.1189961	2.588816	1.14E-24	9.75E-24
1717	ENSMUSG00	102866	Pls3	1	protein codi	MGI Symbol	6	plastin 3 (T-i	481	485	500	413	481	479	896	1075	837	1489	1718	1701	8.5462244	8.4247525	8.8886417	6.7890589	7.8735579	8.3982172	12.176953	13.073805	12.182662	22.708674	23.213166	24.418338	-0.165923	3.7183317	0.1159482	0.5324511	0.5341084	3.7183317	1.24E-07	1.31E-06	1.4435679	3.7183317	6.86E-48	1.39E-46
1724	ENSMUSG00	67533	Ppfibp1	1	protein codi	MGI Symbol	12	PTPRF intera	1665	1531.93	1587	1846	1934	1868	3194	3620.99	3097	4765.99	6151.99	5809.95	29.583084	26.61058	28.212549	30.345285	31.657923	32.751294	43.407574	44.037317	45.077306	72.685905	83.124077	83.403482	0.1667939	5.5242238	0.0763629	0.4386019	0.650792	5.5242238	3.14E-12	5.74E-11	1.5029857	5.5242238	1.38E-57	3.64E-56
17293	ENSMUSG00	215474	Sec22c	1	protein codi	MGI Symbol	7	SEC22 homo	199	207	212	266	284	312	338	324	305	553	625	543	3.535756	3.5957191	3.7687841	4.3726142	4.6488367	5.4702375	4.5935379	3.9403839	4.4393214	8.4337788	8.4448362	7.7949192	0.4091337	2.4086743	0.0004881	0.0109351	0.2491659	2.4086743	0.032775	0.0663744	1.1782232	2.4086743	6.09E-26	5.65E-25
17306	ENSMUSG00	238161	Akap6	1	protein codi	MGI Symbol	2	A kinase (PR	213	159	201	226	228	233	412	525	481	996	1147	1671	3.7845027	2.7619292	3.573234	3.7150783	3.7321647	4.0851453	5.5992238	6.3848813	7.0010282	15.189952	15.497963	23.98768	0.188497	3.0116633	0.120157	0.538237	0.9074355	3.0116633	1.93E-15	4.59E-14	2.4340729	3.0116633	5.60E-108	3.57E-106
17307	ENSMUSG00	240087	Mdc1	1	protein codi	MGI Symbol	3	mediator of	353	261	278	282	327	299	464	552	418	644	851	824	6.2719692	4.5337328	4.9420848	4.6356286	5.3527098	5.242311	6.3059219	6.7132467	6.0840536	9.8216159	11.498489	11.828754	-0.047942	2.8096361	0.6793917	0.936752	0.2802713	2.8096361	0.0103985	0.0265471	1.0748803	2.8096361	1.96E-24	1.65E-23
17334	ENSMUSG00	545156	Kalrn	1	protein codi	MGI Symbol	21	kalirin, RhoG	350	244	273	345	305	324	518	551	594	1519	1553	1477	6.2186664	4.2384322	4.8531984	5.6712478	4.9925887	5.6806313	7.0398007	6.701085	8.6457603	23.166203	20.983729	21.202754	0.0944255	3.330014	0.408828	0.8362231	0.5461103	3.330014	4.00E-07	3.89E-06	2.0936995	3.330014	6.34E-92	2.97E-90
1737	ENSMUSG00	16784	Lamp2	1	protein codi	MGI Symbol	5	lysosomal-as	2455	2234	2527	2610	2874	2551	4180	4608	3776	5531	6865	7240	43.619503	38.805973	44.923195	42.904222	47.044918	44.726205	56.807659	56.041016	54.960254	84.353039	92.758081	103.93226	0.0807741	5.8907427	0.3843469	0.8199988	0.3981111	5.8907427	1.58E-05	0.000108	1.1421053	5.8907427	6.22E-35	8.56E-34
174	ENSMUSG00	20198	S100a4	1	protein codi	MGI Symbol	2	S100 calciun	3551	3903	3734	4043	3989	3670	8607	7868	6237	14364	20095	20773	63.092812	67.797544	66.380376	66.460449	65.296512	64.345422	116.97213	95.688088	90.780484	219.06474	271.51837	298.20231	-0.008564	6.953519	0.926562	0.9936035	0.6212255	6.953519	1.07E-11	1.87E-10	1.9994641	6.953519	7.01E-101	3.90E-99
17410	ENSMUSG00	18655	Pgk1	1	protein codi	MGI Symbol	3	phosphoglyc	5125.19	5459.99	5350	5417.69	5780.35	6018.97	11659.56	12393.61	9940.5	20454.76	32887.95	29499.3	91.062419	94.843432	95.108466	89.058152	94.619377	105.52947	158.45749	150.7271	144.68549	311.95465	444.37336	423.47083	0.0413659	7.5220447	0.6496616	0.9279484	0.6916404	7.5220447	3.00E-14	6.50E-13	2.06986	7.5220447	4.64E-108	2.98E-106
17429	ENSMUSG00	80281	Cttnbp2nl	1	protein codi	MGI Symbol	4	CTTNBP2 N-t	629	636	575	682	668	744	983	1057	980	1527.92	1810	1738	11.175832	11.047717	10.221938	11.210988	10.934588	13.044413	13.359313	12.854894	14.264049	23.302241	24.456246	24.949484	0.1163494	3.9209311	0.2495539	0.724754	0.3183787	3.9209311	0.0013434	0.0050148	1.164026	3.9209311	1.38E-32	1.73E-31
17456	ENSMUSG00	106869	Tnfaip8	1	protein codi	MGI Symbol	13	tumor necro	785.99	843	815.98	1002	983	1038.99	2719.99	2786.95	2607.52	6018.54	7477.87	7988.97	13.96517	14.643436	14.505908	16.471276	16.090868	18.216417	36.965613	33.893991	37.95285	91.788491	101.03902	114.68393	0.2356146	5.4158187	0.0161369	0.1754006	1.3351487	5.4158187	6.85E-44	5.66E-42	2.834253	5.4158187	2.18E-182	3.90E-180
17494	ENSMUSG00	100862446	Ftl1-ps1	1	protein codi	MGI Symbol	2	ferritin light	3539.32	3555.95	3949.66	3503.49	3517.45	3367.79	6668.49	6868.42	5912.41	9763.02	12884.63	9133.23	62.885287	61.769069	70.214225	57.591768	57.577643	59.046831	90.627106	83.531517	86.056027	148.89539	174.09374	131.11011	-0.161618	6.4980766	0.0783798	0.4456065	0.4171725	6.4980766	4.96E-06	3.82E-05	1.2205823	6.4980766	4.25E-40	7.01E-39
17589	ENSMUSG00	16504	Kcnc3	1	protein codi	MGI Symbol	7	potassium vo	512.79	392.94	378.87	362.85	440.94	375.09	629.15	646.97	677.03	1166.16	1135.91	1084.85	9.1110569	6.8256129	6.7352793	5.9646732	7.21781	6.5763827	8.550368	7.8682413	9.8542746	17.785055	15.348118	15.57333	-0.197981	3.3011621	0.065112	0.401144	0.2109101	3.3011621	0.0414924	0.0801174	1.1027145	3.3011621	9.27E-28	9.46E-27
17597	ENSMUSG00	11465	Actg1	1	protein codi	MGI Symbol	9	actin, gamm	33582.73	32708.27	33148.63	41143.33	40962.38	41250.01	69875.82	73401.89	62678.21	77367.2	91984.56	85653.49	596.68513	568.16305	589.29259	676.33049	670.51906	723.22869	949.63678	892.6902	912.29088	1179.9238	1242.8713	1229.5802	0.238915	9.7358917	0.0078331	0.1048568	0.6510845	9.7358917	5.04E-13	9.95E-12	1.0580726	9.7358917	1.94E-31	2.34E-30
17604	ENSMUSG00	215789	Phactr2	1	protein codi	MGI Symbol	5	phosphatase	487	484	514	590	637	552	757	955	781	1046	1286	1377	8.6528301	8.4073819	9.1375236	9.6986556	10.427144	9.6781125	10.287894	11.614403	11.367574	15.9525	17.376095	19.767226	0.1863289	3.5774944	0.0718091	0.4237182	0.3450927	3.5774944	0.0006763	0.0028543	1.019396	3.5774944	1.77E-24	1.49E-23
17682	ENSMUSG00	16828	Ldha	1	protein codi	MGI Symbol	15	lactate dehy	5375	5410.91	5450.99	5842.99	5781	6238.99	9405	9678.99	6788.97	14296.98	19998.97	19247.99	95.500948	93.990882	96.903794	96.049403	94.630017	109.38704	127.81723	117.71277	98.814491	218.04262	270.22084	276.31036	0.0671907	7.1434854	0.4603044	0.8587044	0.2659647	7.1434854	0.0034125	0.0107393	1.4166912	7.1434854	2.02E-53	4.80E-52
17688	ENSMUSG00	268934	Grm4	1	protein codi	MGI Symbol	8	glutamate re	1768	1789	1366	1831	1773	1755	3641	4207	3081	5069	6338	5018	31.413149	31.076046	24.283769	30.098709	29.022491	30.770086	49.48246	51.164182	44.844424	77.307097	85.637395	72.034815	0.0507711	5.5406913	0.5905784	0.907289	0.7457414	5.5406913	1.28E-15	3.10E-14	1.4371663	5.5406913	5.50E-53	1.29E-51
177	ENSMUSG00	20200	S100a6	1	protein codi	MGI Symbol	4	S100 calciun	4427	5158	5320	6176	6501	5842	11859	11132	8012	17815	30052	27702	78.657246	89.597677	94.575147	101.52355	106.4158	102.42669	161.16795	135.38381	116.61588	271.69579	406.05475	397.67008	0.2398375	7.4259141	0.0084372	0.1107085	0.6526512	7.4259141	7.67E-13	1.50E-11	2.0327424	7.4259141	1.93E-104	1.12E-102
17719	ENSMUSG00	80288	Bcl9l	1	protein codi	MGI Symbol	6	B cell CLL/ly	765	570.72	649.72	716.59	618.57	728	1174.86	1335.19	1041.78	1648.85	1879.47	1790.67	13.592228	9.9137624	11.550257	11.779593	10.125461	12.763888	15.966757	16.238152	15.163266	25.146539	25.394906	25.705576	-0.01639	4.017852	0.8774031	0.9850516	0.4348872	4.017852	1.00E-05	7.21E-05	1.1212434	4.017852	1.26E-30	1.48E-29
17737	ENSMUSG00	319565	Syne2	1	protein codi	MGI Symbol	17	spectrin repe	204	146	253	149	219	236	205	310	310	446	594	715	3.6245941	2.5361111	4.4976527	2.4493215	3.5848424	4.1377438	2.7860215	3.7701204	4.5120972	6.8019265	8.0259723	10.264028	-0.068987	2.2670088	0.5979636	0.909608	0.0534565	2.2670088	0.652694	0.7253853	1.2372439	2.2670088	2.93E-28	3.06E-27
17750	ENSMUSG00	13806	Eno1	1	protein codi	MGI Symbol	11	enolase 1, al	18925.54	16661.6	11651.09	22750.05	23004.07	23296.74	17203.04	23467.61	25881.87	37069.6	45604.15	42106.59	336.26177	289.42238	207.12473	373.97441	376.55692	408.45738	233.79532	285.40553	376.71455	565.34685	616.19132	604.45206	0.4767865	8.6056094	1.25E-07	6.30E-06	0.1052834	8.6056094	0.2419999	0.3288983	1.100666	8.6056094	1.02E-33	1.34E-32
17819	ENSMUSG00	236266	Alms1	1	protein codi	MGI Symbol	5	ALMS1, cent	231.28	190.34	219.04	223.14	276.47	249.24	366.95	436.69	433.71	519.59	572.36	549.11	4.1092947	3.3063245	3.8939361	3.6680644	4.525577	4.3698782	4.9869785	5.3108835	6.312715	7.9242444	7.7335783	7.88263	0.1517318	2.434499	0.2063716	0.678619	0.5529035	2.434499	1.15E-06	1.01E-05	1.0576384	2.434499	3.08E-21	2.17E-20
17843	ENSMUSG00	12916	Crem	1	protein codi	MGI Symbol	39	cAMP respo	137.9	104	113	144.88	153.62	163.87	248.92	261.77	230.97	334.78	350.38	347.67	2.4501546	1.8065449	2.008833	2.3815953	2.5146278	2.873102	3.3829097	3.1835626	3.3618035	5.1057152	4.7342427	4.9909016	0.3091086	1.7221393	0.0225064	0.218844	0.6634402	1.7221393	2.88E-07	2.85E-06	1.2424395	1.7221393	2.24E-23	1.79E-22
17887	ENSMUSG00	94219	Cnnm2	1	protein codi	MGI Symbol	4	cyclin M2 [S	382	388	359	463	405	383	524	668	495	855	1050	1019	6.7872302	6.739802	6.3820447	7.6109789	6.629503	6.7150672	7.1213428	8.1240014	7.2048003	13.039568	14.187325	14.628034	0.0741495	3.1432571	0.4978014	0.8711141	0.1745489	3.1432571	0.1009425	0.163599	1.0720556	3.1432571	1.24E-25	1.12E-24
17909	ENSMUSG00	NA	Gm4735	1	processed p	MGI Symbol	1	predicted ge	3082.08	3170.6	6383.61	588.21	601.02	614.84	11750.84	10160.41	4070.4	12906.07	18698.55	15980.06	54.761221	55.0753	113.48324	9.6692309	9.8381825	10.779874	159.69802	123.56764	59.245291	196.82991	252.64991	229.3983	-2.882428	6.7337648	1.00E-184	1.06E-181	0.6175575	6.7337648	1.31E-11	2.25E-10	1.6042104	6.7337648	4.03E-67	1.25E-65
1811	ENSMUSG00	21858	Timp2	1	protein codi	MGI Symbol	2	tissue inhibit	3966	3825	4181	4042	4014	3921	9405	10453	8196	14016	19026	17479	70.466374	66.442636	74.326822	66.44401	65.705741	68.746158	127.81723	127.12603	119.29403	213.7574	257.07433	250.91601	-0.072414	6.9752156	0.42952	0.8463211	0.8251532	6.9752156	1.86E-19	5.87E-18	1.7726914	6.9752156	8.60E-81	3.39E-79
1815	ENSMUSG00	16010	Igfbp4	1	protein codi	MGI Symbol	6	insulin-like g	118	132	121	141	104	151	359	375	387	1757	2675	2054	2.096579	2.2929223	2.1510513	2.3178143	1.7023909	2.6474547	4.8789353	4.5606295	5.6328439	26.79593	36.143899	29.485753	0.0237887	3.3545522	0.8695409	0.9837607	1.2005639	3.3545522	1.39E-22	5.13E-21	3.8199195	3.3545522	2.52E-235	7.73E-233
1835	ENSMUSG00	26943	Serinc3	1	protein codi	MGI Symbol	2	serine incorp	1471	1367.99	1504.98	1387.97	1507.98	1597.98	2284.88	2637.91	2231.99	3311.98	4032.92	3936.9	26.136166	23.76284	26.754456	22.816005	24.684341	28.017084	31.052316	32.081414	32.486954	50.510862	54.491758	56.515317	-0.021827	5.0957116	0.8215464	0.9753425	0.3190106	5.0957116	0.0006814	0.0028683	1.0753918	5.0957116	2.05E-30	2.37E-29
1853	ENSMUSG00	20498	Slc12a4	1	protein codi	MGI Symbol	5	solute carrie	364.89	292.89	306.53	359.97	325.97	319.97	593.95	701.93	578.89	856.96	960.97	865.98	6.4832262	5.087682	5.4492707	5.9173306	5.3358496	5.609974	8.0719877	8.5366471	8.425832	13.06946	12.984375	12.431389	-0.012992	3.0362449	0.9156815	0.9914851	0.5570921	3.0362449	1.57E-07	1.63E-06	1.177132	3.0362449	1.00E-29	1.12E-28
1855	ENSMUSG00	17913	Myo1c	1	protein codi	MGI Symbol	10	myosin IC [S	232	180	168	212	198	203	545	581	484	1381	1682	1619	4.1220874	3.1267123	2.9865836	3.4849407	3.2410904	3.5591609	7.4067402	7.0659353	7.0446936	21.061571	22.726743	23.241205	0.0068193	3.2063628	0.9685033	1	1.0718857	3.2063628	2.63E-21	9.22E-20	2.7111561	3.2063628	5.90E-134	6.03E-132
1872	ENSMUSG00	56336	B4galt5	1	protein codi	MGI Symbol	4	UDP-Gal:bet	492	431	436	543	528	565	740	762	718	1058	1256	1146	8.7416682	7.4867388	7.7508955	8.9260509	8.6429076	9.9060391	10.056858	9.2671992	10.450599	16.135512	16.970743	16.451155	0.195828	3.4551797	0.0607619	0.3926437	0.3114214	3.4551797	0.0023862	0.0079681	1.0475066	3.4551797	2.14E-25	1.91E-24
1887	ENSMUSG00	17135	Mafk	1	protein codi	MGI Symbol	3	v-maf muscu	269.99	236	222.87	255	218.97	278	422	516.99	358	530.96	701.97	647.97	4.7970792	4.0994672	3.9620231	4.1917918	3.5843513	4.8741219	5.7351272	6.2874663	5.2107445	8.0976478	9.4848347	9.3017934	-0.025461	2.5557266	0.8415598	0.979397	0.4248176	2.5557266	0.0001572	0.0008128	1.0648546	2.5557266	2.26E-22	1.71E-21
18969	ENSMUSG00	77739	Adamtsl1	1	protein codi	MGI Symbol	12	ADAMTS-like	83	72	65	89	83	77	299	427	383	708	799	771	1.4747123	1.2506849	1.1555234	1.4630175	1.3586389	1.3500266	4.0635143	5.1930368	5.5746233	10.797677	10.795879	11.067924	0.1048726	2.2534351	0.5398624	0.8878195	1.9323124	2.2534351	3.03E-47	2.77E-45	3.0714127	2.2534351	5.53E-128	5.01E-126
1906	ENSMUSG00	18643	Pfn1	1	protein codi	MGI Symbol	4	profilin 1 [Sc	10559	10137	11294	10771	11128	10828	20213	23711	18759	24833	30214	27639	187.60828	176.08601	200.77664	177.058	182.15583	189.84529	274.70172	288.36556	273.0401	378.727	408.24365	396.76569	-0.039944	8.0291185	0.6586084	0.9304071	0.5668161	8.0291185	3.61E-10	5.23E-09	1.0683937	8.0291185	8.27E-32	1.01E-30
19060	ENSMUSG00	233103	4931406P16	1	protein codi	MGI Symbol	10	RIKEN cDNA	522.46	519.2	515.63	601.15	648.58	623.17	826.72	1060.17	885.69	1218.68	1464.53	1451.19	9.2828698	9.0188278	9.1665006	9.8819438	10.616699	10.925923	11.235413	12.893447	12.891353	18.586035	19.788346	20.832245	0.193843	3.701152	0.0590977	0.3878421	0.4306417	3.701152	1.89E-05	0.0001265	1.1080725	3.701152	6.16E-29	6.62E-28
1907	ENSMUSG00	67145	Tomm34	1	protein codi	MGI Symbol	3	translocase o	495	448	398	448	442	474	687	717	592	1026	1202	1096	8.794971	7.7820394	7.0753588	7.3644029	7.2351613	8.3105531	9.3365697	8.7199236	8.61665	15.647481	16.241109	15.733391	-0.046742	3.3422057	0.6672414	0.9330831	0.1734045	3.3422057	0.0941363	0.1547866	1.0096391	3.3422057	1.29E-23	1.04E-22
19072	ENSMUSG00	NA	Pgk1-rs7	1	processed p	MGI Symbol	1	phosphoglyc	1010.69	973.73	1171.77	1177.87	1127.17	1353.3	2460.44	2333.79	1671.21	4350.74	6437.56	6171.66	17.957554	16.914297	20.830887	19.362297	18.450807	23.727155	33.438238	28.382804	24.324716	66.352946	86.982624	88.595932	0.1432747	5.219056	0.1368796	0.5700718	0.6294678	5.219056	3.61E-11	5.86E-10	2.1190259	5.219056	8.25E-108	5.19E-106
1911	ENSMUSG00	11749	Anxa6	1	protein codi	MGI Symbol	3	annexin A6 [	1892	1765	2058	2106	2072	2114	5256	6201	5078	7424	8772	8361	33.616334	30.659151	36.585649	34.619269	33.916865	37.064366	71.430874	75.41457	73.911062	113.2231	118.52497	120.02453	0.0661916	6.0237735	0.4798754	0.8655476	1.130157	6.0237735	6.15E-34	3.52E-32	1.8023213	6.0237735	9.89E-82	3.91E-80
19119	ENSMUSG00	545370	Hmcn1	1	protein codi	MGI Symbol	6	hemicentin 1	139.44	110.11	160.42	116.14	154.18	116.12	297.53	584.39	578.6	827.52	869.92	979.2	2.4775167	1.9126794	2.8518318	1.9091557	2.5237945	2.0359102	4.0435365	7.1071634	8.421611	12.620471	11.754131	14.056694	-0.160255	2.6107403	0.2410635	0.72048	1.4335729	2.6107403	2.31E-33	1.29E-31	2.4078336	2.6107403	5.27E-96	2.64E-94
19124	ENSMUSG00	NA	Gm10184	1	protein codi	MGI Symbol	1	predicted ps	192.5	92.31	161.83	167.24	173.39	166.92	358.16	300.96	238.75	408.2	414.2	500.33	3.4202665	1.6034823	2.8768978	2.7491579	2.8382457	2.9265771	4.8675194	3.6601788	3.4750426	6.2254404	5.5965619	7.1823792	0.1095856	2.0089959	0.4134098	0.837543	0.6052222	2.0089959	7.90E-07	7.26E-06	1.267153	2.0089959	1.04E-26	1.01E-25
1914	ENSMUSG00	16647	Kpna2	1	protein codi	MGI Symbol	7	karyopherin	339.51	363.2	265.16	388.41	333.31	358.28	677.29	648.99	577.46	845.37	906.34	895.26	6.0322841	6.3090105	4.7138245	6.3848387	5.4559991	6.2816561	9.2046074	7.8928079	8.4050181	12.892701	12.246229	12.851712	0.0866475	3.0540228	0.4359161	0.8487811	0.5791213	3.0540228	4.55E-08	5.10E-07	1.1544892	3.0540228	1.16E-28	1.23E-27
19192	ENSMUSG00	12338	Capn6	1	protein codi	MGI Symbol	2	calpain 6 [Sc	0	7	8	3	9	10	985	1328	972	3776	3883	3196	0	0.1215944	0.1422183	0.0493152	0.1473223	0.1753281	13.386494	16.150709	14.147608	57.587611	52.466078	45.879488	0.4701791	4.0805952	0.3325215	0.7828883	7.3379115	4.0805952	3.78E-305	8.99E-302	9.1712331	4.0805952	0	0
19211	ENSMUSG00	215201	Trmt2b	1	protein codi	MGI Symbol	5	TRM2 tRNA	160	166	163	201	176	180	296	286	256	379	467	443	2.8428189	2.8835235	2.8976972	3.3041183	2.8809692	3.1559063	4.0227433	3.4782401	3.7261189	5.7801124	6.3099816	6.3593908	0.1148376	2.0126212	0.3824326	0.8184442	0.3789404	2.0126212	0.0019302	0.0066925	1.0973249	2.0126212	7.98E-21	5.46E-20
193	ENSMUSG00	330171	Kctd10	1	protein codi	MGI Symbol	7	potassium cl	1016.73	914.97	1060.72	1020.57	1029.86	1035.9	1540.22	1804.2	1491.74	2327.95	2972.13	2764.64	18.06487	15.8936	18.85672	16.776537	16.857926	18.162241	20.932127	21.942101	21.712503	35.503464	40.158642	39.687192	-0.028171	4.5728224	0.7753646	0.962868	0.2904828	4.5728224	0.0024388	0.0081274	1.1272703	4.5728224	2.55E-32	3.14E-31
19377	ENSMUSG00	16852	Lgals1	1	protein codi	MGI Symbol	2	lectin, galact	650	570	626	908	759	851	4397	4214	3444	9442	12203	12019	11.548952	9.9012555	11.128579	14.926067	12.42418	14.920423	59.756764	51.249314	50.127944	143.99953	164.88374	172.53616	0.3755151	5.9068928	0.0001738	0.0044033	2.3062638	5.9068928	4.64E-122	1.63E-119	3.8853147	5.9068928	8.13731607	4.54732369048065e-312
19416	ENSMUSG00	NA	Gm10240	1	processed p	MGI Symbol	1	predicted ge	1441.56	2594.68	533.93	358.04	609.81	412.63	1819.47	1491.95	1003.47	2010.62	2787.75	2413.74	25.613088	45.07121	9.4918249	5.8856045	9.9820672	7.2345644	24.727232	18.144617	14.605658	30.663878	37.667347	34.649923	-1.794954	4.462517	3.60E-72	2.01E-69	-0.480331	4.462517	4.26E-07	4.09E-06	0.3607346	4.462517	0.0001164	0.0001778
19449	ENSMUSG00	66793	Efcab1	1	protein codi	MGI Symbol	4	EF-hand calc	767	840	919	1034	1095	959	1637	1775	1373	1833	2227	2220	13.627763	14.591324	16.337323	16.997305	17.924212	16.813967	22.247401	21.58698	19.984224	27.955003	30.09064	31.86873	0.2156529	4.3868624	0.0275372	0.2485464	0.5186042	4.3868624	7.67E-08	8.39E-07	1.0130211	4.3868624	5.56E-26	5.18E-25
19472	ENSMUSG00	16188	Il3ra	1	protein codi	MGI Symbol	7	interleukin 3	163	128	191	406	359	391	337	345	304	520	661	559	2.8961218	2.2234398	3.3954611	6.6739902	5.8765224	6.8553297	4.5799476	4.1957792	4.4247662	7.9304973	8.9312588	8.0246037	1.1884088	2.4745385	8.52E-24	1.80E-21	0.6325035	2.4745385	1.42E-07	1.49E-06	1.5486632	2.4745385	4.31E-41	7.36E-40
19520	ENSMUSG00	NA	Gm5537	1	processed p	MGI Symbol	1	predicted ge	1202.11	1144.27	1011.24	1407.4	1276.46	1108.73	2299	2619.59	1961.25	3971.29	6586.42	6136.94	21.358632	19.876684	17.9771	23.135403	20.894557	19.439155	31.244212	31.858612	28.546292	60.56597	88.993981	88.097516	0.1004343	5.2405014	0.2975327	0.7595141	0.6304735	5.2405014	2.90E-11	4.77E-10	2.0051107	5.2405014	1.68E-97	8.64E-96
1958	ENSMUSG00	56486	Gabarap	1	protein codi	MGI Symbol	4	gamma-amin	3303	3179	3376	3625	3782	3575	5590	5614	4930	6933	8928	8554	58.686443	55.221213	60.016109	59.589198	61.9081	62.679805	75.970051	68.275664	71.7569	105.73488	120.6328	122.7951	0.0826329	6.2671957	0.3688212	0.8077396	0.3125314	6.2671957	0.000641	0.0027283	1.005525	6.2671957	7.80E-28	7.99E-27
19624	ENSMUSG00	17118	Marcks	1	protein codi	MGI Symbol	1	myristoylate	1736	1259	1273	1656	1691	1563	3030	3544	2894	4509	5636	4640	30.844585	21.869615	22.630482	27.221989	27.680221	27.403786	41.178757	43.100989	42.12261	68.766562	76.152155	66.608518	0.1276354	5.3721288	0.1782991	0.6379818	0.7466126	5.3721288	1.66E-15	3.96E-14	1.4894438	5.3721288	2.87E-56	7.36E-55
1963	ENSMUSG00	13411	Dnah11	1	protein codi	MGI Symbol	4	dynein, axon	181.73	131.78	147.73	146.19	138.33	118.95	319.16	359.03	327.5	461.26	588.41	480.66	3.2289093	2.2891008	2.6262381	2.4031296	2.2643436	2.0855281	4.3374958	4.3664075	4.7668123	7.0346561	7.9504417	6.9000108	-0.267836	2.0973956	0.0472743	0.3473365	0.7252735	2.0973956	1.76E-09	2.37E-08	1.4272549	2.0973956	2.44E-34	3.28E-33
19649	ENSMUSG00	237886	Slfn9	1	protein codi	MGI Symbol	3	schlafen 9 [S	154.94	87	108.93	165	137.97	182.86	268.92	230.42	209	293.6	336.95	325.22	2.7529148	1.5112443	1.9364795	2.7123359	2.2584507	3.2060501	3.6547166	2.802294	3.0420268	4.4776808	4.5527801	4.6686255	0.3974985	1.6743121	0.0035559	0.0576461	0.614609	1.6743121	2.16E-06	1.81E-05	1.1447392	1.6743121	9.71E-20	6.21E-19
19662	ENSMUSG00	66395	Ahnak	1	protein codi	MGI Symbol	5	AHNAK nucle	3966	2633	4026	3382	5107	4314	3741	6394	5367	9738	12157	14066	70.466374	45.736852	71.571343	55.594667	83.597214	75.636553	50.841495	77.761774	78.117501	148.51381	164.2622	201.92142	0.1942587	6.5508865	0.034061	0.2883956	0.1388798	6.5508865	0.1292131	0.1991118	1.4548716	6.5508865	1.09E-55	2.76E-54
19683	ENSMUSG00	NA	Gm12070	1	processed p	MGI Symbol	1	predicted ge	726.35	726.23	480.82	653.43	593.19	643.6	985.31	1097.27	825.55	1396.23	2022.88	1735.13	12.905509	12.615068	8.5476734	10.741343	9.7100121	11.284118	13.390707	13.344645	12.016006	21.293843	27.332624	24.908284	-0.103063	3.8996817	0.3129751	0.7701806	0.1861446	3.8996817	0.0612659	0.1097542	1.110256	3.8996817	5.82E-30	6.59E-29
19692	ENSMUSG00	76900	Ssbp4	1	protein codi	MGI Symbol	15	single strand	188	126	189	175	209	179	322	348	278	783	912	800	3.3403122	2.1886986	3.3599066	2.8767199	3.4211509	3.1383734	4.3760924	4.2322642	4.0463323	11.941499	12.322705	11.484227	0.0877213	2.5003393	0.5049063	0.8736006	0.5113207	2.5003393	2.11E-05	0.00014	2.0086929	2.5003393	1.16E-70	3.92E-69
19697	ENSMUSG00	212326	Fam149a	1	protein codi	MGI Symbol	3	family with s	132	116	112	129	121	131	263	302	266	514	621	563	2.3453256	2.0149924	1.9910557	2.1205535	1.9806663	2.2967984	3.5742618	3.672827	3.8716705	7.8389915	8.3907893	8.0820249	0.0093909	2.0395837	0.9552587	1	0.8068446	2.0395837	2.00E-10	2.97E-09	1.9362863	2.0395837	1.98E-58	5.28E-57
19783	ENSMUSG00	217143	Gpr179	1	protein codi	MGI Symbol	2	G protein-co	230	239	160	297	359	319	320	359	366	627	893	679	4.0865522	4.1515791	2.8443653	4.8822046	5.8765224	5.5929672	4.3489117	4.3660427	5.3271857	9.5623496	12.065982	9.7472378	0.5600247	2.6185786	1.43E-06	6.05E-05	0.3379789	2.6185786	0.0034099	0.0107393	1.5015533	2.6185786	4.13E-42	7.28E-41
19807	ENSMUSG00	69186	Tmem256	1	protein codi	MGI Symbol	5	transmembr	635	735	726	779	817	717	1202	1292	1188	1370	1913	1965	11.282438	12.767408	12.906308	12.805513	13.37359	12.571027	16.335599	15.712889	17.291521	20.89381	25.847955	28.208133	0.0684653	4.0661142	0.4962296	0.8711141	0.416309	4.0661142	2.13E-05	0.0001411	1.0204488	4.0661142	8.83E-26	8.06E-25
19826	ENSMUSG00	80889	Tlnrd1	1	protein codi	MGI Symbol	1	talin rod dor	316	283	272	320	323	310	607	702	607	936	1048	924	5.6145674	4.9158865	4.8354211	5.2602878	5.2872333	5.4351719	8.2493418	8.5374985	8.8349773	14.274895	14.160301	13.264282	0.0566879	3.0566344	0.6213905	0.9178486	0.7373327	3.0566344	5.16E-12	9.22E-11	1.4400648	3.0566344	2.48E-43	4.54E-42
19841	ENSMUSG00	66647	Nsmce3	1	protein codi	MGI Symbol	1	NSE3 homol	465	378	451	415	466	440	644	745	589	1016	1311	1064	8.2619425	6.5660958	8.0175548	6.8219357	7.6280207	7.7144375	8.7521847	9.0604507	8.5729846	15.494972	17.713888	15.274022	-0.043598	3.3314092	0.6878927	0.9398213	0.2087255	3.3314092	0.0448272	0.0851206	1.0863206	3.3314092	5.51E-27	5.41E-26
19880	ENSMUSG00	433926	Lrrc8b	1	protein codi	MGI Symbol	1	leucine rich	160	149	252	163	187	149	252	272	246	474	592	530	2.8428189	2.5882229	4.4798754	2.6794591	3.0610298	2.6123891	3.4247679	3.3079766	3.5805674	7.2289533	7.9989489	7.6083005	-0.244319	2.121616	0.0546681	0.3722915	0.057197	2.121616	0.6472955	0.720228	1.2056418	2.121616	2.83E-26	2.69E-25
19906	ENSMUSG00	623230	Tmem200b	1	protein codi	MGI Symbol	2	transmembr	293	247	284	303	295	260	1107	1248	1049	1815	2349	1928	5.2059121	4.2905441	5.0487485	4.980835	4.8288972	4.5585313	15.044516	15.177775	15.268355	27.680486	31.739072	27.676988	-0.016593	3.7682763	0.8921942	0.9874875	1.6451034	3.7682763	3.98E-56	4.72E-54	2.5823865	3.7682763	1.18E-138	1.27E-136
19910	ENSMUSG00	72313	Fryl	1	protein codi	MGI Symbol	20	FRY like tran	5026	3803	4939.92	4925	5974	5615	8839	13073	11029	17425	19689	20141	89.300049	66.060482	87.818358	80.959117	97.789262	98.446742	120.12509	158.98963	160.52877	265.74791	266.03261	289.12977	0.1888937	7.2147225	0.0383193	0.3108739	0.854391	7.2147225	8.38E-21	2.87E-19	1.755256	7.2147225	1.66E-79	6.37E-78
1992	ENSMUSG00	75580	Zbtb4	1	protein codi	MGI Symbol	7	zinc finger a	737	749	629	829	830	834	1493	1699	1359	2071	2583	2318	13.094735	13.010597	11.181911	13.627433	13.586389	14.622366	20.290391	20.662692	19.780452	31.584731	34.900819	33.275548	0.1656669	4.3273721	0.0958316	0.4858058	0.7038113	4.3273721	4.92E-13	9.79E-12	1.4197482	4.3273721	1.42E-48	2.95E-47
19921	ENSMUSG00	434128	Pnmal2	1	protein codi	MGI Symbol	1	PNMA-like 2	777	779	560	767	745	844	1412	1551	1163	1824	2157	1798	13.805439	13.531716	9.9552787	12.608252	12.195012	14.797694	19.189573	18.862764	16.927642	27.817744	29.144819	25.810801	0.0857295	4.1683878	0.3897084	0.8245501	0.5602162	4.1683878	9.56E-09	1.19E-07	1.1500699	4.1683878	2.06E-32	2.55E-31
20	ENSMUSG00	209446	Tfe3	1	protein codi	MGI Symbol	14	transcriptior	586	534	625	638	591	613	964	1076	912	1486	1803	1742	10.411824	9.2759131	11.110802	10.487699	9.6741636	10.747614	13.101096	13.085966	13.274299	22.662921	24.361664	25.006905	0.0051081	3.8599906	0.9659852	1	0.3576778	3.8599906	0.0003376	0.001573	1.2260472	3.8599906	9.66E-36	1.38E-34
20002	ENSMUSG00	16478	Jund	1	protein codi	MGI Symbol	1	jun D proto-	1983.35	1711.06	2206.29	2036.65	1860.98	1845.55	3221.39	3879.95	3282.91	4305.94	5634.01	5084.35	35.239406	29.722179	39.221842	33.479266	30.462648	32.357682	43.779814	47.186705	47.783254	65.669703	76.125267	72.987288	-0.113417	5.5318012	0.2259081	0.706031	0.413464	5.5318012	8.31E-06	6.07E-05	1.0439787	5.5318012	2.89E-29	3.15E-28
20124	ENSMUSG00	381217	Fam189a2	1	protein codi	MGI Symbol	2	family with	[illegible]	[illegible]	[illegible]	[illegible]	[illegible]	[illegible]	[illegible]	[illegible]	[illegible]	[illegible]	[illegible]	[illegible]	[illegible]	[illegible]	[illegible]	[illegible]	[illegible]	[illegible]	[illegible]	[illegible]	[illegible]	[illegible]	[illegible]	[illegible]	[illegible]	[illegible]	[illegible]	[illegible]	[illegible]	[illegible]	[illegible]	[illegible]	[illegible]	[illegible]	[illegible]	[illegible]
20213	ENSMUSG00	18951	Septin5	1	protein codi	MGI Symbol	5	septin 5 [Sou	[illegible]	[illegible]	[illegible]	[illegible]	[illegible]	[illegible]	[illegible]	[illegible]	[illegible]	[illegible]	[illegible]	[illegible]	[illegible]	[illegible]	[illegible]	[illegible]	[illegible]	[illegible]	[illegible]	[illegible]	[illegible]	[illegible]	[illegible]	[illegible]	[illegible]	[illegible]	[illegible]	[illegible]	[illegible]	[illegible]	[illegible]	[illegible]	[illegible]	[illegible]	[illegible]	[illegible]
2024	ENSMUSG00	109689	Arrb1	1	protein codi	MGI Symbol	10	arrestin, bet	[illegible]	[illegible]	[illegible]	[illegible]	[illegible]	[illegible]	[illegible]	[illegible]	[illegible]	[illegible]	[illegible]	[illegible]	[illegible]	[illegible]	[illegible]	[illegible]	[illegible]	[illegible]	[illegible]	[illegible]	[illegible]	[illegible]	[illegible]	[illegible]	[illegible]	[illegible]	[illegible]	[illegible]	[illegible]	[illegible]	[illegible]	[illegible]	[illegible]	[illegible]	[illegible]	[illegible]
20242	ENSMUSG00	[illegible]	Lratd2	1	protein codi	MGI Symbol	2	LRAT domai	[illegible]	[illegible]	[illegible]	[illegible]	[illegible]	[illegible]	[illegible]	[illegible]	[illegible]	[illegible]	[illegible]	[illegible]	[illegible]	[illegible]	[illegible]	[illegible]	[illegible]	[illegible]	[illegible]	[illegible]	[illegible]	[illegible]	[illegible]	[illegible]	[illegible]	[illegible]	[illegible]	[illegible]	[illegible]	[illegible]	[illegible]	[illegible]	[illegible]	[illegible]	[illegible]	[illegible]
20251	ENSMUSG00	100039192	Tmem254c	1	protein codi	MGI Symbol	3	transmembr	[illegible]	[illegible]	[illegible]	[illegible]	[illegible]	[illegible]	[illegible]	[illegible]	[illegible]	[illegible]	[illegible]	[illegible]	[illegible]	[illegible]	[illegible]	[illegible]	[illegible]	[illegible]	[illegible]	[illegible]	[illegible]	[illegible]	[illegible]	[illegible]	[illegible]	[illegible]	[illegible]	[illegible]	[illegible]	[illegible]	[illegible]	[illegible]	[illegible]	[illegible]	[illegible]	[illegible]
20483	ENSMUSG00	14570	Arhgdig	1	protein codi	MGI Symbol	4	Rho GDP dis	[illegible]	[illegible]	[illegible]	[illegible]	[illegible]	[illegible]	[illegible]	[illegible]	[illegible]	[illegible]	[illegible]	[illegible]	[illegible]	[illegible]	[illegible]	[illegible]	[illegible]	[illegible]	[illegible]	[illegible]	[illegible]	[illegible]	[illegible]	[illegible]	[illegible]	[illegible]	[illegible]	[illegible]	[illegible]	[illegible]	[illegible]	[illegible]	[illegible]	[illegible]	[illegible]	[illegible]
2051	ENSMUSG00	54411	Atp6ap1	1	protein codi	MGI Symbol	4	ATPase, H+ t	[illegible]	[illegible]	[illegible]	[illegible]	[illegible]	[illegible]	[illegible]	[illegible]	[illegible]	[illegible]	[illegible]	[illegible]	[illegible]	[illegible]	[illegible]	[illegible]	[illegible]	[illegible]	[illegible]	[illegible]	[illegible]	[illegible]	[illegible]	[illegible]	[illegible]	[illegible]	[illegible]	[illegible]	[illegible]	[illegible]	[illegible]	[illegible]	[illegible]	[illegible]	[illegible]	[illegible]
2057	ENSMUSG00	71780	Isyna1	1	protein codi	MGI Symbol	9	myo-inositol	[illegible]	[illegible]	[illegible]	[illegible]	[illegible]	[illegible]	[illegible]	[illegible]	[illegible]	[illegible]	[illegible]	[illegible]	[illegible]	[illegible]	[illegible]	[illegible]	[illegible]	[illegible]	[illegible]	[illegible]	[illegible]	[illegible]	[illegible]	[illegible]	[illegible]	[illegible]	[illegible]	[illegible]	[illegible]	[illegible]	[illegible]	[illegible]	[illegible]	[illegible]	[illegible]	[illegible]
20584	ENSMUSG00	230757	5730409E04	1	protein codi	MGI Symbol	2	RIKEN cDNA	[illegible]	[illegible]	[illegible]	[illegible]	[illegible]	[illegible]	[illegible]	[illegible]	[illegible]	[illegible]	[illegible]	[illegible]	[illegible]	[illegible]	[illegible]	[illegible]	[illegible]	[illegible]	[illegible]	[illegible]	[illegible]	[illegible]	[illegible]	[illegible]	[illegible]	[illegible]	[illegible]	[illegible]	[illegible]	[illegible]	[illegible]	[illegible]	[illegible]	[illegible]	[illegible]	[illegible]
20631	ENSMUSG00	16157	Il11ra1	1	protein codi	MGI Symbol	10	interleukin 1	[illegible]	[illegible]	[illegible]	[illegible]	[illegible]	[illegible]	[illegible]	[illegible]	[illegible]	[illegible]	[illegible]	[illegible]	[illegible]	[illegible]	[illegible]	[illegible]	[illegible]	[illegible]	[illegible]	[illegible]	[illegible]	[illegible]	[illegible]	[illegible]	[illegible]	[illegible]	[illegible]	[illegible]	[illegible]	[illegible]	[illegible]	[illegible]	[illegible]	[illegible]	[illegible]	[illegible]
2079	ENSMUSG00	11975	Atp6v0a1	1	protein codi	MGI Symbol	1	ATPase, H+ t	[illegible]	[illegible]	[illegible]	[illegible]	[illegible]	[illegible]	[illegible]	[illegible]	[illegible]	[illegible]	[illegible]	[illegible]	[illegible]	[illegible]	[illegible]	[illegible]	[illegible]	[illegible]	[illegible]	[illegible]	[illegible]	[illegible]	[illegible]	[illegible]	[illegible]	[illegible]	[illegible]	[illegible]	[illegible]	[illegible]	[illegible]	[illegible]	[illegible]	[illegible]	[illegible]	[illegible]
20817	ENSMUSG00	244895	Peak1	1	protein codi	MGI Symbol	4	pseudopodiu	[illegible]	[illegible]	[illegible]	[illegible]	[illegible]	[illegible]	[illegible]	[illegible]	[illegible]	[illegible]	[illegible]	[illegible]	[illegible]	[illegible]	[illegible]	[illegible]	[illegible]	[illegible]	[illegible]	[illegible]	[illegible]	[illegible]	[illegible]	[illegible]	[illegible]	[illegible]	[illegible]	[illegible]	[illegible]	[illegible]	[illegible]	[illegible]	[illegible]	[illegible]	[illegible]	[illegible]
20892	ENSMUSG00	259300	Ehd2	1	protein codi	MGI Symbol	2	EH-domain c	[illegible]	[illegible]	[illegible]	[illegible]	[illegible]	[illegible]	[illegible]	[illegible]	[illegible]	[illegible]	[illegible]	[illegible]	[illegible]	[illegible]	[illegible]	[illegible]	[illegible]	[illegible]	[illegible]	[illegible]	[illegible]	[illegible]	[illegible]	[illegible]	[illegible]	[illegible]	[illegible]	[illegible]	[illegible]	[illegible]	[illegible]	[illegible]	[illegible]	[illegible]	[illegible]	[illegible]
20922	ENSMUSG00	99371	Arfgef2	1	protein codi	MGI Symbol	2	ADP-ribosyla	[illegible]	[illegible]	[illegible]	[illegible]	[illegible]	[illegible]	[illegible]	[illegible]	[illegible]	[illegible]	[illegible]	[illegible]	[illegible]	[illegible]	[illegible]	[illegible]	[illegible]	[illegible]	[illegible]	[illegible]	[illegible]	[illegible]	[illegible]	[illegible]	[illegible]	[illegible]	[illegible]	[illegible]	[illegible]	[illegible]	[illegible]	[illegible]	[illegible]	[illegible]	[illegible]	[illegible]
20950	ENSMUSG00	667840	Myh7b	1	protein codi	MGI Symbol	3	myosin, hea	[illegible]	[illegible]	[illegible]	[illegible]	[illegible]	[illegible]	[illegible]	[illegible]	[illegible]	[illegible]	[illegible]	[illegible]	[illegible]	[illegible]	[illegible]	[illegible]	[illegible]	[illegible]	[illegible]	[illegible]	[illegible]	[illegible]	[illegible]	[illegible]	[illegible]	[illegible]	[illegible]	[illegible]	[illegible]	[illegible]	[illegible]	[illegible]	[illegible]	[illegible]	[illegible]	[illegible]
21008	ENSMUSG00	NA	Morrbid	1	lincRNA	MGI Symbol	50	myeloid RNA	[illegible]	[illegible]	[illegible]	[illegible]	[illegible]	[illegible]	[illegible]	[illegible]	[illegible]	[illegible]	[illegible]	[illegible]	[illegible]	[illegible]	[illegible]	[illegible]	[illegible]	[illegible]	[illegible]	[illegible]	[illegible]	[illegible]	[illegible]	[illegible]	[illegible]	[illegible]	[illegible]	[illegible]	[illegible]	[illegible]	[illegible]	[illegible]	[illegible]	[illegible]	[illegible]	[illegible]
2108	ENSMUSG00	102857	Slc6a8	1	protein codi	MGI Symbol	13	solute carrie	[illegible]	[illegible]	[illegible]	[illegible]	[illegible]	[illegible]	[illegible]	[illegible]	[illegible]	[illegible]	[illegible]	[illegible]	[illegible]	[illegible]	[illegible]	[illegible]	[illegible]	[illegible]	[illegible]	[illegible]	[illegible]	[illegible]	[illegible]	[illegible]	[illegible]	[illegible]	[illegible]	[illegible]	[illegible]	[illegible]	[illegible]	[illegible]	[illegible]	[illegible]	[illegible]	[illegible]
21285	ENSMUSG00	14221	Fjx1	1	protein codi	MGI Symbol	1	four jointed	[illegible]	[illegible]	[illegible]	[illegible]	[illegible]	[illegible]	[illegible]	[illegible]	[illegible]	[illegible]	[illegible]	[illegible]	[illegible]	[illegible]	[illegible]	[illegible]	[illegible]	[illegible]	[illegible]	[illegible]	[illegible]	[illegible]	[illegible]	[illegible]	[illegible]	[illegible]	[illegible]	[illegible]	[illegible]	[illegible]	[illegible]	[illegible]	[illegible]	[illegible]	[illegible]	[illegible]
21288	ENSMUSG00	14269	Fnbp1	1	protein codi	MGI Symbol	10	formin bindi	[illegible]	[illegible]	[illegible]	[illegible]	[illegible]	[illegible]	[illegible]	[illegible]	[illegible]	[illegible]	[illegible]	[illegible]	[illegible]	[illegible]	[illegible]	[illegible]	[illegible]	[illegible]	[illegible]	[illegible]	[illegible]	[illegible]	[illegible]	[illegible]	[illegible]	[illegible]	[illegible]	[illegible]	[illegible]	[illegible]	[illegible]	[illegible]	[illegible]	[illegible]	[illegible]	[illegible]
21333	ENSMUSG00	NA	6330403L08	1	antisense	MGI Symbol	2	RIKEN cDNA	[illegible]	[illegible]	[illegible]	[illegible]	[illegible]	[illegible]	[illegible]	[illegible]	[illegible]	[illegible]	[illegible]	[illegible]	[illegible]	[illegible]	[illegible]	[illegible]	[illegible]	[illegible]	[illegible]	[illegible]	[illegible]	[illegible]	[illegible]	[illegible]	[illegible]	[illegible]	[illegible]	[illegible]	[illegible]	[illegible]	[illegible]	[illegible]	[illegible]	[illegible]	[illegible]	[illegible]

Table S2. Differentially expressed genes (> 2-fold) in *Trpm3*-mutant lenses summarized in Fig. 9A.

21358	ENSMUSG00	625249	Gpx4	1	protein codi	MGI Symbol	7	glutathione p	5106.51	5278.45	5604.3	6087.56	5972.19	5682.93	9060.56	9312.78	7518.08	10804.22	14815.75	14053.47	90.73052	91.689969	99.629229	100.06974	97.759633	99.637746	123.13617	113.25904	109.4268	164.77469	200.18653	201.74155	0.0767828	6.9591037	0.399115	0.8289446	0.2941006	6.9591037	0.0012057	0.0045726	1.006708	6.9591037	3.21E-28	3.34E-27
2156	ENSMUSG00	66848	Fuca2	1	protein codi	MGI Symbol	6	fucosidase, a	664	599	700	714	748	772	1333	1467	1203	1713	1957	1744	11.797698	10.405004	12.444098	11.737017	12.244119	13.535331	18.115935	17.841183	17.509848	26.124888	26.442471	25.035615	0.1145177	4.0899786	0.2548451	0.7300718	0.6261462	4.0899786	1.73E-10	2.59E-09	1.1635111	4.0899786	7.94E-33	9.99E-32
2173	ENSMUSG00	19714	Rev3l	1	protein codi	MGI Symbol	7	REV3 like, DI	826	822	766	836	1026	1049	1321	1787	1335	2022	2282	2395	14.676053	14.278653	13.617399	13.742502	16.794741	18.39192	17.952851	21.73292	19.431128	30.837434	30.833786	34.380905	0.2001501	4.3675799	0.0413357	0.3213735	0.4742041	4.3675799	9.87E-07	8.84E-06	1.1737872	4.3675799	3.40E-34	4.51E-33
2177	ENSMUSG00	16775	Lama4	1	protein codi	MGI Symbol	7	laminin, alph	2	9	8	10	9	7	649	883	572	2354	2416	2543	0.0355352	0.1563356	0.1422183	0.164384	0.1473223	0.1227297	8.8201365	10.738762	8.325547	35.900751	32.644359	36.505487	0.3729067	3.505736	0.4607854	0.8587044	6.3571803	3.505736	6.39E-229	8.67E-226	8.2670532	3.505736	0	0
2192	ENSMUSG00	76743	Gje1	1	protein codi	MGI Symbol	1	gap junction	6725	5366	6534	7161	7561	7043	11258.99	11817	10859	20008	20586	20792	119.48723	93.210767	116.15677	117.71538	123.76709	123.4836	153.0136	143.71456	158.0544	305.14113	278.15264	298.47506	0.1503431	7.4034186	0.0977781	0.4904394	0.4677013	7.4034186	2.53E-07	2.55E-06	1.4229556	7.4034186	5.68E-54	1.37E-52
2194	ENSMUSG00	57319	Smpdl3a	1	protein codi	MGI Symbol	3	sphingomye	173	189	160	216	210	178	390	401	354	670	937	964	3.073798	3.2830479	2.8443653	3.5506943	3.4375201	3.1208406	5.3002361	4.8768332	5.1525238	10.218141	12.660498	13.838494	0.1363481	2.5976496	0.2821612	0.749253	0.73484	2.5976496	3.62E-10	5.24E-09	1.9964622	2.5976496	1.33E-70	4.46E-69
2217	ENSMUSG00	18451	P4ha1	1	protein codi	MGI Symbol	3	procollagen-	5774	6666	5193	6036	6450	6738	10787	12034	8933	14957	20454	17588	102.59023	115.79258	92.317432	99.222179	105.58097	118.13609	146.59909	146.35364	130.02117	228.10855	276.36909	252.48073	0.0556197	7.2406674	0.5404813	0.888141	0.445076	7.2406674	9.42E-07	8.48E-06	1.2846913	7.2406674	1.53E-44	2.90E-43
2228	ENSMUSG00	67972	Atp2b1	1	protein codi	MGI Symbol	11	ATPase, Ca+	220	197	236	194	248	222	806	925	728	1451	1693	1594	3.908876	3.4220129	4.1954389	3.1890495	4.0595475	3.8922844	10.953821	11.249553	10.596151	22.129138	22.875372	22.882323	-0.049078	3.3832528	0.7010838	0.9430758	1.5092618	3.3832528	3.46E-43	2.81E-41	2.5582145	3.3832528	8.52E-123	6.92E-121
2231	ENSMUSG00	71371	Arid5b	1	protein codi	MGI Symbol	4	AT rich inter	508	410	486	460	580	557	691	920	730	1219	1301	1342	9.0259501	7.1219557	8.6397597	7.5616637	9.4941031	9.7657766	9.3909311	11.188744	10.625261	18.590916	17.578771	19.264791	0.1134734	3.5357524	0.2785498	0.7481491	0.3330745	3.5357524	0.0011298	0.004342	1.1611917	3.5357524	3.86E-31	4.60E-30
2235	ENSMUSG00	67603	Dusp6	1	protein codi	MGI Symbol	4	dual specific	158	148	225	171	230	200	315	236	248	967	1499	1248	2.8072837	2.5708523	3.9998888	2.8109663	3.7649029	3.5065625	4.2809599	2.8701562	3.6096777	14.747675	20.254095	17.915394	0.1048973	2.8159422	0.4106626	0.8370832	0.1950604	2.8159422	0.1060276	0.1701169	2.4977402	2.8159422	2.27E-111	1.54E-109
2241	ENSMUSG00	67454	Ikbip	1	protein codi	MGI Symbol	6	IKBKB intera	353	360	435	370	428	427	1121	1360	946	2066	2729	2509	6.2719692	6.2534245	7.7331183	6.0822078	7.0059933	7.486511	15.234781	16.539883	13.769174	31.508476	36.873533	36.017407	0.0217629	4.0046311	0.8461713	0.9809135	1.1697981	4.0046311	1.46E-30	7.35E-29	2.3657354	4.0046311	4.57E-122	3.65E-120
2244	ENSMUSG00	11783	Apaf1	1	protein codi	MGI Symbol	8	apoptotic pe	218	133	176	175	186	189	396	428	377	582	716	681	3.8733408	2.310293	3.1288019	2.8767199	3.0446606	3.3137016	5.3817782	5.2051985	5.4872923	8.8760566	9.6744044	9.7759484	-0.011807	2.4173261	0.9489879	1	0.7878324	2.4173261	1.43E-11	2.46E-10	1.6061953	2.4173261	1.49E-45	2.89E-44
22501	ENSMUSG00	12176	Bnip3	1	protein codi	MGI Symbol	5	BCL2/adeno	1030.15	990.72	1142.43	908.28	1129.45	1045.72	2144.27	1949.85	1565.11	4216.47	6693.32	6828.78	18.303312	17.209424	20.309302	14.930669	18.488129	18.334413	29.141377	23.713449	22.780414	64.3052	90.438386	98.029076	-0.109317	5.1887713	0.2608114	0.7337405	0.4383238	5.1887713	4.21E-06	3.30E-05	2.1791948	5.1887713	1.22E-113	8.66E-112
2254	ENSMUSG00	14219	Ccn2	1	protein codi	MGI Symbol	3	cellular com	824.92	665.49	737.25	811.25	912.79	742.93	3082.48	2671.75	2368.73	3359.99	5264.71	4296.82	14.656864	11.559976	13.106302	13.335652	14.94159	13.025652	41.891979	32.492965	34.477225	51.243061	71.135382	61.682071	0.0714172	4.9680776	0.476297	0.864464	1.4689398	4.9680776	3.09E-52	3.26E-50	2.2271676	4.9680776	7.95E-116	6.04E-114
22540	ENSMUSG00	NA	Gm7336	1	processed p	MGI Symbol	1	predicted ge	5544.48	5545.82	5643.76	6709.75	6149.98	6467.04	10322.89	9024.55	7288.54	11941.8	17283.02	16900.68	98.512204	96.334353	100.33072	110.29755	100.6699	113.3854	140.29168	109.75368	106.08581	182.12387	233.52364	242.61406	0.1359431	7.0901024	0.134876	0.5649012	0.2708254	7.0901024	0.0028526	0.0092458	1.1571477	7.0901024	1.33E-36	1.97E-35
22552	ENSMUSG00	100038347	Fam174b	1	protein codi	MGI Symbol	3	family with s	144.82	143.81	142	159	170	157.87	252.52	312	251	339	479.84	436.84	2.5731065	2.4980694	2.5243742	2.6137055	2.7827543	2.7679051	3.4318349	3.7944438	3.6533432	5.1700742	6.4834723	6.2709623	0.1039308	1.9214648	0.4413562	0.850865	0.519012	1.9214648	3.15E-05	0.0001991	1.2400174	1.9214648	3.40E-25	3.00E-24
2262	ENSMUSG00	237459	Cdk17	1	protein codi	MGI Symbol	6	cyclin-deper	168.01	159.15	206.2	174.04	209.1	214	315.64	309.21	290.32	433.68	430.78	448.58	2.9851375	2.7645348	3.6656758	2.860939	3.4227878	3.7520219	4.2896577	3.7605127	4.2256518	6.6140347	5.8205865	6.4394933	0.0909298	2.0966142	0.4713156	0.864464	0.3812468	2.0966142	0.0014243	0.0052569	1.0024898	2.0966142	4.61E-18	2.63E-17
22739	ENSMUSG00	19122	Prnp	1	protein codi	MGI Symbol	2	prion protei	542	540	626	600	628.99	538.99	1178	1290	1170	1727.95	2037	2134	9.6300491	9.3801368	11.128579	9.8630396	10.296027	9.4500106	16.009431	15.688566	17.029528	26.35289	27.52341	30.634176	-0.02508	4.0172125	0.8102973	0.9719604	0.6927589	4.0172125	2.69E-12	4.93E-11	1.4876045	4.0172125	7.56E-52	1.72E-50
22768	ENSMUSG00	328099	Prps1l3	1	protein codi	MGI Symbol	1	phosphoribc	164.6	142.97	162.94	192.56	165.43	192.62	285.97	325.6	228.62	348.53	419.11	430.73	2.92455	2.4834781	2.8966305	3.1653782	2.7079474	3.3771704	3.8864321	3.9598426	3.3275989	5.3154158	5.6629045	6.1832515	0.1545362	1.9596519	0.230116	0.7105312	0.4311116	1.9596519	0.0004576	0.0020348	1.0477927	1.9596519	8.94E-19	5.36E-18
2278	ENSMUSG00	74007	Btbd11	1	protein codi	MGI Symbol	8	BTB (POZ) d	166	183	120	153	144	204	253	273	244	530	577	528	2.9494246	3.1788241	2.133274	2.5150751	2.3571566	3.5766938	3.4383583	3.3201383	3.5514571	8.0830068	7.7962728	7.5795899	0.0270025	2.0974373	0.8357048	0.9788296	0.3172783	2.0974373	0.0104774	0.0267138	1.5038115	2.0974373	2.09E-38	3.30E-37
2280	ENSMUSG00	22027	Hsp90b1	1	protein codi	MGI Symbol	5	heat shock p	7195.83	6855.37	7406.37	7731.38	8367.17	7824.81	14081.02	14766.03	13173.59	18339.71	21205.54	22619.68	127.85276	119.08205	131.66514	127.09151	136.9634	137.19093	191.36598	179.57971	191.74361	279.69812	286.5237	324.71193	0.0838094	7.5409094	0.355346	0.7993813	0.5716358	7.5409094	2.88E-10	4.22E-09	1.2346518	7.5409094	1.82E-41	3.13E-40
22852	ENSMUSG00	56441	Naa80	1	protein codi	MGI Symbol	7	N(alpha)-ace	828.05	758.61	666.5	674.5	671.37	635.32	1209.62	1278.96	927.67	1751.68	2032.23	2010.02	14.712476	13.177529	11.848559	11.0877	10.989752	11.138946	16.439158	15.554301	13.502378	26.714795	27.458959	28.854408	-0.258635	4.0768175	0.0098893	0.1244348	0.1958465	4.0768175	0.0461029	0.0871771	1.0630211	4.0768175	4.20E-28	4.35E-27
22908	ENSMUSG00	20448	St6galnac4	1	protein codi	MGI Symbol	5	ST6 (alpha-N	380	419	389	477	395	465	778	891	643	1218	1680	1526	6.7516949	7.2782913	6.9153632	7.8411165	6.4658116	8.1527578	10.573291	10.836056	9.3589628	18.575665	22.69972	21.906163	0.0996858	3.5299548	0.352158	0.7963323	0.5556385	3.5299548	7.66E-08	8.39E-07	1.5930635	3.5299548	2.99E-56	7.62E-55
22936	ENSMUSG00	NA	6530402F18	1	processed t	MGI Symbol	3	RIKEN cDNA	331.14	224.28	292.74	310.27	325.35	426	580.89	631.68	593.86	1143.91	1406.6	1300	5.8835691	3.8958835	5.2041219	5.1003422	5.3257007	7.4689781	7.8944978	7.6822892	8.6437226	17.445721	19.005611	18.661869	0.2543425	3.2409561	0.0224614	0.2186305	0.6924493	3.2409561	1.17E-10	1.78E-09	1.8798058	3.2409561	1.16E-73	4.12E-72
230	ENSMUSG00	19411	Rarg	1	protein codi	MGI Symbol	7	retinoic acid	267	229	206	248	233	254	362	403	322	545	663	683	4.7439541	3.9778728	3.6621204	4.076723	3.8140104	4.4533344	4.9197063	4.9011565	4.686759	8.3117712	8.9582823	9.804659	-0.005614	2.4840931	0.972751	1	0.2289625	2.4840931	0.0442599	0.0842329	1.1287141	2.4840931	8.28E-25	7.12E-24
2315	ENSMUSG00	107449	Unc5b	1	protein codi	MGI Symbol	3	unc-5 netrin	385	420	300	498	438	458	1838	2138	1245	4575	5942	4832	6.840533	7.295662	5.333185	8.1863229	7.1696847	8.0300281	24.979061	26.001669	18.121164	69.773125	80.286747	69.364732	0.2636167	4.7977063	0.0137693	0.1574102	1.8280454	4.7977063	3.39E-73	5.76E-71	3.4937366	4.7977063	1.95E-254	7.71E-252
2319	ENSMUSG00	320398	Lrig3	1	protein codi	MGI Symbol	7	leucine-rich	232	184	220	213	223	219	446	518	398	619	797	775	4.1220874	3.1961948	3.9110023	3.5013791	3.6503189	3.8396859	6.0612956	6.2997496	5.7929505	9.440342	10.768855	11.125345	-0.030819	2.6022197	0.8092957	0.9716048	0.6944529	2.6022197	9.26E-10	1.28E-08	1.4814344	2.6022197	4.09E-41	6.99E-40
2321	ENSMUSG00	74747	Ddit4	1	protein codi	MGI Symbol	1	DNA-damag	503	542	404	508	400	576	917	916	581	1452	1923	1494	8.937112	9.4148781	7.1820225	8.3507069	6.5476573	10.0989	12.46235	11.140098	8.4565434	22.144389	25.983072	21.446794	-0.032777	3.6754733	0.7682883	0.961343	0.329585	3.6754733	0.0012289	0.00464	1.4460676	3.6754733	5.59E-48	1.14E-46
2339	ENSMUSG00	23805	Apc2	1	protein codi	MGI Symbol	6	APC regulatc	129	132	96	152	162	187	192	271	225	268	351	298	2.2920228	2.2929223	1.7066192	2.4986367	2.6518012	3.2786359	2.609347	3.2958149	3.2749092	4.0872563	4.74262	4.2778746	0.4182645	1.6524588	0.0018069	0.0333953	0.5448035	1.6524588	2.96E-05	0.0001891	1.059171	1.6524588	3.78E-17	2.02E-16
23466	ENSMUSG00	NA	Ywhaq-ps3	1	transcribed	MGI Symbol	1	tyrosine 3-m	105.12	138.53	196.89	172.61	210.07	165.36	349.6	272.94	280.1	433.88	572.07	538.06	1.867732	2.4063525	3.5001693	2.8374321	3.4386659	2.8992259	4.751186	3.3194086	4.0768981	6.6170849	7.7296599	7.7240041	0.2402482	2.1190034	0.0645198	0.399813	0.6404158	2.1190034	1.61E-07	1.67E-06	1.5062308	2.1190034	1.19E-37	1.83E-36
2347	ENSMUSG00	66713	Actr2	1	protein codi	MGI Symbol	3	ARP2 actin-r	1535	1424	1648	1635	1805	1660	2659	3003	2535	3528	4482	4223	27.273294	24.735768	29.296963	26.876783	29.546304	29.104469	36.136738	36.521521	36.897311	53.805374	60.55961	60.622364	0.0730656	5.2367154	0.4402697	0.850865	0.4302794	5.2367154	4.27E-06	3.34E-05	1.105997	5.2367154	3.53E-32	4.33E-31
2368	ENSMUSG00	260315	Nav3	1	protein codi	MGI Symbol	8	neuron navi	344	322	369	372	448	400	537	575	608	845	850	940	6.1120607	5.5933408	6.5598176	6.1150846	7.3333762	7.013125	7.2980174	6.9929653	8.8495325	12.887058	11.484977	13.493967	0.1638483	3.0663411	0.1335173	0.5624475	0.3392452	3.0663411	0.0013661	0.0050854	1.0513376	3.0663411	3.03E-24	2.53E-23
23745	ENSMUSG00	NA	Gm14760	1	processed p	MGI Symbol	1	predicted ge	835.16	944.61	710.31	982.2	902.6	852.62	1490.73	1305.1	1029.71	1641.68	2500.9	2357.31	14.838804	16.408465	12.627382	16.145796	14.774789	14.948827	20.259541	15.872207	14.987586	25.03719	33.791505	33.839854	0.0640017	4.2885883	0.5172638	0.8790824	0.2202893	4.2885883	0.023339	0.0508416	1.0789743	4.2885883	3.15E-29	3.42E-28
2413	ENSMUSG00	110532	Adarb1	1	protein codi	MGI Symbol	13	adenosine d	804	769	728	847	859	881	1158	1202	1145	1798	2035	1900	14.285165	13.35801	12.941862	13.923324	14.061094	15.446408	15.737624	14.618338	16.665649	27.421219	27.496387	27.27504	0.0974205	4.15694	0.3255532	0.7793548	0.2116427	4.15694	0.0302417	0.0622123	1.017984	4.15694	5.90E-26	5.49E-25
2427	ENSMUSG00	13650	Rhbdf1	1	protein codi	MGI Symbol	9	rhomboid 5	296	264	267	296	310	303	607	620	455	1053	1302	1218	5.259215	4.5858447	4.7465347	4.8657662	5.0744334	5.3124422	8.2493418	7.5402408	6.6225942	16.059257	17.592283	17.484736	0.0636223	3.1248384	0.5768066	0.9029106	0.6202086	3.1248384	1.05E-08	1.30E-07	1.8093623	3.1248384	7.28E-68	2.33E-66
2436	ENSMUSG00	18197	Nsg2	1	protein codi	MGI Symbol	3	neuron spec	423	377	332	431	460	433	900	815	698	939	1858	1422	7.5157025	6.5487251	5.9020581	7.0849501	7.5298059	7.5917078	12.231314	9.9117682	10.159496	14.320648	25.104809	20.413214	0.1531828	3.4988632	0.1548389	0.6001506	0.6935651	3.4988632	1.94E-11	3.27E-10	1.5849995	3.4988632	3.22E-55	8.02E-54
24440	ENSMUSG00	NA	Pgam1-ps2	1	processed p	MGI Symbol	1	phosphoglyc	766.37	729.38	702.66	724.88	639.43	736.48	1418.97	1511.2	1114.97	2194.22	3044.2	2138.22	13.61657	12.669786	12.491386	11.915867	10.466921	12.912566	19.284297	18.378729	16.228558	33.463953	41.132433	30.694755	-0.136334	4.2889395	0.1750443	0.6327705	0.4753254	4.2889395	1.10E-06	9.74E-06	1.4413974	4.2889395	3.28E-50	7.14E-49
2445	ENSMUSG00	66753	Erlec1	1	protein codi	MGI Symbol	5	endoplasmic	640	573	629	707	709	708	1227	1364	1108	1473	1570	1761	11.371276	9.9533674	11.181911	11.621948	11.605723	12.413231	16.675358	16.58853	16.127109	22.464659	21.213429	25.279655	0.1326898	3.9662274	0.1897605	0.6569698	0.6037615	3.9662274	9.35E-10	1.29E-08	1.0848908	3.9662274	1.76E-28	1.86E-27
2452	ENSMUSG00	83554	Fstl3	1	protein codi	MGI Symbol	3	follistatin-lik	268.86	279.85	210.69	343.46	430.59	361.58	388	463.55	398.88	490.97	849.02	732.91	4.7770018	4.8611691	3.7454958	5.6459327	7.0483894	6.3395144	5.2730554	5.6375462	5.8057591	7.487762	11.471736	10.521131	0.5075527	2.7260905	6.53E-06	0.0002432	0.3200196	2.7260905	0.0040723	0.0123877	1.1409158	2.7260905	6.78E-26	6.26E-25
247	ENSMUSG00	56700	Glmp	1	protein codi	MGI Symbol	10	glycosylated	794.81	760.91	736.68	873.86	777.76	776.97	1373	1581.38	1281.59	1816.91	2257.96	2049.87	14.121881	13.217481	13.096169	14.36486	12.731265	13.622469	18.659549	19.232236	18.653737	27.709615	30.508964	29.426466	0.010058	4.2381149	0.9239529	0.9930291	0.4839227	4.2381149	6.40E-07	5.98E-06	1.1161913	4.2381149	6.84E-31	8.07E-30
248	ENSMUSG00	17261	Mef2d	1	protein codi	MGI Symbol	7	myocyte enh	676.73	612.18	633.21	657.47	683.97	721.7	1171.44	1329.05	1288.79	2176.11	2567.35	2437.35	12.02388	10.633948	11.256754	10.807754	11.196003	12.653431	15.920278	16.163479	18.758534	33.187759	34.68936	34.988851	0.0307696	4.2195873	0.7634033	0.9601813	0.5834506	4.2195873	2.91E-09	3.81E-08	1.6008427	4.2195873	1.27E-60	3.57E-59
250	ENSMUSG00	56463	Snd1	1	protein codi	MGI Symbol	12	staphylococ	2274	2017	2179	2251	2416	2430	3940	4361	3882	5021	5820	5346	40.403564	35.036548	38.7367	37.002837	39.54785	42.604734	53.545975	53.037081	56.5031	76.575052	78.638315	76.743348	0.0614801	5.7130209	0.5095118	0.8764009	0.5143004	5.7130209	2.67E-08	3.12E-07	1.022636	5.7130209	2.79E-28	2.91E-27
251	ENSMUSG00	12822	Col18a1	1	protein codi	MGI Symbol	5	collagen, typ	84	68	85	72	83	63	419	717	687	3555	3683	4763	1.4924799	1.1812024	1.5110691	1.1835648	1.3586389	1.1045672	5.6943562	8.7199236	9.9993895	54.21715	49.763731	68.374218	-0.19489	4.108646	0.2408556	0.72048	2.5431148	4.108646	2.25E-88	4.86E-86	5.363175	4.108646	0	0
2515	ENSMUSG00	432530	Adcy1	1	protein codi	MGI Symbol	2	adenylate cy	1297	1359	1303	1486	1515	1590	4679	5753	4157	7441	8798	8106	23.044601	23.606678	23.1638	24.427461	24.799252	27.877172	63.589243	69.966138	60.505767	113.48237	118.87627	116.36393	0.1429582	5.8492208	0.132469	0.5600602	1.4750294	5.8492208	4.68E-55	5.36E-53	2.3203812	5.8492208	4.53E-129	4.22E-127
2516	ENSMUSG00	21452	Tcn2	1	protein codi	MGI Symbol	7	transcobalar	670	598	648	573	591	625	1338	1320	1119	1903	2283	2317	11.904304	10.387633	11.51968	9.4192028	9.6741636	10.958008	18.183887	16.053416	16.287215	29.02257	30.847298	33.261193	-0.170545	4.12122	0.0944183	0.4835194	0.5794095	4.12122	3.84E-09	4.96E-08	1.4618951	4.12122	7.95E-51	1.77E-49
2533	ENSMUSG00	68585	Rtn4	1	protein codi	MGI Symbol	10	reticulon 4 [	4155.27	3792	4239.53	4373.24	4492.69	4057.19	8674.12	9081.43	7868.93	11791.71	14018.85	14009.84	73.829251	65.869405	75.367326	71.889066	73.541486	71.133952	117.88432	110.44543	114.53347	179.83485	189.41903	201.11523	0.0100727	6.8097914	0.9135092	0.9914701	0.6728402	6.8097914	1.73E-13	3.53E-12	1.4071477	6.8097914	1.79E-52	4.13E-51
2544	ENSMUSG00	11568	Aebp1	1	protein codi	MGI Symbol	3	AE binding p	5520	4898	4810	5523	6221	5823	9156	10527	8485	12555	15752	15406	98.077253	85.081315	85.508733	90.78928	101.83244	102.09357	124.43323	128.02599	123.50047	191.47576	212.8369	221.1575	0.1334823	7.0279126	0.1426923	0.5812939	0.4847952	7.0279126	9.79E-08	1.05E-06	1.2191553	7.0279126	2.76E-40	4.57E-39
2552	ENSMUSG00	22433	Xbp1	1	protein codi	MGI Symbol	5	X-box bindin	839	753	757	745	677	749	1543	1655	1344	1830	2305	2093	14.907032	13.08008	13.457403	12.246608	11.08191	13.132077	20.969908	20.127578	19.562124	27.90925	31.144556	30.045609	-0.185228	4.2534056	0.0634561	0.396963	0.5496857	4.2534056	1.41E-08	1.72E-07	1.1044728	4.2534056	2.34E-30	2.71E-29
2580	ENSMUSG00	20787	Srebf1	1	protein codi	MGI Symbol	12	sterol regula	1910	1851	2024	1781	1661	1632	2704	3272	2551	4787	6070	5303	33.936151	32.153025	35.981221	29.276789	27.189147	28.61355	36.748304	39.793013	37.130193	73.006328	82.016249	76.126071	-0.262646	5.4733044	0.0051589	0.0770586	0.1555371	5.4733044	0.0948115	0.1556974	1.1793659	5.4733044	9.34E-37	1.39E-35
2586	ENSMUSG00	66912	Bzw2	1	protein codi	MGI Symbol	10	basic leucine	227	231	223	217	261	191	444	454	365	698	789	789	4.0332493	4.0126141	3.9643342	3.5671327	4.2723464	3.3487672	6.0341149	5.5214021	5.3126305	10.645167	10.660761	11.326319	-0.100719	2.6200106	0.408907	0.8362231	0.4900979	2.6200106	1.42E-05	9.77E-05	1.4415884	2.6200106	8.97E-40	1.47E-38
2596	ENSMUSG00	59027	Nampt	1	protein codi	MGI Symbol	3	nicotinamide	449	430	435	462	452	460	1072	1132	881	1941	2785	2768	7.9776606	7.4693682	7.7331183	7.5945405	7.3988527	8.0650938	14.568854	13.76702	12.823089	29.602106	37.63019	39.735426	-0.007929	4.0300831	0.9493608	1	0.8286124	4.0300831	2.77E-16	7.04E-15	2.2064595	4.0300831	2.59E-108	1.69E-106
25963	ENSMUSG00	NA	Gm13394	1	processed p	MGI Symbol	1	predicted ge	399.22	578.8	436.64	579.88	643.17	509.01	709.07	853.85	608.86	975.93	1495.79	1373.92	7.0931885	10.054117	7.762273	9.532299	10.528142	8.9243769	9.6365087	10.384249	8.8620499	14.883866	20.210723	19.723012	0.2185165	3.5290755	0.0353121	0.2947414	0.2141628	3.5290755	0.0371599	0.0734997	1.1381534	3.5290755	5.26E-30	5.96E-29
2600	ENSMUSG00	19878	Rock2	1	protein codi	MGI Symbol	4	Rho-associat	1352	1213	1379	1453	1635	1481	2355	2562	2216	3512	4624	4718	24.02182	21.070567	24.514874	23.884994	26.763549	25.966095	32.005272	31.158221	32.254217	53.561359	62.478276	67.72823	0.1384437	5.1518367	0.1457779	0.5853583	0.4550005	5.1518367	1.36E-06	1.18E-05	1.4007843	5.1518367	7.59E-50	1.63E-48
2607	ENSMUSG00	20969	Sdc1	1	protein codi	MGI Symbol	5	syndecan 1 [	1753	1617	1802	2000	1978	1836	3163	3417	2868	5112	6243	6303	31.146635	28.088299	32.034665	32.876799	32.378165	32.190244	42.986274	41.556456	41.744176	77.962889	84.35378	90.481355	0.09455	5.5675056	0.3147262	0.7706279	0.4685314	5.5675056	4.91E-07	4.65E-06	1.469861	5.5675056	2.05E-55	5.14E-54
2635	ENSMUSG00	14226	Fkbp1b	1	protein codi	MGI Symbol	2	FK506 bindir	134	111	139	137	127	114	315	346	302	542	636	721	2.3808608	1.9281392	2.4710424	2.2520607	2.0788812	1.9987406	4.2809599	4.2079408	4.3956559	8.2660183	8.5934653	10.35016	-0.097209	2.1823031	0.4916874	0.8711141	0.9259837	2.1823031	8.20E-14	1.72E-12	2.0049762	2.1823031	3.02E-64	9.03E-63
2646	ENSMUSG00	20135	Rrm2	1	protein codi	MGI Symbol	4	ribonucleoti	210	193	227	224	201	240	448	342	333	591	635	635	3.7311998	3.3525304	4.0354433	3.6822015	3.2901978	4.207875	6.0884763	4.1592941	4.8468656	9.0133152	8.5799536	9.1156053	0.0065212	2.4371397	0.9556938	1	0.4387796	2.4371397	0.0001354	0.0007152	1.2639926	2.4371397	5.89E-30	6.65E-29
26508	ENSMUSG00	NA	Gm13370	1	processed p	MGI Symbol	1	predicted ge	1302.46	1932.7	418.59	587.65	535.32	527.65	1367.7	995.81	1822.11	1899.38	2169.05	1906.87	23.141612	33.572204	7.441393	9.6600254	8.7627297	9.2511885	18.58752	12.110721	26.521088	28.967361	29.307635	27.37366	-1.213684	4.2939879	2.43E-34	8.24E-32	-0.169347	4.2939879	0.0817815	0.1380462	0.4160644	4.2939879	1.07E-05	1.82E-05
268	ENSMUSG00	20391	Sgca	1	protein codi	MGI Symbol	5	sarcoglycan,	99	90	85	128	114	138	357	398	323	536	630	671	1.7589942	1.5633561	1.5110691	2.1041151	1.8660823	2.4195281	4.8517546	4.8403481	4.7013141	8.1745126	8.5123949	9.6323955	0.3998703	2.1527591	0.0061947	0.0883026	1.5737352	2.1527591	5.14E-34	2.96E-32	2.4440189	2.1527591	1.35E-85	5.57E-84
2683	ENSMUSG00	26406	Map3k3	1	protein codi	MGI Symbol	3	mitogen-acti	369	350	343	397	368	424	669	722	685	935	1120	1021	6.5562511	6.0797183	6.0976082	6.5260446	6.0238447	7.4339125	9.0919434	8.780732	9.9702792	14.259644	15.133147	14.656745	0.0921106	3.2177409	0.3990115	0.8289109	0.5705683	3.2177409	4.97E-08	5.55E-07	1.2335802	3.2177409	2.89E-33	3.71E-32
2709	ENSMUSG00	15374	Jpt1	1	protein codi	MGI Symbol	5	Jupiter micr	388	400	413	466	434	465	708	735.49	694.79	945	1317	1170.94	6.8938359	6.9482495	7.342018	7.6602941	7.1042082	8.1527578	9.621967	8.9447931	10.112774	14.412154	17.794959	16.809176	0.1128593	3.3551791	0.2915972	0.7546101	0.4359556	3.3551791	2.56E-05	0.0001658	1.2109026	3.3551791	7.46E-33	9.41E-32
27155	ENSMUSG00	NA	Brip1os	1	antisense	MGI Symbol	2	BRCA1 inter	251.85	250.06	297.78	310.49	267.95	268.47	392.21	457.09	414.74	564.38	756.1	696.39	4.4747746	4.3436982	5.2937194	5.1039586	4.3861119	4.7070342	5.3302708	5.5589817	6.0366038	8.6073347	10.216225	9.9968762	0.0089978	2.6416184	0.951099	1	0.2616195	2.6416184	0.0186185	0.0424161	1.0311854	2.6416184	1.22E-21	8.83E-21
27179	ENSMUSG00	NA	Snhg3	1	processed t	MGI Symbol	5	small nucleo	151.56	146.31	170.91	203.62	254.68	209.25	305.52	342.36	291.85	351.59	392.32	500.23	2.6928602	2.541496	3.0383155	3.3471869	4.1688934	3.668741	4.1521234	4.1636723	4.2479211	5.3620837	5.300925	7.1809437	0.4356067	2.0774074	0.0004927	0.0110127	0.6026803	2.0774074	6.27E-07	5.88E-06	1.1088499	2.0774074	6.95E-21	4.79E-20
2723	ENSMUSG00	217333	Trim47	1	protein codi	MGI Symbol	7	tripartite mc	122	94	93	149	103	124.12	583	575	473	1553	2072.32	1807	2.1676494	1.6328386	1.6532874	2.4493215	1.6860218	2.1761727	7.9231734	6.9929653	6.8845869	23.684735	28.000645	25.939998	0.2103279	3.2389707	0.1456342	0.5852474	1.9985508	3.2389707	3.90E-59	5.01E-57	3.830847	3.2389707	7.27E-226	1.92E-223
2732	ENSMUSG00	66874	Ncbp3	1	protein codi	MGI Symbol	3	nuclear cap	743	688	766	728	848	847	1170	1263	1093	1541	2059	1935	13.20134	11.950989	13.617399	11.967155	13.881033	14.850292	15.900708	15.3602	15.908781	23.501724	27.820668	27.777474	0.0696784	4.1061779	0.4850437	0.8690899	0.2828446	4.1061779	0.0038864	0.0119089	1.0292043	4.1061779	2.40E-26	2.28E-25
27523	ENSMUSG00	NA	Gm12992	1	processed t	MGI Symbol	2	predicted ge	106.3	114.26	73.18	111.22	154.21	132.95	275.27	284.77	232.65	506.97	497.4	447.75	1.8886978	1.9847675	1.3009416	1.8282788	2.5242856	2.3309874	3.7410153	3.4632813	3.3862561	7.7317773	6.7207385	6.4275784	0.3670738	1.8883721	0.0101263	0.1264122	1.0315544	1.8883721	4.67E-15	1.07E-13	2.0092127	1.8883721	3.38E-58	8.94E-57
2755	ENSMUSG00	69900	Mfsd11	1	protein codi	MGI Symbol	12	major facilit	244.45	187.05	200.28	285.94	244.35	244.85	436.57	445.89	408.86	514.23	670.32	515.52	4.3432943	3.2491752	3.5604343	4.7003959	3.9998001	4.2929092	5.9331386	5.4227709	5.9510195	7.8424993	9.0571882	7.400436	0.2208683	2.4740975	0.0651097	0.401144	0.6330724	2.4740975	2.41E-08	2.83E-07	1.124755	2.4740975	7.00E-24	5.70E-23
2756	ENSMUSG00	16562	Kif1c	1	protein codi	MGI Symbol	5	kinesin famil	4980	4998	5121	5169	5113	5222	9352	9874	8293	13103	15018	13628	88.482739	86.818377	91.037468	84.970086	83.695429	91.556347	127.09694	120.08442	120.70588	199.83328	202.91928	195.63381	-0.033569	6.9600327	0.713446	0.9454671	0.4659921	6.9600327	2.99E-07	2.96E-06	1.1678116	6.9600327	3.63E-37	5.48E-36
277	ENSMUSG00	16480	Jup	1	protein codi	MGI Symbol	9	junction plal	167	130	173	157	193	199	473	572	456	1476	1810	1555	2.9671922	2.2581811	3.07547	2.5808287	3.1592446	3.4890297	6.428235	6.9564802	6.6371494	22.510412	24.456246	22.322467	0.1518901	3.1783274	0.2471595	0.7231049	1.2713484	3.1783274	1.25E-27	5.65E-26	3.0616909	3.1783274	6.29E-162	9.19E-160
2773	ENSMUSG00	76566	Rflnb	1	protein codi	MGI Symbol	1	refilin B [Sou	160	159	215	203	200	189	332	453	358	663	570	615	2.8428189	2.7619292	3.8221159	3.3369951	3.2738286	3.3137016	4.5119958	5.5092405	5.2107445	10.111384	7.7016906	8.8284996	0.0748122	2.3750644	0.5631366	0.8961357	0.6937207	2.3750644	3.16E-09	4.11E-08	1.4971895	2.3750644	1.03E-39	1.69E-38
2786	ENSMUSG00	98238	Lrrc59	1	protein codi	MGI Symbol	2	leucine rich	2004.37	1789.27	2045.52	2090.03	1944.52	2066.09	3486.03	3736.02	3143.23	4267.15	5116.23	5071.66	35.612881	31.080736	36.363789	34.356748	31.830126	36.224369	47.376364	45.436275	45.750191	65.078118	69.129159	72.80512	-0.009149	5.5238643	0.9243264	0.9931125	0.4271327	5.5238643	4.16E-06	3.27E-05	1.0063737	5.5238643	2.58E-27	2.57E-26
2801	ENSMUSG00	217166	Nr1d1	1	protein codi	MGI Symbol	2	nuclear rece	184.6	202.43	242.44	154.89	197.59	172.4	377.43	349.94	308.2	538.32	622.93	525.92	3.2799023	3.5163354	4.3099246	2.5461437	3.234379	3.0226569	5.1294054	4.2558579	4.4858979	8.2098948	8.4168669	7.5497309	-0.335139	2.2942651	0.0070336	0.0968392	0.3197801	2.2942651	0.00585	0.0167042	1.1222629	2.2942651	9.41E-24	7.63E-23
28087	ENSMUSG00	NA	Ftx	1	lincRNA	MGI Symbol	12	Ftx transcrip	378.14	246.48	260.26	257.3	403.63	269.38	319.87	357.36	294.26	1074.72	1459.45	1256.65	6.7186472	4.2815113	4.6267158	4.2296002	6.6070773	4.722989	4.3471449	4.3460975	4.2829991	16.390508	19.719706	18.039568	-0.004319	3.0494685	0.9745602	1	-0.267144	3.0494685	0.0173134	0.0399313	1.793889	3.0494685	1.37E-67	4.35E-66
2820	ENSMUSG00	16669	Krt19	1	protein codi	MGI Symbol	3	keratin 19 [S	104	138	113	188	164	195	228	202	197	259	483	498	1.8478323	2.3971461	2.008833	3.0904191	2.6845395	3.4188984	3.0985996	2.4566591	2.867365	3.9499977	6.5261694	7.1489314	0.5526213	1.817405	3.04E-05	0.0009757	0.4248939	1.817405	0.0011855	0.0045102	1.4967704	1.817405	4.04E-34	5.36E-33
2823	ENSMUSG00	21973	Top2a	1	protein codi	MGI Symbol	7	topoisomera	577	405.99	537	486	577	568	930	817	908	1174	1327	1382	10.251916	7.0522995	9.5464012	7.9890621	9.4449956	9.9586375	12.639025	9.9360915	13.216078	17.904623	17.930076	19.839002	0.0287531	3.6109089	0.7871961	0.9660842	0.4131487	3.6109089	4.15E-05	0.0002527	1.0524752	3.6109089	4.11E-26	3.88E-25
28259	ENSMUSG00	100038514	Gm11837	1	antisense	MGI Symbol	3	predicted ge	2112.52	2018.91	2170.59	2036.33	2243.51	2049.31	4780.92	5497.93	4293.27	6553.75	8788.79	8596.57	37.534449	35.069726	38.587193	33.474006	36.724336	35.930168	64.974371	66.864058	62.489198	99.950955	118.75183	123.4062	-0.067215	5.9760949	0.4721976	0.864464	0.8055591	5.9760949	3.34E-18	9.79E-17	1.6215082	5.9760949	3.06E-67	9.49E-66
28414	ENSMUSG00	664994	Isoc2a	1	protein codi	MGI Symbol	1	isochorisma	571	493	582	681	687	580	979	979	859	1316	1590	1346	10.14531	8.5637175	10.346379	11.19455	11.245601	10.169031	13.304952	11.906283	12.502876	20.070258	21.483663	19.322212	0.1671801	3.7475691	0.1028197	0.5042041	0.3761391	3.7475691	0.0001757	0.0008927	1.0674388	3.7475691	3.66E-27	3.62E-26
2866	ENSMUSG00	338320	Mia2	1	protein codi	MGI Symbol	23	MIA SH3 dor	757	700	725	759	827	782	1184	1505	1315	1665	1844	1881	13.450087	12.159437	12.88853	12.476745	13.537281	13.710659	16.090973	18.303326	19.140025	25.392842	24.915645	27.002289	0.0451614	4.1297831	0.6535513	0.9286055	0.4755953	4.1297831	1.09E-06	9.64E-06	1.0058375	4.1297831	3.25E-25	2.87E-24
2877	ENSMUSG00	18035	Nfkbia	1	protein codi	MGI Symbol	2	nuclear fact	222	221	554	197	323	221	389	428	436	846	1380	1246	3.9444112	3.8389078	9.848615	3.2383647	5.2872333	3.8747516	5.2866457	5.2051985	6.3460463	12.902309	18.646198	17.886684	-0.505207	3.020814	9.14E-06	0.0003277	-0.067512	3.020814	0.5518929	0.6350511	1.4897179	3.020814	1.10E-47	2.22E-46
28803	ENSMUSG00	68576	Lamtor5	1	protein codi	MGI Symbol	2	late endosor	333.99	353.98	395	430	408	376.97	651	706	586	750.99	1045.99	927	5.9342068	6.1488534	7.0220269	7.0685117	6.6786104	6.6093443	8.8473172	8.5861452	8.5293191	11.453316	14.133143	13.307348	0.0918627	3.1329929	0.4023195	0.831347	0.442521	3.1329929	2.53E-05	0.0001643	1.0264547	3.1329929	2.32E-23	1.85E-22
2891	ENSMUSG00	81535	Sgpp1	1	protein codi	MGI Symbol	2	sphingosine-	268	272	331	323	316	304	532	602	450	657	823	679	4.7617217	4.7248097	5.8842808	5.309603	5.1726493	5.329975	7.2300656	7.3213306	6.5498184	10.019878	11.12016	9.7472378	0.0410227	2.8090906	0.7338379	0.9514929	0.458422	2.8090906	2.38E-05	0.0001561	1.0074682	2.8090906	1.97E-21	1.41E-20
2901	ENSMUSG00	110095	Pygl	1	protein codi	MGI Symbol	6	liver glycoge	2667	3406	2087	5678	5784	5992	2644	4985	3361	4386	6788	6081	47.386238	59.164344	37.10119	93.337232	94.679124	105.05661	35.932883	60.625968	48.919866	66.890694	91.717677	87.294482	1.0284248	6.1092518	8.43E-29	2.29E-26	0.0186485	6.1092518	0.841954	0.8816758	0.7755925	6.1092518	3.63E-17	1.94E-16
2916	ENSMUSG00	94040	Clmn	1	protein codi	MGI Symbol	7	calmin [Sour	277	305	255	365	396	443	563	708	512	726	816	697	4.9216302	5.2980402	4.5332073	6.0000158	6.4821807	7.767036	7.6513664	8.6104685	7.4522379	11.072194	11.025578	10.005633	0.4549346	2.9345519	4.15E-05	0.0012807	0.6857015	2.9345519	2.01E-10	2.99E-09	1.1210569	2.9345519	3.87E-26	3.66E-25
2926	ENSMUSG00	75627	Snapc1	1	protein codi	MGI Symbol	5	small nuclea	241	244.77	164	404	418.05	449.27	418	668.57	410.74	480	642.03	588.7	4.281996	4.2518076	2.9154745	6.6411134	6.8431203	7.8769667	5.6807659	8.1309335	5.9783832	7.320459	8.6749411	8.4509557	0.8978008	2.6980798	2.16E-15	2.64E-13	0.7926145	2.6980798	1.65E-12	3.09E-11	1.0945211	2.6980798	7.61E-23	5.92E-22
2951	ENSMUSG00	217946	Cdca7l	1	protein codi	MGI Symbol	4	cell division	234.27	241.22	254.27	247.81	268.67	228.05	410.84	530.97	436.5	546.74	692.59	643.34	4.1624199	4.1901419	4.5202298	4.0735998	4.3978977	3.9983579	5.583459	6.4574866	6.3533239	8.3383078	9.3580946	9.2353284	-0.045211	2.5776647	0.7125276	0.9454671	0.5152812	2.5776647	3.82E-06	3.02E-05	1.0654659	2.5776647	2.06E-22	1.57E-21
2963	ENSMUSG00	19141	Lgmn	1	protein codi	MGI Symbol	3	legumain [Sc	5520	6494	4557	5851	5831	5523	7776	7624	6581	12946	15335	15468	98.077253	112.80483	81.01108	96.181075	95.448474	96.833724	105.67855	92.720639	95.787456	197.43888	207.2025	222.04753	-0.017113	6.9678598	0.851749	0.9820332	0.0111912	6.9678598	0.9020969	0.9286855	1.1022562	6.9678598	2.00E-33	2.59E-32
2967	ENSMUSG00	56421	Pfkp	1	protein codi	MGI Symbol	13	phosphofruc	82.77	62.52	60.71	73.86	86.77	86	356.13	380.64	278.35	1171.04	1763.33	1699.51	1.4706258	1.0860114	1.0792589	1.2141402	1.4203506	1.5078219	4.839931	4.6292214	4.0514266	17.85948	23.825653	24.396949	0.1867189	2.897822	0.2548601	0.7300718	1.8955417	2.897822	6.21E-44	5.22E-42	4.1832482	2.897822	2.14E-239	6.79E-237
2978	ENSMUSG00	56349	Net1	1	protein codi	MGI Symbol	7	neuroepithe	258	271	284	293	332.88	327.9	398	341	339	671	781.77	861.97	4.5840455	4.707439	5.0487485	4.816451	5.4489604	5.7490092	5.4089589	4.1471324	4.9341966	10.233392	10.563071	12.373824	0.158477	2.7139188	0.1642635	0.618511	0.0124301	2.7139188	0.9130498	0.9383355	1.2097255	2.7139188	3.96E-30	4.51E-29
2991	ENSMUSG00	12499	Entpd5	1	protein codi	MGI Symbol	8	ectonucleosi	245	259	273	269	323	293	387	507	387	628	683	680	4.3530665	4.4989915	4.8531984	4.4219294	5.2872333	5.1371141	5.259465	6.1659711	5.6328439	9.5776006	9.228517	9.7615931	0.1150061	2.6437789	0.3224008	0.777949	0.3170692	2.6437789	0.0044239	0.0132913	1.0591858	2.6437789	1.37E-22	1.05E-21
3003	ENSMUSG00	21809	Tgfb3	1	protein codi	MGI Symbol	1	transforming	119.96	126	82	143	133	150	372	430.99	357.93	818	1015	792.88	2.1314035	2.1886986	1.4577372	2.3506911	2.177096	2.6299219	5.0556098	5.2415619	5.2097256	12.475282	13.714414	11.382018	0.3058765	2.4932924	0.0280691	0.2510891	1.4226152	2.4932924	4.96E-30	2.46E-28	2.6993134	2.4932924	8.35E-113	5.84E-111
3006	ENSMUSG00	238328	Vash1	1	protein codi	MGI Symbol	1	vasohibin 1	166	144	114	119	139	146	239	254	260	377	449	405	2.9494246	2.5013698	2.0266103	1.9561695	2.2753109	2.5597906	3.2480934	3.0890664	3.7843395	5.7496105	6.0667703	5.81389	-0.141046	1.8368358	0.3002503	0.7609003	0.4330025	1.8368358	0.0005291	0.0023151	1.2372034	1.8368358	5.08E-25	4.44E-24
3016	ENSMUSG00	NA	Meg3	1	lincRNA	MGI Symbol	11	maternally e	2250.56	1448.42	1094.57	994.61	1827.39	1077.02	3602.04	3453.34	3743.18	4213.03	7052.43	7665.31	39.987091	25.159959	19.458481	16.349796	29.912809	18.88319	48.95298	41.998412	54.482554	64.252736	95.290586	110.0377	-0.376372	5.5609029	7.31E-05	0.0020532	0.7810966	5.5609029	5.73E-17	1.51E-15	1.6722073	5.5609029	1.80E-70	5.99E-69
3041	ENSMUSG00	218038	Amph	1	protein codi	MGI Symbol	6	amphiphysir	4056	3809	4016	4139	4146	4164	8867	9826	7757	10866	12791	11680	72.06546	66.164706	71.39357	68.038535	67.866468	73.006631	120.50562	119.50066	112.90431	165.71689	172.82864	167.66972	-0.004947	6.7359284	0.9580542	1	0.7515516	6.7359284	2.01E-16	5.17E-15	1.271967	6.7359284	2.14E-43	3.93E-42
3076	ENSMUSG00	15904	Id4	1	protein codi	MGI Symbol	1	inhibitor of I	229	172	198	294	249.98	241	503	507.97	406	549	707	646	4.0687846	2.9877473	3.5199021	4.8328894	4.0919584	4.2254078	6.8359455	6.1777679	5.9093918	8.372775	9.5527987	9.2735134	0.315226	2.5632719	0.008486	0.1110427	0.840003	2.5632719	1.43E-13	2.92E-12	1.3637954	2.5632719	2.94E-34	3.92E-33
308	ENSMUSG00	27419	Naglu	1	protein codi	MGI Symbol	3	alpha-N-ace	573	590	610	669	676	665	1218	1321	1026	1393	1742	1622	10.180845	10.248668	10.844143	10.997289	11.065541	11.65932	16.553045	16.065578	14.933586	21.244582	23.537448	23.284271	0.108526	3.9207307	0.2863284	0.7516196	0.6048601	3.9207307	9.89E-10	1.37E-08	1.1221808	3.9207307	3.22E-30	3.69E-29
3086	ENSMUSG00	78903	Wrnip1	1	protein codi	MGI Symbol	5	Werner helic	576	525.69	531.66	550.81	526.83	520.11	963.62	1015.14	756.74	1227.44	1466.75	1482.46	10.234148	9.1315632	9.4514705	9.0544348	8.6237557	9.1189911	13.095932	12.345807	11.014466	18.719634	19.818342	21.281134	-0.104872	3.6714619	0.3126182	0.7701781	0.3399466	3.6714619	0.0007251	0.0030108	1.053834	3.6714619	1.77E-26	1.70E-25
31072	ENSMUSG00	NA	Gm4524	1	antisense	MGI Symbol	1	predicted ge	327.96	300.25	304.51	353.63	370.45	345.78	507.17	487.52	423.86	938.46	983.13	920.83	5.8270681	5.2155298	5.4133605	5.8131112	6.0639491	6.0624959	6.8926173	5.9290616	6.1693468	14.312412	13.283795	13.218776	0.1245457	2.9858487	0.2651196	0.735798	0.2061703	2.9858487	0.0580604	0.1049018	1.3100256	2.9858487	1.93E-36	2.82E-35
3118	ENSMUSG00	17702	Msx2	1	protein codi	MGI Symbol	2	msh homeol	608	534	490	586	549	640	993	1231	871	1371	1693	1486	10.802712	9.2759131	8.7108688	9.632902	8.9866596	11.221	13.495216	14.971027	12.677537	20.909061	22.875372	21.331952	0.0508556	3.7900682	0.6220755	0.917943	0.5161867	3.7900682	2.46E-07	2.47E-06	1.1776635	3.7900682	9.18E-33	1.15E-31
31194	ENSMUSG00	71683	Gypc	1	protein codi	MGI Symbol	3	glycophorin	83	79	108	97	97	83	266	279	252	402	409	443	1.4747123	1.3722793	1.9199466	1.5945247	1.5878069	1.4552234	3.6150328	3.3931084	3.6678983	6.1308844	5.5263008	6.3593908	-0.03742	1.7098613	0.8355895	0.9788296	1.161923	1.7098613	3.48E-18	1.02E-16	1.9170299	1.7098613	1.04E-50	2.30E-49
3122	ENSMUSG00	13039	Ctsl	1	protein codi	MGI Symbol	7	cathepsin L	5924.98	6112	6258.87	6138	6494.72	6186.06	10443.75	10876.74	8290.16	13872.6	18769.76	18676.91	105.27278	106.16925	111.26571	100.8989	106.313	108.45903	141.93421	132.27942	120.66454	211.57042	253.61208	268.11235	-0.031827	7.2027097	0.7267446	0.9504507	0.2912331	7.2027097	0.0013225	0.0049485	1.1842057	7.2027097	2.85E-38	4.49E-37
3124	ENSMUSG00	26919	Zfp346	1	protein codi	MGI Symbol	13	zinc finger p	251	225	213	285	265	280	403	491	390	573	724	590	4.4596722	3.9083903	3.7865614	4.6849438	4.337823	4.9091875	5.4769106	5.9713843	5.6765093	8.738798	9.7824983	8.4696175	0.1962186	2.5669411	0.0949225	0.4835194	0.4952951	2.5669411	1.15E-05	8.14E-05	1.1514628	2.5669411	1.29E-25	1.16E-24
3181	ENSMUSG00	12380	Cast	1	protein codi	MGI Symbol	13	calpastatin [	555.27	528.58	595.8	680.5	724.61	641.86	908.21	1070.92	899.3	1518.9	1946.92	1810.41	9.8658254	9.1817643	10.591705	11.186331	11.861245	11.253611	12.342891	13.024185	13.089448	23.164678	26.306273	25.98895	0.2110046	3.8981104	0.0382401	0.3107625	0.3758559	3.8981104	0.0001735	0.0008855	1.3485378	3.8981104	9.50E-43	1.70E-41
31863	ENSMUSG00	170676	Peg10	1	protein codi	MGI Symbol	3	paternally ex	317	307	258	221	276	224	406	507	438	763	1006	884	5.632335	5.3327815	4.5865391	3.6328863	4.5178835	3.92735	5.5176817	6.1659711	6.3751566	11.63648	13.592808	12.690071	-0.364205	2.8183278	0.0016331	0.0304803	0.2152358	2.8183278	0.0498609	0.0928235	1.2861551	2.8183278	1.09E-34	1.49E-33
319	ENSMUSG00	12336	Capns1	1	protein codi	MGI Symbol	8	calpain, sma	7170	7033	7086	8264	8151	7990	13114	13876	11477	16308	19855	19484	127.39382	122.1676	125.96983	135.84693	133.42489	140.08717	178.22384	168.75545	167.04948	248.7126	268.27556	279.69835	0.1244195	7.4489542	0.1699054	0.6268354	0.4529177	7.4489542	5.76E-07	5.42E-06	1.0850937	7.4489542	1.37E-32	1.72E-31
32	ENSMUSG00	12444	Ccnd2	1	protein codi	MGI Symbol	8	cyclin D2 [Sc	11961.31	9834.54	10520.89	8909.12	9028.17	9397.69	17863.57	17976.28	16381.82	33540.92	36161.92	33143.01	212.52399	170.83209	187.03284	146.45167	147.78341	164.76794	242.77215	218.62174	238.43988	511.53111	488.61039	475.77732	-0.313467	8.0617849	0.0005298	0.0116228	0.2950417	8.0617849	0.0010774	0.0041714	1.3715966	8.0617849	1.10E-50	2.43E-49
32004	ENSMUSG00	NA	Neat1	1	lincRNA	MGI Symbol	4	nuclear para	257	234.1	278	363	373	359	608.63	787	581	1057.67	1878.88	1657.3	4.5662779	4.066463	4.9420848	5.967139	6.1056904	6.2942797	8.2714941	9.5712412	8.4565434	16.130479	25.386934	23.791012	0.4361822	3.3822002	0.0001129	0.0030044	0.9554992	3.3822002	1.36E-18	4.13E-17	2.2675618	3.3822002	1.80E-100	9.81E-99
3224	ENSMUSG00	15212	Hexb	1	protein codi	MGI Symbol	2	hexosaminic	1015.77	864.56	1069.56	1224.74	1187.14	1139.52	1937.62	1949.4	1637.22	2724.39	3166.61	2994.2	18.047814	15.017946	19.013871	20.132765	19.432465	19.978991	26.332932	23.707977	23.829986	41.549553	42.786405	42.982591	0.1934105	4.7093438	0.0457055	0.3403169	0.5043925	4.7093438	1.31E-07	1.37E-06	1.2898242	4.7093438	1.04E-41	1.80E-40
3250	ENSMUSG00	218442	Serinc5	1	protein codi	MGI Symbol	3	serine incor	86	78	63	72	86	98	267	298	228	506	531	497	1.5280152	1.3549087	1.1199689	1.1835648	1.4077463	1.7182156	3.6286232	3.6241803	3.3185747	7.7169839	7.1747329	7.1345761	0.1020987	1.815188	0.5476512	0.891083	1.4009925	1.815188	2.37E-24	9.52E-23	2.4576718	1.815188	4.18E-80	1.62E-78
3268	ENSMUSG00	13723	Emb	1	protein codi	MGI Symbol	6	embigin [Sou	4046.34	2580.18	4525.78	5163.32	5233.62	5167.55	7356.52	6708.54	5161.45	9122.97	14237.14	11289.57	71.893824	44.819336	80.456073	84.876716	85.669875	90.601685	99.977674	81.587108	75.12569	139.13402	192.3685	162.06498	0.4055363	6.6554392	9.34E-06	0.0003323	0.3807892	6.6554392	3.07E-05	0.0001949	1.3240823	6.6554392	1.02E-46	2.04E-45
3277	ENSMUSG00	19270	Ptprg	1	protein codi	MGI Symbol	8	protein tyros	690	589	865	779	823	785	1849	2143	1903	3221	3560	3721	12.259657	10.231297	15.37735	12.805513	13.471805	13.763258	25.128555	26.062478	27.698454	49.12333	48.101787	53.416012	0.0806989	4.6869914	0.420906	0.8431152	1.0589548	4.6869914	8.43E-28	3.83E-26	1.9922693	4.6869914	2.14E-93	1.04E-91
3285	ENSMUSG00	16195	Il6st	1	protein codi	MGI Symbol	10	interleukin 6	2243	2229	2198	2411	2495	2473	3443	3975	3388	5558	6447	6247	39.852768	38.71912	39.074469	39.632981	40.841012	43.358645	46.791571	48.342673	49.312855	84.764815	87.110175	89.677459	0.0738357	5.7560247	0.4274477	0.8463211	0.2960527	5.7560247	0.0013648	0.0050826	1.1526358	5.7560247	1.98E-35	2.78E-34
3289	ENSMUSG00	408066	BC067074	1	protein codi	MGI Symbol	3	cDNA seque	288	196	335	249	279	287	399	558	475	584	714	724	5.117074	3.4046423	5.9553899	4.0931614	4.566991	5.0319172	5.4225492	6.7862167	6.9136972	8.9065585	9.6473809	10.393226	-0.080371	2.684234	0.498196	0.8711141	0.4027614	2.684234	0.0002471	0.0012028	1.0010814	2.684234	7.87E-21	5.39E-20
33193	ENSMUSG00	52898	Rnasek	1	protein codi	MGI Symbol	3	ribonuclease	932.68	909.35	900.02	894.14	969.8	895.56	1764.19	1850.81	1592.74	2202.85	2588.1	2503.29	16.571502	15.795977	15.999911	14.69823	15.874795	15.701686	23.975958	22.508957	23.182573	33.595569	34.969729	35.935439	-0.063847	4.4916381	0.5168565	0.8789762	0.5262948	4.4916381	4.12E-08	4.69E-07	1.1114242	4.4916381	3.23E-31	3.87E-30
3320	ENSMUSG00	108671	Dnajc9	1	protein codi	MGI Symbol	4	DnaJ heat sh	184	190	200	210	209	192	352.18	343	303	425	522.82	480	3.2692418	3.3004185	3.5554567	3.4520639	3.4211509	3.3663	4.7862491	4.1714558	4.4102111	6.4816564	7.0642068	6.8905363	0.0165168	2.1960281	0.9209089	0.9921337	0.3997255	2.1960281	0.0006754	0.0028543	1.0135626	2.1960281	6.90E-19	4.17E-18
3321	ENSMUSG00	11750	Anxa7	1	protein codi	MGI Symbol	11	annexin A7 [	474	465	541	480	510	528	884	968	753	1227	1510	1529	8.421851	8.07734	9.6175103	7.8904317	8.348263	9.257325	12.013868	11.772505	10.96003	18.712923	20.402724	21.949229	-0.035199	3.629333	0.7436451	0.9550667	0.4124702	3.629333	4.69E-05	0.0002815	1.2256615	3.629333	7.22E-35	9.92E-34
3327	ENSMUSG00	22330	Vcl	1	protein codi	MGI Symbol	3	vinculin [Sou	332.15	270.36	355.05	439.96	464.08	431.46	517.03	639.63	476.65	1250.14	1769.9	1810.88	5.9015144	4.6963218	6.3118244	7.2322382	7.596592	7.5647073	7.0266181	7.7789746	6.9377132	19.065831	23.914425	25.995697	0.4056399	3.4514272	0.0001892	0.004767	0.3645395	3.4514272	0.0007022	0.0029322	2.0291179	3.4514272	8.58E-88	3.69E-86
3330	ENSMUSG00	50527	Ero1l	1	protein codi	MGI Symbol	6	ERO1-like (S	674	675	710	730	738	742	1288	1406	1141	1503	1879	1917	11.975375	11.725171	12.621871	12.000032	12.080428	13.009347	17.504369	17.09932	16.607429	22.922187	25.388556	27.519079	0.0299094	4.0698372	0.768884	0.961343	0.4957236	4.0698372	4.20E-07	4.05E-06	1.0621134	4.0698372	9.45E-28	9.62E-27
334	ENSMUSG00	216558	Ugp2	1	protein codi	MGI Symbol	4	UDP-glucose	894	810	934	1010	1058	983	1624	1866	1609	2169	2317	2218	15.884251	14.070205	16.603983	16.602783	17.318554	17.234755	22.070727	22.693693	23.41924	33.079324	31.306697	31.84002	0.1359705	4.4548608	0.1646992	0.6196605	0.5503928	4.4548608	1.04E-08	1.29E-07	1.0473431	4.4548608	8.59E-28	8.77E-27
3357	ENSMUSG00	71704	Arhgef3	1	protein codi	MGI Symbol	4	Rho guanine	43	47	53	70	68	65	229	306	280	869	1002	1081	0.7640076	0.8164193	0.942196	1.150688	1.1131017	1.1396328	3.1121899	3.7214737	4.0754426	13.253081	13.538761	15.518062	0.4312537	2.3436823	0.0194651	0.1986238	2.1092209	2.3436823	9.93E-46	8.66E-44	4.0651826	2.3436823	7.03E-198	1.45E-195
3359	ENSMUSG00	26363	Btd	1	protein codi	MGI Symbol	2	biotinidase [	239	234	241	248	260	251.77	420.65	439.34	387	662	758	765	4.2464608	4.064726	4.2843253	4.076723	4.2559772	4.4142362	5.7167803	5.3431119	5.6328439	10.096133	10.241897	10.981792	0.0168911	2.6303874	0.8934163	0.9874875	0.4055626	2.6303874	0.0003259	0.0015234	1.3139915	2.6303874	1.37E-33	1.79E-32
33637	ENSMUSG00	100862072	Gm21451	1	unprocessec	MGI Symbol	1	predicted ge	150.87	62.04	129.99	90.49	87.49	80.74	698.74	708.1	639.94	1106.75	1693.14	1479.6	2.6806006	1.0776735	2.3108691	1.4875108	1.4321363	1.4155993	9.4961204	8.6116847	9.314424	16.878996	22.877264	21.240078	-0.48128	3.0732196	0.0013154	0.025502	2.1770655	3.0732196	1.18E-73	2.04E-71	3.3319064	3.0732196	6.51E-176	1.08E-173
3370	ENSMUSG00	239017	Ogdhl	1	protein codi	MGI Symbol	3	oxoglutarate	407	351	338	347	296	307	587	543	478	899	1100	1003	7.2314206	6.0970889	6.0087218	5.7041246	4.8452664	5.3825735	7.9775348	6.6037915	6.9573627	13.71061	14.862912	14.39835	-0.279383	3.0692189	0.0114997	0.1384042	0.1549202	3.0692189	0.1455529	0.2182561	1.1522973	3.0692189	2.66E-29	2.90E-28
3371	ENSMUSG00	76485	Glt8d1	1	protein codi	MGI Symbol	10	glycosyltran	649.29	605.79	705.4	783.71	826.38	809.44	1199.19	1256.64	1009.11	1391.15	1845.05	1840.89	11.536337	10.52295	12.540096	12.882938	13.527133	14.19176	16.297411	15.282852	14.68775	21.216368	24.929832	26.426499	0.2306939	4.022377	0.0210514	0.2081045	0.4195637	4.022377	2.08E-05	0.0001381	1.0691768	4.022377	6.36E-28	6.56E-27
3372	ENSMUSG00	69019	Spcs1	1	protein codi	MGI Symbol	7	signal peptic	1620.71	1685.21	1792.6	1930.29	1875.62	1849.56	3285.81	3579.36	3135.89	4467.85	5725.95	5306.11	28.796157	29.273149	31.867558	31.730878	30.702292	32.427989	44.655304	43.531026	45.643356	68.138985	77.367536	76.170716	0.0768526	5.4960318	0.4141885	0.8384383	0.5733079	5.4960318	7.53E-10	1.06E-08	1.3015638	5.4960318	5.34E-44	1.00E-42
33810	ENSMUSG00	NA	Gm10359	1	processed p	MGI Symbol	1	predicted ge	1432.88	1551.38	1371.95	1578.11	1717.76	1612.48	2831.18	2290.47	1741.58	2767.73	4420.62	4831.79	25.458865	26.948438	24.389544	25.941602	28.118259	28.27131	38.476724	27.85596	25.348962	42.210529	59.730259	69.361718	0.1002358	5.1401512	0.2899033	0.7545724	0.255598	5.1401512	0.0065225	0.018246	1.1576832	5.1401512	5.93E-35	8.18E-34
34712	ENSMUSG00	NA	CAAA01147:	1	protein codi	Clone-based	1		311	302	376	316	323	299	520	533	437	707	946	795	5.5257293	5.2459284	6.6842585	5.1945342	5.2872333	5.242311	7.0669814	6.4821748	6.3606015	10.782426	12.782104	11.412451	-0.150297	2.8897616	0.1798646	0.6411685	0.1901304	2.8897616	0.0786794	0.1340005	1.003699	2.8897616	6.25E-22	4.62E-21
3473	ENSMUSG00	20410	Sorbs3	1	protein codi	MGI Symbol	4	sorbin and S	84	72	97	58	59	65	234	260	211	848	897	893	1.4924799	1.2506849	1.7243965	0.9534272	0.9657794	1.1396328	3.1801416	3.1620365	3.0711371	12.932811	12.120029	12.819269	-0.547109	2.2314159	0.0010863	0.021544	1.0759198	2.2314159	3.25E-15	7.56E-14	3.0827789	2.2314159	8.49E-136	8.86E-134
3475	ENSMUSG00	213053	Slc39a14	1	protein codi	MGI Symbol	13	solute carrie	506	430	456	442	444	486	714	895	730	1303	1685	1486	8.9904148	7.4693682	8.1064412	7.2657725	7.2678996	8.5209469	9.7035091	10.884702	10.625261	19.871996	22.767278	21.331952	-0.092297	3.5842374	0.3868413	0.821768	0.3459852	3.5842374	0.0007183	0.0029849	1.3812027	3.5842374	1.42E-43	2.62E-42

Table S2. Differentially expressed genes (> 2-fold) in *Trpm3* -mutant lenses summarized in Fig. 9A.

3479	ENSMUSG00	12153	Bmp1	1	protein codi	MGI Symbol	7	bone morph	3059	2664	2956	2908	2857	2834	4995	5453	4508	7379	10708	9814	54.351144	46.275342	52.54965	47.802865	46.766642	49.687991	67.883793	66.317634	65.614626	112.53681	144.68369	140.88276	-0.086546	6.2234696	0.3490232	0.7945607	0.3835358	6.2234696	2.95E-05	0.0001887	1.378105	6.2234696	4.96E-50	1.08E-48
3484	ENSMUSG00	14586	Gfra2	1	protein codi	MGI Symbol	3	glial cell line	130	77	91	293	242	254	534	610	388	1910	2277	1895	2.3097904	1.337538	1.6177328	4.816451	3.9613327	4.4533344	7.2572463	7.418624	5.647399	29.129327	30.766227	27.203263	1.3297774	3.4134091	2.91E-24	6.42E-22	1.9517761	3.4134091	2.14E-55	2.51E-53	4.0483595	3.4134091	1.95E-249	7.12E-247
34868	ENSMUSG00	NA	Gm12671	1	processed p	MGI Symbol	1	predicted ge	1517.9	1598.82	1542.35	1606.52	1667.94	1655.17	2660.01	2283.19	1822.86	2825.26	4024.47	5013.55	26.969468	27.772501	27.418793	26.408617	27.302749	29.019785	36.150464	27.767423	26.532005	43.087917	54.377584	71.970934	0.0098073	5.1488292	0.9190087	0.9915359	0.1386484	5.1488292	0.1396891	0.2113468	1.0444082	5.1488292	6.43E-29	6.90E-28
3487	ENSMUSG00	16432	Itm2b	1	protein codi	MGI Symbol	5	integral men	5533	5392	5832	6251	6450	5850	10575	10606	8538	13448	16973	16141	98.308232	93.662403	103.67712	102.75643	105.58097	102.56695	143.71794	128.98676	124.27189	205.09486	229.33473	231.70864	0.0726164	7.1213928	0.4249566	0.8449287	0.425198	7.1213928	2.84E-06	2.31E-05	1.1719783	7.1213928	1.75E-37	2.68E-36
3497	ENSMUSG00	13618	Ednrb	1	protein codi	MGI Symbol	3	endothelin r	2907	2151	2563	3067	3353	3044	3967	3692	3935	5452	6921	6532	51.650466	37.364212	45.563177	50.416571	54.885737	53.369881	53.912914	44.900918	57.274524	83.148214	93.514738	93.768715	0.2377024	5.9080884	0.0101049	0.1263114	0.2135799	5.9080884	0.0203828	0.0456474	1.0069973	5.9080884	1.15E-27	1.17E-26
34975	ENSMUSG00	64009	Syne1	1	protein codi	MGI Symbol	9	spectrin rep	690	965	595	732	924	909	1555	2619	2039	3595	4051	4820	12.259657	16.762652	10.577484	12.032908	15.125088	15.937327	21.132993	31.851437	29.677955	54.827188	54.73605	69.192469	0.1209037	4.849176	0.2211278	0.7007052	1.0614899	4.849176	4.21E-28	1.96E-26	2.173701	4.849176	9.58E-111	6.36E-109
3498	ENSMUSG00	64929	Scel	1	protein codi	MGI Symbol	2	sciellin [Sour	137	130	91	168	157	181	259	289	212	274	356	309	2.4341637	2.2581811	1.6177328	2.7616511	2.5699555	3.1734391	3.5199004	3.5147252	3.0856922	4.178762	4.8101787	4.4357827	0.4278052	1.7059327	0.0013463	0.0258157	0.6825802	1.7059327	1.10E-07	1.17E-06	1.0893983	1.7059327	4.57E-18	2.62E-17
3500	ENSMUSG00	211286	Cln5	1	protein codi	MGI Symbol	2	ceroid-lipofu	780	697	831	845	867	784	1412	1469	1272	1714	2007	2027	13.858742	12.107325	14.772922	13.890447	14.192047	13.745725	19.189573	17.865506	18.514153	26.140139	27.118058	29.098161	0.0383625	4.20623	0.7034606	0.9437707	0.4478396	4.20623	4.08E-06	3.22E-05	1.0156732	4.20623	7.56E-26	6.95E-25
3507	ENSMUSG00	105559	Mbnl2	1	protein codi	MGI Symbol	10	muscleblind	819	789	798	895	1014	1033	1475	1733	1386	2168	2595	2553	14.551679	13.705422	14.186272	14.712367	16.598311	18.111395	20.045765	21.076189	20.173441	33.064073	35.06296	36.64904	0.2191824	4.4321763	0.0254923	0.2367321	0.5304502	4.4321763	4.24E-08	4.80E-07	1.3037515	4.4321763	8.75E-42	1.52E-40
35331	ENSMUSG00	12581	Cdkn2d	1	protein codi	MGI Symbol	3	cyclin depen	220	195	207	238	244	206	378	398	367	490	645	546	3.908876	3.3872716	3.6798977	3.9123391	3.9940709	3.6117594	5.1371519	4.8403481	5.3417408	7.4729686	8.715071	7.8379851	0.0707063	2.3863439	0.5686456	0.8979609	0.4798019	2.3863439	3.11E-05	0.0001969	1.1312484	2.3863439	5.35E-24	4.38E-23
35441	ENSMUSG00	NA	Gm5559	1	processed p	MGI Symbol	1	predicted ge	451.5	501.72	541.46	414.82	630.77	626.1	880.79	880.77	675.79	1205.52	1600.43	1691.6	8.0220796	8.7151893	9.6256878	6.8189768	10.325164	10.977294	11.970243	10.711642	9.8362262	18.385333	21.624591	24.283398	0.0919515	3.6658941	0.3746609	0.812109	0.3031483	3.6658941	0.0028578	0.0092565	1.2864611	3.6658941	2.30E-38	3.63E-37
3564	ENSMUSG00	20356	Sema5a	1	protein codi	MGI Symbol	9	sema domai	12510	11190	13955	12314	14186	12856	21319	26635	22216	30443	36580	36285	222.2729	194.37728	248.08199	202.42245	232.21267	225.40184	289.73265	323.92631	323.35726	464.28486	494.25937	520.88148	-0.010216	8.2848006	0.910091	0.9905825	0.4953208	8.2848006	4.08E-08	4.65E-07	1.1542127	8.2848006	8.93E-37	1.33E-35
35741	ENSMUSG00	NA	Snhg18	1	lincRNA	MGI Symbol	1	small nucleo	222	209	196	223	275	253	416	389	346	455	549	546	3.9444112	3.6304604	3.4843475	3.6657631	4.5015144	4.4358016	5.6535852	4.730893	5.0360826	6.9391851	7.4179441	7.8379851	0.187842	2.3712203	0.1155086	0.5316531	0.4783735	2.3712203	3.03E-05	0.0001928	1.0051522	2.3712203	3.80E-19	2.32E-18
3580	ENSMUSG00	114128	Laptm4b	1	protein codi	MGI Symbol	4	lysosomal-a	1525	1429	1392.99	1671	1563	1604	2540	2707	2386	3739	3997	4048	27.095618	24.822621	24.763578	27.468565	25.584971	28.122631	34.519486	32.921664	34.728593	57.023326	54.006417	58.11019	0.0820678	5.1639484	0.3868137	0.821768	0.4138646	5.1639484	1.02E-05	7.36E-05	1.1411676	5.1639484	5.12E-34	6.77E-33
3587	ENSMUSG00	17909	Myo10	1	protein codi	MGI Symbol	13	myosin X [Sc	189	199	179	192	195	184	226	338	290	774	943	950	3.3580798	3.4567541	3.1821337	3.1561727	3.1919829	3.2260375	3.0714189	4.1106474	4.2209941	11.80424	12.741569	13.63752	-0.062658	2.5499716	0.6377884	0.923451	0.1897925	2.5499716	0.1153645	0.1819025	1.9329062	2.5499716	9.30E-68	2.96E-66
360	ENSMUSG00	27061	Bcap31	1	protein codi	MGI Symbol	1	B cell recept	1027	1086	1025	1158	1176	1202	2090	2090	1647	2316	2853	2709	18.247344	18.864497	18.221715	19.035666	19.250112	21.074441	28.403829	25.417909	23.972336	35.321215	38.548988	38.888464	0.1010276	4.6738794	0.2955887	0.7583646	0.4915562	4.6738794	2.46E-07	2.47E-06	1.0270542	4.6738794	3.15E-27	3.13E-26
36072	ENSMUSG00	NA	Rian	1	processed t	MGI Symbol	69	RNA imprint	1204	892	747	554	693	604	2409	2209	2201	3578	5240	5377	21.392212	15.494596	13.279631	9.1068733	11.343816	10.589819	32.739151	26.865148	32.03589	54.567922	70.801507	77.188362	-0.69246	4.9745468	3.36E-12	2.85E-10	0.8687412	4.9745468	8.62E-20	2.78E-18	2.0137777	4.9745468	2.48E-97	1.26E-95
36243	ENSMUSG00	NA	Gpr137b-ps	1	transcribed	MGI Symbol	8	G protein-co	4362.57	4002.38	4350.29	4280.92	4257.71	4142.37	7088.7	7877.9	6003.7	9404.96	11363.24	10927.45	77.512478	69.523837	77.336338	70.371473	69.695065	72.627397	96.337906	95.808489	87.384767	143.43463	153.53712	156.86665	-0.077117	6.609207	0.3994969	0.8293321	0.3172226	6.609207	0.0005012	0.0022064	1.0163247	6.609207	1.38E-28	1.47E-27
3685	ENSMUSG00	170736	Parvb	1	protein codi	MGI Symbol	4	parvin, beta	173	162	183	189	190	207	530	637	496	1234	1428	1401	3.073798	2.814041	3.2532429	3.1068575	3.1101372	3.6292922	7.2028849	7.7469893	7.2193554	18.81968	19.294762	20.111753	0.1059768	3.0742727	0.4139237	0.8380807	1.2788063	3.0742727	6.16E-29	2.96E-27	2.6709506	3.0742727	1.25E-126	1.07E-124
3687	ENSMUSG00	72709	C1qtnf6	1	protein codi	MGI Symbol	5	C1q and tum	468	460	524	507	481	520	805	828	648	1241	1507	1354	8.3152453	7.9904869	9.3152965	8.3342685	7.8735579	9.1170625	10.940231	10.06987	9.4317386	18.926437	20.362189	19.437055	-0.017251	3.5554357	0.8775384	0.9850516	0.2492381	3.5554357	0.0147928	0.035274	1.1969048	3.5554357	3.36E-33	4.30E-32
3690	ENSMUSG00	17886	Myh9	1	protein codi	MGI Symbol	9	myosin, hea	3644.04	2729.78	3159.43	3168.52	3650.64	3560.87	4913.28	5940.06	5113.41	9006.81	10798.16	11044.31	64.745912	47.417981	56.166082	52.085397	59.757849	62.432066	66.77319	72.241101	74.42646	137.36247	145.90191	158.54421	0.0500521	6.3794464	0.5869625	0.9065075	0.3425945	6.3794464	0.0001847	0.0009295	1.3922135	6.3794464	3.92E-51	8.82E-50
36963	ENSMUSG00	239217	Kctd12	1	protein codi	MGI Symbol	2	potassium cl	2171	2278	2250	1954	2314	1979	4878	5792	4640	6692	8504	6645	38.573499	39.570281	39.998888	32.120632	37.878197	34.697436	66.293722	70.440443	67.535906	102.0594	114.90382	95.390862	-0.174287	5.9484135	0.0615768	0.3941672	0.7899826	5.9484135	1.28E-17	3.54E-16	1.4026859	5.9484135	2.77E-51	6.27E-50
3708	ENSMUSG00	29858	Pmm1	1	protein codi	MGI Symbol	11	phosphomar	219	223	230	282	249	252	410	490	379	461	604	614	3.8911084	3.8736491	4.0887752	4.6356286	4.0759167	4.4182688	5.5720431	5.9592226	5.5164026	7.0306909	8.1610897	8.8141444	0.1473768	2.4796711	0.210077	0.6855684	0.5251668	2.4796711	3.42E-06	2.73E-05	1.0187888	2.4796711	4.57E-20	2.98E-19
3724	ENSMUSG00	56722	Litaf	1	protein codi	MGI Symbol	5	LPS-induced	310	283	344	327	362	363	652	878	557	1633	2196	1729	5.5079616	4.9158865	6.1153855	5.3753566	5.9256298	6.364411	8.8609075	10.677954	8.1072197	24.904812	29.671777	24.820286	0.0946007	3.5732087	0.3984526	0.8286486	0.7438882	3.5732087	2.13E-12	3.95E-11	2.2636552	3.5732087	7.96E-109	5.21E-107
3728	ENSMUSG00	13731	Emp2	1	protein codi	MGI Symbol	2	epithelial me	106	115	129	203	200	178	321	357	231	973	1252	1009	1.8833675	1.9976217	2.2932696	3.3369951	3.2738286	3.1208406	4.362502	4.3417193	3.3622401	14.83918	16.916696	14.484482	0.6566423	2.6570736	7.38E-07	3.28E-05	0.9697482	2.6570736	2.03E-14	4.47E-13	2.9043619	2.6570736	6.16E-134	6.23E-132
3740	ENSMUSG00	14167	Fgf12	1	protein codi	MGI Symbol	6	fibroblast gr	91	116	96	103	94	107	414	373	358	1563	2061	1889	1.6168533	2.0149924	1.7066192	1.6931551	1.5386995	1.8760109	5.6264045	4.5363062	5.2107445	23.837245	27.847692	27.117131	-0.066645	3.1515038	0.6989087	0.9427938	1.5219675	3.1515038	3.02E-33	1.68E-31	3.881715	3.1515038	1.30E-230	3.64E-228
3743	ENSMUSG00	15205	Hes1	1	protein codi	MGI Symbol	4	hes family b	222	190	193	199	223	242	354	327	254	571	713	526	3.9444112	3.3004185	3.4310157	3.2712415	3.6503189	4.2429406	4.8109835	3.9768689	3.6970086	8.708296	9.6338692	7.5508794	0.0629664	2.3471351	0.6114622	0.9139633	0.2265736	2.3471351	0.0544673	0.09968	1.2787863	2.3471351	3.03E-30	3.48E-29
3765	ENSMUSG00	223658	Mroh1	1	protein codi	MGI Symbol	15	maestro hea	331	280	345	298	345	332.14	544	590	545	1482	1910	1644	5.8810816	4.8637746	6.1331628	4.898643	5.6473544	5.8233484	7.3931498	7.1753904	7.9325579	22.601917	25.807419	23.600087	-0.044308	3.4277338	0.7004323	0.9430688	0.4142581	3.4277338	0.0001083	0.000588	2.0935408	3.4277338	1.98E-93	9.68E-92
3770	ENSMUSG00	66168	Grina	1	protein codi	MGI Symbol	5	glutamate re	3144	2761	3013	3156	3219	3128	5013	5328	4731	7131	7866	7635	55.861392	47.960292	53.562955	51.879588	52.692272	54.842638	68.128419	64.797424	68.860425	108.75457	106.28333	109.60259	0.0184858	6.1365695	0.8422103	0.9794342	0.358483	6.1365695	9.33E-05	0.0005155	1.0445569	6.1365695	8.71E-30	9.76E-29
3771	ENSMUSG00	18810	Plec	1	protein codi	MGI Symbol	22	plectin [Sour	17888	12585.66	13849	13819	17794	16851	21582	28403	25792	35019	40970.98	39186.09	317.82715	218.62076	246.1976	227.16224	291.27253	295.44542	293.30691	345.42816	375.40648	534.07324	553.58915	562.52745	0.0564491	8.4723239	0.5310946	0.8841481	0.3738274	8.4723239	3.35E-05	0.0002109	1.0762202	8.4723239	2.52E-32	3.10E-31
3784	ENSMUSG00	17069	Ly6e	1	protein codi	MGI Symbol	16	lymphocyte	133	99	102	101	103	106	289	291	255	339	514	470	2.3630932	1.7196917	1.8132829	1.6602783	1.6860218	1.8584781	3.9276108	3.5390485	3.7115638	5.1700742	6.9450333	6.7469835	-0.180305	1.8175571	0.2160458	0.6933902	0.9220528	1.8175571	6.85E-13	1.34E-11	1.6798106	1.8175571	3.47E-42	6.13E-41
3792	ENSMUSG00	11838	Arc	1	protein codi	MGI Symbol	2	activity regu	2	1	2	1	2	8	325	309	216	828	1150	851	0.0355352	0.0173706	0.0355546	0.0164384	0.0327383	0.1402625	4.4168634	3.7579587	3.1439129	12.627792	15.538499	12.216347	1.0128157	2.1730888	0.2136265	0.6905376	6.9163311	2.1730888	5.07E-134	2.10E-131	8.7462553	2.1730888	7.62E-305	4.02E-302
3800	ENSMUSG00	12651	Chkb	1	protein codi	MGI Symbol	9	choline kina	230.69	194.84	209.11	198.91	255.43	195.48	334.27	391.05	349.89	530.75	730.32	690.16	4.0988118	3.3844923	3.7174077	3.269762	4.1811703	3.4273142	4.5428459	4.7558245	5.0927022	8.0944451	9.8678925	9.9074428	-0.040771	2.4440772	0.7511513	0.958233	0.3610862	2.4440772	0.0017507	0.0062196	1.3162883	2.4440772	1.99E-32	2.47E-31
3832	ENSMUSG00	67203	Nde1	1	protein codi	MGI Symbol	8	nudE neuroc	345	345	316	347	324	337	609	608	539	769	918	930	6.1298283	5.9928652	5.6176215	5.7041246	5.3036024	5.9085578	8.2765225	7.3943007	7.845227	11.727985	12.403775	13.350414	-0.069194	3.0090335	0.5405507	0.888141	0.4057639	3.0090335	0.0001336	0.0007071	1.0791326	3.0090335	2.46E-25	2.18E-24
3835	ENSMUSG00	18203	Ntan1	1	protein codi	MGI Symbol	9	N-terminal A	196.87	234.19	149	233.76	235.55	194.46	389.67	323.6	384.02	401.03	532.89	497.28	3.497911	4.0680264	2.6488152	3.8426402	3.8557517	3.4094307	5.2957513	3.9355192	5.5894695	6.116091	7.20027	7.1385956	0.1209376	2.2586853	0.3282587	0.7793548	0.5308458	2.2586853	4.53E-06	3.53E-05	1.0015904	2.2586853	1.52E-18	8.97E-18
3896	ENSMUSG00	51789	Tnk2	1	protein codi	MGI Symbol	24	tyrosine kina	868	726	768	808	845	845	1486	1736	1629	2574	3160	2990	15.422293	12.611073	13.652954	13.282227	13.831926	14.815227	20.195259	21.112674	23.710343	39.255962	42.697092	42.922299	0.0082398	4.5176121	0.9376787	0.9963741	0.6409393	4.5176121	3.41E-11	5.58E-10	1.5830226	4.5176121	1.45E-60	4.06E-59
3899	ENSMUSG00	22042	Tfrc	1	protein codi	MGI Symbol	7	transferrin r	695	671	649	775	985	930	1639	1567	1134	2432	2433	2596	12.348495	11.655689	11.537457	12.73976	16.123606	16.305516	22.274582	19.057351	16.505542	37.090326	32.874058	37.266317	0.3454015	4.3635085	0.0005019	0.0111412	0.7029045	4.3635085	6.46E-13	1.27E-11	1.5927883	4.3635085	2.39E-60	6.68E-59
3945	ENSMUSG00	110197	Dgkg	1	protein codi	MGI Symbol	5	diacylglycerc	669	685	693	924	965	853	2565	2854	2324	4119	5095	4805	11.886537	11.898877	12.319657	15.189081	15.796223	14.955489	34.859245	34.709431	33.826173	62.818689	68.842305	68.977139	0.3476716	5.0152641	0.0004556	0.0103063	1.5178319	5.0152641	3.68E-55	4.26E-53	2.474249	5.0152641	2.10E-140	2.35E-138
396	ENSMUSG00	11853	Rhoc	1	protein codi	MGI Symbol	6	ras homolog	1027	1071	839	1299	1209	1225	1502	1475	1282	2232	2860	3033	18.247344	18.603938	14.915141	21.353481	19.790294	21.477695	20.412704	17.938476	18.659705	34.040134	38.643571	43.539576	0.274354	4.5873822	0.0044715	0.0687359	0.1388258	4.5873822	0.1489503	0.2227338	1.1668005	4.5873822	1.86E-34	2.52E-33
3964	ENSMUSG00	11820	App	1	protein codi	MGI Symbol	11	amyloid beta	19357.98	17745.96	19338.99	21172.99	22610.98	21514.97	33990.96	35848.99	28063	46719.99	52964.97	47968.98	343.9452	308.25839	343.7947	348.05007	370.12237	377.21794	461.94901	435.9839	408.46123	712.52453	715.6488	688.60833	0.1372282	8.8443479	0.1272997	0.5504537	0.3913919	8.8443479	1.38E-05	9.55E-05	1.0876602	8.8443479	5.15E-33	6.55E-32
3977	ENSMUSG00	19128	Pros1	1	protein codi	MGI Symbol	3	protein S (al	290	269	271	343	359	331	590	563	690	980	1613	1380	5.1526093	4.6726978	4.8176438	5.638371	5.8765224	5.803361	8.0183059	6.8470251	10.043055	14.945937	21.794433	19.810292	0.2421229	3.2576395	0.0323595	0.2787081	0.7628134	3.2576395	1.16E-12	2.22E-11	1.9503154	3.2576395	4.92E-79	1.88E-77
3981	ENSMUSG00	14805	Grik1	1	protein codi	MGI Symbol	11	glutamate re	279	178	330	189	150	134	1811	1801	1306	6900	8409	7993	4.9571655	3.091971	5.8665035	3.1068575	2.4553715	2.3493969	24.612122	21.903183	19.009029	105.2316	113.6202	114.74178	-0.810996	5.1416192	3.19E-11	2.52E-09	2.2367633	5.1416192	2.19E-103	5.47E-101	4.5842365	5.1416192	0	0
3990	ENSMUSG00	54720	Rcan1	1	protein codi	MGI Symbol	7	regulator of	1166	1196	1021	1318	1140	1327	4541	4648	3533	6103	7391	7212	20.717043	20.775266	18.150606	21.66581	18.660823	23.266042	61.713774	56.527483	51.423352	93.076586	99.865255	103.53031	0.0920359	5.6231805	0.3379275	0.7875353	1.5082279	5.6231805	7.11E-57	8.66E-55	2.3133414	5.6231805	1.78E-127	1.58E-125
3997	ENSMUSG00	77975	Tmem50b	1	protein codi	MGI Symbol	3	transmembr	219	204	184	175	211	173	337	321	306	485	557	470	3.8911084	3.5436072	3.2710201	2.8767199	3.4538892	3.0331766	4.5799476	3.9038989	4.4538765	7.3967138	7.526038	6.7469835	-0.192546	2.2090208	0.1215987	0.5403338	0.2709598	2.2090208	0.0207519	0.0462777	1.0170445	2.2090208	2.23E-19	1.39E-18
4	ENSMUSG00	NA	H19	1	lincRNA	MGI Symbol	7	H19, imprint	1117	773	1133	828	762	598	6168	5116	6493	16455	17370	20481	19.84643	13.427492	20.141662	13.610995	12.473287	10.484622	83.825272	62.219148	94.506603	250.95449	234.69889	294.01057	-0.54532	6.5349966	2.90E-08	1.58E-06	2.1707884	6.5349966	1.35E-112	4.27E-110	3.8677493	6.5349966	9.32972419	5.539523741357e-314
4001	ENSMUSG00	15976	Ifnar2	1	protein codi	MGI Symbol	5	interferon (a	296	278	227	303	254	274	474	513	456	685	851	795	5.259215	4.8290334	4.0354433	4.980835	4.1577624	4.8039906	6.4418254	6.2389412	6.6371494	10.446905	11.498489	11.412451	-0.019219	2.7678102	0.8773121	0.9850516	0.4508353	2.7678102	4.01E-05	0.0002456	1.2399402	2.7678102	6.37E-31	7.55E-30
401	ENSMUSG00	18616	Peg3	1	protein codi	MGI Symbol	5	paternally ex	4922	3510	3979	4279	4379	4383	13618	16645	14750	21881	25392	26338	87.452217	60.970889	70.73581	70.339911	71.680478	76.846317	185.07337	202.43114	214.68849	333.70617	343.09005	378.08947	-0.001919	7.4492984	0.9842213	1	1.4582737	7.4492984	3.83E-56	4.60E-54	2.2670838	7.4492984	3.28E-127	2.83E-125
4021	ENSMUSG00	22379	Fmnl3	1	protein codi	MGI Symbol	5	formin-like 3	281	238	289	275	265	266	460	501	420	876	980	922	4.9927007	4.1342084	5.1376349	4.5205598	4.337823	4.6637281	6.2515605	6.093001	6.1131639	13.359838	13.241503	13.235572	-0.076967	2.8600937	0.5133567	0.8777849	0.3721425	2.8600937	0.000742	0.0030755	1.4818081	2.8600937	4.23E-45	8.17E-44
4022	ENSMUSG00	380969	Nckap5l	1	protein codi	MGI Symbol	4	NCK-associa	252	222.98	194.98	231	245.99	250.96	362	440	374	535.95	700	603	4.4774398	3.8733017	3.4662147	3.7972703	4.0266455	4.4000346	4.9197063	5.3511386	5.4436269	8.17375	9.4582166	8.6562362	0.0478499	2.4798921	0.6880868	0.9398213	0.4110715	2.4798921	0.0003083	0.0014551	1.1541639	2.4798921	1.42E-25	1.28E-24
4026	ENSMUSG00	26934	Racgap1	1	protein codi	MGI Symbol	14	Rac GTPase-	488.72	509.68	528.45	601	571	510.38	1182.72	1112.52	1091.99	1773	2551.5	2341.83	8.6833904	8.8534595	9.3944054	9.879478	9.3467808	8.9483969	16.073577	13.530111	15.894081	27.039946	34.475199	33.617635	0.0654872	4.0386057	0.5321878	0.8841481	0.7554713	4.0386057	3.95E-14	8.49E-13	1.8209732	4.0386057	7.81E-76	2.88E-74
4029	ENSMUSG00	14555	Gpd1	1	protein codi	MGI Symbol	3	glycerol-3-pl	2005.63	2231.58	2003.94	2548.35	2490.49	2437.35	4485.55	4996.42	4211.54	5594.97	7695.18	7080.65	35.635268	38.763937	35.624609	41.890795	40.767187	42.733601	60.96019	60.764855	61.299605	85.328643	103.97526	101.64474	0.1885176	5.8882916	0.0427284	0.3270288	0.7341308	5.8882916	2.21E-15	5.22E-14	1.4029932	5.8882916	3.64E-51	8.21E-50
4032	ENSMUSG00	65970	Lima1	1	protein codi	MGI Symbol	6	LIM domain	912	887	867	1017	989	948	2778	3053	2245	3944	4739	4624	16.204068	15.407743	15.412905	16.717852	16.189083	16.621106	37.753989	37.129605	32.676316	60.149772	64.032126	66.378833	0.0748083	5.0462457	0.4461591	0.8516723	1.1938768	5.0462457	9.88E-36	6.22E-34	2.0187204	5.0462457	2.25E-97	1.15E-95
4049	ENSMUSG00	66151	Prr13	1	protein codi	MGI Symbol	5	proline rich	267	222	236	267	224	264	433	461	351	625	752	665	4.7439541	3.8562785	4.1954389	4.3890526	3.6666881	4.6286625	5.8846211	5.6065339	5.1088584	9.5318477	10.160827	9.5462638	-0.013469	2.5897714	0.9203001	0.9921337	0.3767791	2.5897714	0.0008057	0.0032976	1.1926706	2.5897714	7.20E-28	7.39E-27
4071	ENSMUSG00	14199	Fhl1	1	protein codi	MGI Symbol	10	four and a h	879	828	820	968	879	840	1871	2044	1744	3462	4277	4226	15.617736	14.382876	14.577372	15.912371	14.388477	14.727563	25.427543	24.858471	25.384185	52.798811	57.789703	60.66543	0.014642	4.8164432	0.8864027	0.9860841	0.7632951	4.8164432	2.05E-15	4.87E-14	1.9417585	4.8164432	4.29E-90	1.89E-88
40851	ENSMUSG00	353237	Pcdhac2	1	protein codi	MGI Symbol	1	protocadher	225.57	297.74	250.75	288.69	262.48	267.97	623.73	578.58	480.72	1223.18	1507.71	1368.88	4.0078416	5.1719295	4.4576538	4.7456015	4.2965727	4.6982678	8.4767083	7.0365041	6.9969527	18.654665	20.371782	19.650661	0.0098909	3.1963222	0.9372671	0.9963741	0.7219372	3.1963222	3.67E-11	5.93E-10	2.1044313	3.1963222	5.71E-87	2.41E-85
4091	ENSMUSG00	105727	Slc38a1	1	protein codi	MGI Symbol	7	solute carrie	1954	1597	1894	1821.83	2025	1884.81	3011	3217	2532	4390	4993	5209.94	34.717926	27.740886	33.670175	29.947969	33.147515	33.04602	40.920541	39.12412	36.853645	66.951698	67.464108	74.790168	0.0002401	5.4357702	1	1	0.2824395	5.4357702	0.0024224	0.0080774	1.1219695	5.4357702	2.30E-33	2.96E-32
4096	ENSMUSG00	67776	Vwa5a	1	protein codi	MGI Symbol	7	von Willebra	3981	4075	1806	6150	6381	5917	7521	4170	7890	11480	13584	12924	70.732888	70.785292	32.105774	101.09616	104.4515	103.74165	102.21301	50.7142	114.84015	175.08098	183.54345	185.52769	0.832836	6.7546996	1.23E-19	2.01E-17	0.6240578	6.7546996	9.24E-12	1.62E-10	1.6479022	6.7546996	3.12E-70	1.03E-68
4102	ENSMUSG00	12258	Serping1	1	protein codi	MGI Symbol	3	serine (or cy	118	132	154	143	160	122	302	346	273	684	919	753	2.096579	2.2929223	2.7377016	2.3506911	2.6190629	2.1390031	4.1042854	4.2079408	3.9735565	10.431654	12.417287	10.809529	-0.001729	2.3581022	1	1	0.7862154	2.3581022	2.29E-10	3.37E-09	2.2397803	2.3581022	3.09E-82	1.24E-80
41057	ENSMUSG00	93706	Pcdhgc3	1	protein codi	MGI Symbol	3	protocadher	1386.94	1390.6	1430.39	1567.8	1515.44	1513.66	2482.94	2989.74	2312.02	3250.63	4013.32	3432.26	24.64262	24.155589	25.428448	25.772123	24.806454	26.538717	33.744021	36.360257	33.651803	49.575216	54.226928	49.271067	0.0550314	5.0920047	0.5633301	0.8961357	0.4834084	5.0920047	2.64E-07	2.64E-06	1.0443041	5.0920047	9.98E-29	1.06E-27
4108	ENSMUSG00	235587	Parp3	1	protein codi	MGI Symbol	9	poly (ADP-ril	187	193	165	271	232	249	522	459	453	1283	1825	1757	3.3225446	3.3525304	2.9332518	4.4548062	3.7976412	4.3656703	7.0941621	5.5822105	6.5934839	19.566977	24.658922	25.222234	0.3920894	3.2300521	0.0012066	0.0236344	1.0012428	3.2300521	2.58E-18	7.71E-17	2.8531796	3.2300521	3.84E-146	4.50E-144
4111	ENSMUSG00	109652	Acy1	1	protein codi	MGI Symbol	12	aminoacylas	155	166	148	166	199	161	308	346	315	411	545	506	2.7539808	2.8835235	2.6310379	2.7287743	3.2574595	2.8227828	4.1858275	4.2079408	4.5848729	6.268143	7.3638972	7.2637737	0.0917142	2.1135507	0.4961942	0.8711141	0.6486027	2.1135507	7.96E-08	8.68E-07	1.3378209	2.1135507	2.75E-30	3.17E-29
4143	ENSMUSG00	21991	Tpi1	1	protein codi	MGI Symbol	7	triosephospl	4995	4914.99	4885.67	5299.98	5302	5583.96	9352	9831.96	7869.97	12608	16960.88	15613.99	88.749253	85.376442	86.85394	87.123188	86.789197	97.902524	127.09694	119.57314	114.54861	192.28406	229.17097	224.14326	0.0586118	7.004576	0.5202678	0.8802983	0.4689584	7.004576	2.54E-07	2.55E-06	1.3067441	7.004576	7.54E-46	1.47E-44
4150	ENSMUSG00	67389	C1qtnf12	1	protein codi	MGI Symbol	3	C1q and tum	323	315	335	371	373	346	934	986	906	1482	1877	1716	5.7389407	5.4717465	5.9553899	6.0986462	6.1056904	6.0663531	12.693386	11.991415	13.186968	22.601917	25.361532	24.633667	0.0901477	3.6198772	0.419815	0.8419812	1.1408295	3.6198772	3.74E-28	1.76E-26	2.0804503	3.6198772	1.51E-92	7.23E-91
4166	ENSMUSG00	68262	Agpat4	1	protein codi	MGI Symbol	6	1-acylglycer	120	107	97	107	109	132	322	353	293	494	606	633	2.1321142	1.8586567	1.7243965	1.7589087	1.7842366	2.3143313	4.3760924	4.2930726	4.2646596	7.5339724	8.1881132	9.0868948	0.0320594	2.0768398	0.8334178	0.9785665	1.1776748	2.0768398	2.14E-20	7.15E-19	2.1175329	2.0768398	1.63E-67	5.14E-66
4169	ENSMUSG00	16004	Igf2r	1	protein codi	MGI Symbol	3	insulin-like g	3216	2258	2854	3189	3660	3161	12018	12121	12290	30563	33054	39699	57.14066	39.222868	50.736367	52.422056	59.911064	55.42122	163.32881	147.41171	178.88282	466.11498	446.61699	569.89042	0.1896943	7.5758276	0.0397208	0.3159869	1.7348484	7.5758276	5.13E-77	9.02E-75	3.3333531	7.5758276	2.27E-250	8.62E-248
4171	ENSMUSG00	240028	Lnpep	1	protein codi	MGI Symbol	6	leucyl/cystin	675	584	712	609	754	755	922	1137	843	1486	1695	1760	11.993142	10.144444	12.657426	10.010985	12.342334	13.237273	12.530302	13.827829	12.269993	22.662921	22.902396	25.2653	0.0322026	3.9124598	0.7519679	0.958233	0.1518652	3.9124598	0.1279166	0.1975041	1.0256884	3.9124598	7.94E-26	7.26E-25
4174	ENSMUSG00	23984	Pde10a	1	protein codi	MGI Symbol	14	phosphodies	2543	2408	2600	2464	2895	2557	4459	5010	4043	9237	10679	10640	45.183053	41.828462	46.220937	40.504216	47.38867	44.831402	60.599366	60.93001	58.84648	140.87308	144.29185	152.74022	-0.005489	6.2056409	0.9541997	1	0.4371385	6.2056409	2.08E-06	1.74E-05	1.7166815	6.2056409	2.76E-75	1.00E-73
4212	ENSMUSG00	22339	Vegfa	1	protein codi	MGI Symbol	10	vascular end	1579	1244	1286	1624	1801	1705	2098	2541	1917	2851	3740	3911	28.055069	21.609056	22.861586	26.695961	29.480827	29.893445	28.512552	30.902826	27.902227	43.480476	50.5339	56.143516	0.2470134	5.048001	0.0090819	0.116736	0.2682277	5.048001	0.0044378	0.0133204	1.0501807	5.048001	5.55E-29	5.97E-28
4216	ENSMUSG00	83965	Enpp5	1	protein codi	MGI Symbol	3	ectonucleoti	608	596	542	636	651	648	932	1048	805	1276	1753	1649	10.802712	10.352892	9.6352876	10.454822	10.656312	11.361263	12.666205	12.745439	11.716897	19.46022	23.686077	23.671863	0.0763353	3.8088923	0.4554054	0.8562065	0.2704847	3.8088923	0.0069152	0.0191084	1.1181199	3.8088923	6.07E-30	6.84E-29
4217	ENSMUSG00	224794	Enpp4	1	protein codi	MGI Symbol	2	ectonucleoti	633	608	609	635	686	649	966	1066	918	1379	1747	1610	11.246902	10.561339	10.826366	10.438384	11.229232	11.378795	13.128277	12.96435	13.36163	21.031069	23.605006	23.112007	0.0179498	3.8564877	0.8643784	0.9821164	0.2736113	3.8564877	0.0059487	0.01693	1.0540408	3.8564877	5.72E-27	5.60E-26
4225	ENSMUSG00	71461	Ptk7	1	protein codi	MGI Symbol	2	PTK7 protein	527	484	588	475	531	518	994	1071	835	1605	1961	1686	9.3635348	8.4073819	10.453043	7.8082397	8.692015	9.0819969	13.508807	13.025158	12.153552	24.477785	26.496518	24.203009	-0.141959	3.8149637	0.1728808	0.6310968	0.4555368	3.8149637	5.67E-06	4.29E-05	1.4136865	3.8149637	1.54E-46	3.06E-45
4254	ENSMUSG00	21785	Tff2	1	protein codi	MGI Symbol	1	trefoil factor	108	109	125	101	90	93	279	334	304	640	1209	922	1.9189028	1.893398	2.2221604	1.6602783	1.4732229	1.6305516	3.7917074	4.0620007	4.4247662	9.760612	16.335691	13.235572	-0.340853	2.4161202	0.0213047	0.2101705	1.0234187	2.4161202	7.20E-16	1.78E-14	2.7059242	2.4161202	5.29E-115	3.90E-113
4265	ENSMUSG00	17691	Sik1	1	protein codi	MGI Symbol	4	salt inducibl	473	362	452	499	514	501	614	693	594	1044	1141	1073	8.4040834	6.2881658	8.0353321	8.2027613	8.4137396	8.7839391	8.3444742	8.4280434	8.6457603	15.921998	15.416893	15.40322	0.160599	3.3344643	0.1286434	0.5536637	0.16171	3.3344643	0.1210614	0.1887549	1.0404886	3.3344643	8.01E-25	6.90E-24
4266	ENSMUSG00	268970	Arhgap28	1	protein codi	MGI Symbol	5	Rho GTPase	278	229	239	208	240	266	457	468	390	527	696	666	4.9393979	3.9778728	4.2487707	3.4191871	3.9285944	4.6637281	6.2107895	5.6916656	5.6765093	8.037254	9.4041696	9.5606191	-0.134091	2.5582038	0.2662668	0.73627	0.4171571	2.5582038	0.0001843	0.0009282	1.037289	2.5582038	2.05E-21	1.47E-20
4278	ENSMUSG00	77889	Lbh	1	protein codi	MGI Symbol	2	limb-bud an	197	204	253	182	193	183	941	1009	844	2207	2549	2392	3.5002208	3.5436072	4.4976527	2.9917887	3.1592446	3.2085047	12.788518	12.271134	12.284548	33.658861	34.44142	34.337839	-0.302145	3.7620172	0.0140127	0.159617	1.6939165	3.7620172	2.01E-54	2.19E-52	3.1496387	3.7620172	7.15E-184	1.31E-181
4304	ENSMUSG00	65960	Twsg1	1	protein codi	MGI Symbol	4	twisted gast	1532	1490	1772	1672	1575	1656	3907	3891	3322	5825	6713	6180	27.219991	25.882229	31.501346	27.485004	25.781401	29.034338	53.097493	47.321092	48.352215	88.83682	90.704297	88.715655	-0.039906	5.6085255	0.6746783	0.9358476	0.8142823	5.6085255	2.71E-18	8.08E-17	1.6648612	5.6085255	6.93E-70	2.28E-68
4311	ENSMUSG00	58226	Cacna1h	1	protein codi	MGI Symbol	10	calcium char	345.94	299	272	314.98	340	363	463.94	512	474	942	1186	1103.96	6.1465298	5.1938165	4.8354211	5.177767	5.5655087	6.364411	6.3051065	6.2267795	6.8991421	14.366401	16.024921	15.847659	0.0796788	3.0583088	0.4783656	0.8649415	0.2634638	3.0583088	0.0152037	0.0360244	1.5155029	3.0583088	1.59E-48	3.29E-47
4331	ENSMUSG00	104215	Rhoq	1	protein codi	MGI Symbol	3	ras homolog	1001.18	878.51	1051.37	1076.81	1040.78	969.01	1305.12	1456.04	1149.8	2210.18	2819.42	2680.04	17.788584	15.260267	18.690502	17.701033	17.036677	16.989471	17.737036	17.707891	16.735514	33.707359	38.095264	38.472735	-8.59E-05	4.4746794	1	1	0.0126989	4.4746794	0.9005424	0.9276896	1.0921019	4.4746794	2.22E-30	2.57E-29
4364	ENSMUSG00	66905	Plin3	1	protein codi	MGI Symbol	2	perilipin 3 [S	359	293	335	402	347	323	571	585	521	771	977	967	6.3785749	5.0895928	5.9553899	6.6082366	5.6800927	5.6630985	7.7600892	7.1145821	7.5832342	11.758487	13.200968	13.88156	0.044019	3.0240337	0.7014239	0.9430758	0.3658258	3.0240337	0.0006139	0.0026329	1.157264	3.0240337	6.85E-29	7.33E-28
43686	ENSMUSG00	NA	Gm43737	1	TEC	MGI Symbol	1	predicted ge	158.66	140.62	157.77	156.02	158.63	173.33	240.04	277.49	216.42	373.48	386.12	456.36	2.8190103	2.4426571	2.804722	2.5647191	2.5966372	3.0389624	3.2622274	3.3747442	3.150026	5.6959272	5.2171553	6.5511774	0.0224371	1.882952	0.8877342	0.9860841	0.2805305	1.882952	0.0255317	0.0544202	1.1136712	1.882952	7.07E-21	4.86E-20
4411	ENSMUSG00	106957	Slc39a6	1	protein codi	MGI Symbol	4	solute carrie	728	674	730	808	798	726	1379	1296	1184	1530	1953	1902	12.934826	11.7078	12.977417	13.282227	13.062576	12.728822	18.741091	15.761536	17.2333	23.333963	26.388424	27.30375	0.0549572	4.1048706	0.5860342	0.9062982	0.4592228	4.1048706	2.62E-06	2.14E-05	1.0341822	4.1048706	1.71E-26	1.64E-25
4423	ENSMUSG00	225164	Mib1	1	protein codi	MGI Symbol	6	mindbomb E	2094	1761	2230	2597	3028	2849	2946	3484	2906	4324	5105	5753	37.205393	30.589668	39.643342	42.690523	49.565766	49.950983	40.037168	42.371289	42.297272	65.945135	68.977422	82.585949	0.4044986	5.6260366	1.35E-05	0.000459	0.2151542	5.6260366	0.0204527	0.0457691	1.0176921	5.6260366	6.03E-28	6.23E-27
4432	ENSMUSG00	21356	Tapbp	1	protein codi	MGI Symbol	9	TAP binding	787.03	647.37	812.38	723.13	712.01	684.14	1468.74	1451.7	1177.03	2020.67	2352.39	2103.75	13.983649	11.245221	14.441909	11.8871	11.654994	11.994898	19.960689	17.655109	17.131851	30.81715	31.784877	30.199929	-0.158503	4.2219629	0.1129932	0.5262207	0.4650183	4.2219629	1.80E-06	1.53E-05	1.22633	4.2219629	6.92E-37	1.04E-35
4441	ENSMUSG00	13506	Dsc2	1	protein codi	MGI Symbol	4	desmocollin	381	370	375	449	499	471	842	809	736	964	1121	1056	6.7694625	6.4271308	6.6664813	7.3808413	8.1682025	8.2579547	11.443074	9.8387981	10.712592	14.701922	15.146658	15.15918	0.2609969	3.3422519	0.0145764	0.1637182	0.6869303	3.3422519	3.11E-11	5.11E-10	1.1800684	3.3422519	7.27E-31	8.56E-30
4454	ENSMUSG00	81500	Sil1	1	protein codi	MGI Symbol	11	endoplasmic	673	630	732	809	799	802	1558	1607	1307	2039	2604	2660	11.957607	10.943493	13.012971	13.298665	13.078945	14.061316	21.173764	19.543818	19.023584	31.0967	35.184566	38.185055	0.1711109	4.3333205	0.0867898	0.4682017	0.7343381	4.3333205	5.39E-14	1.14E-12	1.5406865	4.3333205	1.23E-56	3.17E-55
4465	ENSMUSG00	30948	Bin1	1	protein codi	MGI Symbol	6	bridging inte	772	746.88	793	888	860	863	1733	1929.89	1740.29	2742.74	3671.62	3413.21	13.716601	12.973771	14.097386	14.597299	14.077463	15.130817	23.552075	23.470702	25.330186	41.829408	49.609967	48.997599	0.1028558	4.6385971	0.2994084	0.760326	0.8266477	4.6385971	1.18E-17	3.29E-16	1.7839042	4.6385971	4.64E-76	1.72E-74
4499	ENSMUSG00	14815	Nr3c1	1	protein codi	MGI Symbol	9	nuclear rece	271	306	335	278	384	377	444	503	357	686	817	779	4.8150245	5.3154109	5.9553899	4.569875	6.285751	6.6098703	6.0341149	6.1173244	5.1961893	10.462156	11.03909	11.182766	0.1173683	2.8129335	0.293318	0.7559743	0.1107982	2.8129335	0.3200565	0.4114393	1.0228585	2.8129335	2.50E-22	1.89E-21
451	ENSMUSG00	21803	Tgfb1	1	protein codi	MGI Symbol	5	transforming	543	688	661	584	618	565	925	1157	736	1722	2474	2022	9.6478167	11.950989	11.750784	9.6000252	10.116131	9.9060391	12.571073	14.071062	10.712592	26.262147	33.42804	29.026384	-0.171139	3.9867088	0.0934372	0.4818966	0.1652272	3.9867088	0.0990747	0.1613318	1.4117479	3.9867088	1.93E-47	3.88E-46
4538	ENSMUSG00	66286	Sec11c	1	protein codi	MGI Symbol	11	SEC11 homo	519	497	533	564	623	527	1080	1042	926	2921	4388	4452	9.2213939	8.6332	9.475292	9.2712573	10.197976	9.2397922	14.677577	12.672469	13.478071	44.548043	59.289506	63.909724	0.0714343	4.4724911	0.4941667	0.8711141	0.5786572	4.4724911	7.80E-09	9.79E-08	2.6179522	4.4724911	7.31E-153	8.68E-151
4584	ENSMUSG00	70417	Megf10	1	protein codi	MGI Symbol	2	multiple EGF	638	570	472	663	693	696	3069	3534	2978	5466	6680	6589	11.33574	9.9012555	8.3908777	10.898659	11.343816	12.202838	41.708781	42.979373	43.345243	83.361727	90.25841	94.586966	0.2168924	5.2690828	0.0328196	0.2811416	2.1112549	5.2690828	3.09E-102	7.53E-100	3.1781097	5.2690828	4.03E-220	1.01E-217
4587	ENSMUSG00	14119	Fbn2	1	protein codi	MGI Symbol	6	fibrillin 2 [Sc	88	52	66	70	73	85	312	562	642	959	861	1044	1.5635504	0.9032724	1.1733007	1.150688	1.1949475	1.4902891	4.2401889	6.8348634	9.3444076	14.625667	11.633606	14.986916	0.0738025	2.5646678	0.6874196	0.9398213	2.4848619	2.5646678	2.96E-79	5.30E-77	3.5010804	2.5646678	5.66E-165	8.54E-163
4598	ENSMUSG00	18596	Pdgfrb	1	protein codi	MGI Symbol	2	platelet deri	703	623	481	964	920	1018	947	1084	1051	1254	1733	1524	12.490636	10.821899	8.5508733	15.846617	15.059612	17.848403	12.87006	13.18326	15.297465	19.124699	23.415842	21.877453	0.6129928	3.9627559	7.16E-10	4.88E-08	0.3750322	3.9627559	0.0001577	0.0008151	1.0159746	3.9627559	4.55E-25	3.99E-24
46	ENSMUSG00	23947	Mid2	1	protein codi	MGI Symbol	5	midline 2 [Sc	218	172	195	243	258	222	351	456	359	603	736	719	3.8733408	2.9877473	3.4665703	3.9945311	4.2232389	3.8922844	4.7702125	5.5457255	5.2252996	9.1963266	9.9446391	10.321449	0.2307377	2.5125511	0.0577013	0.3843054	0.5910505	2.5125511	3.14E-07	3.10E-06	1.5129524	2.5125511	7.62E-42	1.32E-40
4617	ENSMUSG00	56473	Fads2	1	protein codi	MGI Symbol	3	fatty acid de	530	498	595	619	591	583	1052	1118	959	1932	2019	1995	9.4168377	8.6505706	10.577484	10.175369	9.6741636	10.22163	14.297047	13.596757	13.958391	29.464848	27.280199	28.638792	0.0701871	3.9632406	0.4982533	0.8711141	0.5469984	3.9632406	4.37E-08	4.92E-07	1.5755613	3.9632406	1.31E-57	3.45E-56
4618	ENSMUSG00	72982	Tmem138	1	protein codi	MGI Symbol	5	transmembr	246.18	214.95	254.95	239.56	212.11	228.72	457.18	472.26	313.75	581.01	629.51	647.31	4.3740323	3.7338156	4.5323184	3.937983	3.472059	4.0101049	6.2132357	5.7434744	4.566679	8.8609581	8.5057742	9.2923189	-0.146623	2.5059499	0.2218746	0.7019009	0.3892304	2.5059499	0.0005531	0.0024079	1.0765321	2.5059499	9.27E-23	7.17E-22
46364	ENSMUSG00	NA	Gm44850	1	antisense	MGI Symbol	1	predicted ge	27	13	19	10	23	14	265	320	246	1944	2767	2980	0.4797257	0.2258181	0.3377684	0.164384	0.3764903	0.2454594	3.6014425	3.8917372	3.5805674	29.647859	37.386979	42.778746	-0.39907	3.3824517	0.2288777	0.7091949	3.4056801	3.3824517	7.29E-83	1.38E-80	6.7134486	3.3824517	0	0
4641	ENSMUSG00	20409	Ostf1	1	protein codi	MGI Symbol	5	osteoclast st	176	235	248	295	312	279	498	567	498	768	973	952	3.1271008	4.0820966	4.4087663	4.8493278	5.1071727	4.8916547	6.7679938	6.8956718	7.2484657	11.712734	13.146921	13.66623	0.3536675	2.8598137	0.002444	0.0428383	0.8469492	2.8598137	3.54E-14	7.65E-13	1.7292467	2.8598137	1.41E-56	3.63E-55
4654	ENSMUSG00	13194	Ddb1	1	protein codi	MGI Symbol	11	damage spe	10812	10012	10779.99	10893	11466	11141.97	22733	25066.98	21602	30966	35357.99	33687	192.10349	173.91468	191.63894	179.06348	187.6886	195.35007	308.9494	304.85656	314.4204	472.26111	477.7479	483.58645	0.011445	8.1811565	0.8994884	0.9893473	0.7350941	8.1811565	4.79E-16	1.20E-14	1.3621916	8.1811565	4.97E-50	1.08E-48
4660	ENSMUSG00	83921	Cemip2	1	protein codi	MGI Symbol	5	cell migratio	943	817	886	1164	1229	1197	2167	2219	1863	4936	6140	5361	16.754864	14.1918	15.750673	19.134297	20.117677	20.986777	29.450286	26.986765	27.116248	75.27872	82.962071	76.958677	0.3672862	5.1543439	0.0001538	0.0039392	0.8393828	5.1543439	1.98E-18	5.94E-17	2.3325702	5.1543439	8.70E-128	7.80E-126
4685	ENSMUSG00	16452	Jak2	1	protein codi	MGI Symbol	4	Janus kinase	181	165	198	247	245	224	226	247	233	358	527	461	3.2159389	2.8661529	3.5199021	4.0602846	4.0104401	3.92735	3.0714189	3.0039346	3.3913504	5.4598424	7.1206859	6.6177859	0.3220075	2.0831917	0.0087455	0.113655	-0.022025	2.0831917	0.8780749	0.9109656	1.0018063	2.0831917	4.16E-18	2.39E-17
47	ENSMUSG00	217069	Trim25	1	protein codi	MGI Symbol	3	tripartite mc	203	170	165	188	177	184	231	294	219	413	552	553	3.6068265	2.953006	2.9332518	3.0904191	2.8973383	3.2260375	3.1393706	3.5755335	3.1875783	6.298645	7.4584794	7.938472	-0.043519	2.0899142	0.7507317	0.958233	0.0635086	2.0899142	0.6232355	0.6991896	1.1934957	2.0899142	2.12E-25	1.89E-24
4701	ENSMUSG00	21873	Tjp2	1	protein codi	MGI Symbol	7	tight junctio	343	367	458	367	338	328	620	708	630	994	1228	1214	6.0942931	6.3750189	8.1419958	6.0328926	5.5327704	5.7507625	8.4260163	8.6104685	9.1697458	15.159451	16.592414	17.427315	-0.250943	3.2528105	0.0215451	0.2116629	0.346134	3.2528105	0.0009151	0.0036541	1.254966	3.2528105	4.75E-35	6.59E-34
4703	ENSMUSG00	67457	Frmd8	1	protein codi	MGI Symbol	4	FERM domai	363	328.46	269.73	369	292.77	313.92	657.95	608	598.82	928.14	1433.55	1161.87	6.4496454	5.7055551	4.7950666	6.0657694	4.7923941	5.5039005	8.9417701	7.3943007	8.7159162	14.155023	19.369752	16.678974	-0.051168	3.193858	0.6553701	0.9286055	0.5617866	3.193858	1.17E-07	1.24E-06	1.5672724	3.193858	2.14E-52	4.91E-51
4717	ENSMUSG00	12660	Chka	1	protein codi	MGI Symbol	14	choline kina	1273	991	1079	1156	1350	1272	2060	2316	1995	3011	3221.92	2828	22.618178	17.214288	19.181689	19.00279	22.098343	22.301738	27.996119	28.166448	29.037528	45.920629	43.533739	40.596743	0.1034595	4.8191407	0.282555	0.749253	0.5298418	4.8191407	2.35E-08	2.76E-07	1.1399579	4.8191407	2.53E-33	3.25E-32
4722	ENSMUSG00	18739	Pitpnm1	1	protein codi	MGI Symbol	13	phosphatidy	203	166	202	210	200	187	432	410	380	1066	1115	1082	3.6068265	2.8835235	3.5910112	3.4520639	3.2738286	3.2786359	5.8710307	4.9862883	5.5309578	16.257519	15.065588	15.532417	-0.010062	2.8186547	0.9519988	1	0.6999112	2.8186547	1.36E-09	1.86E-08	2.2162994	2.8186547	1.91E-89	8.28E-88
4772	ENSMUSG00	17826	Fam89b	1	protein codi	MGI Symbol	3	family with s	535.9	459.11	477.71	487.76	577.2	501.62	931.91	1017.24	894.05	1102.75	1354.35	1276.25	9.5216666	7.9750271	8.492386	8.0179937	9.4482695	8.7948094	12.664982	12.371346	13.013034	16.817992	18.299622	18.320931	0.0152427	3.5927964	0.8913072	0.9874875	0.5497551	3.5927964	5.01E-08	5.58E-07	1.0403276	3.5927964	2.08E-25	1.85E-24
4773	ENSMUSG00	16998	Ltbp3	1	protein codi	MGI Symbol	12	latent transf	1555.79	1416.3	1563.42	1502.76	1529.73	1467.33	2005.59	2089.33	1649.48	3239.12	4164.65	3811.83	27.642683	24.602014	27.79336	24.702969	25.040369	25.726422	27.256668	25.40976	24.008432	49.399678	56.271659	54.719902	-0.084767	5.0349798	0.3719113	0.809429	-0.061692	5.0349798	0.5129787	0.5996428	1.030384	5.0349798	9.14E-27	8.88E-26
4783	ENSMUSG00	26379	Esrra	1	protein codi	MGI Symbol	6	estrogen rela	162	194	140	163	176	184	274	349	279	359	535	378	2.8783542	3.369901	2.4888197	2.6794591	2.8809692	3.2260375	3.7237556	4.2444259	4.0608874	5.4750933	7.2287798	5.4262973	0.0056534	2.0166092	0.9645108	1	0.4611284	2.0166092	0.0001344	0.000711	1.054215	2.0166092	2.47E-19	1.53E-18
4787	ENSMUSG00	18797	Plcb3	1	protein codi	MGI Symbol	10	phospholipa	563	503	511	527	519	491	668	664	593	1233	1556	1495	10.003169	8.7374237	9.0841918	8.6630365	8.4955853	8.608611	9.0783531	8.0753547	8.6312052	18.804429	21.024264	21.46115	-0.110675	3.5595975	0.288317	0.752955	-0.110366	3.5595975	0.2846792	0.3750453	1.1396103	3.5595975	1.35E-30	1.58E-29
4792	ENSMUSG00	104383	Rcor2	1	protein codi	MGI Symbol	4	REST corepr	206	149	149	153	175	167	295	330	254	346	451	468	3.6601294	2.5882229	2.6488152	2.5150751	2.8646001	2.9279797	4.0091529	4.013354	3.6970086	5.2768309	6.0937938	6.7182729	-0.099016	1.9956241	0.4564821	0.8562065	0.3991621	1.9956241	0.0009666	0.003819	1.0250459	1.9956241	2.07E-18	1.21E-17
48052	ENSMUSG00	NA	Gm10252	1	TEC	MGI Symbol	1	predicted ge	589.82	520.93	582.56	597.43	566.1	546.46	920.14	835.77	727.02	1275.02	1703.01	1520.39	10.479697	9.048879	10.356334	9.8207929	9.266572	9.5809807	12.505024	10.164366	10.581887	19.445274	23.010625	21.82563	-0.059743	3.7097587	0.5641931	0.8963418	0.1537206	3.7097587	0.1274118	0.196911	1.1055736	3.7097587	3.61E-29	3.91E-28
481	ENSMUSG00	16450	Jag2	1	protein codi	MGI Symbol	10	jagged 2 [So	179	163	132	185	182	168	294	364	290	833	1045	845	3.1804037	2.8314117	2.3466014	3.0411039	2.9791841	2.9455125	3.9955626	4.4268511	4.2209941	12.704047	14.119766	12.130215	0.1012304	2.5486546	0.4453188	0.851213	0.5975604	2.5486546	7.50E-07	6.91E-06	2.2200566	2.5486546	3.71E-85	1.52E-83
48326	ENSMUSG00	NA	Gm42047	1	lincRNA	MGI Symbol	2	predicted ge	241.23	47.98	234.5	172.71	258.34	265.53	476.29	679.63	313.3	1072.05	1344.05	1722.35	4.2860825	0.8334425	4.1687729	2.839076	4.2288045	4.6554877	6.4729473	8.2654417	4.5601292	16.349788	18.160451	24.724823	0.3372238	3.0757911	0.0065163	0.0914553	1.0650533	3.0757911	3.16E-20	1.05E-18	2.6764999	3.0757911	2.19E-127	1.93E-125
4837	ENSMUSG00	17161	Maoa	1	protein codi	MGI Symbol	1	monoamine	243	255	255	255	298	264	497	582	430	736	787	822	4.3175312	4.4295091	4.5332073	4.1917918	4.8780047	4.6286625	6.7544034	7.078097	6.2587154	11.224704	10.633738	11.800043	0.0445984	2.7689268	0.7139977	0.9457583	0.5984046	2.7689268	6.20E-08	6.87E-07	1.3410252	2.7689268	2.14E-35	2.99E-34
485	ENSMUSG00	14248	Flii	1	protein codi	MGI Symbol	4	flightless I ac	3062	2763	2921	3781	3636	3615	4995	6149	4911	7345	8369	7479	54.404447	47.995033	51.927445	62.153588	59.518205	63.381117	67.883793	74.782162	71.480352	112.01827	113.07973	107.36317	0.2619369	6.2078885	0.0044229	0.0680992	0.4727084	6.2078885	2.61E-07	2.61E-06	1.1072127	6.2078885	3.46E-33	4.42E-32
4850	ENSMUSG00	20874	Slk	1	protein codi	MGI Symbol	6	STE20-like ki	955	840	896	1025	1111	1083	1337	1624	1279	2067	2286	2436	16.968075	14.591324	15.928446	16.849359	18.186118	18.988036	18.170297	19.750566	18.61604	31.523727	30.887833	34.969472	0.1859093	4.4167048	0.0563462	0.3794882	0.2520617	4.4167048	0.0091041	0.0237803	1.036091	4.4167048	2.76E-27	2.74E-26
48517	ENSMUSG00	NA	Gpx4-ps2	1	transcribed	MGI Symbol	2	glutathione	519.49	575.55	597.69	586.44	598.8	576.07	967.44	1061.21	832.91	1231.78	1738.19	1464.52	9.2301	9.9976625	10.625304	9.6401349	9.801843	10.100127	13.147847	12.906095	12.123132	18.785823	23.485968	21.0236	-0.015295	3.7539911	0.8881239	0.9860841	0.3551216	3.7539911	0.0003892	0.0017817	1.0847446	3.7539911	3.96E-28	4.11E-27
4853	ENSMUSG00	14873	Gsto1	1	protein codi	MGI Symbol	6	glutathione S	288	245	291	308	293	262	507	572	473	1015	1622	1253	5.117074	4.2558028	5.1731895	5.063027	4.796159	4.5935969	6.8903069	6.9564802	6.8845869	15.479721	21.916039	17.987171	-0.008219	3.1494733	0.9400964	0.9971487	0.5116567	3.1494733	2.70E-06	2.21E-05	1.9300984	3.1494733	2.89E-77	1.09E-75
4861	ENSMUSG00	226250	Afap1l2	1	protein codi	MGI Symbol	8	actin filamer	192	173	149	234	190	213	317	301	208	420	536	504	3.4113827	3.0051179	2.6488152	3.8465855	3.1101372	3.7344891	4.3081406	3.6606653	3.0274716	6.4054016	7.2422915	7.2350631	0.2370715	2.1270278	0.0558935	0.377292	0.2806559	2.1270278	0.02151	0.0476218	1.2042864	2.1270278	1.76E-25	1.58E-24
4876	ENSMUSG00	18453	P4hb	1	protein codi	MGI Symbol	3	prolyl 4-hydi	2917	2883	3395	3609	3640	3400	5580	5915	4890	6712	8829	8819	51.828142	50.079508	60.353877	59.326183	59.583681	59.611563	75.834147	71.93633	71.174694	102.36442	119.29513	126.59925	0.1378131	6.243318	0.1342637	0.5634628	0.4322837	6.243318	2.42E-06	2.00E-05	1.1019457	6.243318	6.06E-33	7.69E-32
4877	ENSMUSG00	192662	Arhgdia	1	protein codi	MGI Symbol	2	Rho GDP dis	6506	6087	6564	6938	6801	6645	11538	12309	10353	14149	17394	16570	115.59612	105.73499	116.69009	114.04961	111.32654	116.50554	156.80545	149.6981	150.68949	215.78578	235.02317	237.86706	0.0163825	7.2502116	0.8573772	0.98209	0.4356934	7.2502116	1.55E-06	1.34E-05	1.0267431	7.2502116	2.32E-29	2.54E-28
48985	ENSMUSG00	NA	Gm49759	1	TEC	MGI Symbol	1	predicted ge	213.07	110.16	104.26	183.11	219.18	232.54	183.53	171.51	159.86	440.76	483.74	501.27	3.7857464	1.9135479	1.8534596	3.0100353	3.5877888	4.0770802	2.4942367	2.0858495	2.3267866	6.7220115	6.5361681	7.1958732	0.4981539	1.9439459	8.63E-05	0.0023764	-0.130173	1.9439459	0.3319281	0.423833	1.4377829	1.9439459	7.78E-34	1.03E-32
4899	ENSMUSG00	80879	Slc16a3	1	protein codi	MGI Symbol	8	solute carrie	868	877	730.54	870	884.46	990.57	2011.4	2092.76	1540.51	2687.67	3635.21	3802.31	15.422293	15.234037	12.987017	14.301407	14.477852	17.367478	27.335628	25.451475	22.422357	40.989538	49.118005	54.58324	0.0797029	4.6968095	0.4175735	0.8400992	0.7853531	4.6968095	3.40E-16	8.59E-15	1.7291544	4.6968095	4.57E-72	1.57E-70
49	ENSMUSG00	74617	Scpep1	1	protein codi	MGI Symbol	1	serine carbo	728	710	696	835	890	941	1434	1651	1353	2993	3429	2623.99	12.934826	12.333143	12.372989	13.726063	14.568537	16.498377	19.48856	20.078932	19.693121	45.646112	46.331749	37.668122	0.2504234	4.5061693	0.0114472	0.1383568	0.6548263	4.5061693	1.78E-11	3.03E-10	1.784096	4.5061693	1.53E-75	5.62E-74
4927	ENSMUSG00	20250	Scd2	1	protein codi	MGI Symbol	2	stearoyl-Coe	42128.78	40620.7	43442.75	44361.77	47338.67	45345.79	64075.64	69860.45	60675.63	104927.12	116006.66	117253.25	748.52808	705.6069	772.29408	729.23649	774.89347	795.03924	870.81031	849.62035	883.14302	1600.2389	1567.4516	1683.2037	0.0463788	9.9635071	0.6054431	0.9119565	0.2257588	9.9635071	0.0119182	0.0296551	1.1235184	9.9635071	3.63E-35	5.06E-34
4932	ENSMUSG00	94280	Sfxn3	1	protein codi	MGI Symbol	6	sideroflexin	173	120	133	130	146	116	227	253	201	460	613	577	3.073798	2.0844748	2.3643787	2.1369919	2.3898949	2.0338063	3.0850092	3.0769047	2.9255856	7.0154399	8.2826954	8.2829989	-0.194874	1.9925307	0.1502714	0.5929212	0.2743053	1.9925307	0.03174	0.0646922	1.649675	1.9925307	1.03E-44	1.97E-43
4949	ENSMUSG00	15211	Hexa	1	protein codi	MGI Symbol	1	hexosaminic	1563.94	1439.89	1372.84	1908.89	1885.94	1903.9	2748.88	2383.91	2607.97	3699.59	4115.89	4006.98	27.787489	25.011787	24.405366	31.379096	30.871222	33.380722	37.358238	28.992348	37.9594	56.422285	55.612827	57.521336	0.3086705	5.2212025	0.0010589	0.0210888	0.4333879	5.2212025	3.74E-06	2.97E-05	1.1349596	5.2212025	1.11E-33	1.45E-32

Table S2. Differentially expressed genes (> 2-fold) in *Trpm3*-mutant lenses summarized in Fig. 9A.

4974	ENSMUSG00	286940	Flnb	1	protein codi	MGI Symbol	2	filamin, beta	2393	2164	2350	3329	3832	3436	3273	3813	3717	5180	6454	6675	42.51791	37.59003	41.776616	54.723432	62.726557	60.242744	44.481212	46.372481	54.1015	78.999954	87.204757	95.821521	0.543883	5.8812717	4.15E-09	2.58E-07	0.2498542	5.8812717	0.0068241	0.0189281	1.1042933	5.8812717	9.70E-33	1.22E-31
4977	ENSMUSG00	20229	Sat1	1	protein codi	MGI Symbol	7	spermidine/	125	108	131	135	132	132	234	284	233	384	755	717	2.2209523	1.8760274	2.3288241	2.2191839	2.1607269	2.3143313	3.1801416	3.4539168	3.3913504	5.8563672	10.201362	10.292739	0.0591059	2.0795545	0.7004788	0.9430688	0.6425844	2.0795545	5.62E-07	5.30E-06	2.0388623	2.0795545	2.49E-65	7.59E-64
498	ENSMUSG00	17936	Nab1	1	protein codi	MGI Symbol	5	Ngfi-A bindi	218	231	264	263	311	255	331	366	279	724	892	909	3.8733408	4.0126141	4.6932028	4.323299	5.0908035	4.4708672	4.4984055	4.4511744	4.0608874	11.041692	12.05247	13.048953	0.1431137	2.6728091	0.2251851	0.705095	0.0500495	2.6728091	0.6819854	0.7504762	1.5228774	2.6728091	8.48E-45	1.63E-43
4986	ENSMUSG00	320910	Itgb8	1	protein codi	MGI Symbol	3	integrin beta	262	233	258	258	263	260	225	210	179	534	692	718	4.655116	4.0473553	4.5865391	4.241107	4.3050847	4.5585313	3.0578285	2.5539525	2.6053722	8.1440107	9.3501227	10.307094	-0.020303	2.390543	0.8712484	0.9840733	-0.693662	2.390543	6.40E-09	8.10E-08	1.0657767	2.390543	1.32E-22	1.02E-21
5001	ENSMUSG00	14561	Gdf11	1	protein codi	MGI Symbol	1	growth diffe	588	569	560	652	555	644	1097	1247	1095	1325	1560	1677	10.44736	9.8838849	9.9552787	10.717836	9.0848745	11.291131	14.908613	15.165613	15.937892	20.207517	21.078311	24.073811	0.0373324	3.8567382	0.7175006	0.9476234	0.6030451	3.8567382	1.22E-09	1.67E-08	1.1097509	3.8567382	1.89E-29	2.09E-28
5022	ENSMUSG00	71885	Faap100	1	protein codi	MGI Symbol	8	Fanconi ane	451	420	415	477	452	445	719	891	601	1062	1376	1054	8.0131958	7.295662	7.3775726	7.8411165	7.3988527	7.8021016	9.7714609	10.836056	8.7476464	16.196516	18.592151	15.130469	0.0223872	3.3926382	0.8400747	0.978988	0.3735681	3.3926382	0.0002982	0.0014143	1.1382022	3.3926382	1.68E-29	1.86E-28
50222	ENSMUSG00	NA	Gm4798	1	processed p	MGI Symbol	1	predicted ps	142.26	160.83	147.81	186.47	200.39	186.68	586.75	701.37	508.98	1121.52	1332.26	1205.94	2.5276214	2.7937174	2.6276603	3.0652683	3.2802126	3.2730254	7.9741372	8.5298366	7.4082813	17.104253	18.001148	17.311611	0.2742351	2.994915	0.0347321	0.290709	1.5892589	2.994915	1.19E-42	9.51E-41	2.7201453	2.994915	1.45E-126	1.23E-124
5035	ENSMUSG00	13198	Ddit3	1	protein codi	MGI Symbol	3	DNA-damag	189	174	196	232	205	195	403	342	325	786	1423	1476	3.3580798	3.0224885	3.4843475	3.8137087	3.3556744	3.4188984	5.4769106	4.1592941	4.7304244	11.987252	19.227203	21.188399	0.1028483	2.8856482	0.4172719	0.8400992	0.5403858	2.8856482	4.00E-06	3.16E-05	2.4105102	2.8856482	3.19E-105	1.92E-103
5053	ENSMUSG00	170822	Usp33	1	protein codi	MGI Symbol	11	ubiquitin spe	263.46	250.41	226.96	245.87	290.5	270.51	451.66	428.57	364.82	537.02	635.73	652.2	4.6810567	4.3497779	4.0347322	4.0417093	4.7552361	4.7428011	6.138217	5.2121307	5.3100106	8.1900686	8.5898172	9.3625162	0.0508278	2.5489308	0.6753672	0.9362084	0.3499955	2.5489308	0.0018209	0.0064093	1.0005961	2.5489308	5.45E-20	3.55E-19
5057	ENSMUSG00	18115	Nnt	1	protein codi	MGI Symbol	5	nicotinamide	194.62	284.71	353.07	290.73	306.25	579.48	438.68	425.75	760.98	1298.12	1248.36	578.71	3.4579339	4.9455903	6.2766254	4.7791359	5.0130501	10.159914	5.9618143	5.1778347	11.07618	19.797571	16.867513	8.3075464	0.4373295	3.0968743	7.64E-05	0.0021345	0.5897203	3.0968743	4.25E-08	4.81E-07	1.6139232	3.0968743	5.42E-54	1.31E-52
511	ENSMUSG00	57278	Bcam	1	protein codi	MGI Symbol	5	basal cell ad	208	212	238	282	256	225	674	641	611	1039	987	1046	3.6956646	3.6825722	4.2309934	4.6356286	4.1905007	3.9448828	9.1598952	7.7956361	8.8931979	15.845744	13.336085	15.015627	0.138679	2.9973345	0.2473549	0.7231049	1.1533879	2.9973345	1.44E-25	6.04E-24	1.9276757	2.9973345	2.69E-70	8.92E-69
51143	ENSMUSG00	NA	Gm7049	1	processed p	MGI Symbol	1	predicted ge	119.93	85.24	103.82	115.65	139.37	145.94	264.79	387.81	289.4	442.87	556.04	444.7	2.1308705	1.480672	1.8456376	1.9011009	2.2813675	2.5587387	3.5985885	4.7164206	4.212261	6.754191	7.5130668	6.3837948	0.3033945	1.9571165	0.0319854	0.2764887	1.2015921	1.9571165	8.79E-21	2.98E-19	1.9209462	1.9571165	4.20E-54	1.02E-52
5145	ENSMUSG00	15452	Hprt	1	protein codi	MGI Symbol	1	hypoxanthin	372	371	397	396	458	405	619	732	648	956	1226	1070	6.609554	6.4445014	7.0575815	6.5096062	7.4970676	7.1007891	8.412426	8.9023488	9.4317386	14.579914	16.565391	15.360154	0.069791	3.2663973	0.522784	0.8815586	0.4109332	3.2663973	8.33E-05	0.0004671	1.2097776	3.2663973	1.80E-32	2.24E-31
515	ENSMUSG00	11816	Apoe	1	protein codi	MGI Symbol	11	apolipoprote	338	259	406	269	250	214	556	557	365	1806	2698	2112	6.005455	4.4989915	7.217577	4.4219294	4.0922858	3.7520219	7.556234	6.7740551	5.3126305	27.543227	36.454669	30.31836	-0.528655	3.6008462	3.55E-06	0.0001376	0.1514548	3.6008462	0.1650593	0.2418176	2.4130743	3.6008462	1.12E-124	9.33E-123
51753	ENSMUSG00	NA	Tmed10-ps	1	processed p	MGI Symbol	1	transmembr	178.57	181.53	184.43	220.24	209.07	206.26	329.08	408.7	304.63	455.99	601.25	541.72	3.1727636	3.1532893	3.2786644	3.6203931	3.4222968	3.6163179	4.472312	4.9704781	4.433936	6.9542836	8.1239324	7.7765444	0.1499635	2.2714337	0.2317743	0.7119176	0.5324424	2.2714337	6.45E-06	4.82E-05	1.251346	2.2714337	6.11E-28	6.31E-27
5176	ENSMUSG00	110639	Prps2	1	protein codi	MGI Symbol	2	phosphoribc	178	218	223	211	217	218	584	665	520	962	1080	907	3.162636	3.786796	3.9643342	3.4685023	3.5521041	3.8221531	7.9367638	8.0875164	7.5686791	14.67142	14.592677	13.020243	-0.010414	2.8923813	0.9398307	0.9970287	1.1122371	2.8923813	2.39E-23	9.10E-22	1.9531697	2.8923813	6.86E-71	2.33E-69
5177	ENSMUSG00	20970	Sdc3	1	protein codi	MGI Symbol	5	syndecan 3 [	1336	1363	1283	1383.55	1406	1375	3544	3891	3283	5987.47	7289.06	6088.52	23.737538	23.67616	22.808255	22.743347	23.015015	24.107617	48.164197	47.321092	47.784564	91.314643	98.487869	87.402434	-0.007471	5.5503447	0.9409067	0.9975733	1.0286435	5.5503447	7.32E-28	3.38E-26	1.9809109	5.5503447	3.91E-96	1.96E-94
5182	ENSMUSG00	18415	Hspa4l	1	protein codi	MGI Symbol	7	heat shock p	1653	1569	1847	1845	2144	1848	2764	3306	2933	4113	4877	4485	29.369873	27.254509	32.834642	30.328847	35.095443	32.400638	37.563724	40.20651	42.690261	62.727183	65.896746	64.383449	0.1290839	5.3860336	0.169716	0.6268354	0.4292114	5.3860336	4.15E-06	3.26E-05	1.1094481	5.3860336	1.51E-32	1.89E-31
5201	ENSMUSG00	108116	Slco3a1	1	protein codi	MGI Symbol	8	solute carrie	173	171	160	199	171	194	399	366	333	749	792	735	3.073798	2.9703767	2.8443653	3.2712415	2.7991235	3.4013656	5.4225492	4.4511744	4.8468656	11.422966	10.701296	10.551134	0.0903397	2.4792587	0.4784372	0.8649415	0.7259944	2.4792587	8.48E-10	1.19E-08	1.8771051	2.4792587	1.02E-61	2.94E-60
5202	ENSMUSG00	72157	Pgm1	1	protein codi	MGI Symbol	2	phosphogluc	2347.49	2275.85	2234.51	2497.33	2406.89	2607.82	3979.47	4197.53	3515.57	4948.39	6327.29	5667.84	41.709306	39.532934	39.723517	41.052108	39.398727	45.722419	54.082386	51.049011	51.169656	75.46768	85.492684	81.363453	0.0606346	5.7521932	0.5141778	0.8778227	0.3697101	5.7521932	6.25E-05	0.0003634	1.0024247	5.7521932	2.56E-27	2.56E-26
5209	ENSMUSG00	16412	Itgb1	1	protein codi	MGI Symbol	4	integrin beta	8564	7956	8566	8814	9777	8959	17295	17476	14920	19501	22063	23355	152.16188	138.20068	152.28021	144.88805	160.04111	157.07647	235.04508	212.5375	217.16287	297.4089	298.10947	335.26766	0.0617706	7.7035437	0.4951228	0.8711141	0.5866524	7.7035437	9.30E-11	1.43E-09	1.0723117	7.7035437	6.05E-32	7.39E-31
5217	ENSMUSG00	12304	Pdia4	1	protein codi	MGI Symbol	2	protein disul	2029	1836	1963	2143	2134	2178	3877	4044	3406	4878	6163	6052	36.050497	31.892465	34.896807	35.22749	34.931752	38.186466	52.689783	49.181829	49.574848	74.394165	83.272841	86.878179	0.0751627	5.6639409	0.421705	0.8435875	0.558415	5.6639409	1.74E-09	2.35E-08	1.249812	5.6639409	5.14E-41	8.74E-40
5223	ENSMUSG00	18590	Pdgfa	1	protein codi	MGI Symbol	7	platelet deri	509.17	410.5	432.83	511.75	484.77	521.55	962.13	1019.94	875.95	1290.04	1642.28	1457.32	9.0467382	7.130641	7.6945415	8.4123509	7.9352696	9.1442384	13.075682	12.404183	12.749585	19.674344	22.190057	20.920242	0.0943689	3.6590809	0.3710716	0.8088986	0.6793352	3.6590809	2.06E-11	3.48E-10	1.395556	3.6590809	2.91E-44	5.49E-43
5268	ENSMUSG00	14048	Eya1	1	protein codi	MGI Symbol	9	EYA transcri	756	637	840	755	764	641	1385	1544	1227	1909	2053	2274	13.432319	11.065087	14.932918	12.410992	12.506025	11.238533	18.822633	18.777632	17.859172	29.114076	27.739598	32.643916	-0.124314	4.2075247	0.2130081	0.6901695	0.492617	4.2075247	4.24E-07	4.09E-06	1.1826538	4.2075247	2.16E-34	2.91E-33
5271	ENSMUSG00	72265	Tram1	1	protein codi	MGI Symbol	3	translocatin	739	767	781	721	841	717	1328	1331	1149	1786	2089	1975	13.13027	13.323268	13.884058	11.852086	13.766449	12.571027	18.047983	16.187194	16.72387	27.238208	28.226021	28.351686	-0.078811	4.1586169	0.4304275	0.8463211	0.3369616	4.1586169	0.0005474	0.0023899	1.0550938	4.1586169	9.39E-28	9.57E-27
5274	ENSMUSG00	240726	Slco5a1	1	protein codi	MGI Symbol	8	solute carrie	30	26	29	29	14	23	260	286	225	741	949	876	0.5330285	0.4516362	0.5155412	0.4767136	0.229168	0.4032547	3.5334907	3.4782401	3.2749092	11.300959	12.822639	12.575229	-0.43579	2.1011915	0.0939393	0.4829133	2.774685	2.1011915	4.79E-63	6.50E-61	4.6085654	2.1011915	9.61E-211	2.23E-208
52833	ENSMUSG00	18950	Pnp	1	protein codi	MGI Symbol	5	purine-nucle	273.02	243.24	219.45	431.87	369.95	389.56	475.35	529.4	417.75	683.65	849.96	764.08	4.8509151	4.2252305	3.9012248	7.0992515	6.0557645	6.8300825	6.4601724	6.4383927	6.0804148	10.426316	11.484437	10.968585	0.6224749	2.8360538	2.87E-08	1.58E-06	0.5488975	2.8360538	8.03E-07	7.37E-06	1.3413969	2.8360538	3.54E-35	4.95E-34
5285	ENSMUSG00	93691	Klf7	1	protein codi	MGI Symbol	3	Kruppel-like	743	703	921	797	1179	929	1016	1334	1131	1882	2041	2201	13.20134	12.211548	16.372878	13.101404	19.29922	16.287983	13.807795	16.223679	16.461877	28.7023	27.577457	31.59598	0.2208297	4.2329648	0.0247891	0.2318986	0.1542803	4.2329648	0.1149814	0.1813889	1.0725114	4.2329648	9.74E-29	1.04E-27
5298	ENSMUSG00	67876	Coq10b	1	protein codi	MGI Symbol	4	coenzyme Q	122.98	124.96	139.34	166.04	179.94	171	251.96	293.98	262.08	308.48	365.99	378.34	2.1850617	2.1706331	2.4770867	2.7294318	2.9454636	2.9981109	3.4242243	3.5752903	3.8146143	4.704615	4.945161	5.4311781	0.3435202	1.8143153	0.0097714	0.1232778	0.6613066	1.8143153	1.63E-07	1.69E-06	1.1422713	1.8143153	1.42E-20	9.59E-20
5303	ENSMUSG00	53945	Slc40a1	1	protein codi	MGI Symbol	8	solute carrie	151	116	137	161	156	121	314	380	273	425	625	531	2.6829104	2.0149924	2.4354878	2.6465823	2.5535863	2.1214703	4.2673696	4.6214379	3.9735565	6.4816564	8.4448362	7.6226558	0.0407238	2.0889051	0.79778	0.9701843	0.8532134	2.0889051	4.93E-12	8.84E-11	1.662415	2.0889051	1.47E-44	2.81E-43
5307	ENSMUSG00	11363	Acadl	1	protein codi	MGI Symbol	2	acyl-Coenzyı	741	690	731	1225	1248	1079	1428	1556	1441	2002	2112	2056	13.165805	11.98573	12.995194	20.137039	20.428691	18.917905	19.407018	18.923572	20.973974	30.532415	28.536791	29.514464	0.6411584	4.3601396	5.94E-11	4.51E-09	0.636177	4.3601396	6.26E-11	9.78E-10	1.2153224	4.3601396	4.17E-36	6.00E-35
5322	ENSMUSG00	70827	Trak2	1	protein codi	MGI Symbol	6	trafficking p	848	795	785	881	921	835	1319	1576	1234	2055	2048	2084	15.06694	13.809646	13.955167	14.48223	15.075981	14.639898	17.92567	19.166806	17.961058	31.340715	27.672039	29.916412	0.0453987	4.2729166	0.6489127	0.9276372	0.3625126	4.2729166	0.000188	0.0009434	1.0536557	4.2729166	7.16E-28	7.35E-27
533	ENSMUSG00	16890	Lipe	1	protein codi	MGI Symbol	8	lipase, horm	112.75	98.82	115.86	126.01	146.93	107.21	249.41	303.27	235.48	340.37	424.27	339.05	2.003299	1.716565	2.059676	2.0714027	2.4051182	1.8796928	3.3895689	3.6882723	3.4274472	5.190968	5.7326251	4.867159	0.139974	1.7165846	0.3379978	0.7875353	0.8635882	1.7165846	2.62E-11	4.37E-10	1.4508038	1.7165846	6.67E-31	7.89E-30
5331	ENSMUSG00	12832	Col5a2	1	protein codi	MGI Symbol	4	collagen, typ	68	41	65	49	56	46	238	257	269	796	1213	1113	1.208198	0.7121956	1.1555234	0.8054816	0.916672	0.8065094	3.234503	3.1255514	3.9153359	12.139761	16.389738	15.977431	-0.278314	2.3743998	0.1339837	0.5634552	1.7369444	2.3743998	2.15E-33	1.22E-31	3.8560666	2.3743998	2.86E-190	5.66E-188
5364	ENSMUSG00	14660	Gls	1	protein codi	MGI Symbol	17	glutaminase	4166	3218	3640.99	4043	5023	4377.98	7221.99	7799	6479	8759	10248.52	11424.99	74.019898	55.898667	64.726911	66.460449	82.222206	76.758303	98.149364	94.848932	94.30283	133.58313	138.47532	164.00898	0.2119436	6.5756737	0.0206382	0.2057325	0.5617694	6.5756737	7.95E-10	1.11E-08	1.1637636	6.5756737	1.13E-36	1.68E-35
538	ENSMUSG00	60611	Foxj2	1	protein codi	MGI Symbol	3	forkhead bo	169	138	150.92	155.98	133	174	235	289.91	243.97	346.99	426.97	407.8	3.0027275	2.3971461	2.6829476	2.5640615	2.177096	3.0507094	3.193732	3.5257923	3.5510205	5.2919293	5.7691068	5.8540848	-0.05524	1.8692297	0.7016734	0.9430758	0.3461392	1.8692297	0.0054246	0.0156731	1.0662221	1.8692297	3.05E-19	1.88E-18
539	ENSMUSG00	109552	Sri	1	protein codi	MGI Symbol	7	sorcin [Sour	1978	2267	2041	2458	2552	2436	3501	3755	2977	4645	5631	5269	35.144349	39.379204	36.283435	40.405586	41.774053	42.709931	47.579812	45.667104	43.330688	70.840692	76.084596	75.637991	0.1724871	5.6336912	0.0637175	0.3977113	0.3016913	5.6336912	0.001126	0.004336	1.0061401	5.6336912	2.11E-27	2.11E-26
5414	ENSMUSG00	111175	Pecr	1	protein codi	MGI Symbol	4	peroxisomal	247	301	261	330	336	299	569	591	564	808	981	1007	4.3886017	5.2285577	4.639871	5.4246718	5.5000321	5.242311	7.7329085	7.1875521	8.2091058	12.322773	13.255015	14.455771	0.181186	2.9805626	0.1116055	0.5241324	0.6960903	2.9805626	1.21E-10	1.84E-09	1.4890494	2.9805626	1.40E-45	2.72E-44
5416	ENSMUSG00	14268	Fn1	1	protein codi	MGI Symbol	17	fibronectin 1	146	122.93	180	180	255	229	184	219	195.97	3570.96	6814.94	5764	2.5940723	2.1353708	3.199911	2.9589119	4.1741315	4.0150141	2.5006242	2.6634076	2.8523732	54.460555	92.081683	82.743857	0.4913412	4.4307676	8.95E-05	0.0024496	0.0157721	4.4307676	0.9300128	0.9518057	4.8543614	4.4307676	0	0
5423	ENSMUSG00	22145	Tuba4a	1	protein codi	MGI Symbol	8	tubulin, alph	370.97	323.15	345.84	332.83	347.25	331.72	543.15	602.13	457.67	1068.6	1371.03	1275.08	6.5912533	5.6133171	6.1480957	5.4711925	5.684185	5.8159846	7.381598	7.3229116	6.6614565	16.297172	18.524998	18.304135	-0.112841	3.2084685	0.3105708	0.7696817	0.2204576	3.2084685	0.0397829	0.0775256	1.5338827	3.2084685	5.88E-51	1.32E-49
5431	ENSMUSG00	71728	Stk11ip	1	protein codi	MGI Symbol	9	serine/threo	223	205	191	322	323	265	360	450	336	543	643	580	3.9621789	3.5609779	3.3954611	5.2931646	5.2872333	4.6461953	4.8925256	5.4727554	4.8905311	8.2812693	8.6880475	8.3260647	0.4806469	2.4923966	4.14E-05	0.0012807	0.4841073	2.4923966	2.74E-05	0.0001769	1.2120554	2.4923966	1.96E-27	1.97E-26
5444	ENSMUSG00	19231	Ptma	1	protein codi	MGI Symbol	5	prothymosir	6552.52	6261.72	7052.23	6180.69	6410.75	6032.48	10528.56	9745.96	7925.48	14191.36	18095.12	17279.56	116.42267	108.76998	125.36949	101.60065	104.93848	105.76634	143.0868	118.52723	115.35657	216.43181	244.49652	248.05299	-0.166703	7.1880313	0.0664931	0.4072755	0.1047983	7.1880313	0.2476114	0.3351544	1.016121	7.1880313	8.13E-29	8.66E-28
5481	ENSMUSG00	16978	Lrrfip1	1	protein codi	MGI Symbol	12	leucine rich	469	457	490	724	729	786	951	1173	928	1562	1906	1834	8.3330129	7.938375	8.7108688	11.901401	11.933105	13.780791	12.924422	14.265649	13.507181	23.821994	25.753373	26.327591	0.5898299	3.9095804	7.50E-09	4.48E-07	0.7044514	3.9095804	2.84E-12	5.19E-11	1.6033629	3.9095804	1.60E-58	4.27E-57
5496	ENSMUSG00	18484	Pam	1	protein codi	MGI Symbol	15	peptidylglyci	1153	1034	1175	1202	1184	1189	1704	1822	1419	3107	3724	3416	20.486064	17.961225	20.888308	19.758956	19.381066	20.846514	23.157955	22.158579	20.653761	47.384721	50.317712	49.03765	0.0156977	4.794737	0.8734522	0.9848106	0.1532762	4.794737	0.1087315	0.1735175	1.3064268	4.794737	3.49E-43	6.36E-42
5542	ENSMUSG00	21952	Tnni1	1	protein codi	MGI Symbol	9	troponin I, sl	176	146	145	144	177	133	365	381	380	815	967	966	3.1271008	2.5361111	2.5777061	2.3671295	2.8973383	2.3318641	4.9604774	4.6335996	5.5309578	12.429529	13.065851	13.867204	-0.115515	2.5792394	0.3879089	0.8223108	0.8736677	2.5792394	2.03E-13	4.11E-12	2.2557876	2.5792394	1.76E-87	7.55E-86
5546	ENSMUSG00	14270	Srgap2	1	protein codi	MGI Symbol	12	SLIT-ROBO F	690	585	624	738	703	657	993	1234	1123	1506	1712	1479	12.259657	10.161815	11.093025	12.131539	11.507508	11.519058	13.495216	15.007512	16.345436	22.96794	23.132065	21.231465	0.0693675	3.9212942	0.4958369	0.8711141	0.42001	3.9212942	2.12E-05	0.0001407	1.0065954	3.9212942	8.41E-25	7.22E-24
5564	ENSMUSG00	72017	Cyb5r1	1	protein codi	MGI Symbol	10	cytochrome	254	225	271	286	283	261	512	573	464	705	900	763	4.512975	3.9083903	4.8176438	4.7013822	4.6324675	4.5760641	6.9582587	6.9686419	6.7535906	10.751924	12.160564	10.953082	0.0719571	2.7864835	0.5470657	0.891083	0.6440849	2.7864835	5.57E-09	7.09E-08	1.3557995	2.7864835	3.62E-36	5.24E-35
5568	ENSMUSG00	381290	Atp2b4	1	protein codi	MGI Symbol	8	ATPase, Ca+	34	25	32	34	29	16	1057	1128	892	1934	2563	2379	0.604099	0.4342656	0.5688731	0.5589056	0.4747052	0.280525	14.364999	13.718374	12.983196	29.495349	34.630584	34.151221	-0.277771	3.5950808	0.2861884	0.7516196	4.672435	3.5950808	3.85E-225	4.57E-222	5.9310609	3.5950808	0	0
5574	ENSMUSG00	14645	Glul	1	protein codi	MGI Symbol	5	glutamate-a	374.98	360.97	347.99	322	334.99	320.99	676.99	679.96	642.99	1683.79	2000.98	1864.94	6.6625015	6.270274	6.1863168	5.2931646	5.4834993	5.6278575	9.2005303	8.2694551	9.3588172	25.679408	27.036717	26.771743	-0.221099	3.5776426	0.0455732	0.3403169	0.4875654	3.5776426	3.22E-06	2.59E-05	2.0555634	3.5776426	6.97E-92	3.24E-90
5577	ENSMUSG00	16782	Lamc2	1	protein codi	MGI Symbol	6	laminin, gam	216	173	263	261	272	260	602	584	477	1405	1881	1969	3.8378055	3.0051179	4.6754255	4.2904222	4.452407	4.5585313	8.1813901	7.1024204	6.9428076	21.427594	25.415579	28.265554	0.2082598	3.3672089	0.0794595	0.4480404	0.9490729	3.3672089	1.75E-17	4.79E-16	2.7059149	3.3672089	3.86E-137	4.12E-135
5592	ENSMUSG00	78825	Desi2	1	protein codi	MGI Symbol	4	desumoylati	363	332	325	375	393.97	397	658	702	630.99	950	1269	1092	6.4496454	5.7670471	5.7776171	6.1643998	6.4489514	6.9605266	8.9424496	8.5374985	9.1841554	14.488408	17.146395	15.67597	0.1208372	3.2307725	0.2711642	0.7416878	0.5666878	3.2307725	7.31E-08	8.04E-07	1.3951378	3.2307725	4.72E-42	8.29E-41
5612	ENSMUSG00	21346	Tagln2	1	protein codi	MGI Symbol	4	transgelin 2	356	174	484	172	198	185	655	574	489	2120	3133	3039	6.3252721	3.0224885	8.6042051	2.8274047	3.2410904	3.2435703	8.9016785	6.9808036	7.1174694	32.332027	42.332275	43.625708	-0.945264	3.8279638	8.31E-16	1.05E-13	0.3587207	3.8279638	0.0007484	0.0030992	2.722007	3.8279638	7.26E-158	9.19E-156
5625	ENSMUSG00	74106	Dcaf6	1	protein codi	MGI Symbol	5	DDB1 and CU	2133	1966	2112	2604	2552	2387	3500	3776	3238	4949	5549	5366	37.89833	34.150646	37.545622	42.805592	41.774053	41.850824	47.566221	45.922499	47.129582	75.476983	74.976634	77.030454	0.2062241	5.6560362	0.0266926	0.2441335	0.359588	5.6560362	0.0001036	0.0005668	1.0535977	5.6560362	7.58E-30	8.52E-29
5671	ENSMUSG00	54139	Irf6	1	protein codi	MGI Symbol	4	interferon re	93	76	75	61	57	70	318	305	266	689	993.97	775	1.6523885	1.3201674	1.3332963	1.0027424	0.9330412	1.2272969	4.321731	3.709312	3.8716705	10.507909	13.430262	11.125345	-0.447488	2.2250719	0.0075734	0.1029845	1.4653115	2.2250719	8.07E-28	3.71E-26	3.0257075	2.2250719	5.96E-129	5.50E-127
5673	ENSMUSG00	18845	Plxna2	1	protein codi	MGI Symbol	5	plexin A2 [Sc	171	144	130	162	151	203	256	279	248	613	656	695	3.0382627	2.5013698	2.3110468	2.6630207	2.4717406	3.5591609	3.4791293	3.3931084	3.6096777	9.3488362	8.8637001	9.9769224	0.1436242	2.2277	0.2749515	0.7446986	0.4159425	2.2277	0.0008501	0.0034423	1.8434142	2.2277	5.33E-57	1.39E-55
5722	ENSMUSG00	20163	Rsu1	1	protein codi	MGI Symbol	10	Ras suppres	555	566	570	564	579	539	881	967	889	1266	1459	1498	9.8610281	9.831773	10.133052	9.2712573	9.4777339	9.450186	11.973097	11.760343	12.93953	19.307711	19.713626	21.504215	-0.080898	3.7018997	0.434511	0.8480621	0.2974993	3.7018997	0.002984	0.0096161	1.020934	3.7018997	4.62E-25	4.05E-24
5725	ENSMUSG00	19212	Pter	1	protein codi	MGI Symbol	7	phosphotrie	121	128	136	144	137	151	254	317	285	479	537	614	2.1498818	2.2234398	2.4177105	2.3671295	2.2425726	2.6474547	3.4519486	3.8552522	4.1482183	7.3052081	7.2558033	8.8141444	0.0941321	2.0575357	0.4962978	0.8711141	0.7530597	2.0575357	2.10E-09	2.82E-08	1.7824703	2.0575357	1.55E-50	3.40E-49
573	ENSMUSG00	18807	Pld3	1	protein codi	MGI Symbol	8	phospholipa	3528	3163	3258	3878	3716	3506	8629	8636	7479	10360	12028	12730	62.684157	54.943283	57.918389	63.748113	60.827736	61.470041	117.27112	105.02826	108.85798	157.99991	162.51918	182.74276	0.083851	6.6409245	0.3618029	0.8032775	0.9156384	6.6409245	1.99E-23	7.63E-22	1.519475	6.6409245	2.50E-60	6.95E-59
5761	ENSMUSG00	56017	Slc2a8	1	protein codi	MGI Symbol	8	solute carrie	473	448	416	638	545	516	1089	1353	1229	1703	1874	1886	8.4040834	7.7820394	7.3953499	10.487699	8.9211831	9.0469313	14.79989	16.454751	17.888282	25.972379	25.320997	27.074066	0.2713375	3.9148694	0.0093036	0.1189558	1.058813	3.9148694	5.92E-26	2.53E-24	1.7323121	3.9148694	1.40E-67	4.40E-66
5763	ENSMUSG00	227737	Fam129b	1	protein codi	MGI Symbol	6	family with s	877	820	811	1066	917	972	1622	1372	1283	2865	3542	3030	15.582201	14.243911	14.417377	17.523334	15.010504	17.041894	22.043546	16.685823	18.67426	43.69399	47.858576	43.49651	0.1640354	4.5823021	0.0943417	0.4835194	0.3750702	4.5823021	0.000105	0.0005726	1.6100186	4.5823021	6.63E-63	1.94E-61
5764	ENSMUSG00	20910	Stxbp1	1	protein codi	MGI Symbol	5	syntaxin bin	402	316	402	445	526	455	675	754	678	944	1078	1105	7.1425825	5.4891171	7.1464679	7.3150877	8.6101693	7.9774297	9.1734855	9.1699058	9.8683931	14.396903	14.565654	15.862589	0.2737975	3.2934512	0.010496	0.1296649	0.5122583	3.2934512	8.93E-07	8.09E-06	1.1807918	3.2934512	7.34E-31	8.63E-30
5773	ENSMUSG00	50935	St6galnac6	1	protein codi	MGI Symbol	13	ST6 (alpha-N	504	528	586	624	604	593	858	898	793	1527.78	1742	1693.85	8.9548796	9.1716893	10.417488	10.257561	9.8869625	10.396958	11.660519	10.921187	11.542236	23.300106	23.537448	24.315698	0.0974993	3.7843509	0.3443136	0.7903445	0.257212	3.7843509	0.0107466	0.0272611	1.31769	3.7843509	1.15E-40	1.93E-39
5778	ENSMUSG00	11636	Ak1	1	protein codi	MGI Symbol	6	adenylate ki	727	768	769	883	965	857	1558	1581	1255	4945	6815	5188	12.917058	13.340639	13.670731	14.515107	15.796223	15.02562	21.173764	19.227614	18.266716	75.415979	92.082494	74.475213	0.1832833	5.0138015	0.0637955	0.3979362	0.555238	5.0138015	1.10E-08	1.35E-07	2.5993575	5.0138015	6.22E-155	7.48E-153
5793	ENSMUSG00	56177	Olfm1	1	protein codi	MGI Symbol	5	olfactomedi	907	971	949	1023	916	1033	1674	1784	1398	2044	2835	2282	16.11523	16.866876	16.870642	16.816483	14.994135	18.111395	22.750244	21.696435	20.348103	31.172955	38.305777	32.758758	0.0015386	4.4803847	0.9895838	1	0.3783571	4.4803847	8.12E-05	0.0004567	1.0364787	4.4803847	1.96E-27	1.97E-26
5796	ENSMUSG00	11477	Acvr1	1	protein codi	MGI Symbol	6	activin A rec	178	130	147	142	152	156	259	262	250	363	526	443	3.162636	2.2581811	2.6132607	2.3342527	2.4881098	2.7351188	3.5199004	3.1863598	3.638788	5.5360971	7.1071742	6.3593908	-0.088684	1.9323918	0.5122397	0.8777557	0.3630527	1.9323918	0.0033313	0.0105186	1.2443161	1.9323918	5.92E-26	5.50E-25
5797	ENSMUSG00	12831	Col5a1	1	protein codi	MGI Symbol	3	collagen, typ	512	393	518	538	608	538	997	1021	1045	3354	5127.99	5001	9.0970205	6.8266551	9.2086328	8.8438589	9.9524391	9.4326531	13.549578	12.417074	15.210134	51.151707	69.288057	71.790775	0.1681441	4.5883717	0.1068664	0.5150841	0.7114544	4.5883717	1.61E-12	3.02E-11	2.9357807	4.5883717	3.50E-188	6.79E-186
5807	ENSMUSG00	74270	Usp20	1	protein codi	MGI Symbol	12	ubiquitin spe	296	235	237	280	283	301	461	517	383	611	806	638	5.259215	4.0820966	4.2132162	4.6027518	4.6324675	5.2773766	6.2651509	6.2875879	5.5746233	9.3183343	10.890461	9.1586712	0.0978087	2.671973	0.3959863	0.8267847	0.4209672	2.671973	0.0001516	0.0007898	1.1164577	2.671973	7.31E-25	6.30E-24
5812	ENSMUSG00	14828	Hspa5	1	protein codi	MGI Symbol	6	heat shock p	5087	5077	5149	5753	5804	5222	11797	13551	12617	16876	20390	21111	90.383874	88.190657	91.535232	94.570112	95.006507	91.556347	160.32535	164.80291	183.64235	257.37514	275.50434	303.0544	0.0577354	7.3049694	0.5266476	0.8829715	0.9134045	7.3049694	1.43E-23	5.59E-22	1.6298464	7.3049694	2.30E-69	7.49E-68
582	ENSMUSG00	14132	Fcgrt	1	protein codi	MGI Symbol	6	Fc receptor,	169	178	195	180	185	185	418	500	380	853	1373	1055	3.0027275	3.091971	3.4665703	2.9589119	3.0282915	3.2435703	5.6807659	6.0808394	5.5309578	13.009066	18.551616	15.144825	-0.051376	2.8129491	0.6882141	0.9398213	0.8558784	2.8129491	1.43E-13	2.92E-12	2.2891986	2.8129491	5.38E-94	2.65E-92
5875	ENSMUSG00	56878	Rbms1	1	protein codi	MGI Symbol	9	RNA binding	226	212	236	294	269	320	545	600	509	902	1035	960	4.0154817	3.6825722	4.1954389	4.8328894	4.4032995	5.6105	7.4067402	7.2970072	7.4085724	13.756363	13.984649	13.781073	0.3185362	2.9325047	0.006291	0.0892006	0.8945342	2.9325047	8.08E-16	1.99E-14	1.8036082	2.9325047	7.49E-62	2.16E-60
5891	ENSMUSG00	76747	Dapl1	1	protein codi	MGI Symbol	2	death associ	995	1280	972	842	870	948	1991	2026	1345	2306	3310	3229	17.67878	22.234398	17.279519	13.841132	14.241155	16.621106	27.058385	24.639561	19.57668	35.168705	44.723853	46.353212	-0.356256	4.6470096	0.0002572	0.0062817	0.3179841	4.6470096	0.0008521	0.0034489	1.1423712	4.6470096	2.20E-33	2.84E-32
5910	ENSMUSG00	53416	Stk39	1	protein codi	MGI Symbol	8	serine/threo	3824	3947	4223	4393	4638	4229	7929	8678	7324	11533	12574	11271	67.943372	68.561852	75.073468	72.213888	75.920086	74.146264	107.75788	105.53905	106.60193	175.88928	169.89659	161.79841	0.0711975	6.7172265	0.4368423	0.8498877	0.5964106	6.7172265	6.46E-11	1.00E-09	1.2624272	6.7172265	8.49E-43	1.53E-41
5928	ENSMUSG00	16410	Itgav	1	protein codi	MGI Symbol	9	integrin alph	2991	2705	3514.34	2951.65	3580.89	2980.22	4748.96	6003	4545.11	7821	8377.1	8465	53.142946	46.987537	62.475418	48.520402	58.616101	52.251639	64.540023	73.006557	66.154767	119.27773	113.18918	121.51748	-0.028718	6.1976658	0.7558131	0.9586225	0.3253016	6.1976658	0.0003899	0.001784	1.1223262	6.1976658	4.28E-34	5.67E-33
5970	ENSMUSG00	56348	Hsd17b12	1	protein codi	MGI Symbol	5	hydroxysterc	770	823	939	972	947	857	1524	1664	1346	3141	3777	3851	13.681066	14.296023	16.692869	15.978124	15.501579	15.02562	20.711692	20.237033	19.591235	47.903254	51.033834	55.282199	0.058313	4.6785674	0.55618	0.8953731	0.4387315	4.6785674	5.58E-06	4.25E-05	1.7876337	4.6785674	6.08E-77	2.28E-75
5974	ENSMUSG00	214968	Sema6d	1	protein codi	MGI Symbol	11	sema domai	392	363	294	388	464	440	446	573	491	902	1076	1131	6.9649063	6.3055364	5.2265213	6.378099	7.5952825	7.7144375	6.0612956	6.9686419	7.1465797	13.756363	14.53863	16.235826	0.2288582	3.1390489	0.0343435	0.2887286	0.1252002	3.1390489	0.2446926	0.3320353	1.2673788	3.1390489	5.34E-35	7.41E-34
5978	ENSMUSG00	14118	Fbn1	1	protein codi	MGI Symbol	3	fibrillin 1 [Sc	360	221	425	269.07	406	314	442	752	731	1653	2008.46	2450.94	6.3963426	3.8389078	7.5553454	4.4230801	6.6458721	5.5053031	6.0069342	9.1455824	10.639816	25.209831	27.137785	35.18394	-0.100629	3.6361427	0.3659277	0.8074727	0.5360043	3.6361427	3.85E-07	3.75E-06	2.2997934	3.6361427	1.92E-113	1.35E-111
5980	ENSMUSG00	69976	Galk2	1	protein codi	MGI Symbol	9	galactokinas	151	128	135	127	147	155	230	248	210	328	418	363	2.6829104	2.2234398	2.3999333	2.0876767	2.4062641	2.7175859	3.1257803	3.0160963	3.0565819	5.0023137	5.6479065	5.2109681	-0.020502	1.749518	0.8983239	0.9892101	0.3322184	1.749518	0.0090931	0.0237593	1.1193165	1.749518	2.90E-20	1.92E-19
5984	ENSMUSG00	12521	Cd82	1	protein codi	MGI Symbol	11	CD82 antige	326	279	294	440	415	375	616	638	530	971	1144	1083	5.7922435	4.846404	5.2265213	7.2328957	6.7931944	6.5748047	8.3716549	7.759151	7.7142306	14.808679	15.457428	15.546773	0.377427	3.1590294	0.0006022	0.0128851	0.587928	3.1590294	4.06E-08	4.63E-07	1.5300431	3.1590294	2.97E-49	6.33E-48
5985	ENSMUSG00	241556	Tspan18	1	protein codi	MGI Symbol	5	tetraspanin	258	279	202	289	284	267	695	608	585	1109	1240	1196	4.5840455	4.846404	3.5910112	4.7506974	4.6488367	4.6812609	9.4452925	7.3943007	8.514764	16.913311	16.754555	17.16892	0.1122671	3.1249424	0.3329194	0.7833205	0.959034	3.1249424	1.37E-18	4.13E-17	1.9639185	3.1249424	5.37E-75	1.93E-73
5995	ENSMUSG00	26427	Creb3l1	1	protein codi	MGI Symbol	1	cAMP respor	170	189	207	163	190	202	399	466	432	537	762	626	3.0204951	3.2830479	3.6798977	2.6794591	3.1101372	3.5416281	5.4225492	5.6673423	6.2878257	8.1897635	10.295944	8.9864078	-0.099398	2.4448216	0.4438902	0.850865	0.7982149	2.4448216	4.02E-12	7.27E-11	1.4613211	2.4448216	1.57E-38	2.50E-37
6006	ENSMUSG00	14827	Pdia3	1	protein codi	MGI Symbol	4	protein disul	4537	4279	4857	4834	5107	4742	8508	8946	7174	10380	13007	12855	80.611684	74.328899	86.344265	79.463223	83.597214	83.140597	115.62669	108.79838	104.41866	158.30493	175.74718	184.53718	0.0291167	6.7988292	0.7504766	0.958233	0.4467125	6.7988292	9.32E-07	8.40E-06	1.1039092	6.7988292	2.15E-33	2.79E-32
6106	ENSMUSG00	56264	Cpxm1	1	protein codi	MGI Symbol	2	carboxypept	2990	2912	3155	2871	2897	2834	4714	5173	3793	6292	8304	7828	53.125178	50.583256	56.087329	47.194645	47.421408	49.687991	64.064905	62.912364	55.207692	95.959017	112.20147	112.37316	-0.147141	6.0730154	0.1109065	0.5234037	0.189382	6.0730154	0.0391767	0.0765418	1.0043517	6.0730154	1.06E-27	1.08E-26
6132	ENSMUSG00	13010	Cst3	1	protein codi	MGI Symbol	3	cystatin C [Sc	4408	4556	4457	4930	4785	4509	7676	7950	6336	9598	13753	11522	78.319661	79.140562	79.233352	81.041309	78.32635	79.055452	104.31952	96.685346	92.221444	146.37868	185.82693	165.40158	0.0105069	6.7223077	0.9092629	0.9905825	0.3090184	6.7223077	0.0006904	0.0028944	1.0720688	6.7223077	1.31E-31	1.59E-30
6160	ENSMUSG00	13555	E2f1	1	protein codi	MGI Symbol	2	E2F transcrip	450	441	482	523	512	479	780	762	758	1102	1292	1228	7.9954282	7.6604451	8.5686506	8.5972829	8.3810013	8.3982172	10.600472	9.2671992	11.032805	16.806554	17.457165	17.628289	0.0672168	3.4736189	0.5269016	0.8829715	0.34984	3.4736189	0.0006186	0.0026484	1.0991538	3.4736189	6.63E-28	6.82E-27
617	ENSMUSG00	12870	Cp	1	protein codi	MGI Symbol	16	ceruloplasm	7768.36	7256	7789	8125	8657	8211	12317	13485	10768	16103	22117	22091.59	138.02525	126.04125	138.46726	133.56199	141.70767	143.96192	167.39233	164.00024	156.72988	245.58615	298.83911	317.13105	0.0586328	7.5001907	0.5177298	0.8792337	0.2781681	7.5001907	0.0021118	0.0072118	1.0978858	7.5001907	2.45E-33	3.16E-32
6199	ENSMUSG00	12349	Car2	1	protein codi	MGI Symbol	3	carbonic anh	251	215	206	295	288	284	982	932	709	14206	22875	22288	4.4596722	3.7346841	3.6621204	4.8493278	4.7143132	4.9793188	13.345723	11.334685	10.319603	216.65509	309.08101	319.95057	0.2944158	6.244611	0.0119037	0.141711	1.5618799	6.244611	1.27E-46	1.15E-44	6.1560008	6.244611	0	0
6219	ENSMUSG00	16396	Itch	1	protein codi	MGI Symbol	7	itchy, E3 ubi	923	864	781	1027	961	1070	1497	1664	1486	1870	2287	2228	16.399512	15.008219	13.884058	16.882236	15.730747	18.760109	20.344752	20.237033	21.628956	28.519288	30.901345	31.983573	0.1812899	4.3879803	0.0635322	0.3970765	0.4575984	4.3879803	2.08E-06	1.75E-05	1.0130973	4.3879803	4.96E-26	4.66E-25
6222	ENSMUSG00	66734	Map1lc3a	1	protein codi	MGI Symbol	3	microtubule	376	342	328	413	399	359	810	931	718	1419	1651	1485	6.6806245	5.9407533	5.8309489	6.7890589	6.5312881	6.2942797	11.008183	11.322523	10.450599	21.641107	22.307879	21.317597	0.0885717	3.5186499	0.4228419	0.8444633	0.8295826	3.5186499	1.42E-15	3.41E-14	1.8224175	3.5186499	6.32E-72	2.17E-70
6254	ENSMUSG00	22401	Zmat3	1	protein codi	MGI Symbol	4	zinc finger m	307	251	264	433	457	446	561	640	502	765	944	1050	5.4546588	4.3600266	4.6932028	7.1178269	7.4806984	7.8196344	7.6241857	7.7834744	7.3066864	11.666982	12.755081	15.073048	0.6276105	3.0602965	1.21E-08	6.98E-07	0.6475914	3.0602965	2.25E-09	2.99E-08	1.4452691	3.0602965	4.61E-43	8.36E-42
6258	ENSMUSG00	14696	Gnb4	1	protein codi	MGI Symbol	8	guanine nuc	184	172	182	142	146	128	426	396	427	585	657	671	3.2692418	2.9877473	3.2354656	2.3342527	2.3898949	2.2442	5.7894886	4.8160248	6.2150499	8.9218094	8.8772118	9.6323955	-0.444697	2.3672245	0.0006894	0.0145216	0.8220554	2.3672245	1.45E-12	2.74E-11	1.5306622	2.3672245	1.13E-41	1.95E-40
6281	ENSMUSG00	11747	Anxa5	1	protein codi	MGI Symbol	3	annexin A5 [	1177	1160	1114	585	574	598	2230	2418	1977	3102	4508	4337	20.912487	20.149924	19.803894	9.6164636	9.3958882	10.484622	30.306478	29.406939	28.775536	47.308466	60.910915	62.258867	-1.045397	4.8703209	5.21E-26	1.21E-23	0.5398701	4.8703209	1.20E-08	1.47E-07	1.4860855	4.8703209	2.65E-55	6.62E-54
6290	ENSMUSG00	26570	Slc7a11	1	protein codi	MGI Symbol	5	solute carrie	1874.11	1626.47	2127.7	1946.46	2161.01	2210.45	2942.66	4138.33	3417	4733.2	5466.83	5828.22	33.298471	28.252798	37.824726	31.996687	35.373882	38.755405	39.991776	50.32904	49.734955	72.185826	73.866374	83.665753	0.0947132	5.5860366	0.3115971	0.7696817	0.4952291	5.5860366	9.72E-08	1.05E-06	1.2090054	5.5860366	1.83E-38	2.90E-37
6294	ENSMUSG00	67890	Ufm1	1	protein codi	MGI Symbol	5	ubiquitin-fol	1286.94	1287	1457.06	1446.01	1559	1552.79	2225	2637.74	2014	3189.36	3845.93	3708	22.865859	22.355993	25.902568	23.77009	25.519494	27.224776	30.238526	32.079346	29.314076	48.64079	51.965198	53.229393	0.1052341	5.0376308	0.268491	0.7390421	0.3658335	5.0376308	0.0001031	0.0005647	1.1130676	5.0376308	2.71E-32	3.33E-31
6307	ENSMUSG00	30953	Schip1	1	protein codi	MGI Symbol	8	schwannomi	390	414	408	451	458	478	690.25	740	560	1021	1191	1059	6.9293711	7.1914382	7.2531316	7.4137181	7.4970676	8.3806844	9.3807383	8.9996423	8.1508852	15.571226	16.09248	15.202246	0.1231913	3.3094596	0.2481511	0.7239214	0.3124788	3.3094596	0.0027182	0.008883	1.1325714	3.3094596	6.56E-29	7.04E-28
6322	ENSMUSG00	97064	Wwtr1	1	protein codi	MGI Symbol	2	WW domain	192	205	229	179	214	188	318	364	291	576	736	756	3.4113827	3.5609779	4.0709979	2.9424735	3.5029966	3.2961688	4.321731	4.4268511	4.2355493	8.7845508	9.9446391	10.852595	-0.180802	2.4224503	0.1422606	0.5802817	0.2343008	2.4224503	0.0459496	0.0869564	1.4219168	2.4224503	1.02E-37	1.57E-36
6326	ENSMUSG00	28146	Serp1	1	protein codi	MGI Symbol	1	stress-associ	2793	2682	2953	2754	2939	2754	4930	5336	4372	6231	7640	7226	49.624958	46.588013	52.496318	45.271352	48.108912	48.285366	67.00042	64.894718	63.635125	95.028709	103.22968	103.73128	-0.070002	6.0389351	0.4492867	0.8529148	0.394943	6.0389351	1.71E-05	0.0001159	1.0220716	6.0389351	1.51E-28	1.60E-27
6356	ENSMUSG00	19221	Ptgfrn	1	protein codi	MGI Symbol	3	prostaglandi	633	543	731	619	623	623	1141	1142	844	1511	2338	1934	11.246902	9.4322487	12.995194	10.175369	10.197976	10.922942	15.506588	13.888637	12.284548	23.044195	31.590443	27.763119	-0.105588	3.9864833	0.2999611	0.7607128	0.308592	3.9864833	0.0018687	0.0065317	1.2919354	3.9864833	5.47E-40	9.00E-39
6377	ENSMUSG00	229706	Slc6a17	1	protein codi	MGI Symbol	10	solute carrie	1452	1403	1552	1727	1916	1752	2720	2879	2433	4342	5033	5062	25.798582	24.370985	27.590344	28.389116	31.363278	30.717488	36.965749	35.013473	35.412685	66.219652	68.004577	72.666447	0.2184201	5.3329534	0.0206809	0.2057918	0.4657815	5.3329534	6.70E-07	6.23E-06	1.4117992	5.3329534	6.31E-51	1.41E-49
6392	ENSMUSG00	18160	Npr1	1	protein codi	MGI Symbol	5	natriuretic p	228.64	183.88	204.32	380.05	419.75	328.9	463.5	487.59	493.57	837.49	776.93	712.7	4.0623882	3.1941103	3.6322545	6.2474137	6.8709479	5.766542	6.2991267	5.9299129	7.1839864	12.772523	10.497675	10.231011	0.7955098	2.8007633	4.63E-12	3.89E-10	0.8323114	2.8007633	1.77E-13	3.59E-12	1.6203509	2.8007633	8.75E-49	1.84E-47
6395	ENSMUSG00	68328	Rab13	1	protein codi	MGI Symbol	3	RAB13, mem	144	143	175	158	175	139	236	264	190	356	424	420	2.558537	2.4839992	3.1110246	2.5972671	2.8646001	2.4370609	3.2073223	3.2106832	2.7654789	5.4293404	5.7289769	6.0292193	-0.043923	1.8470734	0.7394901	0.9533391	0.1745043	1.8470734	0.1677516	0.2447988	1.0762889	1.8470734	1.26E-19	8.02E-19
6415	ENSMUSG00	58193	Extl2	1	protein codi	MGI Symbol	6	exostosin-lik	712	717	823	806	836	800	1216	1494	1275	1688	2213	2151	12.650544	12.454737	14.630704	13.24935	13.684604	14.02625	16.525864	18.169548	18.557819	25.743614	29.901476	30.878216	0.0437473	4.2064368	0.6630156	0.9317527	0.4224579	4.2064368	1.45E-05	0.0001	1.1229893	4.2064368	3.48E-31	4.17E-30
6429	ENSMUSG00	66815	Mcub	1	protein codi	MGI Symbol	4	mitochondri	343	358	378	404	395	359	781	921	674	981	1307	1194	6.0942931	6.2186833	6.7198131	6.6411134	6.4658116	6.2942797	10.614063	11.200906	9.8101725	14.961188	17.659842	17.140209	0.027982	3.3349838	0.8067766	0.9712809	0.7334983	3.3349838	1.67E-12	3.12E-11	1.3869519	3.3349838	4.98E-42	8.72E-41
6434	ENSMUSG00	66350	Pla2g12a	1	protein codi	MGI Symbol	4	phospholipa	950	803	979	985	988	934	1861	1966	1503	2566	3371	3198	16.879237	13.948611	17.40396	16.191823	16.172713	16.375647	25.291639	23.90986	21.876394	39.133954	45.548069	45.908198	0.0153182	4.6436445	0.8800818	0.9858245	0.5598701	4.6436445	5.40E-09	6.88E-08	1.4373444	4.6436445	5.73E-51	1.29E-49
6462	ENSMUSG00	19220	Ptgfr	1	protein codi	MGI Symbol	3	prostaglandi	504.77	487.85	584.27	566.65	569.71	475.74	1827.14	2012.44	1412	2730.5	3102.26	2880.15	8.9685606	8.4742588	10.386733	9.314819	9.3256646	8.3410602	24.83147	24.474649	20.551875	41.642736	41.916924	41.345371	-0.043825	4.3893206	0.6743601	0.9358476	1.3284726	4.3893206	2.93E-41	2.30E-39	2.1661534	4.3893206	1.29E-106	8.01E-105
6470	ENSMUSG00	14466	Gba	1	protein codi	MGI Symbol	5	glucosidase,	797	829	885	834	908	791	1481	1715	1432	1895	2184	2177	14.160792	14.400247	15.732896	13.709625	14.863182	13.868455	20.127307	20.857279	20.842978	28.900562	29.509636	31.251453	-0.061462	4.3177731	0.5352926	0.8851661	0.4811607	4.3177731	6.28E-07	5.88E-06	1.017381	4.3177731	3.54E-26	3.36E-25
6478	ENSMUSG00	16905	Lmna	1	protein codi	MGI Symbol	7	lamin A [Sou	534	567	562	594	612	562	1289	1301	959	2914	3893	3908	9.4879081	9.8491437	9.9908332	9.7644092	10.017916	9.8534406	17.51796	15.822344	13.958391	44.441287	52.601196	56.10045	0.0150695	4.4436921	0.8894224	0.986428	0.6899287	4.4436921	3.79E-12	6.88E-11	2.3844996	4.4436921	7.85E-129	7.17E-127
6486	ENSMUSG00	73182	Pear1	1	protein codi	MGI Symbol	17	platelet endo	208	173	199	238.92	239	238.56	389	403	307	813.63	992	880.3	3.6956646	3.0051179	3.5376794	3.9274624	3.9122252	4.1826278	5.2866457	4.9011565	4.4684317	12.408636	13.403644	12.636956	0.2316079	2.672705	0.0559186	0.377292	0.5188649	2.672705	8.72E-06	6.33E-05	1.9093252	2.672705	1.72E-66	5.26E-65
6513	ENSMUSG00	14630	Gclm	1	protein codi	MGI Symbol	7	glutamate-cy	1541.53	1444.69	1563.68	1691.33	1770.29	1664.88	3023.4	3723.66	2785.29	4927.41	6525.28	5154.81	27.389317	25.095166	27.797982	27.802758	28.978131	29.190029	41.089061	45.285957	40.54032	75.147715	88.167873	73.998761	0.0987747	5.4701324	0.2963147	0.7589301	0.661005	5.4701324	1.59E-12	2.99E-11	1.5637863	5.4701324	6.39E-62	1.85E-60
6514	ENSMUSG00	11304	Abca4	1	protein codi	MGI Symbol	10	ATP-binding	785	677	735	674	717	647	1905	2073	1421	3221	4585	3734	13.94758	11.759912	13.066303	11.079481	11.736676	11.34373	25.889615	25.21116	20.682871	49.12333	61.951319	53.60263	-0.182523	4.6967973	0.0687228	0.4138586	0.889318	4.6967973	4.48E-20	1.45E-18	2.0867997	4.6967973	3.00E-102	1.70E-100
653	ENSMUSG00	70186	Fam162a	1	protein codi	MGI Symbol	4	family with s	387	425	389.95	450.92	429.84	401.65	706	776	632.91	1042	1608.98	1376.98	6.8760683	7.3825151	6.9322516	7.4124031	7.0361125	7.0420542	9.5947863	9.4374627	9.2121013	15.891496	21.740116	19.766939	0.0202499	3.4318075	0.8580512	0.98209	0.414445	3.4318075	6.48E-05	0.0003748	1.4381942	3.4318075	4.75E-46	9.34E-45
6536	ENSMUSG00	18033	Nfkb1	1	protein codi	MGI Symbol	10	nuclear fact	163	175	142	231	230	203	286	328	218	461	553	564	2.8961218	3.0398592	2.5243742	3.7972703	3.7649029	3.5591609	3.8868398	3.9890306	3.1730232	7.0306909	7.4719911	8.0963802	0.3943802	2.1721191	0.001585	0.0297583	0.3878989	2.1721191	0.0015805	0.0057132	1.4168172	2.1721191	2.46E-34	3.29E-33
6537	ENSMUSG00	110173	Manba	1	protein codi	MGI Symbol	11	mannosidas	193	166	214	192	196	184	355	371	338	452	620	547	3.4291503	2.8835235	3.8043386	3.1561727	3.2083521	3.2260375	4.8245739	4.5119828	4.9196414	6.8934322	8.3772775	7.8523403	-0.076413	2.273855	0.5410397	0.8882415	0.4940736	2.273855	2.42E-05	0.000158	1.1942023	2.273855	5.44E-26	5.08E-25
6541	ENSMUSG00	68151	Wls	1	protein codi	MGI Symbol	7	wntless WN	3723	3366	3829	3846	4154	3680	7344	7873	6868	9699	11428	11339	66.148843	58.469519	68.069218	63.222084	67.997421	64.52075	99.807523	95.748897	99.964785	147.91903	154.41214	162.77456	0.0227393	6.582911	0.8053337	0.9706794	0.6169831	6.582911	1.51E-11	2.58E-10	1.2713338	6.582911	2.87E-43	5.25E-42
6556	ENSMUSG00	69219	Ddah1	1	protein codi	MGI Symbol	3	dimethylargi	458	438	417	478	501	477	489	498	446	1630	1975	1791	8.1375691	7.6083332	7.4131272	7.8575549	8.2009408	8.3631516	6.6456806	6.056516	6.4915978	24.859059	26.685682	25.710314	0.0763863	3.5952145	0.4728147	0.864464	-0.271695	3.5952145	0.010215	0.0261499	1.7380188	3.5952145	7.69E-68	2.45E-66
6559	ENSMUSG00	12972	Cryz	1	protein codi	MGI Symbol	11	crystallin, ze	667.48	619.84	707.95	643.43	708.82	647.45	1431.23	1597.78	1333.69	1616.29	2232.08	2116	11.85953	10.767007	12.585428	10.576959	11.602776	11.351619	19.450915	19.431687	19.412061	24.649968	30.15928	30.375781	-0.070438	4.1535145	0.4877017	0.8696613	0.7273981	4.1535145	1.01E-13	2.09E-12	1.2750388	4.1535145	3.28E-39	5.31E-38
6561	ENSMUSG00	60599	Trp53inp1	1	protein codi	MGI Symbol	4	transformati	182	142	181	149	187	160	355	389	339	599	706	584	3.2337065	2.4666286	3.2176883	2.4493215	3.0610298	2.80525	4.8245739	4.730893	4.9341966	9.1353228	9.539287	8.3834858	-0.099963	2.3196661	0.4432878	0.850865	0.7002368	2.3196661	3.34E-09	4.34E-08	1.601843	2.3196661	1.05E-44	2.01E-43
6566	ENSMUSG00	72519	Pip4p2	1	protein codi	MGI Symbol	1	phosphatidy	642	602	625	697	735	701	1119	1278	989	1330	1749	1601	11.406811	10.457115	11.110802	11.457564	12.03132	12.290502	15.2076	15.542625	14.395045	20.283772	23.63203	22.98281	0.1176779	3.9212975	0.2447154	0.72048	0.453687	3.9212975	4.50E-06	3.51E-05	1.0210391	3.9212975	2.02E-25	1.80E-24
6585	ENSMUSG00	53378	Sdcbp	1	protein codi	MGI Symbol	8	syndecan bir	834	777	838	906	1008	899	1312	1338	1171	1950	2599	2317	14.818194	13.496975	14.897363	14.89319	16.500096	15.761998	17.830538	16.272326	17.044083	29.739365	35.117007	33.261193	0.1260746	4.3255715	0.2007105	0.6709184	0.2430086	4.3255715	0.0124365	0.0306317	1.1834105	4.3255715	8.92E-35	1.22E-33
6601	ENSMUSG00	52187	Rragd	1	protein codi	MGI Symbol	4	Ras-related (	627	622	595	545	668	589	939	919	837	2000	2302	2091	11.140297	10.804528	10.577484	8.9589277	10.934588	10.326827	12.761338	11.176583	12.182662	30.501913	31.104021	30.016899	-0.106018	3.9968602	0.3002936	0.7609003	0.150706	3.9968602	0.1312472	0.2013644	1.4941566	3.9968602	8.61E-53	2.00E-51
6613	ENSMUSG00	73205	C9orf72	1	protein codi	MGI Symbol	7	C9orf72, me	393.89	343	393	399.27	408	387.97	604.51	585.96	523	854.98	1252.99	1127	6.9984871	5.9581239	6.9864724	6.5633597	6.6786104	6.8022053	8.2155018	7.1262573	7.6123445	13.039263	16.930073	16.178405	0.0074798	3.1967669	0.9545976	1	0.202374	3.1967669	0.0554799	0.101163	1.2115133	3.1967669	1.71E-32	2.13E-31
662	ENSMUSG00	20848	Stat3	1	protein codi	MGI Symbol	14	signal transc	656	546	593	667	666	633	1308	1563	1236	2436	3417	2955	11.655558	9.4843606	10.541929	10.964412	10.901849	11.09827	17.776176	19.008704	17.990168	37.15133	46.169609	42.419864	0.0574257	4.3620717	0.5762271	0.9028794	0.7902966	4.3620717	1.03E-15	2.50E-14	1.989121	4.3620717	1.27E-91	5.83E-90
6628	ENSMUSG00	94181	Nans	1	protein codi	MGI Symbol	4	N-acetylneu	257	250	259	285	303	291	476	513	447	648	919	853	4.5662779	4.3426559	4.6043164	4.6849438	4.9598504	5.1020485	6.4690061	6.2389412	6.506153	9.8826197	12.417287	12.245057	0.1257028	2.7909331	0.2825568	0.749253	0.5072676	2.7909331	4.45E-06	3.48E-05	1.3550169	2.7909331	2.86E-36	4.16E-35
663	ENSMUSG00	20850	Stat5a	1	protein codi	MGI Symbol	7	signal transc	707.15	614.79	712.83	802.1	794.89	732.16	1459.14	1678.63	1368.11	1986.55	2516.37	2226.51	12.564371	10.679286	12.672181	13.18524	13.011668	12.836824	19.830222	20.414959	19.913049	30.296787	34.000532	31.962183	0.1203978	4.2773262	0.230407	0.7109017	0.744412	4.2773262	2.42E-14	5.31E-13	1.4226415	4.2773262	1.51E-48	3.13E-47
6651	ENSMUSG00	22234	Ugcg	1	protein codi	MGI Symbol	4	UDP-glucose	454	388	445	464	449	479	845.18	780	666	1154	1392	1345	8.0664987	6.739802	7.9108911	7.6274173	7.3497453	8.3982172	11.486291	9.4861094	9.6937313	17.599604	18.808339	19.307857	0.0408505	3.4759627	0.7050943	0.9439679	0.432746	3.4759627	2.55E-05	0.0001658	1.2946514	3.4759627	6.64E-38	1.03E-36
6652	ENSMUSG00	230257	Ptbp3	1	protein codi	MGI Symbol	13	polypyrimidi	540	493	587	494	606	545	851	985	812	1277	1637	1543	9.5945138	8.5637175	10.435265	8.1205693	9.9197008	9.5553828	11.565387	11.979254	11.818784	19.475471	22.118715	22.150203	-0.051214	3.7033155	0.624475	0.918746	0.3069127	3.7033155	0.0023151	0.0077687	1.1571012	3.7033155	1.25E-31	1.52E-30
6673	ENSMUSG00	14595	B4galt1	1	protein codi	MGI Symbol	2	UDP-Gal:bet	361	301	368	363	337	352	642	697	562	941	1156	990	6.4141102	5.2285577	6.5420403	5.967139	5.5164013	6.17155	8.725004	8.4766901	8.1799955	14.35115	15.619569	14.211731	-0.042662	3.1494397	0.7066593	0.9445988	0.4817034	3.1494397	5.12E-06	3.94E-05	1.2813357	3.1494397	1.31E-35	1.86E-34
670	ENSMUSG00	65964	Map3k20	1	protein codi	MGI Symbol	6	mitogen-acti	165	155	160	173	177	193	265	254	171	444	494	551	2.931657	2.6924467	2.8443653	2.8438431	2.8973383	3.3838328	3.6014425	3.0890664	2.488931	6.7714246	6.6747986	7.9097615	0.1061584	2.0266911	0.4113706	0.837269	0.1193702	2.0266911	0.3607498	0.4530235	1.3340292	2.0266911	1.98E-30	2.30E-29

Table S2. Differentially expressed genes (> 2-fold) in *Trpm3*-mutant lenses summarized in Fig. 9A.

6709	ENSMUSG00	22004	Tpm2	1	protein codi	MGI Symbol	6	tropomyosir	324	291	368	444	357	366	627	657	576	1193	1274	1327	5.7567083	5.0548515	6.5420403	7.2986493	5.8437841	6.4170094	8.5211488	7.9902229	8.3837676	18.194391	17.213954	19.049462	0.1731413	3.290283	0.1161164	0.5324511	0.5204708	3.290283	9.24E-07	8.34E-06	1.6498672	3.290283	2.59E-58	6.88E-57
6714	ENSMUSG00	230103	Npr2	1	protein codi	MGI Symbol	10	natriuretic p	646	578	596	652	668.18	663	1034.01	1100	1038	1465.01	1629.02	1555.11	11.477881	10.040221	10.595261	10.717836	10.937534	11.624255	14.052557	13.377847	15.108248	22.342803	22.010891	22.324046	0.0512883	3.8708335	0.6166195	0.9156819	0.4049598	3.8708335	4.43E-05	0.0002674	1.0539986	3.8708335	6.55E-27	6.39E-26
6718	ENSMUSG00	12757	Clta	1	protein codi	MGI Symbol	6	clathrin, ligh	2339	2157	2362	2443	2548	2300	4498	4869	3928	5490	6709	6426	41.558459	37.468435	41.989943	40.15901	41.708577	40.325469	61.129389	59.215214	57.172637	83.72775	90.65025	92.247055	0.0140326	5.8425145	0.8819076	0.9859489	0.55283	5.8425145	2.13E-09	2.86E-08	1.1396834	5.8425145	1.01E-34	1.37E-33
6728	ENSMUSG00	70122	Mllt3	1	protein codi	MGI Symbol	8	myeloid/lym	314	296	347	276	380	268	511	555	401	788	961	905	5.5790321	5.1417046	6.1687173	4.5369982	6.2202744	4.6987938	6.9446683	6.7497317	5.836616	12.017754	12.98478	12.991532	-0.126424	2.9199098	0.2595182	0.7337405	0.211338	2.9199098	0.0521667	0.0961552	1.1700763	2.9199098	2.53E-29	2.78E-28
6729	ENSMUSG00	66775	Hacd4	1	protein codi	MGI Symbol	3	3-hydroxyac	1657.65	1448.12	1822.49	1918.75	2146.31	1890.99	3269.45	3586.41	3177.32	4782.63	5290.71	5099.66	29.452492	25.154748	32.398921	31.541179	35.133256	33.154373	44.432966	43.616766	46.246376	72.939681	71.486687	73.207067	0.1984827	5.4917954	0.0346693	0.290709	0.6262133	5.4917954	1.88E-11	3.20E-10	1.3227724	5.4917954	2.77E-45	5.36E-44
6740	ENSMUSG00	11639	Ak4	1	protein codi	MGI Symbol	7	adenylate ki	182.89	193.28	148.33	217.37	160.81	184.38	345.14	303.39	275.32	452.26	541.09	520.46	3.2495197	3.3573942	2.6369044	3.5732149	2.6323219	3.2327	4.690573	3.6897317	4.0073245	6.8973975	7.3110663	7.4713511	0.028937	2.1596271	0.8321639	0.9780596	0.419945	2.1596271	0.0004522	0.0020177	1.2291878	2.1596271	1.10E-26	1.06E-25
675	ENSMUSG00	26360	Angptl2	1	protein codi	MGI Symbol	4	angiopoietin	296	308	272	350	325	330	827	887	805	1734	2222	2391	5.259215	5.3501521	4.8354211	5.7534398	5.3199715	5.7858281	11.239219	10.787409	11.716897	26.445158	30.023082	34.323484	0.1258551	3.7242256	0.2647498	0.735798	1.1264312	3.7242256	8.11E-27	3.55E-25	2.5550854	3.7242256	5.14E-137	5.42E-135
6774	ENSMUSG00	18786	Plaa	1	protein codi	MGI Symbol	6	phospholipa	1835	1765	2007	2063	2038	1921	3334	3836	3124	4467	5066	5015	32.603579	30.659151	35.679008	33.912418	33.360314	33.680533	45.310223	46.6522	45.470295	68.126022	68.450464	71.991749	0.0290974	5.5104739	0.7579314	0.9597905	0.4741753	5.5104739	3.32E-07	3.27E-06	1.0758949	5.5104739	6.90E-31	8.13E-30
6779	ENSMUSG00	14726	Pdpn	1	protein codi	MGI Symbol	2	podoplanin |	1073	934	1070	1069	1064	1005	1550	1616	1288	2526	3341	3047	19.064654	16.224163	19.021693	17.572649	17.416768	17.620477	21.065041	19.653273	18.747036	38.523916	45.142717	43.74055	-0.045751	4.6187571	0.6392506	0.923451	0.1311174	4.6187571	0.1727606	0.2506453	1.2304691	4.6187571	2.92E-38	4.58E-37
6807	ENSMUSG00	60613	Kcnq4	1	protein codi	MGI Symbol	2	potassium v	272	211	210	299	253	276	770	816	764	1334	1674	1587	4.8327922	3.6652016	3.7332295	4.9150814	4.1413932	4.8390563	10.464569	9.9239298	11.120136	20.344776	22.618649	22.781836	0.1838035	3.3818775	0.116423	0.5328909	1.3643468	3.3818775	5.30E-36	3.38E-34	2.4265191	3.3818775	3.44E-112	2.39E-110
6854	ENSMUSG00	18710	Pik3r3	1	protein codi	MGI Symbol	3	phosphoinos	547	439	422	458	406.1	482.91	1205	1298	1054	2275	2930	2514.2	9.7188872	7.6257038	7.5020136	7.5287869	6.6475091	8.4667705	16.37637	15.785859	15.34113	34.695926	39.589392	36.092055	-0.134839	4.109089	0.2045748	0.676928	0.9351635	4.109089	1.12E-20	3.79E-19	2.1515257	4.109089	5.93E-104	3.43E-102
6894	ENSMUSG00	19248	Ptpn12	1	protein codi	MGI Symbol	7	protein tyros	305	286	268	337	326	302	481	511	444	703	951	976	5.4191236	4.9679984	4.7643119	5.5397406	5.3363407	5.2949094	6.5369578	6.2146178	6.4624875	10.721422	12.849663	14.010757	0.0941617	2.8921811	0.41285	0.837543	0.3421607	2.8921811	0.0017406	0.0061863	1.3111346	2.8921811	8.22E-36	1.17E-34
690	ENSMUSG00	14792	Lpcat3	1	protein codi	MGI Symbol	9	lysophospha	428.5	392.91	428.29	407.83	453.71	415.65	652.62	775.51	600.57	980.46	1120.49	1075.62	7.6134244	6.8250918	7.6138327	6.7040724	7.426844	7.2875135	8.8693335	9.4315035	8.7413877	14.952953	15.139767	15.440831	-0.042	3.2849715	0.7006484	0.9430688	0.2951461	3.2849715	0.0045224	0.0135272	1.0460798	3.2849715	6.06E-25	5.27E-24
6927	ENSMUSG00	71817	Tmem50a	1	protein codi	MGI Symbol	2	transmembr	691	666	773	781	833	758	1265	1416	1118	1843	2469	2166	12.277424	11.568835	13.74184	12.83839	13.635496	13.289872	17.191791	17.220937	16.27266	28.107512	33.360481	31.093545	0.0813277	4.2077882	0.4174029	0.8400992	0.4316775	4.2077882	1.03E-05	7.42E-05	1.300539	4.2077882	4.80E-41	8.17E-40
693	ENSMUSG00	12728	Clcn5	1	protein codi	MGI Symbol	5	chloride cha	220	212	235	215	300	240	370	469	379	796	772	782	3.908876	3.6825722	4.1776616	3.5342559	4.910743	4.207875	5.0284291	5.7038273	5.5164026	12.139761	10.431062	11.225832	0.1048678	2.6528077	0.3791642	0.8163671	0.4660702	2.6528077	3.97E-05	0.0002434	1.5209016	2.6528077	5.79E-44	1.08E-42
6931	ENSMUSG00	16765	Stmn1	1	protein codi	MGI Symbol	5	stathmin 1 [	994.39	892.84	1019.67	909.76	939.57	971	1591.78	1794.05	1547.61	2600.35	3379.94	2937.88	17.667942	15.509188	18.126963	14.954998	15.379956	17.024361	21.632846	21.81866	22.525699	39.657824	45.668864	42.174102	-0.115644	4.6114023	0.2374312	0.7163933	0.362895	4.6114023	0.0001532	0.0007964	1.3136429	4.6114023	3.89E-43	7.06E-42
6944	ENSMUSG00	245877	Map7d1	1	protein codi	MGI Symbol	6	MAP7 doma	1040	1043	1082	1145	1189	1134	2360	2564	2400	3420	3893	3457	18.478323	18.117561	19.235021	18.821967	19.462911	19.882209	32.073223	31.182544	34.932365	52.158271	52.601196	49.626217	0.0590716	4.93848	0.5426124	0.8892216	0.8141485	4.93848	9.82E-18	2.78E-16	1.4673572	4.93848	1.18E-53	2.81E-52
6947	ENSMUSG00	20544	Slc9a1	1	protein codi	MGI Symbol	5	solute carrie	220	218	230	261	248	254	335	424	358	610	777	657	3.908876	3.786796	4.0887752	4.2904222	4.0595475	4.4533344	4.5527669	5.1565518	5.2107445	9.3030833	10.49862	9.4314216	0.1191584	2.5370613	0.3196259	0.7756777	0.340505	2.5370613	0.002901	0.0093805	1.3111837	2.5370613	8.77E-33	1.10E-31
6999	ENSMUSG00	19744	Rheb	1	protein codi	MGI Symbol	4	Ras homolog	1049	957	1227	1161	1205	1135	2231	2438	2123	2846	3471	3155	18.638232	16.623687	21.812727	19.084982	19.724818	19.899742	30.320068	29.650173	30.900588	43.404222	46.899242	45.290921	0.0408688	4.8392695	0.6748448	0.9358476	0.6709813	4.8392695	1.57E-12	2.96E-11	1.2486005	4.8392695	1.69E-39	2.75E-38
7019	ENSMUSG00	18669	Abcb1b	1	protein codi	MGI Symbol	9	ATP-binding	65.99	78.42	75.45	119.44	110.01	107.96	369.05	366.13	279.73	539.33	673.01	571.1	1.1724851	1.3622043	1.341296	1.9634024	1.8007694	1.8928424	5.0155183	4.4527554	4.0715127	8.2252983	9.0935348	8.1983027	0.5441891	2.0613616	0.0004508	0.010221	1.8035203	2.0613616	9.27E-41	7.10E-39	2.7172201	2.0613616	3.77E-98	1.96E-96
7045	ENSMUSG00	11610	Agtrap	1	protein codi	MGI Symbol	3	angiotensin	397	343	415	501	477	450	569	627	562	907	1081	977	7.0537444	5.9581239	7.3775726	8.2356381	7.8080813	7.8897656	7.7329085	7.6253726	8.1799955	13.832617	14.606189	14.025112	0.2316426	3.2106929	0.0301016	0.2651774	0.2068903	3.2106929	0.0495814	0.0923937	1.0588554	3.2106929	3.88E-25	3.42E-24
705	ENSMUSG00	16795	Large1	1	protein codi	MGI Symbol	4	LARGE xylos	262	222	233	228	267	240	374	385	353	536	708	586	4.655116	3.8562785	4.142107	3.7479551	4.3705612	4.207875	5.0827905	4.6822463	5.1379687	8.1745126	9.5663105	8.4121964	-0.037635	2.4778712	0.7605562	0.9601813	0.2350289	2.4778712	0.0382254	0.0750716	1.0485061	2.4778712	1.31E-21	9.51E-21
7074	ENSMUSG00	66469	Prxl2b	1	protein codi	MGI Symbol	3	peroxiredoxi	216	245	203	228	174	205	408	379	330	530	640	537	3.8378055	4.2558028	3.6087885	3.7479551	2.8482309	3.5942266	5.5448624	4.6092762	4.8032002	8.0830068	8.6475123	7.7087875	-0.200897	2.373326	0.1018559	0.5024043	0.3525839	2.373326	0.0020316	0.0069879	1.0620416	2.373326	1.08E-21	7.89E-21
7093	ENSMUSG00	66768	5730480H0	1	protein codi	MGI Symbol	9	RIKEN cDNA	206	204	217	227	244	229	356	445	354	472	644	728	3.6601294	3.5436072	3.8576705	3.7315167	3.9940709	4.0150141	4.8381642	5.411947	5.1525238	7.1984514	8.7015592	10.450647	0.0857948	2.448767	0.4779587	0.8649415	0.4784758	2.448767	3.03E-05	0.0001928	1.2531692	2.448767	2.11E-29	2.32E-28
710	ENSMUSG00	64143	Ralb	1	protein codi	MGI Symbol	3	v-ral simian	403	425	439	515	440	485	678	713	617	1075	1344	1181	7.1603501	7.3825151	7.8042274	8.4657757	7.202423	8.5034141	9.2142566	8.6712769	8.9805288	16.394778	18.159776	16.95359	0.1127134	3.39016	0.2884659	0.7530712	0.265308	3.39016	0.0105285	0.0268178	1.2049055	3.39016	7.29E-33	9.20E-32
7147	ENSMUSG00	66681	Pgm2	1	protein codi	MGI Symbol	4	phosphogluc	131	123	138	125	153	145	224	252	209	300	326	350	2.327558	2.1365867	2.4532651	2.0547999	2.5044789	2.5422578	3.0442382	3.064743	3.0420268	4.5752869	4.4048266	5.0243494	0.0371329	1.6594715	0.7932479	0.9685832	0.4040194	1.6594715	0.0016963	0.0060651	1.0170587	1.6594715	1.87E-16	9.49E-16
7151	ENSMUSG00	52206	Anapc4	1	protein codi	MGI Symbol	12	anaphase pr	731	658	657	804	795	766	1288	1395	1202	1437	1778	1829	12.988129	11.42987	11.679675	13.216473	13.013469	13.430134	17.504369	16.965542	17.495293	21.915624	24.02387	26.255814	0.1357717	4.0656008	0.1749663	0.6327705	0.5255126	4.0656008	8.08E-08	8.77E-07	1.0002336	4.0656008	9.53E-25	8.16E-24
7157	ENSMUSG00	68303	Fam114a1	1	protein codi	MGI Symbol	2	family with s	1438	1606	1990	1870	1972	1788	3018	3735	2886	4842	5888	5905	25.549835	27.897222	35.376794	30.739807	32.27995	31.348669	41.015673	45.42387	42.006169	73.84513	79.557113	84.767952	0.0874277	5.5212366	0.3532626	0.7971484	0.5323689	5.5212366	1.13E-08	1.39E-07	1.423042	5.5212366	4.67E-52	1.07E-50
7229	ENSMUSG00	13602	Sparcl1	1	protein codi	MGI Symbol	2	SPARC-like 1	335	253	315	239	234	225	120	227	225	1074	1759	1645	5.9521521	4.3947678	5.5998443	3.9287775	3.8303795	3.9448828	1.6308419	2.7607011	3.2749092	16.379527	23.767147	23.614442	-0.445518	3.0619377	0.0001209	0.0031898	-1.057776	3.0619377	4.51E-19	1.40E-17	2.0007676	3.0619377	1.71E-84	6.99E-83
726	ENSMUSG00	50767	Pnpla6	1	protein codi	MGI Symbol	20	patatin-like	1385	1233	1163	1495	1653	1513	2093	2123	2016	3218	3751	3607	24.608151	21.417979	20.674981	24.575407	27.058194	26.527145	28.4446	25.819244	29.343187	49.077577	50.682529	51.779509	0.2287094	4.9885677	0.0162203	0.1759052	0.3255032	4.9885677	0.0005689	0.0024631	1.1839305	4.9885677	3.95E-36	5.71E-35
7271	ENSMUSG00	27428	Shroom3	1	protein codi	MGI Symbol	11	shroom fam	161	145	188	433	424	425	290	304	271	538	754	546	2.8605865	2.5187404	3.3421293	7.1178269	6.9405167	7.4514453	3.9412012	3.6971503	3.9444462	8.2050145	10.18785	7.8379851	1.3021111	2.5161302	1.09E-28	2.80E-26	0.4087613	2.5161302	0.0007358	0.0030526	1.5901793	2.5161302	8.76E-44	1.63E-42
7278	ENSMUSG00	56334	Tmed2	1	protein codi	MGI Symbol	5	transmembr	1863.75	1594.87	1973.96	1798.68	1990.43	1798.13	3252.91	3576.77	3124.23	4171.96	5055.41	4738.81	33.114398	27.703887	35.091646	29.56742	32.581634	31.526276	44.208182	43.499528	45.473643	63.62638	68.307375	68.026963	-0.033899	5.4480718	0.7191753	0.9477493	0.4736511	5.4480718	3.51E-07	3.44E-06	1.0601203	5.4480718	5.40E-30	6.12E-29
7301	ENSMUSG00	12492	Scarb2	1	protein codi	MGI Symbol	2	scavenger re	878	782	882	825	863	833	1620	1790	1411	2478	2969	2862	15.599969	13.583828	15.679564	13.561679	14.126571	14.604833	22.016365	21.769405	20.53732	37.79187	40.11635	41.084823	-0.085124	4.5012071	0.3898885	0.8246852	0.5201322	4.5012071	6.95E-08	7.66E-07	1.4074215	4.5012071	1.21E-48	2.52E-47
741	ENSMUSG00	11826	Aqp1	1	protein codi	MGI Symbol	1	aquaporin 1	461	493	405	558	458	577	948	733	594	2142	2681	2488	8.190872	8.5637175	7.1997998	9.1726269	7.4970676	10.116433	12.883651	8.9145105	8.6457603	32.667548	36.224969	35.715947	0.1598335	3.963224	0.1258091	0.5479994	0.3453261	3.963224	0.0007457	0.0030895	2.1263366	3.963224	3.32E-101	1.86E-99
7422	ENSMUSG00	11867	Arpc1b	1	protein codi	MGI Symbol	11	actin related	354.11	301.49	340.24	335.91	299.35	338.35	685.19	651.39	534.71	1527.61	2055.48	1772.92	6.2916913	5.2370693	6.0485429	5.5218227	4.900103	5.9322271	9.3119712	7.9219959	7.7827854	23.297513	27.773107	25.45077	-0.104644	3.5123695	0.3526473	0.7966422	0.5093632	3.5123695	1.53E-06	1.32E-05	2.1226248	3.5123695	1.17E-96	5.89E-95
7444	ENSMUSG00	77521	Mtus2	1	protein codi	MGI Symbol	11	microtubule	148	166	144	175	156	179	463	609	405	1250	2278	1781	2.6296075	2.8835235	2.5599288	2.8767199	2.5535863	3.1383734	6.2923316	7.4064623	5.8948366	19.063695	30.779739	25.566761	0.0838204	3.2434373	0.5325363	0.8841481	1.2808711	3.2434373	8.64E-28	3.91E-26	3.2234267	3.2434373	4.09E-178	6.94E-176
7451	ENSMUSG00	12843	Col1a2	1	protein codi	MGI Symbol	8	collagen, typ	227	201	191	198	235	201	205	286	245	781	1523	951	4.0332493	3.4914954	3.3954611	3.2548031	3.8467487	3.5240953	2.7860215	3.4782401	3.5660123	11.910997	20.578377	13.651875	-0.039083	2.7143548	0.7609958	0.9601813	-0.151284	2.7143548	0.215075	0.29889	2.0809194	2.7143548	1.83E-80	7.14E-79
747	ENSMUSG00	79221	Hdac9	1	protein codi	MGI Symbol	12	histone deac	208	219	211	222	215	236	301	424	339	486	582	659	3.6956646	3.8041666	3.7510068	3.6493247	3.5193658	4.1377438	4.090695	5.1565518	4.9341966	7.4119648	7.8638315	9.4601321	0.0056573	2.3769496	0.9707174	1	0.3348397	2.3769496	0.0039421	0.0120455	1.1364761	2.3769496	2.06E-24	1.73E-23
7482	ENSMUSG00	13846	Ephb4	1	protein codi	MGI Symbol	5	Eph receptor	624	561	559	547	508	527	1132	1180	989	1581	1851	1629	11.086994	9.7449199	9.9375014	8.9918045	8.3155248	9.2397922	15.384275	14.350781	14.395045	24.111762	25.010227	23.384758	-0.213141	3.8674646	0.0386979	0.3128085	0.5202345	3.8674646	1.62E-07	1.68E-06	1.236679	3.8674646	2.41E-36	3.52E-35
7500	ENSMUSG00	27053	Asns	1	protein codi	MGI Symbol	7	asparagine s	450	362	305	643	515	535	507	571	561	1452	1505	1675	7.9954282	6.2881658	5.4220714	10.569891	8.4301088	9.3800547	6.8903069	6.9443186	8.1654403	22.144389	20.335166	24.045101	0.5262656	3.5187038	6.01E-07	2.70E-05	0.157397	3.5187038	0.1367847	0.2079793	1.754872	3.5187038	1.85E-67	5.79E-66
7519	ENSMUSG00	27055	Fkbp9	1	protein codi	MGI Symbol	2	FK506 bindir	3619	3471	3866	4126	4167	3957	8084	7930	6408	10495	13542	13404	64.30101	60.293435	68.726977	67.824836	68.21022	69.377339	109.86438	96.442112	93.269415	160.05879	182.97596	192.41823	0.087534	6.6855308	0.3398608	0.7880005	0.6319491	6.6855308	4.83E-12	8.69E-11	1.4698146	6.6855308	8.25E-57	2.13E-55
7528	ENSMUSG00	73998	Herc3	1	protein codi	MGI Symbol	2	hect domain	298	249	261	278	319	292	428	485	414	587	739	711	5.2947502	4.3252853	4.639871	4.569875	5.2217567	5.1195813	5.8166693	5.8984142	6.025833	8.9523113	9.9851743	10.206607	0.064478	2.67973	0.5799303	0.9029106	0.3151646	2.67973	0.0044104	0.013259	1.0319472	2.67973	5.08E-22	3.77E-21
7538	ENSMUSG00	71720	Osbpl3	1	protein codi	MGI Symbol	10	oxysterol bir	206	173	221	260	277	268	532	554	401	759	995	834	3.6601294	3.0051179	3.9287796	4.2739838	4.5342527	4.6987938	7.2300656	6.73757	5.836616	11.575476	13.444179	11.972307	0.3505084	2.775318	0.003179	0.052983	0.9042568	2.775318	1.44E-15	3.46E-14	1.8048846	2.775318	5.70E-60	1.57E-58
7549	ENSMUSG00	19242	Ptn	1	protein codi	MGI Symbol	2	pleiotrophin	1000	893	974	1137	1205	1098	2408	2453	1811	3243	4880	4584	17.767618	15.511967	17.315074	18.69046	19.724818	19.251028	32.72556	29.832598	26.35938	49.458851	65.937281	65.804622	0.1888559	4.9848059	0.0515389	0.3616096	0.8138474	4.9848059	1.50E-17	4.14E-16	1.8408609	4.9848059	4.07E-82	1.63E-80
7557	ENSMUSG00	22793	Zyx	1	protein codi	MGI Symbol	11	zyxin [Sourc	1429	1261	1323	1425	1457	1474.51	2445.43	2473	2066.42	3549	4133	3936	25.389926	21.904357	23.519346	23.424719	23.849842	25.852307	33.234247	30.075831	30.077057	54.125644	55.844013	56.502398	0.0462533	5.0764987	0.6284363	0.9209696	0.3992176	5.0764987	2.24E-05	0.0001476	1.2332401	5.0764987	3.46E-39	5.59E-38
7621	ENSMUSG00	69834	Rab43	1	protein codi	MGI Symbol	7	RAB43, mem	423.14	386.23	424.69	406.67	393.5	446.2	582.01	707.25	602.85	1091.65	1108.58	1027.28	7.51819	6.709056	7.5498345	6.6850039	6.4412579	7.823141	7.909719	8.6013473	8.7745734	16.648706	14.978842	14.746896	-0.056785	3.2635275	0.6049765	0.9119024	0.2156322	3.2635275	0.0390077	0.0762811	1.0901179	3.2635275	6.03E-27	5.90E-26
7643	ENSMUSG00	71699	Slc41a3	1	protein codi	MGI Symbol	2	solute carrie	214	197	232	454	458	482	487	564	432	708	925	791	3.8022703	3.4220129	4.1243297	7.4630333	7.4970676	8.4508156	6.6184999	6.8591868	6.2878257	10.797677	12.498358	11.35503	1.0439942	2.9081482	2.04E-20	3.53E-18	0.8015933	2.9081482	1.10E-12	2.11E-11	1.6111061	2.9081482	9.08E-49	1.90E-47
7648	ENSMUSG00	74122	Tmem43	1	protein codi	MGI Symbol	5	transmembr	500	439	509	527	580	565	908	949	889	1100	1257	1246	8.8838091	7.6257038	9.0486372	8.6630365	9.4941031	9.9060391	12.340037	11.541433	12.93593	16.776052	16.984255	17.886684	0.1347384	3.5752851	0.1956943	0.6634556	0.5261649	3.5752851	1.93E-07	1.99E-06	1.0150837	3.5752851	3.53E-24	2.92E-23
7649	ENSMUSG00	21366	Slc6a6	1	protein codi	MGI Symbol	11	solute carrie	2820	2296	2639	2664	2982	2703	5018	6417	4929	11221	14420	14857.86	50.104683	39.882952	46.914251	43.791896	48.812785	47.391192	68.196371	78.041492	71.742345	171.13098	194.83926	213.2888	0.0323033	6.4862986	0.7284253	0.9507204	0.6712496	6.4862986	3.11E-13	6.26E-12	2.0811511	6.4862986	8.23E-108	5.19E-106
7652	ENSMUSG00	16438	Itpr1	1	protein codi	MGI Symbol	13	inositol 1,4,5	1379.91	1495	1451	1491.99	1967.94	1690.96	2553.98	3606.93	2492.93	4271.85	4798.87	4687.93	24.517714	25.969082	25.794838	24.525927	32.213492	29.647285	34.709479	43.866324	36.284975	65.149798	64.841074	67.296566	0.1793718	5.3101268	0.0577862	0.3843113	0.590949	5.3101268	2.91E-10	4.26E-09	1.370794	5.3101268	4.13E-48	8.47E-47
7653	ENSMUSG00	20893	Bhlhe40	1	protein codi	MGI Symbol	4	basic helix-lo	248	197	294.99	243	242.98	255	371.99	339.99	244	1176.97	2040.98	1654.97	4.4063693	3.4220129	5.2441208	3.9945311	3.9773744	4.4708672	5.0554739	4.1348492	3.5514571	17.949918	27.577187	23.757564	-0.07124	3.183157	0.548675	0.891083	-0.034531	3.183157	0.7629456	0.8186111	2.4076311	3.183157	3.60E-112	2.48E-110
7654	ENSMUSG00	192193	Edem1	1	protein codi	MGI Symbol	5	ER degradat	208	200	188	168	213	174	369	396	315	509	631	634	3.6956646	3.4741247	3.3421293	2.7616511	3.4866275	3.0507094	5.0148388	4.8160248	4.5848729	7.7627368	8.5259067	9.10125	-0.176369	2.336591	0.1615291	0.614056	0.4559825	2.336591	8.59E-05	0.0004795	1.2723921	2.336591	8.13E-30	9.12E-29
7665	ENSMUSG00	69202	Ptms	1	protein codi	MGI Symbol	5	parathymosi	3015	2582	2888	2719	2742	2725	5513	5593	4433	7783	9240	8447	53.569369	44.85095	51.340794	44.696008	44.884191	47.776914	74.923594	68.020269	64.522989	118.69819	124.84846	121.25908	-0.124746	6.1643686	0.1773354	0.6359708	0.4703139	6.1643686	3.05E-07	3.02E-06	1.2845249	6.1643686	8.33E-44	1.55E-42
7666	ENSMUSG00	67784	Plxnd1	1	protein codi	MGI Symbol	5	plexin D1 [Sc	423	303	410	416	407	364	1141	1417	1136	2855	3627	3440	7.5157025	5.263299	7.2886862	6.8383741	6.6622413	6.3819438	15.506588	17.233099	16.534653	43.54148	49.007074	49.382177	-0.01228	4.2803069	0.9144054	0.9914851	1.296738	4.2803069	2.68E-37	1.83E-35	2.8227762	4.2803069	2.23E-171	3.58E-169
7709	ENSMUSG00	70686	Dusp16	1	protein codi	MGI Symbol	7	dual specific	373	370	374	470	513	427	635	598	608	860	1036	1033	6.6273216	6.4271308	6.648704	7.7260477	8.3973705	7.486511	8.6298716	7.2726839	8.8495325	13.115822	13.998161	14.829008	0.2614085	3.2071717	0.0146669	0.1643107	0.3272142	3.2071717	0.0018142	0.0064	1.0901301	3.2071717	2.04E-26	1.95E-25
771	ENSMUSG00	18008	Nes	1	protein codi	MGI Symbol	3	nestin [Sour	448	416	462	418	434	437	960	1113	927	1931	2078	2382	7.959893	7.2261795	8.2131049	6.8712509	7.1042082	7.6618391	13.046735	13.535948	13.492626	29.449597	28.077391	34.194286	-0.113243	3.8935637	0.2901608	0.7545724	0.776389	3.8935637	1.81E-14	4.00E-13	1.9706628	3.8935637	6.00E-87	2.52E-85
7745	ENSMUSG00	71323	Rassf8	1	protein codi	MGI Symbol	4	Ras associat	272	214	254	237	292	243	408	471	405	540	616	692	4.8327922	3.7173135	4.51543	3.8959007	4.7797898	4.2604734	5.5448624	5.7281507	5.8948366	8.2355164	8.3232306	9.9338565	-0.013453	2.5550155	0.9215568	0.9925021	0.3941745	2.5550155	0.0004213	0.0019041	1.0202274	2.5550155	9.91E-21	6.75E-20
7753	ENSMUSG00	52163	Camk1	1	protein codi	MGI Symbol	6	calcium/calr	398.61	347	344	420	460	455	705	715	596	803	1024	973.8	7.0823503	6.0276064	6.1153855	6.9041277	7.5298059	7.9774297	9.581196	8.6956003	8.6748707	12.246518	13.83602	13.979176	0.2207693	3.1907059	0.0402808	0.3175663	0.487343	3.1907059	3.28E-06	2.63E-05	1.059735	3.1907059	7.04E-25	6.10E-24
7779	ENSMUSG00	110595	Timp4	1	protein codi	MGI Symbol	2	tissue inhibi	763.55	656.93	1058.57	594.53	828.51	720.29	1692.36	2018	1877.38	3158.2	3908.39	2975.4	13.566465	11.411284	18.818499	9.7731216	13.561999	12.62871	22.999763	24.542268	27.325551	48.16557	52.809142	42.712712	-0.284066	4.6433084	0.0042573	0.0660302	0.7735283	4.6433084	9.28E-16	2.28E-14	1.7144761	4.6433084	6.86E-71	2.33E-69
7791	ENSMUSG00	22317	Vamp1	1	protein codi	MGI Symbol	6	vesicle-asso	568	513	397	606	597	564	808	850	766	1392	1593	1537	10.092007	8.91113	7.0575815	9.96167	9.7723785	9.8885063	10.981002	10.337427	11.149247	21.229331	21.524199	22.064071	0.1846758	3.6812314	0.0742336	0.4305368	0.3163868	3.6812314	0.0018302	0.0064301	1.3142694	3.6812314	6.17E-40	1.01E-38
7795	ENSMUSG00	12527	Cd9	1	protein codi	MGI Symbol	3	CD9 antigen	57	62	80	70	60.99	71.95	353	293	225	1392	2423.93	1909.97	1.0127542	1.0769787	1.4221827	1.150688	0.998354	1.2614859	4.7973932	3.5633719	3.2749092	21.229331	32.751507	27.418162	-0.044121	3.0896219	0.832383	0.9781838	1.7275964	3.0896219	2.07E-35	1.29E-33	4.5340569	3.0896219	9.63E-279	4.36E-276
782	ENSMUSG00	26395	Map2k1	1	protein codi	MGI Symbol	4	mitogen-acti	859	883	848	803	953	882	1483	1587	1301	1965	2715	2583	15.262384	15.338261	15.075136	13.200035	15.599793	15.463941	20.154487	19.300584	18.936253	29.968129	36.684369	37.079699	-0.045539	4.399042	0.6472983	0.9272669	0.3543242	4.399042	0.0002445	0.0011912	1.1836263	4.399042	5.48E-35	7.58E-34
7829	ENSMUSG00	13400	Dmpk	1	protein codi	MGI Symbol	19	dystrophia m	330	261	260	288	326	316	501	561	444	630	799	691	5.863314	4.5337328	4.6220937	4.734259	5.3363407	5.5403688	6.8087648	6.8227018	6.4624875	9.6081025	10.795879	9.9195012	0.0554128	2.7715528	0.6250004	0.9189759	0.4207566	2.7715528	0.0001146	0.0006197	1.0143984	2.7715528	1.37E-21	9.92E-21
7850	ENSMUSG00	234353	Psd3	1	protein codi	MGI Symbol	15	pleckstrin ar	806	753	967	967	1160.2	1088	1216.62	1716	1291	2048	2244.57	2353	14.3207	13.08008	17.190633	15.895932	18.99148	19.0757	16.53429	20.869441	18.790701	31.233959	30.328042	33.777983	0.2751527	4.3865467	0.0048252	0.0729934	0.3344895	4.3865467	0.000557	0.0024205	1.0963851	4.3865467	3.59E-30	4.10E-29
7880	ENSMUSG00	17364	Trpm1	1	protein codi	MGI Symbol	23	transient rec	426	337	343	416	438	411	485	532	407	922	1273	1065	7.5690054	5.8539002	6.0976082	6.8383741	7.1696847	7.205986	6.5913192	6.4700131	5.9239469	14.061382	17.200442	15.288377	0.1201686	3.1578883	0.2686768	0.7390421	-0.038865	3.1578883	0.7192672	0.7812758	1.2546949	3.1578883	1.17E-34	1.59E-33
7888	ENSMUSG00	29875	Iqgap1	1	protein codi	MGI Symbol	8	IQ motif con	1389	1085	1458	1394	1606	1460	2396	2634.95	2354	3647.97	4133.97	4247	24.679222	18.847127	25.919279	22.915129	26.288844	25.597906	32.562476	32.045415	34.262828	55.635031	55.857119	60.966891	0.1073436	5.1180538	0.2602939	0.7337405	0.5096791	5.1180538	6.19E-08	6.87E-07	1.3124692	5.1180538	4.89E-44	9.19E-43
7889	ENSMUSG00	23991	Cib1	1	protein codi	MGI Symbol	10	calcium and	233	229	257	248	229	233	483	457	403	589	768	703	4.139855	3.9778728	4.5687618	4.076723	3.7485338	4.0851453	6.5641385	5.5578872	5.8657263	8.9828133	10.377015	10.091765	-0.091172	2.605658	0.4502997	0.8529148	0.5029454	2.605658	6.75E-06	5.02E-05	1.2157846	2.605658	8.24E-29	8.78E-28
7904	ENSMUSG00	13032	Ctsc	1	protein codi	MGI Symbol	5	cathepsin C	275	167	244	268	276	256	202	159	135	1461	4066	3256	4.886095	2.9008942	4.3376571	4.405491	4.5178835	4.4884	2.7452505	1.9337069	1.9649455	22.281647	54.938727	46.740805	0.1467707	3.7197275	0.2179974	0.6953662	-0.867521	3.7197275	2.36E-12	4.37E-11	3.3558735	3.7197275	4.56E-209	1.03E-206
7966	ENSMUSG00	78388	Mvp	1	protein codi	MGI Symbol	3	major vault	243	208	216.36	241.4	295.67	226	300.77	306.05	257	693.92	875.22	858.26	4.3175312	3.6130897	3.846293	3.9682296	4.8398646	3.9624156	4.0875692	3.7220818	3.7406741	10.582944	11.825743	12.320566	0.1181504	2.5792246	0.3277776	0.7793548	-0.027943	2.5792246	0.8188313	0.8634584	1.5606436	2.5792246	2.95E-46	5.82E-45
7974	ENSMUSG00	11674	Aldoa	1	protein codi	MGI Symbol	12	aldolase A, f	9820	9720	9515	10775	10729	11310	20362	21492	16274	30583	44967	40113	174.47801	168.84246	169.15085	177.12375	175.62454	198.29611	276.72668	261.3788	236.87055	466.42	607.58232	575.83351	0.104658	8.1839936	0.2467949	0.7227346	0.5967081	8.1839936	4.25E-11	6.80E-10	1.6868037	8.1839936	1.80E-74	6.42E-73
798	ENSMUSG00	13014	Cstb	1	protein codi	MGI Symbol	1	cystatin B [S	309	293	282	290	330	301	483	520	440	719	960	881	5.490194	5.0895928	5.0131939	4.7671358	5.4018173	5.2773766	6.5641385	6.3240729	6.4042669	10.965438	12.971268	12.647005	-0.013791	2.8723496	0.9094367	0.9905825	0.3069272	2.8723496	0.0048868	0.014431	1.2308831	2.8723496	8.26E-32	1.01E-30
8002	ENSMUSG00	71960	Myh14	1	protein codi	MGI Symbol	9	myosin, hea	291	286	287	317	333	334	528	624	602	1077.08	1120	1013	5.1703769	4.9679984	5.1020803	5.2109726	5.4509247	5.8559594	7.1757042	7.5888875	8.7622016	16.4265	15.133147	14.541903	0.1154742	3.0946834	0.3080523	0.7675052	0.6250069	3.0946834	6.03E-09	7.64E-08	1.596278	3.0946834	3.40E-53	8.01E-52
802	ENSMUSG00	12505	Cd44	1	protein codi	MGI Symbol	9	CD44 antige	2048	1758	446	1786	1987	1795	4292	4687	3886	7324	11428	10391	36.388082	30.537557	7.9286684	29.358981	32.525488	31.471399	58.329778	57.001788	56.561321	111.698	154.41214	149.16576	0.3183245	5.9799318	0.0007382	0.0154468	1.1990138	5.9799318	2.26E-37	1.56E-35	2.471695	5.9799318	2.11E-145	2.42E-143
8031	ENSMUSG00	12862	Cox6a2	1	protein codi	MGI Symbol	1	cytochrome	103	80	88	63	58	69	1758	1784	1396	4116	5931	6976	1.8300647	1.3896499	1.5644009	1.0356192	0.9494103	1.2097641	23.891833	21.696435	20.318992	62.772936	80.138118	100.14246	-0.583875	4.7362322	0.0003558	0.0084969	3.7836189	4.7362322	4.43E-213	4.68E-210	5.6662316	4.7362322	0	0
805	ENSMUSG00	22388	Wdr1	1	protein codi	MGI Symbol	11	WD repeat d	5061	4765	4806	5867	5932	5615	10592	11559	9464	13487	15696.18	15464	89.921916	82.771022	85.437624	96.444089	97.101758	98.446742	143.94898	140.57684	137.74996	205.68965	212.08267	221.99011	0.1778301	7.0710092	0.0509371	0.3600463	0.7100971	7.0710092	6.58E-15	1.49E-13	1.30944	7.0710092	5.12E-46	1.00E-44
8083	ENSMUSG00	74105	Gga2	1	protein codi	MGI Symbol	6	golgi associa	500.96	437	546.81	448.93	482	468.16	814.24	866.21	691.48	1207.31	1391.46	1321.45	8.900866	7.5909626	9.7207963	7.3796906	7.889927	8.2081615	11.065806	10.534568	10.064597	18.412632	18.801043	18.96979	-0.158956	3.5289319	0.1305331	0.556332	0.2732142	3.5289319	0.0073253	0.0199972	1.1001574	3.5289319	2.55E-28	2.67E-27
8109	ENSMUSG00	77590	Chst15	1	protein codi	MGI Symbol	4	carbohydrat	387.14	394.93	521.14	493.56	413.72	440.93	529.71	668.91	502.97	922.56	1231.98	1104.92	6.8785557	6.8601804	9.2644534	8.1133364	6.7722419	7.730743	7.1989437	8.1350685	7.3208049	14.069922	16.646191	15.86144	-0.024399	3.2685486	0.8252766	0.975947	-0.02048	3.2685486	0.8459519	0.8849843	1.0186211	3.2685486	6.97E-24	5.69E-23
8137	ENSMUSG00	17345	Mki67	1	protein codi	MGI Symbol	2	antigen iden	755	591	723	641	714	686	1044	1251	1236	1656	1719	2031	13.414552	10.266039	12.852976	10.537014	11.687568	12.027509	14.188324	15.21426	17.990168	25.255584	23.226678	29.155582	-0.092998	4.035628	0.357198	0.8002987	0.3748836	4.035628	0.0001335	0.0007071	1.0875654	4.035628	4.77E-29	5.14E-28
8138	ENSMUSG00	70495	Atp6ap2	1	protein codi	MGI Symbol	2	ATPase, H+ t	833	824	922	867	977	862	1596	1754	1529	2384	2814	2721	14.800426	14.313394	16.390655	14.252092	15.992653	15.113284	21.690197	21.331584	22.254828	36.35828	38.022031	39.060728	-0.004523	4.4960438	0.9670829	1	0.5204358	4.4960438	6.56E-08	7.26E-07	1.317936	4.4960438	6.00E-43	1.08E-41
8146	ENSMUSG00	76954	Denn2b	1	protein codi	MGI Symbol	11	DENN domai	356	324	315	376	373	349	743	711	595	926	1013	945	6.3252721	5.6280821	5.5998443	6.1808382	6.1056904	6.1189516	10.097629	8.6469536	8.6603155	14.122386	13.687391	13.565743	0.0685381	3.1404342	0.540541	0.888141	0.6424628	3.1404342	1.06E-09	1.45E-08	1.2367591	3.1404342	5.50E-33	6.98E-32
8168	ENSMUSG00	72584	Cul4b	1	protein codi	MGI Symbol	4	cullin 4B [So	491	441	444	511	512	486	885	963	856	1122	1319	1298	8.7239005	7.6604451	7.8931138	8.4000221	8.3810013	8.5209469	12.027459	11.711697	12.45921	17.111573	17.821982	18.633159	0.059688	3.5485268	0.5745923	0.9024016	0.5759274	3.5485268	1.39E-08	1.70E-07	1.1418283	3.5485268	4.83E-30	5.49E-29
8173	ENSMUSG00	56501	Elf4	1	protein codi	MGI Symbol	3	E74-like fact	144.01	110	98	156	128	157	281	312	237	413	443.71	422	2.5587147	1.9107686	1.7421738	2.5643903	2.0952503	2.7526516	3.818888	3.7944438	3.449571	6.298645	5.9952932	6.0579298	0.2530991	1.8752718	0.0646202	0.399813	0.8339936	1.8752718	6.09E-11	9.53E-10	1.5620231	1.8752718	4.32E-37	6.52E-36
8179	ENSMUSG00	70415	Stk26	1	protein codi	MGI Symbol	4	serine/threo	563	509	572	641	684	634	2143	2067	1800	3027	3275	3261	10.003169	8.8416475	10.168606	10.537014	11.196494	11.115803	29.124118	25.13819	26.199274	46.164645	44.250942	46.812581	0.1792418	4.5516702	0.0795602	0.4480404	1.4714304	4.5516702	1.28E-50	1.27E-48	2.2417162	4.5516702	2.35E-114	1.69E-112
8181	ENSMUSG00	14735	Gpc4	1	protein codi	MGI Symbol	1	glypican 4 [S	484	359	438	372	427	414	640	694	653	1058	1125	1142	8.5995272	6.2360539	7.7864501	6.1150846	6.9896242	7.2585844	8.6978233	8.440205	9.5045143	16.135512	15.200705	16.393734	-0.151798	3.3006565	0.1577203	0.607108	0.2354624	3.3006565	0.0231315	0.0504475	1.0772822	3.3006565	1.63E-26	1.57E-25
8196	ENSMUSG00	18824	Plp2	1	protein codi	MGI Symbol	3	proteolipid p	585.52	599.91	544.93	798.66	755.36	706.82	1810.98	1768.55	1578.76	3017.27	4119.65	4156.58	10.403296	10.420811	9.687375	13.128692	12.364596	12.392543	24.61185	21.508537	22.979092	46.016253	55.663631	59.668886	0.3122836	4.6470785	0.001978	0.0359287	1.1788023	4.6470785	3.35E-33	1.85E-31	2.402701	4.6470785	3.72E-131	3.54E-129
8203	ENSMUSG00	18715	Pim2	1	protein codi	MGI Symbol	3	proviral inte	128	115	79	135	139	130	236	325	218	345	447	398	2.2742551	1.9976217	1.4044054	2.2191839	2.2753109	2.2792656	3.2073223	3.9525456	3.1730232	5.2615799	6.0397469	5.713403	0.2541748	1.7667809	0.0721278	0.4245441	0.867339	1.7667809	2.66E-11	4.43E-10	1.5835621	1.7667809	8.64E-37	1.29E-35
8214	ENSMUSG00	13595	Ebp	1	protein codi	MGI Symbol	4	phenylalkyla	191	168	182	182	183	205	267	268	243	406	559	509	3.3936151	2.9182648	3.2354656	2.9917887	2.9955532	3.5942266	3.6286232	3.2593299	3.536902	6.1918883	7.5530615	7.3068395	0.003691	2.0974692	0.9833807	1	0.1256739	2.0974692	0.3010049	0.392256	1.1422032	2.0974692	2.44E-23	1.94E-22
8215	ENSMUSG00	53627	Porcn	1	protein codi	MGI Symbol	7	porcupine O	401	301	297	368	374	375	408	429	326	733	1161	1114	7.1248149	5.2285577	5.2798532	6.049331	6.1220596	6.5748047	5.5448624	5.2173602	4.7449796	11.178951	15.687128	15.991786	0.0881425	2.9933037	0.4272502	0.8463046	-0.18389	2.9933037	0.0932058	0.153485	1.2827481	2.9933037	1.89E-35	2.65E-34
8233	ENSMUSG00	17698	Msn	1	protein codi	MGI Symbol	3	moesin [Sou	823	724	876	1009	1000	990	2287	2289	2199	5506	6888.13	6883	14.62275	12.576332	15.5729	16.586345	16.369143	17.357484	31.081128	27.838083	32.006779	83.971765	93.070608	98.80742	0.234293	5.2661012	0.016929	0.1811511	1.0877187	5.2661012	1.17E-29	5.69E-28	2.6892829	5.2661012	1.38E-165	2.12E-163
8254	ENSMUSG00	56726	Sh3bgrl	1	protein codi	MGI Symbol	3	SH3-binding	231	230	260	243	261	225	360	378	343	633	724	737	4.1043198	3.9952435	4.6220937	3.9945311	4.2723464	3.9448828	4.8925256	4.5971146	4.9924172	9.6538553	9.7824983	10.579844	-0.058272	2.5502025	0.6228536	0.9180952	0.1859873	2.5502025	0.1026354	0.1658363	1.2384202	2.5502025	6.25E-30	7.03E-29
8263	ENSMUSG00	11605	Gla	1	protein codi	MGI Symbol	3	galactosidas	162	185	199	203	224	226	343	400	279	530	543	555	2.8783542	3.2135654	3.5376794	3.3369951	3.6666881	3.9624156	4.6614897	4.8646715	4.0608874	8.0830068	7.3368737	7.9671826	0.1862413	2.2843684	0.1294509	0.5549566	0.49893	2.2843684	2.42E-05	0.0001581	1.2789765	2.2843684	2.54E-29	2.79E-28
8269	ENSMUSG00	83453	Chrdl1	1	protein codi	MGI Symbol	5	chordin-like	309	300	321	339	377	367	1717	1810	1396	2884	3599	3207	5.490194	5.2111871	5.706508	5.5726174	6.171167	6.4345422	23.334629	22.012638	20.318992	43.983758	48.628745	46.037396	0.1473502	4.3290138	0.1854499	0.6505304	2.0008376	4.3290138	3.85E-85	7.78E-83	3.0788618	4.3290138	3.97E-198	8.37E-196
8285	ENSMUSG00	110651	Rps6ka3	1	protein codi	MGI Symbol	9	ribosomal pr	305	256	298	341	364	345	513	577	504	740	900	911	5.4191236	4.4468797	5.2976304	5.6054942	5.9583681	6.0488203	6.971849	7.0172886	7.3357967	11.285708	12.160564	13.077664	0.2161079	2.9319941	0.0549798	0.373611	0.4918344	2.9319941	5.69E-06	4.31E-05	1.2687542	2.9319941	1.52E-33	1.98E-32
8295	ENSMUSG00	192176	Flna	1	protein codi	MGI Symbol	16	filamin, alph	4194	3404	4041	3939	4275	4163	5652	6593	5967	10229	11606	12502	74.517391	59.129603	71.838002	64.750855	69.978087	72.989099	76.812652	80.181948	86.850593	156.00203	156.81723	179.46976	0.0155892	6.5829751	0.8657323	0.9824939	0.246905	6.5829751	0.0068317	0.0189436	1.2605153	6.5829751	1.21E-42	2.15E-41
8313	ENSMUSG00	55936	Ctps2	1	protein codi	MGI Symbol	13	cytidine 5pri	377	361	391	419	410	408	632	730	635	889	1015	987	6.6983921	6.2707952	6.9509178	6.8876893	6.7113487	7.1533875	8.5891005	8.8780255	9.2425216	13.5581	13.714414	14.168665	0.0588982	3.1930609	0.5911028	0.9073765	0.422927	3.1930609	5.22E-05	0.000311	1.0568631	3.1930609	6.14E-25	5.34E-24
8317	ENSMUSG00	108012	Ap1s2	1	protein codi	MGI Symbol	4	adaptor-rela	714	710	782	767	803	705	966	1017	864	1736	2097	2117	12.686079	12.333143	13.901836	12.608252	13.144422	12.360633	13.128277	12.368427	12.575651	26.47566	28.334114	30.390136	-0.029976	4.067252	0.7668507	0.9609679	-0.031853	4.067252	0.7508491	0.8079249	1.1304444	4.067252	1.65E-31	1.99E-30
8323	ENSMUSG00	12111	Bgn	1	protein codi	MGI Symbol	8	biglycan [Sou	363	315	332	445	544	488	422	520	498	826	1061	875	6.4496454	5.4717465	5.9020581	7.3150877	8.9048139	8.5560125	5.7351272	6.3240729	7.2484657	12.59729	14.335954	12.560873	0.4750546	3.0885214	9.48E-06	0.0003362	0.1142822	3.0885214	0.2900925	0.3806462	1.1484936	3.0885214	1.30E-28	1.38E-27
8335	ENSMUSG00	19703	Renbp	1	protein codi	MGI Symbol	10	renin binding	176	151	146	171	177	172	257	268	247	577	750	688	3.1271008	2.6229642	2.5954834	2.8109663	2.8973383	3.0156438	3.4927197	3.2593299	3.5951226	8.7998018	10.133803	9.8764354	0.063665	2.254072	0.6335982	0.9226029	0.3085443	2.254072	0.0121532	0.0301214	1.7879134	2.254072	1.30E-54	3.20E-53
8358	ENSMUSG00	14605	Tsc22d3	1	protein codi	MGI Symbol	6	TSC22 doma	306	267	276	323	296	332	444	410	338	776	982	724	5.4368912	4.6379565	4.9065302	5.309603	4.8452664	5.8208938	6.0341149	4.9862883	4.9196414	11.834742	13.268527	10.393226	0.091882	2.7937525	0.4165406	0.8394313	0.089518	2.7937525	0.4259241	0.5178608	1.2450674	2.7937525	3.16E-32	3.88E-31
8361	ENSMUSG00	75746	Morc4	1	protein codi	MGI Symbol	10	microrchidia	219	233	215	261	280	257	411	526	489	744	740	735	3.8911084	4.0473553	3.8221159	4.2904222	4.5833601	4.5059328	5.5856334	6.397043	7.1174694	11.346711	9.9986861	10.551134	0.1857314	2.6850409	0.1169176	0.5328909	0.6982036	2.6850409	4.91E-10	7.06E-09	1.4381242	2.6850409	2.67E-39	4.35E-38
8378	ENSMUSG00	11601	Angpt2	1	protein codi	MGI Symbol	2	angiopoietin	341	310	430	515	424	449	878	985	1007	2108	1237	1574	6.0587578	5.3848934	7.6442318	8.4657757	6.9405167	7.8722328	11.932326	11.979254	14.657038	32.149016	16.71402	22.595217	0.2866315	3.6786066	0.0076251	0.1033364	1.0138383	3.6786066	6.23E-23	2.36E-21	1.9028844	3.6786066	7.99E-79	3.03E-77
8392	ENSMUSG00	13685	Eif4ebp1	1	protein codi	MGI Symbol	2	eukaryotic tr	706	597	511	807	701	660	825	913	758	1500	1802	1963	12.543938	10.370262	9.0841918	13.265788	11.474769	11.571656	11.212038	11.103613	11.032805	22.876434	24.348152	28.179422	0.1827245	3.8901732	0.070773	0.4210041	0.0597023	3.8901732	0.55541	0.6385568	1.2367072	3.8901732	1.53E-36	2.26E-35
8419	ENSMUSG00	319520	Dusp4	1	protein codi	MGI Symbol	1	dual specific	348	366	326	357	370	373	1042	855	1009	1707	2186	2107	6.1831311	6.3576483	5.7953944	5.8685086	6.056583	6.5397391	14.161144	10.398235	14.686148	26.033382	29.536659	30.246583	0.0093005	3.7682767	0.9391772	0.9967924	1.0952105	3.7682767	2.03E-26	8.79E-25	2.2263362	3.7682767	5.83E-107	3.65E-105
8441	ENSMUSG00	20563	Slit2	1	protein codi	MGI Symbol	23	slit guidance	1010	784	892	1056	1139	1037	2663	2990	2399	4180	4920	5200	17.945294	13.618569	15.857337	17.35895	18.644454	18.181527	36.191099	36.363419	34.91781	63.748997	66.477751	74.647477	0.1925581	5.1146061	0.0482357	0.3514107	1.1806072	5.1146061	5.30E-35	3.23E-33	2.1112821	5.1146061	4.84E-106	2.99E-104
8446	ENSMUSG00	14182	Fgfr1	1	protein codi	MGI Symbol	19	fibroblast gr	2778.95	2590.99	2315.96	3124	2733	2948.99	4931.88	5303	4172	6396.7	7515.96	6808	49.375323	45.007112	41.171477	51.35356	44.736868	51.704089	67.02597	64.493382	60.724095	97.555792	101.55368	97.730773	0.1246251	6.0104318	0.1777527	0.6369864	0.5041256	6.0104318	4.35E-08	4.91E-07	1.1308154	6.0104318	2.26E-34	3.04E-33
8459	ENSMUSG00	11886	Asah1	1	protein codi	MGI Symbol	5	N-acylsphing	494	462	401	717	762	626	1119	1138	928	1564	1973	2266	8.7772034	8.0252282	7.1286906	11.786332	12.473287	10.975541	15.2076	13.83999	13.507181	23.852496	26.658659	32.529073	0.5584068	3.9547174	5.48E-08	2.91E-06	0.8303087	3.9547174	1.89E-16	4.88E-15	1.7948955	3.9547174	1.30E-72	4.53E-71
846	ENSMUSG00	71722	Cic	1	protein codi	MGI Symbol	15	capicua tran	3460	3145	3224	3550	3633	3583	5374	6169	5221	9028	10083	9960	61.475959	54.630612	57.313961	58.356318	59.469097	62.820067	73.034535	75.025396	75.992449	137.68563	136.23885	142.97863	0.0588506	6.3752667	0.5224327	0.8815586	0.3695692	6.3752667	5.43E-05	0.0003224	1.2654334	6.3752667	8.92E-43	1.60E-41
8470	ENSMUSG00	60406	Sap30	1	protein codi	MGI Symbol	1	sin3 associa	192	179	172	181	162	160	322	371	294	507	683	615	3.4113827	3.1093416	3.0576927	2.9753503	2.6518012	2.80525	4.3760924	4.5119828	4.2792147	7.7322348	9.228517	8.8284996	-0.183606	2.2733624	0.149978	0.5924289	0.4597973	2.2733624	0.0001081	0.0005874	1.42962	2.2733624	1.99E-36	2.91E-35
8481	ENSMUSG00	12367	Casp3	1	protein codi	MGI Symbol	4	caspase 3 [S	148	154	184	125	168	170	252	316	248	458	547	539	2.6296075	2.6750761	3.2710201	2.0547999	2.7500161	2.9805781	3.4247679	3.8430905	3.6096777	6.984938	7.3909207	7.7374981	-0.141361	2.0666185	0.2914247	0.7545748	0.3444932	2.0666185	0.0049675	0.0146285	1.366769	2.0666185	4.17E-32	5.10E-31
8507	ENSMUSG00	13666	Eif2ak3	1	protein codi	MGI Symbol	5	eukaryotic tr	214.92	208.99	214.95	238	191.98	193.92	353	375.97	315	461.98	585.94	548.9	3.8186165	3.6302867	3.8212271	3.9123391	3.1425481	3.399963	4.7973932	4.5724264	4.5848729	7.0456368	7.9170677	7.8796154	-0.107849	2.3065461	0.384688	0.8203223	0.3081334	2.3065461	0.0079084	0.0212052	1.0198086	2.3065461	8.06E-20	5.19E-19
8546	ENSMUSG00	14681	Gnao1	1	protein codi	MGI Symbol	12	guanine nuc	221	214	205	250	219	233	356	405	321	553	611	579	3.9266436	3.7173135	3.6443431	4.1095998	3.5848424	4.0851453	4.8381642	4.9254799	4.6722038	8.4337788	8.2556719	8.3117094	0.0608739	2.4002948	0.613588	0.9148505	0.3554433	2.4002948	0.0020423	0.0070169	1.1466631	2.4002948	6.93E-25	6.02E-24
8561	ENSMUSG00	64209	Herpud1	1	protein codi	MGI Symbol	6	homocysteir	1663	1413	1594	1752	1747	1804	3011	3265	3149	4714	5556	5441	29.547549	24.544691	28.33699	28.800076	28.596893	31.629194	40.920541	39.707881	45.834174	71.893008	75.071216	78.1071	0.1109913	5.4492178	0.2395191	0.7180282	0.6172518	5.4492178	3.93E-11	6.32E-10	1.4492455	5.4492178	1.11E-53	2.65E-52
8581	ENSMUSG00	326618	Tpm4	1	protein codi	MGI Symbol	4	tropomyosir	824.89	891.38	873.54	988.06	1068.35	1032.68	1286.26	1318.13	1044.19	2475.88	3370.41	3232.33	14.656331	15.483827	15.529168	16.242125	17.487974	18.105785	17.480722	16.030674	15.198344	37.759538	45.540097	46.401015	0.1824596	4.5286984	0.0617965	0.3941672	0.0931346	4.5286984	0.3397583	0.4320868	1.5060246	4.5286984	1.71E-55	4.31E-54
8589	ENSMUSG00	73711	Mvb12a	1	protein codi	MGI Symbol	7	multivesicul	196	178	217	239	215	219	401	436	321	598	743	663	3.4824532	3.091971	3.8576705	3.9287775	3.5193658	3.8396859	5.4497299	5.3024919	4.6722038	9.1200719	10.039221	9.5175533	0.1139138	2.4781361	0.3643524	0.8055715	0.5659702	2.4781361	1.02E-06	9.09E-06	1.4594881	2.4781361	5.54E-39	8.91E-38
8597	ENSMUSG00	234797	6430548M0	1	protein codi	MGI Symbol	9	RIKEN cDNA	221	177	179	215	162	203	120	142	110	252	310	335	3.9266436	3.0746004	3.1821337	3.5342559	2.6518012	3.5591609	1.6308419	1.7269584	1.6010667	3.843241	4.1886388	4.8090201	-0.064508	1.6578297	0.6135708	0.9148505	-1.034809	1.6578297	3.41E-15	7.93E-14	0.3357044	1.6578297	0.0048113	0.0062322
8611	ENSMUSG00	12554	Cdh13	1	protein codi	MGI Symbol	8	cadherin 13	6	4	9	3	2	6	470	496	363	1596	1890	1622	0.1066057	0.0694825	0.1599956	0.0493152	0.0327383	0.1051969	6.387464	6.0321926	5.2835202	24.340526	25.537185	23.284271	-0.839346	2.9664964	0.2069448	0.6796504	5.700596	2.9664964	7.99E-167	4.47E-164	7.7451714	2.9664964	0	0
8663	ENSMUSG00	270118	Maml2	1	protein codi	MGI Symbol	3	mastermind	389	352	516	485	553	469	715	799	658	986	1118	1162	6.9116035	6.1144596	9.1730782	7.9726237	9.0521362	8.2228891	9.7170995	9.7171813	9.5772901	15.037443	15.106123	16.68084	0.1863852	3.3712666	0.078551	0.446216	0.386718	3.3712666	0.0001826	0.0009206	1.0771462	3.3712666	2.06E-26	1.97E-25
8756	ENSMUSG00	66952	2310030G0	1	protein codi	MGI Symbol	3	RIKEN cDNA	358	287	284	312	333	356	747	717	541	620	917	955	6.3608073	4.985369	5.0487485	5.1287806	5.4509247	6.2416813	10.151991	8.7199236	7.8743373	9.4555929	12.390264	13.709296	0.0361467	3.009522	0.7520712	0.958233	0.7068086	3.009522	2.67E-11	4.43E-10	1.118125	3.009522	1.22E-26	1.18E-25
8770	ENSMUSG00	23821	Bace1	1	protein codi	MGI Symbol	6	beta-site API	1713.16	1676.29	1618.76	1909.54	1874.23	1818.9	3253.02	3575.49	3034.32	4446.13	5497.82	4606	30.438773	29.118203	28.777155	31.389781	30.679539	31.890433	44.209677	43.483961	44.164989	67.807734	74.285103	66.120438	0.0890245	5.4473482	0.3443406	0.7903445	0.5778932	5.4473482	5.66E-10	8.08E-09	1.2370667	5.4473482	6.20E-40	1.02E-38
878	ENSMUSG00	29865	Cabp5	1	protein codi	MGI Symbol	3	calcium binc	353	202	282	423	430	386	1182	864	1132	4543	3772	4742	6.2719692	3.508866	5.0131939	6.9534429	7.0387316	6.7676656	16.063792	10.50769	16.476432	69.285094	50.966276	68.072757	0.4900113	4.4859408	9.99E-06	0.0003515	1.5391217	4.4859408	2.69E-49	2.61E-47	3.6704761	4.4859408	2.50E-272	1.08E-269
8799	ENSMUSG00	15288	Hmbs	1	protein codi	MGI Symbol	9	hydroxymetl	707.8	686.66	736.77	800.84	755.92	791.95	1506	1681.8	1288.04	1796.03	2249.88	1928.57	12.57592	11.927712	13.097769	13.164528	12.373763	13.885111	20.467065	20.453511	18.747618	27.391175	30.399789	27.68517	0.0680183	4.2184332	0.4973287	0.8711141	0.666558	4.2184332	7.77E-12	1.38E-10	1.1849575	4.2184332	2.46E-34	3.29E-33
8800	ENSMUSG00	71732	Vps11	1	protein codi	MGI Symbol	6	VPS11, COR	1073	906	994	1109	1131	1101	2003	2301	1819	3190	3472	3418	19.064654	15.737785	17.67062	18.230185	18.513501	19.303627	27.221469	27.984023	26.475822	48.650551	46.912754	49.066361	0.0950765	4.8079139	0.3278271	0.7793548	0.6387217	4.8079139	2.14E-11	3.61E-10	1.4627218	4.8079139	4.47E-53	1.05E-51
8803	ENSMUSG00	84004	Mcam	1	protein codi	MGI Symbol	6	melanoma c	66	82	87	73	98	61	369	389	363	870	955	1144	1.1726628	1.4243911	1.5466237	1.2000032	1.604176	1.0695016	5.0148388	4.730893	5.2835202	13.268332	12.90371	16.422445	-0.093271	2.4900074	0.5638587	0.8961357	1.8556334	2.4900074	9.70E-45	8.30E-43	3.359511	2.4900074	1.38E-158	1.79E-156
8807	ENSMUSG00	54721	Tyk2	1	protein codi	MGI Symbol	6	tyrosine kina	305.22	304	191.39	354	357	373.48	473.15	442	470.2	649.36	804	758.25	5.4230324	5.2806696	3.4023943	5.8191934	5.8437841	6.5481548	6.4302736	5.375462	6.8438325	9.903361	10.863437	10.884894	0.3669829	2.7971825	0.0010825	0.0215151	0.3992723	2.7971825	0.0002737	0.0013134	1.1655817	2.7971825	2.18E-27	2.18E-26
8821	ENSMUSG00	235041	Kank2	1	protein codi	MGI Symbol	3	KN motif anc	495	452	450	507	517	527	874	956	691	1167	1483	1378	8.794971	7.8515219	7.9997775	8.3342685	8.462847	9.2397922	11.877965	11.626565	10.05761	17.797866	20.037907	19.781581	0.0787794	3.57419	0.4541483	0.8556939	0.4464443	3.57419	1.18E-05	8.29E-05	1.2254547	3.57419	1.56E-34	2.11E-33
8832	ENSMUSG00	71602	Myo1e	1	protein codi	MGI Symbol	4	myosin IE [S	280	234	291	180	161	176	436	495	486	760	822	690	4.9749331	4.064726	5.1731895	2.9589119	2.6354321	3.085775	5.9253921	6.020031	7.0738039	11.590727	11.106649	9.9051459	-0.711501	2.6550292	4.49E-09	2.77E-07	0.4189754	2.6550292	0.0001367	0.0007203	1.1978115	2.6550292	1.82E-29	2.01E-28
8837	ENSMUSG00	12306	Anxa2	1	protein codi	MGI Symbol	6	annexin A2 [	1855	1976	1886	2855	2870	2931	8727	8329	6812	11597	16299	14990	32.958932	34.324352	33.527956	46.93163	46.979441	51.388674	118.60298	101.29462	99.149696	176.86534	220.22782	215.18571	0.5272166	6.6191202	1.47E-08	8.42E-07	1.6620364	6.6191202	3.16E-70	4.84E-68	2.602517	6.6191202	5.06E-161	7.28E-159

Table S2. Differentially expressed genes (> 2-fold) in *Trpm3*-mutant lenses summarized in Fig. 9A.

8848	ENSMUSG00	93683	Glce	1	protein codi	MGI Symbol	5	glucuronyl C	389	312	429	397	415	453	577	570	592	839	1035	1156	6.9116035	5.4196346	7.6264546	6.5260446	6.7931944	7.9423641	7.8416313	6.9321569	8.61665	12.795552	13.984649	16.594708	0.0908046	3.1805941	0.4023691	0.831347	0.2274115	3.1805941	0.0306977	0.0629322	1.120693	3.1805941	6.33E-28	6.53E-27
8880	ENSMUSG00	74211	1700017B05	1	protein codi	MGI Symbol	6	RIKEN cDNA	136	128	157	122	142	151	277	362	255	424	550	454	2.4163961	2.2234398	2.7910335	2.0054847	2.3244183	2.6474547	3.7645267	4.4025277	3.7115638	6.4664055	7.4314559	6.5172989	-0.092514	1.9927456	0.5037811	0.873469	0.679927	1.9927456	3.91E-08	4.48E-07	1.4590357	1.9927456	1.11E-34	1.51E-33
8915	ENSMUSG00	68801	Elovl5	1	protein codi	MGI Symbol	4	ELOVL famil	209	178	189	209	198	198	295	333	284	451	591	573	3.7134322	3.091971	3.3599066	3.4356255	3.2410904	3.4714969	4.0091529	4.049839	4.1336632	6.8781813	7.9854371	8.2255777	-0.002393	2.233451	1	1	0.2623977	2.233451	0.0267806	0.0564712	1.1845698	2.233451	1.42E-25	1.28E-24
8918	ENSMUSG00	66111	Tmed3	1	protein codi	MGI Symbol	2	transmembr	1537	1574	1641	1706	1613	1600	3282	3598	2769	3763	5102	5045	27.308829	27.341362	29.172522	28.043909	26.403428	28.0525	44.603525	43.75772	40.303216	57.389349	68.936887	72.422408	-0.022992	5.366528	0.8099118	0.9718533	0.6183503	5.366528	3.57E-11	5.81E-10	1.245683	5.366528	2.41E-40	4.01E-39
8926	ENSMUSG00	22003	Tpm1	1	protein codi	MGI Symbol	21	tropomyosir	957	870	882	1346	1442	1337	2471	2530	1890	4077	4408	4825	17.003611	15.112443	15.679564	22.126086	23.604305	23.44137	33.581752	30.769047	27.509237	62.178149	59.559741	69.264245	0.5332807	5.0636821	3.33E-08	1.81E-06	0.9428288	5.0636821	6.45E-23	2.43E-21	1.9985509	5.0636821	1.22E-95	6.09E-94
8932	ENSMUSG00	26432	Plod2	1	protein codi	MGI Symbol	5	procollagen	553.23	541.66	578.38	611.4	740.67	671.34	1203.07	1285.96	998.69	1451.65	1965.46	1910.74	9.8295794	9.4089721	10.282025	10.050437	12.124133	11.770478	16.350141	15.639432	14.536085	22.139051	26.55678	27.429215	0.2013272	3.9645126	0.0480196	0.3503737	0.6566545	3.9645126	3.88E-11	6.25E-10	1.367045	3.9645126	7.14E-44	1.33E-42
8964	ENSMUSG00	17436	Me1	1	protein codi	MGI Symbol	4	malic enzym	2168.15	2095.91	2448.16	2454.01	2603.86	2340.14	4877.23	5589.94	4710.59	6778.41	8244.42	7644.59	38.522861	36.407264	43.521634	40.339996	42.622957	41.029236	66.283258	67.983054	68.563354	103.37723	111.39644	109.74026	0.0660277	6.0060825	0.4785403	0.8649415	0.7759959	6.0060825	4.66E-17	1.24E-15	1.4540461	6.0060825	6.97E-55	1.73E-53
8970	ENSMUSG00	56693	Crtap	1	protein codi	MGI Symbol	2	cartilage ass	636	616	685	681	671	660	1373	1278	1092	1777	2103	1986	11.300205	10.700304	12.177439	11.19455	10.983695	11.571656	18.659549	15.542625	15.894226	27.100949	28.415185	28.509594	-0.018229	4.0820339	0.8621417	0.98209	0.5514716	4.0820339	2.04E-08	2.42E-07	1.2978722	4.0820339	1.95E-40	3.26E-39
8985	ENSMUSG00	74769	Pik3cb	1	protein codi	MGI Symbol	3	phosphatidy	169.96	141.88	126.73	189.23	171.74	156.52	299.14	344.17	244.75	366.73	460.88	394.9	3.0197844	2.4645441	2.2529151	3.1106383	2.8112367	2.7442358	4.065417	4.185685	3.5623735	5.5929832	6.2272898	5.6689016	0.1644279	1.9575875	0.2153998	0.6929555	0.6128239	1.9575875	6.82E-07	6.33E-06	1.1771399	1.9575875	5.76E-23	4.50E-22
900	ENSMUSG00	22785	Slc30a4	1	protein codi	MGI Symbol	2	solute carrie	1025.99	1013	1044	1001.99	1131.99	1047	1381	1504	1269	1977	2813	3081	18.229399	17.596442	18.559484	16.471112	18.529706	18.356855	18.768272	18.291165	18.470488	30.151141	38.008519	44.22863	-0.027601	4.5264461	0.7793051	0.9647847	0.0300317	4.5264461	0.7575968	0.8138925	1.0475561	4.5264461	3.27E-28	3.40E-27
9003	ENSMUSG00	19228	Pth1r	1	protein codi	MGI Symbol	6	parathyroid	217	215	164	103	94	114	276	250	213	327	410	374	3.8555732	3.7346841	2.9154745	1.6931551	1.5386995	1.9987406	3.7509363	3.0404197	3.1002474	4.9870627	5.5398126	5.3688762	-1.008569	1.8147213	6.08E-14	6.28E-12	-0.088092	1.8147213	0.4779587	0.5667966	0.5978014	1.8147213	2.44E-07	4.97E-07
9008	ENSMUSG00	71268	Lrrfip2	1	protein codi	MGI Symbol	18	leucine rich	528	461	539	560	570	585	836	924	769	1066	1407	1324	9.3813024	8.0078575	9.5819557	9.2055037	9.3304116	10.256695	11.361532	11.237391	11.192912	16.257519	19.011015	19.006396	0.0940559	3.5922936	0.3649994	0.8062064	0.3255183	3.5922936	0.0013091	0.0049021	1.0095545	3.5922936	3.89E-24	3.21E-23
9011	ENSMUSG00	211770	Trib1	1	protein codi	MGI Symbol	2	tribbles pseu	1119	1036	859	1407	1270	1410	2159	2178	1721	2368	2790	2434	19.881965	17.995966	15.270686	23.128828	20.788812	24.721266	29.341563	26.488136	25.049417	36.114265	37.697749	34.940761	0.3686204	4.7024057	0.0001258	0.0033022	0.6057728	4.7024057	2.09E-10	3.09E-09	1.0329152	4.7024057	2.01E-27	2.01E-26
9014	ENSMUSG00	18571	Pdcd6ip	1	protein codi	MGI Symbol	6	programmec	3211	2961	3193	3395	3601	3318	5456	6031	5056	7396	8217	7993	57.051822	51.434417	56.762866	55.808366	58.945285	58.173872	74.148944	73.347084	73.590849	112.79607	111.02595	114.74178	0.0655489	6.2270326	0.476866	0.864464	0.4199905	6.2270326	4.60E-06	3.57E-05	1.0347873	6.2270326	2.58E-29	2.82E-28
9042	ENSMUSG00	22041	Trf	1	protein codi	MGI Symbol	9	transferrin [	190	123	161	216	235	193	160	183	167	403	908	685	3.3758475	2.1365867	2.8621426	3.5506943	3.8467487	3.3838328	2.1744558	2.2255872	2.4307104	6.1461354	12.268658	9.8333695	0.3663989	2.1995476	0.0037524	0.0600125	-0.294145	2.1995476	0.0235816	0.0512291	1.7586259	2.1995476	7.03E-53	1.64E-51
9043	ENSMUSG00	235559	Topbp1	1	protein codi	MGI Symbol	10	topoisomera	377	366	367	419	387	378	675	643	533	898	999	995	6.6983921	6.3576483	6.524263	6.8876893	6.3348584	6.6274031	9.1734855	7.8199594	7.7578961	13.695359	13.498226	14.283508	0.0197738	3.150584	0.8641012	0.9821164	0.3379519	3.150584	0.0013166	0.0049282	1.0827103	3.150584	4.84E-26	4.56E-25
9047	ENSMUSG00	235567	Dnajc13	1	protein codi	MGI Symbol	14	DnaJ heat sh	2991.6	2542.9	2801.7	3159.43	3290.5	3051.8	4031.82	4902.68	4469.26	6496	7747.65	7864.83	53.153607	44.171759	49.806615	51.935972	53.862666	53.506637	54.793841	59.624819	65.050759	99.070212	104.68422	112.90187	0.1147307	6.0652906	0.2139619	0.6905376	0.2865615	6.0652906	0.0018202	0.006409	1.105876	6.0652906	4.65E-33	5.92E-32
9058	ENSMUSG00	74840	Manf	1	protein codi	MGI Symbol	7	mesencepha	674.69	616.89	637.56	763.03	831.53	670.42	1140.54	1301.34	1068.11	1520.73	2055.75	2185.12	11.987634	10.715764	11.334085	12.542992	13.611434	11.754348	15.500337	15.826479	15.546504	23.192587	27.776755	31.368018	0.1559427	4.0751965	0.1216035	0.5403338	0.4618125	4.0751965	2.81E-06	2.29E-05	1.274815	4.0751965	4.87E-39	7.84E-38
9088	ENSMUSG00	330267	Thsd7a	1	protein codi	MGI Symbol	6	thrombospo	210	154	172	159	184	176	271	312	253	456	519	556	3.7311998	2.6750761	3.0576927	2.6137055	3.0119224	3.085775	3.6829846	3.7944438	3.6824535	6.9544361	7.012592	7.9815379	-0.11958	2.1186537	0.3515445	0.7957545	0.2390154	2.1186537	0.0475803	0.0893836	1.2139718	2.1186537	3.04E-26	2.88E-25
9094	ENSMUSG00	19309	Pygm	1	protein codi	MGI Symbol	4	muscle glycc	10036	8691	8566	10563.91	10744	10593	22117	24122	21670.93	32326	35327.95	33266	178.31582	150.96809	152.28021	173.65377	175.87007	185.72508	300.57775	293.36401	315.42368	493.00241	477.342	477.54288	0.1524709	8.1359707	0.0917202	0.4774475	0.9171128	8.1359707	5.95E-24	2.34E-22	1.5881445	8.1359707	1.54E-66	4.74E-65
9111	ENSMUSG00	97827	Exd2	1	protein codi	MGI Symbol	7	exonuclease	738	685	657	773	843	774	1577	1564	1488	2133	2428	2572	13.112502	11.898877	11.679675	12.706883	13.799188	13.570397	21.43198	19.020866	21.658066	32.53029	32.8065	36.92179	0.1272854	4.3365138	0.2029706	0.6750986	0.7588005	4.3365138	6.63E-15	1.50E-13	1.4786487	4.3365138	1.92E-52	4.41E-51
9127	ENSMUSG00	252972	Tpcn1	1	protein codi	MGI Symbol	4	two pore cha	1156	1091	1092	1327	1345	1376	2278	2464	2130	3014	3363	3006	20.539367	18.95135	19.412793	21.813756	22.016498	24.12515	30.958815	29.966376	31.002474	45.966382	45.439975	43.151984	0.2060265	4.8846321	0.031632	0.2749349	0.6420372	4.8846321	1.26E-11	2.17E-10	1.1917554	4.8846321	3.00E-36	4.35E-35
9147	ENSMUSG00	76650	Srxn1	1	protein codi	MGI Symbol	3	sulfiredoxin	236	320	276	313	308	344	516	429	436	876	1109	929	4.1931579	5.5585996	4.9065302	5.145219	5.0416961	6.0312875	7.01262	5.2173602	6.3460463	13.359838	14.984517	13.336059	0.1439585	2.9404961	0.2036703	0.6754944	0.3385541	2.9404961	0.0019766	0.0068233	1.5072136	2.9404961	5.19E-47	1.04E-45
9166	ENSMUSG00	239273	Abcc4	1	protein codi	MGI Symbol	6	ATP-binding	293	261	314	265	299	271	513	502	413	649	906	806	5.2059121	4.5337328	5.582067	4.3561758	4.8943738	4.7513922	6.971849	6.1051627	6.0112778	9.8978706	12.241635	11.570359	-0.129605	2.791482	0.2575034	0.7317625	0.3174006	2.791482	0.0037085	0.0114629	1.1388546	2.791482	3.62E-27	3.58E-26
9207	ENSMUSG00	14225	Fkbp1a	1	protein codi	MGI Symbol	10	FK506 bindir	3736	3578	3734	4201	4294	3876	7654	8164	6823	9522	11981	11594	66.379822	62.152092	66.380376	69.057716	70.289101	67.957181	104.02053	99.287945	99.309803	145.21961	161.88413	166.43516	0.0889637	6.6192362	0.3319993	0.7826146	0.6346743	6.6192362	3.89E-12	7.05E-11	1.2807017	6.6192362	7.00E-44	1.31E-42
9223	ENSMUSG00	18018	Nfatc1	1	protein codi	MGI Symbol	7	nuclear fact	40	44	32	54	62	52	350	318	264	605	695	760	0.7107047	0.7643074	0.5688731	0.8876736	1.0148869	0.9117063	4.7566221	3.8674138	3.8425602	9.2268286	9.3906579	10.910016	0.4598663	2.0175471	0.0201617	0.2030461	2.6037977	2.0175471	1.41E-65	2.06E-63	3.8477755	2.0175471	5.99E-160	7.90E-158
9231	ENSMUSG00	106877	Afap1l1	1	protein codi	MGI Symbol	3	actin filamer	108	91	106.63	176	176	153	228.9	265	213	453	580.97	552.89	1.9189028	1.5807268	1.8955917	2.8931583	2.8809692	2.6825203	3.1108309	3.2228449	3.1002474	6.9086832	7.8499144	7.936893	0.6493984	1.9703992	2.51E-06	0.0001015	0.8071177	1.9703992	1.29E-09	1.76E-08	2.0731794	1.9703992	3.72E-63	1.09E-61
924	ENSMUSG00	83429	Ctns	1	protein codi	MGI Symbol	5	cystinosis, n	160	143	140	138	143	152	251	267	216	313	425	361	2.8428189	2.4839992	2.4888197	2.2684991	2.3407875	2.6649875	3.4111776	3.2471682	3.1439129	4.7735493	5.7424886	5.1822575	-0.104914	1.7856651	0.4558352	0.8562065	0.3271883	1.7856651	0.0092636	0.0241305	1.0076197	1.7856651	5.42E-17	2.86E-16
9241	ENSMUSG00	380928	Lmo7	1	protein codi	MGI Symbol	16	LIM domain	77	77	76	84	92	102	324	371	246	472	673	633	1.3681066	1.337538	1.3510735	1.3808255	1.5059612	1.7883469	4.4032731	4.5119828	3.5805674	7.1984514	9.0933996	9.0868948	0.2014646	2.0038484	0.2031277	0.6750986	1.6250121	2.0038484	2.24E-33	1.26E-31	2.6450324	2.0038484	1.91E-94	9.47E-93
9265	ENSMUSG00	208177	Phldb2	1	protein codi	MGI Symbol	11	pleckstrin hc	113	101	92	118	138	106	304	369	311	426	516	569	2.0077409	1.754433	1.6355101	1.9397311	2.2589418	1.8584781	4.1314661	4.4876595	4.5266523	6.4969074	6.9720568	8.1681566	0.1678557	1.985589	0.2509062	0.724754	1.2835024	1.985589	1.26E-23	4.92E-22	2.0025675	1.985589	9.28E-59	2.49E-57
9305	ENSMUSG00	19274	Ptprm	1	protein codi	MGI Symbol	7	protein tyro:	315	336	284	291	325	310	586	680	584	816	955	881	5.5967997	5.8365296	5.0487485	4.7835742	5.3199715	5.4351719	7.9639445	8.2699415	8.5002088	12.44478	12.90371	12.647005	-0.085777	2.9974586	0.4531969	0.8548924	0.5849921	2.9974586	3.92E-08	4.49E-07	1.2045601	2.9974586	6.76E-31	7.98E-30
9311	ENSMUSG00	210126	Lpp	1	protein codi	MGI Symbol	6	LIM domain	248	206	318	223	314	296	319	462	388	714	827	780	4.4063693	3.5783485	5.6531761	3.6657631	5.139911	5.1897125	4.3353213	5.6186956	5.647399	10.889183	11.174207	11.197122	0.0370052	2.6884869	0.7664738	0.9609679	0.1953469	2.6884869	0.0836871	0.1407735	1.2869113	2.6884869	7.06E-33	8.93E-32
9324	ENSMUSG00	54371	Chst2	1	protein codi	MGI Symbol	1	carbohydrat	697	666	613	699	633	578	1386	1252	1111	1885	2145	1840	12.38403	11.568835	10.897475	11.490441	10.361668	10.133966	18.836224	15.226422	16.170774	28.748053	28.982678	26.413723	-0.123381	4.0761485	0.2232515	0.7026163	0.52698	4.0761485	7.99E-08	8.69E-07	1.2715739	4.0761485	5.69E-39	9.13E-38
9383	ENSMUSG00	54324	Arhgef5	1	protein codi	MGI Symbol	8	Rho guanine	464.15	346.35	390	432.76	453	457	1043.28	1107.13	795.48	1492.58	1674.79	1553	8.24684	6.0163155	6.9331405	7.1138817	7.4152219	8.0124953	14.178539	13.464559	11.578332	22.763272	22.629324	22.293756	0.0886524	3.6634668	0.4101977	0.8370832	0.8889566	3.6634668	3.11E-18	9.19E-17	1.675242	3.6634668	3.14E-62	9.12E-61
9448	ENSMUSG00	244654	Mtss2	1	protein codi	MGI Symbol	6	MTSS I-BAR (	6341	4993	5103	5308	5826	5863	9050	10674	7964	13881	16020	15017	112.66447	86.731524	90.717477	87.255024	95.366628	102.79488	122.99266	129.81376	115.91723	211.69852	216.45804	215.5733	-0.023589	7.0490487	0.7962925	0.9697184	0.3460401	7.0490487	0.000139	0.0007307	1.149863	7.0490487	3.67E-36	5.31E-35
9480	ENSMUSG00	77057	Ston1	1	protein codi	MGI Symbol	7	stonin 1 [Sou	343	324	330	347	343	364	663	853	641	942	1139	1035	6.0942931	5.6280821	5.8665035	5.7041246	5.6146161	6.3819438	9.0104013	10.373912	9.3298525	14.366401	15.38987	14.857719	0.0084172	3.1940135	0.9466453	0.9996602	0.7080841	3.1940135	1.59E-11	2.72E-10	1.3429734	3.1940135	8.10E-39	1.30E-37
9484	ENSMUSG00	16601	Klf9	1	protein codi	MGI Symbol	2	Kruppel-like	512	519	445	328	286	324	799	989	904	1257	1398	1428	9.0970205	9.0153537	7.9108911	5.391795	4.681575	5.6806313	10.858689	12.0279	13.157857	19.170452	18.88941	20.499346	-0.724951	3.5194811	1.74E-11	1.40E-09	0.4692549	3.5194811	3.41E-06	2.72E-05	1.169751	3.5194811	7.44E-32	9.07E-31
9487	ENSMUSG00	19039	Lgals3bp	1	protein codi	MGI Symbol	5	lectin, galact	295	293	322	334	280	350	524	636	428	854	1203	968	5.2414474	5.0895928	5.7242852	5.4904254	4.5833601	6.1364844	7.1213428	7.7348277	6.2296051	13.024317	16.254621	13.895915	0.012468	3.0242779	0.9171199	0.9915152	0.3955464	3.0242779	0.0002593	0.0012544	1.427962	3.0242779	5.29E-43	9.58E-42
9490	ENSMUSG00	218699	Pxk	1	protein codi	MGI Symbol	6	PX domain c	201	142	146	190	225	195	292	257	251	384	482	378	3.5712913	2.4666286	2.5954834	3.1232959	3.6830572	3.4188984	3.9683819	3.1255514	3.6533432	5.8563672	6.5126577	5.4262973	0.2447841	2.0029289	0.0525901	0.3650868	0.3136535	2.0029289	0.0106616	0.0270904	1.0447127	2.0029289	6.66E-19	4.03E-18
95	ENSMUSG00	16402	Itga5	1	protein codi	MGI Symbol	4	integrin alph	227	175	189	168	177	195	255	247	217	670	882	862	4.0332493	3.0398592	3.3599066	2.7616511	2.8973383	3.4188984	3.465539	3.0039346	3.158468	10.218141	11.917353	12.374255	-0.201973	2.4290495	0.1089341	0.5191452	-0.116359	2.4290495	0.3374491	0.4296123	1.7266811	2.4290495	1.16E-54	2.85E-53
9510	ENSMUSG00	228140	Tnks1bp1	1	protein codi	MGI Symbol	8	tankyrase 1	296	224	271	278	233	265	450	539	432	867	1014	984	5.259215	3.8910197	4.8176438	4.569875	3.8140104	4.6461953	6.115657	6.5551448	6.2878257	13.222579	13.700902	14.125599	-0.100475	2.8764955	0.3897022	0.8245501	0.4418655	2.8764955	6.18E-05	0.0003604	1.555702	2.8764955	4.45E-48	9.12E-47
9598	ENSMUSG00	78926	Gas2l1	1	protein codi	MGI Symbol	7	growth arre:	272	253	235	259	252	263	439	548	476	915	1238	1103	4.8327922	4.3947678	4.1776616	4.2575454	4.1250241	4.6111297	5.9661632	6.6645999	6.9282524	13.954625	16.727532	15.833878	-0.045739	2.9655384	0.7091686	0.9449835	0.5446621	2.9655384	8.61E-07	7.85E-06	1.7952647	2.9655384	3.30E-63	9.75E-62
9600	ENSMUSG00	94352	Loxl2	1	protein codi	MGI Symbol	2	lysyl oxidase	1218.58	1294.87	1320.65	1300.31	1377.35	1469.56	7949.31	9095.93	7441.8	13766.86	16663.49	15234.04	21.651264	22.4927	23.477569	21.375015	22.546039	25.76552	108.0339	110.62178	108.31653	209.95778	225.15271	218.68897	0.0430119	6.5452301	0.6528437	0.9285027	2.2735414	6.5452301	3.62E-124	1.37E-121	3.2732071	6.5452301	1.81E-239	5.94E-237
9625	ENSMUSG00	224454	Zdhhc14	1	protein codi	MGI Symbol	1	zinc finger, [	261	290	214	245	246	235	508	643	449	732	867	651	4.6373484	5.0374809	3.8043386	4.0274078	4.0268092	4.1202109	6.9038973	7.8199594	6.5352633	11.1637	11.714677	9.3452899	-0.147556	2.7421385	0.2135472	0.6905376	0.6585575	2.7421385	2.16E-09	2.88E-08	1.2567394	2.7421385	2.54E-31	3.05E-30
9659	ENSMUSG00	70099	Smc4	1	protein codi	MGI Symbol	11	structural m	420	364	360	426	416	451	725	716	650	884	1076	1023	7.4623997	6.322907	6.399822	7.0027581	6.8095636	7.9072985	9.853003	8.707762	9.4608489	13.481845	14.53863	14.685456	0.1050173	3.2424897	0.3303819	0.7813364	0.4725442	3.2424897	5.62E-06	4.27E-05	1.0814439	3.2424897	3.90E-26	3.68E-25
9674	ENSMUSG00	67946	Spata6	1	protein codi	MGI Symbol	14	spermatoge	134	149	167	189	186	196	241	318	267	396	555	473	2.3808608	2.5882229	2.9688063	3.1068575	3.0446606	3.4364313	3.2752741	3.8674138	3.8862256	6.0393787	7.4990146	6.7900493	0.2712536	2.0518678	0.0344664	0.2895053	0.4746438	2.0518678	0.0001287	0.0006843	1.3578385	2.0518678	9.47E-31	1.11E-29
9690	ENSMUSG00	225912	Cyb561a3	1	protein codi	MGI Symbol	9	cytochrome	271.45	243.15	244.05	280.88	241.11	280.4	969.18	1005.46	824.52	2513.07	3761.72	3678.73	4.82302	4.2236672	4.338546	4.6172176	3.9467641	4.9162006	13.171494	12.228081	12.001014	38.326721	50.827375	52.809214	0.0091812	4.1184597	0.9489986	1	1.4823003	4.1184597	1.95E-43	1.60E-41	3.4068454	4.1184597	1.18E-218	2.87E-216
9700	ENSMUSG00	219151	Scara3	1	protein codi	MGI Symbol	1	scavenger re	250	231	288	286	301	291	741	705	554	1669	2007	2014	4.4419046	4.0126141	5.1198576	4.7013822	4.9271121	5.1020485	10.070449	8.5739835	8.0635543	25.453846	27.118058	28.911542	0.1179311	3.5253594	0.3114615	0.7696817	0.9768591	3.5253594	2.09E-19	6.56E-18	2.585758	3.5253594	7.63E-130	7.18E-128
9705	ENSMUSG00	54004	Diaph2	1	protein codi	MGI Symbol	7	diaphanous	174	164	193	202	258	249	255	318	245	493	546	608	3.0915656	2.8487823	3.4310157	3.3205567	4.2232389	4.3656703	3.465539	3.8674138	3.5660123	7.5187215	7.3774089	8.7280127	0.3447985	2.2365743	0.0047019	0.0715831	0.2197883	2.2365743	0.0707846	0.12316	1.333752	2.2365743	1.63E-31	1.97E-30
9730	ENSMUSG00	72278	Ccpg1	1	protein codi	MGI Symbol	10	cell cycle prc	1598.02	1655.19	1762.73	1839.53	1771.83	1752.98	2802.48	3061	2385.46	3758.42	4400.02	4439.31	28.393009	28.751683	31.336551	30.238929	29.003339	30.73467	38.086681	37.226899	34.720733	57.319499	59.451917	63.727556	0.0241348	5.2915663	0.7993902	0.9701843	0.3146738	5.2915663	0.0007516	0.003107	1.0285677	5.2915663	3.17E-28	3.30E-27
9739	ENSMUSG00	217310	Hid1	1	protein codi	MGI Symbol	8	HID1 domair	253	269	232	246	281	243	464	544	417	677	859	800	4.4952074	4.6726978	4.1243297	4.0438462	4.5997292	4.2604734	6.3059219	6.6159532	6.0694984	10.324897	11.606583	11.484227	-0.042813	2.7311433	0.7197374	0.9477493	0.5153305	2.7311433	3.30E-06	2.65E-05	1.3299196	2.7311433	8.03E-35	1.10E-33
9748	ENSMUSG00	58250	Chst11	1	protein codi	MGI Symbol	1	carbohydrat	207	211	257	234	229	238	318	380	283	556	713	707	3.677897	3.6652016	4.5687618	3.8465855	3.7485338	4.1728094	4.321731	4.6214379	4.119108	8.4795317	9.6338692	10.149186	-0.018029	2.4562787	0.9015455	0.9896888	0.1350754	2.4562787	0.2521929	0.3401238	1.247211	2.4562787	9.46E-30	1.06E-28
975	ENSMUSG00	69660	Tmbim1	1	protein codi	MGI Symbol	8	transmembr	637	576	727	865	882	785	1746	1800	1564	3278	4017	3945	11.317973	10.005479	12.924085	14.219215	14.437584	13.763258	23.728749	21.891022	22.764258	49.992635	54.276651	56.631595	0.3091327	4.6800771	0.0019633	0.0357998	0.9976443	4.6800771	1.59E-24	6.38E-23	2.2322982	4.6800771	4.22E-115	3.13E-113
9787	ENSMUSG00	14824	Grn	1	protein codi	MGI Symbol	11	granulin [Sou	2921	2618	2872	2744	2849	2805	5455	5970	4813	7554	10228	9979	51.899213	45.476293	51.056358	45.106968	46.635689	49.179539	74.135353	72.605222	70.053947	115.20572	138.19806	143.25138	-0.074934	6.2355452	0.4180874	0.8407314	0.5466017	6.2355452	2.69E-09	3.53E-08	1.4181944	6.2355452	9.37E-53	2.18E-51
9863	ENSMUSG00	214597	Sidt2	1	protein codi	MGI Symbol	14	SID1 transm	328.63	293.68	281.46	354.91	405.75	328.04	561.97	593.96	550.55	830.72	913.61	932.08	5.8389724	5.1014048	5.0035942	5.8341523	6.6417799	5.7514638	7.6373684	7.2235507	8.013339	12.669274	12.344459	13.380273	0.1937747	3.0060935	0.082573	0.4563371	0.5196673	3.0060935	1.28E-06	1.12E-05	1.2675938	3.0060935	6.74E-34	8.90E-33
9874	ENSMUSG00	20166	Rtkn	1	protein codi	MGI Symbol	9	rhotekin [So	224.89	176.98	198.89	202.61	204.54	202.72	345.93	302.16	296.46	488.66	603.55	577.23	3.9957597	3.074253	3.5357239	3.3305841	3.3481446	3.5542518	4.7013094	3.6747728	4.3150204	7.4525323	8.1550094	8.2863006	-0.051546	2.2790058	0.6962746	0.9427892	0.2568079	2.2790058	0.029443	0.0608194	1.1726157	2.2790058	1.84E-25	1.65E-24
9923	ENSMUSG00	232146	Eva1a	1	protein codi	MGI Symbol	4	eva-1 homol	125	167	86	158	154	145	411	356	298	954	1148	1023	2.2209523	2.9008942	1.5288464	2.5972671	2.5208481	2.5422578	5.5856334	4.3295576	4.3374353	14.549412	15.511475	14.685456	0.2017994	2.6408085	0.1343006	0.5634628	1.0964328	2.6408085	5.14E-19	1.59E-17	2.74738	2.6408085	1.04E-122	8.36E-121
9924	ENSMUSG00	112407	Egln3	1	protein codi	MGI Symbol	1	egl-9 family	157	151	127	161	164	184	576	484	405	590	1062	842	2.7895161	2.6229642	2.257715	2.6465823	2.6845395	3.2260375	7.828041	5.8862525	5.8948366	8.9980642	14.349466	12.087149	0.1554655	2.6017278	0.2385029	0.7163933	1.3528717	2.6017278	1.75E-30	8.73E-29	2.2090722	2.6017278	6.39E-82	2.55E-80
9950	ENSMUSG00	320487	Heatr5a	1	protein codi	MGI Symbol	8	HEAT repeat	181	138	157	171	180	182	324	340	282	436	468	553	3.2159389	2.3971461	2.7910335	2.8109663	2.9464458	3.1909719	4.4032731	4.1349708	4.1045529	6.6494169	6.3234934	7.938472	0.0902015	2.1105472	0.5013113	0.8720852	0.5895927	2.1105472	1.11E-06	9.84E-06	1.3149855	2.1105472	1.85E-29	2.04E-28
9958	ENSMUSG00	65106	Arl6ip5	1	protein codi	MGI Symbol	1	ADP-ribosyla	406	428	444	484	506	469	777	876	685	1139	1353	1277	7.213653	7.434627	7.8931138	7.9561853	8.2827865	8.2228891	10.559701	10.653631	9.9702792	17.370839	18.281381	18.331698	0.1179011	3.4727096	0.2668909	0.7370376	0.4686362	3.4727096	5.14E-06	3.95E-05	1.2599489	3.4727096	6.55E-36	9.38E-35
9966	ENSMUSG00	230514	Leprot	1	protein codi	MGI Symbol	2	leptin recept	476	486	472	579	599	503	1002	1062	895	1457	1953	1849	8.4573863	8.4421231	8.3908777	9.5178332	9.8051168	8.8190047	13.61753	12.915703	13.026861	22.220643	26.388424	26.54292	0.1545541	3.8196063	0.1382986	0.5727276	0.6452374	3.8196063	1.57E-10	2.36E-09	1.5714339	3.8196063	2.31E-56	5.93E-55
9971	ENSMUSG00	236900	Pdk3	1	protein codi	MGI Symbol	3	pyruvate deł	302	294	288	295	334	292	623	726	626	1052	1302	1198	5.3658207	5.1069634	5.1198576	4.8493278	5.4672938	5.1195813	8.4667874	8.8293788	9.1115252	16.044006	17.592283	17.19763	-0.014371	3.1911115	0.9094367	0.9905825	0.7597303	3.1911115	9.82E-13	1.90E-11	1.7049554	3.1911115	5.14E-61	1.46E-59
9983	ENSMUSG00	320712	Abi3bp	1	protein codi	MGI Symbol	8	ABI gene fan	294	383	498	614	724	639	1091	1919	1078	2774	3019	2906	5.2236798	6.6529489	8.8530871	10.093177	11.85126	11.203467	14.827071	23.338261	15.690454	42.306153	40.791937	41.716455	0.6771351	4.2876531	7.56E-11	5.61E-09	1.3795667	4.2876531	2.23E-42	1.76E-40	2.5897562	4.2876531	7.15E-146	8.28E-144
10060	ENSMUSG00	216166	Plk5	2	protein codi	MGI Symbol	4	polo like kin	366	362	278	277	221	224	1341	1687	1217	1607	2329	1826	6.5029483	6.2881658	4.9420848	4.5534366	3.6175807	3.92735	18.224658	20.516752	17.71362	24.508287	31.468838	26.212749	-0.551394	3.8298366	1.43E-06	6.05E-05	1.670813	3.8298366	3.04E-60	4.01E-58	2.2124913	3.8298366	3.79E-105	2.26E-103
10103	ENSMUSG00	666060	Frmpd1	2	protein codi	MGI Symbol	3	FERM and PI	305	281	347	299	283	217	1000	1279	823	1057	1210	1040	5.4191236	4.8811453	6.1687173	4.9150814	4.6324675	3.8046203	13.590349	15.554787	11.97889	16.120261	16.349203	14.929495	-0.300296	3.3225188	0.0080372	0.1068883	1.3220026	3.3225188	3.19E-37	2.17E-35	1.5252585	3.3225188	2.52E-49	5.38E-48
10175	ENSMUSG00	78906	Misp	2	protein codi	MGI Symbol	6	mitotic spinc	2802	1569	3331	38	34	18	3467	4027	2121	2416	2017	2171	49.784866	27.254509	59.216131	0.6246592	0.5565509	0.3155906	47.11774	48.97508	30.871478	36.84631	27.253175	31.165322	-6.492134	4.9104764	0	0	-0.100706	4.9104764	0.2735571	0.3633151	-0.516634	4.9104764	2.81E-08	6.35E-08
10275	ENSMUSG00	66042	Sostdc1	2	protein codi	MGI Symbol	1	sclerostin dc	73	68	100	106	119	96	661	665	508	741	686	570	1.2970361	1.1812024	1.7777283	1.7424703	1.947928	1.68315	8.9832206	8.0875164	7.3940173	11.300959	9.2690522	8.1825119	0.3384809	2.4307634	0.0292705	0.2599914	2.5234414	2.4307634	1.98E-87	4.09E-85	2.7544183	2.4307634	4.86E-105	2.89E-103
10315	ENSMUSG00	16981	Lrrn3	2	protein codi	MGI Symbol	1	leucine rich	76	47	85	79	96	76	288	329	286	286	247	224	1.350339	0.8164193	1.5110691	1.2986336	1.5714377	1.3324938	3.9140205	4.0011923	4.1627735	4.3617735	3.3373993	3.2155836	0.1959165	1.4168538	0.2435495	0.72048	1.7160026	1.4168538	1.30E-35	8.10E-34	1.5671109	1.4168538	4.00E-29	4.33E-28
1040	ENSMUSG00	330064	Slc5a6	2	protein codi	MGI Symbol	7	solute carrie	111.94	130.99	109.15	130.87	122.97	126.26	236.7	273.76	220.97	292.83	350.56	282.98	1.9889072	2.275378	1.9403905	2.1512933	2.0129135	2.2136929	3.2168356	3.3293812	3.216252	4.4659375	4.7366749	4.0622583	0.038229	1.6033154	0.7989806	0.9701843	0.6534197	1.6033154	4.42E-07	4.23E-06	1.095425	1.6033154	3.36E-18	1.94E-17
10501	ENSMUSG00	52538	Acaa2	2	protein codi	MGI Symbol	2	acetyl-Coenz	183	201	224	208	201	190	436	517	395	454	734	552	3.2514741	3.4914954	3.9821115	3.4191871	3.2901978	3.3312344	5.9253921	6.2875879	5.7492851	6.9239342	9.9176157	7.9241168	-0.094899	2.4293038	0.457092	0.8564485	0.7449084	2.4293038	6.75E-11	1.05E-09	1.2094626	2.4293038	4.16E-27	4.10E-26
10566	ENSMUSG00	20608	Sstr4	2	protein codi	MGI Symbol	1	somatostatir	11	13	9	14	10	17	299	318	252	270	267	226	0.1954438	0.2258181	0.1599956	0.2301376	0.1636914	0.2980578	4.0635143	3.8674138	3.6678983	4.1177582	3.607634	3.2442942	0.2383033	1.0867131	0.5747599	0.902499	4.3042102	1.0867131	1.08E-105	2.86E-103	4.2209638	1.0867131	5.99E-100	3.23E-98
10567	ENSMUSG00	242022	Frem2	2	protein codi	MGI Symbol	4	Fras1 relatec	718	465	1021	732	1049	1040	1579	2614	2124	1149	1207	1436	12.75715	8.07734	18.150606	12.032908	17.171231	18.234125	21.459161	31.790628	30.915143	17.523349	16.308668	20.614188	0.2831705	4.2366072	0.0041241	0.0645457	1.1112403	4.2366072	1.47E-30	7.37E-29	0.4824229	4.2366072	7.85E-07	1.51E-06
1070	ENSMUSG00	15114	Hap1	2	protein codi	MGI Symbol	5	huntingtin-a	377	260	284	295	311	302	695	703	587	705	790	884	6.6983921	4.5163622	5.0487485	4.8493278	5.0908035	5.2949094	9.4452925	8.5496601	8.5438743	10.751924	10.674273	12.690071	-0.09404	2.9578975	0.4149444	0.838693	0.7067946	2.9578975	2.79E-11	4.60E-10	1.0691684	2.9578975	2.22E-24	1.87E-23
10716	ENSMUSG00	18787	Serpine1	2	protein codi	MGI Symbol	3	serine (or cy	110	95	113	60	82	87	479	641	706	448	638	410	1.954438	1.6502093	2.008833	0.986304	1.3422697	1.5253547	6.5097771	7.7956361	10.275937	6.8324284	8.6204888	5.8856664	-0.544946	2.2516722	0.0004086	0.0094444	2.1278117	2.2516722	2.69E-68	4.05E-66	1.9280533	2.2516722	3.70E-55	9.19E-54
10812	ENSMUSG00	272636	Esyt3	2	protein codi	MGI Symbol	5	extended sy	449.91	408.96	435	429	467.89	444	1359.81	1517.58	1039.95	1262.89	1606.95	1394.86	7.9938291	7.1038903	7.7331183	7.0520733	7.6589584	7.7845688	18.480292	18.45632	15.13663	19.26028	21.712687	20.023611	-0.021447	3.7368487	0.8477426	0.9812274	1.1904649	3.7368487	2.54E-32	1.35E-30	1.4180988	3.7368487	2.37E-45	4.60E-44
10904	ENSMUSG00	20778	Scarb1	2	protein codi	MGI Symbol	11	scavenger re	551	578	572	572	569	610	1478	1591	1263	1601	1965	1806	9.7899576	10.040221	10.168606	9.4027644	9.3140425	10.695016	20.086536	19.349231	18.383157	24.416781	26.550565	25.925643	-0.029182	4.0264848	0.7839121	0.9653561	0.9466883	4.0264848	8.20E-22	2.94E-20	1.3579797	4.0264848	2.06E-43	3.79E-42
10953	ENSMUSG00	140559	Igsf8	2	protein codi	MGI Symbol	8	immunoglob	342	292	346	297	303	313	754	712	567	832	873.04	799.03	6.0765254	5.0722221	6.15094	4.8822046	4.9598504	5.4877703	10.247123	8.6591153	8.2527712	12.688796	11.796288	11.470303	-0.174732	3.012277	0.122561	0.5421773	0.6511197	3.012277	6.83E-10	9.65E-09	1.0552871	3.012277	4.04E-24	3.33E-23
11007	ENSMUSG00	14869	Gstp2	2	protein codi	MGI Symbol	6	glutathione	1651.19	1685.43	1643.19	1905.32	1764.82	1789.68	3966.04	4217.7	4290.76	4100.23	5640.42	4911.34	29.337714	29.27697	29.211454	31.320411	28.888591	31.378124	53.899867	51.294312	62.452664	62.532429	76.211877	70.50368	0.0604051	5.5385217	0.5218371	0.8814816	0.9323285	5.5385217	1.57E-23	6.10E-22	1.2526315	5.5385217	7.11E-41	1.21E-39
11067	ENSMUSG00	73102	Slc22a23	2	protein codi	MGI Symbol	5	solute carrie	605	523	675	670	686	598	1445	1855	1451	2032	2045	1860	10.749409	9.0848362	11.999666	11.013728	11.229232	10.484622	19.638054	22.559914	21.119526	30.989943	27.631504	26.700828	0.040558	4.1608435	0.6965147	0.9427938	0.9926173	4.1608435	4.81E-24	1.90E-22	1.4222657	4.1608435	7.67E-48	1.55E-46
11110	ENSMUSG00	66425	Pcp4l1	2	protein codi	MGI Symbol	1	Purkinje cell	535	669	477	700	852	701	4290	3934	2914	3912	5359	3969	9.5056757	11.620947	8.4797642	11.50688	13.94651	12.290502	58.302597	47.844044	42.413713	59.661741	72.409404	56.976122	0.3499799	5.085481	0.0005336	0.0116804	2.3265266	5.085481	3.64E-123	1.33E-120	2.6742803	5.085481	3.52E-160	4.84E-158
11121	ENSMUSG00	56338	Txnip	2	protein codi	MGI Symbol	6	thioredoxin i	143	173.7	181	168	270.51	162.63	1166.13	690.28	985.93	1410	1160.85	762.83	2.5407694	3.0172773	3.2176883	2.7616511	4.4280169	2.8513613	15.848114	8.3949636	14.350361	21.503848	15.685101	10.950641	0.1964512	3.1609991	0.118612	0.5353037	2.1325139	3.1609991	5.59E-80	1.02E-77	2.4532299	3.1609991	1.39E-105	8.52E-104
11242	ENSMUSG00	14745	Lpar1	2	protein codi	MGI Symbol	8	lysophospha	15	7	19	16	19	12	406	315	282	517	491	431	0.2665143	0.1215944	0.3377684	0.2630144	0.3110137	0.2103938	5.5176817	3.8309288	4.1045529	7.8847444	6.6342633	6.1871274	0.1218303	1.6432999	0.7590449	0.9601813	4.2048363	1.6432999	4.04E-114	1.32E-111	4.8265403	1.6432999	2.64E-163	3.91E-161
11272	ENSMUSG00	243961	Shank1	2	protein codi	MGI Symbol	6	SH3 and mul	533.79	422.76	374.7	345.8	390.98	428.79	2470.96	3498.71	2296.82	1776.84	2010.93	1588.91	9.4841769	7.3436049	6.6611481	5.6843985	6.4000076	7.5178947	33.581209	42.550187	33.430564	27.098509	27.171159	22.809254	-0.262157	4.2740143	0.0146002	0.1637562	2.2222778	4.2740143	4.06E-110	1.20E-107	1.7142801	4.2740143	3.67E-66	1.12E-64
11291	ENSMUSG00	22351	Vill	2	protein codi	MGI Symbol	10	villin-like [Sc	70	42	56	66	68	55	251	325	271	320	429	399	1.2437333	0.7295662	0.9955279	1.0849344	1.1131017	0.9643047	3.4111776	3.9525456	3.9444462	4.880306	5.7965356	5.7277583	0.0950692	1.5552164	0.6481117	0.9272839	1.9299063	1.5552164	5.21E-41	4.05E-39	2.4674616	1.5552164	5.62E-70	1.85E-68
1135	ENSMUSG00	15903	Id3	2	protein codi	MGI Symbol	3	inhibitor of [	989	921	724	848	700	861	2101	1912	1392	2054	2670	2212	17.572174	15.998344	12.870753	13.939763	11.4584	15.095752	28.553323	23.25313	20.260772	31.325464	36.07634	31.753888	-0.198448	4.4343186	0.0447319	0.3364636	0.6341332	4.4343186	4.07E-11	6.51E-10	1.0944136	4.4343186	3.36E-30	3.85E-29
11393	ENSMUSG00	16790	Anpep	2	protein codi	MGI Symbol	6	alanyl (mem	122	75	80	96	103	107	819	929	715	719	988	1035	2.1676494	1.3027968	1.4221827	1.5780863	1.6860218	1.8760109	11.130496	11.2982	10.406934	10.965438	13.349597	14.857719	0.0706406	2.8114523	0.642749	0.92573	2.7471392	2.8114523	1.14E-110	3.50E-108	3.0019045	2.8114523	7.74E-133	7.58E-131
11405	ENSMUSG00	237339	L3mbtl3	2	protein codi	MGI Symbol	7	L3MBTL3 his	267	228	260	299	306	347	610	716	569	769	783	798	4.7439541	3.9605022	4.6220937	4.9150814	5.0089578	6.083886	8.2901128	8.707762	8.2818815	11.727985	10.579691	11.455517	0.2632696	2.8989659	0.020852	0.206779	0.9243718	2.8989659	2.29E-17	6.19E-16	1.3407077	2.8989659	2.07E-35	2.91E-34
11489	ENSMUSG00	58188	Vstm2b	2	protein codi	MGI Symbol	4	V-set and tra	143	85	118	106	99	97	956	1009	800.89	851	951.97	842.94	2.5407694	1.476503	2.0977194	1.7424703	1.6205452	1.7006828	12.992374	12.271134	11.657076	12.978564	12.862769	12.100643	-0.270242	2.8780218	0.0644778	0.399813	2.5949436	2.8780218	1.91E-106	5.18E-104	2.6339525	2.8780218	3.88E-109	2.56E-107
11541	ENSMUSG00	66797	Cntnap2	2	protein codi	MGI Symbol	6	contactin as:	336	341	398	403	405	383	1264	1353	1026	1622	1847	1774	5.9699197	5.9233827	7.0753588	6.624675	6.629503	6.7150672	17.178201	16.454751	14.933586	24.737051	24.95618	25.466274	0.0742849	3.7760908	0.5002633	0.8719351	1.3567523	3.7760908	2.37E-40	1.78E-38	1.9862578	3.7760908	9.89E-86	4.10E-84
1155	ENSMUSG00	232370	Clstn3	2	protein codi	MGI Symbol	9	calsyntenin :	119	122	123	133	126	134	378	430	388	429	352	329	2.1143466	2.1192161	2.1866059	2.1863071	2.062512	2.3493969	5.1371519	5.2295219	5.647399	6.5426603	4.7561318	4.7228884	0.0382198	1.9448073	0.8034302	0.9701985	1.3170423	1.9448073	6.71E-27	2.95E-25	1.315501	1.9448073	1.45E-26	1.40E-25
11569	ENSMUSG00	98999	Znfx1	2	protein codi	MGI Symbol	5	zinc finger, N	401	347	311	453	443	489	681	820	750	837	913	860	7.1248149	6.0276064	5.5287351	7.4465949	7.2515304	8.5735453	9.2550276	9.9725766	10.916364	12.76505	12.336217	12.345544	0.3160686	3.2025514	0.003242	0.0536574	0.6895987	3.2025514	3.67E-11	5.93E-10	1.0029672	3.2025514	2.96E-22	2.23E-21
11616	ENSMUSG00	16509	Kcne1	2	protein codi	MGI Symbol	2	potassium v	145	147	160	194	171	188	415	457	354	357	417	409	2.5763046	2.5534817	2.8443653	3.1890495	2.7991235	3.2961688	5.6399948	5.5578872	5.1525238	5.4445914	5.6343947	5.8713111	0.2184725	2.1043858	0.0913231	0.4773794	1.0364901	2.1043858	2.97E-18	8.83E-17	1.0878801	2.1043858	7.42E-20	4.79E-19
1166	ENSMUSG00	22190	Ubc	2	protein codi	MGI Symbol	3	ubiquitin C [	12158.12	10867.69	13397.56	13378.57	13904.73	13301.47	26085.61	28749.97	24507.62	32639.46	35789.05	31264.63	216.02083	188.77855	238.17222	219.92228	227.60852	233.21218	354.51254	349.6479	356.71214	497.78298	483.57227	448.81264	0.082362	8.3129047	0.3613753	0.8029654	0.7224322	8.3129047	1.42E-15	3.41E-14	1.153364	8.3129047	1.02E-36	1.53E-35
11844	ENSMUSG00	108151	Sema3d	2	protein codi	MGI Symbol	5	sema domai	180	147	202	192	221	233	319	431	338	471	434	450	3.1981713	2.5534817	3.5910112	3.1561727	3.6175807	4.0851453	4.3353213	5.2416835	4.9196414	7.1832004	5.8640943	6.4598778	0.2160437	2.1974499	0.0818019	0.4555204	0.6357352	2.1974499	6.63E-08	7.33E-07	1.0609751	2.1974499	4.47E-20	2.92E-19
11850	ENSMUSG00	101476	Plekha1	2	protein codi	MGI Symbol	12	pleckstrin hc	583	457	600	535	570	575	1323	1427	1190	1575	1815	1725	10.358521	7.938375	10.66637	8.7945437	9.3304116	10.081367	17.980032	17.354716	17.320631	24.020256	24.523804	24.762865	-0.038263	3.942372	0.7158224	0.9467164	0.8626832	3.942372	3.17E-18	9.31E-17	1.3400723	3.942372	4.35E-42	7.65E-41
11854	ENSMUSG00	12571	Cdk6	2	protein codi	MGI Symbol	6	cyclin-deper	585	535	629	599	724	669.99	1621	2099	1472	1715	1805	1657	10.394057	9.2932837	11.181911	9.8466012	11.85126	11.746809	22.029956	25.527364	21.425184	26.15539	24.388687	23.786706	0.1155079	4.1232967	0.2571163	0.7310999	1.1608931	4.1232967	2.89E-32	1.53E-30	1.2676808	4.1232967	3.87E-38	6.02E-37
11914	ENSMUSG00	245007	Zbtb38	2	protein codi	MGI Symbol	12	zinc finger a	843	736	873	880	1056	993	1496	1579	1945	2101	2066	2132	14.978102	12.784779	15.519568	14.465791	17.285815	17.410083	20.331162	19.203291	28.309771	32.042259	27.915251	30.605465	0.1836685	4.3913893	0.0613539	0.3938332	0.647064	4.3913893	1.91E-11	3.24E-10	1.0649529	4.3913893	1.82E-28	1.92E-27
12109	ENSMUSG00	116914	Slc19a2	2	protein codi	MGI Symbol	4	solute carrie	156	145	156	145	171	158	314	343	310	428	425	386	2.7717484	2.5187404	2.7732562	2.3835679	2.7991235	2.7701844	4.2673696	4.1714558	4.5120972	6.5274093	5.7424886	5.5411396	-0.020258	1.990389	0.8865003	0.9860841	0.6823664	1.990389	1.75E-08	2.10E-07	1.1418944	1.990389	6.02E-22	4.46E-21
12260	ENSMUSG00	NA	Serpina3h	2	transcribed	MGI Symbol	2	serine (or cy	697.59	457.67	1056.47	0	0	0	985.39	1217.71	1183.64	3	1157.78	1264.87	12.394513	7.9500134	18.781167	0	0	0	13.391794	14.809398	17.22806	0.0457529	15.64362	18.157568	-12.56687	3.3179198	5.77568776	0.00E+00	0.2155241	3.3179198	0.0278535	0.0581685	-0.200959	3.3179198	0.0406281	0.0482519
12287	ENSMUSG00	80888	Hspb8	2	protein codi	MGI Symbol	2	heat shock p	126	104	157	218	229	252	696	659	752	771	926	761	2.2387199	1.8065449	2.7910335	3.5835711	3.7485338	4.4182688	9.4588829	8.0145463	10.945474	11.758487	12.511869	10.924371	0.7804444	2.8040886	9.09E-10	6.12E-08	2.0533534	2.8040886	3.11E-68	4.61E-66	2.3647067	2.8040886	1.31E-90	5.91E-89
12291	ENSMUSG00	14264	Fmod	2	protein codi	MGI Symbol	2	fibromodulir	108	121	135	166	155	159	581	711	562	728	617	515	1.9189028	2.1018455	2.3999333	2.7287743	2.5372172	2.7877172	7.8959927	8.6469536	8.1799955	11.102696	8.3367423	7.3929712	0.3260909	2.4945675	0.0170898	0.1818064	1.9448561	2.4945675	1.25E-59	1.63E-57	2.0603201	2.4945675	8.78E-67	2.71E-65
12301	ENSMUSG00	237979	Sdk2	2	protein codi	MGI Symbol	6	sidekick cell	5410	4324	5821	4567	5549	4985	12426	15137	15187	21544	20012	20603	96.122815	75.110577	103.48157	75.07417	90.832376	87.401071	168.87368	184.09133	221.04909	328.5666	270.3969	295.76192	-0.11701	7.3797063	0.1992786	0.6693502	1.0631114	7.3797063	3.18E-31	1.64E-29	1.7035048	7.3797063	2.45E-75	8.91E-74
12376	ENSMUSG00	170829	Tram2	2	protein codi	MGI Symbol	2	translocatin	162	118	143	146	171	191	364	420	358	467	481	404	2.8783542	2.0497336	2.5421515	2.4000063	2.7991235	3.3487672	4.946887	5.1079051	5.2107445	7.1221966	6.499146	5.7995347	0.1925584	2.1088866	0.1399881	0.5757088	1.0313615	2.1088866	1.26E-17	3.50E-16	1.3779505	2.1088866	5.54E-31	6.57E-30
12708	ENSMUSG00	81907	Tmem108	2	protein codi	MGI Symbol	6	transmembr	467.05	425.42	459.63	483.02	575.77	451.01	791.24	983.26	881.7	1255.23	1106.04	1047.47	8.2983661	7.3898107	8.1709727	7.9400757	9.4248616	7.9074738	10.753228	11.958092	12.833278	19.143458	14.944523	15.036729	0.0837644	3.4894198	0.4309885	0.8463211	0.5748046	3.4894198	1.58E-08	1.91E-07	1.0409458	3.4894198	4.37E-25	3.84E-24
12738	ENSMUSG00	20708	Serpinb6b	2	protein codi	MGI Symbol	4	serine (or cy	56	65	51	93	107	110	557	612	444	316	425	401	0.9949866	1.1290905	0.9066415	1.5287711	1.7514983	1.9286094	7.5698244	7.4429474	6.4624875	4.8193022	5.7424886	5.7564689	0.7769239	1.9944202	1.99E-06	8.20E-05	2.8229457	1.9944202	3.38E-96	8.03E-94	2.4270312	1.9944202	2.93E-68	9.39E-67
12772	ENSMUSG00	14702	Gng2	2	protein codi	MGI Symbol	6	guanine nuc	919	930.65	959.23	1084.62	1136	1108.72	2040	2350.47	1941.78	2288.46	2563.97	2469.82	16.328441	16.165971	17.052503	17.829417	18.595347	19.43898	27.724312	28.585661	28.262903	34.901203	34.643691	35.454967	0.1729474	4.6253758	0.0749942	0.4330969	0.7714164	4.6253758	6.91E-16	1.71E-14	1.0834526	4.6253758	8.15E-30	9.14E-29
12929	ENSMUSG00	73713	Rbm20	2	protein codi	MGI Symbol	3	RNA binding	208	202	158	321	246	306	825	844	778	655	678	568	3.6956646	3.508866	2.8088108	5.2767262	4.0268092	5.3650406	11.212038	10.264457	11.323908	9.9893764	9.1609583	8.1538013	0.5489664	2.8449504	3.78E-06	0.0001455	1.7101646	2.8449504	2.48E-53	2.64E-51	1.4457904	2.8449504	6.74E-38	1.04E-36
13236	ENSMUSG00	380694	Ccnjl	2	protein codi	MGI Symbol	2	cyclin J-like		241	218	197	316	241	260	535	574	410	614	574	522	4.281996	3.786796	3.5021248	5.1945342	3.9449635	4.5585313	7.2708367	6.9808036	5.9676124	9.3640872	7.7557376	7.4934582	0.2433301	2.5665478	0.0388629	0.3130219	0.8064657	2.5665478	6.05E-13	1.19E-11	1.0872822	2.5665478	1.39E-22	1.07E-21
13237	ENSMUSG00	16513	Kcnj10	2	protein codi	MGI Symbol	1	potassium ir	138	138	138	234	203	241	465	475	517	599	524	474	2.4519313	2.3971461	2.4532651	3.8465855	3.3229361	4.2254078	6.3195123	5.7767974	7.5250136	9.1353228	7.0801507	6.8044046	0.6399747	2.3802258	4.30E-07	1.99E-05	1.4224419	2.3802258	5.29E-33	2.89E-31	1.6538208	2.3802258	1.54E-44	2.92E-43
13476	ENSMUSG00	16493	Kcna5	2	protein codi	MGI Symbol	1	potassium v	94	91	77	81	90	85	999	1252	883.99	868	1156	1202	1.6701561	1.5807268	1.3688508	1.3315104	1.4732229	1.4902891	13.576759	15.226422	12.866609	13.23783	15.619569	17.255051	-0.106008	3.0469493	0.5189335	0.8796851	3.1723997	3.0469493	5.84E-147	2.77E-144	3.3180365	3.0469493	1.56E-160	2.18E-158
13824	ENSMUSG00	231287	Atp10d	2	polymorphic	MGI Symbol	6	ATPase, clas	236	104	184	97	117	92	357	532	381	320	500	596	4.1931579	1.8065449	3.2710201	1.5945247	1.9151898	1.6130188	4.8517546	6.4700131	5.5455129	4.880306	6.755869	8.5557492	-0.851475	2.1355203	4.02E-10	2.77E-08	0.867764	2.1355203	1.04E-13	2.15E-12	1.1269878	2.1355203	1.69E-22	1.29E-21
13900	ENSMUSG00	NA	Gm9817	2	TEC	MGI Symbol	1	predicted ge	85.49	127.54	112.47	184.82	153.58	157.29	253.27	266.5	239.41	264.24	422.51	168.99	1.5189537	2.2154494	1.9994111	3.038145	2.513973	2.7577361	3.4420277	3.2410874	3.484649	4.0299127	5.7088444	2.4258994	0.533745	1.6320338	9.13E-05	0.002485	0.8234344	1.6320338	2.28E-10	3.36E-09	1.0878128	1.6320338	2.53E-17	1.37E-16
14070	ENSMUSG00	NA	Rpsa-ps10	2	processed p	MGI Symbol	1	ribosomal pr	18649.06	22871.02	25529	826.84	815.14	769.46	21108.5	31777.96	20531.28	21685.62	29244.08	24933.58	331.34938	397.28388	453.83627	13.591926	13.343143	13.490798	286.87188	386.47334	298.8359	330.72644	395.13835	357.92862	-4.870169	8.0944595	0	0	-0.282416	8.0944595	0.0016963	0.0060651	-0.125678	8.0944595	0.1622308	0.1810611
14149	ENSMUSG00	16491	Kcna3	2	protein codi	MGI Symbol	1	potassium v	68	73	133	61	66	63	237	304	209	352	418	343	1.208198	1.2680555	2.3643787	1.0027424	1.0803635	1.1045672	3.2209127	3.6971503	3.0420268	5.3683366	5.6479065	4.9238624	-0.60096	1.5495301	0.00024	0.0059067	1.0452994	1.5495301	6.40E-15	1.45E-13	1.720246	1.5495301	1.04E-40	1.75E-39
14327	ENSMUSG00	16002	Igf2	2	protein codi	MGI Symbol	9	insulin-like g	2190	1163	2418	674	703	718	3865	5863	5946.38	3937	4279	4661	38.911084	20.202035	42.985471	11.079481	11.507508	12.588559	52.526699	71.303922	86.550465	60.043015	57.816727	66.909979	-1.53724	5.4763096	3.88E-56	1.68E-53	1.0431375	5.4763096	3.19E-29	1.54E-27	0.8560577	5.4763096	3.13E-20	2.07E-19
14450	ENSMUSG00	70713	Gpr137c	2	protein codi	MGI Symbol	7	G protein-co	294.51	233.56	283.14	275.3	280.95	288.82	802.02	897.17	728.26	324.05	388.06	371.98	5.2327412	4.0570829	5.03346	4.5254914	4.5989108	5.0638269	10.899732	10.911093	10.599935	4.9420724	5.243365	5.3398785	-0.013877	2.6927377	0.9150727	0.9914851	1.1786363	2.6927377	9.82E-29	4.69E-27	0.1174077	2.6927377	0.3027148	0.3270917
14495	ENSMUSG00	16527	Kcnk3	2	protein codi	MGI Symbol	2	potassium cl	69.31	82.75	82.2	106.19	86.83	110.14	2189.8	2597.5	1959.39	2194.3	2424.01	1831.62	1.2314736	1.4374191	1.4612927	1.7455936	1.4213327	1.931064	29.760146	31.58996	28.519219	33.465173	32.752588	26.293425	0.3001091	4.0195371	0.0534831	0.3679138	4.441653	4.0195371	1.92E-281	3.04E-278	4.4829943	4.0195371	1.31E-285	6.22E-283
14675	ENSMUSG00	15270	H2ax	2	protein codi	MGI Symbol	1	H2A.X variar	686	593	670	684	629.2	707	2619.17	2910	2058	3619	4036	3620	12.188586	10.30078	11.91078	11.243865	10.299465	12.395698	35.595434	35.390485	29.954503	55.193211	54.533374	51.966128	-0.019879	4.794513	0.8506104	0.9820332	1.553509	4.794513	2.23E-57	2.75E-55	2.2327891	4.794513	3.39E-115	2.54E-113
1471	ENSMUSG00	63873	Trpv4	2	protein codi	MGI Symbol	8	transient rec	159	160	133	172	146	174	553	647	420	416	446	463	2.8250513	2.7792998	2.3643787	2.8274047	2.3898949	3.0507094	7.515463	7.8686061	6.1131639	6.3443978	6.0262351	6.6464965	0.0510262	2.275871	0.7061285	0.9442878	1.4332568	2.275871	1.54E-34	9.11E-33	1.253564	2.275871	2.36E-26	2.25E-25
14773	ENSMUSG00	57265	Fzd2	2	protein codi	MGI Symbol	1	frizzled class	489	363	308	425	328	374	1298	1318	1015	779	753	644	8.6883863	6.3055364	5.4754033	6.9863197	5.369079	6.5572719	17.640273	16.029093	14.773479	11.880495	10.174339	9.2448029	-0.114321	3.327874	0.2959075	0.7586157	1.2431452	3.327874	1.72E-34	1.01E-32	0.6118497	3.327874	3.54E-09	8.91E-09
1484	ENSMUSG00	83675	Bicc1	2	protein codi	MGI Symbol	4	BicC family F	138	147	160	129	123	121	431	456	404	570	601	522	2.4519313	2.5534817	2.8443653	2.1205535	2.0134046	2.1214703	5.8574404	5.5457255	5.8802814	8.6930451	8.1205545	7.4934582	-0.327442	2.2479663	0.0160889	0.1750795	1.137486	2.2479663	9.60E-22	3.41E-20	1.6295763	2.2479663	2.77E-44	5.24E-43
14911	ENSMUSG00	319922	Vwc2	2	protein codi	MGI Symbol	4	von Willebra	1	2	0	0	0	0	523	530	402	377	433	357	0.0177676	0.0347412	0	0	0	0	7.1077525	6.4456897	5.8511711	5.7496105	5.8505825	5.1248364	-3.202869	1.6771219	0.2507219	0.724754	8.3722959	1.6771219	4.70E-200	3.72E-197	8.1517077	1.6771219	2.67E-179	4.60E-177
14974	ENSMUSG00	320452	P4ha3	2	protein codi	MGI Symbol	5	procollagen-	375	363	368	348	353	354	4608	4627.03	3527	3679	4411	3809	6.6628568	6.3055364	6.5420403	5.720563	5.7783076	6.2066156	62.624328	56.272452	51.336021	56.108268	59.600276	54.679277	-0.140438	4.9877712	0.2024476	0.674352	3.1250362	4.9877712	3.70E-207	3.51E-204	3.1262959	4.9877712	2.62E-207	5.78E-205
15	ENSMUSG00	18053	Ngfr	2	protein codi	MGI Symbol	1	nerve growtl	1455	1553	1531	1603	1652	1561	4417	5134	3955	5830	7102	5855	25.851885	26.976579	27.217021	26.350754	27.041825	27.36872	60.028571	62.438059	57.565627	88.913075	95.960363	84.050188	0.0127815	5.6714094	0.8946788	0.988253	1.1694243	5.6714094	9.36E-36	5.93E-34	1.7482781	5.6714094	1.87E-76	6.98E-75
15111	ENSMUSG00	57775	Usp29	2	protein codi	MGI Symbol	9	ubiquitin spe	92	90	98	78	78	89	374	389.54	342	468.84	514	535	1.6346209	1.5633561	1.7421738	1.2821952	1.2767932	1.5604203	5.0827905	4.7374603	4.977862	7.1502584	6.9450333	7.6800769	-0.264329	1.9718816	0.0948156	0.4835194	1.5812809	1.9718816	9.97E-35	6.00E-33	2.1388954	1.9718816	5.30E-65	1.60E-63
15183	ENSMUSG00	17294	Mest	2	protein codi	MGI Symbol	11	mesoderm s	1461.14	1195.08	1386.89	1911.23	1937.15	1761.25	3921.67	3338.88	2997.14	4687.37	3929.1	3192.28	25.960978	20.759285	24.655136	31.417562	31.709486	30.879666	53.296864	40.606386	43.623829	71.486875	53.08897	45.826086	0.3975042	5.3053852	2.62E-05	0.0008554	0.9461168	5.3053852	7.37E-24	2.89E-22	1.2551294	5.3053852	1.53E-40	2.57E-39
15296	ENSMUSG00	226025	Trpm3	2	protein codi	MGI Symbol	24	transient rec	13726.12	11928.46	14382.55	4808.22	5515.07	5127.78	27461.35	34020.96	26090.58	37835.88	43207.72	42899	243.88046	207.20479	255.68267	79.039441	90.276971	89.904406	373.20933	413.75199	379.75236	577.03335	583.81139	615.82733	-1.447054	8.3484441	1.79E-55	7.39E-53	0.7231787	8.3484441	1.29E-15	3.11E-14	1.3298802	8.3484441	6.39E-48	1.30E-46

Table S2. Differentially expressed genes (> 2-fold) in *Trpm3*-mutant lenses summarized in Fig. 9A.

15327	ENSMUSG00	268902	Robo2	2	protein codi	MGI Symbol	13	roundabout	73	62	67	62	84	88	281	376	287	341	429	482	1.2970361	1.0769787	1.191078	1.0191808	1.375008	1.5428875	3.818888	4.5727912	4.1773287	5.2005761	5.7965356	6.9192469	0.1400089	1.715625	0.3987401	0.8286486	1.8186916	1.715625	8.13E-40	5.94E-38	2.3289474	1.715625	2.01E-67	6.28E-66
1565	ENSMUSG00	233328	Lrrk1	2	protein codi	MGI Symbol	7	leucine-rich	133	132	103	153	135	151	245	321	251	296	359	339	2.3630932	2.2929223	1.8310602	2.5150751	2.2098343	2.6474547	3.3296355	3.9038989	3.6533432	4.5142831	4.8507139	4.8664413	0.1825736	1.7319437	0.1776233	0.6367629	0.7475031	1.7319437	4.07E-09	5.24E-08	1.1330278	1.7319437	8.20E-20	5.27E-19
1566	ENSMUSG00	56847	Aldh1a3	2	protein codi	MGI Symbol	5	aldehyde de	63	52	53	61	63	60	294	278	229	337	342	370	1.1193599	0.9032724	0.942196	1.0027424	1.031256	1.0519688	3.9955626	3.3809467	3.3331298	5.1395723	4.6210144	5.3114551	0.0578374	1.4660228	0.7819779	0.9653561	1.8513494	1.4660228	1.51E-37	1.06E-35	2.3432468	1.4660228	1.65E-62	4.83E-61
15689	ENSMUSG00	212073	Syne3	2	protein codi	MGI Symbol	3	spectrin rep	195	183	203	206	247	234	394	506	398	570	556	499	3.4646856	3.1788241	3.6087885	3.3863103	4.0431784	4.1026781	5.3545975	6.1538094	5.7929505	8.6930451	7.5125263	7.1632867	0.169127	2.4021012	0.1628791	0.616065	0.7559398	2.4021012	4.91E-11	7.78E-10	1.1874602	2.4021012	7.78E-26	7.13E-25
15694	ENSMUSG00	26366	Ceacam10	2	protein codi	MGI Symbol	4	carcinoembr	1044	1117	1317	1800	1679	1540	3589	3911	3378.99	2729.99	2644.99	2240.98	18.549393	19.402987	23.412682	29.589119	27.483791	27.000531	48.775762	47.564326	49.181713	41.634958	35.738412	32.169904	0.4543726	5.0654953	1.83E-06	7.57E-05	1.2456782	5.0654953	1.33E-39	9.64E-38	0.8357221	5.0654953	1.01E-18	6.03E-18
1572	ENSMUSG00	215748	Cnksr3	2	protein codi	MGI Symbol	2	Cnksr family	260	229	235	255	245	235	720	804	631	607	750	714	4.6195807	3.9778728	4.1776616	4.1917918	4.0104401	4.1202109	9.7850512	9.7779897	9.184301	9.2573305	10.133803	10.249673	-0.051784	2.8218056	0.6608263	0.9306923	1.1705126	2.8218056	5.43E-27	2.40E-25	1.2146861	2.8218056	7.08E-29	7.57E-28
15733	ENSMUSG00	26367	Ceacam2	2	protein codi	MGI Symbol	4	carcinoembr	2062.22	1997.46	2019.39	2926.41	3056.13	2922.59	5425.4	5797.83	6403.7	7655.55	6649.22	5664.88	36.640738	34.697126	35.899268	48.105496	50.02623	51.241223	73.733079	70.511346	93.206827	116.75446	89.842518	81.320961	0.4780652	6.0285149	2.66E-07	1.26E-05	1.1464757	6.0285149	5.71E-35	3.46E-33	1.4246189	6.0285149	1.31E-52	3.02E-51
15779	ENSMUSG00	11489	Adam12	2	protein codi	MGI Symbol	7	a disintegrin	1279.98	1158.26	1164.66	1421.79	1360.14	1307.9	2577.33	2638.16	2107.74	2918.43	3214.92	2992.61	22.742196	20.119699	20.704491	23.371952	22.264326	22.931166	35.026814	32.084454	30.678476	44.508848	43.439157	42.959766	0.1092996	4.9147486	0.253954	0.7286507	0.6215225	4.9147486	4.72E-11	7.49E-10	1.0421745	4.9147486	2.65E-28	2.77E-27
16003	ENSMUSG00	13134	Dach1	2	protein codi	MGI Symbol	3	dachshund f	143	112	125	88	80	65	269	336	273	301	342	407	2.5407694	1.9455099	2.2221604	1.4465791	1.3095315	1.1396328	3.6558039	4.0863241	3.9735565	4.5905378	4.6210144	5.8426006	-0.779654	1.680197	1.78E-07	8.63E-06	0.8053765	1.680197	1.53E-10	2.30E-09	1.1662939	1.680197	2.85E-21	2.01E-20
16126	ENSMUSG00	329941	Col8a2	2	protein codi	MGI Symbol	2	collagen, typ	79	61	59	54	57	74	2226	2820	2329	1885	1430	1429	1.4036418	1.059608	1.0488597	0.8876736	0.9330412	1.2974281	30.252117	34.259934	33.898949	28.748053	19.321785	20.513701	-0.176381	3.8804702	0.3382774	0.7877514	4.807485	3.8804702	3.01901067	1.43403006	4.2846837	3.8804702	8.07E-249	2.84E-246
16140	ENSMUSG00	20745	Spock1	2	protein codi	MGI Symbol	6	sparc/osteo	1486	1146	1472	1490	1598	1525	2867	3310	2769	3477	3707	3380	26.402681	19.906735	26.168161	24.493215	26.157891	26.737539	38.96353	40.255157	40.303216	53.027575	50.088013	48.52086	0.0946827	5.1369051	0.3197823	0.7756777	0.7218475	5.1369051	1.50E-14	3.33E-13	1.0650838	5.1369051	9.28E-30	1.04E-28
16182	ENSMUSG00	20564	Slit3	2	protein codi	MGI Symbol	4	slit guidance	3862	3243	3728	3887	3986	4169	7591	9228	7644	8944	10207	10334	68.618542	56.332933	66.273712	63.896058	65.247405	73.094295	103.16434	112.22797	111.25958	136.40455	137.91431	148.3475	0.0807235	6.5750428	0.3787705	0.8157604	0.7725387	6.5750428	3.24E-17	8.72E-16	1.1442864	6.5750428	1.62E-35	2.29E-34
16249	ENSMUSG00	237806	Dnah9	2	protein codi	MGI Symbol	6	dynein, axon	115	89	211	123	130	121	421	485	288	503	544	672	2.0432761	1.5459855	3.7510068	2.0219231	2.1279886	2.1214703	5.7215369	5.8984142	4.1918838	7.671231	7.3503854	9.6467508	-0.22478	2.2077214	0.1017917	0.5023488	1.1127965	2.2077214	3.41E-20	1.12E-18	1.7499935	2.2077214	5.94E-50	1.28E-48
1631	ENSMUSG00	74051	Steap2	2	protein codi	MGI Symbol	9	six transmer	215	151	186	180	194	258	507	484	341	534	511	557	3.8200379	2.6229642	3.3065747	2.9589119	3.1756138	4.5234656	6.8903069	5.8862525	4.9633069	8.1440107	6.9044981	7.9958932	0.1255436	2.3743364	0.311673	0.7696817	0.8656745	2.3743364	6.52E-14	1.37E-12	1.2403715	2.3743364	1.14E-27	1.16E-26
16406	ENSMUSG00	75739	Mpp7	2	protein codi	MGI Symbol	14	membrane p	159	147	95	166	155	174	307	299	253	331	363	330	2.8250513	2.5534817	1.6888419	2.7287743	2.5372172	3.0507094	4.1722371	3.6363419	3.6824535	5.0480665	4.9047609	4.7372437	0.2324041	1.8205176	0.0815847	0.4548443	0.6997352	1.8205176	1.78E-08	2.13E-07	1.0541218	1.8205176	7.22E-18	4.06E-17
16470	ENSMUSG00	230971	Megf6	2	protein codi	MGI Symbol	7	multiple EGF	1015	903	847	1116	996	1051	3826	3793	3405	2180	2153	1995	18.034132	15.685673	15.057359	18.345254	16.303667	18.426986	51.996675	46.129247	49.560293	33.247085	29.090772	28.638792	0.121688	4.8339234	0.2118523	0.6880673	1.5980456	4.8339234	1.36E-62	1.83E-60	0.898961	4.8339234	5.62E-21	3.89E-20
16520	ENSMUSG00	67935	Ces5a	2	protein codi	MGI Symbol	6	carboxyleste	114	180	115	106	106	129	298	336	282	376	390	354	2.0255085	3.1267123	2.0443876	1.7424703	1.7351292	2.2617328	4.049924	4.0863241	4.1045529	5.7343596	5.2695778	5.0817705	-0.331481	1.8161159	0.0193876	0.1982582	0.7639306	1.8161159	5.61E-10	8.04E-09	1.1570658	1.8161159	1.29E-21	9.37E-21
16549	ENSMUSG00	56747	Sez6l	2	protein codi	MGI Symbol	6	seizure relat	426	377	439	432	449	516	975	1475	972	1420	962	792	7.5690054	6.5487251	7.8042274	7.1013885	7.3497453	9.0469313	13.25059	17.938476	14.147608	21.656358	12.998292	11.369385	0.0991333	3.523871	0.351563	0.7957545	1.0500215	3.523871	3.59E-25	1.48E-23	1.0678264	3.523871	5.88E-26	5.47E-25
1678	ENSMUSG00	80909	Castor2	2	protein codi	MGI Symbol	3	cytosolic arg	518	479	450	510	498	559	1494	1739	1420	2454	2498	2142	9.2036262	8.3205288	7.9997775	8.3835837	8.1518333	9.8008422	20.303981	21.149159	20.668316	37.425847	33.752321	30.749018	0.0443096	4.1807736	0.6733839	0.9358026	1.2832276	4.1807736	4.25E-38	3.03E-36	1.9972046	4.1807736	3.12E-90	1.38E-88
16892	ENSMUSG00	16682	Krt4	2	protein codi	MGI Symbol	1	keratin 4 [So	28	55	39	37	28	48	910	928	679	503	823	494	0.4974933	0.9553843	0.6933141	0.6082208	0.458336	0.841575	12.367218	11.286038	9.8829483	7.671231	11.12016	7.0915103	-0.180653	2.4585248	0.445066	0.8510722	3.9600957	2.4585248	4.20E-176	2.50E-173	3.5881203	2.4585248	1.26E-139	1.39E-137
17019	ENSMUSG00	242466	Zfp462	2	protein codi	MGI Symbol	4	zinc finger p	144	129	166	139	171	169	269	395	322	392	434	440	2.558537	2.2408105	2.951029	2.2849375	2.7991235	2.9630453	3.6558039	4.8038631	4.686759	5.9783749	5.8640943	6.3163249	0.0533216	2.0020739	0.7007654	0.9430688	0.7636464	2.0020739	3.48E-10	5.05E-09	1.2283427	2.0020739	5.22E-25	4.55E-24
17104	ENSMUSG00	13808	Eno3	2	protein codi	MGI Symbol	8	enolase 3, b	5429	5262	5853	5363	5362	5426	10564	12984	9675	11590	15945	14095	96.460399	91.404222	104.05044	88.159136	87.771346	95.133041	143.56845	157.90724	140.8211	176.75858	215.44466	202.33773	-0.106948	7.0599191	0.2403522	0.7199863	0.5995575	7.0599191	4.27E-11	6.81E-10	1.0263038	7.0599191	2.89E-29	3.15E-28
17131	ENSMUSG00	211945	Plekhh1	2	protein codi	MGI Symbol	4	pleckstrin ho	345.98	332.58	340.59	473.05	380.29	446.63	838.55	797.52	841.56	1201.5	1148.94	1035.07	6.1472406	5.777122	6.0547649	7.7761848	6.2250215	7.8306801	11.396187	9.699182	12.249034	18.324024	15.524176	14.858724	0.2793969	3.3575272	0.00988	0.1244348	0.8892991	3.3575272	1.15E-17	3.24E-16	1.4369627	3.3575272	1.33E-44	2.53E-43
17219	ENSMUSG00	NA	Rpl15-ps3	2	processed p	MGI Symbol	1	ribosomal pr	1689.42	1371.02	1594.26	136.39	133.19	128.4	1992.98	2455.62	2027.54	2904.12	2685.27	1768.2	30.01697	23.815473	28.341612	2.2420333	2.1802062	2.2512131	27.085294	29.864462	29.511153	44.290607	36.282665	25.383013	-3.620988	4.5568378	5.36E-213	7.27E-210	0.0735805	4.5568378	0.43504	0.5260795	0.3663856	4.5568378	9.13E-05	0.0001411
17273	ENSMUSG00	380698	Obscn	2	protein codi	MGI Symbol	10	obscurin, cy	121	77	88.33	98	95	72	239	340	233	273	305	274.98	2.1498818	1.337538	1.5702674	1.6109631	1.5550686	1.2623625	3.2480934	4.1349708	3.3913504	4.1635111	4.1210801	3.947416	-0.186974	1.4843638	0.2286575	0.7091949	1.0954875	1.4843638	1.12E-16	2.91E-15	1.2744382	1.4843638	2.40E-22	1.82E-21
17291	ENSMUSG00	75216	Cep128	2	protein codi	MGI Symbol	13	centrosomal	250	150	250	76	60	69	232	262	286	191	327	358	4.4419046	2.6055936	4.4443208	1.2493184	0.9821486	1.2097641	3.152961	3.1863598	4.1627735	2.9129327	4.4183383	5.1391917	-1.737577	1.6830555	2.23E-34	7.85E-32	-0.132717	1.6830555	0.2715673	0.3611268	0.1233552	1.6830555	0.300252	0.3245414
17424	ENSMUSG00	212986	Scfd2	2	protein codi	MGI Symbol	9	Sec1 family	735	581	733	228	252	229	939	1248	974	957	926	827	13.059199	10.092332	13.030749	3.7479551	4.1250241	4.0150141	12.761338	15.177775	14.176718	14.595165	12.511869	11.87182	-1.60503	3.4386259	1.14E-48	4.32E-46	0.2199572	3.4386259	0.0261949	0.0554853	0.1069952	3.4386259	0.2817985	0.3055685
17487	ENSMUSG00	NA	Gm15772	2	processed p	MGI Symbol	1	predicted ge	2086.15	235.61	10866.44	263.32	223.65	208.78	4454.87	14540.55	4515.21	255.45	3223.31	4893.75	37.065917	4.0926927	193.17578	4.3285593	3.6609589	3.6605006	60.543238	176.8375	65.719568	3.8958568	43.55252	70.251171	-4.328294	5.7996753	0	0	0.372753	5.7996753	4.25E-05	0.0002572	-0.991238	5.7996753	8.17E-27	7.94E-26
1753	ENSMUSG00	240725	Sulf1	2	protein codi	MGI Symbol	10	sulfatase 1 [	2299	2150	2591	2537	2455	2461	7913	8799	7137	5532	6603	6162	40.847754	37.346841	46.060941	41.704219	40.186247	43.148252	107.54043	107.01061	103.88012	84.36829	89.218006	88.45726	0.0090629	6.1146587	0.9238548	0.9930291	1.3577308	6.1146587	2.40E-48	2.26E-46	1.0765804	6.1146587	3.05E-31	3.65E-30
17817	ENSMUSG00	380669	Lin28b	2	protein codi	MGI Symbol	3	lin-28 homo	112	102	116	113	136	127	305	337	363	411	417	447	1.9899732	1.7718036	2.0621649	1.8575391	2.2262035	2.2266672	4.1450564	4.0984857	5.2835202	6.268143	5.6343947	6.4168119	0.115224	1.9102716	0.4331284	0.8466385	1.2119768	1.9102716	7.03E-22	2.55E-20	1.6520203	1.9102716	1.67E-40	2.79E-39
1809	ENSMUSG00	56745	C1qtnf1	2	protein codi	MGI Symbol	4	C1q and tum	862.87	871.77	554.77	94.72	100	93.8	1274.86	1329.74	1048	1606	1993.87	1679	15.331145	15.143189	9.8623035	1.5570452	1.6369143	1.6445778	17.325792	16.171871	15.253799	24.493036	26.940649	24.102522	-3.058394	3.8322352	4.83E-137	3.53E-134	0.273284	3.8322352	0.005122	0.0149859	0.9049641	3.8322352	8.36E-21	5.71E-20
1824	ENSMUSG00	268451	Rab11fip4	2	protein codi	MGI Symbol	2	RAB11 famil	373	360	274	376	335	353	1012	990	702	772	885	764	6.6273216	6.2534245	4.8709756	6.1808382	5.483663	6.1890828	13.753433	12.040062	10.217717	11.773738	11.957888	10.967437	0.0075668	3.1643716	0.9525917	1	1.0208722	3.1643716	7.15E-23	2.68E-21	0.9665538	3.1643716	1.65E-20	1.11E-19
1828	ENSMUSG00	140579	Elmo2	2	protein codi	MGI Symbol	12	engulfment	979	955	969	1005	966	987	2056	2408	1899	2132	2676	2362	17.394498	16.588946	17.226188	16.520591	15.812592	17.304886	27.941757	29.285322	27.640234	32.515039	36.157411	33.907181	-0.045143	4.5927319	0.6465556	0.9270796	0.728974	4.5927319	2.19E-14	4.83E-13	1.0024298	4.5927319	7.92E-26	7.24E-25
1899	ENSMUSG00	18858	Pmp22	2	protein codi	MGI Symbol	5	peripheral m	7338	7184	6432	8022	8083	7762	16011	15862	12895	16560	18317	17615	130.37878	124.79056	114.34349	131.86884	132.31178	136.08969	217.59508	192.90855	187.68869	252.55584	247.4945	252.86833	0.1153306	7.466332	0.2033824	0.6750986	0.6949766	7.466332	1.93E-14	4.26E-13	1.0268482	7.466332	2.08E-29	2.30E-28
19057	ENSMUSG00	NA	Gm6969	2	processed p	MGI Symbol	1	predicted ps	713.82	385.42	441.55	156.68	175.63	158.32	631.16	751.12	748.22	830.02	1032.99	866.28	12.682881	6.6949858	7.8495595	2.5755684	2.8749126	2.7757949	8.5776846	9.1348801	10.890456	12.658599	13.95749	12.435695	-1.725399	3.1163809	3.50E-50	1.39E-47	0.0704265	3.1163809	0.4900662	0.5789143	0.5214008	3.1163809	2.31E-07	4.72E-07
19286	ENSMUSG00	98932	Myl9	2	protein codi	MGI Symbol	4	myosin, light	511	432	475	469	491	437	2332	2487	1656	2505	3830	3584	9.0792529	7.5041095	8.4442096	7.7096093	8.0372493	7.6618391	31.692694	30.246095	24.103332	38.203646	51.749956	51.449338	-0.095986	4.5348738	0.3636229	0.8050619	1.7822048	4.5348738	3.79E-72	6.20E-70	2.4986556	4.5348738	8.52E-139	9.30E-137
19922	ENSMUSG00	56222	Cited4	2	protein codi	MGI Symbol	1	Cbp/p300-in	261	223	245	282	251	258	697	768	609	776	963	798	4.6373484	3.8736491	4.3554344	4.6356286	4.1086549	4.5234656	9.4724732	9.3401693	8.8640876	11.834742	13.011804	11.455517	0.0443908	2.930517	0.7191741	0.9477493	1.1055548	2.930517	3.00E-24	1.19E-22	1.4969256	2.930517	1.23E-43	2.28E-42
1996	ENSMUSG00	228033	Atp5g3	2	protein codi	MGI Symbol	5	ATP synthase	2611	2460	2703	2798	2890	2590	5383	5805	4887	5849	7104	6597	46.391251	42.731734	48.051997	45.994641	47.306824	45.409984	73.156848	70.598545	71.131028	89.202843	95.987386	94.701808	0.0161497	6.0073105	0.8631912	0.9821164	0.6475648	6.0073105	2.01E-12	3.73E-11	1.0289217	6.0073105	8.03E-29	8.58E-28
2022	ENSMUSG00	18452	P4ha2	2	protein codi	MGI Symbol	15	procollagen-	2175	1977	1660	2054	2057	2061	4398	5181	3832	4532	5276	4946	38.64457	34.341723	29.51029	33.764472	33.671328	36.135127	59.770355	63.009657	55.775343	69.117334	71.287929	71.001235	0.0149177	5.6375411	0.8745768	0.9848106	0.8009483	5.6375411	6.09E-18	1.77E-16	1.0444241	5.6375411	2.85E-29	3.11E-28
20262	ENSMUSG00	NA	Gm10382	2	antisense	MGI Symbol	2	predicted ge	126.01	113.83	193.44	207.86	214.27	212.53	327.21	570.82	339.76	298.92	388.77	344.15	2.2388976	1.9772981	3.4388377	3.4168857	3.5074163	3.7262486	4.4468981	6.9421295	4.9452585	4.5588159	5.2529584	4.940371	0.4767849	2.0710783	0.0001702	0.0043222	1.0994423	2.0710783	3.96E-20	1.29E-18	0.9486654	2.0710783	5.92E-15	2.63E-14
2031	ENSMUSG00	11687	Alox15	2	protein codi	MGI Symbol	2	arachidonate	1060	1037	922	2015	2083	2012	2471	3267	2515	2888	4188	2519	18.833675	18.013337	16.390655	33.123375	34.096925	35.276019	33.581752	39.732204	36.606208	44.044762	56.587159	36.16096	0.944862	5.071955	4.28E-23	8.66E-21	1.0460907	5.071955	3.87E-28	1.81E-26	1.3617541	5.071955	2.36E-46	4.67E-45
2047	ENSMUSG00	18822	Plod1	2	protein codi	MGI Symbol	6	procollagen-	1439	1294	1441	1464	1593	1518	2729	3077	2638	3425	3707	3375	25.567603	22.477587	25.617065	24.065817	26.076045	26.614809	37.088062	37.421485	38.396491	52.234525	50.088013	48.449083	0.0593397	5.1128152	0.5333857	0.8849579	0.6161315	5.1128152	5.21E-11	8.21E-10	1.0333506	5.1128152	3.77E-28	3.91E-27
20544	ENSMUSG00	69368	Wdfy1	2	protein codi	MGI Symbol	14	WD repeat a	475	511	437	102	135	136	779	661	787.04	1033	1012	1017	8.4396187	8.8763887	7.7686728	1.6767167	2.2098343	2.3844625	10.586882	8.0388696	11.455487	15.754238	13.673879	14.599324	-2.00157	3.1507728	1.14E-61	5.43E-59	0.2589656	3.1507728	0.0109239	0.0275796	0.8106681	3.1507728	8.08E-16	3.88E-15
20808	ENSMUSG00	NA	Gm6166	2	processed p	MGI Symbol	1	predicted ge	80735.8	90539.46	85026.56	3564.45	3830.17	3093.67	87359.33	85550.41	75362.61	80038.74	91755.77	90842.79	1434.4829	1572.7269	1511.5412	58.593853	62.696601	54.240736	1187.2438	1040.4366	1096.9143	1220.6673	1239.7799	1304.074	-4.68596	9.9395903	0	0	-0.442752	9.9395903	8.32E-07	7.61E-06	-0.263457	9.9395903	0.0033249	0.0043852
2096	ENSMUSG00	19216	Ptger1	2	protein codi	MGI Symbol	3	prostaglandi	110.38	123.75	172.91	169.39	220.28	158.29	854.92	1085.14	833.62	520.57	522.51	405.01	1.9611897	2.1496147	3.0738701	2.7845005	3.6057949	2.7752689	11.618661	13.197124	12.133466	7.9391903	7.0600182	5.8140336	0.3532445	2.6627489	0.0070033	0.0967032	2.3629613	2.6627489	1.63E-92	3.70E-90	1.533402	2.6627489	7.06E-38	1.09E-36
20979	ENSMUSG00	21824	Thbd	2	protein codi	MGI Symbol	1	thrombomo	35	19	30	43	40	43	2482	2875	2169	3296	3286	2695	0.6218666	0.3300419	0.5333185	0.7068512	0.6547657	0.7539109	33.731246	34.964826	31.570125	50.267152	44.399571	38.68749	0.5097839	4.3240792	0.0227626	0.2202174	6.0749736	4.3240792	0	0	6.4856745	4.3240792	0	0
21638	ENSMUSG00	NA	Igkc	2	IG C gene	MGI Symbol	1	immunoglob	107	124	128	39	31	58	582	618	533	354	326	368	1.9011351	2.1539573	2.2754923	0.6410976	0.5074434	1.0169031	7.9095831	7.5159175	7.7578961	5.3988385	4.4048266	5.2827445	-1.555553	2.0128817	2.82E-20	4.78E-18	1.8712553	2.0128817	1.04E-54	1.18E-52	1.2500346	2.0128817	8.38E-24	6.80E-23
22392	ENSMUSG00	67122	Nrarp	2	protein codi	MGI Symbol	1	Notch-regul	144	104	103	160.24	114.87	156.39	335.33	270.92	303	375.61	385.63	365.8	2.558537	1.8065449	1.8310602	2.6340891	1.8803235	2.7419566	4.5572517	3.294842	4.4102111	5.7284117	5.2105315	5.2511629	0.2259493	1.8361052	0.0993097	0.4939124	0.9802531	1.8361052	7.38E-15	1.65E-13	1.3847295	1.8361052	5.50E-29	5.93E-28
22442	ENSMUSG00	NA	Gm4294	2	processed p	MGI Symbol	1	predicted ge	2679.74	2256.84	2789.68	348.07	420.81	524.49	3704.5	3495.82	3316.64	2799.62	3858.78	2983.29	47.612597	39.202718	49.592932	5.7217137	6.8882992	9.1957849	50.345448	42.51504	48.2742	42.696882	52.138824	42.825975	-2.647084	5.1899337	4.92E-153	4.67E-150	0.04896	5.1899337	0.5957331	0.6760799	0.0135959	5.1899337	0.8859323	0.8951666
226	ENSMUSG00	12709	Ckb	2	protein codi	MGI Symbol	5	creatine kina	3923.14	3851.24	4314.3	4614.43	4624.26	4328.5	9822.03	11365.08	9614	11843.19	15189.77	13418.04	69.704854	66.898441	76.696534	75.853843	75.695174	75.890779	133.48481	138.21845	139.93323	180.61997	205.24019	192.61977	0.0926251	6.8992908	0.3114551	0.7696817	0.9484916	6.8992908	3.91E-25	1.61E-23	1.4394333	6.8992908	9.59E-55	2.37E-53
22668	ENSMUSG00	NA	Hsp25-ps1	2	processed p	MGI Symbol	1	heat shock p	5826.87	6162.7	6039.53	9004.14	7798.74	8162.45	16061.71	15247.93	13939.5	16958.04	20032.26	10314.19	103.5296	107.04994	107.36644	148.01365	127.65869	143.11071	218.28424	185.44043	202.89154	258.62633	270.67065	148.06313	0.3973918	7.396588	1.21E-05	0.0004152	0.9319376	7.396588	1.57E-24	6.35E-23	1.0911378	7.396588	7.58E-33	9.56E-32
22722	ENSMUSG00	628900	Serpina3i	2	protein codi	MGI Symbol	2	serine (or cy	185.46	141.53	298.77	0	0	0	289.71	379.74	447.83	3	410.96	329.46	3.2951825	2.4584644	5.3113189	0	0	0	3.93726	4.6182759	6.5182338	0.0457529	5.5527838	4.7294919	-10.74449	1.6462004	1.27E-138	1.01E-135	0.4430298	1.6462004	0.0001146	0.0006197	-0.078449	1.6462004	0.483602	0.5092804
2380	ENSMUSG00	21664	Phlda1	2	protein codi	MGI Symbol	2	pleckstrin ho	476.35	413	354.75	546.95	426	493.04	1126.32	1129.02	896.24	1072.46	1196.67	1082.8	8.4636049	7.1740676	6.3064913	8.9909825	6.973255	8.6443779	15.307082	13.730779	13.04491	16.356041	16.169091	15.543901	0.1648139	3.5228092	0.1197525	0.5376413	0.9394473	3.5228092	2.16E-20	7.19E-19	1.1310612	3.5228092	5.59E-29	6.01E-28
2599	ENSMUSG00	66109	Tspan13	2	protein codi	MGI Symbol	3	tetraspanin	130	102	137	114	124	122	287	325	285	334	357	349	2.3097904	1.7718036	2.4354878	1.8739775	2.0297738	2.1390031	3.9004301	3.9525456	4.1482183	5.0938194	4.8236905	5.0099941	-0.108839	1.7537375	0.4439138	0.850865	0.8808083	1.7537375	2.57E-12	4.73E-11	1.1955993	1.7537375	4.44E-22	3.32E-21
2669	ENSMUSG00	11421	Ace	2	protein codi	MGI Symbol	4	angiotensin	2129.76	2043.19	2226.23	2186.95	2210.09	2110.56	8246.21	7995.08	6635.9	7854.08	7061.24	7595.66	37.840763	35.491485	39.576321	35.949958	36.17728	37.004053	112.06887	97.233594	96.586534	119.78223	95.409625	109.03786	-0.049081	6.1531725	0.5997464	0.9102607	1.4378421	6.1531725	1.13E-53	1.22E-51	1.5217118	6.1531725	1.08E-59	2.94E-58
278	ENSMUSG00	14230	Fkbp10	2	protein codi	MGI Symbol	5	FK506 bindi	1370.35	1184.46	1502.27	1502.09	1501.58	1446.43	3224.46	3517.43	2946.29	3559.62	4345.23	4309.16	24.347856	20.574809	26.706279	24.691955	24.579578	25.359986	43.821537	42.777854	42.883699	54.287609	58.711609	61.859215	0.0593445	5.2352522	0.5344725	0.8850615	0.8542591	5.2352522	9.42E-20	3.01E-18	1.2877679	5.2352522	1.46E-42	2.60E-41
2827	ENSMUSG00	20851	Stat5b	2	protein codi	MGI Symbol	3	signal transc	2044.85	1857.21	1728.17	2211.9	2095.11	2201.84	3896.86	4409.37	3553.89	4181.45	5277.63	4794.49	36.332114	32.260896	30.722168	36.360096	34.295156	38.604448	52.959687	53.625341	51.727409	63.771111	71.309954	68.826265	0.1375412	5.5748996	0.1411933	0.5774157	0.6727431	5.5748996	4.32E-13	8.63E-12	1.0379193	5.5748996	6.96E-29	7.44E-28
302	ENSMUSG00	107022	Gramd3	2	protein codi	MGI Symbol	5	GRAM doma	419	411	390	514	503	484	1043	1168	992	1147	1334	1178	7.444632	7.1393264	6.9331405	8.4493373	8.233679	8.4858813	14.174734	14.204841	14.438711	17.492847	18.024658	16.910525	0.2260066	3.5770733	0.032788	0.2811416	0.9924757	3.5770733	1.50E-22	5.54E-21	1.2847402	3.5770733	5.34E-37	8.03E-36
3066	ENSMUSG00	18003	Nedd9	2	protein codi	MGI Symbol	6	neural precu	504	447	512	554	562	513	1357	1634	1182	1463	1498	1527	8.9548796	7.7646688	9.1019691	9.1068733	9.1994585	8.9943328	18.442103	19.872183	17.20419	22.312149	20.240583	21.920519	0.0807175	3.8626876	0.4421512	0.850865	1.1051983	3.8626876	1.38E-28	6.54E-27	1.3199207	3.8626876	3.36E-40	5.55E-39
30755	ENSMUSG00	NA	Gm10171	2	processed p	MGI Symbol	1	predicted ge	137.76	39.78	249.85	383.3	268.02	150.59	346.13	267.58	489.13	439.31	273.04	406.1	2.4476671	0.6910034	4.4416542	6.3008385	4.3872578	2.6402662	4.7040275	3.254222	7.1193615	6.6998976	3.6892449	5.8296808	0.8230234	2.1394854	4.04E-11	3.12E-09	0.9856274	2.1394854	2.10E-16	5.38E-15	1.0960596	2.1394854	8.36E-20	5.37E-19
3281	ENSMUSG00	268709	Fam107a	2	protein codi	MGI Symbol	4	family with	26	21	33	20	31	25	491	730	564	540	358	434	0.4619581	0.3647831	0.5866504	0.328768	0.5074434	0.4383203	6.6728613	8.8780255	8.2091058	8.2355164	4.8372022	6.2301932	-0.1468	1.9959522	0.6014728	0.9110318	4.0691661	1.9959522	1.95E-153	1.03E-150	3.763866	1.9959522	1.90E-126	1.60E-124
3316	ENSMUSG00	18074	Nid2	2	protein codi	MGI Symbol	4	nidogen 2 [S	137.08	83.03	161.08	108.03	107.02	102.04	530.3	569.7	566.68	775.18	700.67	716.36	2.4355851	1.4422829	2.8635648	1.7758403	1.7518257	1.7890482	7.206962	6.9285084	8.2481136	11.822236	9.4672694	10.283551	-0.340063	2.4946911	0.0178182	0.1864243	1.7310142	2.4946911	1.14E-47	1.05E-45	2.2276062	2.4946911	9.41E-80	3.62E-78
3329	ENSMUSG00	70561	Txndc16	2	protein codi	MGI Symbol	12	thioredoxin	333.49	271.44	328.86	285.7	314.05	311.18	775.98	877.83	660.74	680.95	840.94	846.02	5.925323	4.7150821	5.8462374	4.6964507	5.1407294	5.4558606	10.545839	10.675886	9.6171712	10.385139	11.362561	12.144857	-0.108608	3.0259645	0.3425974	0.7893553	0.9045492	3.0259645	7.80E-18	2.23E-16	1.0404218	3.0259645	3.41E-23	2.69E-22
333	ENSMUSG00	268977	Ltbp1	2	protein codi	MGI Symbol	10	latent transf	471	363	427	451	550	504	1629	2075	1707	2446	2553	2354	8.3685482	6.3055364	7.5909	7.4137181	9.0030288	8.8365375	22.138678	25.235483	24.845645	37.303839	34.495467	33.792338	0.1817603	4.2433918	0.0857046	0.4647222	1.6979617	4.2433918	1.54E-64	2.12E-62	2.2456658	4.2433918	1.56E-111	1.06E-109
3416	ENSMUSG00	219140	Spata13	2	protein codi	MGI Symbol	9	spermatoger	491	419	383	470	496	466	1464	1677	1207	1592	1829	1681	8.7239005	7.2782913	6.8086995	7.7260477	8.119095	8.1702906	19.896271	20.395135	17.568069	24.279522	24.712969	24.131232	0.0741802	3.9029528	0.4872723	0.8693576	1.3433831	3.9029528	6.66E-41	5.14E-39	1.6805309	3.9029528	2.38E-63	7.07E-62
34468	ENSMUSG00	67464	Entpd4	2	protein codi	MGI Symbol	6	ectonucleosi	245.97	414.57	439.3	408.4	424.86	364.12	854.53	754.89	871.46	939.15	924.64	1031.83	4.3703011	7.2013395	7.8095606	6.7134423	6.9545942	6.3840477	11.613361	9.1807297	12.684233	14.322936	12.493493	14.812213	0.0491442	3.2681164	0.6571852	0.9291083	0.7856191	3.2681164	2.72E-14	5.94E-13	1.1017245	3.2681164	6.55E-27	6.39E-26
3467	ENSMUSG00	21933	Tnfrsf10b	2	protein codi	MGI Symbol	3	tumor necro	390	346	293	624.94	700.47	742.91	837	952	858	988.93	964	940	6.9293711	6.0102358	5.208744	10.273013	11.466094	13.025302	11.375122	11.577918	12.488321	15.082128	13.025315	13.493967	0.9367592	3.4465295	3.50E-19	5.55E-17	0.9648964	3.4465295	1.21E-20	4.07E-19	1.1958808	3.4465295	4.08E-31	4.86E-30
35	ENSMUSG00	72014	Btbd17	2	protein codi	MGI Symbol	7	BTB (POZ) d	1851	1817	1770	2588	2413	2271	5315	5236	4824	5942	6504	5671	32.887861	31.562423	31.465792	42.542578	39.498743	39.817017	72.232705	63.67855	70.214054	90.621182	87.880344	81.408815	0.3453891	5.8356144	0.0002162	0.0053494	1.1034955	5.8356144	2.24E-32	1.20E-30	1.4380979	5.8356144	2.68E-53	6.36E-52
3505	ENSMUSG00	58187	Cldn10	2	protein codi	MGI Symbol	3	claudin 10 [S	9	10	13	6	7	5	565	633	532	351	433	356	0.1599086	0.1737062	0.2311047	0.0986304	0.114584	0.0876641	7.6785471	7.6983426	7.7433409	5.3530857	5.8505825	5.1104811	-0.889386	1.8262547	0.0370828	0.3030025	5.3421296	1.8262547	1.37E-187	8.70E-185	4.8395637	1.8262547	3.30E-142	3.73E-140
3702	ENSMUSG00	69354	Slc38a4	2	protein codi	MGI Symbol	5	solute carrie	1888	1658	2136	1865	2009	1917	5002	5100	3701	5679	6241	6158	33.545263	28.800494	37.972277	30.657615	32.885609	33.610402	67.978925	62.024562	53.868618	86.610181	84.326757	88.399839	-0.046205	5.7419042	0.6231318	0.9180952	0.8743877	5.7419042	4.84E-21	1.69E-19	1.3703012	5.7419042	9.67E-49	2.02E-47
3823	ENSMUSG00	67896	Ccdc80	2	protein codi	MGI Symbol	6	coiled-coil d	2381	2304	2534	2720	2771	2717	6496	6165	4735	6675	7296	6646	42.304699	40.021917	45.047636	44.712446	45.358896	47.636652	88.282907	74.976749	68.918645	101.80013	98.58164	95.405217	0.1125041	6.0488313	0.224923	0.7050666	0.8662363	6.0488313	6.48E-21	2.25E-19	1.2154654	6.0488313	2.88E-39	4.68E-38
39043	ENSMUSG00	NA	Gm15459	2	processed p	MGI Symbol	1	predicted ge	12417.66	8305.69	29384.93	3417.6	3264.91	3152.15	19943.48	48508.26	17894.45	22331.35	21552.58	18405.82	220.63224	144.27502	522.38423	56.179874	53.443779	55.266055	271.03885	589.94187	260.45644	340.57444	291.21281	264.22077	-2.427814	7.9995935	1.76E-143	1.52E-140	0.3381729	7.9995935	0.0001737	0.0008864	0.0141216	7.9995935	0.8753283	0.8851111
3961	ENSMUSG00	17174	Masp1	2	protein codi	MGI Symbol	4	mannan-bin	0	2	4	3	1	1	927	1199	958	1400	1596	1504	0	0.0347412	0.0711091	0.0493152	0.0163691	0.0175328	12.598253	14.581853	13.943836	21.351339	21.564734	21.590347	-0.314787	3.1780611	1	1	8.5271137	3.1780611	1.39E-307	4.39E-304	9.1731812	3.1780611	0	0
3967	ENSMUSG00	23872	Ets2	2	protein codi	MGI Symbol	6	E26 avian le	190	242	215	191	208	181	424	470	428	491	582	538	3.3758475	4.2036909	3.8221159	3.1397343	3.4047818	3.1734391	5.7623079	5.715989	6.2296051	7.4882195	7.8638315	7.7231428	-0.230543	2.3901783	0.0608316	0.3926437	0.6333874	2.3901783	2.15E-08	2.54E-07	1.0165555	2.3901783	9.65E-20	6.17E-19
39867	ENSMUSG00	NA	Gm28635	2	protein codi	MGI Symbol	1	predicted ge	177.97	239.66	224.75	82.58	0	143.11	262.53	322.48	344.43	182.9	151.55	0	3.162103	4.1630437	3.9954444	1.357483	0	2.5091208	3.5678743	3.9218982	5.013231	2.7893999	2.0477039	0	-1.570577	1.4570283	8.69E-29	2.29E-26	0.1392193	1.4570283	0.2340937	0.320076	-1.234239	1.4570283	1.12E-20	7.61E-20
402	ENSMUSG00	22776	Zim1	2	protein codi	MGI Symbol	4	zinc finger, i	216	159	162	187	187	195	694	856	753	1055	1250	1251	3.8378055	2.7619292	2.8799199	3.0739807	3.0610298	3.4188984	9.4317022	10.410397	10.96003	16.089759	16.889672	17.95846	0.0107848	3.0957238	0.9499663	1	1.6998948	3.0957238	9.63E-52	9.84E-50	2.4259388	3.0957238	2.09E-105	1.27E-103
4044	ENSMUSG00	16668	Krt18	2	protein codi	MGI Symbol	1	keratin 18 [S	18	11	17	16	19	24	367	406	315	431	545	577	0.3198171	0.1910769	0.3022138	0.2630144	0.3110137	0.4207875	4.9876581	4.9376416	4.5848729	6.5731622	7.3638972	8.8229892	0.2843758	1.7541702	0.3524416	0.7965509	4.1518425	1.7541702	1.78E-118	6.05E-116	4.7659255	1.7541702	2.23E-168	3.47E-166
4106	ENSMUSG00	16529	Kcnk5	2	protein codi	MGI Symbol	2	potassium cl	70	56	72	105	91	88	367	373	311	380	377	293	1.2437333	0.9727549	1.2799644	1.7260319	1.489592	1.5428875	4.9876581	4.5363062	4.5266523	5.7953634	5.0939252	4.2060982	0.4456965	1.6915419	0.006527	0.0914553	2.0060472	1.6915419	1.35E-48	1.28E-46	2.1082271	1.6915419	6.93E-54	1.67E-52
4148	ENSMUSG00	59092	Pcbp4	2	protein codi	MGI Symbol	23	poly(rC) binc	3000	3054	3172	3622	3577	3591	5949	6880	6312	7194	8290	7911	53.302855	53.049885	56.389543	59.539883	58.552425	62.96033	80.848986	83.67235	91.87212	109.71538	112.01231	113.56465	0.1537627	6.2863908	0.0945632	0.4835194	0.6556683	6.2863908	8.83E-13	1.72E-11	1.0428299	6.2863908	9.98E-30	1.11E-28
41726	ENSMUSG00	NA	Gstp-ps	2	processed p	MGI Symbol	1	glutathione	1125.2	1152.57	1051.39	1272.94	1153.99	1105.21	2298.77	2890.31	2528.72	2641.83	3374.14	2783.61	19.992124	20.02086	18.690858	20.925096	18.889828	19.37744	31.241086	35.151022	36.805904	40.290434	45.590496	39.959512	0.0119803	4.8590945	0.9044106	0.9900784	0.8137116	4.8590945	8.56E-18	2.43E-16	1.1001592	4.8590945	3.78E-31	4.51E-30
44	ENSMUSG00	66355	Gmpr	2	protein codi	MGI Symbol	4	guanosine m	351.83	357.96	388	439.53	464.81	408	814.43	962.67	847.25	913.96	980.93	897.09	6.2511811	6.2179885	6.8975859	7.2251697	7.6085415	7.1533875	11.068388	11.707683	12.331853	13.938764	13.254069	12.877982	0.183385	3.2932952	0.0896495	0.4747324	0.8578588	3.2932952	9.17E-17	2.39E-15	1.0486378	3.2932952	1.98E-24	1.67E-23
4416	ENSMUSG00	212307	Mapre2	2	protein codi	MGI Symbol	9	microtubule	2248	2315	2299	1882	1934	1821	5264	6067	4897	6286	6890	7027	39.941606	40.212994	40.869974	30.937068	31.657923	31.927252	71.539597	73.784905	71.27658	95.867511	93.095875	100.87458	-0.356584	5.9139855	0.0001344	0.0034983	0.8397619	5.9139855	1.02E-19	3.25E-18	1.259888	5.9139855	6.26E-42	1.09E-40
4542	ENSMUSG00	58801	Pmaip1	2	protein codi	MGI Symbol	3	phorbol-12-	85	95	100	122	185	179	278	393	284	289	283	237	1.5102475	1.6502093	1.7777283	2.0054847	3.0282915	3.1383734	3.778117	4.7795397	4.1336632	4.4075264	3.8238218	3.4022023	0.7236718	1.6783319	2.58E-07	1.22E-05	1.3634552	1.6783319	1.38E-25	5.82E-24	1.2335177	1.6783319	1.10E-20	7.44E-20
46670	ENSMUSG00	NA	Gm44732	2	antisense	MGI Symbol	1	predicted ge	423.6	537.31	412.77	970.4	691.01	538.99	1263.65	1301.05	979.35	1295.35	1308.33	1359.66	7.5263631	9.3334098	7.3379292	15.951823	11.311242	9.4500106	17.173444	15.822952	14.254588	19.755326	17.677812	19.518305	0.6021513	3.7924081	4.15E-09	2.58E-07	0.9652815	3.7924081	7.24E-22	2.62E-20	1.2338185	3.7924081	6.33E-35	8.71E-34
4775	ENSMUSG00	12333	Capn1	2	protein codi	MGI Symbol	6	calpain 1 [Sc	533	574	568.97	641.9	641	658.98	1277	1403.97	1158.95	1428.99	1751.94	1506.97	9.4701405	9.970738	10.114741	10.551809	10.492621	11.553773	17.354876	17.074632	16.868694	21.793464	23.671754	21.632982	0.1408704	3.9210079	0.167608	0.6254031	0.7953747	3.9210079	9.59E-16	2.35E-14	1.1828924	3.9210079	3.56E-33	4.55E-32
4832	ENSMUSG00	27360	Add3	2	protein codi	MGI Symbol	16	adducin 3 (g	534	413	512	504	474	450	1113	1209	1005	1294	1402	1251	9.4879081	7.1740676	9.1019691	8.2849533	7.7589739	7.8897656	15.126058	14.70347	14.627928	19.734737	18.943457	17.95846	-0.105734	3.6632456	0.3146649	0.7706279	0.7874148	3.6632456	4.20E-15	9.65E-14	1.1365281	3.6632456	4.59E-30	5.22E-29
4908	ENSMUSG00	63828	Fn3k	2	protein codi	MGI Symbol	3	fructosamin	303	267	236	278	303	236	594	704	641	674	1012	880	5.3835883	4.6379565	4.1954389	4.569875	4.9598504	4.1377438	8.0726673	8.5618218	9.3298525	10.279145	13.673879	12.63265	-0.055587	2.9341534	0.6307062	0.9222269	0.868056	2.9341534	6.30E-16	1.57E-14	1.3647471	2.9341534	3.34E-38	5.23E-37
5	ENSMUSG00	107815	Scml2	2	protein codi	MGI Symbol	9	Scm polycon	121	104	110	120	121	128	254	315	209	271	359	301	2.1498818	1.8065449	1.9555012	1.9726079	1.9806663	2.2442	3.4519486	3.8309288	3.0420268	4.1330092	4.8507139	4.3209405	0.0669195	1.6121625	0.6626316	0.9314156	0.8083329	1.6121625	5.19E-10	7.45E-09	1.1716616	1.6121625	2.94E-20	1.95E-19
5221	ENSMUSG00	80752	Fam20c	2	protein codi	MGI Symbol	6	family with	792	751	753	843	849	847	1781	1919	1763	2203	2361	2006	14.071954	13.045338	13.386294	13.857571	13.897403	14.850292	24.204411	23.338261	25.660733	33.597857	31.901213	28.7967	0.0727885	4.3916867	0.4642049	0.8612616	0.8534885	4.3916867	1.03E-18	3.13E-17	1.2189702	4.3916867	1.47E-36	2.18E-35
5222	ENSMUSG00	19085	Prkar1b	2	protein codi	MGI Symbol	11	protein kina	457	462	421	489	475	518	1535	1677	1540	2405	2734	2195	8.1198015	8.0252282	7.4842363	8.0383773	7.775343	9.0819969	20.861186	20.395135	22.414934	36.67855	36.941092	31.509848	0.0744464	4.190818	0.4810135	0.8657879	1.4293994	4.190818	1.80E-46	1.60E-44	2.1531885	4.190818	1.57E-103	8.99E-102
5411	ENSMUSG00	16011	Igfbp5	2	protein codi	MGI Symbol	2	insulin-like g	6003	5783	6167	7158	7903	7853	30848	36625	32878	27939	26579	21055	106.65901	100.45432	109.63251	117.66606	129.36534	137.68518	419.23508	445.42148	478.54429	426.09647	359.12848	302.2505	0.2804295	8.0290151	0.0020135	0.0364339	2.0842591	8.0290151	1.35E-109	3.88E-107	1.7795436	8.0290151	7.25E-82	2.88E-80
54408	ENSMUSG00	NA	Gm36684	2	lincRNA	MGI Symbol	1	predicted ge	405.29	425.79	425.47	1.11	1.09	2.29	624.5	768.76	631.44	527.51	544.81	432.85	7.201038	7.3962379	7.5637007	0.0182466	0.0178424	0.0401501	8.4871729	9.3494121	9.1907052	8.045032	7.36133	6.2136847	-8.083484	2.5848807	3.02E-217	4.77E-214	0.2865893	2.5848807	0.0057715	0.0165297	-0.036367	2.5848807	0.7395575	0.7571717
56	ENSMUSG00	12561	Cdh4	2	protein codi	MGI Symbol	6	cadherin 4 [	1300	1117	1339	1768	1810	1789	5156	5801	4956	7347	8337	7856	23.097904	19.402987	23.803782	29.06309	29.628149	31.366202	70.071839	70.549898	72.135334	112.04878	112.64736	112.77511	0.4417348	5.883937	3.19E-06	0.0001258	1.682075	5.883937	2.00E-70	3.11E-68	2.3476448	5.883937	1.36E-131	1.30E-129
5630	ENSMUSG00	11931	Atp1b1	2	protein codi	MGI Symbol	4	ATPase, Na+	659.19	521.53	642.37	673.31	709.57	709.45	5110.76	5378.12	3689.05	7541.29	8248.6	6888.05	11.712236	9.0593014	11.419593	11.068139	11.615053	12.438654	69.457012	65.406968	53.694684	115.01188	111.45292	98.879914	0.1256683	5.6045759	0.2151687	0.6926815	2.550697	5.6045759	1.77E-147	8.87E-145	3.337349	5.6045759	2.53E-241	8.60E-239
5911	ENSMUSG00	241447	Cers6	2	protein codi	MGI Symbol	7	ceramide sy	324	246	263	265	306	365	921	1073	817	1139	1178	1032	5.7567083	4.2731734	4.6754255	4.3561758	5.0089578	6.3994766	12.516711	13.049481	11.891559	17.370839	15.916827	14.814653	0.0983928	3.2925055	0.3861446	0.8216464	1.3497377	3.2925055	7.95E-38	5.64E-36	1.7095343	3.2925055	1.34E-60	3.77E-59
60	ENSMUSG00	11684	Alox12	2	protein codi	MGI Symbol	2	arachidonate	779	623	741	474	545	466	4547	4791	3010	6714	5596	5333	13.840975	10.821899	13.172967	7.7918013	8.9211831	8.1702906	61.795317	58.266603	43.811008	102.39492	75.611686	76.556729	-0.603987	5.3328898	3.14E-09	1.99E-07	2.1153043	5.3328898	8.40E-105	2.16E-102	2.750045	5.3328898	2.28E-171	3.61E-169
6530	ENSMUSG00	19885	Rorc	2	protein codi	MGI Symbol	4	RAR-related	185	167	205	220	189	206.95	511	481	447	573.97	721.94	571	3.2870094	2.9008942	3.6443431	3.6164479	3.0937681	3.6284156	6.9446683	5.8497675	6.506153	8.7535914	9.7546641	8.1968671	0.0721773	2.4887368	0.5754434	0.902499	0.9718966	2.4887368	2.01E-17	5.48E-16	1.4422946	2.4887368	2.29E-37	3.47E-36
6598	ENSMUSG00	56376	Pdlim5	2	protein codi	MGI Symbol	13	PDZ and LIM	1193	1185	1103	1377	1487	1375	2308	2697	2408	2595	3051	2997	21.196769	20.584189	19.608344	22.635676	24.340916	24.107617	31.366525	32.800048	35.048806	39.576232	41.224313	43.022786	0.2114579	4.8933317	0.027025	0.2461529	0.6923532	4.8933317	2.44E-13	4.93E-12	1.0122118	4.8933317	1.01E-26	9.75E-26
6733	ENSMUSG00	13131	Dab1	2	protein codi	MGI Symbol	8	disabled 1 [S	631	641	617	734	773	672	1506	1740	1259	1579	1703	1536	11.211367	11.13457	10.968584	12.065785	12.653348	11.78205	20.467065	21.161321	18.324936	24.08126	23.01049	22.049716	0.1319664	4.0599953	0.1914591	0.6590076	0.8481838	4.0599953	4.95E-18	1.44E-16	1.0531255	4.0599953	5.21E-27	5.11E-26
6835	ENSMUSG00	74246	Gale	2	protein codi	MGI Symbol	7	galactose-4-	211	211	203	241	270	237	456	474	469	452	627	487	3.7489674	3.6652016	3.6087885	3.9616543	4.4196687	4.1552766	6.1971991	5.7646357	6.8263663	6.8934322	8.4718597	6.9910233	0.1856625	2.4521371	0.1226401	0.5421773	0.7670695	2.4521371	1.11E-11	1.93E-10	1.0213198	2.4521371	1.06E-19	6.78E-19
6852	ENSMUSG00	50927	Nasp	2	protein codi	MGI Symbol	8	nuclear auto	2210	1924	2371	2630	2772	2577	5714	5693	5872	3432	3601	3178	39.266436	33.42108	42.149939	43.23299	45.375265	45.182058	77.655254	69.236437	85.467853	52.341282	48.655768	45.621092	0.2204494	5.711318	0.0176382	0.1853213	1.0165705	5.711318	5.27E-28	2.44E-26	0.3524489	5.711318	0.0001405	0.0002131
7179	ENSMUSG00	14400	Gabrb1	2	protein codi	MGI Symbol	2	gamma-ami	158	137	162	189	211	176	276	431	337	553	429	437	2.8072837	2.3797755	2.8799199	3.1068575	3.4538892	3.085775	3.7509363	5.2416835	4.9050863	8.4337788	5.7965356	6.2732591	0.2591293	2.1442839	0.0443725	0.3340243	0.7866414	2.1442839	6.32E-11	9.86E-10	1.3427964	2.1442839	2.68E-30	3.09E-29
7184	ENSMUSG00	22223	Uchl1	2	protein codi	MGI Symbol	4	ubiquitin car	17217	20964	18403	21399	21415	20909	58675	68745	54668	73440	92162	82874	305.90508	364.15776	327.15535	351.76531	350.5452	366.59358	797.41372	836.0546	795.70105	1120.0302	1245.2688	1189.6798	0.1001337	9.3902074	0.2658302	0.73627	1.2844742	9.3902074	5.25E-45	4.53E-43	1.8338507	9.3902074	2.93E-87	1.25E-85
723	ENSMUSG00	29811	Ndrg2	2	protein codi	MGI Symbol	13	N-myc dowr	134	125	124	49	78	53	309	380	316	325	352	352	2.3808608	2.171328	2.2043831	0.8054816	1.2767932	0.9292391	4.1994178	4.6214379	4.5994281	4.9565608	4.7561318	5.05306	-1.161765	1.7059357	7.71E-14	7.79E-12	0.9899419	1.7059357	1.25E-15	3.02E-14	1.1270313	1.7059357	6.27E-20	4.06E-19
7566	ENSMUSG00	13848	Ephb6	2	protein codi	MGI Symbol	4	Eph receptor	218	181	152	199	154	169	379	425	319	375	545	459	3.8733408	3.1440829	2.7021471	3.2712415	2.5208481	2.9630453	5.1507422	5.1687135	4.6430935	5.7191086	7.3638972	6.5890753	-0.150897	2.172712	0.2393253	0.7176737	0.6235691	2.172712	9.82E-08	1.06E-06	1.0188766	2.172712	7.64E-19	4.60E-18
788	ENSMUSG00	15507	Hspb1	2	protein codi	MGI Symbol	2	heat shock p	41444.64	46518.75	43854	72919.12	72471.68	70635.26	118380.02	129697.57	120652.11	128506.72	157634.23	96132.83	736.37254	808.0597	779.60498	1198.6736	1186.2993	1238.4348	1608.8258	1577.3402	1756.1098	1959.8504	2129.9124	1380.0141	0.6407087	10.412996	1.14E-12	1.05E-10	1.0885324	10.412996	3.65E-33	2.00E-31	1.2348483	10.412996	6.23E-42	1.09E-40
7922	ENSMUSG00	17304	Mfge8	2	protein codi	MGI Symbol	10	milk fat glob	5087	5202	5282	6640	6616	6303	15243	14739	12223	13091	15781	14366	90.383874	90.361985	93.899611	109.15097	108.29825	110.50932	207.15769	179.25098	177.90762	199.65027	213.22874	206.22801	0.2559329	7.218761	0.0049113	0.0740587	1.0389163	7.218761	6.46E-30	3.16E-28	1.1726285	7.218761	1.79E-37	2.73E-36
7960	ENSMUSG00	18576	Pde3b	2	protein codi	MGI Symbol	4	phosphodies	244	241	267	261	235	221	612	685	514	615	744	696	4.3352988	4.1863203	4.7465347	4.2904222	3.8467487	3.8747516	8.3172935	8.3307499	7.4813482	9.3793381	10.052733	9.9912776	-0.142795	2.7376835	0.2266981	0.7065721	0.8637354	2.7376835	2.58E-15	6.07E-14	1.1492901	2.7376835	3.40E-26	3.23E-25
8066	ENSMUSG00	29810	Bag3	2	protein codi	MGI Symbol	1	BCL2-associ	768	815	859	939	1083	1044	2188	2741	2355	2529	2447	2302	13.645531	14.157058	15.270686	15.435657	17.727782	18.304256	29.735683	33.335161	34.277383	38.569668	33.063223	33.045864	0.2565753	4.634263	0.008776	0.1137754	1.1762493	4.634263	1.93E-34	1.12E-32	1.2807082	4.634263	1.90E-40	3.17E-39
8469	ENSMUSG00	108150	Galnt7	2	protein codi	MGI Symbol	4	polypeptide	173.89	125	150	174.91	182	195	356	349	320	364	394	395	3.0896111	2.171328	2.6665925	2.8752404	2.9791841	3.4188984	4.8381642	4.2444259	4.6576487	5.5513481	5.3236248	5.6703372	0.2254564	2.0106504	0.0820809	0.4558759	0.7925755	2.0106504	5.36E-11	8.43E-10	1.061586	2.0106504	7.34E-19	4.44E-18
8632	ENSMUSG00	72361	Ces2g	2	protein codi	MGI Symbol	2	carboxyleste	96	94	100	86	67	76	377	394	262	267	345	306	1.7056913	1.6328386	1.7777283	1.4137023	1.0967326	1.3324938	5.1235615	4.7917014	3.8134498	4.0720053	4.6615496	4.3927169	-0.413041	1.6289997	0.0088368	0.1142175	1.4260349	1.6289997	1.43E-28	6.78E-27	1.359998	1.6289997	1.18E-25	1.07E-24
8651	ENSMUSG00	330836	Slc7a6	2	protein codi	MGI Symbol	7	solute carrie	1305	1244	1380	1362	1442	1289	3018	3199	2600	3034	3377	3333	23.186742	21.609056	24.532651	22.3891	23.604305	22.599795	41.015673	38.90521	37.843395	46.271401	45.629139	47.846161	-0.015249	5.0469632	0.8751474	0.9848106	0.7644612	5.0469632	4.35E-16	1.10E-14	1.0112973	5.0469632	6.37E-27	6.23E-26
8689	ENSMUSG00	73230	Bmper	2	protein codi	MGI Symbol	3	BMP-binding	1093	847	1078	1224	1181	1166	2341	2820	2749	3021	2850	2641	19.420007	14.712918	19.163911	20.120601	19.331958	20.443259	31.815007	34.295934	40.012113	46.073139	38.508453	37.912305	0.1685591	4.8376263	0.0814013	0.4540881	0.9934412	4.8376263	1.54E-25	6.42E-24	1.2004607	4.8376263	1.88E-36	2.76E-35
8697	ENSMUSG00	56199	Abcb10	2	protein codi	MGI Symbol	1	ATP-binding	112.37	120.47	114.4	128.84	111.97	131.87	259.91	283.34	215.39	263.81	302.22	294.21	1.9965473	2.092639	2.0337212	2.1179234	1.832853	2.312052	3.5322676	3.4458901	3.1350342	4.0233548	4.0835174	4.2234681	0.0303201	1.5721076	0.8411705	0.9793456	0.72468	1.5721076	2.12E-08	2.52E-07	1.009218	1.5721076	1.75E-15	8.16E-15
8790	ENSMUSG00	12282	Hyou1	2	protein codi	MGI Symbol	9	hypoxia up-r	1854	1513	1659	1659	1697	1742	3756	3613	2951	3857	4386	4321	32.941164	26.281754	29.492513	27.271305	27.778436	30.542159	51.045351	43.940145	42.952254	58.822938	59.262483	62.029182	-0.051803	5.3621466	0.5842359	0.9056802	0.6368393	5.3621466	8.31E-12	1.47E-10	1.0217314	5.3621466	7.07E-28	7.27E-27
8914	ENSMUSG00	14860	Gsta4	2	protein codi	MGI Symbol	2	glutathione	362	232	518	112	121	131	498	686	374	191	449	394	6.4318778	4.0299847	9.2086328	1.8411007	1.9806663	2.2967984	6.7679938	8.3429116	5.4436269	2.9129327	6.0667703	5.6559819	-1.684684	2.363651	1.68E-42	6.13E-40	0.0693813	2.363651	0.5346291	0.6193873	-0.418704	2.363651	0.0001145	0.0001751
8969	ENSMUSG00	71640	Zfp949	2	protein codi	MGI Symbol	5	zinc finger p	402	253	348	165	185	176	362	589	402	326	421	458	7.1425825	4.3947678	6.1864946	2.7123359	3.0282915	3.085775	4.9197063	7.1632288	5.8511711	4.9718118	5.6884417	6.5747201	-1.005036	2.3779231	1.96E-17	2.86E-15	0.0210548	2.3779231	0.8674208	0.9024748	-0.038278	2.3779231	0.7276583	0.745632
9095	ENSMUSG00	269132	Colgalt2	2	protein codi	MGI Symbol	2	collagen bet	272	293	304	346	286	327	1029	1072	791	729	865	703	4.8327922	5.0895928	5.4042941	5.6876862	4.681575	5.7332297	13.984469	13.03732	11.513125	11.117947	11.687653	10.091765	0.0703963	3.1197765	0.5355809	0.8853347	1.3304778	3.1197765	3.95E-37	2.64E-35	1.1019209	3.1197765	1.69E-25	1.52E-24
9273	ENSMUSG00	23945	Mgll	2	protein codi	MGI Symbol	10	monoglyceri	106	122	120	175	156	153	631	676	476.48	688	777	650	1.8833675	2.1192161	2.133274	2.8767199	2.5535863	2.6825203	8.5755102	8.2212948	6.9352389	10.492658	10.49862	9.3309346	0.4024127	2.5441391	0.0030859	0.0520708	1.9516864	2.5441391	6.21E-59	7.87E-57	2.3036935	2.5441391	7.07E-83	2.86E-81
9538	ENSMUSG00	232237	Fgd5	2	protein codi	MGI Symbol	4	FYVE, RhoGE	1818	1654	1947	2006	2023	1949	4569	5775	4475	5973	7012	6345	32.30153	28.731012	34.612371	32.975429	33.114777	34.171452	62.094304	70.233695	65.134306	91.093962	94.744307	91.084277	0.0680432	5.8072164	0.4689589	0.864464	1.0459977	5.8072164	2.82E-29	1.36E-27	1.5337892	5.8072164	3.48E-60	9.62E-59
958	ENSMUSG00	56213	Htra1	2	protein codi	MGI Symbol	5	HtrA serine p	6376	6860	6750	7167	6888	6721	22061	23448	17880	21812	28417	24526	113.28633	119.16248	119.99666	117.81401	112.75066	117.83803	299.81669	285.16704	260.24612	332.65386	383.96306	352.07769	-0.01666	7.7685604	0.854959	0.9820332	1.2619529	7.7685604	3.63E-43	2.92E-41	1.6003866	7.7685604	2.77E-67	8.63E-66
9609	ENSMUSG00	14733	Gpc1	2	protein codi	MGI Symbol	5	glypican 1 [S	2019.78	1938.61	2119.64	1880.23	1860.51	1917.91	5215.01	5752.15	4840.79	5751.24	7512.15	6417.21	35.88668	33.674865	37.681441	30.907972	30.454955	33.626357	70.873806	69.9558	70.458435	87.71191	101.5022	92.120872	-0.175154	5.8590275	0.0614136	0.3938332	0.9783291	5.8590275	5.42E-26	2.33E-24	1.3915169	5.8590275	2.54E-50	5.55E-49
9733	ENSMUSG00	19249	Ptpn13	2	protein codi	MGI Symbol	5	protein tyro	289	217	214	213	244	224	501	530	488	584	681	647	5.1348417	3.7694253	3.8043386	3.5013791	3.9940709	3.92735	6.8087648	6.4456897	7.1029142	8.9065585	9.2014935	9.2878687	-0.153697	2.6034443	0.1975649	0.6666121	0.6789217	2.6034443	8.55E-10	1.19E-08	1.1084869	2.6034443	3.75E-24	3.10E-23
9763	ENSMUSG00	16979	Lrrn1	2	protein codi	MGI Symbol	1	leucine rich	93	111	131	108	111	83	616	787	574	643	689	523	1.6523885	1.9281392	2.3288241	1.7753471	1.8169749	1.4552234	8.3716549	9.5712412	8.3546573	9.8063649	9.3095875	7.5078135	-0.224246	2.4537718	0.1342022	0.5634628	2.1542793	2.4537718	2.30E-71	3.64E-69	2.1703125	2.4537718	3.78E-72	1.31E-70
9915	ENSMUSG00	13631	Eef2k	2	protein codi	MGI Symbol	9	eukaryotic e	185	172	188	189	168	145	785.99	846	644	974	1283	1210	3.2870094	2.9877473	3.3421293	3.1068575	2.7500161	2.5422578	10.681878	10.28878	9.373518	14.854431	17.33556	17.369894	-0.193396	3.0559579	0.1267687	0.5491396	1.6581902	3.0559579	2.05E-49	2.00E-47	2.3657551	3.0559579	1.02E-100	5.63E-99
993	ENSMUSG00	21687	Tek	2	protein codi	MGI Symbol	4	TEK receptor	296	229	327	261	268	233	850	1101	869	877	1115	1100	5.259215	3.9778728	5.8131717	4.2904222	4.3869304	4.0851453	11.551797	13.390008	12.648427	13.375089	15.065588	15.790812	-0.236376	3.2129884	0.0413858	0.321476	1.3216135	3.2129884	1.85E-36	1.22E-34	1.5562638	3.2129884	2.32E-50	5.07E-49
999	ENSMUSG00	240913	Adamts4	2	protein codi	MGI Symbol	3	a disintegrin	1056	1249	683	1354	1250	1370	5084	5519	4152.89	4321	6683	5470	18.762605	21.695909	12.141885	22.257593	20.461429	24.019953	69.093334	67.120305	60.445946	65.899382	90.298945	78.523403	0.3427189	5.5261117	0.0003664	0.0086809	1.902181	5.5261117	9.72E-88	2.05E-85	2.1575659	5.5261117	3.24E-111	2.18E-109

Table S2. Differentially expressed genes (> 2-fold) in *Trpm3*-mutant lenses summarized in Fig. 9A.

10086	ENSMUSG00	219228	Pcdh17	3	protein codi	MGI Symbol	2	protocadher	643	598	554	1698	2193	2052	642	891	439	560	632	434	11.424579	10.387633	9.848615	27.912402	35.897531	35.977331	8.725004	10.836056	6.3897118	8.5405355	8.5394184	6.2301932	1.6557982	3.9073991	5.85E-64	2.93E-61	-0.282193	3.9073991	0.0050701	0.01488	-0.441632	3.9073991	1.64E-05	2.73E-05
10178	ENSMUSG00	18483	Palm	3	protein codi	MGI Symbol	8	paralemmin	739	2948	9	6753	6776	6448	1122	1201	4162	1183	2330	1182	13.13027	51.208599	0.1599956	111.00851	110.91731	113.05158	15.248371	14.606176	60.578543	18.041881	31.482349	16.967946	2.374702	5.5331785	2.32E-134	1.58E-131	0.4812553	5.5331785	2.53E-07	2.55E-06	0.0437772	5.5331785	0.6309978	0.6529222
10279	ENSMUSG00	78376	Sapcd1	3	protein codi	MGI Symbol	4	suppressor A	1844.71	1846.11	1974.92	1909.6	1992.39	1875.1	1703.54	1822.76	1479.02	771.9	1084.55	989.16	32.776103	32.068082	35.108712	31.390767	32.613717	32.875777	23.151703	22.167822	21.527361	11.772213	14.654155	14.199673	-0.045052	4.66222	0.632054	0.9225563	-0.580228	4.66222	6.90E-10	9.71E-09	-1.29772	4.66222	1.39E-41	2.40E-40
10365	ENSMUSG00	214058	Megf11	3	protein codi	MGI Symbol	10	multiple EGF	399	316	254	612	604	648	75	58	108	37	50	56	7.0892797	5.4891171	4.51543	10.0603	9.8869625	11.361263	1.0192762	0.7053774	1.5719564	0.5642854	0.6755869	0.8038959	0.872328	2.128237	1.62E-16	2.24E-14	-2.395141	2.128237	5.72E-72	9.22E-70	-3.056733	2.128237	2.44E-99	1.30E-97
10409	ENSMUSG00	242939	Cpz	3	protein codi	MGI Symbol	2	carboxypept	908	783	1042	1024	1018	993	812	734	667	400	403	412	16.132997	13.601198	18.523929	16.832921	16.663788	17.410083	11.035363	8.9266722	9.7082864	6.1003825	5.4452304	5.914377	0.0772256	3.6019116	0.4309459	0.8463211	-0.702216	3.6019116	1.20E-12	2.30E-11	-1.467112	3.6019116	7.26E-47	1.45E-45
10641	ENSMUSG00	231125	Zfyve28	3	protein codi	MGI Symbol	6	zinc finger, F	1958	2100	2374	2812	3200	2833	1971.98	2478.99	2161	866	796.98	753	34.788996	36.47831	42.203271	46.224779	52.381258	49.670458	26.799896	30.14868	31.453684	13.207328	10.768585	10.809529	0.3859814	5.0012878	3.18E-05	0.001007	-0.360194	5.0012878	0.0001117	0.0006059	-1.706649	5.0012878	2.14E-69	6.99E-68
10702	ENSMUSG00	18605	Enpp1	3	protein codi	MGI Symbol	5	ectonucleoti	237	275	267	211	189	192	244	297	198	115	142	122	4.2109255	4.7769215	4.7465347	3.4685023	3.0937681	3.3663	3.3160451	3.6120186	2.8819201	1.75386	1.9186668	1.7513446	-0.468369	1.6971968	9.38E-05	0.0025461	-0.481493	1.6971968	3.50E-05	0.0002187	-1.338094	1.6971968	4.05E-26	3.82E-25
10752	ENSMUSG00	226970	Arhgef4	3	protein codi	MGI Symbol	7	Rho guanine	697.32	726.93	645.02	1083.71	1014.71	1149.04	435.35	440.83	386.92	312.39	456.72	371.42	12.389716	12.627228	11.466703	17.814458	16.609933	20.145903	5.9165584	5.3612328	5.6316794	4.7642462	6.171081	5.3318396	0.5802086	3.3623317	3.69E-09	2.32E-07	-1.109982	3.3623317	5.12E-27	2.27E-25	-1.162717	3.3623317	3.30E-29	3.58E-28
11177	ENSMUSG00	19736	Rgs4	3	protein codi	MGI Symbol	3	regulator of	1506	1422	1360	1535	1528	1552	775	686	621	504	478	302	26.758033	24.701027	24.177105	25.232943	25.012051	27.210925	10.53252	8.3429116	9.0387495	7.686482	6.4586107	4.3352958	0.0341421	4.0464031	0.7203254	0.9477493	-1.438751	4.0464031	7.84E-49	7.52E-47	-2.034409	4.0464031	6.33E-91	2.86E-89
11246	ENSMUSG00	268935	Scube3	3	protein codi	MGI Symbol	7	signal peptic	2970.87	2687.7	3089.1	2606.6	2559.27	2531.44	3365.29	4014.2	3452	951.32	896.04	953.3	52.785284	46.687025	54.915806	42.848332	41.893057	44.383263	45.735465	48.819411	50.244385	14.50854	12.107058	13.684892	-0.257793	5.2867574	0.0053107	0.0787472	-0.092484	5.2867574	0.3152741	0.4065016	-1.938289	5.2867574	8.41E-90	3.68E-88
11430	ENSMUSG00	227541	Camk1d	3	protein codi	MGI Symbol	4	calcium/calr	1392	1285	1509	1295	1310	1296	815	931	799	545	646	665	24.732525	22.321251	26.825921	21.287727	21.443578	22.722525	11.076134	11.322523	11.629567	8.3117712	8.7285827	9.5462638	-0.174726	4.0526166	0.0669595	0.4085518	-1.118363	4.0526166	9.43E-31	4.79E-29	-1.47385	4.0526166	1.18E-50	2.61E-49
11718	ENSMUSG00	268512	Slc26a11	3	protein codi	MGI Symbol	4	solute carrie	132	98	185	350	480	328	156	236	226	192	224	221	2.3453256	1.7023211	3.2887974	5.7534398	7.8571887	5.7507625	2.1200944	2.8701562	3.2894644	2.9281836	3.0266293	3.1725178	1.4023128	1.8802729	3.74E-31	1.15E-28	0.1743265	1.8802729	0.1810754	0.2604021	0.3171917	1.8802729	0.0137626	0.0170886
11916	ENSMUSG00	17872	Ppp1r15a	3	protein codi	MGI Symbol	3	protein phos	8340	13367	6114	8754	9049	8365	6800	7452	6293	4650	5434	5316	148.18194	232.19313	108.69031	143.90175	148.12438	146.66198	92.414373	90.62883	91.595572	70.916947	73.422784	76.31269	-0.156917	6.889188	0.0829932	0.4570411	-0.832578	6.889188	6.44E-20	2.08E-18	-1.148283	6.889188	6.31E-36	9.04E-35
11922	ENSMUSG00	77593	Usp45	3	protein codi	MGI Symbol	8	ubiquitin spe	3153	3893	2427	3418	4306	3437	1596	1767	1534	1099	1050	831	56.0213	67.623838	43.145467	56.186449	70.485531	60.260277	21.690197	21.489686	22.327603	16.760801	14.187325	11.929241	0.1644041	5.2632438	0.0736021	0.4294221	-1.348508	5.2632438	1.55E-46	1.39E-44	-1.960518	5.2632438	3.51E-92	1.65E-90
11969	ENSMUSG00	14863	Gstm2	3	protein codi	MGI Symbol	4	glutathione S	1068.07	1000.8	1170	1267.96	1197.38	1267.36	584.44	486.63	414.78	129.92	140.87	152	18.97706	17.38452	20.799422	20.843233	19.600085	22.220385	7.9427435	5.9182377	6.037186	1.9814042	1.9033985	2.1820032	0.1324604	3.5890204	0.1690127	0.6268354	-1.522668	3.5890204	4.87E-52	5.08E-50	-3.23525	3.5890204	2.16E-171	3.54E-169
12204	ENSMUSG00	12319	Car8	3	protein codi	MGI Symbol	1	carbonic anh	361	318	372	328	320	312	101	156	123	54	62	40	6.4141102	5.5238583	6.6131494	5.391795	5.2381258	5.4702375	1.3726252	1.8972219	1.7902837	0.8235516	0.8377278	0.5742114	-0.204194	1.7739494	0.0658762	0.4042792	-1.869981	1.7739494	5.11E-52	5.27E-50	-3.048584	1.7739494	5.61E-103	3.19E-101
12310	ENSMUSG00	72315	Ccdc74a	3	protein codi	MGI Symbol	10	coiled-coil d	243	226	277	309	311	292	232	273	250	83	133	144	4.3175312	3.925761	4.9243075	5.0794654	5.0908035	5.1195813	3.152961	3.3201383	3.638788	1.2658294	1.7970611	2.0671609	0.2159154	1.8599082	0.0624593	0.3950971	-0.38167	1.8599082	0.001165	0.004443	-1.35314	1.8599082	3.85E-26	3.64E-25
12400	ENSMUSG00	239319	Card6	3	protein codi	MGI Symbol	2	caspase recr	1098.27	1308.34	886	1106.29	1227.55	1126.47	796	772	833	498	388.75	455	19.513642	22.726682	15.750673	18.185637	20.093942	19.750187	10.817918	9.388816	12.124442	7.5949762	5.2526881	6.5316542	0.0006223	3.7988118	0.9969352	1	-0.844998	3.7988118	6.23E-18	1.80E-16	-1.584219	3.7988118	1.56E-55	3.94E-54
12419	ENSMUSG00	104681	Slc16a6	3	protein codi	MGI Symbol	5	solute carrie	173.95	191.09	222.25	264.33	319.95	280.98	112.72	184.6	97.78	49.53	69.74	62.17	3.0906772	3.3193525	3.9510012	4.3451621	5.2373074	4.9263697	1.5319041	2.2450459	1.4232028	0.7553799	0.9423086	0.892468	0.4855695	1.4262918	4.23E-05	0.0012953	-0.98124	1.4262918	2.77E-14	6.03E-13	-1.992727	1.4262918	2.69E-42	4.77E-41
12646	ENSMUSG00	13048	Cux2	3	protein codi	MGI Symbol	11	cut-like hom	577	548	432	522	524	556	464	598	429	269	277	242	10.251916	9.5191018	7.6797864	8.5808445	8.577431	9.7482438	6.3059219	7.2726839	6.2441603	4.1025072	3.7427514	3.4739787	-0.02969	2.8289304	0.7834836	0.9653561	-0.467848	2.8289304	6.49E-06	4.84E-05	-1.278855	2.8289304	2.46E-31	2.96E-30
12697	ENSMUSG00	231712	Trafd1	3	protein codi	MGI Symbol	9	TRAF type zi	9081.84	9288.61	7730.68	9174.67	10094.48	8694.91	7441.02	8463.24	7924.57	5278.98	4435.97	4938.02	161.36267	161.34895	137.43049	150.81689	165.23799	152.44623	101.12606	102.92721	115.34332	80.509493	59.937664	70.886679	0.0259769	6.9256718	0.7744041	0.9623913	-0.526832	6.9256718	6.44E-09	8.13E-08	-1.122835	6.9256718	2.08E-34	2.80E-33
12740	ENSMUSG00	216028	Lrrtm3	3	protein codi	MGI Symbol	4	leucine rich	278	215	310	239	295	251	140	119	80	19	31	19	4.9393979	3.7346841	5.5109578	3.9287775	4.8288972	4.4007359	1.9026489	1.4472398	1.1644122	0.2897682	0.4188639	0.2727504	-0.107382	1.4187816	0.3604215	0.8022503	-1.646429	1.4187816	9.54E-38	6.71E-36	-3.834275	1.4187816	1.63E-115	1.23E-113
1295	ENSMUSG00	76257	Slc38a3	3	protein codi	MGI Symbol	14	solute carrie	2917	2591	2746	2714	2793	2713	2404	2337	1998	1426	1598	1451	51.828142	45.007286	48.81642	44.613816	45.719017	47.56652	32.671199	28.421843	29.081194	21.747864	21.591757	20.829517	-0.078923	5.1876397	0.3939311	0.8260973	-0.691776	5.1876397	1.01E-13	2.09E-12	-1.182475	5.1876397	4.05E-36	5.84E-35
1298	ENSMUSG00	71889	Epn3	3	protein codi	MGI Symbol	4	epsin 3 [Sou	852	743	724	808	805	859	414	508	446	228	244	231	15.138011	12.906373	12.870753	13.282227	13.17716	15.060686	5.6264045	6.1781328	6.4915978	3.477218	3.2968641	3.3160706	0.0207803	3.1952817	0.8366744	0.9788296	-1.161153	3.1952817	5.23E-30	2.57E-28	-2.019608	3.1952817	5.73E-76	2.12E-74
13135	ENSMUSG00	237761	Sowaha	3	protein codi	MGI Symbol	1	sosondowah	1344	1264	1361.95	1506	1470	1391	849	911	942	474	553	545	23.879679	21.956468	24.211771	24.756229	24.062641	24.388142	11.538206	11.079289	13.710953	7.2289533	7.4719911	7.8236298	0.0637087	4.0680577	0.5050848	0.8736006	-0.948493	4.0680577	1.27E-22	4.71E-21	-1.63627	4.0680577	8.23E-61	2.33E-59
13180	ENSMUSG00	30923	Foxe3	3	protein codi	MGI Symbol	1	forkhead bo:	5327	4324	3870	4586	4078	4231	4045	3597	2895	1465	1959	1590	94.648102	75.110577	68.798087	75.3865	66.753366	74.18133	54.972961	43.745558	42.137165	22.342651	26.469495	22.824902	-0.141173	5.7957487	0.1225217	0.5421773	-0.760069	5.7957487	1.38E-16	3.58E-15	-1.734979	5.7957487	2.35E-75	8.60E-74
13224	ENSMUSG00	14362	Fzd1	3	protein codi	MGI Symbol	1	frizzled class	2802	2458	2440	2629	2505	2918	2229	2368	1671	1310	1529	1484	49.784866	42.696993	43.376571	43.216552	41.004704	51.160747	30.292888	28.798855	24.321659	19.978753	20.659447	21.303241	-0.005294	5.1155978	0.9568991	1	-0.703204	5.1155978	4.44E-14	9.50E-13	-1.132908	5.1155978	3.16E-33	4.06E-32
13244	ENSMUSG00	239368	Erich5	3	protein codi	MGI Symbol	1	glutamate ri	3810	3300	3494	3248	3419	3602	2727	3435	2425	1115	1523	1511	67.694625	57.323058	62.113828	53.391921	55.966101	63.153191	37.060882	41.775366	35.296244	17.004816	20.578377	21.690834	-0.117468	5.4713209	0.2013778	0.6724391	-0.712841	5.4713209	1.16E-14	2.58E-13	-1.657846	5.4713209	1.94E-68	6.25E-67
13719	ENSMUSG00	320472	Ppm1e	3	protein codi	MGI Symbol	2	protein phos	1419	1504	1811	2493.33	2859	2735	1573	2361	1933	960	784	668	25.21225	26.125418	32.19466	40.986354	46.79938	47.952242	21.377619	28.713723	28.135109	14.640918	10.593203	9.5893297	0.7003363	4.7896441	7.57E-14	7.73E-12	-0.094397	4.7896441	0.3157262	0.4069189	-1.263871	4.7896441	9.48E-39	1.51E-37
13801	ENSMUSG00	329628	Fat4	3	protein codi	MGI Symbol	2	FAT atypical	3099.39	2459.13	3118.07	2043.46	3036.83	2468.07	1225.2	2037.6	1922.88	1416.37	1475.61	1791.86	55.068778	42.716622	55.430814	33.591212	49.710305	43.272209	16.650896	24.780637	27.987811	21.600997	19.938056	25.722659	-0.275506	5.1142936	0.0029007	0.0492956	-1.142315	5.1142936	4.43E-34	2.57E-32	-1.187744	5.1142936	1.57E-36	2.31E-35
14144	ENSMUSG00	NA	Sox1ot	3	sense overl	MGI Symbol	2	Sox1 overlap	9386.99	9369	7735	8892.99	9044.98	9348	7690	9003	8064	4418.98	5239.99	4819	166.78445	162.74537	137.50729	146.18652	148.05857	163.89673	104.50978	109.49159	117.37275	67.393671	70.801372	69.178113	-0.027797	6.930208	0.7592612	0.9601813	-0.495133	6.930208	4.79E-08	5.35E-07	-1.171245	6.930208	3.10E-37	4.69E-36
14172	ENSMUSG00	319832	Tmem229a	3	protein codi	MGI Symbol	1	transmembr	478	418	458	472	487	453	290	338	193	345	504	399	8.4929215	7.2609207	8.1419958	7.7589245	7.9717727	7.9423641	3.9412012	4.1106474	2.8091444	5.2615799	6.8099159	5.7277583	-0.013291	2.6625565	0.9062842	0.9904176	-1.131241	2.6625565	9.19E-25	3.75E-23	-0.42232	2.6625565	6.64E-05	0.0001043
14240	ENSMUSG00	319710	Frmd6	3	protein codi	MGI Symbol	4	FERM domai	1250.58	1215.72	856.69	1044.68	1273.81	1111.08	529.47	511.46	444.82	519.82	587.32	574.23	22.219828	21.117815	15.229621	17.172867	20.851178	19.480357	7.195682	6.2202122	6.4744228	7.9277521	7.9357139	8.2432347	-0.026591	3.7289597	0.786472	0.9660842	-1.558516	3.7289597	1.70E-54	1.87E-52	-1.280569	3.7289597	6.60E-38	1.02E-36
14624	ENSMUSG00	22040	Trex1	3	protein codi	MGI Symbol	2	three prime	8585.67	8100.97	8037.95	9481.97	8557.01	8203.84	7943.87	9365.82	9503.75	3455.84	2607.14	2774.67	152.54691	140.7189	142.89291	155.86841	140.07092	143.83639	107.95997	113.90409	138.32853	52.704865	35.226992	39.831176	0.0119313	6.8280391	0.8956721	0.9886006	-0.276223	6.8280391	0.002294	0.0077049	-1.77199	6.8280391	5.57E-80	2.15E-78
14754	ENSMUSG00	266744	Lgsn	3	protein codi	MGI Symbol	2	lengsin, lens	4154.45	460.36	33	5722.73	7292.27	7377.9	2885.7	1245.67	580.32	2910.11	840.63	1018.85	73.814682	7.9967403	0.5866504	94.072521	119.36821	129.35534	39.21767	15.149438	8.4466459	44.38196	11.358372	14.625881	2.0572244	5.5372095	9.47E-104	6.00E-101	-0.390142	5.5372095	4.17E-05	0.0002538	-0.230933	5.5372095	0.0164699	0.0202883
14872	ENSMUSG00	280635	Emilin3	3	protein codi	MGI Symbol	2	elastin micro	2030	1845	1949	2070	1894	1920	1836	1790	1418	313	439	360	36.068265	32.048801	34.647925	34.027487	31.003157	33.663	24.951881	21.769405	20.639206	4.7735493	5.931653	5.1679022	-0.058327	4.5644132	0.5343653	0.8850615	-0.609163	4.5644132	9.18E-11	1.41E-09	-2.692151	4.5644132	1.60E-155	1.98E-153
14945	ENSMUSG00	14609	Gja1	3	protein codi	MGI Symbol	8	gap junction	2156	1887	1946	1976	2094	2262	1175	1441	1012	1440	2285	1675	38.306985	32.778367	34.594593	32.482277	34.276986	39.659222	15.96866	17.524979	14.729814	21.961377	30.874321	24.045101	0.0098216	4.8104337	0.917191	0.9915152	-1.130794	4.8104337	1.35E-32	7.29E-31	-0.458026	4.8104337	1.00E-06	1.90E-06
15274	ENSMUSG00	74694	Tbc1d30	3	protein codi	MGI Symbol	5	TBC1 domain	654	657	545	426.5	504	488	253	272.3	254	334	296.72	259.17	11.620022	11.4125	9.6886194	7.0109773	8.2500482	8.5560125	3.4383583	3.3116251	3.6970086	5.0938194	4.0092029	3.7204589	-0.458866	2.7192217	8.77E-06	0.000318	-1.648574	2.7192217	1.79E-51	1.81E-49	-1.353875	2.7192217	2.91E-36	4.22E-35
15438	ENSMUSG00	75770	Brsk2	3	protein codi	MGI Symbol	17	BR serine/th	393	463	355	256	397	322	168	205	176	225	191	212	6.982674	8.0425988	6.3109356	4.2082302	6.4985499	5.6455656	2.2831786	2.4931441	2.5617068	3.4314652	2.5807419	3.0433202	-0.38436	2.1567683	0.0004392	0.01003	-1.539798	2.1567683	4.85E-40	3.60E-38	-1.240033	2.1567683	2.76E-27	2.74E-26
15521	ENSMUSG00	208936	Adamts18	3	protein codi	MGI Symbol	7	a disintegrin	9725	8562	9949	10014	11083	10123	7291	8605	7984	5425	5914	5934	172.79009	148.72728	176.86619	164.61413	181.41921	177.48466	99.087234	104.65125	116.20833	82.736438	79.908418	85.184255	0.0709951	7.0490411	0.4324345	0.8466385	-0.639481	7.0490411	1.91E-12	3.56E-11	-1.007901	7.0490411	3.04E-28	3.17E-27
15875	ENSMUSG00	211187	Lrtm2	3	protein codi	MGI Symbol	4	leucine-rich	641	628	575	695	677	707	787	787	568	359	331	285	11.389043	10.908752	10.221938	11.424688	11.08191	12.395698	10.695605	9.5712412	8.2673264	5.4750933	4.4723853	4.0912559	0.1015416	3.194759	0.3160781	0.7715928	-0.187674	3.194759	0.0626119	0.11187	-1.213751	3.194759	5.87E-30	6.63E-29
16494	ENSMUSG00	268860	Abat	3	protein codi	MGI Symbol	7	4-aminobuty	800	816	874	880	865	852	786	909	663	473	541	474	14.214095	14.174429	15.537346	14.465791	14.159309	14.937956	10.682014	11.054966	9.6500658	7.2137023	7.3098502	6.8044046	-0.012059	3.5437951	0.9072678	0.9905825	-0.483752	3.5437951	9.70E-07	8.70E-06	-1.041959	3.5437951	7.22E-25	6.24E-24
16545	ENSMUSG00	14862	Gstm1	3	protein codi	MGI Symbol	4	glutathione S	12656.9	11760.87	13323.83	11946.88	12568.78	11924.9	6955.97	6122.97	5325.99	3049.99	3712.91	3720.99	224.88297	204.29365	236.8615	196.38758	205.74016	209.07704	94.534059	74.465594	77.520595	46.515264	50.167867	53.415868	-0.123947	7.1227973	0.1696178	0.6268354	-1.433944	7.1227973	1.27E-54	1.42E-52	-2.149531	7.1227973	5.72E-115	4.18E-113
16912	ENSMUSG00	75764	Slx1b	3	protein codi	MGI Symbol	4	SLX1 structu	2493	2211.86	2044.26	1745.44	2045.43	2168.28	1598.59	1633.79	2335.61	1035.65	1129.89	946.14	44.294672	38.421388	36.341389	28.69224	33.481937	38.016047	21.725396	19.869629	33.995159	15.794653	15.266778	13.582108	-0.249224	4.8195238	0.0075968	0.1030996	-0.657257	4.8195238	2.36E-12	4.37E-11	-1.415147	4.8195238	1.73E-49	3.70E-48
17455	ENSMUSG00	13869	Erbb4	3	protein codi	MGI Symbol	5	erb-b2 recep	696	527	564	613	775	720	145	162	184	140	276	265	12.366262	9.1543187	10.026388	10.076739	12.686086	12.623625	1.9706006	1.970192	2.678148	2.1351339	3.7292397	3.8041503	0.1655736	2.7734919	0.1021194	0.5026278	-2.258092	2.7734919	7.64E-85	1.51E-82	-1.698968	2.7734919	3.28E-53	7.75E-52
1861	ENSMUSG00	59091	Jph2	3	protein codi	MGI Symbol	2	junctophilin	412	415	488	358	351	373	227	243	176	387	459	339	7.3202587	7.2088088	8.6753143	5.884947	5.7455693	6.5397391	3.0850092	2.9552879	2.5617068	5.9021201	6.2018877	4.8664413	-0.353327	2.4731982	0.0010412	0.0208678	-1.428532	2.4731982	2.02E-36	1.30E-34	-0.450563	2.4731982	2.43E-05	3.97E-05
19177	ENSMUSG00	NA	H2-K2	3	transcribed	MGI Symbol	2	histocompat	701	750.83	594.94	630.99	723.93	595.92	520.59	639	712.26	441.97	336	363.72	12.4551	13.042385	10.576417	10.372466	11.850114	10.448154	7.0749997	7.7713127	10.367053	6.7404652	4.539944	5.2213039	-0.142818	3.1984773	0.1573779	0.6065485	-0.519427	3.1984773	2.88E-07	2.85E-06	-1.131887	3.1984773	1.41E-27	1.42E-26
19282	ENSMUSG00	18111	Nnat	3	protein codi	MGI Symbol	10	neuronatin [	1189	1162	1125	1373	1388	1406	121	139	152	181	296	246	21.125698	20.184665	19.999444	22.569922	22.720371	24.651134	1.6444322	1.6904733	2.2123831	2.7604231	3.9994744	3.5313999	0.189823	3.5971787	0.047236	0.3473237	-3.471055	3.5971787	1.85E-194	1.35E-191	-2.569586	3.5971787	4.38E-124	3.62E-122
19808	ENSMUSG00	54710	Hs3st3b1	3	protein codi	MGI Symbol	1	heparan sulf	288.69	258.99	194.4	281.96	247.1	270.16	222.28	224.69	186.72	126.06	133.66	120.34	5.1293337	4.4988178	3.4559039	4.6349711	4.0448153	4.7366646	3.0208628	2.7326076	2.717738	1.9225356	1.8059789	1.7275149	0.0352135	1.7454129	0.7711825	0.9614588	-0.627204	1.7454129	1.49E-07	1.55E-06	-1.262208	1.7454129	3.23E-23	2.55E-22
19837	ENSMUSG00	208151	Tmem132b	3	protein codi	MGI Symbol	3	transmembr	1400	1167	1734	1222	1280	1256	941	1146	914	231	234	236	24.874666	20.271518	30.825809	20.087724	20.952503	22.021213	12.788518	13.937284	13.303409	3.5229709	3.1617467	3.387847	-0.268627	3.9710071	0.0048398	0.0730973	-0.923722	3.9710071	8.00E-22	2.88E-20	-2.915392	3.9710071	1.18E-157	1.48E-155
20299	ENSMUSG00	74376	Myo18b	3	protein codi	MGI Symbol	4	myosin XVIII	3477	3951.91	3160.97	3684.95	4365.84	4062	2999.88	3623.9	3276	2025.92	1268	1242.99	61.778009	68.647142	56.193459	60.57468	71.46506	71.218285	40.769416	44.072708	47.682678	30.897217	17.132884	17.843474	0.1231004	5.6138666	0.1793429	0.6401309	-0.494008	5.6138666	8.00E-08	8.69E-07	-1.504162	5.6138666	4.55E-57	1.19E-55
20899	ENSMUSG00	270120	Fat3	3	protein codi	MGI Symbol	5	FAT atypical	689	573	756	643	963	758	254	292	373	289	345	324	12.241889	9.9533674	13.439626	10.569891	15.763485	13.289872	3.4519486	3.5512102	5.4290717	4.4075264	4.6615496	4.651112	0.1534316	3.0657998	0.1257825	0.5479994	-1.524862	3.0657998	7.41E-46	6.52E-44	-1.37567	3.0657998	3.39E-38	5.29E-37
20940	ENSMUSG00	633640	Tmem267	3	protein codi	MGI Symbol	5	transmembr	194	240.68	169	534.5	682.53	975.3	183	221	241.77	201	261.65	209	3.4469179	4.1807617	3.0043609	8.7863245	11.172431	17.099752	2.4870339	2.687731	3.5189991	3.0654422	3.5353462	3.0002544	1.7975195	2.4467089	6.81E-59	3.08E-56	-0.295995	2.4467089	0.0164379	0.0383497	-0.146129	2.4467089	0.2286779	0.2510041
20942	ENSMUSG00	20674	Sox2	3	protein codi	MGI Symbol	1	SRY (sex det	946	898	1176	1112	1277	1079	736	809	604	364	429	365	16.808167	15.59882	20.906085	18.2795	20.903396	18.917905	10.002497	9.8387981	8.7913119	5.5513481	5.7965356	5.2396787	0.1243703	3.6994403	0.1996811	0.6698342	-0.89566	3.6994403	9.72E-20	3.10E-18	-1.683437	3.6994403	2.90E-61	8.27E-60
2208	ENSMUSG00	103098	Slc6a15	3	protein codi	MGI Symbol	5	solute carrie	932	894	1028	800	823	786	88	86	108	79	126	94	16.55942	15.529338	18.275047	13.15072	13.471805	13.780791	1.1959507	1.0459044	1.5719564	1.2048255	1.702479	1.3493967	-0.317868	3.0137195	0.0012148	0.0237454	-3.732157	3.0137195	5.81E-202	5.02E-199	-3.556146	3.0137195	9.15E-188	1.74E-185
22689	ENSMUSG00	100043899	R3hdml	3	protein codi	MGI Symbol	1	R3H domain	1560	1618	1709	1911	2216	1896	1082	1096	1033	941	1274	1152	27.717484	28.105669	30.381377	31.413781	36.274021	33.242213	14.704758	13.3292	15.035472	14.35115	17.213954	16.537287	0.2275702	4.5321125	0.0154064	0.1695953	-1.00172	4.5321125	1.41E-25	5.94E-24	-0.840853	4.5321125	1.29E-18	7.67E-18
23017	ENSMUSG00	101359	Prrt4	3	protein codi	MGI Symbol	1	proline-rich	1718	1542	1604	1657	1847	1917	519	552	494	574	700	767	30.524768	26.785502	28.514762	27.238428	30.233808	33.610402	7.0533911	6.7132467	7.1902451	8.7540489	9.4582166	11.010503	0.0855446	4.2334267	0.3637813	0.8050619	-2.034366	4.2334267	4.20E-93	9.72E-91	-1.553605	4.2334267	7.86E-57	2.04E-55
2640	ENSMUSG00	58185	Rsad2	3	protein codi	MGI Symbol	3	radical S-ade	812	896	935	731	915	747	226	351	246	91	76	71	14.427306	15.564079	16.62176	12.01647	14.977766	13.097011	3.0714189	4.2687492	3.5805674	1.387837	1.0268921	1.0192252	-0.217263	3.0527136	0.0276011	0.2485464	-2.089338	3.0527136	3.03E-84	5.87E-82	-3.766752	3.0527136	4.21E-196	8.51E-194
2751	ENSMUSG00	216881	Wscd1	3	protein codi	MGI Symbol	6	WSC domain	262	303	304	289	273	276	107	113	93	142	206	156	4.655116	5.263299	5.4042941	4.7506974	4.4687761	4.8390563	1.4541673	1.3742697	1.3536291	2.1656358	2.783418	2.2394243	-0.124713	1.7447386	0.2774626	0.7468832	-1.871961	1.7447386	7.04E-48	6.55E-46	-1.087594	1.7447386	1.44E-19	9.15E-19
2775	ENSMUSG00	13447	Doc2b	3	protein codi	MGI Symbol	1	double C2, b	383.72	509.78	333.91	569.43	635.88	555.89	278.85	387.59	270	126.93	125.93	112.9	6.8177905	8.8551966	5.9360127	9.3605178	10.408811	9.7463152	3.7896688	4.7137451	3.9298911	1.9358039	1.7015332	1.6207116	0.4490719	2.5070132	1.70E-05	0.000574	-0.794685	2.5070132	3.99E-13	7.99E-12	-2.040829	2.5070132	2.18E-62	6.34E-61
27784	ENSMUSG00	NA	Gm12408	3	lincRNA	MGI Symbol	6	predicted ge	262	270	312	291	283	305	266	307	369	143	139	79	4.655116	4.6900684	5.5465124	4.7835742	4.6324675	5.3475078	3.6150328	3.7336354	5.3708511	2.1808868	1.8781316	1.1340674	-0.013267	1.9834857	0.9177057	0.9915152	-0.232529	1.9834857	0.041942	0.080723	-1.521499	1.9834857	2.22E-33	2.87E-32
31079	ENSMUSG00	74319	Mettl23	3	protein codi	MGI Symbol	8	methyltrans	360.56	379.55	389.81	867.14	910.73	906.77	524.25	617.2	458.24	635.52	676.1	532.77	6.4062924	6.5930202	6.9297628	14.254394	14.90787	15.898228	7.1247404	7.5061881	6.6697529	9.6922877	9.135286	7.6480646	1.1764591	3.2330564	2.08E-30	6.19E-28	0.0972659	3.2330564	0.3662605	0.458555	0.409296	3.2330564	9.58E-05	0.0001478
3355	ENSMUSG00	24056	Sh3bp5	3	protein codi	MGI Symbol	4	SH3-domain	638	590	667	859	790.69	815.63	366	427	355	164	219	241	11.33574	10.248668	11.857448	14.120585	12.942918	14.300288	4.9740677	5.1930368	5.167079	2.5011568	2.9590706	3.4596234	0.3066285	3.0325335	0.0021737	0.0391105	-1.124274	3.0325335	3.82E-27	1.71E-25	-1.903629	3.0325335	3.71E-65	1.13E-63
3367	ENSMUSG00	NA	Ncoa4-ps	3	processed p	MGI Symbol	1	nuclear rece	147.71	173.52	164.51	863.08	874.36	851	276.03	342.49	252.86	185.2	263.41	254.36	2.6244549	3.0141506	2.9245409	14.187654	14.312524	14.920423	3.751344	4.1652533	3.6804158	2.8244771	3.5591269	3.65141	2.34082	2.6108626	2.79E-94	1.66E-91	0.4391081	2.6108626	0.0003021	0.0014287	0.2310933	2.6108626	0.0651628	0.0759939
34420	ENSMUSG00	622645	Tmem200c	3	protein codi	MGI Symbol	2	transmembr	338	298	350	285	317	321	196	221	202	168	230	191	6.005455	5.1764459	6.2220492	4.6849438	5.1890184	5.6280328	2.6637084	2.687731	2.9401407	2.5621607	3.1076997	2.7418592	-0.16748	2.0373284	0.1365245	0.5693514	-1.070399	2.0373284	3.53E-20	1.15E-18	-1.045356	2.0373284	3.64E-19	2.24E-18
36310	ENSMUSG00	NA	E530011L22	3	antisense	MGI Symbol	2	RIKEN cDNA	331.74	227.15	200.77	536.31	682.94	581.54	377.51	332.27	272.4	332.1	384.55	392.04	5.8942297	3.9457372	3.5691452	8.816078	11.179143	10.196032	5.1304926	4.040961	3.9648234	5.0648426	5.1959388	5.6278455	1.1709843	2.5972636	7.59E-27	1.90E-24	-0.029462	2.5972636	0.8020481	0.8494378	0.2454121	2.5972636	0.0301381	0.0362923
3720	ENSMUSG00	14663	Glycam1	3	protein codi	MGI Symbol	4	glycosylation	417	369	421	434	429	372	143	104	125	159	322	335	7.4090968	6.4097602	7.4842363	7.1342653	7.0223624	6.5222063	1.9434199	1.2648146	1.819394	2.4249021	4.3507796	4.8090201	-0.042066	2.2713993	0.6992379	0.9427938	-2.090856	2.2713993	3.64E-65	5.24E-63	-0.872513	2.2713993	3.60E-15	1.63E-14
3865	ENSMUSG00	224023	Klhl22	3	protein codi	MGI Symbol	7	kelch-like 22	7024	7330	8775	11280	11327	10404	8425	11172	9766	2739	2670	2096	124.79975	127.32667	155.99566	185.42515	185.41329	182.41138	114.49869	135.87027	142.14562	41.772369	36.07634	30.088675	0.4389458	6.9281641	1.27E-06	5.44E-05	-0.056255	6.9281641	0.5345314	0.6193497	-1.919038	6.9281641	1.98E-92	9.39E-91
3880	ENSMUSG00	15160	Serpind1	3	protein codi	MGI Symbol	2	serine (or cy	786	694	1020	679	762	708	1121	1575	1057	367	430	426	13.965348	12.055213	18.132829	11.161673	12.473287	12.413231	15.234781	19.154644	15.384796	5.597101	5.8100473	6.115351	-0.292389	3.6226149	0.0032383	0.0536574	0.1744846	3.6226149	0.0741087	0.1278897	-1.332353	3.6226149	9.33E-39	1.49E-37
4163	ENSMUSG00	100037282	Rsph3b	3	protein codi	MGI Symbol	3	radial spoke	896.28	857.14	886.25	2054.87	2141.83	1930.85	1247.9	1390.33	1001.52	1269.41	1666.86	1483.22	15.924761	14.889056	15.755117	33.778774	35.059922	33.853231	16.959396	16.908747	14.577276	19.359716	22.522176	21.292044	1.140878	4.4427455	1.58E-32	5.02E-30	0.0577821	4.4427455	0.5570687	0.6400765	0.4404486	4.4427455	5.04E-06	8.86E-06
4215	ENSMUSG00	224796	Clic5	3	protein codi	MGI Symbol	3	chloride intr	7074	9109	5280	6595	7630	7071	4198	3868	3943	6410	5827	5381	125.68813	158.22901	93.864056	108.41124	124.89656	123.97452	57.052285	47.041373	57.390965	97.75863	78.732897	77.245783	-0.08058	6.5819466	0.3748309	0.812109	-1.226554	6.5819466	2.27E-40	1.71E-38	-0.57446	6.5819466	2.82E-10	8.04E-10
42913	ENSMUSG00	NA	Gm43718	3	TEC	MGI Symbol	1	predicted ge	619	592	612	661	600	630	176	229	236	33	29	25	10.998156	10.283409	10.879697	10.865782	9.8214859	11.045672	2.3919014	2.7850244	3.4350159	0.5032816	0.3918404	0.3588821	-0.019568	2.5927635	0.8537078	0.9820332	-1.90374	2.5927635	5.12E-65	7.25E-63	-4.680616	2.5927635	1.18E-212	2.81E-210
43269	ENSMUSG00	NA	Sox2ot	3	processed t	MGI Symbol	18	SOX2 overla	883	773.46	724	7049	7032	7003	576	698	510	266	314	282	15.688807	13.435483	12.870753	115.87428	115.10782	122.78229	7.828041	8.4888518	7.4231276	4.0567544	4.2426857	4.0481901	3.0743447	5.1637089	5.72E-211	6.80E-208	-0.821339	5.1637089	2.68E-16	6.82E-15	-1.764929	5.1637089	4.69E-61	1.34E-59
4422	ENSMUSG00	77805	Esco1	3	protein codi	MGI Symbol	9	establishme	437	434.29	441.58	871.61	1011.49	925.12	601.48	843.44	658.88	615.71	635	643.71	7.7644492	7.5438882	7.8500928	14.327873	16.557225	16.219956	8.1743231	10.257646	9.5900986	9.3901663	8.5799536	9.2406398	1.0240675	3.388538	6.10E-24	1.32E-21	0.2758712	3.388538	0.0076344	0.0206351	0.2320988	3.388538	0.0252862	0.0306402
464	ENSMUSG00	16822	Lcp2	3	protein codi	MGI Symbol	4	lymphocyte	604	528	580	510	533	524	398	540	381	93	135	136	10.731641	9.1716893	10.310824	8.3835837	8.7247533	9.1871938	5.4089589	6.5673065	5.5455129	1.4183389	1.8240846	1.9523186	-0.20051	2.7124313	0.0522062	0.3636061	-0.78332	2.7124313	4.50E-14	9.61E-13	-2.534522	2.7124313	1.07E-98	5.57E-97
4708	ENSMUSG00	104174	Gldc	3	protein codi	MGI Symbol	3	glycine deca	1238	1095	1195	2819	2728	2643	1190	1328	1251	978	1109	1025	21.996311	19.020833	21.243854	46.339848	44.655023	46.339224	16.172515	16.150709	18.208495	14.915435	14.984517	14.714166	1.1412985	4.615719	2.06E-33	6.74E-31	-0.301585	4.615719	0.0016811	0.0060175	-0.480639	4.615719	6.33E-07	1.23E-06
4749	ENSMUSG00	12894	Cpt1a	3	protein codi	MGI Symbol	8	carnitine pal	10896	11027	9650	11044	12248	10890	5983	6929	6192	6574	6449	7024	193.59597	191.54587	171.55078	181.54568	200.48927	190.93233	81.311058	84.268272	90.125502	100.25979	87.137198	100.83151	0.041567	7.1230477	0.6455732	0.9268112	-1.122458	7.1230477	1.48E-34	8.78E-33	-0.949759	7.1230477	2.27E-25	2.02E-24
480	ENSMUSG00	110175	Ggct	3	protein codi	MGI Symbol	6	gamma-glut	6893	6211	7863	7794	8404	7419	7254	9695	8027	4310	3529	3942	122.47219	107.88894	139.78278	128.12088	137.56628	130.07594	98.584391	117.90748	116.83421	65.731622	47.682923	56.588529	0.0965941	6.7245449	0.2869366	0.7516196	-0.151088	6.7245449	0.0955209	0.1565377	-1.122854	6.7245449	3.21E-34	4.27E-33
4820	ENSMUSG00	20411	Sorbs1	3	protein codi	MGI Symbol	27	sorbin and S	22992	24219	21914	24870	28151.94	25369	18290.97	25155	21888	13228	10781	10943	408.51308	420.69914	389.57139	408.82299	460.82314	444.78992	248.58066	305.92703	318.58317	201.73965	145.67005	157.08987	0.108996	8.3480849	0.2254614	0.7054191	-0.481244	8.3480849	9.33E-08	1.01E-06	-1.272652	8.3480849	4.78E-44	9.01E-43
5117	ENSMUSG00	105445	Dock9	3	protein codi	MGI Symbol	12	dedicator of	533	385	497	505	502	532	227	325	286	542	553	544	9.4701405	6.6876901	8.8353098	8.3013917	8.2173099	9.3274563	3.0850092	3.9525456	4.1627735	8.2660183	7.4719911	7.8092745	0.0483104	2.8302213	0.6491507	0.9276372	-1.157288	2.8302213	6.58E-26	2.80E-24	-0.086081	2.8302213	0.4125441	0.4386311
5200	ENSMUSG00	20450	St8sia2	3	protein codi	MGI Symbol	2	ST8 alpha-N-	2098	1801	2125	2095	2277	2171	1759	1545	1295	591	773	695	37.276463	31.284493	37.776727	34.438447	37.272539	38.063736	23.905424	18.789794	18.848922	9.0133152	10.444573	9.9769224	0.0459025	4.6737252	0.6242012	0.9187451	-0.78896	4.6737252	5.86E-17	1.54E-15	-1.851887	4.6737252	3.89E-80	1.51E-78
5240	ENSMUSG00	17919	Myo5b	3	protein codi	MGI Symbol	11	myosin VB [	3253	3195	3551	2742	2988	2885	2444	2842	2423	1498	1505	1370	57.798062	55.499143	63.127133	45.074091	48.911	50.582164	33.214813	34.563491	35.267134	22.845933	20.335166	19.666739	-0.287353	5.3405073	0.0018229	0.0336261	-0.775743	5.3405073	5.51E-17	1.46E-15	-1.48937	5.3405073	6.14E-56	1.56E-54
53362	ENSMUSG00	NA	AC113595.1	3	antisense	Clone-based	1	novel transc	1374.61	1530.58	1665.04	1808.33	2096.05	1905.47	1575.56	2249.84	2050.45	914.21	752.8	743.94	24.423546	26.587129	29.599888	29.726051	34.310543	33.408248	21.41241	27.361831	29.844612	13.942577	10.171636	10.67947	0.2735473	4.6013506	0.0036602	0.0589348	-0.036279	4.6013506	0.7025868	0.7676337	-1.213669	4.6013506	7.74E-36	1.11E-34
5366	ENSMUSG00	109019	Nabp1	3	protein codi	MGI Symbol	10	nucleic acid	771	642	765	781	970	796	625	838	631	354	341	369	13.698834	11.15194	13.599622	12.83839	15.878069	13.956119	8.4939681	10.191487	9.184301	5.3988385	4.6075026	5.2970998	0.1506999	3.3685493	0.1299398	0.5560487	-0.462858	3.3685493	3.61E-06	2.87E-05	-1.330008	3.3685493	1.84E-37	2.81E-36
53675	ENSMUSG00	NA	Gm49463	3	antisense	MGI Symbol	1	predicted ge	272.38	296.4	328.62	284.26	342	371.99	210.13	284.12	177.37	147.76	152.31	165.42	4.8395439	5.1486529	5.8419709	4.6727794	5.598247	6.5220309	2.85574	3.4553762	2.5816473	2.2534813	2.0579728	2.3746511	0.0835062	1.9962958	0.4575238	0.8564485	-0.825751	1.9962958	1.02E-12	1.96E-11	-1.24394	1.9962958	1.36E-24	1.16E-23
53749	ENSMUSG00	224098	Gm536	3	lincRNA	MGI Symbol	2	predicted ge	270	213	240	317	349	328	189	245	182	143	117	135	4.7972569	3.6999429	4.266548	5.2109726	5.712831	5.7507625	2.5685759	2.9796113	2.6490377	2.1808868	1.5808733	1.9379633	0.3856674	1.8432302	0.0007707	0.0160211	-0.63523	1.8432302	1.17E-07	1.24E-06	-1.166885	1.8432302	3.85E-20	2.52E-19
5386	ENSMUSG00	12839	Col9a1	3	protein codi	MGI Symbol	4	collagen, typ	234	209	316	202	193	179	137	159	151	309	187	177	4.1576227	3.6304604	5.6176215	3.3205567	3.1592446	3.1383734	1.8618778	1.9337069	2.197828	4.7125455	2.526695	2.5408853	-0.477589	1.6868608	7.10E-05	0.0020013	-1.162475	1.6868608	7.56E-21	2.60E-19	-0.462326	1.6868608	0.0001112	0.0001704
5458	ENSMUSG00	53972	Ngef	3	protein codi	MGI Symbol	4	neuronal gu	4832.85	4768.92	4813.89	4957.78	5262.8	5053.5	3379.96	3889.84	3215.94	2318.98	2313.99	2155.94	85.868234	82.839115	85.577886	81.497968	86.147527	88.602068	45.934836	47.306984	46.808496	35.366663	31.266027	30.949131	0.0110611	5.9607133	0.9041242	0.9900784	-0.860439	5.9607133	8.65E-21	2.95E-19	-1.381964	5.9607133	1.24E-49	2.67E-48
5606	ENSMUSG00	20739	Spta1	3	protein codi	MGI Symbol	2	spectrin alph	422	363	410	405	540	466	381	536	424	270	214	246	7.4979349	6.3055364	7.2886862	6.6575518	8.8393373	8.1702906	5.1779229	6.5186598	6.1713845	4.1177582	2.8915119	3.5313999	0.1662411	2.6063695	0.1193519	0.5363903	-0.237874	2.6063695	0.0256996	0.0546434	-1.004209	2.6063695	5.49E-19	3.34E-18
5905	ENSMUSG00	73649	Cybrd1	3	protein codi	MGI Symbol	1	cytochrome	213	225	210	255	303	240	117	151	118	98	98	70	3.7845027	3.9083903	3.7332295	4.1917918	4.9598504	4.207875	1.5900708	1.8364135	1.7175079	1.4945937	1.3241503	1.0048699	0.2260505	1.4744648	0.0584408	0.3855467	-1.148472	1.4744648	4.71E-19	1.46E-17	-1.580498	1.4744648	3.68E-31	4.39E-30
6161	ENSMUSG00	67017	Fam210b	3	protein codi	MGI Symbol	1	family with s	2768	2531	2727	2585	2738	2542	2582	2740	2228	1142	1314	1177	49.180767	43.965049	48.478652	42.493262	44.818714	44.568409	35.090281	33.323	32.428879	17.416592	17.754424	16.896169	-0.102815	5.1496158	0.2669628	0.7370376	-0.489853	5.1496158	1.26E-07	1.33E-06	-1.443406	5.1496158	4.75E-52	1.08E-50
6409	ENSMUSG00	68659	Gask1b	3	protein codi	MGI Symbol	5	golgi associa	1697.14	1424.12	1682.54	1432.57	1432.88	1352	1183.38	1187.31	1011.79	423.66	475.68	457	30.154136	24.737853	29.91099	23.549158	23.455018	23.704363	16.082547	14.439683	14.726757	6.4612201	6.4272635	6.5603648	-0.262096	4.1922843	0.0056641	0.0827823	-0.906198	4.1922843	2.59E-21	9.13E-20	-2.123999	4.1922843	7.15E-100	3.84E-98
6818	ENSMUSG00	27028	Ermap	3	protein codi	MGI Symbol	10	erythroblast	2525	1804	2312	1781	2185	1811	2087	2587	2753	1689	1055	1116	44.863236	31.336605	41.101079	29.276789	35.766578	31.751924	28.363058	31.462263	40.070334	25.758865	14.254884	16.020497	-0.276973	4.9458277	0.0030067	0.0508249	-0.232192	4.9458277	0.0126056	0.0309201	-1.067717	4.9458277	2.17E-29	2.39E-28
7042	ENSMUSG00	71890	Mad2l2	3	protein codi	MGI Symbol	8	MAD2 mitot	228	208	264	205	210	184	146	163	152	165	219	172	4.051017	3.6130897	4.6932028	3.3698719	3.4375201	3.2260375	1.9841909	1.9823536	2.2123831	2.5164078	2.9590706	2.4691088	-0.299257	1.6014116	0.0132021	0.1522088	-1.001042	1.6014116	1.04E-15	2.52E-14	-0.634006	1.6014116	1.87E-07	3.85E-07
7141	ENSMUSG00	100952	Emilin1	3	protein codi	MGI Symbol	3	elastin micro	1623	1400	1735	1431	1406	1488	910	1026	788	378	508	451	28.836844	24.318873	30.843587	23.52335	23.015015	26.088825	12.367218	12.477882	11.46946	5.7648615	6.8639629	6.4742331	-0.209978	4.1361459	0.0267005	0.2441335	-1.209014	4.1361459	5.21E-36	3.34E-34	-2.134676	4.1361459	9.43E-101	5.21E-99
7178	ENSMUSG00	14397	Gabra4	3	protein codi	MGI Symbol	5	gamma-amin	204	192	206	215	235	214	228	269	207	124	103	84	3.6245941	3.3351598	3.6621204	3.5342559	3.8467487	3.7520219	3.0985996	3.2714916	3.0129165	1.8911186	1.391709	1.2058439	0.0679451	1.5717745	0.5972096	0.909608	-0.176964	1.5717745	0.1441895	0.2166034	-1.247328	1.5717745	1.31E-20	8.87E-20
7194	ENSMUSG00	13839	Epha5	3	protein codi	MGI Symbol	11	Eph receptor	285	281	281	320	364	343	151	137	124	295	280	258	5.0637712	4.8811453	4.9954166	5.2602878	5.9583681	6.0137547	2.0521427	1.66615	1.8048389	4.4990321	3.7832866	3.7036633	0.2054468	2.0396508	0.0681549	0.4127435	-1.437114	2.0396508	5.15E-31	2.63E-29	-0.319647	2.0396508	0.0053788	0.0069277
745	ENSMUSG00	268379	Abca13	3	protein codi	MGI Symbol	3	ATP-binding	896	561	1159	681	1023	866	16	18	37	3	7	0	15.919786	9.7449199	20.603871	11.19455	16.745634	15.183416	0.2174456	0.2189102	0.5385406	0.0457529	0.0945822	0	-0.101107	2.8791369	0.3047615	0.7643114	-5.594048	2.8791369	2.28E-294	4.33E-291	-8.275725	2.8791369	0	0
7558	ENSMUSG00	76156	Fam131b	3	protein codi	MGI Symbol	3	family with s	721.04	633.39	1036.18	1171.77	1250.6	909.74	387.51	628.39	341.28	186.51	182.45	183.61	12.811163	11.002379	18.420465	19.262023	20.471251	15.950301	5.2663961	7.6422773	4.9673823	2.8444559	2.4652166	2.6357737	0.3998518	3.3541789	4.36E-05	0.0013313	-1.233986	3.3541789	6.65E-34	3.78E-32	-2.410912	3.3541789	1.33E-101	7.50E-100
76	ENSMUSG00	14526	Gcg	3	protein codi	MGI Symbol	3	glucagon [Sc	2862	4564	1965	1610	1458	1621	674	684	895	619	880	835	50.850923	79.279527	34.932362	26.465823	23.866211	28.420689	9.1598952	8.3185882	13.026861	9.440342	11.890329	11.986662	-1.068083	4.6722835	5.29E-30	1.52E-27	-2.43946	4.6722835	1.23E-136	5.29E-134	-2.307541	4.6722835	1.06E-123	8.66E-122
7644	ENSMUSG00	54563	Nup210	3	protein codi	MGI Symbol	11	nucleoporin	693	577	638	638	630	619	324	345	322	210	229	227	12.312959	10.02285	11.341907	10.487699	10.31256	10.852811	4.4032731	4.1957792	4.686759	3.2027008	3.094188	3.2586495	-0.089385	2.8630945	0.3804841	0.8167045	-1.342908	2.8630945	1.92E-36	1.26E-34	-1.816928	2.8630945	2.45E-60	6.82E-59
7772	ENSMUSG00	243616	Slc6a11	3	protein codi	MGI Symbol	2	solute carrie	341	245	353	307	331	351	251	303	204.99	152	135	99	6.0587578	4.2558028	6.275381	5.0465886	5.4181864	6.1540172	3.4111776	3.6849887	2.9836606	2.3181454	1.8240846	1.4211731	0.0022059	2.0153697	0.9937663	1	-0.714016	2.0153697	3.86E-10	5.57E-09	-1.578772	2.0153697	5.08E-37	7.65E-36
8100	ENSMUSG00	12426	Cckbr	3	protein codi	MGI Symbol	2	cholecystoki	954	780	980	838	864	865	1135	1538	1211	380	361	348	16.950308	13.549087	17.421738	13.775379	14.14294	15.165883	15.425046	18.704662	17.626289	5.7953634	4.8777374	4.9956388	-0.153513	3.7236889	0.1185711	0.5353037	0.1117471	3.7236889	0.2506154	0.3384288	-1.613791	3.7236889	1.51E-55	3.81E-54
8160	ENSMUSG00	101772	Ano1	3	protein codi	MGI Symbol	11	anoctamin 1	561	395	547	610	647	583	313	252	296	487	653	566	9.9676338	6.8613964	9.724174	10.027424	10.590836	10.22163	4.2537792	3.064743	4.308325	7.4272157	8.8231649	8.1250907	0.216547	2.9554552	0.0359019	0.2966087	-1.195949	2.9554552	8.16E-28	3.73E-26	-0.121626	2.9554552	0.2383696	0.2608583
8429	ENSMUSG00	11733	Ank1	3	protein codi	MGI Symbol	17	ankyrin 1, er	2363.19	1831.5	1832.33	1977.48	2595.12	1964.47	1389.61	1650.95	2158.14	1148.92	568.89	879.81	41.988258	31.814297	32.57385	32.506606	42.479891	34.442684	18.885285	20.078323	31.412056	17.522129	7.6866926	12.629922	0.0410562	4.7518971	0.6622717	0.9313962	-0.597622	4.7518971	2.08E-10	3.08E-09	-1.494757	4.7518971	5.86E-54	1.41E-52
9188	ENSMUSG00	20501	Slc16a1	3	protein codi	MGI Symbol	1	solute carrie	603	545	660	502	564	576	425	349	314	572	772	639	10.713874	9.4669899	11.733007	8.2520765	9.2321968	10.0989	5.7758983	4.2444259	4.5703178	8.723547	10.431062	9.1730265	-0.210812	3.0905462	0.0401072	0.3175663	-1.130073	3.0905462	4.83E-27	2.15E-25	-0.170575	3.0905462	0.09183	0.1053478
9320	ENSMUSG00	81877	Tnxb	3	protein codi	MGI Symbol	4	tenascin XB	454	379	496.94	449	530.96	535	223	324	285	415.95	458	443	8.0664987	6.5834664	8.8342432	7.3808413	8.6913603	9.3800547	3.0306478	3.9403839	4.1482183	6.3436353	6.188376	6.3593908	0.1157383	2.7122128	0.2724567	0.742133	-1.077993	2.7122128	1.58E-22	5.79E-21	-0.313581	2.7122128	0.0030945	0.0040887

Table S3. Enriched GO categories for genes within each cluster in Fig. 9A.

Analysis Type:	PANTHER Overrepresentation Test (Released 20230705)
Annotation Version and Release Date:	GO Ontology database DOI: 10.5281/zenodo.7942786 Released 2023-05-10
Analyzed List:	upload_1 (Mus musculus)
Reference List:	Mus musculus (all genes in database)
Test Type:	FISHER
Correction:	FDR

GO biological process complete	Mus musculu	upload_1 (91	upload_1 (ex	upload_1 (ov	upload_1 (fo	upload_1 (ra	upload_1 (FDR)
activation of meiosis involved in egg activation (GO:0060466)	3	3	0.12	+	24.04	1.16E-03	2.52E-02
calcium ion export (GO:1901660)	4	3	0.17	+	18.03	1.97E-03	3.80E-02
positive regulation of plasma membrane repair (GO:1905686)	4	3	0.17	+	18.03	1.97E-03	3.80E-02
negative regulation of cardiac muscle contraction (GO:0055118)	4	3	0.17	+	18.03	1.97E-03	3.79E-02
positive regulation of extracellular exosome assembly (GO:1903553)	4	3	0.17	+	18.03	1.97E-03	3.79E-02
positive regulation of protein folding (GO:1903334)	4	3	0.17	+	18.03	1.97E-03	3.78E-02
modulation of microtubule cytoskeleton involved in cerebral cortex radial glia guided migration (GO:0021815)	4	3	0.17	+	18.03	1.97E-03	3.78E-02
actomyosin contractile ring organization (GO:0044837)	8	5	0.33	+	15.03	9.89E-05	3.28E-03
actomyosin contractile ring assembly (GO:0000915)	8	5	0.33	+	15.03	9.89E-05	3.27E-03
assembly of actomyosin apparatus involved in cytokinesis (GO:0000912)	8	5	0.33	+	15.03	9.89E-05	3.26E-03
activation of meiosis (GO:0090427)	7	4	0.29	+	13.74	6.66E-04	1.62E-02
regulation of plasma membrane repair (GO:1905684)	7	4	0.29	+	13.74	6.66E-04	1.61E-02
negative regulation of hypoxia-induced intrinsic apoptotic signaling pathway (GO:1903298)	8	4	0.33	+	12.02	9.67E-04	2.17E-02
regulation of hypoxia-induced intrinsic apoptotic signaling pathway (GO:1903297)	8	4	0.33	+	12.02	9.67E-04	2.16E-02
growth hormone receptor signaling pathway via JAK-STAT (GO:0060397)	8	4	0.33	+	12.02	9.67E-04	2.16E-02
metanephric glomerulus development (GO:0072224)	8	4	0.33	+	12.02	9.67E-04	2.16E-02
peptidyl-proline hydroxylation (GO:0019511)	14	7	0.58	+	12.02	1.13E-05	5.10E-04
peptidyl-proline hydroxylation to 4-hydroxy-L-proline (GO:0018401)	11	5	0.46	+	10.93	3.04E-04	8.63E-03
regulation of neutrophil apoptotic process (GO:0033029)	9	4	0.37	+	10.68	1.35E-03	2.85E-02
positive regulation of aspartic-type peptidase activity (GO:1905247)	9	4	0.37	+	10.68	1.35E-03	2.84E-02
regulation of cardiac muscle cell membrane potential (GO:0086036)	9	4	0.37	+	10.68	1.35E-03	2.84E-02
NADH regeneration (GO:0006735)	16	7	0.67	+	10.52	2.21E-05	9.18E-04
glucose catabolic process to pyruvate (GO:0061718)	16	7	0.67	+	10.52	2.21E-05	9.16E-04
canonical glycolysis (GO:0061621)	16	7	0.67	+	10.52	2.21E-05	9.13E-04
regulation of aspartic-type peptidase activity (GO:1905245)	12	5	0.5	+	10.02	4.16E-04	1.11E-02
purine ribonucleoside biosynthetic process (GO:0046129)	10	4	0.42	+	9.62	1.83E-03	3.63E-02
phospholipase C-activating G protein-coupled acetylcholine receptor signaling pathway (GO:0007207)	10	4	0.42	+	9.62	1.83E-03	3.62E-02
cellular response to interleukin-12 (GO:0071349)	10	4	0.42	+	9.62	1.83E-03	3.62E-02
ribonucleoside biosynthetic process (GO:0042455)	10	4	0.42	+	9.62	1.83E-03	3.61E-02
purine nucleoside biosynthetic process (GO:0042451)	10	4	0.42	+	9.62	1.83E-03	3.61E-02
glycolytic process through glucose-6-phosphate (GO:0061620)	19	7	0.79	+	8.86	5.35E-05	1.97E-03
hyaluronan catabolic process (GO:0030214)	19	7	0.79	+	8.86	5.35E-05	1.96E-03
inositol trisphosphate metabolic process (GO:0032957)	11	4	0.46	+	8.74	2.42E-03	4.41E-02
glucose catabolic process (GO:0006007)	22	8	0.92	+	8.74	1.68E-05	7.15E-04
regulation of aspartic-type endopeptidase activity involved in amyloid precursor protein catabolic process (GO:1902959)	11	4	0.46	+	8.74	2.42E-03	4.41E-02
egg activation (GO:0007343)	14	5	0.58	+	8.59	7.32E-04	1.74E-02
glycosylceramide metabolic process (GO:0006677)	15	5	0.62	+	8.01	9.44E-04	2.13E-02
glycolytic process through fructose-6-phosphate (GO:0061615)	21	7	0.87	+	8.01	8.98E-05	3.05E-03
ganglioside metabolic process (GO:0001573)	28	9	1.16	+	7.73	1.13E-05	5.09E-04
protein hydroxylation (GO:0018126)	28	9	1.16	+	7.73	1.13E-05	5.08E-04
negative regulation of endoplasmic reticulum stress-induced intrinsic apoptotic signaling pathway (GO:1902236)	19	6	0.79	+	7.59	3.68E-04	1.00E-02
insulin metabolic process (GO:1901142)	19	6	0.79	+	7.59	3.68E-04	1.00E-02

Table S3. Enriched GO categories for genes within each cluster in Fig. 9A.

N-acetylglucosamine metabolic process (GO:0006044)	16	5	0.67	+	7.51	1.20E-03	2.58E-02
negative regulation of vascular permeability (GO:0043116)	20	6	0.83	+	7.21	4.62E-04	1.21E-02
ganglioside biosynthetic process (GO:0001574)	17	5	0.71	+	7.07	1.50E-03	3.09E-02
negative regulation of cellular response to hypoxia (GO:1900038)	17	5	0.71	+	7.07	1.50E-03	3.08E-02
regulation of endoplasmic reticulum stress-induced intrinsic apoptotic signaling pathway (GO:1902235)	31	9	1.29	+	6.98	2.22E-05	9.15E-04
glutamine metabolic process (GO:0006541)	21	6	0.87	+	6.87	5.75E-04	1.43E-02
regulation of cellular response to hypoxia (GO:1900037)	25	7	1.04	+	6.73	2.22E-04	6.47E-03
positive regulation of fibroblast migration (GO:0010763)	25	7	1.04	+	6.73	2.22E-04	6.46E-03
glomerulus vasculature development (GO:0072012)	18	5	0.75	+	6.68	1.85E-03	3.64E-02
receptor catabolic process (GO:0032801)	18	5	0.75	+	6.68	1.85E-03	3.63E-02
regulation of anoikis (GO:2000209)	22	6	0.92	+	6.56	7.07E-04	1.68E-02
cortical cytoskeleton organization (GO:0030865)	52	14	2.16	+	6.47	2.66E-07	1.99E-05
cortical actin cytoskeleton organization (GO:0030866)	41	11	1.71	+	6.45	5.22E-06	2.67E-04
monosaccharide catabolic process (GO:0046365)	41	11	1.71	+	6.45	5.22E-06	2.67E-04
hexose catabolic process (GO:0019320)	34	9	1.41	+	6.36	4.12E-05	1.57E-03
kidney vasculature development (GO:0061440)	19	5	0.79	+	6.33	2.27E-03	4.20E-02
renal system vasculature development (GO:0061437)	19	5	0.79	+	6.33	2.27E-03	4.19E-02
ceramide catabolic process (GO:0046514)	19	5	0.79	+	6.33	2.27E-03	4.19E-02
cell-substrate junction assembly (GO:0007044)	42	11	1.75	+	6.3	6.35E-06	3.16E-04
focal adhesion assembly (GO:0048041)	31	8	1.29	+	6.2	1.28E-04	4.04E-03
glycosphingolipid metabolic process (GO:0006687)	52	13	2.16	+	6.01	1.44E-06	9.09E-05
cell-substrate junction organization (GO:0150115)	44	11	1.83	+	6.01	9.27E-06	4.35E-04
regulation of ryanodine-sensitive calcium-release channel activity (GO:0060314)	20	5	0.83	+	6.01	2.74E-03	4.89E-02
retinal rod cell differentiation (GO:0060221)	20	5	0.83	+	6.01	2.74E-03	4.89E-02
regulation of cellular response to vascular endothelial growth factor stimulus (GO:1902547)	24	6	1	+	6.01	1.04E-03	2.29E-02
cellular response to dexamethasone stimulus (GO:0071549)	24	6	1	+	6.01	1.04E-03	2.29E-02
cell migration involved in sprouting angiogenesis (GO:0002042)	20	5	0.83	+	6.01	2.74E-03	4.88E-02
peptidyl-proline modification (GO:0018208)	41	10	1.71	+	5.86	2.83E-05	1.12E-03
platelet-derived growth factor receptor signaling pathway (GO:0048008)	41	10	1.71	+	5.86	2.83E-05	1.12E-03
response to dexamethasone (GO:0071548)	29	7	1.21	+	5.8	4.79E-04	1.25E-02
endoplasmic reticulum calcium ion homeostasis (GO:0032469)	29	7	1.21	+	5.8	4.79E-04	1.24E-02
vacuolar acidification (GO:0007035)	25	6	1.04	+	5.77	1.25E-03	2.67E-02
negative regulation of protein localization to plasma membrane (GO:1903077)	26	6	1.08	+	5.55	1.48E-03	3.06E-02
cell-cell fusion (GO:0140253)	39	9	1.62	+	5.55	1.02E-04	3.36E-03
syncytium formation by plasma membrane fusion (GO:0000768)	39	9	1.62	+	5.55	1.02E-04	3.35E-03
regulation of vascular permeability (GO:0043114)	48	11	2	+	5.51	1.87E-05	7.88E-04
glycosaminoglycan catabolic process (GO:0006027)	31	7	1.29	+	5.43	6.76E-04	1.63E-02
syncytium formation (GO:0006949)	41	9	1.71	+	5.28	1.43E-04	4.42E-03
ligand-gated ion channel signaling pathway (GO:1990806)	32	7	1.33	+	5.26	7.96E-04	1.86E-02
ER-nucleus signaling pathway (GO:0006984)	28	6	1.16	+	5.15	2.06E-03	3.92E-02
negative regulation of protein localization to cell periphery (GO:1904376)	28	6	1.16	+	5.15	2.06E-03	3.92E-02
synaptic vesicle maturation (GO:0016188)	28	6	1.16	+	5.15	2.06E-03	3.91E-02
negative regulation of sequestering of calcium ion (GO:0051283)	61	13	2.54	+	5.12	6.60E-06	3.26E-04
hyaluronan metabolic process (GO:0030212)	33	7	1.37	+	5.1	9.32E-04	2.11E-02
endoplasmic reticulum unfolded protein response (GO:0030968)	52	11	2.16	+	5.09	3.57E-05	1.38E-03
regulation of cardiac muscle contraction (GO:0055117)	71	15	2.95	+	5.08	1.44E-06	9.09E-05
regulation of actin filament-based movement (GO:1903115)	48	10	2	+	5.01	8.98E-05	3.05E-03
glomerulus development (GO:0032835)	53	11	2.2	+	4.99	4.15E-05	1.58E-03
positive regulation of stress fiber assembly (GO:0051496)	58	12	2.41	+	4.97	1.93E-05	8.05E-04

Table S3. Enriched GO categories for genes within each cluster in Fig. 9A.

myoblast fusion (GO:0007520)	29	6	1.21	+	4.97	2.40E-03	4.38E-02
regulation of cardiac muscle cell contraction (GO:0086004)	39	8	1.62	+	4.93	4.94E-04	1.27E-02
positive regulation of actin filament bundle assembly (GO:0032233)	69	14	2.87	+	4.88	4.83E-06	2.53E-04
chondrocyte development (GO:0002063)	30	6	1.25	+	4.81	2.79E-03	4.96E-02
release of sequestered calcium ion into cytosol (GO:0051209)	60	12	2.5	+	4.81	2.58E-05	1.04E-03
sphingolipid catabolic process (GO:0030149)	30	6	1.25	+	4.81	2.79E-03	4.95E-02
positive regulation of response to endoplasmic reticulum stress (GO:1905898)	35	7	1.46	+	4.81	1.26E-03	2.68E-02
blood vessel endothelial cell migration (GO:0043534)	40	8	1.66	+	4.81	5.73E-04	1.43E-02
glial cell migration (GO:0008347)	50	10	2.08	+	4.81	1.21E-04	3.85E-03
negative regulation of blood coagulation (GO:0030195)	40	8	1.66	+	4.81	5.73E-04	1.43E-02
muscle cell migration (GO:0014812)	35	7	1.46	+	4.81	1.26E-03	2.68E-02
glutamate receptor signaling pathway (GO:0007215)	41	8	1.71	+	4.69	6.61E-04	1.61E-02
negative regulation of hemostasis (GO:1900047)	41	8	1.71	+	4.69	6.61E-04	1.61E-02
negative regulation of blood vessel endothelial cell migration (GO:0043537)	36	7	1.5	+	4.67	1.45E-03	3.00E-02
regulation of stress fiber assembly (GO:0051492)	98	19	4.08	+	4.66	1.90E-07	1.48E-05
receptor metabolic process (GO:0043112)	47	9	1.96	+	4.6	3.48E-04	9.55E-03
macrophage differentiation (GO:0030225)	42	8	1.75	+	4.58	7.59E-04	1.79E-02
positive regulation of collagen biosynthetic process (GO:0032967)	37	7	1.54	+	4.55	1.67E-03	3.37E-02
regulation of long-term neuronal synaptic plasticity (GO:0048169)	37	7	1.54	+	4.55	1.67E-03	3.37E-02
negative regulation of smooth muscle cell migration (GO:0014912)	37	7	1.54	+	4.55	1.67E-03	3.36E-02
columnar/cuboidal epithelial cell development (GO:0002066)	48	9	2	+	4.51	3.99E-04	1.07E-02
protein localization to lysosome (GO:0061462)	48	9	2	+	4.51	3.99E-04	1.07E-02
intracellular pH reduction (GO:0051452)	43	8	1.79	+	4.47	8.70E-04	2.01E-02
negative regulation of coagulation (GO:0050819)	43	8	1.79	+	4.47	8.70E-04	2.00E-02
positive regulation of epithelial to mesenchymal transition (GO:0010718)	54	10	2.25	+	4.45	2.10E-04	6.14E-03
chondrocyte differentiation (GO:0002062)	92	17	3.83	+	4.44	1.49E-06	9.30E-05
regulation of focal adhesion assembly (GO:0051893)	65	12	2.7	+	4.44	5.15E-05	1.91E-03
regulation of cell-substrate junction assembly (GO:0090109)	65	12	2.7	+	4.44	5.15E-05	1.90E-03
regulation of amyloid-beta formation (GO:1902003)	38	7	1.58	+	4.43	1.91E-03	3.71E-02
regulation of striated muscle contraction (GO:0006942)	88	16	3.66	+	4.37	3.62E-06	1.98E-04
collagen fibril organization (GO:0030199)	55	10	2.29	+	4.37	2.39E-04	6.91E-03
positive regulation of cell-matrix adhesion (GO:0001954)	66	12	2.75	+	4.37	5.87E-05	2.12E-03
actin filament bundle organization (GO:0061572)	66	12	2.75	+	4.37	5.87E-05	2.12E-03
neuromuscular junction development (GO:0007528)	50	9	2.08	+	4.33	5.19E-04	1.32E-02
myoblast differentiation (GO:0045445)	39	7	1.62	+	4.31	2.17E-03	4.08E-02
regulation of amyloid precursor protein catabolic process (GO:1902991)	45	8	1.87	+	4.27	1.13E-03	2.46E-02
endothelial cell migration (GO:0043542)	79	14	3.29	+	4.26	1.87E-05	7.91E-04
calcium ion transmembrane import into cytosol (GO:0097553)	96	17	3.99	+	4.26	2.48E-06	1.46E-04
Rho protein signal transduction (GO:0007266)	68	12	2.83	+	4.24	7.57E-05	2.65E-03
regulation of calcineurin-NFAT signaling cascade (GO:0070884)	40	7	1.66	+	4.21	2.47E-03	4.47E-02
aminoglycan catabolic process (GO:0006026)	40	7	1.66	+	4.21	2.47E-03	4.47E-02
positive regulation of collagen metabolic process (GO:0010714)	40	7	1.66	+	4.21	2.47E-03	4.46E-02
actin filament bundle assembly (GO:0051017)	63	11	2.62	+	4.2	1.62E-04	4.90E-03
monosaccharide biosynthetic process (GO:0046364)	63	11	2.62	+	4.2	1.62E-04	4.90E-03
regulation of actin filament bundle assembly (GO:0032231)	115	20	4.78	+	4.18	4.25E-07	3.06E-05
response to ischemia (GO:0002931)	46	8	1.91	+	4.18	1.28E-03	2.72E-02
positive regulation of glial cell differentiation (GO:0045687)	52	9	2.16	+	4.16	6.67E-04	1.61E-02
hexose biosynthetic process (GO:0019319)	52	9	2.16	+	4.16	6.67E-04	1.61E-02
regulation of long-term synaptic potentiation (GO:1900271)	58	10	2.41	+	4.14	3.49E-04	9.57E-03

Table S3. Enriched GO categories for genes within each cluster in Fig. 9A.

cellular response to hypoxia (GO:0071456)	93	16	3.87	+	4.14	6.75E-06	3.32E-04
regulation of cell-substrate junction organization (GO:0150116)	70	12	2.91	+	4.12	9.67E-05	3.23E-03
regulation of actomyosin structure organization (GO:0110020)	111	19	4.62	+	4.12	1.02E-06	6.73E-05
nucleus localization (GO:0051647)	41	7	1.71	+	4.1	2.79E-03	4.96E-02
regulation of calcineurin-mediated signaling (GO:0106056)	41	7	1.71	+	4.1	2.79E-03	4.95E-02
negative regulation of response to endoplasmic reticulum stress (GO:1903573)	41	7	1.71	+	4.1	2.79E-03	4.94E-02
regulation of mesenchymal cell proliferation (GO:0010464)	41	7	1.71	+	4.1	2.79E-03	4.94E-02
negative regulation of cold-induced thermogenesis (GO:0120163)	47	8	1.96	+	4.09	1.45E-03	2.99E-02
response to acetylcholine (GO:1905144)	53	9	2.2	+	4.08	7.53E-04	1.78E-02
oligosaccharide metabolic process (GO:0009311)	53	9	2.2	+	4.08	7.53E-04	1.78E-02
negative regulation of fat cell differentiation (GO:0045599)	59	10	2.45	+	4.07	3.94E-04	1.06E-02
regulation of protein localization to plasma membrane (GO:1903076)	118	20	4.91	+	4.07	6.10E-07	4.25E-05
ceramide biosynthetic process (GO:0046513)	59	10	2.45	+	4.07	3.94E-04	1.06E-02
cellular response to unfolded protein (GO:0034620)	65	11	2.7	+	4.07	2.07E-04	6.06E-03
positive regulation of small GTPase mediated signal transduction (GO:0051057)	77	13	3.2	+	4.06	5.76E-05	2.10E-03
homotypic cell-cell adhesion (GO:0034109)	54	9	2.25	+	4.01	8.48E-04	1.97E-02
sprouting angiogenesis (GO:0002040)	60	10	2.5	+	4.01	4.44E-04	1.17E-02
gluconeogenesis (GO:0006094)	48	8	2	+	4.01	1.63E-03	3.31E-02
carbohydrate catabolic process (GO:0016052)	102	17	4.24	+	4.01	5.12E-06	2.66E-04
positive regulation of dendritic spine development (GO:0060999)	66	11	2.75	+	4.01	2.33E-04	6.75E-03
regulation of Rho protein signal transduction (GO:0035023)	85	14	3.54	+	3.96	3.85E-05	1.48E-03
mucopolysaccharide metabolic process (GO:1903510)	79	13	3.29	+	3.96	7.27E-05	2.56E-03
mammary gland epithelium development (GO:0061180)	67	11	2.79	+	3.95	2.61E-04	7.48E-03
neuroinflammatory response (GO:0150076)	55	9	2.29	+	3.93	9.52E-04	2.14E-02
glycolytic process (GO:0006096)	49	8	2.04	+	3.92	1.83E-03	3.63E-02
cellular response to decreased oxygen levels (GO:0036294)	98	16	4.08	+	3.92	1.21E-05	5.35E-04
hexose metabolic process (GO:0019318)	166	27	6.91	+	3.91	1.49E-08	1.54E-06
actomyosin structure organization (GO:0031032)	118	19	4.91	+	3.87	2.28E-06	1.35E-04
negative regulation of endothelial cell migration (GO:0010596)	56	9	2.33	+	3.86	1.07E-03	2.34E-02
cellular response to acetylcholine (GO:1905145)	50	8	2.08	+	3.85	2.05E-03	3.91E-02
regulation of protein localization to cell periphery (GO:1904375)	150	24	6.24	+	3.85	1.23E-07	1.01E-05
monosaccharide metabolic process (GO:0005996)	194	31	8.07	+	3.84	1.89E-09	2.37E-07
ceramide metabolic process (GO:0006672)	94	15	3.91	+	3.84	2.86E-05	1.12E-03
regulation of collagen metabolic process (GO:0010712)	63	10	2.62	+	3.82	6.24E-04	1.54E-02
regulation of epithelial to mesenchymal transition (GO:0010717)	101	16	4.2	+	3.81	1.69E-05	7.19E-04
cellular response to oxygen levels (GO:0071453)	114	18	4.74	+	3.8	5.34E-06	2.71E-04
tissue regeneration (GO:0042246)	57	9	2.37	+	3.8	1.19E-03	2.58E-02
regulation of cell shape (GO:0008360)	140	22	5.82	+	3.78	5.35E-07	3.81E-05
carbohydrate biosynthetic process (GO:0016051)	121	19	5.03	+	3.77	3.17E-06	1.78E-04
regulation of release of cytochrome c from mitochondria (GO:0090199)	51	8	2.12	+	3.77	2.29E-03	4.23E-02
regulation of purine nucleotide metabolic process (GO:1900542)	51	8	2.12	+	3.77	2.29E-03	4.22E-02
substrate adhesion-dependent cell spreading (GO:0034446)	51	8	2.12	+	3.77	2.29E-03	4.22E-02
response to unfolded protein (GO:0006986)	96	15	3.99	+	3.76	3.55E-05	1.38E-03
positive regulation of cell-substrate adhesion (GO:0010811)	141	22	5.87	+	3.75	5.96E-07	4.21E-05
modulation by symbiont of entry into host (GO:0052372)	58	9	2.41	+	3.73	1.33E-03	2.80E-02
regulation of cell-matrix adhesion (GO:0001952)	129	20	5.37	+	3.73	2.09E-06	1.26E-04
cellular response to acid chemical (GO:0071229)	84	13	3.49	+	3.72	1.26E-04	3.99E-03
regulation of nucleotide metabolic process (GO:0006140)	52	8	2.16	+	3.7	2.55E-03	4.59E-02
regulation of smooth muscle cell migration (GO:0014910)	104	16	4.33	+	3.7	2.33E-05	9.53E-04

Table S3. Enriched GO categories for genes within each cluster in Fig. 9A.

striated muscle cell development (GO:0055002)	65	10	2.7	+	3.7	7.75E-04	1.82E-02
glucose metabolic process (GO:0006006)	131	20	5.45	+	3.67	2.58E-06	1.50E-04
regulation of response to endoplasmic reticulum stress (GO:1905897)	79	12	3.29	+	3.65	2.65E-04	7.58E-03
regulation of sequestering of calcium ion (GO:0051282)	132	20	5.49	+	3.64	2.86E-06	1.63E-04
regulation of oligodendrocyte differentiation (GO:0048713)	53	8	2.2	+	3.63	2.83E-03	5.00E-02
regulation of phosphoprotein phosphatase activity (GO:0043666)	53	8	2.2	+	3.63	2.83E-03	5.00E-02
protein localization to vacuole (GO:0072665)	73	11	3.04	+	3.62	5.03E-04	1.29E-02
positive regulation of gliogenesis (GO:0014015)	87	13	3.62	+	3.59	1.73E-04	5.19E-03
regulation of blood coagulation (GO:0030193)	67	10	2.79	+	3.59	9.54E-04	2.14E-02
regulation of endothelial cell migration (GO:0010594)	175	26	7.28	+	3.57	1.37E-07	1.11E-05
regulation of cell-substrate adhesion (GO:0010810)	229	34	9.53	+	3.57	1.71E-09	2.16E-07
cellular response to starvation (GO:0009267)	176	26	7.32	+	3.55	1.52E-07	1.23E-05
viral life cycle (GO:0019058)	95	14	3.95	+	3.54	1.12E-04	3.62E-03
maintenance of protein location (GO:0045185)	102	15	4.24	+	3.54	6.60E-05	2.35E-03
regulation of blood vessel endothelial cell migration (GO:0043535)	96	14	3.99	+	3.51	1.24E-04	3.95E-03
regulation of muscle contraction (GO:0006937)	165	24	6.86	+	3.5	5.81E-07	4.12E-05
regulation of hemostasis (GO:1900046)	69	10	2.87	+	3.48	1.17E-03	2.53E-02
carbohydrate derivative catabolic process (GO:1901136)	159	23	6.61	+	3.48	1.08E-06	7.06E-05
positive regulation of protein localization to plasma membrane (GO:1903078)	63	9	2.62	+	3.43	2.22E-03	4.15E-02
osteoclast differentiation (GO:0030316)	63	9	2.62	+	3.43	2.22E-03	4.14E-02
bone mineralization (GO:0030282)	63	9	2.62	+	3.43	2.22E-03	4.14E-02
myofibril assembly (GO:0030239)	63	9	2.62	+	3.43	2.22E-03	4.13E-02
gland morphogenesis (GO:0022612)	126	18	5.24	+	3.43	1.81E-05	7.68E-04
negative regulation of wound healing (GO:0061045)	63	9	2.62	+	3.43	2.22E-03	4.13E-02
sphingolipid metabolic process (GO:0006665)	140	20	5.82	+	3.43	6.33E-06	3.16E-04
regulation of protein processing (GO:0070613)	70	10	2.91	+	3.43	1.29E-03	2.73E-02
positive regulation of Ras protein signal transduction (GO:0046579)	70	10	2.91	+	3.43	1.29E-03	2.72E-02
epithelial cell migration (GO:0010631)	113	16	4.7	+	3.4	5.73E-05	2.10E-03
pyruvate metabolic process (GO:0006090)	78	11	3.24	+	3.39	8.25E-04	1.92E-02
G1/S transition of mitotic cell cycle (GO:0000082)	78	11	3.24	+	3.39	8.25E-04	1.92E-02
glycosaminoglycan metabolic process (GO:0030203)	100	14	4.16	+	3.37	1.82E-04	5.44E-03
regulation of glial cell differentiation (GO:0045685)	93	13	3.87	+	3.36	3.10E-04	8.75E-03
glycolipid metabolic process (GO:0006664)	93	13	3.87	+	3.36	3.10E-04	8.73E-03
negative regulation of transforming growth factor beta receptor signaling pathway (GO:0030512)	93	13	3.87	+	3.36	3.10E-04	8.72E-03
calcium ion transmembrane transport (GO:0070588)	179	25	7.45	+	3.36	6.68E-07	4.58E-05
regulation of protein localization to membrane (GO:1905475)	208	29	8.65	+	3.35	9.03E-08	7.69E-06
response to starvation (GO:0042594)	201	28	8.36	+	3.35	1.52E-07	1.22E-05
actin filament organization (GO:0007015)	273	38	11.36	+	3.35	9.73E-10	1.29E-07
epithelium migration (GO:0090132)	115	16	4.78	+	3.34	6.92E-05	2.45E-03
regulation of action potential (GO:0098900)	72	10	2.99	+	3.34	1.56E-03	3.19E-02
regulation of coagulation (GO:0050818)	72	10	2.99	+	3.34	1.56E-03	3.19E-02
insulin receptor signaling pathway (GO:0008286)	72	10	2.99	+	3.34	1.56E-03	3.19E-02
negative regulation of extrinsic apoptotic signaling pathway (GO:2001237)	101	14	4.2	+	3.33	2.00E-04	5.89E-03
lung alveolus development (GO:0048286)	65	9	2.7	+	3.33	2.68E-03	4.81E-02
liposaccharide metabolic process (GO:1903509)	94	13	3.91	+	3.32	3.41E-04	9.41E-03
cell cycle G1/S phase transition (GO:0044843)	80	11	3.33	+	3.31	9.95E-04	2.21E-02
regulation of protein depolymerization (GO:1901879)	95	13	3.95	+	3.29	3.73E-04	1.01E-02
maintenance of location in cell (GO:0051651)	110	15	4.58	+	3.28	1.41E-04	4.39E-03
regulation of intracellular pH (GO:0051453)	81	11	3.37	+	3.26	1.09E-03	2.39E-02

Table S3. Enriched GO categories for genes within each cluster in Fig. 9A.

tissue migration (GO:0090130)	118	16	4.91	+	3.26	9.09E-05	3.06E-03
astrocyte differentiation (GO:0048708)	74	10	3.08	+	3.25	1.87E-03	3.66E-02
negative regulation of epithelial cell migration (GO:0010633)	74	10	3.08	+	3.25	1.87E-03	3.65E-02
positive regulation of endothelial cell migration (GO:0010595)	111	15	4.62	+	3.25	1.54E-04	4.71E-03
regulation of protein maturation (GO:1903317)	74	10	3.08	+	3.25	1.87E-03	3.65E-02
tissue remodeling (GO:0048771)	126	17	5.24	+	3.24	5.86E-05	2.13E-03
regulation of cellular pH (GO:0030641)	89	12	3.7	+	3.24	6.97E-04	1.67E-02
angiogenesis (GO:0001525)	342	46	14.23	+	3.23	4.69E-11	7.00E-09
regulation of release of sequestered calcium ion into cytosol (GO:0051279)	82	11	3.41	+	3.22	1.19E-03	2.58E-02
regulation of epithelial cell migration (GO:0010632)	247	33	10.27	+	3.21	2.98E-08	2.89E-06
regulation of cell morphogenesis (GO:0022604)	277	37	11.52	+	3.21	4.39E-09	5.10E-07
negative regulation of cysteine-type endopeptidase activity (GO:2000117)	90	12	3.74	+	3.21	7.62E-04	1.79E-02
positive regulation of osteoblast differentiation (GO:0045669)	75	10	3.12	+	3.21	2.05E-03	3.91E-02
negative regulation of calcium ion transport (GO:0051926)	75	10	3.12	+	3.21	2.05E-03	3.91E-02
regeneration (GO:0031099)	113	15	4.7	+	3.19	1.84E-04	5.48E-03
regulation of small GTPase mediated signal transduction (GO:0051056)	227	30	9.44	+	3.18	1.56E-07	1.24E-05
regulation of Notch signaling pathway (GO:0008593)	91	12	3.79	+	3.17	8.32E-04	1.93E-02
negative regulation of protein depolymerization (GO:1901880)	76	10	3.16	+	3.16	2.23E-03	4.16E-02
aminoglycan metabolic process (GO:0006022)	114	15	4.74	+	3.16	2.01E-04	5.91E-03
calcium-mediated signaling (GO:0019722)	152	20	6.32	+	3.16	1.87E-05	7.90E-04
extracellular matrix organization (GO:0030198)	275	36	11.44	+	3.15	1.14E-08	1.23E-06
response to acid chemical (GO:0001101)	107	14	4.45	+	3.15	3.41E-04	9.41E-03
positive regulation of insulin secretion (GO:0032024)	115	15	4.78	+	3.14	2.19E-04	6.39E-03
negative regulation of protein secretion (GO:0050709)	92	12	3.83	+	3.14	9.07E-04	2.07E-02
carbohydrate metabolic process (GO:0005975)	414	54	17.22	+	3.14	2.76E-12	5.04E-10
external encapsulating structure organization (GO:0045229)	277	36	11.52	+	3.12	1.35E-08	1.41E-06
extracellular structure organization (GO:0043062)	277	36	11.52	+	3.12	1.35E-08	1.40E-06
neural retina development (GO:0003407)	77	10	3.2	+	3.12	2.44E-03	4.42E-02
cell-matrix adhesion (GO:0007160)	131	17	5.45	+	3.12	9.04E-05	3.05E-03
cellular response to type II interferon (GO:0071346)	108	14	4.49	+	3.12	3.71E-04	1.01E-02
positive regulation of epithelial cell migration (GO:0010634)	162	21	6.74	+	3.12	1.43E-05	6.24E-04
connective tissue development (GO:0061448)	247	32	10.27	+	3.11	9.13E-08	7.74E-06
viral process (GO:0016032)	124	16	5.16	+	3.1	1.53E-04	4.68E-03
cell-substrate adhesion (GO:0031589)	186	24	7.74	+	3.1	3.86E-06	2.08E-04
sphingolipid biosynthetic process (GO:0030148)	93	12	3.87	+	3.1	9.87E-04	2.19E-02
cellular response to topologically incorrect protein (GO:0035967)	86	11	3.58	+	3.07	1.69E-03	3.39E-02
regulation of dendritic spine development (GO:0060998)	86	11	3.58	+	3.07	1.69E-03	3.39E-02
regulation of Ras protein signal transduction (GO:0046578)	196	25	8.15	+	3.07	2.94E-06	1.67E-04
regulation of sodium ion transport (GO:0002028)	102	13	4.24	+	3.06	6.86E-04	1.64E-02
regulation of muscle system process (GO:0090257)	259	33	10.77	+	3.06	8.21E-08	7.07E-06
calcium ion transport (GO:0006816)	244	31	10.15	+	3.05	2.13E-07	1.63E-05
maintenance of location (GO:0051235)	189	24	7.86	+	3.05	4.94E-06	2.57E-04
intracellular calcium ion homeostasis (GO:0006874)	221	28	9.19	+	3.05	8.58E-07	5.75E-05
regulation of tissue remodeling (GO:0034103)	87	11	3.62	+	3.04	1.83E-03	3.63E-02
ameboidal-type cell migration (GO:0001667)	222	28	9.23	+	3.03	9.31E-07	6.21E-05
response to topologically incorrect protein (GO:0035966)	119	15	4.95	+	3.03	3.05E-04	8.62E-03
regulation of G protein-coupled receptor signaling pathway (GO:0008277)	135	17	5.62	+	3.03	1.26E-04	3.99E-03
nephron development (GO:0072006)	137	17	5.7	+	2.98	1.48E-04	4.55E-03
positive regulation of protein localization to membrane (GO:1905477)	113	14	4.7	+	2.98	5.60E-04	1.40E-02

Table S3. Enriched GO categories for genes within each cluster in Fig. 9A.

cartilage development (GO:0051216)	186	23	7.74	+	2.97	1.14E-05	5.11E-04
proteoglycan metabolic process (GO:0006029)	89	11	3.7	+	2.97	2.15E-03	4.06E-02
actin filament-based movement (GO:0030048)	89	11	3.7	+	2.97	2.15E-03	4.06E-02
cellular response to extracellular stimulus (GO:0031668)	251	31	10.44	+	2.97	3.73E-07	2.72E-05
cellular response to nutrient levels (GO:0031669)	219	27	9.11	+	2.96	2.14E-06	1.27E-04
negative regulation of transmembrane receptor protein serine/threonine kinase signaling pathway (GO:0090101)	146	18	6.07	+	2.96	1.03E-04	3.36E-03
actin cytoskeleton organization (GO:0030036)	528	65	21.96	+	2.96	1.71E-13	4.32E-11
myelination (GO:0042552)	122	15	5.07	+	2.96	3.88E-04	1.05E-02
regulation of pH (GO:0006885)	106	13	4.41	+	2.95	9.48E-04	2.13E-02
blood vessel development (GO:0001568)	572	70	23.79	+	2.94	2.49E-14	7.97E-12
kidney epithelium development (GO:0072073)	139	17	5.78	+	2.94	1.73E-04	5.18E-03
memory (GO:0007613)	164	20	6.82	+	2.93	4.98E-05	1.86E-03
positive regulation of smooth muscle cell proliferation (GO:0048661)	115	14	4.78	+	2.93	6.55E-04	1.60E-02
biomineral tissue development (GO:0031214)	107	13	4.45	+	2.92	1.02E-03	2.27E-02
negative regulation of intrinsic apoptotic signaling pathway (GO:2001243)	99	12	4.12	+	2.91	1.61E-03	3.27E-02
cholesterol homeostasis (GO:0042632)	99	12	4.12	+	2.91	1.61E-03	3.27E-02
regulation of cell migration (GO:0030334)	1009	122	41.97	+	2.91	5.05E-24	1.32E-20
response to hypoxia (GO:0001666)	207	25	8.61	+	2.9	6.99E-06	3.42E-04
positive regulation of binding (GO:0051099)	199	24	8.28	+	2.9	1.08E-05	4.95E-04
negative regulation of angiogenesis (GO:0016525)	108	13	4.49	+	2.89	1.11E-03	2.41E-02
response to carbohydrate (GO:0009743)	133	16	5.53	+	2.89	3.14E-04	8.79E-03
myeloid leukocyte differentiation (GO:0002573)	158	19	6.57	+	2.89	9.01E-05	3.05E-03
blood vessel morphogenesis (GO:0048514)	458	55	19.05	+	2.89	3.12E-11	4.75E-09
sterol homeostasis (GO:0055092)	100	12	4.16	+	2.88	1.73E-03	3.47E-02
ensheathment of neurons (GO:0007272)	125	15	5.2	+	2.88	4.90E-04	1.26E-02
axon ensheathment (GO:0008366)	125	15	5.2	+	2.88	4.90E-04	1.26E-02
vasculature development (GO:0001944)	601	72	25	+	2.88	2.74E-14	8.60E-12
polyol metabolic process (GO:0019751)	92	11	3.83	+	2.87	2.72E-03	4.87E-02
cellular response to external stimulus (GO:0071496)	285	34	11.86	+	2.87	2.15E-07	1.64E-05
positive regulation of protein localization to nucleus (GO:1900182)	101	12	4.2	+	2.86	1.87E-03	3.65E-02
T cell proliferation (GO:0042098)	101	12	4.2	+	2.86	1.87E-03	3.64E-02
actin filament-based process (GO:0030029)	581	69	24.17	+	2.86	1.42E-13	3.64E-11
response to monosaccharide (GO:0034284)	118	14	4.91	+	2.85	8.24E-04	1.92E-02
negative regulation of blood vessel morphogenesis (GO:2000181)	110	13	4.58	+	2.84	1.29E-03	2.72E-02
protein localization to extracellular region (GO:0071692)	136	16	5.66	+	2.83	3.93E-04	1.06E-02
second-messenger-mediated signaling (GO:0019932)	221	26	9.19	+	2.83	7.07E-06	3.44E-04
epithelial cell proliferation (GO:0050673)	213	25	8.86	+	2.82	1.09E-05	4.97E-04
regulation of cell motility (GO:2000145)	1066	125	44.34	+	2.82	1.49E-23	3.34E-20
positive regulation of mitotic cell cycle (GO:0045931)	128	15	5.32	+	2.82	6.13E-04	1.52E-02
response to oxygen levels (GO:0070482)	239	28	9.94	+	2.82	3.42E-06	1.90E-04
negative regulation of protein localization (GO:1903828)	239	28	9.94	+	2.82	3.42E-06	1.89E-04
response to wounding (GO:0009611)	350	41	14.56	+	2.82	1.96E-08	1.97E-06
negative regulation of vasculature development (GO:1901343)	111	13	4.62	+	2.82	1.39E-03	2.90E-02
regulation of gliogenesis (GO:0014013)	137	16	5.7	+	2.81	4.23E-04	1.12E-02
nephron epithelium development (GO:0072009)	103	12	4.28	+	2.8	2.17E-03	4.09E-02
regulation of muscle adaptation (GO:0043502)	103	12	4.28	+	2.8	2.17E-03	4.08E-02
response to glucose (GO:0009749)	112	13	4.66	+	2.79	1.49E-03	3.08E-02
calcium ion homeostasis (GO:0055074)	250	29	10.4	+	2.79	2.74E-06	1.58E-04
hemostasis (GO:0007599)	121	14	5.03	+	2.78	1.03E-03	2.27E-02

Table S3. Enriched GO categories for genes within each cluster in Fig. 9A.

regulation of response to wounding (GO:1903034)	173	20	7.2	+	2.78	9.73E-05	3.24E-03
neuron apoptotic process (GO:0051402)	130	15	5.41	+	2.77	7.09E-04	1.69E-02
positive regulation of supramolecular fiber organization (GO:1902905)	182	21	7.57	+	2.77	6.75E-05	2.39E-03
response to decreased oxygen levels (GO:0036293)	217	25	9.03	+	2.77	1.46E-05	6.33E-04
receptor-mediated endocytosis (GO:0006898)	165	19	6.86	+	2.77	1.51E-04	4.63E-03
intracellular monoatomic cation homeostasis (GO:0030003)	436	50	18.14	+	2.76	1.04E-09	1.36E-07
regulation of locomotion (GO:0040012)	1109	127	46.13	+	2.75	4.16E-23	7.24E-20
positive regulation of cell migration (GO:0030335)	594	68	24.71	+	2.75	1.01E-12	2.05E-10
striated muscle cell differentiation (GO:0051146)	236	27	9.82	+	2.75	7.55E-06	3.63E-04
epithelial cell development (GO:0002064)	210	24	8.74	+	2.75	2.42E-05	9.80E-04
positive regulation of protein binding (GO:0032092)	105	12	4.37	+	2.75	2.51E-03	4.53E-02
response to endoplasmic reticulum stress (GO:0034976)	228	26	9.48	+	2.74	1.17E-05	5.18E-04
regulation of heart contraction (GO:0008016)	193	22	8.03	+	2.74	5.39E-05	1.97E-03
intracellular monoatomic ion homeostasis (GO:0006873)	448	51	18.64	+	2.74	8.92E-10	1.19E-07
neuron death (GO:0070997)	132	15	5.49	+	2.73	8.18E-04	1.91E-02
regulation of actin filament-based process (GO:0032970)	406	46	16.89	+	2.72	6.88E-09	7.75E-07
negative regulation of cellular response to growth factor stimulus (GO:0090288)	106	12	4.41	+	2.72	2.70E-03	4.83E-02
response to hexose (GO:0009746)	115	13	4.78	+	2.72	1.85E-03	3.64E-02
positive regulation of locomotion (GO:0040017)	637	72	26.5	+	2.72	5.45E-13	1.19E-10
supramolecular fiber organization (GO:0097435)	576	65	23.96	+	2.71	8.86E-12	1.46E-09
regulation of protein-containing complex disassembly (GO:0043244)	133	15	5.53	+	2.71	8.77E-04	2.02E-02
positive regulation of cell motility (GO:2000147)	621	70	25.83	+	2.71	1.27E-12	2.52E-10
mitotic cell cycle phase transition (GO:0044772)	151	17	6.28	+	2.71	4.18E-04	1.11E-02
wound healing (GO:0042060)	258	29	10.73	+	2.7	4.80E-06	2.52E-04
Ras protein signal transduction (GO:0007265)	178	20	7.4	+	2.7	1.38E-04	4.32E-03
cell recognition (GO:0008037)	143	16	5.95	+	2.69	6.48E-04	1.59E-02
gliogenesis (GO:0042063)	295	33	12.27	+	2.69	1.20E-06	7.65E-05
small GTPase mediated signal transduction (GO:0007264)	242	27	10.07	+	2.68	1.14E-05	5.10E-04
intracellular chemical homeostasis (GO:0055082)	566	63	23.54	+	2.68	2.61E-11	4.00E-09
negative regulation of apoptotic signaling pathway (GO:2001234)	243	27	10.11	+	2.67	1.22E-05	5.38E-04
regulation of actin filament organization (GO:0110053)	279	31	11.61	+	2.67	2.84E-06	1.62E-04
regulation of protein localization to nucleus (GO:1900180)	162	18	6.74	+	2.67	3.31E-04	9.25E-03
positive regulation of apoptotic process (GO:0043065)	632	70	26.29	+	2.66	2.12E-12	4.06E-10
mammary gland development (GO:0030879)	154	17	6.41	+	2.65	5.13E-04	1.31E-02
regulation of angiogenesis (GO:0045765)	299	33	12.44	+	2.65	1.56E-06	9.70E-05
cell junction assembly (GO:0034329)	281	31	11.69	+	2.65	3.25E-06	1.82E-04
regulation of smooth muscle cell proliferation (GO:0048660)	191	21	7.94	+	2.64	1.25E-04	3.98E-03
vascular process in circulatory system (GO:0003018)	246	27	10.23	+	2.64	1.49E-05	6.46E-04
regulation of transforming growth factor beta receptor signaling pathway (GO:0017015)	137	15	5.7	+	2.63	1.15E-03	2.51E-02
protein secretion (GO:0009306)	128	14	5.32	+	2.63	1.67E-03	3.37E-02
muscle cell development (GO:0055001)	183	20	7.61	+	2.63	1.94E-04	5.74E-03
synaptic vesicle cycle (GO:0099504)	156	17	6.49	+	2.62	5.86E-04	1.46E-02
muscle cell differentiation (GO:0042692)	312	34	12.98	+	2.62	2.09E-06	1.26E-04
regulation of vasculature development (GO:1901342)	303	33	12.6	+	2.62	3.06E-06	1.73E-04
regulation of fat cell differentiation (GO:0045598)	147	16	6.11	+	2.62	8.49E-04	1.97E-02
membrane lipid metabolic process (GO:0006643)	184	20	7.65	+	2.61	2.07E-04	6.07E-03
establishment of protein localization to extracellular region (GO:0035592)	129	14	5.37	+	2.61	1.79E-03	3.57E-02
regulation of wound healing (GO:0061041)	129	14	5.37	+	2.61	1.79E-03	3.57E-02
positive regulation of programmed cell death (GO:0043068)	656	71	27.29	+	2.6	3.57E-12	6.29E-10

Table S3. Enriched GO categories for genes within each cluster in Fig. 9A.

ossification (GO:0001503)	259	28	10.77	+	2.6	2.12E-05	8.83E-04
plasma membrane organization (GO:0007009)	149	16	6.2	+	2.58	9.68E-04	2.15E-02
positive regulation of angiogenesis (GO:0045766)	177	19	7.36	+	2.58	3.40E-04	9.46E-03
vacuole organization (GO:0007033)	177	19	7.36	+	2.58	3.40E-04	9.44E-03
positive regulation of vasculature development (GO:1904018)	177	19	7.36	+	2.58	3.40E-04	9.43E-03
tube morphogenesis (GO:0035239)	756	81	31.45	+	2.58	1.85E-13	4.53E-11
regulation of cellular response to transforming growth factor beta stimulus (GO:1903844)	140	15	5.82	+	2.58	1.40E-03	2.93E-02
cellular homeostasis (GO:0019725)	674	72	28.04	+	2.57	5.51E-12	9.38E-10
positive regulation of cytoskeleton organization (GO:0051495)	197	21	8.19	+	2.56	1.85E-04	5.51E-03
positive regulation of endopeptidase activity (GO:0010950)	169	18	7.03	+	2.56	5.25E-04	1.33E-02
negative regulation of cell migration (GO:0030336)	311	33	12.94	+	2.55	4.13E-06	2.22E-04
negative regulation of protein transport (GO:0051224)	151	16	6.28	+	2.55	1.10E-03	2.41E-02
negative regulation of transmembrane transport (GO:0034763)	151	16	6.28	+	2.55	1.10E-03	2.40E-02
axon guidance (GO:0007411)	236	25	9.82	+	2.55	7.44E-05	2.62E-03
neuron projection guidance (GO:0097485)	236	25	9.82	+	2.55	7.44E-05	2.61E-03
regulation of extrinsic apoptotic signaling pathway (GO:2001236)	170	18	7.07	+	2.55	5.59E-04	1.41E-02
sensory perception of sound (GO:0007605)	170	18	7.07	+	2.55	5.59E-04	1.40E-02
regulation of calcium ion transport (GO:0051924)	293	31	12.19	+	2.54	8.70E-06	4.13E-04
regulation of insulin secretion (GO:0050796)	218	23	9.07	+	2.54	1.59E-04	4.83E-03
protein localization to plasma membrane (GO:0072659)	218	23	9.07	+	2.54	1.59E-04	4.83E-03
regulation of neuron apoptotic process (GO:0043523)	294	31	12.23	+	2.53	9.09E-06	4.29E-04
positive regulation of intracellular transport (GO:0032388)	190	20	7.9	+	2.53	4.88E-04	1.26E-02
endocrine system development (GO:0035270)	133	14	5.53	+	2.53	2.32E-03	4.27E-02
muscle structure development (GO:0061061)	532	56	22.13	+	2.53	2.56E-09	3.14E-07
transmembrane receptor protein tyrosine kinase signaling pathway (GO:0007169)	400	42	16.64	+	2.52	2.23E-07	1.69E-05
negative regulation of cell motility (GO:2000146)	324	34	13.48	+	2.52	3.44E-06	1.90E-04
regulation of muscle cell differentiation (GO:0051147)	162	17	6.74	+	2.52	8.62E-04	1.99E-02
monoatomic cation homeostasis (GO:0055080)	515	54	21.42	+	2.52	5.63E-09	6.49E-07
negative regulation of supramolecular fiber organization (GO:1902904)	172	18	7.15	+	2.52	6.34E-04	1.56E-02
skeletal system development (GO:0001501)	507	53	21.09	+	2.51	8.48E-09	9.29E-07
cell migration (GO:0016477)	938	98	39.02	+	2.51	1.68E-15	5.86E-13
regulation of synaptic plasticity (GO:0048167)	249	26	10.36	+	2.51	5.88E-05	2.12E-03
negative regulation of endopeptidase activity (GO:0010951)	182	19	7.57	+	2.51	7.34E-04	1.74E-02
monoatomic ion homeostasis (GO:0050801)	527	55	21.92	+	2.51	4.31E-09	5.04E-07
cell cycle phase transition (GO:0044770)	163	17	6.78	+	2.51	9.17E-04	2.08E-02
regulation of cysteine-type endopeptidase activity (GO:2000116)	240	25	9.98	+	2.5	8.56E-05	2.92E-03
enzyme-linked receptor protein signaling pathway (GO:0007167)	615	64	25.58	+	2.5	2.06E-10	2.86E-08
vacuolar transport (GO:0007034)	164	17	6.82	+	2.49	1.58E-03	3.23E-02
learning or memory (GO:0007611)	328	34	13.64	+	2.49	4.21E-06	2.25E-04
positive regulation of peptidase activity (GO:0010952)	193	20	8.03	+	2.49	5.28E-04	1.34E-02
regulation of osteoblast differentiation (GO:0045667)	145	15	6.03	+	2.49	1.92E-03	3.73E-02
positive regulation of neurogenesis (GO:0050769)	319	33	13.27	+	2.49	6.08E-06	3.05E-04
positive regulation of protein localization (GO:1903829)	523	54	21.76	+	2.48	7.72E-09	8.52E-07
response to ketone (GO:1901654)	155	16	6.45	+	2.48	1.41E-03	2.94E-02
negative regulation of establishment of protein localization (GO:1904950)	155	16	6.45	+	2.48	1.41E-03	2.94E-02
regulation of blood circulation (GO:1903522)	263	27	10.94	+	2.47	5.03E-05	1.87E-03
sensory perception of mechanical stimulus (GO:0050954)	205	21	8.53	+	2.46	3.99E-04	1.07E-02
cellular response to peptide hormone stimulus (GO:0071375)	215	22	8.94	+	2.46	2.85E-04	8.10E-03
regulation of supramolecular fiber organization (GO:1902903)	391	40	16.26	+	2.46	1.13E-06	7.37E-05

Table S3. Enriched GO categories for genes within each cluster in Fig. 9A.

mesenchymal cell differentiation (GO:0048762)	176	18	7.32	+	2.46	1.17E-03	2.53E-02
regulation of body fluid levels (GO:0050878)	323	33	13.44	+	2.46	7.49E-06	3.61E-04
positive regulation of epithelial cell proliferation (GO:0050679)	235	24	9.78	+	2.46	1.46E-04	4.52E-03
positive regulation of cell growth (GO:0030307)	196	20	8.15	+	2.45	5.82E-04	1.45E-02
cell junction organization (GO:0034330)	559	57	23.25	+	2.45	3.98E-09	4.73E-07
branching morphogenesis of an epithelial tube (GO:0048754)	167	17	6.95	+	2.45	1.71E-03	3.43E-02
intrinsic apoptotic signaling pathway (GO:0097193)	177	18	7.36	+	2.44	1.20E-03	2.59E-02
positive regulation of protein transport (GO:0051222)	345	35	14.35	+	2.44	7.14E-06	3.46E-04
cellular response to organonitrogen compound (GO:0071417)	533	54	22.17	+	2.44	1.26E-08	1.34E-06
regulation of actin cytoskeleton organization (GO:0032956)	356	36	14.81	+	2.43	5.15E-06	2.66E-04
regulation of peptide transport (GO:0090087)	267	27	11.11	+	2.43	6.08E-05	2.19E-03
protein localization to cell periphery (GO:1990778)	277	28	11.52	+	2.43	4.43E-05	1.67E-03
negative regulation of secretion by cell (GO:1903531)	188	19	7.82	+	2.43	8.79E-04	2.02E-02
negative regulation of neuron apoptotic process (GO:0043524)	198	20	8.24	+	2.43	6.26E-04	1.54E-02
regulation of protein kinase B signaling (GO:0051896)	179	18	7.45	+	2.42	1.28E-03	2.72E-02
positive regulation of protein secretion (GO:0050714)	189	19	7.86	+	2.42	9.11E-04	2.08E-02
endocytosis (GO:0006897)	488	49	20.3	+	2.41	7.87E-08	6.82E-06
regulation of protein secretion (GO:0050708)	329	33	13.69	+	2.41	1.57E-05	6.71E-04
inorganic cation transmembrane transport (GO:0098662)	509	51	21.17	+	2.41	4.42E-08	4.13E-06
cellular response to organic cyclic compound (GO:0071407)	420	42	17.47	+	2.4	8.02E-07	5.40E-05
negative regulation of cytoskeleton organization (GO:0051494)	170	17	7.07	+	2.4	1.87E-03	3.66E-02
negative regulation of secretion (GO:0051048)	210	21	8.74	+	2.4	4.85E-04	1.26E-02
regulation of cysteine-type endopeptidase activity involved in apoptotic process (GO:0043281)	210	21	8.74	+	2.4	4.85E-04	1.26E-02
heart morphogenesis (GO:0003007)	280	28	11.65	+	2.4	5.15E-05	1.90E-03
regulation of cellular response to growth factor stimulus (GO:0090287)	320	32	13.31	+	2.4	2.27E-05	9.31E-04
regulation of peptide hormone secretion (GO:0090276)	260	26	10.82	+	2.4	9.67E-05	3.23E-03
inorganic ion homeostasis (GO:0098771)	450	45	18.72	+	2.4	3.07E-07	2.27E-05
morphogenesis of a branching epithelium (GO:0061138)	200	20	8.32	+	2.4	6.76E-04	1.63E-02
positive regulation of establishment of protein localization (GO:1904951)	361	36	15.02	+	2.4	5.96E-06	3.01E-04
myeloid cell differentiation (GO:0030099)	311	31	12.94	+	2.4	3.28E-05	1.28E-03
lung development (GO:0030324)	221	22	9.19	+	2.39	3.65E-04	9.98E-03
regulation of calcium ion transmembrane transport (GO:1903169)	201	20	8.36	+	2.39	7.04E-04	1.68E-02
regulation of intrinsic apoptotic signaling pathway (GO:2001242)	181	18	7.53	+	2.39	1.38E-03	2.88E-02
negative regulation of programmed cell death (GO:0043069)	997	99	41.47	+	2.39	2.32E-14	7.57E-12
protein processing (GO:0016485)	212	21	8.82	+	2.38	5.29E-04	1.34E-02
tube development (GO:0035295)	991	98	41.22	+	2.38	3.88E-14	1.15E-11
circulatory system development (GO:0072359)	974	96	40.52	+	2.37	8.67E-14	2.30E-11
negative regulation of locomotion (GO:0040013)	356	35	14.81	+	2.36	9.93E-06	4.60E-04
regulation of anatomical structure morphogenesis (GO:0022603)	946	93	39.35	+	2.36	2.56E-13	5.91E-11
negative regulation of DNA-binding transcription factor activity (GO:0043433)	173	17	7.2	+	2.36	2.08E-03	3.94E-02
cell-cell adhesion (GO:0098609)	458	45	19.05	+	2.36	6.83E-07	4.65E-05
negative regulation of apoptotic process (GO:0043066)	967	95	40.22	+	2.36	1.39E-13	3.63E-11
respiratory tube development (GO:0030323)	224	22	9.32	+	2.36	4.19E-04	1.11E-02
regulation of peptide secretion (GO:0002791)	265	26	11.02	+	2.36	1.24E-04	3.95E-03
regulation of endopeptidase activity (GO:0052548)	358	35	14.89	+	2.35	1.07E-05	4.92E-04
cellular response to peptide (GO:1901653)	266	26	11.06	+	2.35	1.31E-04	4.11E-03
positive regulation of cell adhesion (GO:0045785)	543	53	22.59	+	2.35	5.70E-08	5.14E-06
cellular response to nitrogen compound (GO:1901699)	575	56	23.92	+	2.34	2.41E-08	2.36E-06
monoatomic cation transmembrane transport (GO:0098655)	514	50	21.38	+	2.34	1.56E-07	1.24E-05

Table S3. Enriched GO categories for genes within each cluster in Fig. 9A.

eye morphogenesis (GO:0048592)	175	17	7.28	+	2.34	2.24E-03	4.16E-02
vesicle-mediated transport in synapse (GO:0099003)	175	17	7.28	+	2.34	2.24E-03	4.16E-02
regulation of protein localization (GO:0032880)	980	95	40.76	+	2.33	3.29E-13	7.47E-11
regulation of binding (GO:0051098)	403	39	16.76	+	2.33	3.78E-06	2.05E-04
regulation of apoptotic signaling pathway (GO:2001233)	424	41	17.64	+	2.32	3.07E-06	1.73E-04
cellular response to salt (GO:1902075)	207	20	8.61	+	2.32	9.13E-04	2.08E-02
positive regulation of endocytosis (GO:0045807)	207	20	8.61	+	2.32	9.13E-04	2.07E-02
transmembrane receptor protein serine/threonine kinase signaling pathway (GO:0007178)	218	21	9.07	+	2.32	6.94E-04	1.66E-02
positive regulation of protein catabolic process (GO:0045732)	208	20	8.65	+	2.31	9.56E-04	2.14E-02
regulation of metal ion transport (GO:0010959)	458	44	19.05	+	2.31	1.27E-06	8.11E-05
axonogenesis (GO:0007409)	386	37	16.06	+	2.3	1.26E-05	5.52E-04
regulation of programmed cell death (GO:0043067)	1639	157	68.18	+	2.3	3.11E-21	2.03E-18
cell population proliferation (GO:0008283)	867	83	36.06	+	2.3	2.44E-11	3.78E-09
regulation of apoptotic process (GO:0042981)	1588	152	66.06	+	2.3	1.75E-20	9.80E-18
small molecule biosynthetic process (GO:0044283)	439	42	18.26	+	2.3	2.53E-06	1.48E-04
regulation of canonical Wnt signaling pathway (GO:0060828)	251	24	10.44	+	2.3	4.60E-04	1.20E-02
macroautophagy (GO:0016236)	178	17	7.4	+	2.3	2.53E-03	4.56E-02
regulation of synapse organization (GO:0050807)	304	29	12.65	+	2.29	9.45E-05	3.17E-03
cognition (GO:0050890)	367	35	15.27	+	2.29	1.59E-05	6.79E-04
regulation of myeloid cell differentiation (GO:0045637)	231	22	9.61	+	2.29	5.84E-04	1.45E-02
regulation of cell junction assembly (GO:1901888)	242	23	10.07	+	2.28	6.62E-04	1.61E-02
response to steroid hormone (GO:0048545)	179	17	7.45	+	2.28	2.64E-03	4.73E-02
lipid homeostasis (GO:0055088)	179	17	7.45	+	2.28	2.64E-03	4.73E-02
gland development (GO:0048732)	453	43	18.84	+	2.28	2.11E-06	1.26E-04
morphogenesis of a branching structure (GO:0001763)	211	20	8.78	+	2.28	1.10E-03	2.40E-02
regulation of cell adhesion (GO:0030155)	844	80	35.11	+	2.28	8.23E-11	1.18E-08
positive regulation of nervous system development (GO:0051962)	380	36	15.81	+	2.28	1.91E-05	8.02E-04
chemical homeostasis (GO:0048878)	921	87	38.31	+	2.27	1.64E-11	2.60E-09
cell morphogenesis involved in differentiation (GO:0000904)	572	54	23.79	+	2.27	1.22E-07	1.01E-05
regulation of system process (GO:0044057)	636	60	26.46	+	2.27	3.15E-08	3.03E-06
morphogenesis of an epithelium (GO:0002009)	530	50	22.05	+	2.27	4.22E-07	3.05E-05
axon development (GO:0061564)	435	41	18.09	+	2.27	4.33E-06	2.30E-04
cell-cell adhesion via plasma-membrane adhesion molecules (GO:0098742)	191	18	7.94	+	2.27	2.08E-03	3.94E-02
regulation of cellular localization (GO:0060341)	1085	102	45.13	+	2.26	2.43E-13	5.70E-11
renal system development (GO:0072001)	330	31	13.73	+	2.26	6.36E-05	2.27E-03
positive regulation of growth (GO:0045927)	309	29	12.85	+	2.26	1.15E-04	3.68E-03
cell part morphogenesis (GO:0032990)	565	53	23.5	+	2.26	1.88E-07	1.47E-05
skeletal system morphogenesis (GO:0048705)	256	24	10.65	+	2.25	5.23E-04	1.33E-02
cell adhesion (GO:0007155)	898	84	37.35	+	2.25	5.36E-11	7.85E-09
regulation of protein transport (GO:0051223)	580	54	24.13	+	2.24	1.68E-07	1.33E-05
anatomical structure formation involved in morphogenesis (GO:0048646)	999	93	41.55	+	2.24	5.86E-12	9.88E-10
cellular response to hormone stimulus (GO:0032870)	419	39	17.43	+	2.24	9.53E-06	4.44E-04
cell morphogenesis (GO:0000902)	753	70	31.32	+	2.23	3.00E-09	3.62E-07
regulation of synapse structure or activity (GO:0050803)	312	29	12.98	+	2.23	1.30E-04	4.08E-03
regulation of protein binding (GO:0043393)	226	21	9.4	+	2.23	1.43E-03	2.97E-02
epithelial tube morphogenesis (GO:0060562)	366	34	15.22	+	2.23	4.33E-05	1.64E-03
cell-cell junction organization (GO:0045216)	194	18	8.07	+	2.23	2.37E-03	4.34E-02
cell morphogenesis involved in neuron differentiation (GO:0048667)	485	45	20.17	+	2.23	2.51E-06	1.47E-04
response to oxidative stress (GO:0006979)	345	32	14.35	+	2.23	8.19E-05	2.82E-03

Table S3. Enriched GO categories for genes within each cluster in Fig. 9A.

synapse organization (GO:0050808)	345	32	14.35	+	2.23	8.19E-05	2.82E-03
regulation of endocytosis (GO:0030100)	345	32	14.35	+	2.23	8.19E-05	2.81E-03
negative regulation of transport (GO:0051051)	518	48	21.55	+	2.23	1.04E-06	6.86E-05
regulation of neuron death (GO:1901214)	367	34	15.27	+	2.23	4.45E-05	1.67E-03
import into cell (GO:0098657)	659	61	27.41	+	2.23	3.35E-08	3.18E-06
cell motility (GO:0048870)	1114	103	46.34	+	2.22	4.89E-13	1.08E-10
kidney development (GO:0001822)	314	29	13.06	+	2.22	1.41E-04	4.39E-03
apoptotic process (GO:0006915)	1000	92	41.6	+	2.21	1.13E-11	1.85E-09
regulation of establishment of protein localization (GO:0070201)	609	56	25.33	+	2.21	1.66E-07	1.32E-05
striated muscle tissue development (GO:0014706)	218	20	9.07	+	2.21	2.11E-03	3.99E-02
cellular component morphogenesis (GO:0032989)	665	61	27.66	+	2.21	6.35E-08	5.65E-06
positive regulation of proteolysis (GO:0045862)	371	34	15.43	+	2.2	5.04E-05	1.87E-03
programmed cell death (GO:0012501)	1059	97	44.05	+	2.2	4.26E-12	7.43E-10
animal organ morphogenesis (GO:0009887)	1070	98	44.51	+	2.2	3.13E-12	5.64E-10
cell death (GO:0008219)	1062	97	44.18	+	2.2	4.63E-12	7.98E-10
respiratory system development (GO:0060541)	252	23	10.48	+	2.19	8.78E-04	2.02E-02
cell projection morphogenesis (GO:0048858)	537	49	22.34	+	2.19	1.57E-06	9.72E-05
inorganic ion transmembrane transport (GO:0098660)	571	52	23.75	+	2.19	6.44E-07	4.43E-05
regulation of blood pressure (GO:0008217)	220	20	9.15	+	2.19	2.21E-03	4.15E-02
positive regulation of neuron projection development (GO:0010976)	231	21	9.61	+	2.19	1.63E-03	3.31E-02
endomembrane system organization (GO:0010256)	507	46	21.09	+	2.18	2.73E-06	1.58E-04
blood circulation (GO:0008015)	441	40	18.34	+	2.18	1.51E-05	6.49E-04
apoptotic signaling pathway (GO:0097190)	331	30	13.77	+	2.18	1.83E-04	5.47E-03
glycoprotein metabolic process (GO:0009100)	320	29	13.31	+	2.18	2.48E-04	7.14E-03
protein complex oligomerization (GO:0051259)	254	23	10.57	+	2.18	9.41E-04	2.13E-02
negative regulation of neuron death (GO:1901215)	243	22	10.11	+	2.18	1.25E-03	2.68E-02
circulatory system process (GO:0003013)	475	43	19.76	+	2.18	6.51E-06	3.22E-04
tissue morphogenesis (GO:0048729)	642	58	26.71	+	2.17	1.97E-07	1.52E-05
regulation of hormone secretion (GO:0046883)	333	30	13.85	+	2.17	1.94E-04	5.74E-03
response to abiotic stimulus (GO:0009628)	989	89	41.14	+	2.16	8.03E-11	1.17E-08
regulation of monoatomic ion transmembrane transporter activity (GO:0032412)	289	26	12.02	+	2.16	6.58E-04	1.61E-02
regulation of monoatomic ion transport (GO:0043269)	634	57	26.37	+	2.16	2.86E-07	2.13E-05
regulation of epithelial cell proliferation (GO:0050678)	423	38	17.6	+	2.16	3.03E-05	1.18E-03
glial cell differentiation (GO:0010001)	234	21	9.73	+	2.16	1.79E-03	3.57E-02
positive regulation of ERK1 and ERK2 cascade (GO:0070374)	234	21	9.73	+	2.16	1.79E-03	3.57E-02
negative regulation of cell activation (GO:0050866)	223	20	9.28	+	2.16	2.41E-03	4.39E-02
response to growth factor (GO:0070848)	459	41	19.09	+	2.15	1.40E-05	6.14E-04
negative regulation of intracellular signal transduction (GO:1902532)	560	50	23.29	+	2.15	1.62E-06	9.90E-05
developmental maturation (GO:0021700)	325	29	13.52	+	2.15	2.83E-04	8.08E-03
monoatomic ion transmembrane transport (GO:0034220)	594	53	24.71	+	2.15	1.06E-06	6.93E-05
cellular response to endogenous stimulus (GO:0071495)	1088	97	45.26	+	2.14	1.51E-11	2.41E-09
membrane organization (GO:0061024)	685	61	28.49	+	2.14	1.69E-07	1.32E-05
metal ion transport (GO:0030001)	618	55	25.71	+	2.14	6.02E-07	4.21E-05
plasma membrane bounded cell projection morphogenesis (GO:0120039)	529	47	22	+	2.14	4.03E-06	2.17E-04
muscle organ development (GO:0007517)	305	27	12.69	+	2.13	5.39E-04	1.36E-02
regulation of intracellular transport (GO:0032386)	339	30	14.1	+	2.13	2.35E-04	6.78E-03
regulation of transmembrane receptor protein serine/threonine kinase signaling pathway (GO:0090092)	272	24	11.31	+	2.12	1.35E-03	2.84E-02
positive regulation of cell projection organization (GO:0031346)	454	40	18.88	+	2.12	2.29E-05	9.38E-04
neuron projection morphogenesis (GO:0048812)	523	46	21.76	+	2.11	6.25E-06	3.13E-04

Table S3. Enriched GO categories for genes within each cluster in Fig. 9A.

rhythmic process (GO:0048511)	273	24	11.36	+	2.11	1.37E-03	2.87E-02
positive regulation of monoatomic ion transport (GO:0043270)	273	24	11.36	+	2.11	1.37E-03	2.87E-02
regulation of intracellular protein transport (GO:0033157)	228	20	9.48	+	2.11	2.82E-03	4.99E-02
positive regulation of transport (GO:0051050)	1095	96	45.55	+	2.11	4.88E-11	7.22E-09
negative regulation of signaling (GO:0023057)	1439	126	59.86	+	2.11	3.97E-14	1.15E-11
sensory organ morphogenesis (GO:0090596)	309	27	12.85	+	2.1	6.04E-04	1.50E-02
negative regulation of cell communication (GO:0010648)	1442	126	59.98	+	2.1	4.17E-14	1.19E-11
response to extracellular stimulus (GO:0009991)	435	38	18.09	+	2.1	4.40E-05	1.66E-03
regulation of GTPase activity (GO:0043087)	344	30	14.31	+	2.1	4.18E-04	1.11E-02
regulation of small molecule metabolic process (GO:0062012)	379	33	15.77	+	2.09	1.75E-04	5.23E-03
response to nutrient levels (GO:0031667)	402	35	16.72	+	2.09	1.01E-04	3.33E-03
regulation of neurogenesis (GO:0050767)	483	42	20.09	+	2.09	2.19E-05	9.09E-04
monoatomic cation transport (GO:0006812)	714	62	29.7	+	2.09	2.98E-07	2.21E-05
regulation of cytoskeleton organization (GO:0051493)	530	46	22.05	+	2.09	1.14E-05	5.12E-04
regulation of leukocyte migration (GO:0002685)	242	21	10.07	+	2.09	2.37E-03	4.34E-02
response to endogenous stimulus (GO:0009719)	1317	114	54.78	+	2.08	1.29E-12	2.53E-10
positive regulation of cell differentiation (GO:0045597)	982	85	40.85	+	2.08	1.12E-09	1.46E-07
regulation of monoatomic cation transmembrane transport (GO:1904062)	371	32	15.43	+	2.07	2.52E-04	7.25E-03
positive regulation of hydrolase activity (GO:0051345)	545	47	22.67	+	2.07	9.13E-06	4.30E-04
negative regulation of developmental process (GO:0051093)	1009	87	41.97	+	2.07	9.93E-10	1.31E-07
skin development (GO:0043588)	302	26	12.56	+	2.07	9.07E-04	2.07E-02
regulation of transmembrane transporter activity (GO:0022898)	302	26	12.56	+	2.07	9.07E-04	2.07E-02
neuron projection development (GO:0031175)	767	66	31.9	+	2.07	1.15E-07	9.66E-06
negative regulation of signal transduction (GO:0009968)	1314	113	54.66	+	2.07	2.19E-12	4.08E-10
regulation of protein serine/threonine kinase activity (GO:0071900)	349	30	14.52	+	2.07	4.55E-04	1.19E-02
positive regulation of cell development (GO:0010720)	547	47	22.75	+	2.07	9.58E-06	4.46E-04
response to radiation (GO:0009314)	431	37	17.93	+	2.06	1.02E-04	3.34E-03
response to organic cyclic compound (GO:0014070)	699	60	29.08	+	2.06	6.15E-07	4.27E-05
alcohol metabolic process (GO:0006066)	303	26	12.6	+	2.06	9.35E-04	2.12E-02
cellular response to growth factor stimulus (GO:0071363)	443	38	18.43	+	2.06	7.71E-05	2.68E-03
regulation of DNA-binding transcription factor activity (GO:0051090)	443	38	18.43	+	2.06	7.71E-05	2.68E-03
regulation of nervous system development (GO:0051960)	583	50	24.25	+	2.06	4.52E-06	2.39E-04
cellular response to cytokine stimulus (GO:0071345)	724	62	30.12	+	2.06	3.66E-07	2.68E-05
modulation of chemical synaptic transmission (GO:0050804)	596	51	24.79	+	2.06	5.15E-06	2.66E-04
regulation of monoatomic ion transmembrane transport (GO:0034765)	491	42	20.42	+	2.06	2.80E-05	1.11E-03
response to hormone (GO:0009725)	643	55	26.75	+	2.06	1.72E-06	1.05E-04
positive regulation of cellular component biogenesis (GO:0044089)	550	47	22.88	+	2.05	1.04E-05	4.77E-04
regulation of transporter activity (GO:0032409)	316	27	13.14	+	2.05	7.58E-04	1.79E-02
developmental growth (GO:0048589)	515	44	21.42	+	2.05	2.35E-05	9.59E-04
regulation of trans-synaptic signaling (GO:0099177)	597	51	24.83	+	2.05	5.24E-06	2.67E-04
tissue development (GO:0009888)	1828	156	76.04	+	2.05	1.05E-16	4.86E-14
positive regulation of secretion (GO:0051047)	434	37	18.05	+	2.05	1.08E-04	3.50E-03
protein maturation (GO:0051604)	305	26	12.69	+	2.05	9.98E-04	2.21E-02
anatomical structure morphogenesis (GO:0009653)	2338	198	97.25	+	2.04	4.69E-21	2.94E-18
positive regulation of cell communication (GO:0010647)	1855	157	77.16	+	2.03	1.67E-16	7.09E-14
growth (GO:0040007)	520	44	21.63	+	2.03	2.63E-05	1.05E-03
cell-cell signaling by wnt (GO:0198738)	272	23	11.31	+	2.03	2.33E-03	4.27E-02
Wnt signaling pathway (GO:0016055)	272	23	11.31	+	2.03	2.33E-03	4.27E-02
negative regulation of hydrolase activity (GO:0051346)	355	30	14.77	+	2.03	5.24E-04	1.33E-02

Table S3. Enriched GO categories for genes within each cluster in Fig. 9A.

response to organonitrogen compound (GO:0010243)	876	74	36.44	+	2.03	3.95E-08	3.70E-06
regulation of vesicle-mediated transport (GO:0060627)	616	52	25.62	+	2.03	4.73E-06	2.49E-04
cytoskeleton organization (GO:0007010)	1198	101	49.83	+	2.03	1.15E-10	1.63E-08
regulation of cell differentiation (GO:0045595)	1685	142	70.09	+	2.03	1.11E-14	3.79E-12
regulation of Wnt signaling pathway (GO:0030111)	321	27	13.35	+	2.02	1.23E-03	2.64E-02
monoatomic ion transport (GO:0006811)	892	75	37.1	+	2.02	4.89E-08	4.53E-06
positive regulation of cell population proliferation (GO:0008284)	1062	89	44.18	+	2.01	2.49E-09	3.07E-07
heart development (GO:0007507)	597	50	24.83	+	2.01	8.97E-06	4.25E-04
negative regulation of multicellular organismal process (GO:0051241)	1242	104	51.66	+	2.01	8.89E-11	1.27E-08
homeostatic process (GO:0042592)	1494	125	62.15	+	2.01	8.29E-13	1.71E-10
intracellular signal transduction (GO:0035556)	1518	127	63.14	+	2.01	4.78E-13	1.07E-10
negative regulation of molecular function (GO:0044092)	1028	86	42.76	+	2.01	4.13E-09	4.87E-07
regulation of cell development (GO:0060284)	957	80	39.81	+	2.01	2.09E-08	2.06E-06
regulation of ERK1 and ERK2 cascade (GO:0070372)	335	28	13.93	+	2.01	9.77E-04	2.17E-02
positive regulation of signaling (GO:0023056)	1855	155	77.16	+	2.01	7.75E-16	2.83E-13
regulation of peptidase activity (GO:0052547)	455	38	18.93	+	2.01	1.57E-04	4.76E-03
negative regulation of cell differentiation (GO:0045596)	720	60	29.95	+	2	1.45E-06	9.09E-05
response to cytokine (GO:0034097)	830	69	34.53	+	2	2.06E-07	1.58E-05
negative regulation of cell population proliferation (GO:0008285)	734	61	30.53	+	2	1.16E-06	7.50E-05
regulation of developmental process (GO:0050793)	2641	219	109.86	+	1.99	2.58E-22	2.53E-19
positive regulation of secretion by cell (GO:1903532)	387	32	16.1	+	1.99	5.47E-04	1.38E-02
muscle tissue development (GO:0060537)	375	31	15.6	+	1.99	7.22E-04	1.72E-02
cellular response to organic substance (GO:0071310)	1743	144	72.5	+	1.99	2.85E-14	8.77E-12
response to light stimulus (GO:0009416)	328	27	13.64	+	1.98	1.43E-03	2.97E-02
regulation of cell population proliferation (GO:0042127)	1786	147	74.29	+	1.98	2.11E-14	7.04E-12
negative regulation of phosphorus metabolic process (GO:0010563)	438	36	18.22	+	1.98	3.04E-04	8.61E-03
negative regulation of phosphate metabolic process (GO:0045936)	438	36	18.22	+	1.98	3.04E-04	8.60E-03
regulation of transmembrane transport (GO:0034762)	634	52	26.37	+	1.97	1.03E-05	4.74E-04
regulation of localization (GO:0032879)	2330	191	96.92	+	1.97	7.58E-19	3.83E-16
negative regulation of catalytic activity (GO:0043086)	660	54	27.45	+	1.97	9.28E-06	4.34E-04
positive regulation of signal transduction (GO:0009967)	1628	133	67.72	+	1.96	6.28E-13	1.33E-10
response to nitrogen compound (GO:1901698)	959	78	39.89	+	1.96	8.80E-08	7.54E-06
response to peptide hormone (GO:0043434)	320	26	13.31	+	1.95	2.04E-03	3.91E-02
export from cell (GO:0140352)	505	41	21.01	+	1.95	1.35E-04	4.23E-03
positive regulation of intracellular signal transduction (GO:1902533)	1023	83	42.55	+	1.95	3.65E-08	3.44E-06
regulation of cell growth (GO:0001558)	444	36	18.47	+	1.95	3.35E-04	9.35E-03
regulation of multicellular organismal development (GO:2000026)	1579	128	65.68	+	1.95	3.16E-12	5.64E-10
negative regulation of response to stimulus (GO:0048585)	1743	141	72.5	+	1.94	2.10E-13	4.98E-11
regulation of cell communication (GO:0010646)	3490	282	145.17	+	1.94	4.85E-28	2.53E-24
regulation of growth (GO:0040008)	669	54	27.83	+	1.94	1.09E-05	4.97E-04
regulation of neuron projection development (GO:0010975)	570	46	23.71	+	1.94	5.81E-05	2.11E-03
vesicle-mediated transport (GO:0016192)	1240	100	51.58	+	1.94	1.46E-09	1.88E-07
cellular response to stress (GO:0033554)	1551	125	64.52	+	1.94	8.54E-12	1.42E-09
regulation of signaling (GO:0023051)	3487	281	145.05	+	1.94	8.23E-28	3.23E-24
lipid biosynthetic process (GO:0008610)	534	43	22.21	+	1.94	8.90E-05	3.03E-03
regulation of hydrolase activity (GO:0051336)	983	79	40.89	+	1.93	1.25E-07	1.03E-05
negative regulation of protein phosphorylation (GO:0001933)	349	28	14.52	+	1.93	1.95E-03	3.76E-02
secretion (GO:0046903)	561	45	23.34	+	1.93	7.98E-05	2.76E-03
positive regulation of developmental process (GO:0051094)	1498	120	62.31	+	1.93	3.88E-11	5.85E-09

Table S3. Enriched GO categories for genes within each cluster in Fig. 9A.

positive regulation of MAPK cascade (GO:0043410)	537	43	22.34	+	1.93	9.63E-05	3.22E-03
positive regulation of cellular component organization (GO:0051130)	1275	102	53.04	+	1.92	1.52E-09	1.94E-07
positive regulation of multicellular organismal process (GO:0051240)	1876	150	78.04	+	1.92	8.03E-14	2.17E-11
regulation of plasma membrane bounded cell projection organization (GO:0120035)	763	61	31.74	+	1.92	4.41E-06	2.33E-04
negative regulation of protein modification process (GO:0031400)	488	39	20.3	+	1.92	2.60E-04	7.44E-03
regulation of intracellular signal transduction (GO:1902531)	1690	135	70.3	+	1.92	2.36E-12	4.35E-10
regulation of cell projection organization (GO:0031344)	777	62	32.32	+	1.92	3.49E-06	1.92E-04
regulation of hormone levels (GO:0010817)	615	49	25.58	+	1.92	4.55E-05	1.71E-03
epithelium development (GO:0060429)	1194	95	49.67	+	1.91	7.32E-09	8.19E-07
regulation of protein catabolic process (GO:0042176)	365	29	15.18	+	1.91	1.61E-03	3.28E-02
cellular response to oxygen-containing compound (GO:1901701)	1109	88	46.13	+	1.91	3.24E-08	3.09E-06
regulation of MAPK cascade (GO:0043408)	744	59	30.95	+	1.91	8.15E-06	3.89E-04
regulation of signal transduction (GO:0009966)	3015	239	125.41	+	1.91	4.02E-22	3.50E-19
leukocyte differentiation (GO:0002521)	467	37	19.43	+	1.9	4.66E-04	1.21E-02
secretion by cell (GO:0032940)	442	35	18.39	+	1.9	5.40E-04	1.36E-02
positive regulation of organelle organization (GO:0010638)	531	42	22.09	+	1.9	1.96E-04	5.80E-03
regulation of transport (GO:0051049)	1961	155	81.57	+	1.9	6.54E-14	1.80E-11
regulation of secretion (GO:0051046)	772	61	32.11	+	1.9	5.21E-06	2.68E-04
cellular response to chemical stimulus (GO:0070887)	2362	186	98.25	+	1.89	1.35E-16	6.04E-14
neuron development (GO:0048666)	940	74	39.1	+	1.89	6.25E-07	4.32E-05
regulation of biological quality (GO:0065008)	3089	243	128.49	+	1.89	4.06E-22	3.35E-19
negative regulation of phosphorylation (GO:0042326)	382	30	15.89	+	1.89	2.00E-03	3.83E-02
nervous system development (GO:0007399)	2068	162	86.02	+	1.88	2.91E-14	8.78E-12
regulation of cellular component biogenesis (GO:0044087)	1023	80	42.55	+	1.88	2.28E-07	1.72E-05
regulation of membrane potential (GO:0042391)	487	38	20.26	+	1.88	4.17E-04	1.11E-02
positive regulation of cell cycle (GO:0045787)	359	28	14.93	+	1.88	2.38E-03	4.35E-02
regulation of anatomical structure size (GO:0090066)	577	45	24	+	1.87	1.54E-04	4.69E-03
regulation of secretion by cell (GO:1903530)	693	54	28.83	+	1.87	2.52E-05	1.01E-03
protein localization to membrane (GO:0072657)	488	38	20.3	+	1.87	4.27E-04	1.12E-02
response to peptide (GO:1901652)	386	30	16.06	+	1.87	2.11E-03	3.98E-02
negative regulation of cellular component organization (GO:0051129)	760	59	31.61	+	1.87	1.54E-05	6.62E-04
cell surface receptor signaling pathway (GO:0007166)	1908	148	79.37	+	1.86	1.15E-12	2.32E-10
carbohydrate derivative metabolic process (GO:1901135)	919	71	38.23	+	1.86	1.78E-06	1.08E-04
embryonic organ development (GO:0048568)	520	40	21.63	+	1.85	4.09E-04	1.09E-02
inflammatory response (GO:0006954)	535	41	22.25	+	1.84	3.41E-04	9.40E-03
behavior (GO:0007610)	744	57	30.95	+	1.84	2.90E-05	1.14E-03
regulation of multicellular organismal process (GO:0051239)	3264	250	135.77	+	1.84	2.25E-21	1.53E-18
cell-cell signaling (GO:0007267)	914	70	38.02	+	1.84	3.69E-06	2.01E-04
system development (GO:0048731)	3484	266	144.92	+	1.84	1.06E-22	1.38E-19
response to organic substance (GO:0010033)	2609	199	108.53	+	1.83	1.89E-16	7.80E-14
regulation of cellular component organization (GO:0051128)	2598	198	108.07	+	1.83	2.56E-16	1.03E-13
transmembrane transport (GO:0055085)	1026	78	42.68	+	1.83	1.18E-06	7.60E-05
regulation of developmental growth (GO:0048638)	395	30	16.43	+	1.83	2.49E-03	4.49E-02
plasma membrane bounded cell projection organization (GO:0120036)	1213	92	50.46	+	1.82	1.17E-07	9.74E-06
organonitrogen compound catabolic process (GO:1901565)	1084	82	45.09	+	1.82	6.84E-07	4.64E-05
embryonic morphogenesis (GO:0048598)	636	48	26.46	+	1.81	1.50E-04	4.60E-03
locomotion (GO:0040011)	530	40	22.05	+	1.81	6.81E-04	1.63E-02
organic hydroxy compound metabolic process (GO:1901615)	465	35	19.34	+	1.81	1.60E-03	3.27E-02
hemopoiesis (GO:0030097)	771	58	32.07	+	1.81	3.97E-05	1.52E-03

Table S3. Enriched GO categories for genes within each cluster in Fig. 9A.

cell projection organization (GO:0030030)	1267	95	52.7	+	1.8	9.96E-08	8.39E-06
neurogenesis (GO:0022008)	1428	107	59.4	+	1.8	1.54E-08	1.57E-06
regulation of protein-containing complex assembly (GO:0043254)	441	33	18.34	+	1.8	1.92E-03	3.72E-02
positive regulation of molecular function (GO:0044093)	1513	113	62.94	+	1.8	7.66E-09	8.51E-07
regulation of organelle organization (GO:0033043)	1192	89	49.58	+	1.79	3.19E-07	2.35E-05
cell activation (GO:0001775)	804	60	33.44	+	1.79	3.84E-05	1.48E-03
in utero embryonic development (GO:0001701)	539	40	22.42	+	1.78	7.80E-04	1.83E-02
regulation of phosphate metabolic process (GO:0019220)	1397	103	58.11	+	1.77	5.66E-08	5.13E-06
regulation of phosphorus metabolic process (GO:0051174)	1398	103	58.15	+	1.77	7.86E-08	6.85E-06
response to oxygen-containing compound (GO:1901700)	1566	115	65.14	+	1.77	1.31E-08	1.38E-06
chemotaxis (GO:0006935)	504	37	20.96	+	1.76	1.67E-03	3.37E-02
head development (GO:0060322)	752	55	31.28	+	1.76	1.10E-04	3.55E-03
taxis (GO:0042330)	506	37	21.05	+	1.76	1.71E-03	3.43E-02
regulation of proteolysis (GO:0030162)	766	56	31.86	+	1.76	1.19E-04	3.82E-03
epithelial cell differentiation (GO:0030855)	631	46	26.25	+	1.75	4.47E-04	1.17E-02
transport (GO:0006810)	3582	261	149	+	1.75	1.48E-19	7.98E-17
regulation of cell activation (GO:0050865)	716	52	29.78	+	1.75	2.26E-04	6.55E-03
regulation of protein phosphorylation (GO:0001932)	1143	83	47.54	+	1.75	2.73E-06	1.58E-04
regulation of molecular function (GO:0065009)	2589	188	107.69	+	1.75	1.79E-13	4.46E-11
protein catabolic process (GO:0030163)	786	57	32.69	+	1.74	1.04E-04	3.40E-03
lipid metabolic process (GO:0006629)	1228	89	51.08	+	1.74	1.01E-06	6.72E-05
localization within membrane (GO:0051668)	566	41	23.54	+	1.74	1.04E-03	2.29E-02
small molecule metabolic process (GO:0044281)	1603	116	66.68	+	1.74	1.97E-08	1.96E-06
macromolecule catabolic process (GO:0009057)	1023	74	42.55	+	1.74	1.16E-05	5.16E-04
generation of neurons (GO:0048699)	1246	90	51.83	+	1.74	1.14E-06	7.39E-05
positive regulation of phosphorus metabolic process (GO:0010562)	915	66	38.06	+	1.73	3.80E-05	1.47E-03
positive regulation of phosphate metabolic process (GO:0045937)	915	66	38.06	+	1.73	3.80E-05	1.47E-03
neuron differentiation (GO:0030182)	1166	84	48.5	+	1.73	3.37E-06	1.88E-04
cell development (GO:0048468)	2461	177	102.37	+	1.73	1.95E-12	3.78E-10
regulation of response to stimulus (GO:0048583)	4047	291	168.34	+	1.73	2.12E-21	1.51E-18
regulation of mitotic cell cycle (GO:0007346)	501	36	20.84	+	1.73	2.43E-03	4.42E-02
intracellular transport (GO:0046907)	1336	96	55.57	+	1.73	6.00E-07	4.22E-05
positive regulation of catabolic process (GO:0009896)	529	38	22	+	1.73	2.14E-03	4.03E-02
positive regulation of response to stimulus (GO:0048584)	2325	167	96.71	+	1.73	1.19E-11	1.93E-09
carbohydrate derivative biosynthetic process (GO:1901137)	543	39	22.59	+	1.73	1.71E-03	3.43E-02
negative regulation of protein metabolic process (GO:0051248)	1046	75	43.51	+	1.72	1.43E-05	6.24E-04
establishment of localization (GO:0051234)	3752	269	156.07	+	1.72	2.95E-19	1.54E-16
multicellular organism development (GO:0007275)	4273	306	177.74	+	1.72	2.11E-22	2.21E-19
embryo development ending in birth or egg hatching (GO:0009792)	852	61	35.44	+	1.72	9.10E-05	3.06E-03
response to stress (GO:0006950)	3304	236	137.44	+	1.72	1.59E-16	6.94E-14
regulation of phosphorylation (GO:0042325)	1249	89	51.95	+	1.71	2.40E-06	1.42E-04
cellular localization (GO:0051641)	2766	197	115.06	+	1.71	1.86E-13	4.48E-11
localization (GO:0051179)	4361	310	181.4	+	1.71	2.90E-22	2.68E-19
establishment of localization in cell (GO:0051649)	1820	129	75.71	+	1.7	9.92E-09	1.08E-06
organic substance transport (GO:0071702)	2131	151	88.64	+	1.7	4.08E-10	5.56E-08
positive regulation of protein metabolic process (GO:0051247)	1454	103	60.48	+	1.7	4.03E-07	2.92E-05
animal organ development (GO:0048513)	2939	208	122.25	+	1.7	5.49E-14	1.54E-11
chordate embryonic development (GO:0043009)	835	59	34.73	+	1.7	1.61E-04	4.88E-03
embryo development (GO:0009790)	1233	87	51.29	+	1.7	4.28E-06	2.28E-04

Table S3. Enriched GO categories for genes within each cluster in Fig. 9A.

response to lipid (GO:0033993)	711	50	29.58	+	1.69	6.32E-04	1.56E-02
regulation of cellular response to stress (GO:0080135)	669	47	27.83	+	1.69	8.95E-04	2.05E-02
regulation of catalytic activity (GO:0050790)	1812	127	75.37	+	1.68	2.68E-08	2.61E-06
positive regulation of protein phosphorylation (GO:0001934)	772	54	32.11	+	1.68	3.84E-04	1.04E-02
cellular lipid metabolic process (GO:0044255)	917	64	38.14	+	1.68	1.15E-04	3.69E-03
positive regulation of transcription by RNA polymerase II (GO:0045944)	1264	88	52.58	+	1.67	5.67E-06	2.87E-04
negative regulation of cellular process (GO:0048523)	4860	336	202.16	+	1.66	9.98E-23	1.42E-19
positive regulation of catalytic activity (GO:0043085)	1085	75	45.13	+	1.66	4.02E-05	1.53E-03
regulation of protein kinase activity (GO:0045859)	608	42	25.29	+	1.66	2.18E-03	4.09E-02
protein localization (GO:0008104)	1970	136	81.95	+	1.66	1.64E-08	1.66E-06
positive regulation of cellular process (GO:0048522)	5698	393	237.02	+	1.66	1.10E-27	3.46E-24
cellular macromolecule localization (GO:0070727)	1976	136	82.19	+	1.65	1.77E-08	1.79E-06
regulation of protein modification process (GO:0031399)	1498	103	62.31	+	1.65	1.59E-06	9.78E-05
regulation of response to external stimulus (GO:0032101)	1122	77	46.67	+	1.65	3.85E-05	1.47E-03
cellular developmental process (GO:0048869)	3809	261	158.44	+	1.65	2.61E-16	1.02E-13
positive regulation of phosphorylation (GO:0042327)	834	57	34.69	+	1.64	4.52E-04	1.18E-02
cell differentiation (GO:0030154)	3779	258	157.19	+	1.64	6.49E-16	2.42E-13
intracellular protein transport (GO:0006886)	674	46	28.04	+	1.64	1.83E-03	3.63E-02
catabolic process (GO:0009056)	1922	131	79.95	+	1.64	6.29E-08	5.64E-06
organic substance catabolic process (GO:1901575)	1658	113	68.97	+	1.64	6.96E-07	4.70E-05
positive regulation of biological process (GO:0048518)	6398	433	266.13	+	1.63	7.02E-30	5.50E-26
macromolecule localization (GO:0033036)	2386	161	99.25	+	1.62	2.75E-09	3.34E-07
establishment of protein localization (GO:0045184)	1264	85	52.58	+	1.62	2.92E-05	1.14E-03
protein transport (GO:0015031)	1164	78	48.42	+	1.61	7.20E-05	2.54E-03
negative regulation of biological process (GO:0048519)	5547	371	230.74	+	1.61	4.43E-23	6.95E-20
phosphorylation (GO:0016310)	883	59	36.73	+	1.61	6.35E-04	1.56E-02
regulation of kinase activity (GO:0043549)	704	47	29.28	+	1.6	2.32E-03	4.27E-02
regulation of catabolic process (GO:0009894)	976	65	40.6	+	1.6	3.46E-04	9.50E-03
central nervous system development (GO:0007417)	935	62	38.89	+	1.59	6.43E-04	1.58E-02
regulation of cell cycle (GO:0051726)	1072	71	44.59	+	1.59	2.47E-04	7.12E-03
nitrogen compound transport (GO:0071705)	1576	104	65.56	+	1.59	7.17E-06	3.47E-04
regulation of response to stress (GO:0080134)	1501	99	62.44	+	1.59	1.50E-05	6.47E-04
anatomical structure development (GO:0048856)	5436	357	226.12	+	1.58	1.13E-20	6.57E-18
positive regulation of protein modification process (GO:0031401)	990	65	41.18	+	1.58	5.10E-04	1.31E-02
positive regulation of DNA-templated transcription (GO:0045893)	1604	105	66.72	+	1.57	8.61E-06	4.10E-04
response to external stimulus (GO:0009605)	2393	156	99.54	+	1.57	4.98E-08	4.59E-06
positive regulation of RNA biosynthetic process (GO:1902680)	1611	105	67.01	+	1.57	1.17E-05	5.19E-04
developmental process (GO:0032502)	5810	376	241.68	+	1.56	5.68E-21	3.43E-18
regulation of protein metabolic process (GO:0051246)	2512	162	104.49	+	1.55	5.22E-08	4.79E-06
positive regulation of macromolecule biosynthetic process (GO:0010557)	1819	117	75.66	+	1.55	6.38E-06	3.17E-04
cellular component organization (GO:0016043)	5371	345	223.42	+	1.54	2.74E-18	1.34E-15
positive regulation of biosynthetic process (GO:0009891)	1993	128	82.9	+	1.54	2.14E-06	1.27E-04
response to chemical (GO:0042221)	4018	258	167.14	+	1.54	6.62E-13	1.38E-10
positive regulation of cellular biosynthetic process (GO:0031328)	1920	123	79.87	+	1.54	3.55E-06	1.94E-04
organelle organization (GO:0006996)	2976	190	123.79	+	1.53	3.77E-09	4.51E-07
regulation of immune system process (GO:0002682)	1602	101	66.64	+	1.52	6.18E-05	2.21E-03
cellular catabolic process (GO:0044248)	1017	64	42.3	+	1.51	1.81E-03	3.61E-02
cellular component organization or biogenesis (GO:0071840)	5582	347	232.19	+	1.49	3.74E-16	1.43E-13
positive regulation of nitrogen compound metabolic process (GO:0051173)	3060	189	127.29	+	1.48	5.27E-08	4.80E-06

Table S3. Enriched GO categories for genes within each cluster in Fig. 9A.

phosphate-containing compound metabolic process (GO:0006796)	1764	108	73.38	+	1.47	9.87E-05	3.28E-03
positive regulation of RNA metabolic process (GO:0051254)	1750	107	72.79	+	1.47	1.19E-04	3.82E-03
phosphorus metabolic process (GO:0006793)	1787	109	74.33	+	1.47	1.08E-04	3.51E-03
cellular component assembly (GO:0022607)	2279	139	94.8	+	1.47	1.03E-05	4.73E-04
positive regulation of macromolecule metabolic process (GO:0010604)	3507	213	145.88	+	1.46	2.06E-08	2.04E-06
positive regulation of nucleobase-containing compound metabolic process (GO:0045935)	1953	118	81.24	+	1.45	7.64E-05	2.67E-03
positive regulation of metabolic process (GO:0009893)	3858	232	160.48	+	1.45	6.85E-09	7.78E-07
cellular response to stimulus (GO:0051716)	6786	407	282.27	+	1.44	2.82E-17	1.34E-14
cell communication (GO:0007154)	5495	329	228.57	+	1.44	5.76E-13	1.24E-10
positive regulation of cellular metabolic process (GO:0031325)	2958	177	123.04	+	1.44	1.36E-06	8.66E-05
organic substance biosynthetic process (GO:1901576)	2349	140	97.71	+	1.43	2.79E-05	1.11E-03
biosynthetic process (GO:0009058)	2414	143	100.41	+	1.42	2.77E-05	1.11E-03
negative regulation of cellular metabolic process (GO:0031324)	2179	127	90.64	+	1.4	1.67E-04	5.02E-03
signaling (GO:0023052)	5328	310	221.63	+	1.4	1.43E-10	2.00E-08
signal transduction (GO:0007165)	5050	293	210.06	+	1.39	8.85E-10	1.20E-07
negative regulation of metabolic process (GO:0009892)	3033	173	126.16	+	1.37	2.74E-05	1.09E-03
organonitrogen compound metabolic process (GO:1901564)	4671	265	194.3	+	1.36	7.00E-08	6.17E-06
protein modification process (GO:0036211)	2329	132	96.88	+	1.36	3.89E-04	1.05E-02
negative regulation of nitrogen compound metabolic process (GO:0051172)	2383	135	99.12	+	1.36	3.58E-04	9.81E-03
cellular component biogenesis (GO:0044085)	2526	142	105.07	+	1.35	3.37E-04	9.38E-03
multicellular organismal process (GO:0032501)	7636	423	317.63	+	1.33	2.18E-12	4.12E-10
protein metabolic process (GO:0019538)	3702	205	153.99	+	1.33	2.36E-05	9.60E-04
response to stimulus (GO:0050896)	8887	492	369.67	+	1.33	1.62E-15	5.77E-13
regulation of cellular process (GO:0050794)	11419	621	474.99	+	1.31	1.11E-21	8.66E-19
negative regulation of macromolecule metabolic process (GO:0010605)	2796	151	116.3	+	1.3	1.23E-03	2.64E-02
regulation of biological process (GO:0050789)	12257	657	509.85	+	1.29	1.06E-22	1.28E-19
regulation of metabolic process (GO:0019222)	6630	354	275.79	+	1.28	6.71E-08	5.94E-06
biological regulation (GO:0065007)	12654	672	526.36	+	1.28	1.23E-22	1.38E-19
primary metabolic process (GO:0044238)	6922	367	287.93	+	1.27	7.65E-08	6.70E-06
regulation of nitrogen compound metabolic process (GO:0051171)	5498	290	228.7	+	1.27	7.76E-06	3.72E-04
regulation of primary metabolic process (GO:0080090)	5708	300	237.43	+	1.26	6.91E-06	3.38E-04
metabolic process (GO:0008152)	8033	422	334.15	+	1.26	5.94E-09	6.80E-07
regulation of cellular metabolic process (GO:0031323)	5280	277	219.63	+	1.26	2.45E-05	9.89E-04
cellular process (GO:0009987)	14994	781	623.7	+	1.25	1.22E-31	1.91E-27
organic substance metabolic process (GO:0071704)	7643	397	317.92	+	1.25	1.30E-07	1.06E-05
regulation of biosynthetic process (GO:0009889)	3977	205	165.43	+	1.24	1.21E-03	2.61E-02
cellular metabolic process (GO:0044237)	5498	281	228.7	+	1.23	1.38E-04	4.31E-03
regulation of cellular biosynthetic process (GO:0031326)	3839	196	159.69	+	1.23	2.57E-03	4.62E-02
regulation of macromolecule metabolic process (GO:0060255)	6107	310	254.03	+	1.22	7.79E-05	2.70E-03
nitrogen compound metabolic process (GO:0006807)	6311	319	262.52	+	1.22	7.84E-05	2.71E-03
biological_process (GO:0008150)	20726	898	862.13	+	1.04	1.21E-08	1.30E-06
G protein-coupled receptor signaling pathway (GO:0007186)	1878	38	78.12	-	0.49	4.88E-07	3.49E-05
sensory perception (GO:0007600)	1657	33	68.93	-	0.48	1.55E-06	9.64E-05
detection of stimulus (GO:0051606)	799	13	33.24	-	0.39	1.12E-04	3.63E-03
RNA processing (GO:0006396)	810	13	33.69	-	0.39	8.48E-05	2.90E-03
Unclassified (UNCLASSIFIED)	1271	17	52.87	-	0.32	1.21E-08	1.30E-06
defense response to bacterium (GO:0042742)	404	5	16.81	-	0.3	1.94E-03	3.76E-02
ncRNA metabolic process (GO:0034660)	496	6	20.63	-	0.29	3.13E-04	8.77E-03
ribonucleoprotein complex biogenesis (GO:0022613)	415	5	17.26	-	0.29	1.43E-03	2.97E-02

Table S3. Enriched GO categories for genes within each cluster in Fig. 9A.

detection of stimulus involved in sensory perception (GO:0050906)	700	7	29.12	-	0.24	2.06E-06	1.25E-04
ncRNA processing (GO:0034470)	392	3	16.31	-	0.18	1.40E-04	4.36E-03
peptide biosynthetic process (GO:0043043)	400	3	16.64	-	0.18	1.48E-04	4.55E-03
immunoglobulin mediated immune response (GO:0016064)	286	2	11.9	-	0.17	1.23E-03	2.63E-02
B cell mediated immunity (GO:0019724)	291	2	12.1	-	0.17	1.29E-03	2.73E-02
translation (GO:0006412)	376	2	15.64	-	0.13	5.01E-05	1.87E-03
production of molecular mediator of immune response (GO:0002440)	316	1	13.14	-	0.08	6.39E-05	2.28E-03
detection of chemical stimulus (GO:0009593)	642	2	26.71	-	0.07	2.22E-09	2.76E-07
immunoglobulin production (GO:0002377)	251	0	10.44	-	< 0.01	6.12E-05	2.20E-03
detection of chemical stimulus involved in sensory perception of smell (GO:0050911)	553	0	23	-	< 0.01	3.09E-10	4.25E-08
detection of chemical stimulus involved in sensory perception (GO:0050907)	611	0	25.42	-	< 0.01	1.69E-11	2.65E-09
macromolecule methylation (GO:0043414)	196	0	8.15	-	< 0.01	6.68E-04	1.61E-02
sensory perception of smell (GO:0007608)	1157	0	48.13	-	< 0.01	2.02E-21	1.51E-18
sensory perception of chemical stimulus (GO:0007606)	1258	0	52.33	-	< 0.01	1.92E-23	3.76E-20

Table S4. Genes associated with Ca^{2+} dynamics within Cluster 1 in Fig. 9A that were most upregulated in *Trpm3*-M/M lenses.

unique	ensembl ge	entrezgene	external ge	gene biotyp	external ge	transcript c	description	p7.wt 1	p7.wt 2	p7.wt 3	p7.null 1	p7.null 2	p7.null 3	p7.im 1	p7.im 2	p7.im 3	p7.mm 1	p7.mm 2	p7.mm 3	p7.wt 1.cpm	p7.wt 2.cpm	p7.wt 3.cpm	p7.null 1.cp	p7.null 2.cp	p7.null 3.cp	p7.im 1.cpm	p7.im 2.cpm	p7.im 3.cpm	p7.mm 1.cp	p7.mm 2.cp	p7.mm 3.cp	logFC.Null.W	logCPM.Null	PValue.Null.	FDR.Null.WT	logFC.IM.WT	logCPM.IM.W	PValue.IM.W	FDR.IM.WT.F	logFC.MM.W	logCPM.MM	PValue.MM.	FDR.MM.WT	ca
76	ENSMUSG00	14526	Gcg	protein cod	MGI Symbol	3	glucagon [Sc	2862	4564	1965	1610	1458	1621	674	684	895	619	880	835	50.850923	79.279527	34.932362	26.465823	23.866211	28.420689	9.1598952	8.3185882	13.026861	9.440342	11.890329	11.986662	-1.068083	4.6722835	5.29273686	1.52366667	-2.43946	4.6722835	1.22598834	5.29404059	-2.307541	4.6722835	1.05791501	8.66395916	1
11177	ENSMUSG00	19736	Rgs4	protein cod	MGI Symbol	3	regulator of	1506	1422	1360	1535	1528	1552	775	686	621	504	478	302	26.758033	24.701027	24.177105	25.232943	25.012051	27.210925	10.53252	8.3429116	9.0387495	7.686482	6.4586107	4.3352958	0.0341421	4.0464031	0.7203254	0.9477493	-1.438751	4.0464031	7.83982132	7.52306086	-2.034409	4.0464031	6.32883711	2.86304536	1
10702	ENSMUSG00	18605	Enpp1	protein cod	MGI Symbol	5	ectonucleoti	237	275	267	211	189	192	244	297	198	115	142	122	4.2109255	4.7769215	4.7465347	3.4685023	3.0937681	3.3663	3.3160451	3.6120186	2.8819201	1.75386	1.9186668	1.7513446	-0.468369	1.6971968	9.38031313	0.0025461	-0.481493	1.6971968	3.49892216	0.0002187	-1.338094	1.6971968	4.05158244	3.82224759	1
14945	ENSMUSG00	14609	Gja1	protein cod	MGI Symbol	8	gap junction	2156	1887	1946	1976	2094	2262	1175	1441	1012	1440	2285	1675	38.306985	32.778367	34.594593	32.482277	34.276986	39.659222	15.96866	17.524979	14.729814	21.961377	30.874321	24.045101	0.0098216	4.8104337	0.917191	0.9915152	-1.130794	4.8104337	1.35125413	7.29370128	-0.458026	4.8104337	1.00472000	1.90363782	1
1861	ENSMUSG00	59091	Jph2	protein cod	MGI Symbol	2	junctophilin	412	415	488	358	351	373	227	243	176	387	459	339	7.3202587	7.2088088	8.6753143	5.884947	5.7455693	6.5397391	3.0850092	2.9552879	2.5617068	5.9021201	6.2018877	4.8664413	-0.353327	2.4731982	0.0010412	0.0208678	-1.428532	2.4731982	2.01904617	1.30482575	-0.450563	2.4731982	2.42634861	3.96803440	1
14263	ENSMUSG00	14062	F2r	protein cod	MGI Symbol	1	coagulation	2118	1814	2273	1891	2027	2050	1284	1265	989	1993	3477	3205	37.631815	31.510311	40.407765	31.085013	33.180253	35.942266	17.450008	15.384524	14.395045	30.395156	46.980313	46.008685	-0.128664	4.9858969	0.1689366	0.6268354	-1.213386	4.9858969	3.50495765	2.36149629	0.1723505	4.9858969	0.0640188	0.0747424	1
9003	ENSMUSG00	19228	Pth1r	protein cod	MGI Symbol	6	parathyroid	217	215	164	103	94	114	276	250	213	327	410	374	3.8555732	3.7346841	2.9154745	1.6931551	1.5386995	1.9987406	3.7509363	3.0404197	3.1002474	4.9870627	5.5398126	5.3688762	-1.008569	1.8147213	6.08172250	6.28003954	-0.088092	1.8147213	0.4779587	0.5667966	0.5978014	1.8147213	2.43727105	4.97081902	1
12139	ENSMUSG00	66092	Ghitm	protein cod	MGI Symbol	4	growth horn	3811	3811	3821	3557	3595	3468	6986	7626	6511	8391	9975	9818	67.712393	66.199447	67.927	58.471387	58.84707	60.803794	94.942178	92.744962	94.768595	127.97077	134.77959	140.94018	-0.180358	6.4747962	0.049355	0.3541817	0.4847931	6.4747962	1.10306143	1.17084733	1.0000598	6.4747962	1.13793544	1.15619109	1
539	ENSMUSG00	109552	Sri	protein cod	MGI Symbol	7	sorcin [Sour	1978	2267	2041	2458	2552	2436	3501	3755	2977	4645	5631	5269	35.144349	39.379204	36.283435	40.405586	41.774053	42.709931	47.579812	45.667104	43.330688	70.840692	76.084596	75.637991	0.1724871	5.6336912	0.0637175	0.3977113	0.3016913	5.6336912	0.001126	0.004336	1.0061401	5.6336912	2.10584794	2.10806696	1
3497	ENSMUSG00	13618	Ednrb	protein cod	MGI Symbol	3	endothelin r	2907	2151	2563	3067	3353	3044	3967	3692	3935	5452	6921	6532	51.650466	37.364212	45.563177	50.416571	54.885737	53.369881	53.912914	44.900918	57.274524	83.148214	93.514738	93.768715	0.2377024	5.9080884	0.0101049	0.1263114	0.2135799	5.9080884	0.0203828	0.0456474	1.0069973	5.9080884	1.15031216	1.16627167	1
11361	ENSMUSG00	217124	Ppp1r9b	protein cod	MGI Symbol	3	protein phos	1415	1294	1262	1488	1473	1417	2236	2461	2144	3077	3462	3304	25.14118	22.477587	22.434932	24.460338	24.111748	24.843995	30.38802	29.929891	31.206246	46.927193	46.777637	47.429858	0.0676209	4.9739151	0.4788286	0.8651334	0.3855849	4.9739151	4.25947802	0.0002577	1.0105193	4.9739151	6.63664908	6.46647859	1
7829	ENSMUSG00	13400	Dmpk	protein cod	MGI Symbol	19	dystrophia n	330	261	260	288	326	316	501	561	444	630	799	691	5.863314	4.5337328	4.6220937	4.734259	5.3363407	5.5403688	6.8087648	6.8227018	6.4624875	9.6081025	10.795879	9.9195012	0.0554128	2.7715528	0.6250004	0.9189759	0.4207566	2.7715528	0.0001146	0.0006197	1.0143984	2.7715528	1.37051522	9.92370018	1
4598	ENSMUSG00	18596	Pdgfrb	protein cod	MGI Symbol	2	platelet deri	703	623	481	964	920	1018	947	1084	1051	1254	1733	1524	12.490636	10.821899	8.5508733	15.846617	15.059612	17.848403	12.87006	13.18326	15.297465	19.124699	23.415842	21.877453	0.6129928	3.9627559	7.15817443	4.88231471	0.3750322	3.9627559	0.0001577	0.0008151	1.0159746	3.9627559	4.55094313	3.98837267	1
8507	ENSMUSG00	13666	Eif2ak3	protein cod	MGI Symbol	5	eukaryotic tr	214.92	208.99	214.95	238	191.98	193.92	353	375.97	315	461.98	585.94	548.9	3.8186165	3.6302867	3.8212271	3.9123391	3.1425481	3.399963	4.7973932	4.5724264	4.5848729	7.0456368	7.9170677	7.8796154	-0.107849	2.3065461	0.384688	0.8203223	0.3081334	2.3065461	0.0079084	0.0212052	1.0198086	2.3065461	8.05709243	5.18712221	1
360	ENSMUSG00	27061	Bcap31	protein cod	MGI Symbol	1	B cell recept	1027	1086	1025	1158	1176	1202	2090	2090	1647	2316	2853	2709	18.247344	18.864497	18.221715	19.035666	19.250112	21.074441	28.403829	25.417909	23.972336	35.321215	38.548988	38.888464	0.1010276	4.6738794	0.2955887	0.7583646	0.4915562	4.6738794	2.45603636	2.47432035	1.0270542	4.6738794	3.15465325	3.12830959	1
3770	ENSMUSG00	66168	Grina	protein cod	MGI Symbol	5	glutamate re	3144	2761	3013	3156	3219	3128	5013	5328	4731	7131	7866	7635	55.861392	47.960292	53.562955	51.879588	52.692272	54.842638	68.128419	64.797424	68.860425	108.75457	106.28333	109.60259	0.0184858	6.1365695	0.8422103	0.9794342	0.358483	6.1365695	9.32818902	0.0005155	1.0445569	6.1365695	8.71362337	9.76172429	1
17819	ENSMUSG00	236266	Alms1	protein cod	MGI Symbol	5	ALMS1, cent	231.28	190.34	219.04	223.14	276.47	249.24	366.95	436.69	433.71	519.59	572.36	549.11	4.1092947	3.3063245	3.8939361	3.6680644	4.525577	4.3698782	4.9869785	5.3108835	6.312715	7.9242444	7.7335783	7.88263	0.1517318	2.434499	0.2063716	0.678619	0.5529035	2.434499	1.14504323	1.00908262	1.0576384	2.434499	3.07884558	2.16659504	1
148	ENSMUSG00	14265	Fmr1	protein cod	MGI Symbol	13	fragile X me	589	481	549.07	518.01	613.03	599	982.03	1045.16	883	1204.03	1469.03	1489	10.465127	8.35527	9.760973	8.5152553	10.034776	10.502155	13.34613	12.7109	12.852199	18.362609	19.849148	21.375018	0.0233005	3.7108362	0.8257539	0.975947	0.4451759	3.7108362	8.94624038	6.47290812	1.0603343	3.7108362	9.44768383	9.16782394	1
1070	ENSMUSG00	15114	Hap1	protein cod	MGI Symbol	5	huntingtin-a	377	260	284	295	311	302	695	703	587	705	790	884	6.6983921	4.5163622	5.0487485	4.8493278	5.0908035	5.2949094	9.4452925	8.5496601	8.5438743	10.751924	10.674273	12.690071	-0.09404	2.9578975	0.4149444	0.838693	0.7067946	2.9578975	2.78625565	4.59538692	1.0691684	2.9578975	2.22366052	1.86614619	1
11557	ENSMUSG00	22393	Wfs1	protein cod	MGI Symbol	3	wolframin E	1253	1171	1200	1363	1442	1477	2403	2415	1947	2667	3461	3364	22.262826	20.341	21.33274	22.405538	23.604305	25.895964	32.657608	29.370454	28.338881	40.6743	46.764125	48.291175	0.1692377	4.918917	0.0762877	0.4386019	0.4992316	4.918917	1.28265604	1.35091268	1.08622	4.918917	1.31578212	1.53750678	1
3964	ENSMUSG00	11820	App	protein cod	MGI Symbol	11	amyloid beta	19357.98	17745.96	19338.99	21172.99	22610.98	21514.97	33990.96	35848.99	28063	46719.99	52964.97	47968.98	343.9452	308.25839	343.7947	348.05007	370.12237	377.21794	461.94901	435.9839	408.46123	712.52453	715.6488	688.60833	0.1372282	8.8443479	0.1272997	0.5504537	0.3913919	8.8443479	1.38179050	9.54691622	1.0876602	8.8443479	5.15187811	6.55191995	1
11616	ENSMUSG00	16509	Kcne1	protein cod	MGI Symbol	2	potassium v	145	147	160	194	171	188	415	457	354	357	417	409	2.5763046	2.5534817	2.8443653	3.1890495	2.7991235	3.2961688	5.6399948	5.5578872	5.1525238	5.4445914	5.6343947	5.8713111	0.2184725	2.1043858	0.0913231	0.4773794	1.0364901	2.1043858	2.97403981	8.82918069	1.0878801	2.1043858	7.41982623	4.79186602	1
5928	ENSMUSG00	16410	Itgav	protein cod	MGI Symbol	9	integrin alph	2991	2705	3514.34	2951.65	3580.89	2980.22	4748.96	6003	4545.11	7821	8377.1	8465	53.142946	46.987537	62.475418	48.520402	58.616101	52.251639	64.540023	73.006557	66.154767	119.27773	113.18918	121.51748	-0.028718	6.1976658	0.7558131	0.9586225	0.3253016	6.1976658	0.0003899	0.001784	1.1223262	6.1976658	4.27968527	5.67043376	1
15007	ENSMUSG00	18795	Plcb1	protein cod	MGI Symbol	8	phospholipa	158	147	163	169	191	197	299	307	270	436.99	378.98	438.99	2.8072837	2.5534817	2.8976972	2.7780895	3.1265064	3.4539641	4.0635143	3.7336354	3.9298911	6.6645154	5.1206785	6.3018261	0.1791019	2.0057841	0.1623529	0.614973	0.5050714	2.0057841	3.46999265	0.0002174	1.1286945	2.0057841	1.08941713	7.93670457	1
8446	ENSMUSG00	14182	Fgfr1	protein cod	MGI Symbol	19	fibroblast gr	2778.95	2590.99	2315.96	3124	2733	2948.99	4931.88	5303	4172	6396.7	7515.96	6808	49.375323	45.007112	41.171477	51.35356	44.736868	51.704089	67.02597	64.493382	60.724095	97.555792	101.55368	97.730773	0.1246251	6.0104318	0.1777527	0.6369864	0.5041256	6.0104318	4.35106474	4.90915856	1.1308154	6.0104318	2.26358756	3.04159573	1
4787	ENSMUSG00	18797	Plcb3	protein cod	MGI Symbol	10	phospholipa	563	503	511	527	519	491	668	664	593	1233	1556	1495	10.003169	8.7374237	9.0841918	8.6630365	8.4955853	8.608611	9.0783531	8.0753547	8.6312052	18.804429	21.024264	21.46115	-0.110675	3.5595975	0.288317	0.752955	-0.110366	3.5595975	0.2846792	0.3750453	1.1396103	3.5595975	1.35381215	1.58000190	1
8546	ENSMUSG00	14681	Gnao1	protein cod	MGI Symbol	12	guanine nuc	221	214	205	250	219	233	356	405	321	553	611	579	3.9266436	3.7173135	3.6443431	4.1095998	3.5848424	4.0851453	4.8381642	4.9254799	4.6722038	8.4337788	8.2556719	8.3117094	0.0608739	2.4002948	0.613588	0.9148505	0.3554433	2.4002948	0.0020423	0.0070169	1.1466631	2.4002948	6.93351526	6.02087705	1
10181	ENSMUSG00	114774	Pawr	protein cod	MGI Symbol	2	PRKC, apopt	145	103	157	131	136	121	270	274	268	301	401	420	2.5763046	1.7891742	2.7910335	2.1534303	2.2262035	2.1214703	3.6693942	3.3323	3.9007808	4.5905378	5.4182069	6.0292193	-0.136488	1.7900651	0.3229684	0.777949	0.6058157	1.7900651	1.25976966	1.10099464	1.1664904	1.7900651	8.32958825	6.11050875	1
8985	ENSMUSG00	74769	Pik3cb	protein cod	MGI Symbol	3	phosphatidy	169.96	141.88	126.73	189.23	171.74	156.52	299.14	344.17	244.75	366.73	460.88	394.9	3.0197844	2.4645441	2.2529151	3.1106383	2.8112367	2.7442358	4.065417	4.185685	3.5623735	5.5929832	6.2272898	5.6689016	0.1644279	1.9575875	0.2153998	0.6929555	0.6128239	1.9575875	6.81721684	6.33074878	1.1771399	1.9575875	5.75865847	4.50265477	1
9127	ENSMUSG00	252972	Tpcn1	protein cod	MGI Symbol	4	two pore cha	1156	1091	1092	1327	1345	1376	2278	2464	2130	3014	3363	3006	20.539367	18.95135	19.412793	21.813756	22.016498	24.12515	30.958815	29.966376	31.002474	45.966382	45.439975	43.151984	0.2060265	4.8846321	0.031632	0.2749349	0.6420372	4.8846321	1.25678092	2.17080341	1.1917554	4.8846321	2.99604182	4.34540417	1
2599	ENSMUSG00	66109	Tspan13	protein cod	MGI Symbol	3	tetraspanin	130	102	137	114	124	122	287	325	285	334	357	349	2.3097904	1.7718036	2.4354878	1.8739775	2.0297738	2.1390031	3.9004301	3.9525456	4.1482183	5.0938194	4.8236905	5.0099941	-0.108839	1.7537375	0.4439138	0.850865	0.8808083	1.7537375	2.57099319	4.73341770	1.1955993	1.7537375	4.44028378	3.31624967	1
3321	ENSMUSG00	11750	Anxa7	protein cod	MGI Symbol	11	annexin A7 [	474	465	541	480	510	528	884	968	753	1227	1510	1529	8.421851	8.07734	9.6175103	7.8904317	8.348263	9.257325	12.013868	11.772505	10.96003	18.712923	20.402724	21.949229	-0.035199	3.629333	0.7436451	0.9550667	0.4124702	3.629333	4.69086093	0.0002815	1.2256615	3.629333	7.22454292	9.91808638	1
1020	ENSMUSG00	13057	Cyba	protein cod	MGI Symbol	3	cytochrome	225	231	222	266	239	201	363	410	394	611	693	682	3.9977141	4.0126141	3.9465569	4.3726142	3.9122252	3.5240953	4.9332967	4.9862883	5.7347299	9.3183343	9.3636344	9.7903037	-0.016614	2.5196021	0.9018796	0.9896888	0.3867806	2.5196021	0.0006732	0.0028473	1.2513002	2.5196021	5.20581269	5.90862850	1
1471	ENSMUSG00	63873	Trpv4	protein cod	MGI Symbol	8	transient rec	159	160	133	172	146	174	553	647	420	416	446	463	2.8250513	2.7792998	2.3643787	2.8274047	2.3898949	3.0507094	7.515463	7.8686061	6.1131639	6.3443978	6.0262351	6.6464965	0.0510262	2.275871	0.7061285	0.9442878	1.4332568	2.275871	1.54347911	9.10748543	1.253564	2.275871	2.35758411	2.25322425	1
7880	ENSMUSG00	17364	Trpm1	protein cod	MGI Symbol	23	transient rec	426	337	343	416	438	411	485	532	407	922	1273	1065	7.5690054	5.8539002	6.0976082	6.8383741	7.1696847	7.205986	6.5913192	6.4700131	5.9239469	14.061382	17.200442	15.288377	0.1201686	3.1578883	0.2686768	0.7390421	-0.038865	3.1578883	0.7192672	0.7812758	1.2546949	3.1578883	1.16550043	1.58628283	1
8295	ENSMUSG00	192176	Flna	protein cod	MGI Symbol	16	filamin, alph	4194	3404	4041	3939	4275	4163	5652	6593	5967	10229	11606	12502	74.517391	59.129603	71.838002	64.750855	69.978087	72.989099	76.812652	80.181948	86.850593	156.00203	156.81723	179.46976	0.0155892	6.5829751	0.8657323	0.9824939	0.246905	6.5829751	0.0068317	0.0189436	1.2605153	6.5829751	1.20565423	2.15295398	1
3743	ENSMUSG00	15205	Hes1	protein cod	MGI Symbol	4	hes family b	222	190	193	199	223	242	354	327	254	571	713	526	3.9444112	3.3004185	3.4310157	3.2712415	3.6503189	4.2429406	4.8109835	3.9768689	3.6970086	8.708296	9.6338692	7.5508794	0.0629664	2.3471351	0.6114622	0.9139633	0.2265736	2.3471351	0.0544673	0.09968	1.2787863	2.3471351	3.02959256	3.48018493	1
9207	ENSMUSG00	14225	Fkbp1a	protein cod	MGI Symbol	10	FK506 bindi	3736	3578	3734	4201	4294	3876	7654	8164	6823	9522	11981	11594	66.379822	62.152092	66.380376	69.057716	70.289101	67.957181	104.02053	99.287945	99.309803	145.21961	161.88413	166.43516	0.0889637	6.6192362	0.3319993	0.7826146	0.6346743	6.6192362	3.88637084	7.04590134	1.2807017	6.6192362	7.00486100	1.30996416	1
3224	ENSMUSG00	15212	Hexb	protein cod	MGI Symbol	2	hexosaminid	1015.77	864.56	1069.56	1224.74	1187.14	1139.52	1937.62	1949.4	1637.22	2724.39	3166.61	2994.2	18.047814	15.017946	19.013871	20.132765	19.432465	19.978991	26.332932	23.707977	23.829986	41.549553	42.786405	42.982591	0.1934105	4.7093438	0.0457055	0.3403169	0.5043925	4.7093438	1.30756033	1.37409548	1.2898242	4.7093438	1.04243519	1.80384960	1
10399	ENSMUSG00	56188	Fxyd1	protein cod	MGI Symbol	17	FXYD domai	594	695	614	776	756	733	1161	1144	974	1592	2136	2068	10.553965	12.072583	10.915252	12.756198	12.375072	12.851552	15.778395	13.91296	14.176718	24.279522	28.861072	29.686727	0.1790718	4.0537363	0.074747	0.4322417	0.3867651	4.0537363	9.10386978	0.0005049	1.3042113	4.0537363	9.81812875	1.65964809	1
6947	ENSMUSG00	20544	Slc9a1	protein cod	MGI Symbol	5	solute carrie	220	218	230	261	248	254	335	424	358	610	777	657	3.908876	3.786796	4.0887752	4.2904222	4.0595475	4.4533344	4.5527669	5.1565518	5.2107445	9.3030833	10.49862	9.4314216	0.1191584	2.5370613	0.3196259	0.7756777	0.340505	2.5370613	0.002901	0.0093805	1.3111837	2.5370613	8.76893289	1.10191617	1
13005	ENSMUSG00	269999	Orai3	protein cod	MGI Symbol	3	ORAI calcium	199	184	215	236	244	224	367	445	348	593	687	570	3.535756	3.1961948	3.8221159	3.8794623	3.9940709	3.92735	4.9876581	5.411947	5.0651929	9.0438171	9.282564	8.1825119	0.1615088	2.4439045	0.1894068	0.6567026	0.5524064	2.4439045	1.81136657	1.53642700	1.3288261	2.4439045	1.70833585	2.12702366	1
11599	ENSMUSG00	53901	Rcan2	protein cod	MGI Symbol	7	regulator of	3493	3335	3809	3735	4033	3642	5078	5572	4901	8884	11751	12754	62.06229	57.93103	67.713672	61.397422	66.016755	63.854503	69.011792	67.764874	71.3348	135.4895	158.77643	183.08729	0.0271566	6.4724296	0.7683006	0.961343	0.148838	6.4724296	0.1037399	0.1670671	1.3466529	6.4724296	3.58670337	7.39125423	1
14610	ENSMUSG00	109305	Orai1	protein cod	MGI Symbol	3	ORAI calcium	264	298	239	324	270	305	539	556	452	784	901	832	4.6906512	5.1764459	4.2487707	5.3260414	4.4196687	5.3475078	7.3251981	6.7618934	6.5789287	11.95675	12.174076	11.943596	0.0952977	2.8577623	0.4110519	0.8370832	0.5494608	2.8577623	4.89096308	4.64177315	1.3529417	2.8577623	1.27501086	1.88964169	1
7652	ENSMUSG00	16438	Itpr1	protein cod	MGI Symbol	13	inositol 1,4,5	1379.91	1495	1451	1491.99	1967.94	1690.96	2553.98	3606.93	2492.93	4271.85	4798.87	4687.93	24.517714	25.969082	25.794838	24.525927	32.213492	29.647285	34.709479	43.866324	36.284975	65.149798	64.841074	67.296566	0.1793718	5.3101268	0.0577862	0.3843113	0.590949	5.3101268	2.91292545	4.25735258	1.370794	5.3101268	4.12843506	8.47087107	1
6429	ENSMUSG00	66815	Mcub	protein cod	MGI Symbol	4	mitochondri	343	358	378	404	395	359	781	921	674	981	1307	1194	6.0942931	6.2186833	6.7198131	6.6411134	6.4658116	6.2942797	10.614063	11.200906	9.8101725	14.961188	17.659842	17.140209	0.027982	3.3349838	0.8067766	0.9712809	0.7334983	3.3349838	1.66871347	3.12062559	1.3869519	3.3349838	4.98335221	8.71857202	1
451	ENSMUSG00	21803	Tgfb1	protein cod	MGI Symbol	5	transforming	543	688	661	584	618	565	925	1157	736	1722	2474	2022	9.6478167	11.950989	11.750784	9.6000252	10.116131	9.9060391	12.571073	14.071062	10.712592	26.262147	33.42804	29.026384	-0.171139	3.9867088	0.0934372	0.4818966	0.1652272	3.9867088	0.0990747	0.1613318	1.4117479	3.9867088	1.92961886	3.87555586	1
8561	ENSMUSG00	64209	Herpud1	protein cod	MGI Symbol	6	homocysteir	1663	1413	1594	1752	1747	1804	3011	3265	3149	4714	5556	5441	29.547549	24.544691	28.33699	28.800076	28.596893	31.629194	40.920541	39.707881	45.834174	71.893008	75.071216	78.1071	0.1109913	5.4492178	0.2395191	0.7180282	0.6172518	5.4492178	3.93222314	6.32083246	1.4492455	5.4492178	1.10812691	2.65168908	1
19662	ENSMUSG00	66395	Ahnak	protein cod	MGI Symbol	5	AHNAK nucl	3966	2633	4026	3382	5107	4314	3741	6394	5367	9738	12157	14066	70.466374	45.736852	71.571343	55.594667	83.597214	75.636553	50.841495	77.761774	78.117501	148.51381	164.2622	201.92142	0.1942587	6.5508865	0.034061	0.2883956	0.1388798	6.5508865	0.1292131	0.1991118	1.4548716	6.5508865	1.08906586	2.75896684	1
22739	ENSMUSG00	19122	Prnp	protein cod	MGI Symbol	2	prion protei	542	540	626	600	628.99	538.99	1178	1290	1170	1727.95	2037	2134	9.6300491	9.3801368	11.128579	9.8630396	10.296027	9.4500106	16.009431	15.688566	17.029528	26.35289	27.52341	30.634176	-0.02508	4.0172125	0.8102973	0.9719604	0.6927589	4.0172125	2.68873980	4.93108651	1.4876045	4.0172125	7.56019977	1.71822722	1
12531	ENSMUSG00	228071	Sestd1	protein cod	MGI Symbol	4	SEC14 and s	162	137	141.98	148	178	126.98	261	284	263	460	565.99	514	2.8783542	2.3797755	2.5240187	2.4328831	2.9137075	2.2263165	3.5470811	3.4539168	3.828005	7.0154399	7.6475086	7.378616	-0.03669	2.0354555	0.8062402	0.9710044	0.4751623	2.0354555	0.000119	0.0006402	1.502389	2.0354555	2.00810218	3.05720684	1
4311	ENSMUSG00	58226	Cacna1h	protein cod	MGI Symbol	10	calcium cha	345.94	299	272	314.98	340	363	463.94	512	474	942	1186	1103.96	6.1465298	5.1938165	4.8354211	5.177767	5.5655087	6.364411	6.3051065	6.2267795	6.8991421	14.366401	16.024921	15.847659	0.0796788	3.0583088	0.4783656	0.8649415	0.2634638	3.0583088	0.0152037	0.0360244	1.5155029	3.0583088	1.59175145	3.29447468	1
2669	ENSMUSG00	11421	Ace	protein cod	MGI Symbol	4	angiotensin	2129.76	2043.19	2226.23	2186.95	2210.09	2110.56	8246.21	7995.08	6635.9	7854.08	7061.24	7595.66	37.840763	35.491485	39.576321	35.949958	36.17728	37.004053	112.06887	97.233594	96.586534	119.78223	95.409625	109.03786	-0.049081	6.1531725	0.5997464	0.9102607	1.4378421	6.1531725	1.12718444	1.21684684	1.5217118	6.1531725	1.07565170	2.93640552	1
16901	ENSMUSG00	53902	Rcan3	protein cod	MGI Symbol	3	regulator of	316	284	322	347	334	310	545	642	500	900	1220	1157	5.6145674	4.9332571	5.7242852	5.7041246	5.4672938	5.4351719	7.4067402	7.8077977	7.277576	13.725861	16.48432	16.609064	0.029887	3.1063926	0.8006742	0.9701843	0.4679813	3.1063926	1.33120742	9.28522065	1.5254276	3.1063926	3.30849849	7.03148448	1
9094	ENSMUSG00	19309	Pygm	protein cod	MGI Symbol	4	muscle glyco	10036	8691	8566	10563.91	10744	10593	22117	24122	21670.93	32326	35327.95	33266	178.31582	150.96809	152.28021	173.65377	175.87007	185.72508	300.57775	293.36401	315.42368	493.00241	477.342	477.54288	0.1524709	8.1359707	0.0917202	0.4774475	0.9171128	8.1359707	5.94623095	2.34394995	1.5881445	8.1359707	1.54247436	4.74223509	1
12359	ENSMUSG00	12257	Tspo	protein cod	MGI Symbol	2	translocator	160	133	151	176	161	155	344	319	288	436	721	580	2.8428189	2.310293	2.6843698	2.8931583	2.6354321	2.7175859	4.67508	3.8795755	4.1918838	6.6494169	9.7419631	8.3260647	0.0740942	2.1878024	0.585729	0.9062583	0.7004597	2.1878024	9.26081371	1.15608055	1.6595288	2.1878024	6.11378011	1.19754455	1
15607	ENSMUSG00	108058	Camk2d	protein cod	MGI Symbol	28	calcium/calr	588	536	463	651	754	704	1133	1238	1150	1806	2246	2198	10.44736	9.3106543	8.2308822	10.701398	12.342334	12.3431	15.397865	15.056158	16.738425	27.543227	30.347363	31.552914	0.3380186	4.0682595	0.0008936	0.0183355	0.7530129	4.0682595	3.92888165	8.46357725	1.6761238	4.0682595	8.04763799	2.41938483	1
12792	ENSMUSG00	104027	Synpo	protein cod	MGI Symbol	12	synaptopodi	1111	1001	1072	1155	1175	1142	2257	2396	1775	3868	4519	4541	19.739824	17.387994	19.057248	18.986351	19.233743	20.022472	30.673418	29.139382	25.835395	58.990699	61.059544	65.187345	0.0518608	5.0103066	0.593363	0.9073765	0.6086203	5.0103066	1.53646442	2.31321902	1.7211609	5.0103066	9.12057897	3.19725093	1
4465	ENSMUSG00	30948	Bin1	protein cod	MGI Symbol	6	bridging inte	772	746.88	793	888	860	863	1733	1929.89	1740.29	2742.74	3671.62	3413.21	13.716601	12.973771	14.097386	14.597299	14.077463	15.130817	23.552075	23.470702	25.330186	41.829408	49.609967	48.997599	0.1028558	4.6385971	0.2994084	0.760326	0.8266477	4.6385971	1.17665130	3.28770216	1.7839042	4.6385971	4.64090507	1.72221086	1
1911	ENSMUSG00	11749	Anxa6	protein cod	MGI Symbol	3	annexin A6 [	1892	1765	2058	2106	2072	2114	5256	6201	5078	7424	8772	8361	33.616334	30.659151	36.585649	34.619269	33.916865	37.064366	71.430874	75.41457	73.911062	113.2231	118.52497	120.02453	0.0661916	6.0237735	0.4798754	0.8655476	1.130157	6.0237735	6.15255833	3.52104242	1.8023213	6.0237735	9.88508168	3.91284483	1
10716	ENSMUSG00	18787	Serpine1	protein cod	MGI Symbol	3	serine (or cy	110	95	113	60	82	87	479	641	706	448	638	410	1.954438	1.6502093	2.008833	0.986304	1.3422697	1.5253547	6.5097771	7.7956361	10.275937	6.8324284	8.6204888	5.8856664	-0.544946	2.2516722	0.0004086	0.0094444	2.1278117	2.2516722	2.68688363	4.05164993	1.9280533	2.2516722	3.69675143	9.19349179	1
4853	ENSMUSG00	14873	Gsto1	protein cod	MGI Symbol	6	glutathione S	288	245	291	308	293	262	507	572	473	1015	1622	1253	5.117074	4.2558028	5.1731895	5.063027	4.796159	4.5935969	6.8903069	6.9564802	6.8845869	15.479721	21.916039	17.987171	-0.008219	3.1494733	0.9400964	0.9971487	0.5116567	3.1494733	2.70469538	2.20933844	1.9300984	3.1494733	2.89221369	1.08600909	1
5985	ENSMUSG00	241556	Tspan18	protein cod	MGI Symbol	5	tetraspanin	258	279	202	289	284	267	695	608	585	1109	1240	1196	4.5840455	4.846404	3.5910112	4.7506974	4.6488367	4.6812609	9.4452925	7.3943007	8.514764	16.913311	16.754555	17.16892	0.1122671	3.1249424	0.3329194	0.7833205	0.959034	3.1249424	1.36521792	4.13043641	1.9639185	3.1249424	5.36805471	1.93168635	1
2635	ENSMUSG00	14226	Fkbp1b	protein cod	MGI Symbol	2	FK506 bindi	134	111	139	137	127	114	315	346	302	542	636	721	2.3808608	1.9281392	2.4710424	2.2520607	2.0788812	1.9987406	4.2809599	4.2079408	4.3956559	8.2660183	8.5934653	10.35016	-0.097209	2.1823031	0.4916874	0.8711141	0.9259837	2.1823031	8.19604325	1.71503107	2.0049762	2.1823031	3.02100113	9.02500339	1
6462	ENSMUSG00	19220	Ptgfr	protein cod	MGI Symbol	3	prostaglandi	504.77	487.85	584.27	566.65	569.71	475.74	1827.14	2012.44	1412	2730.5	3102.26	2880.15	8.9685606	8.4742588	10.386733	9.314819	9.3256646	8.3410602	24.83147	24.474649	20.551875	41.642736	41.916924	41.345371	-0.043825	4.3893206	0.6743601	0.9358476	1.3284726	4.3893206	2.93206350	2.30203332	2.1661534	4.3893206	1.29027586	8.01151681	1
22501	ENSMUSG00	12176	Bnip3	protein cod	MGI Symbol	5	BCL2/adeno	1030.15	990.72	1142.43	908.28	1129.45	1045.72	2144.27	1949.85	1565.11	4216.47	6693.32	6828.78	18.303312	17.209424	20.309302	14.930669	18.488129	18.334413	29.141377	23.713449	22.780414	64.3052	90.438386	98.029076	-0.109317	5.1887713	0.2608114	0.7337405	0.4383238	5.1887713	4.20697832	3.30299951	2.1791948	5.1887713	1.22095351	8.65601375	1
2254	ENSMUSG00	14219	Ccn2	protein cod	MGI Symbol	3	cellular com	824.92	665.49	737.25	811.25	912.79	742.93	3082.48	2671.75	2368.73	3359.99	5264.71	4296.82	14.656864	11.559976	13.106302	13.335652	14.94159	13.025652	41.891979	32.492965	34.477225	51.243061	71.135382	61.682071	0.0714172	4.9680776	0.476297	0.864464	1.4689398	4.9680776	3.08614137	3.25759367	2.2271676	4.9680776	7.94778691	6.04031805	1
3990	ENSMUSG00	54720	Rcan1	protein cod	MGI Symbol	7	regulator of	1166	1196	1021	1318	1140	1327	4541	4648	3533	6103	7391	7212	20.717043	20.775266	18.150606	21.66581	18.660823	23.266042	61.713774	56.527483	51.423352	93.076586	99.865255	103.53031	0.0920359	5.6231805	0.3379275	0.7875353	1.5082279	5.6231805	7.10680028	8.65571829	2.3133414	5.6231805	1.77823868	1.57881004	1
15795	ENSMUSG00	20541	Slc8a1	protein cod	MGI Symbol	6	solute carrie	800	586	743	706	790	696	1545	1882	1536	3785	5239	4775	14.214095	10.179186	13.208522	11.60551	12.931623	12.202838	20.997089	22.888279	22.356714	57.72487	70.787995	68.546481	-0.033001	4.8221786	0.7441802	0.9554955	0.817392	4.8221786	4.11781831	1.09885601	2.3901861	4.8221786	6.50138799	6.30236591	1
5035	ENSMUSG00	13198	Ddit3	protein cod	MGI Symbol	3	DNA-damag	189	174	196	232	205	195	403	342	325	786	1423	1476	3.3580798	3.0224885	3.4843475	3.8137087	3.3556744	3.4188984	5.4769106	4.1592941	4.7304244	11.987252	19.227203	21.188399	0.1028483	2.8856482	0.4172719	0.8400992	0.5403858	2.8856482	4.00174364	3.16277576	2.4105102	2.8856482	3.18695024	1.91620426	1
515	ENSMUSG00	11816	Apoe	protein cod	MGI Symbol	11	apolipoprote	338	259	406	269	250	214	556	557	365	1806	2698	2112	6.005455	4.4989915	7.217577	4.4219294	4.0922858	3.7520219	7.556234	6.7740551	5.3126305	27.543227	36.454669	30.31836	-0.528655	3.6008462	3.54879674	0.0001376	0.1514548	3.6008462	0.1650593	0.2418176	2.4130743	3.6008462	1.11955392	9.32961604	1
17306	ENSMUSG00	238161	Akap6	protein cod	MGI Symbol	2	A kinase (PR	213	159	201	226	228	233	412	525	481	996	1147	1671	3.7845027	2.7619292	3.573234	3.7150783	3.7321647	4.0851453	5.5992238	6.3848813	7.0010282	15.189952	15.497963	23.98768	0.188497	3.0116633	0.120157	0.538237	0.9074355	3.0116633	1.93081966	4.58569671	2.4340729	3.0116633	5.60442544	3.57329138	1
2228	ENSMUSG00	67972	Atp2b1	protein cod	MGI Symbol	11	ATPase, Ca+	220	197	236	194	248	222	806	925	728	1451	1693	1594	3.908876	3.4220129	4.1954389	3.1890495	4.0595475	3.8922844	10.953821	11.249553	10.596151	22.129138	22.875372	22.882323	-0.049078	3.3832528	0.7010838	0.9430758	1.5092618	3.3832528	3.46243592	2.81137959	2.5582145	3.3832528	8.52262671	6.92008152	1
4254	ENSMUSG00	21785	Tff2	protein cod	MGI Symbol	1	trefoil factor	108	109	125	101	90	93	279	334	304	640	1209	922	1.9189028	1.893398	2.2221604	1.6602783	1.4732229	1.6305516	3.7917074	4.0620007	4.4247662	9.760612	16.335691	13.235572	-0.340853	2.4161202	0.0213047	0.2101705	1.0234187	2.4161202	7.19509101	1.78003553	2.7059242	2.4161202	5.29080819	3.89633161	1
14786	ENSMUSG00	16854	Lgals3	protein cod	MGI Symbol	6	lectin, galact	321	336	358	572	637	583	951	1129	742	2561	4467	4911	5.7034054	5.8365296	6.3642674	9.4027644	10.427144	10.22163	12.924422	13.730535	10.799923	39.057699	60.356933	70.4988	0.7468929	4.4224731	1.61539624	1.44776078	1.0662317	4.4224731	6.80094859	2.78487119	3.2465571	4.4224731	8.94925578	2.29778189	1
5630	ENSMUSG00	11931	Atp1b1	protein cod	MGI Symbol	4	ATPase, Na+	659.19	521.53	642.37	673.31	709.57	709.45	5110.76	5378.12	3689.05	7541.29	8248.6	6888.05	11.712236	9.0593014	11.419593	11.068139	11.615053	12.438654	69.457012	65.406968	53.694684	115.01188	111.45292	98.879914	0.1256683	5.6045759	0.2151687	0.6926815	2.550697	5.6045759	1.77419799	8.87098996	3.337349	5.6045759	2.53366544	8.59636488	1
20950	ENSMUSG00	668940	Myh7b	protein cod	MGI Symbol	3	myosin, hea	856	651	520	729	978	839	3185	3716	3366	6870	10728	11522	15.209081	11.308276	9.2441873	11.983593	16.009022	14.71003	43.285261	45.192798	48.992642	104.77407	144.95392	165.40158	0.255841	5.7224041	0.0101748	0.1266853	1.9424256	5.7224041	1.19148421	2.63234884	3.5371156	5.7224041	1.18603949	4.89885876	1
878	ENSMUSG00	29865	Cabp5	protein cod	MGI Symbol	3	calcium binc	353	202	282	423	430	386	1182	864	1132	4543	3772	4742	6.2719692	3.508866	5.0131939	6.9534429	7.0387316	6.7676656	16.063792	10.50769	16.476432	69.285094	50.966276	68.072757	0.4900113	4.4859408	9.99054947	0.0003515	1.5391217	4.4859408	2.68913370	2.60681328	3.6704761	4.4859408	2.49813126	1.07873850	1
9223	ENSMUSG00	18018	Nfatc1	protein cod	MGI Symbol	7	nuclear fact	40	44	32	54	62	52	350	318	264	605	695	760	0.7107047	0.7643074	0.5688731	0.8876736	1.0148869	0.9117063	4.7566221	3.8674138	3.8425602	9.2268286	9.3906579	10.910016	0.4598663	2.0175471	0.0201617	0.2030461	2.6037977	2.0175471	1.41166162	2.06319775	3.8477755	2.0175471	5.98798975	7.90081981	1
5568	ENSMUSG00	381290	Atp2b4	protein cod	MGI Symbol	8	ATPase, Ca+	34	25	32	34	29	16	1057	1128	892	1934	2563	2379	0.604099	0.4342656	0.5688731	0.5589056	0.4747052	0.280525	14.364999	13.718374	12.983196	29.495349	34.630584	34.151221	-0.277771	3.5950808	0.2861884	0.7516196	4.672435	3.5950808	3.85099941	4.57306180	5.9310609	3.5950808	0	0	1
